# Asymmetric α-C(*sp*^3^)−H allylic alkylation of primary alkylamines by synergistic Ir/ketone catalysis

**DOI:** 10.1038/s41467-024-45131-3

**Published:** 2024-01-31

**Authors:** Jianyu Li, Sheng Gong, Shaolun Gao, Jianfeng Chen, Wen-Wen Chen, Baoguo Zhao

**Affiliations:** The Education Ministry Key Lab of Resource Chemistry, Shanghai Frontiers Science Center of Biomimetic Catalysis and Shanghai Normal University, Shanghai, 200234 China

**Keywords:** Organocatalysis, Asymmetric catalysis

## Abstract

Primary alkyl amines are highly reactive in N-nucleophilic reactions with electrophiles. However, their α-C−H bonds are unreactive towards electrophiles due to their extremely low acidity (p*K*_a_ ~57). Nonetheless, 1,8-diazafluoren-9-one (DFO) can activate primary alkyl amines by increasing the acidity of the α-amino C−H bonds by up to 10^44^ times. This makes the α-amino C−H bonds acidic enough to be deprotonated under mild conditions. By combining DFO with an iridium catalyst, direct asymmetric α-C−H alkylation of NH_2_-unprotected primary alkyl amines with allylic carbonates has been achieved. This reaction produces a wide range of chiral homoallylic amines with high enantiopurities. The approach has successfully switched the reactivity between primary alkyl amines and allylic carbonates from intrinsic allylic amination to the α-C−H alkylation, enabling the construction of pharmaceutically significant chiral homoallylic amines from readily available primary alkyl amines in a single step.

## Abstract (web summary)

Primary alkyl amines are highly reactive in N-nucleophilic reactions with electrophiles but their α-C–H bonds are unreactive towards electrophiles due to their extremely low acidity. Combining 8-diazafluoren-9-one with an iridium catalyst, the authors demonstrate direct asymmetric α-C-H alkylation of unprotected primary alkyl amines with allylic carbonates.

## Introduction

**Fig. 1:**
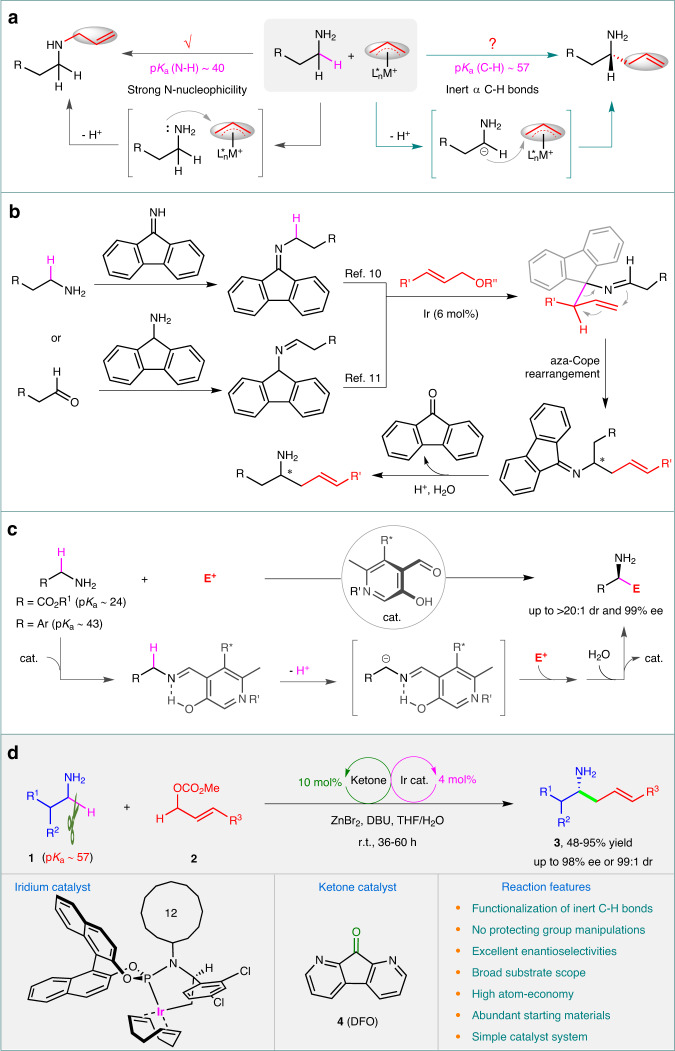
α-C−H functionalization of alkyl amines with electrophiles. **a** N- versus α-C−H allylic alkylation. **b** Allylic alkylation of alkyl amines through protecting group strategy. **c** α-C−H Functionalization of activated primary amines through carbonyl catalysis. **d** This work: asymmetric α-C−H allylic alkylation of primary alkyl amines by synergistic Iridium/ketone catalysis. M metal, L ligand, E^+^ electrophile, cat. catalyst, Me methyl, DBU 1,8-diazabicyclo[5.4.0]undec-7-ene, DFO 1,8-diazafluoren-9-one, THF tetrahydrofuran, ee enantiomeric excess, dr diastereomeric ratio, r.t. room temperature.

Chiral amines are widely found in various natural products, bioactive molecules, pharmaceuticals, materials, and catalysts, making them incredibly important^1,2^. This has spurred the development of innovative methodologies for synthesizing chiral amines^2^. One of the most effective techniques in organic synthesis is transition-metal-catalyzed asymmetric allylic amination, which could serve as a reliable platform for producing chiral amines^3,4^. Primary alkyl amines are a commonly found chemical feedstock. They serve as excellent N-nucleophiles in transition-metal-catalyzed allylic substitution reactions, typically yielding higher-order amines (Fig. 1a)^3,4^. However, if allylic substitution occurs on the α-amino C-H bonds of primary alkyl amines, it would provide a straightforward and intriguing strategy for synthesizing chiral homoallylic amines (Fig. 1a)^5^. These molecules are not only biologically significant (See Supplementary Fig. 1 in Supplementary Information (SI) for selected examples of bioactive chiral homoallylic amines), but they can also serve as powerful building blocks for constructing nitrogen-containing compounds^6^. However, achieving this transformation is difficult. This is because the acidity of the α-amino C(*sp*^3^)−H bond is very low (with a p*K*_a_ estimated to be ~57 by calculation, see SI and Supplementary Data 1)^7^, making it challenging to deprotonate it into an active nucleophilic carbanion^8,9^. Additionally, the unprotected NH_2_ group can seriously interfere with the α-C−H allylic alkylation process (Fig. 1a). To prevent the interruption of the NH_2_ group and facilitate α-C−H deprotonation, protecting group strategies must be employed. The only successful example of such a strategy is reported by Niu et al, where they discover that imines, made from either 9*H*-fluoren-9-imine and an alkyl amine^10^ or fluoren-9-amine and an alkyl aldehyde^11^, can react with allylic electrophiles in the presence of an iridium catalyst (Fig. 1b). This reaction forms linear chiral homoallylic amines upon hydrolysis. The reaction proceeds via a pathway involving an Ir-catalyzed allylic substitution on the fluorenyl carbon and subsequent 2-aza-Cope rearrangement, ultimately resulting in formal α-C(*sp*^3^)−H allylic alkylation of primary amines. The strategy is also effective for activated substrates^12–15^. Although stoichiometric fluorenone or fluoren-9-amine is used, these elegant studies represent an important early advance in the area. If without protecting group manipulations to the active NH_2_ group, direct asymmetric α-C−H allylic alkylation of NH_2_-unprotected primary alkyl amines would be especially synthetically attractive, regarding atom- and step-efficiencies^16–19^. However, developing this transformation is highly challenging and has not yet been achieved.

Carbonyl catalysis is a process that uses an appropriate aldehyde or ketone to catalyze α-C−H functionalization of primary amines with electrophiles, constructing chiral amines in just one step (Fig. 1c)^20–25^. Although a series of asymmetric transformations have been achieved by using chiral pyridoxals as carbonyl catalysts^23^, the strategy is mainly limited to activated primary amines such as glycinates^20,26,27^, benzylamines^28^, and propargylic amines^29^ (Fig. 1c). The reason for this limitation is that primary alkyl amines are highly inert for α-C−H transformation towards electrophiles due to their extremely low acidity (p*K*_a_ ~ 57). Thus, more powerful carbonyl catalysts are required to activate primary alkyl amines. After considering this challenge, we discover that 1,8-diazafluoren-9-one (**4**, DFO)^30^ can effectively activate primary alkyl amines by forming imines. The acidity of the α-amino C−H bonds is improved by up to 10^44^ times, making it acidic enough to be deprotonated under mild conditions (Fig. 1d and Supplementary Fig. 8). The strong electron-withdrawing property of the 14π-electron system of the diazafluorene accounts for its extraordinary activating power. Furthermore, in the presence of an iridium catalyst, allylic carbonates can become active electrophiles for allylic alkylation^31–35^.

Herein, by combining the iridium catalysis and the DFO catalysis^36–45^, we have successfully achieved direct asymmetric α-C−H allylic alkylation of NH_2_-unprotected primary alkyl amines **1** with allylic carbonates **2**, producing a broad variety of chiral homoallylic amines **3** in good yields with excellent diastereo- and enantioselectivities (up to 95% yield, 98% ee or 99:1 dr) (Fig. 1d). The reaction undergoes asymmetric allylic substitution on the diazafluorenyl carbon first and then 2-aza-Cope rearrangement, resulting in formal α-C−H allylic alkylation of primary amines without the protection of the strongly nucleophilic NH_2_ group^46^.

## Results

### Optimization studies

**Fig. 2:**
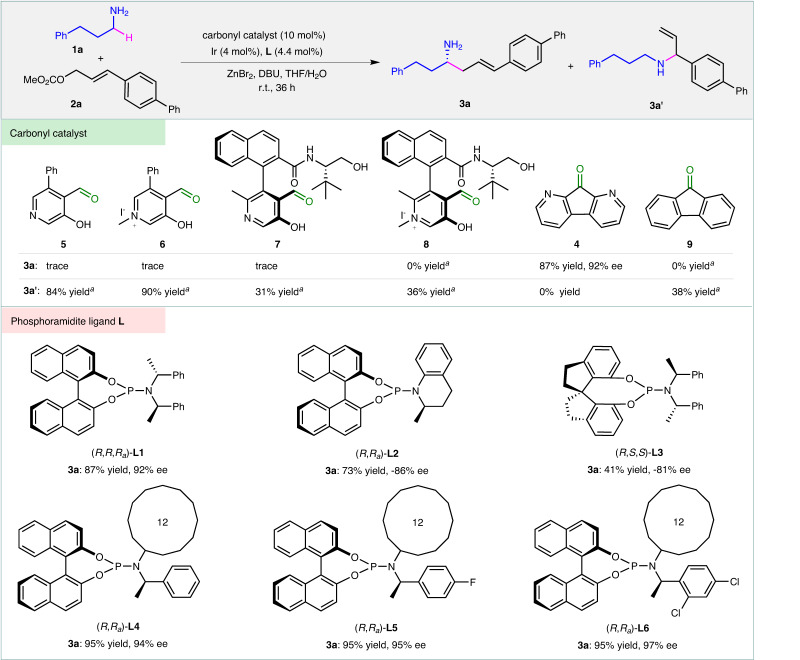
Catalyst screening. Reactions were carried out with **1a** (0.45 mmol), **2a** (0.30 mmol), carbonyl catalyst (0.030 mmol), Ir(COD)_2_BF_4_ (0.012 mmol), phosphoramidite ligand (0.0132 mmol), ZnBr_2_ (0.45 mmol), and DBU (0.33 mmol) in THF/H_2_O (1.45 mL/0.05 mL) at room temperature for 36 h. During the screening of carbonyl catalysts, phosphoramidite (*R*,*R*,*R*_*a*_)-**L1** was used. For the screening of phosphoramide ligands, compound **4** (DFO) was used as the carbonyl catalyst. Isolated yields based on **2a**. The ee values were determined by high-performance liquid chromatography (HPLC). ^*a*^The NMR yields of **3a** and **3a’** were determined by ^1^H NMR analysis of crude reaction mixtures. Ph phenyl, COD 1,5-cyclooctadiene, NMR nuclear magnetic resonance.

The studies began by searching for efficient carbonyl catalysts that could activate primary alkyl amines (Fig. 2 and Supplementary Table 1). When the catalyst Ir(COD)_2_BF_4_/(*R*,*R*,*R*_*a*_)-**L1** was utilized to activate allylic carbonate **2a**, several electron-withdrawing aldehydes **5-8** and ketones **4** and **9** were evaluated in the reaction of **2a** and primary amine **1a**. Only diazafluorenone **4** (DFO) was found to be capable of promoting the desired α-C−H allylic alkylation of **1a**, resulting in the formation of chiral homoallylic amine **3a** in 87% yield with 92% ee. The Ir(COD)_2_BF_4_/(*R*,*R*,*R*_*a*_)-**L1** complex controlled the enantioselectivity of the reaction. When phosphoramidite ligand (*R*,*R*_*a*_)-**L6** was applied, the enantioselectivity was further improved to 97% ee. Without the presence of DFO or iridium catalyst, the α-C−H allylic alkylation did not occur at all (Supplementary Table 1, entries 1 and 2). It was observed that the addition of a small amount of water to the solvent, tetrahydrofuran, was crucial for enhancing the reaction efficiency (Supplementary Table 1, entry 32 vs 24). It was believed that water facilitated the hydrolysis of the imine between **3a** and **4**, thus accelerating the release of alkylation product **3a** and the regeneration of carbonyl catalyst **4** (DFO). In the absence of ZnBr_2_, classic allylic amination occurred predominantly (Supplementary Table 1, entry 3 vs 4). The additive ZnBr_2_ acted as a Lewis acid, promoting the condensation of alkyl amine **1a** with diazafluorenone **4** to form an imine that initiated the transformation. Additionally, ZnBr_2_ also likely inhibited the undesired allylic amination of **1a** by coordinating with the NH_2_ group.

### Scope of the reaction

**Fig. 3:**
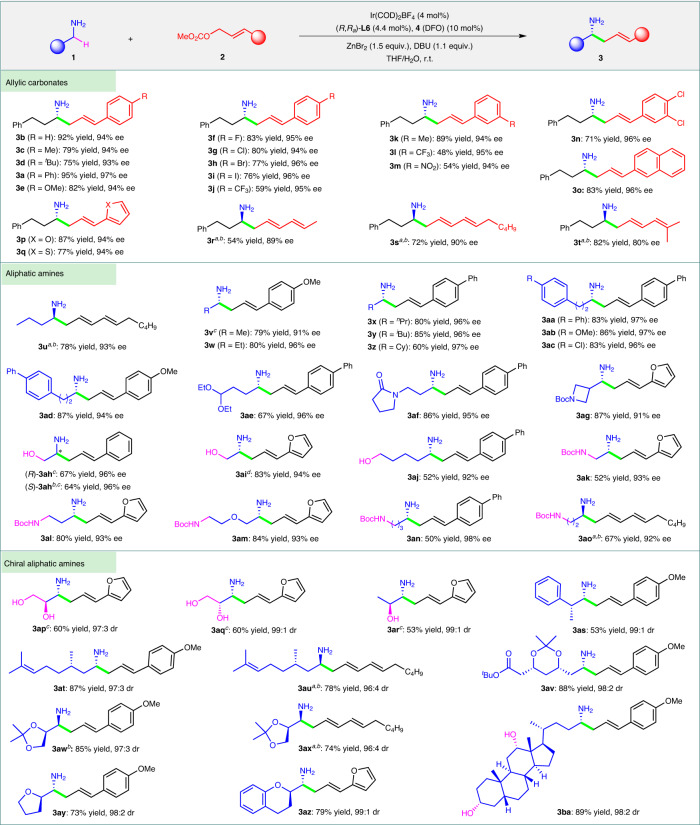
Substrate scope. The reactions were carried out with **1** (0.45 mmol), **2** (0.30 mmol), **4** (0.030 mmol), Ir(COD)_2_BF_4_ (0.012 mmol), (*R*,*R*_*a*_)-**L6** (0.0132 mmol), ZnBr_2_ (0.45 mmol) and DBU (0.33 mmol) in THF/H_2_O (1.45 mL/0.05 mL) at room temperature for 36–60 h unless otherwise stated. Isolated yields were based on carbonates **2**. The ee values were determined by HPLC analysis. The absolute configuration of **3a** was determined based on X-ray analysis (Supplementary Fig. 2) and those of **3b**-**ba** were tentatively assigned by analogy. ^*a*^**4** (0.060 mmol) was used. ^*b*^(*S*,*S*_*a*_)-**L6** was used. ^*c*^Reaction in double scale. ^*d*^Reaction in triple scale. ^*t*^Bu *tert*-butyl, Et ethyl, ^*n*^Pr *n*-propyl, ^*i*^Bu *iso*-butyl, Cy cyclohexyl, Boc *tert* -butoxycarbonyl.

Under the optimized conditions, we investigated the substrate scopes of both reaction partners (Fig. 3). In the presence of catalytic amounts of Ir(COD)_2_BF_4_/(*R*,*R*_*a*_)-**L6** and diazafluorenone **4**, various cinnamyl (for **3a**-**n**), naphthyl (for **3o**), heteroaryl (for **3p-q**), and dienyl carbonates (for **3r**-**t**) smoothly reacted with primary alkyl amine **1a**, producing α-substituted chiral amines **3a**-**t** in moderate to high yields (48-95%) with high enantioselectivities (80-97% ee). However, aliphatic allylic carbonates such as (*E*)-hex-2-en-1-yl methyl carbonate are ineffective for the α allylic alkylation of primary amines, mainly because the corresponding intermediates generated from the Ir-catalyzed allylic substitution cannot smoothly undergo the 2-aza-Cope rearrangement to form the linear homoallylic amines under the standard conditions. The reaction was also applicable to a broad range of primary alkyl amines. Simple alkyl amines such as ethylamine (for **3v**), *n*-propylamine (for **3w**), and *n*-butylamine (for **3u** and **3x**), as well as those bearing diverse substituents (for **3ae**-**ao**), displayed good activity (50–87% yields) and excellent enantioselectivity (91–98% ee) in the reaction. When the smallest primary amine i.e., methylamine was applied to the reaction with (*E*)-3-(4-methoxyphenyl)allyl methyl carbonate (**2b**), a mixture of linear and branched allylic amines, instead of the desired linear homoallylic amines, were obtained as the major products. Secondary amines such as 2-butylamine and cyclohexylamine were inert for the α-C−H allylic alkylation likely due to steric effect and a lower acidity. Primary alkyl amines bearing one or more chiral moieties underwent α-C−H allylic alkylation to afford the corresponding chiral amines **3ap**-**ba** with high diastereoselectivities. Functional groups, such as acetal (for **3ae**), lactam (for **3af**), NHBoc (for **3ak-ao**), OH (for **3ah-aj,** **3ap-ar**, and **3ba**), and ester (**3av**), were all well tolerated by the reaction. It is especially impressive that for amino alcohols (for **3ah-aj,** **3ap-ar**, and **3ba**) containing one or two much more acidic O−H groups, the alkylation still occurred on the highly inert α-C−H bonds.

### Synthetic utility

**Fig. 4:**
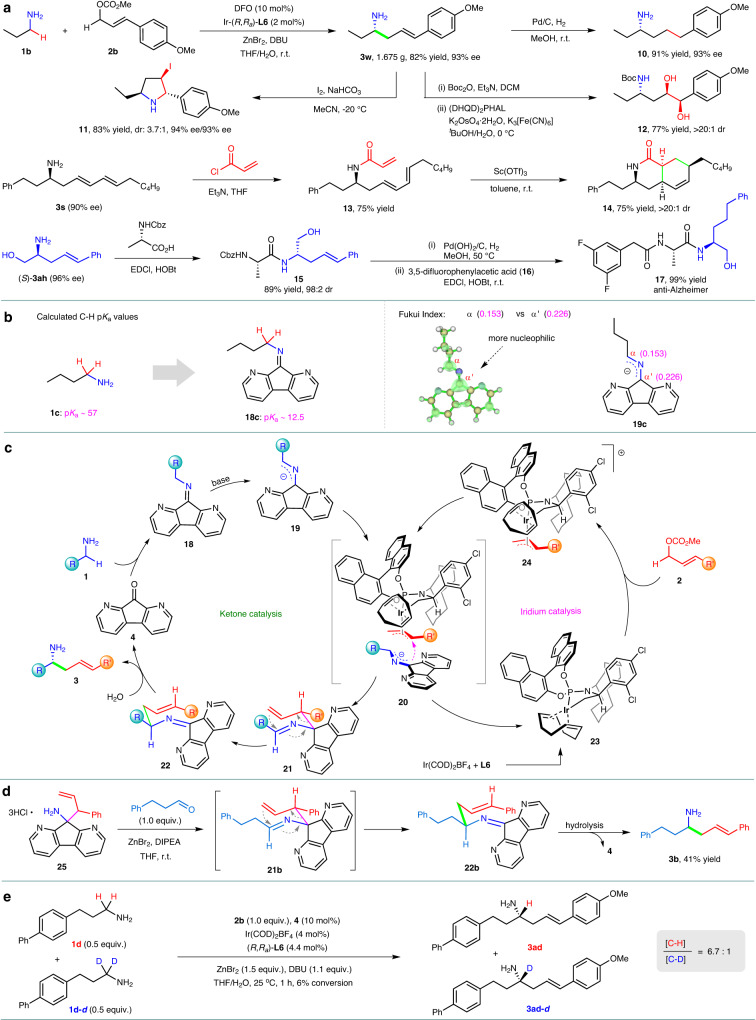
Synthetic utility and mechanistic studies. **a** Synthetic applications. **b** Computational studies. **c** Proposed mechanism. **d** Control experiment. **e** Kinetic isotope effect studies. (DHQD)_2_PHAL *bis* (dihydroquinidino)phthalazine, Cbz benzyloxycarbonyl, EDCl 1-ethyl−3-(3-dimethylaminopropyl)carbodiimide hydrochloride, HOBt 1-hydroxybenzotriazole, DIPEA diisopropylethylamine.

The reaction can be scaled up to gram scale, even with a lower catalyst loading (Fig. 4a). When 2 mol% Ir(COD)_2_BF_4_/(*R*,*R*_*a*_)-**L6** was used, 1.675 g of chiral homoallylic amine **3w** was obtained from *n*-propylamine and allylic carbonate **2b** in 82% yield with 93% ee. The products, **3**, which were obtained from the α-C−H allylic alkylation, have demonstrated their synthetic usefulness through various transformations of compound **3w**, as shown in Fig. 4a. For instance, hydrogenation of **3w** resulted in chiral aliphatic amine **10** in 91% yield. Reaction of **3w** with I_2_ produced chiral pyrrolidine **11** in 83% yield and 3.7:1 dr, without any loss of enantioselectivity^11^. X-ray analysis confirmed that the major diastereomer was (2*S*,3*R*,5*S*)-**11** (Supplementary Fig. 3). Treatment of **3w** with *tert*-butyldicarbonate (Boc_2_O), followed by asymmetric dihydroxylation, led to the formation of chiral amino diol **12** with high enantiopurity. Bioactive chiral bicyclic compounds^47^, such as **14**, can also be synthesized in 56% total yield over two steps from compound **3s** via intramolecular Diels-Alder reaction. Moreover, the asymmetric α-C−H allylic alkylation can be used for the rapid synthesis of pharmaceuticals. For example, product **3ah** underwent condensation with *N*-carbobenzyloxy-L-alanine, followed by hydrogenation of the C−C double bond and the carbobenzyloxy group, and subsequent condensation with acid **16**, producing anti-Alzheimer’s compound **17**^48^ in 88% total yield over three steps.

### Mechanistic studies

Computational studies have shown that forming imines with diazafluorenone **4** can significantly increase the acidity of the α C−H bonds of alkyl amines, by up to 10^44^ times (from p*K*_a_ 57 of **1c** to p*K*_a_ 12.5 of **18c**) (Fig. 4b). This increased acidity allows for the deprotonation of the α C−H bonds under mild conditions, producing active 2-azaallyl anions^30,49^. Furthermore, the calculated Fukui index reveals that the α‘ carbon of the delocalized carbanion **19c** is more nucleophilic than the α carbon^50,51^ (Fig. 4b). In addition, Ir-catalyzed allylic substitution reactions typically result in branched products^31–35,52^, while the current transformation displayed linear regioselectivity. These suggest that the transformation most likely began with Ir-catalyzed allylic substitution, which occurred on the α‘ carbon of the delocalized carbanion **19c**, generating a branched intermediate that then underwent aza-Cope rearrangement to form the final linear product **3**, as the pathway of the transformation reported by Niu^10,11^.

An Iridium/ketone dual catalysis pathway has been proposed (Fig. 4c). The condensation of alkyl amine **1** with diazafluorenone **4** yields imine **18**, which is deprotonated at the α C−H bond to form 2-azaallyl anion **19**^49^. The delocalized carbanion **19** then undergoes asymmetric addition at the diazafluorenyl carbon to (π-allyl)Ir(III) species **24**, generated from iridium complex **23** and allylic carbonate **2**, resulting in the formation of branched intermediate **21** and the dissociation of the iridium catalyst **23**. Subsequently, compound **21** undergoes 2-aza-Cope rearrangement followed by hydrolysis, leading to α-C−H allylic alkylation product **3** and the regeneration of ketone catalyst **4**. The proposed reaction pathway is in agreement with results from control experiments shown in Fig. 4d, where amine **25** reacted with 3-phenyl propanal to produce imine intermediate **21b**, which underwent in situ 2-aza-Cope rearrangement and subsequent hydrolysis to yield amine **3b**, the same product obtained from the current α-C−H allylic alkylation of alkyl amine **1a** with cinnamyl methyl carbonate (Fig. 4d). Kinetic isotope effect studies were performed using equimolar amounts of primary alkyl amines **1d** and **1d-*d*** (Fig. 4e). The ratio of products **3ad** and deuterated **3ad-*d*** was determined as 6.7:1, indicating that the deprotonation of imine **18** to form active 2-azaallyl carbanion **19** likely is the rate-determining step for the transformation. As shown in the proposed transition state **20** (Fig. 4c), the chiral environment of the (π-allyl)Ir(III) complex controls the approaching direction of the diazafluorenyl carbanion, determining the absolute configuration of the resulting adduct **21**. The chirality of compound **21** is then transferred to the final product in a stereospecific way via 2-aza-Cope rearrangement, leading to the formation of chiral homoallylic amine **3** in high enantioselectivity.

## Discussion

In summary, we have successfully developed a direct asymmetric α-C−H allylic alkylation of NH_2_-unprotected primary alkyl amines through synergistic iridium/ketone catalysis, producing a wide range of chiral homoallylic amines in 48–95% yields with excellent diastereo- and enantioselectivities (up to 98% ee and 99:1 dr) via asymmetric allylic substitution and in situ 2-aza-Cope rearrangement. The 1,8-diazafluoren-9-one (DFO) catalyst displays extraordinary power in activating the inert α-C−H bonds of alkyl amines, improving the acidity by up to 10^44^ times and leading to the asymmetric α-C−H allylic alkylation. The reaction features excellent stereocontrol, good efficiency and a broad substrate scope, all while avoiding the need for protecting group manipulations and utilizing a simple catalyst system, which provides a straightforward and practical method for synthesizing pharmaceutically significant chiral homoallylic amines, from readily available alkyl primary amines.

## Methods

### General procedure for catalytic asymmetric α-C(*sp*^3^)−H allylic alkylation

In a glove box, to a dry 5-mL vial equipped with a magnetic stirrer bar were added Ir(COD)_2_BF_4_ (5.9 mg, 0.012 mmol), (*R,R*_*a*_)-**L6** (8.8 mg, 0.0132 mmol), THF (0.20 mL) and primary amine **1** (0.15 mmol) (Note: Introducing a part of the primary amine **1** during the catalyst preparation is helpful for accelerating the formation of the active Ir catalyst)^53^. The vial was sealed and taken out of the glove box. The mixture was stirred at 50 ^o^C for 30 min and then cooled down to room temperature, which was used as the solution of the Ir catalyst. In the glove box, to a 10 mL Schlenk tube equipped with a magnetic stirrer bar were added 1,8-diazafluoran-9-one (**4**, DFO) (5.5 mg, 0.030 mmol), THF (0.20 mL), primary amine **1** (0.30 mmol) and a solution of ZnBr_2_ (0.101 g, 0.45 mmol) in THF (0.30 mL). The Schlenk tube was sealed and taken out of the glove box. After being stirred at room temperature for 30 min, to the mixture were added allylic carbonate **2** (0.30 mmol, dissolved in 0.40 mL dry THF), 1,8-diazabicyclo[5.4.0]undec-7-ene (DBU) (0.050 g, 0.33 mmol), the pre-prepared solution of the Ir catalyst, THF (0.35 mL) and water (0.050 mL). The reaction mixture was stirred at room temperature for 36 h. A hydroxylamine (NH_2_OH) aqueous solution (0.050 mL, 50 wt% in water) was added to quench the reaction. After stirring at room temperature for 1 h, ammonium hydroxide solution (5.0 mL, 25–28 wt% in water) was added and the resulting mixture was extracted with DCM (20 mL × 3). The combined organic layers were dried over Na_2_SO_4_, filtered, concentrated under reduced pressure and purified via column chromatography on silica gel (ethyl acetate: dichloromethane: triethylamine = 100:10:1, the silica gel column was eluted with 1% v/v solution of Et_3_N in petroleum ether before sample loading) to afford compound **3**. For some specific substrates, the reaction conditions were slightly changed, the detailed information of which can be found in the SI.

### Supplementary information

Supplementary Information

Peer Review File

Description of Additional Supplementary Files

Supplementary Data 1

## Data Availability

The authors declare that the data supporting the findings of this study are available within the article and Supplementary Information file, or from the corresponding author upon request. The X-ray crystallographic coordinates for structures reported in this study have been deposited at the Cambridge Crystallographic Data Centre (CCDC), under deposition numbers of CCDC 2235306 [(*S*)-NHBoc-**3a** in Supplementary Fig. 2] and CCDC 2235307 [(2*S*, 3*R*, 5*S*)-NTs-**11** in Supplementary Fig. 3]. These data can be obtained free of charge from The Cambridge Crystallographic Data Centre via https://www.ccdc.cam.ac.uk/structures/. Coordinates of the optimized structures are available from the Supplementary Data 1.

## References

[1] Vitaku E, Smith DT, Njardarson JT (2014). Analysis of the structural diversity, substitution patterns, and frequency of nitrogen heterocycles among U.S. FDA approved pharmaceuticals. J. Med. Chem..

[2] Nugent, T. C. *Chiral Amine Synthesis: Methods, Developments and Applications*. (Wiley‐VCH Press, 2010).

[3] Kazmaier, U. *Transition Metal Catalyzed Enantioselective Allylic Substitution in Organic Synthesis*. (Springer, 2012).

[4] Lu Z, Ma S (2008). Metal-catalyzed enantioselective allylation in asymmetric synthesis. Angew. Chem. Int. Ed..

[5] Wu X, Ren J, Shao Z, Yang X, Qian D (2021). Transition-metal-catalyzed asymmetric couplings of α-aminoalkyl fragments to access chiral alkylamines. ACS Catal..

[6] Yus M, González-Gómez JC, Foubelo F (2013). Diastereoselective allylation of carbonyl compounds and imines: application to the synthesis of natural products. Chem. Rev..

[7] Bordwell FG, Liu W-Z (1998). Effects of sulfenyl, sulfinyl and sulfonyl groups on acidities and homolytic bond dissociation energies of adjacent C—H and N—H bonds. J. Phys. Org. Chem..

[8] Campos KR (2007). Direct *sp*^3^ C–H bond activation adjacent to nitrogen in heterocycles. Chem. Soc. Rev..

[9] Mitchell EA, Peschiulli A, Lefevre N, Meerpoel L, Maes BUW (2012). Direct α-functionalization of saturated cyclic amines. Chem. Eur. J..

[10] Cao C-G, He B, Fu Z, Niu D (2019). Synthesis of β^3^-amino esters by iridium-catalyzed asymmetric allylic alkylation reaction. Org. Process. Res. Dev..

[11] Liu J, Cao C-G, Sun H-B, Zhang X, Niu D (2016). Catalytic asymmetric umpolung allylation of imines. J. Am. Chem. Soc..

[12] Zhan M, Pu X, He B, Niu D, Zhang X (2018). Intramolecular umpolung allylation of imines. Org. Lett..

[13] Mori-Quiroz LM, Londhe SS, Clift MD (2020). Formal α-allylation of primary amines by a dearomative, palladium-catalyzed umpolung allylation of N-(Aryloxy)imines. J. Org. Chem..

[14] Wang Y, Deng L-F, Zhang X, Niu D (2019). Catalytic asymmetric synthesis of α-tetrasubstituted α-trifluoromethyl homoallylic amines by Ir-catalyzed umpolung allylation of imines. Org. Lett..

[15] Shi L-M (2019). Catalytic asymmetric synthesis of α-trifluoromethyl homoallylic amines via umpolung allylation/2-Aza-cope rearrangement: stereoselectivity and mechanistic insight. Org. Lett..

[16] Yamaguchi J, Yamaguchi AD, Itami K (2012). C-H bond functionalization: emerging synthetic tools for natural products and pharmaceuticals. Angew. Chem. Int. Ed..

[17] Ye J, Kalvet I, Schoenebeck F, Rovis T (2018). Direct α-alkylation of primary aliphatic amines enabled by CO_2_ and electrostatics. Nat. Chem..

[18] Vasu D, Fuentes de Arriba AL, Leitch JA, de Gombert A, Dixon DJ (2019). Primary α-tertiary amine synthesis via α-C–H functionalization. Chem. Sci..

[19] Askey HE (2021). Photocatalytic hydroaminoalkylation of styrenes with unprotected primary alkylamines. J. Am. Chem. Soc..

[20] Chen J (2018). Carbonyl catalysis enables a biomimetic asymmetric Mannich reaction. Science.

[21] Wang Q, Gu Q, You S-L (2019). Enantioselective carbonyl catalysis enabled by chiral aldehydes. Angew. Chem. Int. Ed..

[22] Li S, Chen X-Y, Enders D (2018). Aldehyde catalysis: new options for asymmetric organocatalytic reactions. Chem.

[23] Xiao X, Zhao B (2023). Vitamin B_6_-based biomimetic asymmetric catalysis. Acc. Chem. Res..

[24] Wen W, Guo Q-X (2023). Recent advances in chiral aldehyde catalysis for asymmetric functionalization of amines. Synthesis.

[25] Li B-J, Ei-Nachef C, Beauchemin AM (2017). Organocatalysis using aldehydes: the development and improvement of catalytic hydroaminations, hydrations and hydrolyses. Chem. Commun..

[26] Cheng A (2021). Efficient asymmetric biomimetic aldol reaction of glycinates and trifluoromethyl ketones by carbonyl catalysis. Angew. Chem. Int. Ed..

[27] Ma J (2021). Enantioselective synthesis of pyroglutamic acid esters from glycinate via carbonyl catalysis. Angew. Chem. Int. Ed..

[28] Hou C (2022). Catalytic asymmetric α C(*sp*^3^)–H addition of benzylamines to aldehydes. Nat. Catal..

[29] Ji P (2022). Direct asymmetric α-C−H addition of N-unprotected propargylic amines to trifluoromethyl ketones by carbonyl catalysis. Angew. Chem. Int. Ed..

[30] Grigg R, Mongkolaussavaratana T, Anthony Pounds C, Sivagnanam S (1990). 1,8-diazafluorenone and related compounds. A new reagent for the detection of α-amino acids and latent fingerprints. Tetrahedron Lett..

[31] Cheng Q (2019). Iridium-catalyzed asymmetric allylic substitution reactions. Chem. Rev..

[32] Hartwig JF, Stanley LM (2010). Mechanistically driven development of iridium catalysts for asymmetric allylic substitution. Acc. Chem. Res..

[33] Qu J, Helmchen G (2017). Applications of iridium-catalyzed asymmetric allylic substitution reactions in target-oriented synthesis. Acc. Chem. Res..

[34] Rössler SL, Petrone DA, Carreira EM (2019). Iridium-catalyzed asymmetric synthesis of functionally rich molecules enabled by (Phosphoramidite,Olefin) ligands. Acc. Chem. Res..

[35] Stivala CE, Zbieg JR, Liu P, Krische MJ (2022). Chiral Amines via Enantioselective π-Allyliridium-*C*,*O*-Benzoate-Catalyzed Allylic Alkylation: Student Training via Industrial–Academic Collaboration. Acc. Chem. Res..

[36] Allen AE, MacMillan DWC (2012). Synergistic catalysis: a powerful synthetic strategy for new reaction development. Chem. Sci..

[37] Romiti F (2019). Different strategies for designing dual-catalytic enantioselective processes: from fully cooperative to non-cooperative systems. J. Am. Chem. Soc..

[38] Chen D-F, Gong L-Z (2022). Organo/transition-metal combined catalysis rejuvenates both in asymmetric synthesis. J. Am. Chem. Soc..

[39] Krautwald S, Sarlah D, Schafroth MA, Carreira EM (2013). Enantio- and diastereodivergent dual catalysis: α-allylation of branched aldehydes. Science.

[40] Næsborg L, Halskov KS, Tur F, Mønsted SMN, Jørgensen KA (2015). Asymmetric γ-allylation of α,β-unsaturated aldehydes by combined organocatalysis and transition-metal catalysis. Angew. Chem. Int. Ed..

[41] Jiang X, Beiger JJ, Hartwig JF (2017). Stereodivergent allylic substitutions with aryl acetic acid esters by synergistic iridium and lewis base catalysis. J. Am. Chem. Soc..

[42] Wei L, Zhu Q, Xu S-M, Chang X, Wang C-J (2018). Stereodivergent synthesis of α,α-disubstituted α-amino acids via synergistic Cu/Ir catalysis. J. Am. Chem. Soc..

[43] Jiang R, Ding L, Zheng C, You S-L (2021). Iridium-catalyzed Z-retentive asymmetric allylic substitution reactions. Science.

[44] Bhaskararao B (2022). Ir and NHC dual chiral synergetic catalysis: mechanism and stereoselectivity in γ-butyrolactone formation. J. Am. Chem. Soc..

[45] Huo X (2022). Stereodivergent Pd/Cu catalysis for asymmetric desymmetric alkylation of allylic geminal dicarboxylates. CCS Chem..

[46] Biya E, Neetha M, Anilkumar G (2021). An overview of iridium-catalyzed allylic amination reactions. ChemistrySelect.

[47] Frankowski KJ (2010). *N*-Alkyl-octahydroisoquinolin-1-one-8-carboxamides: selective and nonbasic κ-opioid receptor ligands. ACS Med. Chem. Lett..

[48] Garofalo AW (2002). A series of C-terminal amino alcohol dipeptide Aβ inhibitors. Bioorg. Med. Chem. Lett..

[49] Tang S, Zhang X, Sun J, Niu D, Chruma JJ (2018). 2-Azaallyl anions, 2-azaallyl cations, 2-azaallyl radicals, and azomethine ylides. Chem. Rev..

[50] Parr RG, Yang W (1984). Density functional approach to the frontier-electron theory of chemical reactivity. J. Am. Chem. Soc..

[51] Lu T, Chen F (2012). Multiwfn: a multifunctional wavefunction analyzer. J. Comput. Chem..

[52] Madrahimov ST, Li Q, Sharma A, Hartwig JF (2015). Origins of regioselectivity in iridium catalyzed allylic substitution. J. Am. Chem. Soc..

[53] Huo X, Zhang J, Fu J, He R, Zhang W (2018). Ir/Cu dual catalysis: enantio- and diastereodivergent access to α,α-disubstituted α-amino acids bearing vicinal stereocenters. J. Am. Chem. Soc..

