# Supplementary material for: Asymmetric α-C(sp3)−H allylic alkylation of primary alkylamines by synergistic Ir/ketone catalysis
Source: Nat Commun. 2024 Jan 31;15:939. doi: 10.1038/s41467-024-45131-3 (PMC10830461; doi:10.1038/s41467-024-45131-3)
Supplement: Supplementary file 1 — Supplementary Information [file 41467_2024_45131_MOESM1_ESM.pdf]

SUPPLEMENTARY INFORMATION

Asymmetric α-C(sp^3)–H Allylic Alkylation of Primary Alkylamines by Synergistic Ir/Ketone Catalysis

Jianyu Li[1], Sheng Gong[1], Shaolun Gao[1], Jianfeng Chen[1]*, Wen-Wen Chen[1] & Baoguo Zhao[1]*

[1] The Education Ministry Key Lab of Resource Chemistry and Shanghai Frontiers Science Center of Biomimetic Catalysis, Shanghai Normal University, Shanghai 200234, China

Email: jfchen@shnu.edu.cn; zhaobg2006@shnu.edu.cn

Table of Contents

1. Supplementary Methods 2
1.1 General information 2
1.2 Representative bioactive homoallylic amines 4
1.3 Reaction condition optimization 5
1.4 Procedure for the synthesis of pyridoxals **5** and **6** 7
1.5 General Procedure for catalytic asymmetric α-C–H allylic alkylation 10
1.6 Procedure for synthesis of **3w** in gram-scale 34
1.7 Determination of absolute configuration of compound **3a** by X-ray analysis 35
1.8 Procedure for synthesis of compound **10** 38
1.9 Procedure for synthesis of compound **11** 39
1.10 Determination of absolute configuration of compound (2*S*,3*R*,5*S*)-**11** by X-ray analysis 40
1.11 Procedure for synthesis of compound **12** 43
1.12 Procedure for synthesis of compound **14** 45
1.13 Procedure for synthesis of compound **17** 50
2. Supplementary Discussion 52
2.1 Investigation on the aza-Cope rearrangement of intermediate **21** 52
2.2 Synthesis of alkyl amine **1d-*d*** 55
2.3 Kinetic isotope effect studies 58
2.4 Investigation on the influence of $ZnBr_2$ 60
2.5 Determination of pK_a values by calculations 63
3. Supplementary Figures 65
3.1 NMR spectra 65
3.2 Chromatograms for the determination of enantiomeric and diastereomeric excesses 223
4. Supplementary References 281

1. Supplementary Methods

1.1 General Information

Chemicals

Chemicals were commercially purchased from Bidepharm, Leyan, Energy Chemical, Macklin, Aladdin, Tansoole and Adamas-beta, and directly used without further purification unless otherwise stated. Dry toluene and THF were freshly distilled from sodium-benzophenone under argon atmosphere. Dichloromethane (DCM) was freshly distilled from CaH_2. Methanol was freshly distilled from magnesium turnings. Column chromatography was performed on silica gel (200-300 mesh).

1,8-Diazafluoran-9-one (DFO) was purified by column chromatography on silica gel to give a golden yellow power. Pyridoxals **7-8** were prepared according to the reported procedure[1]. Ligand **L2** was purchased from Strem. Ligands **L1**[2], **L3**[3] and **L4-L6**[4] were prepared according to the corresponding literature procedures.

Nuclear Magnetic Resonance (NMR) Spectroscopy

^{1}H NMR spectra were recorded on Bruker AVANCE-NEO instrument (400 MHz spectrometer) at 298K. The analytical sample was dissolved in an appropriate deuterated solvent. The deuterated solvent employed and the measuring frequency are indicated in each ^{1}H NMR data. Chemical shifts are reported in parts per million (ppm) with the solvent resonance as the internal reference ($CDCl_3$ δ 7.26, CD_3OD δ 3.31, DMSO-d_6 δ 2.50, D_2O δ 4.79, CD_2Cl_2 δ 5.32). The multiplicity is described as s (singlet), d (doublet), t (triplet), q (quartet), m (multiplet), and brs (broad singlet). ^{13}C NMR spectra were recorded on Bruker AVANCE-NEO instrument (100 MHz spectrometer) at 298K. The deuterated solvent employed and the measuring frequency are both indicated in each ^{13}C NMR data. Chemical shifts are reported in ppm with the solvent resonance as the internal reference ($CDCl_3$ δ 77.16, CD_3OD δ 49.00, DMSO-d_6 δ 39.52, CD_2Cl_2 δ 54.00).

High Resolution Mass Spectrometry (HRMS)

HRMS were recorded on a liquid chromatograph/quadrupole time of flight (6545 LC/Q-TOF, Agilent) using electrospray ionization (ESI) at Shanghai Normal University. The calculated values are based on the most abundant isotope.

Optical rotations

Optical rotations were measured with a Rudolph Autopol I Automatic Polarimeter using a sodium lamp (sodium D line, λ = 589 nm) in the indicated solvent at the indicated temperature. The measurements were carried out in a 2.0 mL cell (100 mm length) with concentrations (g/100mL) reported in the corresponding solvent. The optical rotation values ($[\alpha]_D$) were reported at a given temperature (°C) in deg. mL g^{-1} dm^{-1}.

High Performance Liquid Chromatography (HPLC)

HPLC analysis was performed on a 1260 infinity II (Agilent) with a Chiralcel AD-H, AS-H or Chiralpak OD-H, OD-3, OJ-H column (Daicel Chemical Industries, Ltd.). The solvents (*n*-hexane and *iso*-propanol, HPLC-grade) used as the eluent were purchased from *J&K* scientific Ltd. The column type and the eluent (a mixture of *n*-hexane and *iso*-propanol) are indicated for each experiment.

X-ray crystallography

X-ray crystallography was performed on a Bruker D8 Venture diffractometer using GaKα radiation (λ = 1.34139 Å) at Shanghai Institute of Organic Chemistry, Chinese Academy of Science.

Melting points

Melting points were recorded on an SGW X-4B digital melting point measuring instrument and uncorrected.

1.2 Representative bioactive homoallylic amines

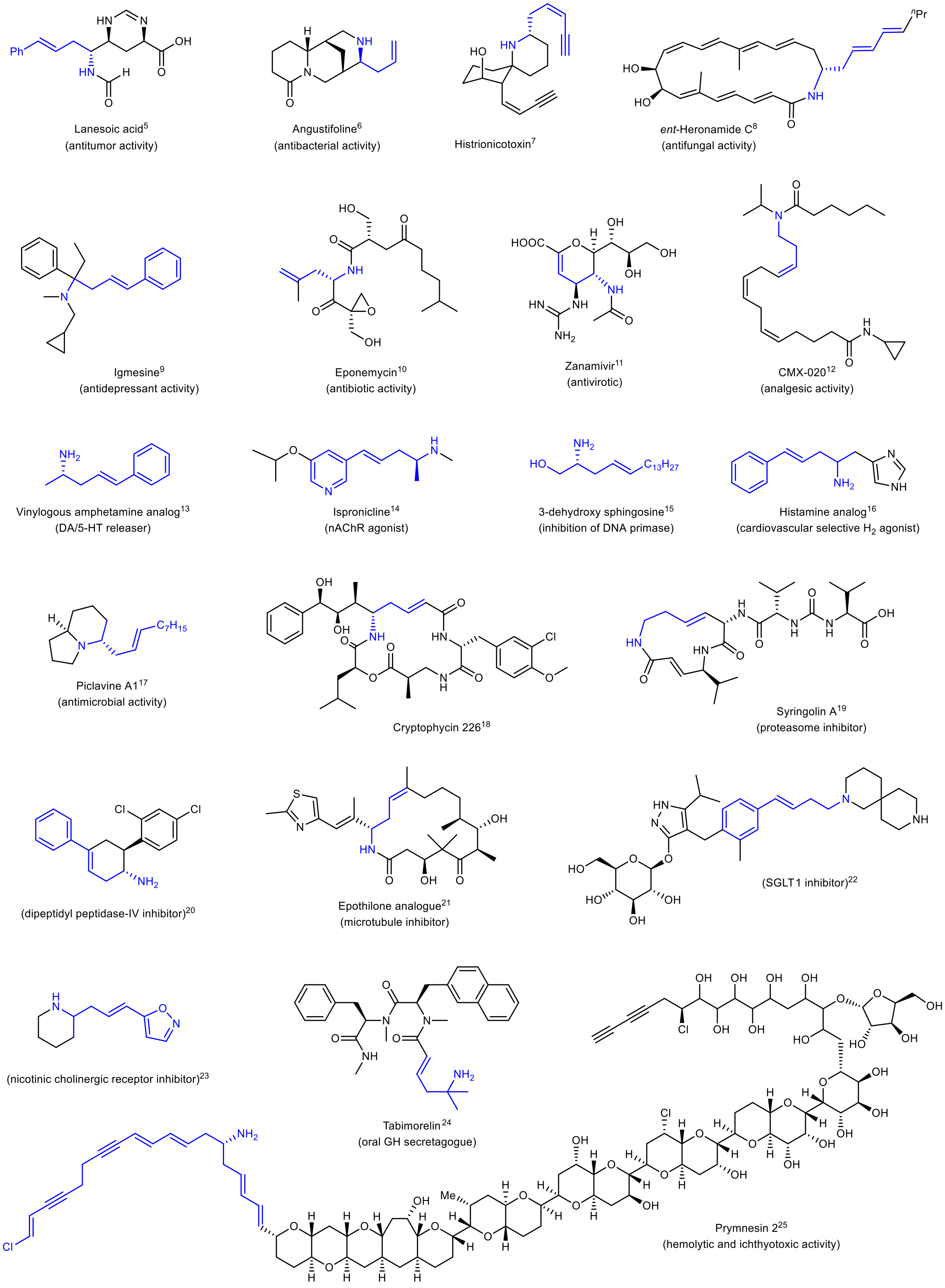

Supplementary Fig. 1. Selected bioactive homoallylic amines

1.3 Reaction condition optimization

Supplementary Table 1. Investigation on reaction conditions for the asymmetric α-C−H allylic alkylation[a]

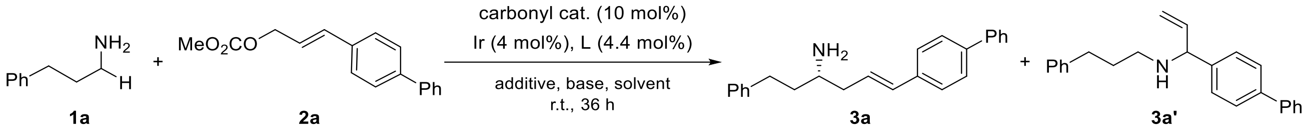

entry	carbonyl cat.	Ir	ligand	additive	base	solvent	**3a/3a′**[b]	yield[c]	ee[d]
1	none	$[Ir(COD)Cl]_2$	**L1**	$ZnBr_2$	DBU	THF/H_2O (29:1)	<1:20	19%[e] (**3a′**)	-
2	**4**	none	none	$ZnBr_2$	DBU	THF/H_2O (29:1)	-	n.r.[f]	-
3	**4**	$[Ir(COD)Cl]_2$	**L1**	none	DBU	THF/H_2O (29:1)	<1:20	87%[e] (**3a′**)	-
4	**4**	$[Ir(COD)Cl]_2$	**L1**	$ZnBr_2$	DBU	THF/H_2O (29:1)	>20:1	89%	91%
5	**4**	$[Ir(COD)Cl]_2$	**L1**	$ZnBr_2$	TBD	THF/H_2O (29:1)	>20:1	86%	90%
6	**4**	$[Ir(COD)Cl]_2$	**L1**	$ZnBr_2$	TMG	THF/H_2O (29:1)	>20:1	87%	91%
7	**4**	$[Ir(COD)Cl]_2$	**L1**	$ZnBr_2$	Et_3N	THF/H_2O (29:1)	>20:1	88%	91%
8	**4**	$[Ir(COD)Cl]_2$	**L1**	$ZnBr_2$	DABCO	THF/H_2O (29:1)	2:1	22%	85%
9	**4**	$[Ir(COD)Cl]_2$	**L1**	$ZnBr_2$	Cs_2CO_3	THF/H_2O (29:1)	>20:1	83%	89%
10	**4**	$[Ir(COD)Cl]_2$	**L1**	$ZnBr_2$	$NaHCO_3$	THF/H_2O (29:1)	>20:1	58%	71%
11	**4**	$[Ir(COD)Cl]_2$	**L1**	$ZnBr_2$	KO^tBu	THF/H_2O (29:1)	>20:1	71%	75%
12	**4**	$[Ir(COE)_2Cl]_2$	**L1**	$ZnBr_2$	DBU	THF/H_2O (29:1)	-	n.r.[f]	-
13	**4**	$Ir(COD)_2BF_4$	**L1**	$ZnBr_2$	DBU	THF/H_2O (29:1)	>20:1	87%	92%
14	**4**	Ir(acac)(COD)	**L1**	$ZnBr_2$	DBU	THF/H_2O (29:1)	8:1	37%	79%
15	**5**	$Ir(COD)_2BF_4$	**L1**	$ZnBr_2$	DBU	THF/H_2O (29:1)	<1:20	84%[e] (**3a′**)	-
16	**6**	$Ir(COD)_2BF_4$	**L1**	$ZnBr_2$	DBU	THF/H_2O (29:1)	<1:20	90%[e] (**3a′**)	-
17	**7**	$Ir(COD)_2BF_4$	**L1**	$ZnBr_2$	DBU	THF/H_2O (29:1)	<1:20	31%[e] (**3a′**)	-
18	**8**	$Ir(COD)_2BF_4$	**L1**	$ZnBr_2$	DBU	THF/H_2O (29:1)	<1:20	36%[e] (**3a′**)	-
19	**9**	$Ir(COD)_2BF_4$	**L1**	$ZnBr_2$	DBU	THF/H_2O (29:1)	<1:20	38%[e] (**3a′**)	-
20	**4**	$Ir(COD)_2BF_4$	**L2**	$ZnBr_2$	DBU	THF/H_2O (29:1)	>20:1	73%	-86%
21	**4**	$Ir(COD)_2BF_4$	**L3**	$ZnBr_2$	DBU	THF/H_2O (29:1)	>20:1	41%	-81%
22	**4**	$Ir(COD)_2BF_4$	**L4**	$ZnBr_2$	DBU	THF/H_2O (29:1)	>20:1	95%	94%
23	**4**	$Ir(COD)_2BF_4$	**L5**	$ZnBr_2$	DBU	THF/H_2O (29:1)	>20:1	95%	95%
24	**4**	$Ir(COD)_2BF_4$	**L6**	$ZnBr_2$	DBU	THF/H_2O (29:1)	>20:1	95%	97%
25	**4**	$Ir(COD)_2BF_4$	**L6**	$ZnBr_2$	DBU	CH_2Cl_2/H_2O (29:1)	-	trace[g]	-
26	**4**	$Ir(COD)_2BF_4$	**L6**	$ZnBr_2$	DBU	$CHCl_3/H_2O$ (29:1)	-	trace[g]	-

27	**4**	$Ir(COD)_2BF_4$	**L6**	$ZnBr_2$	DBU	toluene/H_2O (29:1)	-	trace[g]	-
28	**4**	$Ir(COD)_2BF_4$	**L6**	$ZnBr_2$	DBU	CH_3CN/H_2O (29:1)	-	n.r.[f]	-
29	**4**	$Ir(COD)_2BF_4$	**L6**	$ZnBr_2$	DBU	EtOH/H_2O (29:1)	-	trace[g]	-
30	**4**	$Ir(COD)_2BF_4$	**L6**	$ZnBr_2$	DBU	^{t}BuOMe/H_2O (29:1)	-	n.r.[f]	-
31	**4**	$Ir(COD)_2BF_4$	**L6**	$ZnBr_2$	DBU	DMF/H_2O (29:1)	3.5:1	39%	87%
32	**4**	$Ir(COD)_2BF_4$	**L6**	$ZnBr_2$	DBU	THF	12:1	29%	94%
33	**4**	$Ir(COD)_2BF_4$	**L6**	$ZnBr_2$	DBU	THF/H_2O (14:1)	10:1	45%	92%
34	**4**	$Ir(COD)_2BF_4$	**L6**	$ZnBr_2$	DBU	THF/H_2O (13:2)	7:1	40%	92%
35	**4**	$Ir(COD)_2BF_4$	**L6**	$ZnBr_2$	DBU	THF/H_2O (4:1)	16:1	31%	93%
36	**4**	$Ir(COD)_2BF_4$	**L6**	$ZnCl_2$	DBU	THF/H_2O (29:1)	13:1	16%	88%
37	**4**	$Ir(COD)_2BF_4$	**L6**	$Zn(OAc)_2$	DBU	THF/H_2O (29:1)	>20:1	13%	70%
38	**4**	$Ir(COD)_2BF_4$	**L6**	$Zn(OTf)_2$	DBU	THF/H_2O (29:1)	-	n.r.[f]	-
39	**4**	$Ir(COD)_2BF_4$	**L6**	$CuBr_2$	DBU	THF/H_2O (29:1)	-	n.r.[f]	-
40	**4**	$Ir(COD)_2BF_4$	**L6**	$FeBr_2$	DBU	THF/H_2O (29:1)	>20:1	4%[e] (**3a′**)	-
41	**4**	$Ir(COD)_2BF_4$	**L6**	$MgBr_2$	DBU	THF/H_2O (29:1)	<1:20	12%[e] (**3a′**)	-
42	**4**	$Ir(COD)_2BF_4$	**L6**	$Sc(OTf)_3$	DBU	THF/H_2O (29:1)	-	n.r.[f]	-

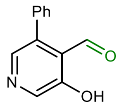

5

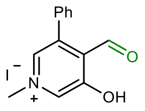

6

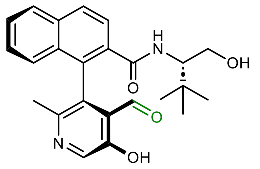

7

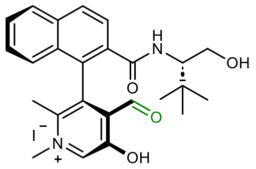

8

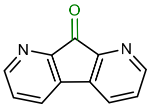

4

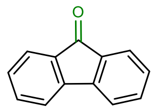

9

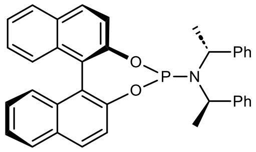

(R,R,R_a)-**L1**

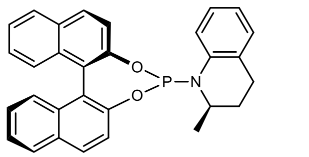

(R,R_a)-**L2**

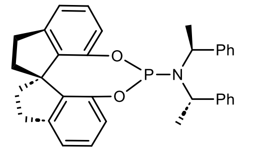

(R,S,S)-**L3**

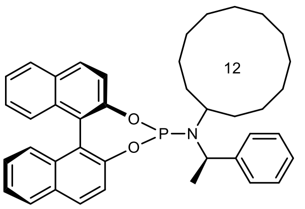

(R,R_a)-**L4**

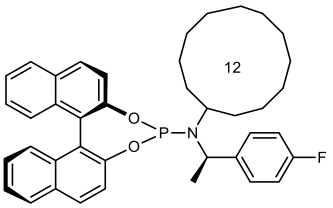

(R,R_a)-**L5**

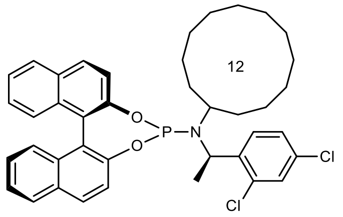

(R,R_a)-**L6**

[a]Reactions were carried out with **1a** (0.45 mmol), **2a** (0.30 mmol), **4** (DFO) (0.030 mmol), Ir (0.012 mmol), phosphoramidite ligand (0.0132 mmol), additive (0.45 mmol), and base (0.33 mmol) in solvent (1.5 mL) at room temperature (r.t.) for 36 hours. [b]The ratio of **3a/3a′** was determined by ^{1}H NMR analysis of the crude reaction mixtures. [c]Isolated yields were based on carbonate **2a**. [d]The ee values were determined by HPLC analysis. [e]NMR yield of **3a′** determined by ^{1}H NMR analysis of crude reaction mixtures. [f]Neither the α-C–H allylic alkylation nor allylic amination was observed. [g]A trace amount of product **3a** was formed.

1.4 Procedure for the Synthesis of Pyridoxals 5 and 6 (Fig. 2)

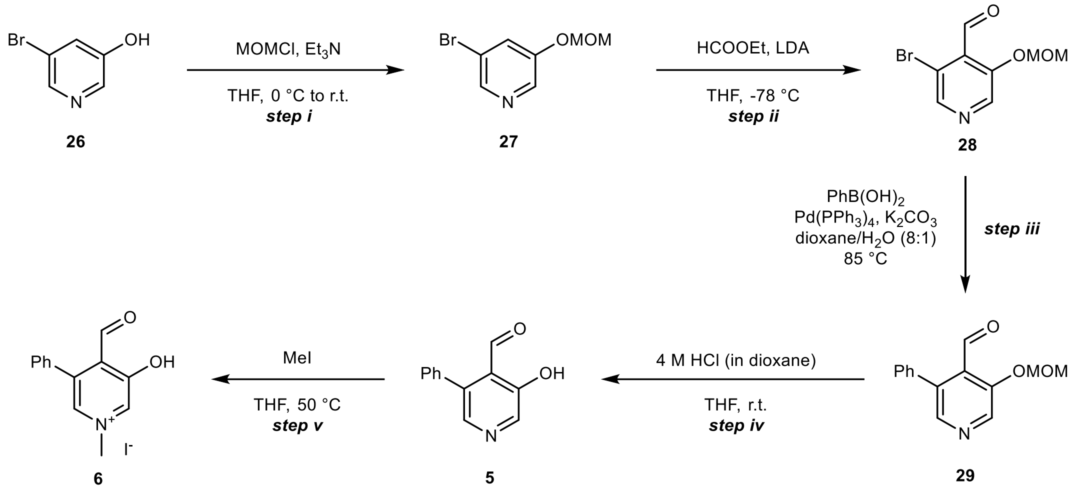

***Step i.* Synthesis of compound 27**

To a 250-mL round-bottom flask equipped with a magnetic stirrer bar was added compound **26** (10.00 g, 57.47 mmol). The sealed flask was evacuated and refilled with N_2 three times, followed by addition of dry THF (100 mL) and Et_3N (11.63 g, 114.94 mmol). The mixture was stirred at room temperature for 10 min. To the resulting solution was added chloromethyl methyl ether (MOMCl) (6.94 g, 86.20 mmol) at 0 °C dropwise over 10 min via a syringe. The mixture was allowed to warm up to room temperature and stirred for 17 hours. The mixture was diluted with water (200 mL) and extracted with ethyl acetate (100 mL× 3). The combined organic layers were dried over anhydrous Na_2SO_4, filtered, concentrated, and purified via column chromatography on silica gel (petroleum ether : ethyl acetate : DCM = 10:1:1) to afford compound **27** (11.45 g, 91% yield) as a light yellow oil.

27: Light yellow oil; ^{1}H NMR (400 MHz, $CDCl_3$) δ ^{1}H NMR (400 MHz, $CDCl_3$) δ 8.35-8.29 (m, 2H), 7.55 (t, J = 2.4 Hz, 1H), 5.18 (s, 2H), 3.48 (s, 3H); ^{13}C NMR (100 MHz, $CDCl_3$) δ 153.9, 144.2, 138.0, 125.9, 120.4, 94.9, 56.5; HRMS (ESI): *m/z* Calcd. For $C_7H_9BrNO_2$ $(M + H)^+$: 217.9811; Found: 217.9814.

***Step ii.* Synthesis of compound 28**

To a solution of diisopropylamine (6.07 g, 60.00 mmol) in anhydrous THF (60 mL) was added *n*-BuLi (24.0 mL, 2.5 M in hexane, 60.00 mmol) at -78 ºC over 15 min. After the mixture was stirred at -78 ºC for 1 hour, a solution of compound **27** (11.00 g, 50.45 mmol) in THF (40 mL) was added dropwise over 13 min at -78 ºC. Upon the reaction mixture was stirred at -78 ºC for 1.5 hours, ethyl

formate (9.26 g, 125.00 mmol) was added over 10 min. After stirring at -78 °C for 3 hours, the reaction was quenched by the addition of saturated NH_4Cl aqueous solution (100 mL). After warming to room temperature, the mixture was extracted with ethyl acetate (100 mL ×3), dried over anhydrous Na_2SO_4, filtered, concentrated, and purified via column chromatography on silica gel (petroleum ether : ethyl acetate = 10:1) to give compound **28** (8.53 g, 69% yield) as a yellow solid.

28: Yellow solid; M.p. 56-57 °C; ^{1}H NMR (400 MHz, $CDCl_3$) δ 10.37 (s, 1H), 8.59 (s, 1H), 8.51 (s, 1H), 5.31 (s, 2H), 3.52 (s, 3H); ^{13}C NMR (100 MHz, $CDCl_3$) δ 189.2, 153.5, 146.8, 137.9, 129.7, 119.2, 95.8, 57.0; HRMS (ESI): *m/z* Calcd. For $C_8H_9BrNO_3$ $(M + H)^+$: 245.9760; Found: 245.9763.

***Step iii*. Synthesis of compound 29**

To a 100-mL round-bottom flask equipped with a magnetic stirrer bar were added compound **28** (2.50 g, 10.16 mmol), $PhB(OH)_2$ (1.38 g, 11.32 mmol), $Pd(PPh_3)_4$ (0.594 g, 0.51 mmol), and K_2CO_3 (3.49 g, 25.25 mmol). The sealed flask was evacuated and refilled with N_2 three times, followed by addition of 1,4-dioxane (30 mL) and H_2O (3.8 mL) via a syringe. Upon being stirred at 85 °C for 24 hours, the reaction mixture was cooled down to room temperature and filtered. After the filter cake was washed with ethyl acetate (10 mL), ethyl acetate (30 mL) and water (60 mL) were added to the filtrate. The resulting mixture was separated and the aqueous layer was extracted with ethyl acetate (30 mL × 3). The combined organic layers were dried over Na_2SO_4, filtered, concentrated, and purified by column chromatography on silica gel (petroleum ether : ethyl acetate = 5:1) to give compound **29** (2.35 g, 95% yield) as a yellow solid.

29: Yellow solid; M.p. 44-45 °C; ^{1}H NMR (400 MHz, $CDCl_3$) δ 10.13 (s, 1H), 8.68 (s, 1H), 8.40 (s, 1H), 7.49-7.41 (m, 3H), 7.36-7.30 (m, 2H), 5.35 (s, 2H), 3.55 (s, 3H); ^{13}C NMR (100 MHz, $CDCl_3$) δ 191.0, 151.5, 145.2, 138.4, 137.9, 134.8, 129.9, 129.7, 128.8, 128.7, 95.8, 56.9; HRMS (ESI): *m/z* Calcd. For $C_{14}H_{14}NO_3$ $(M + H)^+$: 244.0968; Found: 244.0965.

***Step iv*. Synthesis of pyridoxal 5**

To a stirred solution of compound **29** (0.650 g, 2.67 mmol) in THF (5 mL) was added HCl solution (4 M in dioxane, 10.0 mL, 40.00 mmol). Upon being stirred at room temperature for 11 hours, the reaction was quenched by addition of saturated aqueous $NaHCO_3$ solution till pH 7~8 and water (20 mL) was added. The resulting mixture was extracted with ethyl acetate (20 mL × 3). The combined organic layers were dried over Na_2SO_4, filtered, concentrated and purified by column chromatography

on silica gel (petroleum ether : ethyl acetate = 1:1) to afford pyridoxal **5** (0.454 g, 85% yield) as a yellow solid.

5: Yellow solid; M.p. 83-84 °C; ^{1}H NMR (400 MHz, $CDCl_3$) δ 11.10 (s, 1H), 9.98 (s, 1H), 8.54 (s, 1H), 8.28 (s, 1H), 7.56-7.47 (m, 3H), 7.44-7.37 (m, 2H); ^{13}C NMR (100 MHz, $CDCl_3$) δ 197.7, 155.0, 141.9, 141.6, 138.3, 133.6, 130.4, 129.2, 129.0, 121.0.; HRMS (ESI): *m/z* Calcd. For $C_{12}H_{10}NO_2$ $(M + H)^+$: 200.0706; Found: 200.0704.

Step v. **Synthesis of pyridoxal 6**

To a 25-mL Schlenk tube was added pyridoxal **5** (0.250 g, 1.25 mmol). The sealed tube was evacuated and refilled with N_2 three times, followed by addition of dry THF (4 mL) and MeI (1.774 g, 12.50 mmol). Upon being stirred at 50 °C for 12 hours, the reaction mixture was filtered. The filter cake was washed by THF (5 mL × 2) and dried under reduced pressure to give pyridoxal **6** (0.381 g, 89% yield) as an orange solid.

^{1}H NMR spectroscopic analysis in D_2O showed that compound **6** exists mainly as its hydrate in the NMR.

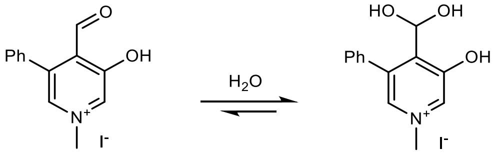

6-*aldehyde* **6**-*hydrate*

6: Orange solid; M.p. 186-187 °C; ^{1}H NMR (400 MHz, D_2O) δ 9.97 (s, 0.2H for **6**-*aldehyde*), 8.60 (s, 0.2H for **6**-*aldehyde*), 8.46 (s, 0.2H for **6**-*aldehyde*), 8.40 (s, 0.8H for **6**-*hydrate*), 8.30 (s, 0.8H for **6**-*hydrate*), 7.67-7.40 (m, 5H), 6.03 (s, 0.8H for **6**-*hydrate*), 4.39 (s, 0.6H for **6**-*aldehyde*), 4.31 (s, 2.4H for **6**-*hydrate*); ^{13}C NMR (100 MHz, D_2O) for **6**-*aldehyde*: δ 194.3, 157.6, 143.9, 136.8, 136.0, 132.8, 130.7, 130.4, 130.0, 128.3, 48.9; **6**-*hydrate*: δ 154.7, 140.4, 139.1, 137.5, 132.9, 132.5, 129.7, 129.2, 128.9, 87.4, 48.0; HRMS (ESI): *m/z* Calcd. For $C_{13}H_{12}NO_2$ $(M - I^-)$: 214.0863; Found: 214.0868.

1.5 General Procedure for Catalytic Asymmetric α-C−H Allylic Alkylation of Primary Alkyl Amines (Fig. 3)

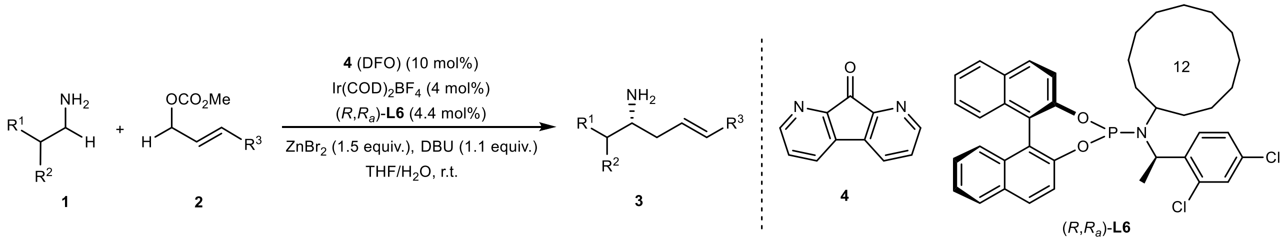

In a glove box, to a dry 5-mL vial equipped with a magnetic stirrer bar were added $Ir(COD)_2BF_4$ (5.9 mg, 0.012 mmol), (*R,R$_a$*)-**L6** (8.8 mg, 0.0132 mmol), THF (0.20 mL) and primary amine **1** (0.15 mmol) (The purpose to introduce a part of the primary amine **1** during the catalyst preparation is to accelerate the formation of the active Ir catalyst)[26]. The vial was sealed and taken out of the glove box. The mixture was stirred at 50 ºC for 30 min and then cooled down to room temperature, which was used as the solution of the Ir catalyst. In the glove box, to a 10 mL Schlenk tube equipped with a magnetic stirrer bar were added 1,8-diazafluoran-9-one (**4**, DFO) (5.5 mg, 0.030 mmol), THF (0.20 mL), primary amine **1** (0.30 mmol) and a solution of $ZnBr_2$ (0.101 g, 0.45 mmol) in THF (0.30 mL). The Schlenk tube was sealed and taken out of the glove box. After being stirred at room temperature for 30 min, to the mixture were added allylic carbonate **2** (0.30 mmol, dissolved in 0.40 mL dry THF), 1,8-diazabicyclo[5.4.0]undec-7-ene (DBU) (0.050 g, 0.33 mmol), the pre-prepared solution of the Ir catalyst, THF (0.35 mL) and water (0.050 mL). The reaction mixture was stirred at room temperature for 36 hours. A hydroxylamine (NH_2OH) aqueous solution (0.050 mL, 50 wt% in water) was added to quench the reaction. After stirring at room temperature for 1 hour, ammonium hydroxide solution (5.0 mL, 25-28 wt% in water) was added and the resulting mixture was extracted with DCM (20 mL × 3). The combined organic layers were dried over Na_2SO_4, filtered, concentrated under reduced pressure and purified via column chromatography on silica gel (ethyl acetate : DCM : Et_3N = 100:10:1, the silica gel column was eluted with 1% v/v solution of Et_3N in petroleum ether before sample loading) to afford compound **3**.

For some specific substrates, the reaction conditions were slightly changed. For compounds **3c**, **3j**, **3l-n**, **3r-t**, **3u**, **3ae**, **3ah**, **3aj**, **3am-as**, **3au**, **3aw-ay**, the reaction time was 60 hours. For compound **3aw**, ligand (*S*,*S$_a$*)-**L6** was used instead of (*R*,*R$_a$*)-**L6**. For compounds **3r-u**, **3ao**, **3au**, **3ax**, (*S*,*S$_a$*)-**L6** instead of (*R*,*R$_a$*)-**L6** and 20 mol % of DFO (11 mg, 0.06 mmol) were used. For compound **3ai**, the reaction was carried out in triple scale (0.90 mmol). For compound **3v**, **3ar**, the reaction was carried out in double scale (0.60 mmol).

For compounds **3ah** and **3ap-aq**, the reactions were carried out in double scale (0.60 mmol) with 1.8 equiv. of $ZnBr_2$ used. And no primary amine **1** was added during the preparation of the Ir catalyst. These compounds were purified by column chromatography on silica gel (DCM : MeOH : 2.9 M ammonia solution in ethanol = 100:3:5, the silica gel column was eluted with 1% v/v solution of Et_3N in petroleum ether before sample loading).

The enantiomeric excesses (ee's) of compounds **3** were determined by chiral HPLC analysis after the products were converted to the *N*-Boc derivatives (for **3a-q**, **3v-ag**, and **3aj-an**) by reaction with *tert*-butyldicarbonate (Boc_2O), *N*-benzoyl derivative (for **3r-u** and **3ao**) by reaction with benzoyl chloride, or oxazolidine-2-thiones (for **3ah-ai**) by treatment with 1,1'-thiocarbonyldiimidazole. For compounds **3ap-ba** bearing multiple chiral centers, the diastereomeric ratios (dr values) were determined by chiral HPLC analysis after the products were converted to the *N*-Boc derivatives (for **3as-at**, **3av-aw**, and **3ay-ba**) by reaction with *tert*-butyldicarbonate (Boc_2O), oxazolidine-2-thiones (for **3ar**) by treatment with 1,1'-thiocarbonyldiimidazole, *N*-benzoyl derivatives (for **3au** and **3ax**) by treatment with benzoyl chloride, or *N,O,O*-cyclohexanecarbonyl derivatives (for **3ap-aq**) by reaction with cyclohexanecarbonyl chloride.

Compound 3a (Fig. 3)

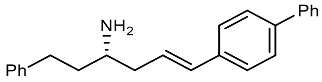

3a

The product was obtained in 95% yield (92.8 mg, 0.284 mmol, 97% ee) as a pale yellow solid from the corresponding primary amine **1** (0.45 mmol) and allylic carbonate **2** (0.30 mmol); M.p. 109-111 °C; $[\alpha]_D^{25}$ = +12.6 (c = 0.10, MeOH); ^{1}H NMR (400 MHz, $CDCl_3$) δ 7.61 (d, *J* = 7.6 Hz, 2H), 7.56 (d, *J* = 8.0 Hz, 2H), 7.48-7.40 (m, 4H), 7.38-7.28 (m, 3H), 7.26-7.17 (m, 3H), 6.51 (d, *J* = 15.6 Hz, 1H), 6.32-6.20 (m, 1H), 3.00-2.89 (m, 1H), 2.87-2.75 (m, 1H), 2.75-2.64 (m, 1H), 2.51-2.40 (m, 1H), 2.30-2.18 (m, 1H), 1.90-1.78 (m, 1H), 1.75-1.62 (m, 1H), 1.47 (brs, 2H); ^{13}C NMR (100 MHz, $CDCl_3$) δ 142.3, 140.9, 140.0, 136.5, 132.3, 128.9, 128.55, 128.51, 127.6, 127.4, 127.0, 126.6, 126.0, 50.8, 42.1, 39.6, 32.8; HRMS (ESI): *m*/*z* Calcd. for $C_{24}H_{26}N$ $(M + H)^+$: 328.2060; Found: 328.2063.

Compound 3b (Fig. 3)

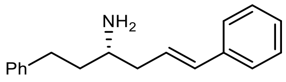

3b

The product was obtained in 92% yield (69.1 mg, 0.275 mmol, 94% ee) as a yellow oil from the corresponding primary amine **1** (0.45 mmol) and allylic carbonate **2** (0.30 mmol); $[\alpha]_D^{25}$ = +12.5 (c = 0.10, MeOH); ^{1}H NMR (400 MHz, $CDCl_3$) δ 7.39-7.34 (m, 2H), 7.34-7.27 (m, 4H), 7.25-7.16 (m, 4H), 6.47 (d, *J* = 15.6 Hz, 1H), 6.27-6.15 (m, 1H), 2.97-2.87 (m, 1H), 2.85-2.74 (m, 1H), 2.73-2.63 (m, 1H), 2.48-2.37 (m, 1H), 2.27-2.15 (m, 1H), 1.89-1.76 (m, 1H), 1.73-1.60 (m, 1H), 1.31 (brs, 2H); ^{13}C NMR (100 MHz, $CDCl_3$) δ 142.2, 137.4, 132.8, 128.6, 128.50, 128.46, 127.3, 127.2, 126.1, 125.9, 50.7, 41.7, 39.3, 32.7; HRMS (ESI): *m*/*z* Calcd. For $C_{18}H_{22}N$ $(M + H)^+$: 252.1747; Found: 252.1749.

Compound 3c (Fig. 3)

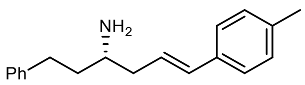

3c

The product was obtained in 79% yield (62.9 mg, 0.237 mmol, 94% ee) as a yellow solid from the corresponding primary amine **1** (0.45 mmol) and allylic carbonate **2** (0.30 mmol); M.p. 43-45 °C; $[\alpha]_D^{25}$ = +15.9 (c = 0.10, MeOH); ^{1}H NMR (400 MHz, $CDCl_3$) δ 7.33-7.24 (m, 4H), 7.24-7.16 (m, 3H), 7.12 (d, *J* = 7.6 Hz, 2H), 6.43 (d, *J* = 16.0 Hz, 1H), 6.20-6.09 (m, 1H), 2.95-2.86 (m, 1H), 2.84-2.74 (m, 1H), 2.73-2.62 (m, 1H), 2.46-2.37 (m, 1H), 2.34 (s, 3H), 2.25-2.13 (m, 1H), 1.88-1.75 (m, 1H), 1.72-1.60 (m, 1H), 1.43 (brs, 2H); ^{13}C NMR (100 MHz, $CDCl_3$) δ 142.3, 137.0, 134.7, 132.6, 129.3, 128.52, 128.50, 126.3, 126.1, 125.9, 50.8, 42.0, 39.5, 32.8, 21.3; HRMS (ESI): *m*/*z* Calcd. For $C_{19}H_{24}N$ $(M + H)^+$: 266.1903; Found: 266.1907.

Compound 3d (Fig. 3)

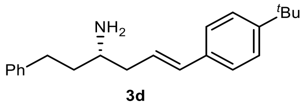

3d

The product was obtained in 75% yield (69.2 mg, 0.225 mmol, 93% ee) as a brown solid from the corresponding primary amine **1** (0.45 mmol) and allylic carbonate **2** (0.30 mmol); M.p. 34-35 °C; $[\alpha]_D^{25}$ = +13.0 (c = 0.10, MeOH); ^{1}H NMR (400 MHz, $CDCl_3$) δ 7.36-7.27 (m, 6H), 7.24-7.16 (m, 3H), 6.45 (d, *J* = 15.6 Hz, 1H), 6.21-6.11 (m, 1H), 2.95-2.86 (m, 1H), 2.84-2.74 (m, 1H), 2.73-2.62 (m, 1H),

2.47-2.37 (m, 1H), 2.25-2.14 (m, 1H), 1.87-1.76 (m, 1H), 1.72-1.60 (m, 1H), 1.52 (brs, 2H), 1.32 (s, 9H); ^{13}C NMR (100 MHz, $CDCl_3$) δ 150.4, 142.3, 134.7, 132.6, 128.53, 128.51, 126.5, 125.93, 125.89, 125.6, 50.8, 42.0, 39.5, 34.7, 32.8, 31.4; HRMS (ESI): *m/z* Calcd. For $C_{22}H_{30}N$ $(M + H)^+$: 308.2373; Found: 308.2374.

Compound 3e (Fig. 3)

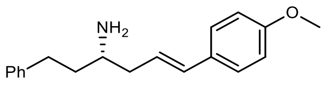

3e

The product was obtained in 82% yield (69.0 mg, 0.246 mmol, 94% ee) as a brown solid from the corresponding primary amine **1** (0.45 mmol) and allylic carbonate **2** (0.30 mmol); M.p. 53-54 °C; $[\alpha]_D^{25}$ = +16.3 (c = 0.10, MeOH); ^{1}H NMR (400 MHz, $CDCl_3$) δ 7.34-7.27 (m, 4H), 7.25-7.16 (m, 3H), 6.86 (d, *J* = 8.0 Hz, 2H), 6.41 (d, *J* = 16.0 Hz, 1H), 6.12-6.00 (m, 1H), 3.81 (s, 3H), 2.95-2.85 (m, 1H), 2.84-2.74 (m, 1H), 2.73-2.62 (m, 1H), 2.46-2.35 (m, 1H), 2.25-2.13 (m, 1H), 1.88-1.76 (m, 1H), 1.73-1.60 (m, 1H), 1.48 (brs, 2H); ^{13}C NMR (100 MHz, $CDCl_3$) δ 159.0, 142.3, 132.1, 130.3, 128.48, 128.47, 127.3, 125.9, 125.0, 114.0, 55.4, 50.8, 41.9, 39.5, 32.8; HRMS (ESI): *m/z* Calcd. For $C_{19}H_{24}NO$ $(M + H)^+$: 282.1852; Found: 282.1855.

Compound 3f (Fig. 3)

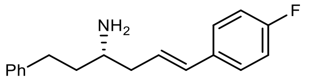

3f

The product was obtained in 83% yield (66.7 mg, 0.248 mmol, 95% ee) as a yellow solid from the corresponding primary amine **1** (0.45 mmol) and allylic carbonate **2** (0.30 mmol); M.p. 42-43 °C; $[\alpha]_D^{25}$ = +9.8 (c = 0.10, MeOH); ^{1}H NMR (400 MHz, $CDCl_3$) δ 7.35-7.27 (m, 4H), 7.24-7.16 (m, 3H), 6.99 (t, *J* = 8.4 Hz, 2H), 6.42 (d, *J* = 15.6 Hz, 1H), 6.16-6.06 (m, 1H), 2.96-2.86 (m, 1H), 2.84-2.74 (m, 1H), 2.72-2.62 (m, 1H), 2.47-2.35 (m, 1H), 2.25-2.13 (m, 1H), 1.88-1.75 (m, 1H), 1.73-1.60 (m, 1H), 1.53 (brs, 2H); ^{13}C NMR (100 MHz, $CDCl_3$) δ 162.2 (d, J_{C-F} = 245 Hz), 142.2, 133.6 (d, J_{C-F} = 3.3 Hz), 131.6, 128.55, 128.49, 127.6 (d, J_{C-F} = 7.6 Hz), 127.1 (d, J_{C-F} = 2.2 Hz), 126.0, 115.5 (d, J_{C-F} = 21.7 Hz), 50.7, 41.9, 39.5, 32.8; ^{19}F NMR (376 MHz, $CDCl_3$) δ -115.2; HRMS (ESI): *m/z* Calcd. For $C_{18}H_{21}FN$ $(M + H)^+$: 270.1653; Found: 270.1657.

Compound 3g (Fig. 3)

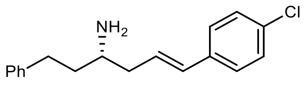

3g

The product was obtained in 80% yield (68.8 mg, 0.241 mmol, 94% ee) as a yellow solid from the corresponding primary amine **1** (0.45 mmol) and allylic carbonate **2** (0.30 mmol); M.p. 45-46 °C; $[\alpha]_D^{25}$ = +12.9 (c = 0.10, MeOH); ^{1}H NMR (400 MHz, $CDCl_3$) δ 7.32-7.23 (m, 6H), 7.22-7.16 (m, 3H), 6.40 (d, *J* = 16.0 Hz, 1H), 6.23-6.12 (m, 1H), 2.96-2.85 (m, 1H), 2.84-2.73 (m, 1H), 2.72-2.61 (m, 1H), 2.46-2.36 (m, 1H), 2.26-2.13 (m, 1H), 1.87-1.74 (m, 1H), 1.72-1.60 (m, 1H), 1.44 (brs, 2H); ^{13}C NMR (100 MHz, $CDCl_3$) δ 142.2, 136.0, 132.8, 131.5, 128.8, 128.55, 128.49, 128.1, 127.4, 126.0, 50.7, 41.9, 39.6, 32.8; HRMS (ESI): *m/z* Calcd. For $C_{18}H_{21}ClN$ $(M+H)^+$: 286.1357; Found: 286.1360.

Compound 3h (Fig. 3)

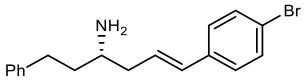

3h

The product was obtained in 77% yield (76.4 mg, 0.232 mmol, 96% ee) as a yellow solid from the corresponding primary amine **1** (0.45 mmol) and allylic carbonate **2** (0.30 mmol); M.p. 49-51 °C; $[\alpha]_D^{25}$ = +9.3 (c = 0.10, MeOH); ^{1}H NMR (400 MHz, $CDCl_3$) δ 7.42 (d, *J* = 7.6 Hz, 2H), 7.30 (t, *J* = 7.2 Hz, 2H), 7.24-7.14 (m, 5H), 6.39 (d, *J* = 15.6 Hz, 1H), 6.26-6.15 (m, 1H), 2.97-2.87 (m, 1H), 2.85-2.73 (m, 1H), 2.72-2.61 (m, 1H), 2.46-2.36 (m, 1H), 2.26-2.13 (m, 1H), 1.88-1.75 (m, 1H), 1.73-1.60 (m, 1H), 1.33 (brs, 2H); ^{13}C NMR (100 MHz, $CDCl_3$) δ 142.2, 136.4, 131.7, 131.5, 128.53, 128.47, 128.3, 127.7, 126.0, 120.9, 50.7, 42.0, 39.6, 32.8; HRMS (ESI): *m*/*z* Calcd. For $C_{18}H_{21}BrN$ $(M + H)^+$: 330.0852; Found: 330.0855.

Compound 3i (Fig. 3)

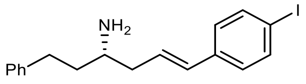

3i

The product was obtained in 76% yield (85.4 mg, 0.227 mmol, 96% ee) as a pale yellow solid from the corresponding primary amine **1** (0.45 mmol) and allylic carbonate **2** (0.30 mmol); M.p. 52-54 °C; $[\alpha]_D^{25}$ = +9.5 (c = 0.10, MeOH); ^{1}H NMR (400 MHz, $CDCl_3$) δ 7.61 (d, *J* = 7.6 Hz, 2H), 7.30 (t, *J* = 7.2 Hz, 2H), 7.24-7.16 (m, 3H), 7.09 (d, *J* = 7.6 Hz, 2H), 6.37 (d, *J* = 15.6 Hz, 1H), 6.26-6.15 (m, 1H),

2.96-2.86 (m, 1H), 2.84-2.73 (m, 1H), 2.72-2.61 (m, 1H), 2.46-2.35 (m, 1H), 2.25-2.13 (m, 1H), 1.88-1.75 (m, 1H), 1.72-1.60 (m, 1H), 1.48 (brs, 2H); ^{13}C NMR (100 MHz, $CDCl_3$) δ 142.1, 137.6, 136.9, 131.6, 128.5, 128.45, 128.40, 127.9, 125.9, 92.3, 50.6, 41.9, 39.5, 32.7; HRMS (ESI): *m*/*z* Calcd. For $C_{18}H_{21}IN$ $(M + H)^+$: 378.0713; Found: 378.0717.

Compound 3j (Fig. 3)

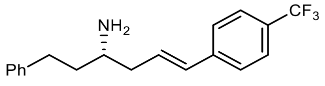

3j

The product was obtained in 59% yield (56.9 mg, 0.178 mmol, 95% ee) as a brown solid from the corresponding primary amine **1** (0.45 mmol) and allylic carbonate **2** (0.30 mmol); M.p. 63-64 °C; $[\alpha]_D^{25}$ = +9.3 (c = 0.10, MeOH); ^{1}H NMR (400 MHz, $CDCl_3$) δ 7.55 (d, *J* = 8.4 Hz, 2H), 7.43 (d, *J* = 8.4 Hz, 2H), 7.32-7.27 (m, 2H), 7.24-7.16 (m, 3H), 6.49 (d, *J* = 16.0 Hz, 1H), 6.37-6.27 (m, 1H), 2.99-2.89 (m, 1H), 2.85-2.74 (m, 1H), 2.73-2.62 (m, 1H), 2.49-2.40 (m, 1H), 2.30-2.19 (m, 1H), 1.88-1.77 (m, 1H), 1.73-1.62 (m, 1H), 1.44 (brs, 2H); ^{13}C NMR (100 MHz, $CDCl_3$) δ 142.1, 140.9 (q, *J* = 1.4 Hz), 131.5, 130.4, 129.1 (q, *J* = 32.1 Hz), 128.6, 128.5, 126.3, 126.0, 125.6 (q, *J* = 3.8 Hz), 124.4 (q, *J* = 270.2 Hz), 50.7, 41.9, 39.6, 32.8; ^{19}F NMR (376 MHz, $CDCl_3$) δ -62.4; HRMS (ESI): *m*/*z* Calcd. For $C_{19}H_{21}F_3N$ $(M + H)^+$: 320.1621; Found: 320.1624.

Compound 3k (Fig. 3)

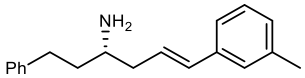

3k

The product was obtained in 89% yield (71.1 mg, 0.268 mmol, 94% ee) as a yellow oil from the corresponding primary amine **1** (0.45 mmol) and allylic carbonate **2** (0.30 mmol); $[\alpha]_D^{25}$ = +14.0 (c = 0.20, MeOH); ^{1}H NMR (400 MHz, $CDCl_3$) δ 7.30 (t, *J* = 7.6 Hz, 2H), 7.24-7.13 (m, 6H), 7.04 (d, *J* = 7.6 Hz, 1H), 6.44 (d, *J* = 16.0 Hz, 1H), 6.25-6.13 (m, 1H), 2.96-2.87 (m, 1H), 2.85-2.74 (m, 1H), 2.73-2.62 (m, 1H), 2.47-2.38 (m, 1H), 2.35 (s, 3H), 2.25-2.15 (m, 1H), 1.88-1.76 (m, 1H), 1.74-1.60 (m, 3H); ^{13}C NMR (100 MHz, $CDCl_3$) δ 142.3, 138.2, 137.4, 132.9, 128.6, 128.53, 128.50, 128.1, 127.1, 126.9, 125.9, 123.3, 50.7, 41.9, 39.5, 32.8, 21.5; HRMS (ESI): *m*/*z* Calcd. For $C_{19}H_{24}N$ $(M + H)^+$: 266.1903; Found: 266.1908.

Compound 3l (Fig. 3)

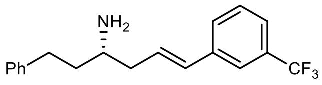

3l

The product was obtained in 48% yield (46.4 mg, 0.145 mmol, 95% ee) as a yellow oil from the corresponding primary amine **1** (0.45 mmol) and allylic carbonate **2** (0.30 mmol); $[\alpha]_D^{25}$ = +13.4 (c = 0.10, MeOH); ^{1}H NMR (400 MHz, $CDCl_3$) δ 7.59 (s, 1H), 7.50 (d, *J* = 7.6 Hz, 1H), 7.46 (d, *J* = 7.2 Hz, 1H), 7.40 (t, *J* = 7.6 Hz, 1H), 7.29 (t, *J* = 7.2 Hz, 2H), 7.24-7.16 (m, 3H), 6.48 (d, *J* = 15.6 Hz, 1H), 6.34-6.23 (m, 1H), 2.99-2.89 (m, 1H), 2.85-2.74 (m, 1H), 2.73-2.62 (m, 1H), 2.50-2.39 (m, 1H), 2.29-2.17 (m, 1H), 1.89-1.76 (m, 1H), 1.74-1.65 (m, 1H), 1.62 (brs, 2H); ^{13}C NMR (100 MHz, $CDCl_3$) δ 142.1, 138.2, 131.5, 131.0 (q, *J* = 31.8 Hz), 129.5, 129.4, 129.1, 128.6, 128.5, 126.0, 124.3 (q, *J* = 270.8 Hz),123.8 (q, *J* = 3.6 Hz), 122.8 (q, *J* = 3.6 Hz), 50.6, 41.9, 39.5, 32.8; ^{19}F NMR (376 MHz, $CDCl_3$) δ -62.7; HRMS (ESI): *m/z* Calcd. For $C_{19}H_{21}F_3N$ $(M + H)^+$: 320.1621; Found: 320.1622.

Compound 3m (Fig. 3)

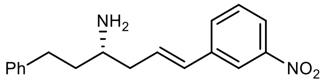

3m

The product was obtained in 54% yield (48.0 mg, 0.162 mmol, 94% ee) as a yellow oil from the corresponding primary amine **1** (0.45 mmol) and allylic carbonate **2** (0.30 mmol); $[\alpha]_D^{25}$ = +9.3 (c = 0.10, MeOH); ^{1}H NMR (400 MHz, $CDCl_3$) δ 8.20 (t, *J* = 2.0 Hz, 1H), 8.05 (dd, *J* = 8.0, 1.2 Hz, 1H), 7.63 (d, *J* = 7.6 Hz, 1H), 7.45 (t, *J* = 8.0 Hz, 1H), 7.29 (t, *J* = 7.6 Hz, 2H), 7.23-7.15 (m, 3H), 6.50 (d, *J* = 15.6 Hz, 1H), 6.42-6.32 (m, 1H), 3.00-2.90 (m, 1H), 2.85-2.74 (m, 1H), 2.73-2.62 (m, 1H), 2.51-2.40 (m, 1H), 2.30-2.20 (m, 1H), 1.89-1.77 (m, 1H), 1.74-1.62 (m, 1H), 1.39 (brs, 2H); ^{13}C NMR (100 MHz, $CDCl_3$) δ 148.7, 142.0, 139.2, 132.1, 131.0, 130.5, 129.5, 128.6, 128.5, 126.0, 121.8, 120.7, 50.6, 41.9, 39.6, 32.7; HRMS (ESI): *m/z* Calcd. For $C_{18}H_{21}N_2O_2$ $(M + H)^+$: 297.1598; Found: 297.1602.

Compound 3n (Fig. 3)

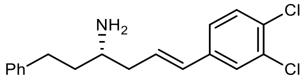

3n

The product was obtained in 71% yield (67.7 mg, 0.212 mmol, 96% ee) as a yellow solid from the corresponding primary amine **1** (0.45 mmol) and allylic carbonate **2** (0.30 mmol); M.p. 57-58 °C; $[\alpha]_D^{25}$

= +9.0 (c = 0.10, MeOH); ^{1}H NMR (400 MHz, $CDCl_3$) δ 7.42 (d, *J* = 2.0 Hz, 1H), 7.35 (d, *J* = 8.4 Hz, 1H), 7.32-7.27 (m, 2H), 7.24-7.17 (m, 3H), 7.15 (dd, *J* = 8.4, 2.0 Hz, 1H), 6.35 (d, *J* = 16.0 Hz, 1H), 6.27-6.15 (m, 1H), 2.96-2.87 (m, 1H), 2.84-2.73 (m, 1H), 2.73-2.62 (m, 1H), 2.47-2.36 (m, 1H), 2.26-2.15 (m, 1H), 1.87-1.75 (m, 1H), 1.72-1.59 (m, 1H), 1.33 (brs, 2H); ^{13}C NMR (100 MHz, $CDCl_3$) δ 142.1, 137.6, 132.7, 130.8, 130.5, 130.4, 129.7, 128.6, 128.5, 127.8, 126.0, 125.4, 50.6, 41.9, 39.6, 32.8; HRMS (ESI): *m*/*z* Calcd. For $C_{18}H_{20}Cl_2N$ $(M + H)^+$: 320.0967; Found: 320.0969.

Compound 3o (Fig. 3)

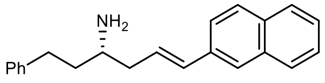

3o

The product was obtained in 83% yield (75.3 mg, 0.250 mmol, 96% ee) as a white solid from the corresponding primary amine **1** (0.45 mmol) and allylic carbonate **2** (0.30 mmol); M.p. 75-77 °C; $[\alpha]_D^{25}$ = +19.9 (c = 0.10, MeOH); ^{1}H NMR (400 MHz, $CDCl_3$) δ 7.84-7.74 (m, 3H), 7.70 (s, 1H), 7.59 (dd, *J* = 8.4, 1.6 Hz, 1H), 7.50-7.39 (m, 2H), 7.31 (t, *J* = 7.2 Hz, 2H), 7.25-7.18 (m, 3H), 6.63 (d, *J* = 16.0 Hz, 1H), 6.41-6.28 (m, 1H), 3.02-2.91 (m, 1H), 2.88-2.76 (m, 1H), 2.76-2.65 (m, 1H), 2.54-2.43 (m, 1H), 2.34-2.21 (m, 1H), 1.93-1.79 (m, 1H), 1.77-1.63 (m, 1H), 1.37 (brs, 2H); ^{13}C NMR (100 MHz, $CDCl_3$) δ 142.3, 134.9, 133.8, 132.9, 128.55, 128.52, 128.2, 128.0, 127.9, 127.8, 126.3, 126.0, 125.79, 125.77, 123.6, 50.8, 42.1, 39.6, 32.8; HRMS (ESI): *m*/*z* Calcd. For $C_{22}H_{24}N$ $(M + H)^+$: 302.1903; Found: 302.1906.

Compound 3p (Fig. 3)

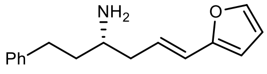

3p

The product was obtained in 87% yield (62.6 mg, 0.260 mmol, 94% ee) as an orange oil from the corresponding primary amine **1** (0.45 mmol) and allylic carbonate **2** (0.30 mmol); $[\alpha]_D^{25}$ = +14.1 (c = 0.10, MeOH); ^{1}H NMR (400 MHz, $CDCl_3$) δ 7.34-7.23 (m, 3H), 7.23-7.15 (m, 3H), 6.38-6.32 (m, 1H), 6.27 (d, *J* = 15.6 Hz, 1H), 6.18-6.04 (m, 2H), 2.94-2.84 (m, 1H), 2.83-2.72 (m, 1H), 2.71-2.60 (m, 1H), 2.44-2.34 (m, 1H), 2.22-2.10 (m, 1H), 1.87-1.73 (m, 1H), 1.71-1.58 (m, 1H), 1.52 (brs, 2H); ^{13}C NMR (100 MHz, $CDCl_3$) δ 152.9, 142.2, 141.6, 128.51, 128.49, 126.2, 125.9, 121.3, 111.3, 106.8, 50.7, 41.7, 39.5, 32.8; HRMS (ESI): *m*/*z* Calcd. For $C_{16}H_{20}NO$ $(M + H)^+$: 242.1539; Found: 242.1540.

Compound 3q (Fig. 3)

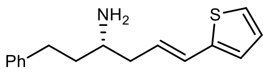

3q

The product was obtained in 77% yield (59.1 mg, 0.230 mmol, 94% ee) as a yellow oil from the corresponding primary amine **1** (0.45 mmol) and allylic carbonate **2** (0.30 mmol); $[\alpha]_D^{25}$ = +18.3 (c = 0.11, MeOH); ^{1}H NMR (400 MHz, $CDCl_3$) δ 7.29 (t, *J* = 7.2 Hz, 2H), 7.24-7.16 (m, 3H), 7.11 (d, *J* = 4.8 Hz, 1H), 6.97-6.92 (m, 1H), 6.91-6.87 (m, 1H), 6.59 (d, *J* = 15.6 Hz, 1H), 6.08-5.98 (m, 1H), 2.95-2.85 (m, 1H), 2.84-2.73 (m, 1H), 2.72-2.61 (m, 1H), 2.44-2.34 (m, 1H), 2.22-2.10 (m, 1H), 1.88-1.74 (m, 1H), 1.72-1.59 (m, 1H), 1.44 (brs, 2H); ^{13}C NMR (100 MHz, $CDCl_3$) δ 142.7, 142.2, 128.54, 128.50, 127.4, 127.3, 126.0, 125.9, 124.9, 123.6, 50.7, 41.8, 39.5, 32.8; HRMS (ESI): *m/z* Calcd. For $C_{16}H_{20}NS$ $(M + H)^+$: 258.1311; Found: 258.1314.

Compound 3r (Fig. 3)

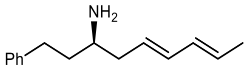

3r

The product was obtained in 54% yield (35.1 mg, 0.163 mmol, 89% ee) as a pale yellow oil from the corresponding primary amine **1** (0.45 mmol) and allylic carbonate **2** (0.30 mmol); $[\alpha]_D^{25}$ = -11.4 (c = 0.10, MeOH); ^{1}H NMR (400 MHz, $CDCl_3$) δ 7.28 (t, *J* = 8.0 Hz, 2H), 7.22-7.15 (m, 3H), 6.12-5.98 (m, 2H), 5.67-5.57 (m, 1H), 5.57-5.46 (m, 1H), 2.84-2.70 (m, 2H), 2.69-2.58 (m, 1H), 2.31-2.21 (m, 1H), 2.09-1.97 (m, 1H), 1.81-1.70 (m, 4H), 1.66-1.55 (m, 1H), 1.42 (brs, 2H); ^{13}C NMR (100 MHz, $CDCl_3$) δ 142.4, 133.4, 131.5, 128.5, 128.1, 128.0, 125.9, 50.8, 41.5, 39.5, 32.8, 18.2; HRMS (ESI): *m/z* Calcd. For $C_{15}H_{22}N$ $(M + H)^+$: 216.1747; Found: 216.1751.

Compound 3s (Fig. 3)

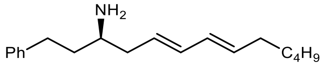

3s

The product was obtained in 72% yield (58.9 mg, 0.217 mmol, 90% ee) as a brown oil from the corresponding primary amine **1** (0.45 mmol) and allylic carbonate **2** (0.30 mmol); $[\alpha]_D^{25}$ = -11.3 (*c* = 0.10, MeOH); ^{1}H NMR (400 MHz, $CDCl_3$) δ 7.28 (t, *J* = 8.0 Hz, 2H), 7.23-7.15 (m, 3H), 6.13-5.95

(m, 2H), 5.66-5.56 (m, 1H), 5.56-5.47 (m, 1H), 2.86-2.78 (m, 1H), 2.78-2.71 (m, 1H), 2.69-2.58 (m, 1H), 2.31-2.22 (m, 1H), 2.10-1.99 (m, 3H), 1.81-1.70 (m, 1H), 1.66-1.57 (m, 1H), 1.54 (brs, 2H), 1.42-1.22 (m, 6H), 0.88 (t, *J* = 6.8 Hz, 3H); ^{13}C NMR (100 MHz, $CDCl_3$) δ 142.3, 133.7, 133.5, 130.0, 128.5, 128.2, 125.9, 50.8, 41.5, 39.4, 32.8, 32.7, 31.5, 29.1, 22.6, 14.2; HRMS (ESI): *m/z* Calcd. For $C_{19}H_{30}N$ $(M + H)^+$: 272.2373; Found: 272.2378.

Compound 3t (Fig. 3)

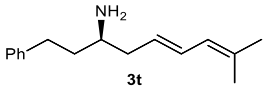

3t

The product was obtained in 82% yield (56.2 mg, 0.245 mmol, 80% ee) as an orange oil from the corresponding primary amine **1** (0.45 mmol) and allylic carbonate **2** (0.30 mmol); $[\alpha]_D^{25}$ = -11.3 (*c* = 0.10, MeOH); ^{1}H NMR (400 MHz, $CDCl_3$) δ 7.28 (t, *J* = 7.6 Hz, 2H), 7.22-7.15 (m, 3H), 6.30 (dd, *J* = 14.8, 10.8 Hz, 1H), 5.81 (d, *J* = 10.8 Hz, 1H), 5.56-5.46 (m, 1H), 2.85-2.79 (m, 1H), 2.79-2.71 (m, 1H), 2.69-2.59 (m, 1H), 2.35-2.26 (m, 1H), 2.12-2.02 (m, 1H), 1.82-1.71 (m, 7H), 1.67-1.56 (m, 1H), 1.30 (brs, 2H); ^{13}C NMR (100 MHz, $CDCl_3$) δ 142.4, 133.9, 129.7, 128.5, 128.0, 125.9, 125.0, 50.9, 41.9, 39.6, 32.8, 26.0, 18.4; HRMS (ESI): *m/z* Calcd. For $C_{16}H_{24}N$ $(M + H)^+$: 230.1903; Found: 230.1901.

Compound 3u (Fig. 3)

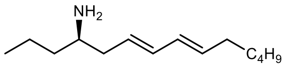

3u

The product was obtained in 78% yield (48.6 mg, 0.233 mmol, 93% ee) as a pale yellow oil from the corresponding primary amine **1** (0.45 mmol) and allylic carbonate **2** (0.30 mmol); $[\alpha]_D^{25}$ = -5.1 (*c* = 0.10, MeOH); ^{1}H NMR (400 MHz, $CDCl_3$) δ 6.11-5.94 (m, 2H), 5.66-5.46 (m, 2H), 2.82-2.71 (m, 1H), 2.27-2.17 (m, 1H), 2.04 (q, *J* = 7.2 Hz, 2H), 2.01-1.91 (m, 1H), 1.45-1.20 (m, 12H), 0.91 (t, *J* = 6.8 Hz, 3H), 0.87 (t, *J* = 6.8 Hz, 3H); ^{13}C NMR (100 MHz, $CDCl_3$) δ 133.5, 133.2, 130.2, 128.8, 51.0, 41.5, 40.0, 32.7, 31.6, 29.2, 22.7, 19.5, 14.3, 14.2; HRMS (ESI): *m/z* Calcd. For $C_{14}H_{28}N$ $(M + H)^+$: 210.2216; Found: 210.2220.

Compound 3v (Fig. 3)

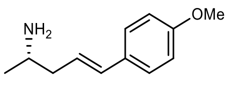

3v

The product was obtained in 79% yield (91.0 mg, 0.476 mmol, 91% ee) as a brown solid from the corresponding primary amine **1** (0.90 mmol) and allylic carbonate **2** (0.60 mmol); M.p. 35-36 °C; $[\alpha]_D^{25}$ = +23.4 (c = 0.10, MeOH); ^{1}H NMR (400 MHz, $CDCl_3$) δ 7.28 (d, *J* = 8.8 Hz, 2H), 6.84 (d, *J* = 8.8 Hz, 2H), 6.38 (d, *J* = 15.6 Hz, 1H), 6.11-5.98 (m, 1H), 3.79 (s, 3H), 3.08-2.97 (m, 1H), 2.33-2.22 (m, 1H), 2.20-2.08 (m, 1H), 1.50 (brs, 2H), 1.11 (d, *J* = 6.0 Hz, 3H); ^{13}C NMR (100 MHz, $CDCl_3$) δ 158.9, 131.9, 130.4, 127.2, 125.5, 114.0, 55.4, 47.0, 43.9, 23.7; HRMS (ESI): *m/z* Calcd. For $C_{12}H_{18}NO$ (M + H)$^+$: 192.1383; Found: 192.1379.

Compound 3w (Fig. 3)

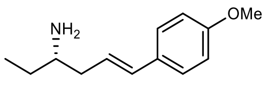

3w

The product was obtained in 80% yield (49.0 mg, 0.239 mmol, 96% ee) as a brown solid from the corresponding primary amine **1** (0.45 mmol) and allylic carbonate **2** (0.30 mmol); M.p. 46-48 °C; $[\alpha]_D^{25}$ = +33.8 (*c* = 0.10, MeOH); ^{1}H NMR (400 MHz, $CDCl_3$) δ 7.29 (d, *J* = 8.4 Hz, 2H), 6.83 (d, *J* = 8.0 Hz, 2H), 6.39 (d, *J* = 16.0 Hz, 1H), 6.11-5.99 (m, 1H), 3.79 (s, 3H), 2.82-2.70 (m, 1H), 2.41-2.30 (m, 1H), 2.16-2.05 (m, 1H), 1.60 (brs, 2H), 1.54-1.44 (m, 1H), 1.42-1.30 (m, 1H), 0.95 (t, *J* = 7.2 Hz, 3H); ^{13}C NMR (100 MHz, $CDCl_3$) δ 158.9, 131.9, 130.4, 127.3, 125.5, 114.1, 55.4, 52.8, 41.3, 30.5, 10.8; HRMS (ESI): *m/z* Calcd. For $C_{13}H_{20}NO$ (M + H)$^+$: 206.1539; Found: 206.1541.

Compound 3x (Fig. 3)

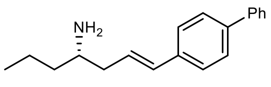

3x

The product was obtained in 80% yield (63.6 mg, 0.240 mmol, 96% ee) as a yellow solid from the corresponding primary amine **1** (0.45 mmol) and allylic carbonate **2** (0.30 mmol); M.p. 85-87 °C; $[\alpha]_D^{25}$ = +24.8 (c = 0.10, MeOH); ^{1}H NMR (400 MHz, $CDCl_3$) δ 7.59 (d, *J* = 7.6 Hz, 2H), 7.55 (d, *J* = 7.2 Hz, 2H), 7.47-7.39 (m, 4H), 7.34 (t, *J* = 7.6 Hz, 1H), 6.49 (d, *J* = 16.0 Hz, 1H), 6.32-6.20 (m, 1H), 2.96-2.84 (m, 1H), 2.45-2.35 (m, 1H), 2.23-2.12 (m, 1H), 1.58 (brs, 2H), 1.50-1.42 (m, 2H), 1.42-1.31

(m, 2H), 0.95 (t, J = 6.0 Hz, 3H); ^{13}C NMR (100 MHz, $CDCl_3$) δ 140.9, 140.0, 136.6, 132.1, 128.9, 127.9, 127.4, 127.0, 126.6, 50.9, 42.0, 40.1, 19.6, 14.3; HRMS (ESI): *m/z* Calcd. For $C_{19}H_{24}N$ $(M + H)^+$: 266.1903; Found: 266.1905.

Compound 3y (Fig. 3)

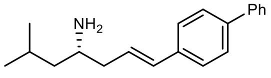

3y

The product was obtained in 85% yield (70.9 mg, 0.254 mmol, 96% ee) as a yellow solid from the corresponding primary amine **1** (0.45 mmol) and allylic carbonate **2** (0.30 mmol); M.p. 69-70 °C; $[\alpha]_D^{25}$ = +22.8 (c = 0.10, MeOH); ^{1}H NMR (400 MHz, $CDCl_3$) δ 7.60 (d, J = 8.0 Hz, 2H), 7.55 (d, J = 7.6 Hz, 2H), 7.48-7.40 (m, 4H), 7.34 (t, J = 7.2 Hz, 1H), 6.50 (d, J = 15.6 Hz, 1H), 6.32-6.22 (m, 1H), 3.02-2.92 (m, 1H), 2.44-2.34 (m, 1H), 2.22-2.10 (m, 1H), 1.84-1.69 (m, 1H), 1.37 (brs, 2H), 1.29 (t, J = 6.4 Hz, 2H), 0.94 (d, J = 7.6 Hz, 3H), 0.93 (d, J = 7.2 Hz, 3H); ^{13}C NMR (100 MHz, $CDCl_3$) δ 140.9, 140.0, 136.6, 132.1, 128.9, 127.9, 127.4, 127.0, 126.6, 48.9, 47.3, 42.5, 25.0, 23.6, 22.2; HRMS (ESI): *m/z* Calcd. For $C_{20}H_{26}N$ $(M + H)^+$: 280.2060; Found: 280.2062.

Compound 3z (Fig. 3)

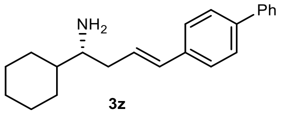

3z

The product was obtained in 60% yield (54.9 mg, 0.180 mmol, 97% ee) as a yellow solid from the corresponding primary amine **1** (0.45 mmol) and allylic carbonate **2** (0.30 mmol); M.p. 102-104 °C; $[\alpha]_D^{25}$ = +26.0 (c = 0.10, MeOH); ^{1}H NMR (400 MHz, $CDCl_3$) δ 7.60 (d, J = 7.6 Hz, 2H), 7.55 (d, J = 8.4 Hz, 2H), 7.48-7.40 (m, 4H), 7.34 (t, J = 7.2 Hz, 1H), 6.50 (d, J = 16.0 Hz, 1H), 6.34-6.20 (m, 1H), 2.76-2.60 (m, 1H), 2.52-2.40 (m, 1H), 2.22-2.10 (m, 1H), 1.88-1.63 (m, 5H), 1.40-0.98 (m, 8H); ^{13}C NMR (100 MHz, $CDCl_3$) δ 140.9, 139.9, 136.7, 131.9, 128.9, 128.7, 127.3, 127.0, 126.6, 55.9, 43.6, 38.8, 29.9, 28.4, 26.8, 26.6, 26.5; HRMS (ESI): *m/z* Calcd. For $C_{22}H_{28}N$ $(M + H)^+$: 306.2216; Found: 306.2220.

Compound 3aa (Fig. 3)

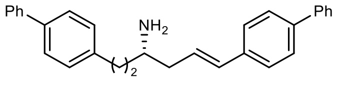

3aa

The product was obtained in 83% yield (100.8 mg, 0.250 mmol, 97% ee) as a yellow solid from the corresponding primary amine **1** (0.45 mmol) and allylic carbonate **2** (0.30 mmol); M.p. 169-171 °C; $[\alpha]_D^{25}$ = +12.3 (c = 0.10, MeOH); ^{1}H NMR (400 MHz, $CDCl_3$) δ 7.63-7.57 (m, 4H), 7.57-7.50 (m, 4H), 7.47-7.40 (m, 6H), 7.37-7.27 (m, 4H), 6.52 (d, J = 16.0 Hz, 1H), 6.32-6.21 (m, 1H), 3.02-2.92 (m, 1H), 2.91-2.79 (m, 1H), 2.79-2.68 (m, 1H), 2.53-2.42 (m, 1H), 2.32-2.22 (m, 1H), 1.94-1.81 (m, 1H), 1.79-1.65 (m, 1H), 1.48 (brs, 2H); ^{13}C NMR (100 MHz, $CDCl_3$) δ 141.4, 141.2, 140.9, 140.1, 139.0, 136.6, 132.4, 129.0, 128.92, 128.87, 127.6, 127.4, 127.3, 127.2, 127.14, 127.06, 126.6, 50.9, 42.1, 39.6, 32.5; HRMS (ESI): m/z Calcd. For $C_{30}H_{30}N$ $(M + H)^+$: 404.2373; Found: 404.2377.

Compound 3ab (Fig. 3)

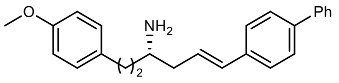

3ab

The product was obtained in 86% yield (92.6 mg, 0.259 mmol, 97% ee) as a pale yellow solid from the corresponding primary amine **1** (0.45 mmol) and allylic carbonate **2** (0.30 mmol); M.p. 119-121 °C; $[\alpha]_D^{25}$ = +3.4 (c = 0.10, MeOH); ^{1}H NMR (400 MHz, $CDCl_3$) δ 7.59 (d, J = 8.0 Hz, 2H), 7.55 (d, J = 8.0 Hz, 2H), 7.47-7.39 (m, 4H), 7.34 (t, J = 7.2 Hz, 1H), 7.13 (d, J = 8.4 Hz, 2H), 6.84 (d, J = 8.4 Hz, 2H), 6.50 (d, J = 16.0 Hz, 1H), 6.32-6.18 (m, 1H), 3.79 (s, 3H), 2.97-2.87 (m, 1H), 2.79-2.69 (m, 1H), 2.68-2.58 (m, 1H), 2.49-2.39 (m, 1H), 2.28-2.17 (m, 1H), 1.86-1.74 (m, 1H), 1.70-1.58 (m, 1H), 1.46 (brs, 2H); ^{13}C NMR (100 MHz, $CDCl_3$) δ 157.9, 140.9, 140.0, 136.5, 134.3, 132.3, 129.4, 128.9, 127.6, 127.4, 127.0, 126.6, 113.9, 55.4, 50.7, 42.1, 39.8, 31.9; HRMS (ESI): m/z Calcd. For $C_{25}H_{28}NO$ $(M + H)^+$: 358.2165; Found: 358.2169.

Compound 3ac (Fig. 3)

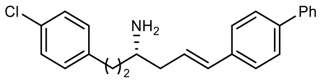

3ac

The product was obtained in 83% yield (90.0 mg, 0.249 mmol, 96% ee) as a yellow solid from the corresponding primary amine **1** (0.45 mmol) and allylic carbonate **2** (0.30 mmol); M.p. 143-144 °C;

[α]$_D^{25}$ = +6.8 (c = 0.10, MeOH); ^{1}H NMR (400 MHz, $CDCl_3$) δ 7.60 (d, J = 7.2 Hz, 2H), 7.55 (d, J = 8.0 Hz, 2H), 7.47-7.40 (m, 4H), 7.34 (t, J = 7.2 Hz, 1H), 7.25 (d, J = 8.0 Hz, 2H), 7.14 (d, J = 8.0 Hz, 2H), 6.50 (d, J = 16.0 Hz, 1H), 6.29-6.18 (m, 1H), 2.96-2.86 (m, 1H), 2.82-2.71 (m, 1H), 2.71-2.59 (m, 1H), 2.49-2.38 (m, 1H), 2.28-2.16 (m, 1H), 1.85-1.73 (m, 1H), 1.70-1.57 (m, 1H), 1.37 (brs, 2H); ^{13}C NMR (100 MHz, $CDCl_3$) δ 140.9, 140.7, 140.1, 136.5, 132.4, 131.7, 129.9, 128.9, 128.6, 127.4, 127.0, 126.6, 50.7, 42.1, 39.4, 32.2; HRMS (ESI): m/z Calcd. For $C_{24}H_{25}ClN$ $(M + H)^+$: 362.1670; Found: 362.1671.

Compound 3ad (Fig. 3)

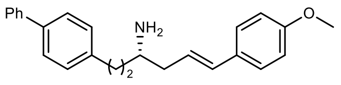

3ad

The product was obtained in 87% yield (93.7 mg, 0.262 mmol, 94% ee) as a pale yellow solid from the corresponding primary amine **1** (0.45 mmol) and allylic carbonate **2** (0.30 mmol); M.p. 110-111 °C; [α]$_D^{25}$ = +12.0 (c = 0.10, MeOH); ^{1}H NMR (400 MHz, $CDCl_3$) δ 7.58 (d, J = 7.2 Hz, 2H), 7.52 (d, J = 7.6 Hz, 2H), 7.43 (t, J = 7.6 Hz, 2H), 7.34 (d, J = 7.6 Hz, 1H), 7.32-7.26 (m, 4H), 6.85 (d, J = 8.8 Hz, 2H), 6.42 (d, J = 15.6 Hz, 1H), 6.11-6.01 (m, 1H), 3.80 (s, 3H), 3.00-2.89 (m, 1H), 2.89-2.78 (m, 1H), 2.77-2.66 (m, 1H), 2.50-2.37 (m, 1H), 2.28-2.16 (m, 1H), 2.06 (brs, 2H), 1.92-1.79 (m, 1H), 1.78-1.65 (m, 1H); ^{13}C NMR (100 MHz, $CDCl_3$) δ 159.1, 141.4, 141.2, 139.0, 132.3, 130.3, 128.95, 128.85, 127.33, 127.29, 127.2, 127.1, 124.9, 114.1, 55.4, 50.9, 41.7, 39.2, 32.4; HRMS (ESI): m/z Calcd. For $C_{25}H_{28}NO$ $(M + H)^+$: 358.2165; Found: 358.2170.

Compound 3ae (Fig. 3)

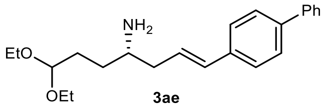

3ae

The product was obtained in 67% yield (71.0 mg, 0.201 mmol, 96% ee) as a brown oil from the corresponding primary amine **1** (0.45 mmol) and allylic carbonate **2** (0.30 mmol); [α]$_D^{25}$ = +10.5 (c = 0.10, MeOH); ^{1}H NMR (400 MHz, $CDCl_3$) δ 7.59 (d, J = 7.6 Hz, 2H), 7.55 (d, J = 7.6 Hz, 2H), 7.47-7.39 (m, 4H), 7.33 (t, J = 7.2 Hz, 1H), 6.50 (d, J = 16.0 Hz, 1H), 6.31-6.20 (m, 1H), 4.51 (t, J = 4.8 Hz, 1H), 3.72-3.60 (m, 2H), 3.56-3.45 (m, 2H), 2.95-2.85 (m, 1H), 2.47-2.36 (m, 1H), 2.24-2.13 (m, 1H), 1.84-1.64 (m, 2H), 1.63-1.36 (m, 4H), 1.21 (t, J = 7.2 Hz, 6H); ^{13}C NMR (100 MHz, $CDCl_3$) δ

140.8, 140.0, 136.5, 132.3, 128.9, 127.6, 127.3, 127.0, 126.6, 103.1, 61.3, 61.1, 51.1, 41.8, 32.7, 30.5, 15.5; HRMS (ESI): *m/z* Calcd. For $C_{23}H_{32}NO_2$ $(M + H)^+$: 354.2428; Found: 354.2430.

Compound 3af (Fig. 3)

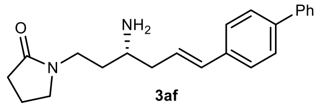

3af

The product was obtained in 86% yield (86.2 mg, 0.258 mmol, 95% ee) as a pale yellow solid from the corresponding primary amine **1** (0.45 mmol) and allylic carbonate **2** (0.30 mmol); M.p. 81-83 °C; $[\alpha]_D^{25}$ = +29.3 (c = 0.10, MeOH); ^{1}H NMR (400 MHz, $CDCl_3$) δ 7.59 (d, J = 7.6 Hz, 2H), 7.55 (d, J = 8.0 Hz, 2H), 7.47-7.39 (m, 4H), 7.33 (t, J = 7.6 Hz, 1H), 6.50 (d, J = 15.6 Hz, 1H), 6.28-6.17 (m, 1H), 3.69-3.58 (m, 1H), 3.47-3.30 (m, 2H), 3.28-3.17 (m, 1H), 2.92-2.81 (m, 1H), 2.45-2.35 (m, 3H), 2.29-2.18 (m, 1H), 2.09-1.96 (m, 2H), 1.77-1.69 (m, 3H), 1.55-1.43 (m, 1H); ^{13}C NMR (100 MHz, $CDCl_3$) δ 175.4, 140.8, 140.1, 136.4, 132.6, 128.9, 127.4, 127.2, 127.0, 126.6, 48.5, 47.3, 41.9, 39.8, 34.9, 31.1, 18.0; HRMS (ESI): *m/z* Calcd. For $C_{22}H_{27}N_2O$ $(M + H)^+$: 335.2118; Found: 335.2121.

Compound 3ag (Fig. 3)

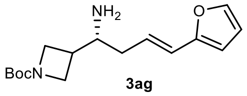

3ag

The product was obtained in 87% yield (76.0 mg, 0.260 mmol, 91% ee) as an orange oil from the corresponding primary amine **1** (0.45 mmol) and allylic carbonate **2** (0.30 mmol); $[\alpha]_D^{25}$ = +8.9 (c = 0.10, MeOH); ^{1}H NMR (400 MHz, $CDCl_3$) δ 7.33-7.28 (m, 1H), 6.37-6.31 (m, 1H), 6.25 (d, J = 15.6 Hz, 1H), 6.19-6.13 (m, 1H), 6.11-6.01 (m, 1H), 4.02-3.90 (m, 2H), 3.80-3.70 (m, 1H), 3.70-3.61 (m, 1H), 3.05-2.94 (m, 1H), 2.52-2.38 (m, 1H), 2.36-2.23 (m, 1H), 2.10-1.98 (m, 1H), 1.56 (brs, 2H), 1.42 (s, 9H); ^{13}C NMR (100 MHz, $CDCl_3$) δ 156.4, 152.6, 141.8, 125.1, 121.8, 111.3, 107.1, 79.5, 54.2, 52.3, 51.5, 38.9, 35.0, 28.5; HRMS (ESI): *m/z* Calcd. For $C_{16}H_{25}N_2O_3$ $(M + H)^+$: 293.1860; Found: 293.1861.

Compound (*R*)-3ah (Fig. 3)

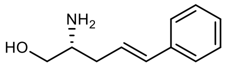

(*R*)-3ah

The product was obtained in 67% yield (71.2 mg, 0.402 mmol, 96% ee) as a pale yellow solid from the corresponding primary amine **1** (0.90 mmol) and allylic carbonate **2** (0.60 mmol); M.p. 111-113 °C; $[\alpha]_D^{25}$ = +16.0 (*c* = 0.10, MeOH); ^{1}H NMR (400 MHz, CD_3OD) δ 7.38 (d, *J* = 7.2 Hz, 2H), 7.28 (t, *J* = 7.2 Hz, 2H), 7.19 (t, *J* = 7.6 Hz, 1H), 6.50 (d, *J* = 16.0 Hz, 1H), 6.33-6.21 (m, 1H), 3.64-3.57 (m, 1H), 3.47-3.39 (m, 1H), 3.03-2.92 (m, 1H), 2.47-2.36 (m, 1H), 2.31-2.20 (m, 1H); ^{13}C NMR (100 MHz, CD_3OD) δ 138.8, 134.2, 129.5, 128.2, 127.19, 127.16, 66.4, 53.9, 37.6; HRMS (ESI): *m/z* Calcd. For $C_{11}H_{16}NO$ $(M + H)^+$: 178.1226; Found: 178.1227.

Compound (*S*)-3ah (Fig. 3)

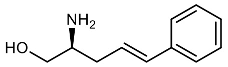

(*S*)-**3ah**

The product was obtained in 64% yield (67.5 mg, 0.381 mmol, 96% ee) as a pale yellow solid from the corresponding primary amine **1** (0.90 mmol) and allylic carbonate **2** (0.60 mmol); M.p. 110-111 °C; $[\alpha]_D^{25}$ = -24.0 (*c* = 0.10, MeOH); ^{1}H NMR (400 MHz, CD_3OD) δ 7.38 (d, *J* = 7.2 Hz, 2H), 7.28 (t, *J* = 7.6 Hz, 2H), 7.18 (t, *J* = 7.6 Hz, 1H), 6.48 (d, *J* = 15.6 Hz, 1H), 6.33-6.22 (m, 1H), 3.58 (dd, *J* = 10.8, 4.8 Hz, 1H), 3.40 (dd, *J* = 10.8, 6.8 Hz, 1H), 2.97-2.87 (m, 1H), 2.45-2.34 (m, 1H), 2.287-2.16 (m, 1H); ^{13}C NMR (100 MHz, CD_3OD) δ 138.8, 133.9, 129.5, 128.1, 127.7, 127.1, 67.1, 53.9, 38.1; HRMS (ESI): *m/z* Calcd. For $C_{11}H_{16}NO$ $(M + H)^+$: 178.1226; Found: 178.1229.

Compound 3ai (Fig. 3)

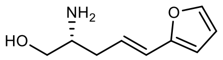

3ai

The product was obtained in 83% yield (125.0 mg, 0.749 mmol, 94% ee) as an orange oil from the corresponding primary amine **1** (1.35 mmol) and allylic carbonate **2** (0.90 mmol); $[\alpha]_D^{25}$ = +22.0 (*c* = 0.10, MeOH); ^{1}H NMR (400 MHz, $CDCl_3$) δ 7.34-7.29 (m, 1H), 6.37-6.32 (m, 1H), 6.28 (d, *J* = 16.0 Hz, 1H), 6.19-6.14 (m, 1H), 6.14-6.03 (m, 1H), 3.70-3.59 (m, 1H), 3.44-3.33 (m, 1H), 3.06-2.93 (m, 1H), 2.45-2.21 (m, 4H), 2.21-2.10 (m, 1H); ^{13}C NMR (100 MHz, $CDCl_3$) δ 152.7, 141.8, 125.3, 121.6, 111.3, 107.1, 66.3, 52.6, 37.9; HRMS (ESI): *m/z* Calcd. For $C_9H_{14}NO_2$ $(M + H)^+$: 168.1019; Found: 168.1021.

Compound 3aj (Fig. 3)

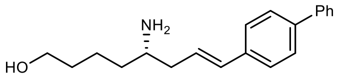

3aj

The product was obtained in 52% yield (46.0 mg, 0.156 mmol, 92% ee) as a colorless oil from the corresponding primary amine **1** (0.45 mmol) and allylic carbonate **2** (0.30 mmol); Colorless oil; $[\alpha]_D^{25}$ = +4.9 (*c* = 0.10, MeOH); ^{1}H NMR (400 MHz, CD_3OD) δ 7.61 (d, *J* = 7.6 Hz, 2H), 7.57 (d, *J* = 8.0 Hz, 2H), 7.47 (d, *J* = 8.0 Hz, 2H), 7.42 (t, *J* = 8.0 Hz, 2H), 7.31 (t, *J* = 7.6 Hz, 1H), 6.53 (d, *J* = 15.6 Hz, 1H), 6.38-6.24 (m, 1H), 3.58 (t, *J* = 6.0 Hz, 2H), 3.02-2.91 (m, 1H), 2.50-2.40 (m, 1H), 2.35-2.23 (m, 1H), 1.65-1.40 (m, 6H); ^{13}C NMR (100 MHz, CD_3OD) δ 142.0, 141.2, 137.8, 133.9, 129.9, 128.3, 128.0, 127.72, 127.69, 127.5, 62.7, 52.2, 41.0, 36.9, 33.6, 23.3; HRMS (ESI): *m/z* Calcd. For $C_{20}H_{26}NO$ $(M + H)^+$: 296.2009; Found: 296.2012.

Compound 3ak (Fig. 3)

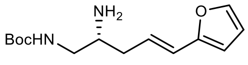

3ak

The product was obtained in 52% yield (41.6 mg, 0.156 mmol, 93% ee) as a brown oil from the corresponding primary amine **1** (0.45 mmol) and allylic carbonate **2** (0.30 mmol); $[\alpha]_D^{25}$ = +7.5 (*c* = 0.10, MeOH); ^{1}H NMR (400 MHz, $CDCl_3$) δ 7.33-7.28 (m, 1H), 6.36-6.31 (m, 1H), 6.27 (d, *J* = 16.0 Hz, 1H), 6.17-6.13 (m, 1H), 6.13-6.03 (m, 1H), 5.05 (brs, 1H), 3.32-3.20 (m, 1H), 3.03-2.88 (m, 2H), 2.40-2.29 (m, 1H), 2.18-2.06 (m, 1H), 1.77 (brs, 2H), 1.43 (s, 9H); ^{13}C NMR (100 MHz, $CDCl_3$) δ 156.4, 152.7, 141.7, 125.3, 121.6, 111.3, 107.0, 79.4, 51.2, 46.7, 39.3, 28.5; HRMS (ESI): *m/z* Calcd. For $C_{14}H_{23}N_2O_3$ $(M + H)^+$: 267.1703; Found: 267.1706.

Compound 3al (Fig. 3)

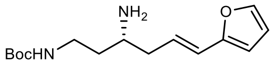

3al

The product was obtained in 80% yield (67.0 mg, 0.239 mmol, 93% ee) as a brown oil from the corresponding primary amine **1** (0.45 mmol) and allylic carbonate **2** (0.30 mmol); $[\alpha]_D^{25}$ = +13.6 (*c* = 0.10, MeOH); ^{1}H NMR (400 MHz, $CDCl_3$) δ 7.33-7.28 (m, 1H), 6.37-6.32 (m, 1H), 6.26 (d, *J* = 16.4 Hz, 1H), 6.18-6.14 (m, 1H), 6.14-6.04 (m, 1H), 5.12 (brs, 1H), 3.40-3.25 (m, 1H), 3.25-3.14 (m, 1H), 2.97-2.86 (m, 1H), 2.39-2.29 (m, 1H), 2.21-2.10 (m, 1H), 1.73-1.61 (m, 4H), 1.44 (s, 9H); ^{13}C NMR

(100 MHz, $CDCl_3$) δ 156.3, 152.8, 141.7, 125.9, 121.5, 111.3, 106.9, 79.3, 49.6, 42.3, 38.4, 37.3, 28.6; HRMS (ESI): *m/z* Calcd. For $C_{15}H_{25}N_2O_3$ $(M + H)^+$: 281.1860; Found: 281.1863.

Compound 3am (Fig. 3)

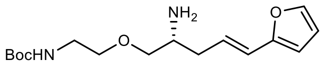

3am

The product was obtained in 84% yield (78.0 mg, 0.252 mmol, 93% ee) as a brown oil from the corresponding primary amine **1** (0.45 mmol) and allylic carbonate **2** (0.30 mmol); $[\alpha]_D^{25}$ = +3.1 (*c* = 0.10, MeOH); ^{1}H NMR (400 MHz, $CDCl_3$) δ 7.34-7.28 (m, 1H), 6.38-6.32 (m, 1H), 6.28 (d, *J* = 16.0 Hz, 1H), 6.19-6.15 (m, 1H), 6.15-6.04 (m, 1H), 4.96 (brs, 1H), 3.59-3.48 (m, 2H), 3.48-3.42 (m, 1H), 3.37-3.29 (m, 2H), 3.29-3.23 (m, 1H), 3.14-3.04 (m, 1H), 2.39-2.28 (m, 1H), 2.23-2.10 (m, 1H), 1.78 (brs, 2H), 1.44 (s, *J* = 2.5 Hz, 9H); ^{13}C NMR (100 MHz, $CDCl_3$) δ 156.1, 152.8, 141.7, 125.6, 121.4, 111.3, 107.0, 79.5, 75.9, 70.4, 50.8, 40.5, 37.9, 28.5; HRMS (ESI): *m/z* Calcd. For $C_{16}H_{27}N_2O_4$ $(M + H)^+$: 311.1965; Found: 311.1967.

Compound 3an (Fig. 3)

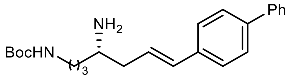

3an

The product was obtained in 50% yield (57.0 mg, 0.150 mmol, 98% ee) as a yellow solid from the corresponding primary amine **1** (0.45 mmol) and allylic carbonate **2** (0.30 mmol); M.p. 116-117 °C; $[\alpha]_D^{25}$ = +11.1 (*c* = 0.10, MeOH); ^{1}H NMR (400 MHz, $CDCl_3$) δ 7.59 (d, *J* = 7.2 Hz, 2H), 7.54 (d, *J* = 7.6 Hz, 2H), 7.47-7.39 (m, 4H), 7.37-7.29 (m, 1H), 6.49 (d, *J* = 15.6 Hz, 1H), 6.30-6.17 (m, 1H), 4.73 (brs, 1H), 3.21-3.08 (m, 2H), 2.95-2.85 (m, 1H), 2.46-2.34 (m, 1H), 2.25-2.12 (m, 1H), 1.74-1.49 (m, 4H), 1.49-1.39 (m, 10H), 1.39-1.31 (m, 1H); ^{13}C NMR (100 MHz, $CDCl_3$) δ 156.1, 140.9, 140.1, 136.5, 132.4, 128.9, 127.44, 127.35, 127.0, 126.6, 79.2, 51.0, 42.0, 40.7, 34.8, 28.6, 27.0; HRMS (ESI): *m/z* Calcd. For $C_{24}H_{33}N_2O_2$ $(M + H)^+$: 381.2537; Found: 381.2539.

Compound 3ao (Fig. 3)

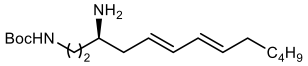

3ao

The product was obtained in 67% yield (62.3 mg, 0.201 mmol, 92% ee) as a pale yellow oil from the

corresponding primary amine **1** (0.45 mmol) and allylic carbonate **2** (0.30 mmol); $[\alpha]_D^{25}$ = -10.1 (*c* = 0.10, MeOH); ^1H NMR (400 MHz, $CDCl_3$) δ 6.12-5.91 (m, 2H), 5.66-5.56 (m, 1H), 5.54-5.43 (m, 1H), 5.14 (brs, 1H), 3.39-3.24 (m, 1H), 3.24-3.10 (m, 1H), 2.91-2.76 (m, 1H), 2.27-2.16 (m, 1H), 2.11-1.97 (m, 3H), 1.70-1.57 (m, 1H), 1.50-1.20 (m, 18H), 0.88 (t, *J* = 6.4 Hz, 3H); ^{13}C NMR (100 MHz, $CDCl_3$) δ 156.3, 133.9, 133.7, 130.0, 127.9, 79.2, 49.8, 42.1, 38.4, 37.2, 32.7, 31.6, 29.1, 28.6, 22.7, 14.2; HRMS (ESI): *m/z* Calcd. For $C_{18}H_{35}N_2O_2$ $(M + H)^+$: 311.2693; Found: 311.2697.

Compound 3ap (Fig. 3)

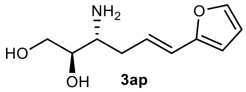

3ap

The product was obtained in 60% yield (71.4 mg, 0.362 mmol, 97:3 dr) as an orange oil from the corresponding primary amine **1** (0.90 mmol) and allylic carbonate **2** (0.60 mmol); $[\alpha]_D^{25}$ = +33.00 (*c* = 0.10, MeOH); ^1H NMR (400 MHz, CD_3OD) δ 7.40-7.36 (m, 1H), 6.40-6.31 (m, 2H), 6.24-6.12 (m, 2H), 3.71-3.59 (m, 2H), 3.57-3.49 (m, 1H), 2.97-2.89 (m, 1H), 2.59-2.49 (m, 1H), 2.29-2.17 (m, 1H); ^{13}C NMR (100 MHz, CD_3OD) δ 154.3, 142.8, 126.7, 122.7, 112.1, 107.7, 75.4, 64.7, 54.5, 37.0; HRMS (ESI): *m/z* Calcd. For $C_{10}H_{16}NO_3$ $(M + H)^+$: 198.1125; Found: 198.1128.

Compound 3aq (Fig. 3)

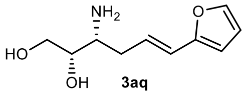

3aq

The product was obtained in 60% yield (70.4 mg, 0.357 mmol, 99:1 dr) as an orange oil from the corresponding primary amine **1** (0.90 mmol) and allylic carbonate **2** (0.60 mmol); $[\alpha]_D^{25}$ = +8.0 (*c* = 0.10, MeOH); ^1H NMR (400 MHz, CD_3OD) δ 7.39-7.36 (m, 1H), 6.39-6.31 (m, 2H), 6.24-6.20 (m, 1H), 6.20-6.10 (m, 1H), 3.69-3.59 (m, 2H), 3.58-3.51 (m, 1H), 2.97-2.89 (m, 1H), 2.52-2.40 (m, 1H), 2.33-2.21 (m, 1H); ^{13}C NMR (100 MHz, CD_3OD) δ 154.2, 142.8, 126.4, 122.6, 112.1, 107.8, 74.2, 65.0, 53.7, 38.1; HRMS (ESI): *m/z* Calcd. For $C_{10}H_{16}NO_3$ $(M + H)^+$: 198.1125; Found: 198.1127.

Compound 3ar (Fig. 3)

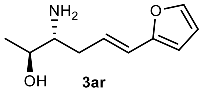

3ar

The product was obtained in 53% yield (57.7 mg, 0.319 mmol, 99:1 dr) as an orange oil from the corresponding primary amine **1** (0.90 mmol) and allylic carbonate **2** (0.60 mmol); $[\alpha]_D^{25}$ = +57.0 (*c* = 0.10, MeOH); ^{1}H NMR (400 MHz, $CDCl_3$) δ 7.35-7.28 (m, 1H), 6.37-6.31 (m, 1H), 6.27 (d, *J* = 15.6 Hz, 1H), 6.18-6.03 (m, 2H), 3.81-3.69 (m, 1H), 2.92-2.82 (m, 1H), 2.44-2.33 (m, 1H), 2.20-1.90 (m, 4H), 1.14 (d, *J* = 5.6 Hz, 3H); ^{13}C NMR (100 MHz, $CDCl_3$) δ 152.8, 141.6, 126.4, 121.4, 111.3, 106.9, 69.8, 55.8, 35.9, 17.7; HRMS (ESI): *m/z* Calcd. For $C_{10}H_{16}NO_2$ $(M + H)^+$: 182.1176; Found: 182.1177.

Compound 3as (Fig. 3)

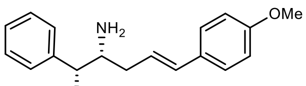

3as

The product was obtained in 53% yield (45.0 mg, 0.160 mmol, 99:1 dr) as an orange oil from the corresponding primary amine **1** (0.45 mmol) and allylic carbonate **2** (0.30 mmol); $[\alpha]_D^{25}$ = +43.3 (*c* = 0.10, MeOH); ^{1}H NMR (400 MHz, $CDCl_3$) δ 7.32 (t, *J* = 7.2 Hz, 2H), 7.27 (d, *J* =7.6 Hz, 2H), 7.25-7.19 (m, 3H), 6.84 (d, *J* = 8.0 Hz, 2H), 6.35 (d, *J* = 15.6 Hz, 1H), 6.07-5.95 (m, 1H), 3.80 (s, 3H), 3.05-2.95 (m, 1H), 2.79-2.68 (m, 1H), 2.35-2.23 (m, 1H), 2.07-1.93 (m, 1H), 1.54 (brs, 2H), 1.33 (d, *J* = 6.4 Hz, 3H); ^{13}C NMR (100 MHz, $CDCl_3$) δ 158.9, 145.3, 132.0, 130.4, 128.5, 127.9, 127.2, 126.4, 125.6, 114.0, 56.7, 55.4, 45.7, 39.3, 16.7; HRMS (ESI): *m/z* Calcd. For $C_{19}H_{24}NO$ $(M + H)^+$: 282.1852; Found: 282.1855.

Compound 3at (Fig. 3)

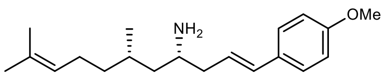

3at

The product was obtained in 87% yield (78.6 mg, 0.261 mmol, 97:3 dr) as a pale yellow oil from the corresponding primary amine **1** (0.45 mmol) and allylic carbonate **2** (0.30 mmol); $[\alpha]_D^{25}$ = +42.0 (*c* = 0.10, MeOH); ^{1}H NMR (400 MHz, $CDCl_3$) δ 7.29 (d, *J* = 8.8 Hz, 2H), 6.84 (d, *J* = 8.8 Hz, 2H), 6.40 (d, *J* = 15.6 Hz, 1H), 6.11-6.01 (m, 1H), 5.14-5.06 (m, 1H), 3.80 (s, 3H), 3.00-2.90 (m, 1H), 2.40-2.30 (m, 1H), 2.12-1.88 (m, 3H), 1.68 (s, 3H), 1.64-1.54 (m, 4H), 1.44-1.27 (m, 4H), 1.28-1.08 (m, 2H), 0.92 (d, *J* = 6.8 Hz, 3H); ^{13}C NMR (100 MHz, $CDCl_3$) δ 158.9, 132.1, 131.4, 130.4, 127.3, 125.4, 124.9, 114.0, 55.4, 48.7, 45.5, 41.8, 36.8, 29.5, 25.9, 25.5, 20.4, 17.8; HRMS (ESI): *m/z* Calcd. For $C_{20}H_{32}NO$ $(M + H)^+$: 302.2478; Found: 302.2482.

Compound 3au (Fig. 3)

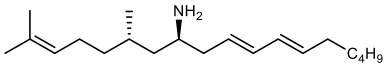

3au

The product was obtained in 78% yield (68.1 mg, 0.234 mmol, 96:4 dr) as a brown oil from the corresponding primary amine **1** (0.45 mmol) and allylic carbonate **2** (0.30 mmol); $[\alpha]_D^{25}$ = +23.3 (*c* = 0.10, MeOH)); ^{1}H NMR (400 MHz, $CDCl_3$) δ 6.11-5.94 (m, 2H), 5.64-5.45 (m, 2H), 5.08 (t, *J* = 7.2 Hz, 1H), 2.93-2.80 (m, 1H), 2.27-2.17 (m, 1H), 2.08-1.88 (m, 7H), 1.67 (s, 3H), 1.59 (s, 3H), 1.57-1.48 (m, 1H), 1.42-1.21 (m, 8H), 1.19-1.05 (m, 2H), 0.92-0.83 (m, 6H); ^{13}C NMR (100 MHz, $CDCl_3$) δ 133.6, 133.5, 131.3, 130.1, 128.3, 124.9, 48.8, 45.1, 41.1, 36.9, 32.7, 31.5, 29.4, 29.1, 25.8, 25.4, 22.6, 20.3, 17.8, 14.1; HRMS (ESI): *m/z* Calcd. For $C_{20}H_{38}NO$ $(M + H)^+$: 292.2999; Found: 292.3002.

Compound 3av (Fig. 3)

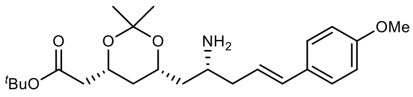

3av

The product was obtained in 88% yield (111.0 mg, 0.265 mmol, 98:2 dr) as an orange oil from the corresponding primary amine **1** (0.45 mmol) and allylic carbonate **2** (0.30 mmol); $[\alpha]_D^{25}$ = +18.8 (*c* = 0.10, MeOH); ^{1}H NMR (400 MHz, $CDCl_3$) δ 7.27 (d, *J* = 8.0 Hz, 2H), 6.82 (d, *J* = 7.6 Hz, 2H), 6.37 (d, *J* = 16.0 Hz, 1H), 6.08-5.97 (m, 1H), 4.32-4.19 (m, 1H), 4.10-3.98 (m, 1H), 3.78 (s, 3H), 3.07-2.96 (m, 1H), 2.47-2.36 (m, 1H), 2.36-2.23 (m, 2H), 2.20-2.09 (m, 1H), 1.84 (brs, 2H), 1.57-1.49 (m, 3H), 1.45 (s, 3H), 1.44-1.39 (m, 10H), 1.36 (s, 3H); ^{13}C NMR (100 MHz, $CDCl_3$) δ 170.4, 159.0, 132.2, 130.3, 127.2, 124.8, 114.0, 98.7, 80.7, 68.2, 66.3, 55.4, 49.2, 43.8, 42.7, 42.1, 36.8, 30.2, 28.2 19.9; HRMS (ESI): *m/z* Calcd. For $C_{24}H_{38}NO_5$ $(M + H)^+$: 420.2744; Found: 420.2751.

Compound 3aw (Fig. 3)

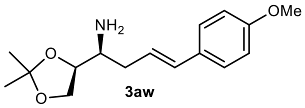

3aw

The product was obtained in 85% yield (70.6 mg, 0.255 mmol, 97:3 dr) as a white solid from the corresponding primary amine **1** (0.45 mmol) and allylic carbonate **2** (0.30 mmol); M.p. 52-53 °C; $[\alpha]_D^{25}$ = -15.6 (*c* = 0.10, MeOH); ^{1}H NMR (400 MHz, $CDCl_3$) δ 7.29 (d, *J* = 8.8 Hz, 2H), 6.84 (d, *J* =

8.8 Hz, 2H), 6.41 (d, J = 15.6 Hz, 1H), 6.11-5.98 (m, 1H), 4.07-3.97 (m, 2H), 3.94-3.86 (m, 1H), 3.80 (s, 3H), 3.09-2.99 (m, 1H), 2.51-2.40 (m, 1H), 2.22-2.11 (m, 1H), 1.75 (brs, 2H), 1.44 (s, 3H), 1.36 (s, 3H); ^{13}C NMR (100 MHz, $CDCl_3$) δ 159.1, 132.5, 130.2, 127.3, 124.3, 114.1, 109.1, 79.3, 65.9, 55.4, 52.8, 37.8, 26.7, 25.4; HRMS (ESI): *m/z* Calcd. For $C_{16}H_{24}NO_3$ $(M + H)^+$: 278.1751; Found: 278.1753.

Compound 3ax (Fig. 3)

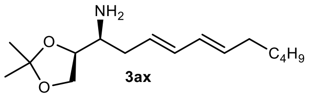

3ax

The product was obtained in 74% yield (59.5 mg, 0.223 mmol, 96:4 dr) as a brown oil from the corresponding primary amine **1** (0.45 mmol) and allylic carbonate **2** (0.30 mmol); $[\alpha]_D^{25}$ = +3.8 (c = 0.10, MeOH); ^{1}H NMR (400 MHz, $CDCl_3$) δ 6.14-5.94 (m, 2H), 5.67-5.57 (m, 1H), 5.57-5.45 (m, 1H), 4.04-3.92 (m, 2H), 3.90-3.80 (m, 1H), 3.04-2.90 (m, 1H), 2.39-2.28 (m, 1H), 2.10-1.95 (m, 3H), 1.61 (brs, 2H), 1.42 (s, 3H), 1.40-1.37 (m, 1H), 1.35 (s, 3H), 1.33-1.22 (m, 5H), 0.88 (t, J = 6.8 Hz, 3H); ^{13}C NMR (100 MHz, $CDCl_3$) δ 134.0, 133.8, 129.9, 127.4, 109.0, 79.2, 65.9, 52.8, 37.3, 32.7, 31.5, 29.1, 26.7, 25.4, 22.6, 14.1; HRMS (ESI): *m/z* Calcd. For $C_{16}H_{30}NO_2$ $(M + H)^+$: 268.2271; Found: 268.2276.

Compound 3ay (Fig. 3)

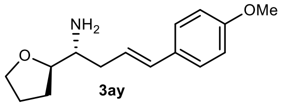

3ay

The product was obtained in 73% yield (54.4 mg, 0.220 mmol, 98:2 dr) as a pale yellow oil from the corresponding primary amine **1** (0.45 mmol) and allylic carbonate **2** (0.30 mmol); $[\alpha]_D^{25}$ = +22.1 (c = 0.10, MeOH); ^{1}H NMR (400 MHz, $CDCl_3$) δ 7.27 (d, J = 8.8 Hz, 2H), 6.82 (d, J =8.0 Hz, 2H), 6.40 (d, J = 15.6 Hz, 1H), 6.11-5.99 (m, 1H), 3.86-3.79 (m, 1H), 3.78 (s, 3H), 3.77-3.71 (m, 1H), 3.67 (q, J = 7.2 Hz, 1H), 2.81-2.72 (m, 1H), 2.45-2.32 (m, 1H), 2.20-2.08 (m, 1H), 2.02-1.82 (m, 3H), 1.73 (brs, 2H), 1.67-1.56 (m, 1H); ^{13}C NMR (100 MHz, $CDCl_3$) δ 158.9, 132.1, 130.3, 127.3, 124.8, 114.0, 83.2, 68.1, 55.40, 55.36, 38.3, 28.6, 26.3; HRMS (ESI): *m/z* Calcd. For $C_{15}H_{22}NO_2$ $(M + H)^+$: 248.1645; Found: 248.1646.

Compound 3az (Fig. 3)

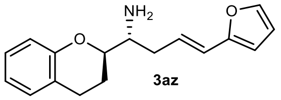

3az

The product was obtained in 79% yield (63.8 mg, 0.237 mmol, 99:1 dr) as a brown oil from the corresponding primary amine **1** (0.45 mmol) and allylic carbonate **2** (0.30 mmol); $[\alpha]_D^{25}$ = -65.9 (c = 0.10, MeOH); ^{1}H NMR (400 MHz, $CDCl_3$) δ 7.34-7.30 (m, 1H), 7.13-7.01 (m, 2H), 6.89-6.78 (m, 2H), 6.38-6.29 (m, 2H), 6.23-6.12 (m, 2H), 3.95-3.85 (m, 1H), 3.06-2.97 (m, 1H), 2.91-2.83 (m, 1H), 2.83-2.74 (m, 1H), 2.64-2.51 (m, 1H), 2.40-2.27 (m, 1H), 2.05-1.96 (m, 1H), 1.96-1.63 (m, 3H); ^{13}C NMR (100 MHz, $CDCl_3$) δ 155.0, 152.9, 141.7, 129.6, 127.3, 125.9, 122.2, 121.5, 120.3, 116.9, 111.3, 106.9, 78.7, 54.7, 37.6, 25.0, 24.4; HRMS (ESI): *m/z* Calcd. For $C_{17}H_{20}NO_2$ $(M + H)^+$: 270.1489; Found: 270.1491.

Compound 3ba (Fig. 3)

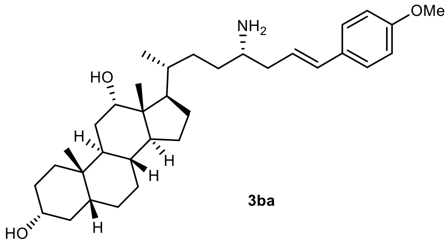

3ba

The product was obtained in 89% yield (139.0 mg, 0.266 mmol, 98:2 dr) as a pale yellow solid from the corresponding primary amine **1** (0.45 mmol) and allylic carbonate **2** (0.30 mmol); M.p. 75-77 °C; $[\alpha]_D^{25}$ = +46.3 (c = 0.10, MeOH); ^{1}H NMR (400 MHz, $CDCl_3$) δ 7.29 (d, J = 8.0 Hz, 2H), 6.84 (d, J = 8.0 Hz, 2H), 6.39 (d, J = 16.0 Hz, 1H), 6.10-5.99 (m, 1H), 4.01-3.95 (m, 1H), 3.80 (s, 3H), 3.65-3.53 (m, 1H), 2.83-2.73 (s, 1H), 2.42-2.33 (m, 1H), 2.14-2.02 (m, 1H), 1.92-1.00 (m, 30H), 0.98 (d, J = 6.4 Hz, 3H), 0.90 (s, 3H), 0.67 (s, 3H); ^{13}C NMR (100 MHz, $CDCl_3$) δ 158.9, 132.1, 130.4, 127.3, 125.3, 114.1, 73.3, 71.9, 55.4, 51.8, 48.4, 47.5, 46.6, 42.2, 41.5, 36.6, 36.1, 35.8, 35.3, 34.2, 33.8, 32.5, 30.6, 28.7, 27.7, 27.3, 26.3, 23.8, 23.3, 18.0, 12.9; HRMS (ESI): *m/z* Calcd. For $C_{34}H_{54}NO_3$ $(M + H)^+$: 524.4098; Found: 524.4101.

Compound 3a′ (Table. S1)

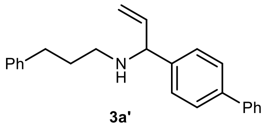

3a'

Yellow oil; ^{1}H NMR (400 MHz, $CDCl_3$) δ 7.62-7.54 (m, 4H), 7.47-7.38 (m, 4H), 7.37-7.30 (m, 1H), 7.30-7.23 (m, 2H), 7.22-7.15 (m, 3H), 6.02-5.89 (m, 1H), 5.23 (dt, *J* = 17.2, 1.2 Hz, 1H), 5.12 (dt, *J* = 10.0, 1.6 Hz, 1H), 4.22 (d, *J* = 7.2 Hz, 1H), 2.73-2.63 (m, 3H), 2.63-2.54 (m, 1H), 1.90-1.79 (m, 2H); ^{13}C NMR (100 MHz, $CDCl_3$) δ 142.34, 142.25, 141.2, 141.1, 140.2, 128.9, 128.5, 128.4, 127.8, 127.4, 127.3, 127.2, 125.9, 115.1, 66.0, 47.3, 33.8, 32.0; HRMS (ESI): *m/z* Calcd. For $C_{24}H_{26}N$ $(M + H)^+$: 328.2060; Found: 328.2060.

1.6 Procedure for Synthesis of 3w in Gram-scale (Fig. 4a)

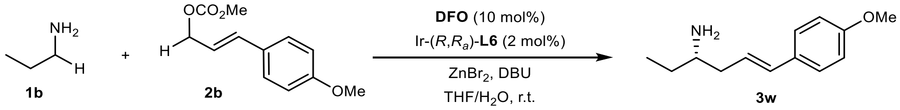

In a glove box, to a dry 5-mL vial equipped with a magnetic stirrer bar were added Ir(COD)$_2$BF$_4$ (0.099 g, 0.20 mmol), (*R*,*R*a)-**L6** (0.148 g, 0.22 mmol), THF (1.0 mL) and propylamine (**1b)** (0.118 g, 2.0 mmol). The vial was sealed and taken out of the glove box. The mixture was stirred at 50 ºC for 30 min and then cooled down to room temperature, which was used as the solution of the Ir catalyst. In the glove box, to a 50-mL round-bottom flask equipped with a magnetic stirrer bar were added 1,8-diazafluoran-9-one (**4**, DFO) (0.182 g, 1.0 mmol), THF (1.0 mL), propylamine (**1b**) (0.236 g, 4.0 mmol) and a solution of $ZnBr_2$ (1.62 g, 7.2 mmol) in THF (3.0 mL). After the mixture was stirred at room temperature for 30 min, a solution of allylic carbonate **2b** (2.22 g, 10 mmol) in THF (3.5 mL), 1,8-diazabicyclo[5.4.0]undec-7-ene (DBU) (1.68 g, 11 mmol), the pre-prepared solution of the Ir catalyst, THF (0.5 mL) and water (1.0 mL) were added. The reaction mixture was stirred at room temperature for 15 hours. A solution of $ZnBr_2$ (1.62 g, 7.2 mmol) and propylamine (**1b**) (0.355 g, 6.0 mmol) in THF (3.0 mL), prepared by stirring at 0 °C for 30 min, was added. After the resulting mixture was stirred at room temperature for 10 hours, a solution of $ZnBr_2$ (0.81 g, 3.6 mmol) and propylamine (**1b**) (0.177 g, 3.0 mmol) in THF (2.0 mL), prepared by stirring at 0 °C for 30 min, was added. The reaction mixture continued to stir at room temperature for 3 days. $NH_2OH\cdot HCl$ (0.348 g, 5.0 mmol) dissolved in 5.0 mL of 1 M HCl aqueous solution was added to quench the reaction. After stirring at room temperature for 1 hour, ammonium hydroxide (20 mL, 25-28 wt% in water) was added. The mixture was diluted with ethyl acetate (50 mL) and water (30 mL) and then separated. The aqueous layer was extracted with DCM (50 mL × 3). The combined organic layers were dried over Na_2SO_4, filtered, concentrated under reduced pressure and purified via column chromatography on silica gel (dichloromethane : methanol : triethylamine = 100:3:1, the silica gel column was eluted with 1% v/v solution of Et_3N in petroleum ether before sample loading) to afford compound **3w** (1.675 g, 82% yield, 93% ee) as a brown solid.

1.7 Determination of the Absolute Configuration of Alkylation Products 3a by X-ray Analysis

The absolute configuration of compound **3a** was determined as (*S*) by X-ray analysis of its *N*-Boc derivative (*S*)-NHBoc-**3a**. Compound (*S*)-NHBoc-**3a** was prepared by the following procedure. The absolute configurations of other alkylation products **3b-ba** were proposed by analogy.

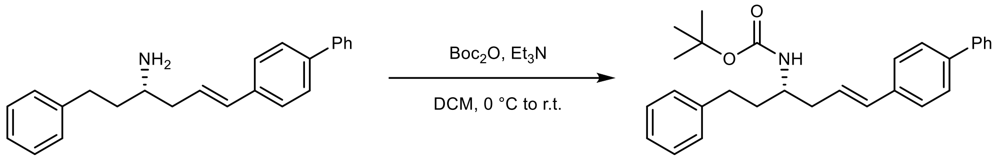

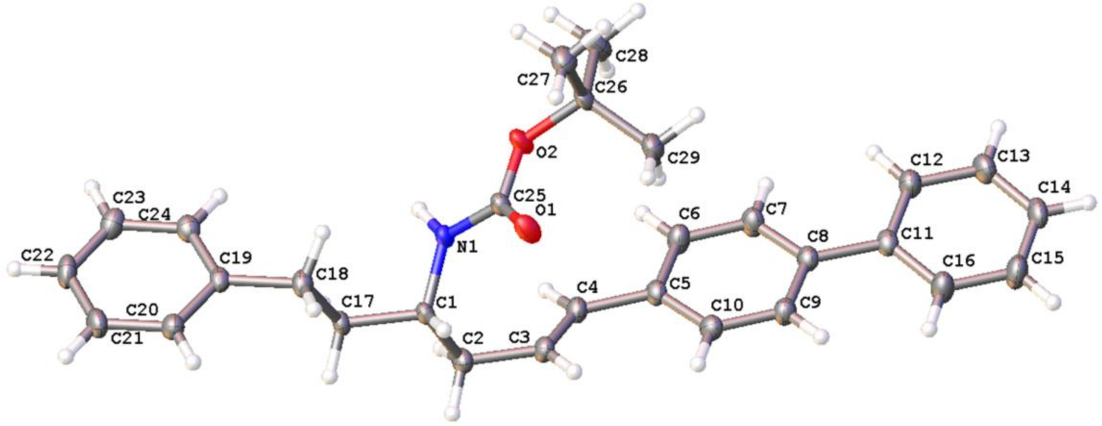

Supplementary Fig. 2. Synthesis of (*S*)-NHBoc-**3a** for X-ray analysis to determine the absolute configuration of compound **3a** (CCDC 2235306)

Synthesis of compound (*S*)-NHBoc-3a (Supplementary Fig. 2)

To a dry 5-mL vial equipped with a magnetic stirred bar were added compound (*S*)-**3a** (0.080 g, 0.245 mmol), DCM (0.8 mL) and triethylamine (0.074 g, 0.734 mmol). After cooling to 0 °C, *tert*-butyl dicarbonate (Boc_2O) (0.133 g. 0.612 mmol) was added and the reaction mixture was allowed to warm up to room temperature. Upon being stirred for 2 hours, the mixture was diluted with water (5 mL) and extracted with DCM (5 mL × 3). The combined organic layers were dried over anhydrous Na_2SO_4, filtered, concentrated, and purified via column chromatography on silica gel (petroleum ether : ethyl acetate = 5:1) to afford compound (*S*)-NHBoc-**3a** (0.085 g, 81% yield) as a pale yellow solid.

(*S*)-NHBoc-**3a**: Pale yellow solid; M.p. 136-137 °C; $[\alpha]_D^{25}$ = -4.3 (*c* = 0.10, MeOH); ^{1}H NMR (400 MHz, $CDCl_3$) δ 7.61 (d, *J* = 7.2 Hz, 2H), 7.55 (d, *J* = 8.4 Hz, 2H), 7.48-7.39 (m, 4H), 7.35 (t, *J* = 7.2 Hz, 1H), 7.30 (t, *J* = 7.6 Hz, 2H), 7.24-7.16 (m, 3H), 6.47 (d, *J* = 16.0 Hz, 1H), 6.28-6.17 (m, 1H), 4.45 (d, *J* = 8.4 Hz, 0.87H for major amide isomer), 4.25 (brs, 0.13H for minor amide isomer), 3.93-3.72 (m, 0.87H for major amide isomer), 3.72-3.50 (m, 0.13H for minor amide isomer), 2.82-2.62 (m, 2H), 2.54-2.34 (m, 2H), 1.95-1.82 (m, 1H), 1.80-1.67 (m, 1H), 1.45 (s, 9H); ^{13}C NMR (100 MHz, $CDCl_3$) δ 155.7, 142.0, 140.9, 140.1, 136.5, 132.5, 128.9, 128.6, 128.5, 127.4, 127.3, 127.0, 126.7, 126.3, 126.0, 79.3, 50.5, 39.2, 36.9, 32.7, 28.6; HRMS (ESI): *m/z* Calcd. For $C_{29}H_{33}NO_2Na$ $(M + Na)^+$: 450.2404; Found: 450.2396.

The single crystal of compound (*S*)-NHBoc-**3a** was obtained by recrystallization from a mixed solvent of *n*-hexane and toluene.

Data collection and structure solution for (*S*)-NHBoc-3a (Supplementary Fig. 2, CCDC 2235306)

A colorless crystal with approximate dimensions 0.07 × 0.06 × 0.05 mm^3 was selected for the X-ray analysis. All measurements were performed on a Bruker D8 Venture diffractometer with GaKα (λ = 1.34139 Å) radiation. The crystal was kept at 193 K during data collection. Using *Olex2*[27], the crystal structure was solved with the *ShelXT*[28] structure solution program using Intrinsic Phasing and refined with the *ShelXL*[29] refinement package using Least Squares minimization. The absolute configuration was established on the basis of the absolute structure parameter.

Supplementary Table 2. Crystal data and structure refinement for (*S*)-NHBoc-**3a**

Identification code	mj21123_0m
Empirical formula	$C_{29}H_{33}NO_2$
Formula weight	427.56
Temperature/K	193
Crystal system	monoclinic
Space group	$P2_1$
a/Å	5.3294(2)
b/Å	8.9523(3)
c/Å	25.4780(10)
α/°	90
β/°	95.160(2)
γ/°	90
Volume/Å^3	1210.64(8)
Z	2
ρ_{calc}g/cm^3	1.173
μ/mm^{-1}	0.363
F(000)	460.0
Crystal size/mm^3	0.07 × 0.06 × 0.05
Radiation	GaKα (λ = 1.34139)
2Θ range for data collection/°	9.096 to 110.108
Index ranges	$-6 \leq h \leq 6$, $-10 \leq k \leq 10$, $-31 \leq l \leq 30$
Reflections collected	12900
Independent reflections	4498 [R_{int} = 0.0347, R_{sigma} = 0.0364]
Data/restraints/parameters	4498/1/292
Goodness-of-fit on F^2	1.060
Final R indexes [I>=2σ (I)]	R_1 = 0.0394, wR_2 = 0.1041
Final R indexes [all data]	R_1 = 0.0438, wR_2 = 0.1081
Largest diff. peak/hole / e Å^{-3}	0.18/-0.21
Flack parameter	0.05(14)

1.8 Procedure for the Synthesis of Compound 10 (Fig. 4a)

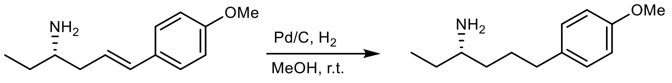

To a dry 5-mL vial equipped with a magnetic stirrer bar were added compound **3w** (0.062 g, 0.30 mmol), Pd/C (10% on carbon, wetted with ca. 55% water, 0.027 g). The sealed vial was evacuated and refilled with H_2 three times, followed by addition of methanol (0.9 mL) via a syringe. The reaction mixture was stirred at room temperature under H_2 (balloon) atmosphere for 8 hours. The reaction mixture was filtered through a pad of Celite and washed with DCM. The combined filtrates were concentrated under reduced pressure to give compound **10** (0.0565 g, 91% yield, 93% ee) as colorless oil. The enantiomeric excess (ee) of product **10** was determined by chiral HPLC analysis after being converted to its *N*-Boc derivative.

10: Colorless oil; $[\alpha]_D^{25}$ = +9.6 (c = 0.10, MeOH); ^{1}H NMR (400 MHz, $CDCl_3$) δ 7.09 (d, J = 8.8 Hz, 2H), 6.81 (d, J = 8.8 Hz, 2H), 3.77 (s, 3H), 2.69-2.60 (m, 1H), 2.60-2.48 (m, 2H), 1.73-1.51 (m, 4H), 1.50-1.38 (m, 2H), 1.35-1.22 (m, 2H), 0.90 (t, J = 7.6 Hz, 3H); ^{13}C NMR (100 MHz, $CDCl_3$) δ 157.8, 134.7, 129.3, 113.8, 55.3, 52.7, 37.0, 35.2, 30.5, 28.4, 10.4; HRMS (ESI): *m/z* Calcd. For $C_{13}H_{22}NO$ $(M + H)^+$: 208.1696; Found: 208.1697.

1.9 Procedure for Synthesis of Compound 11 (Fig. 4a)

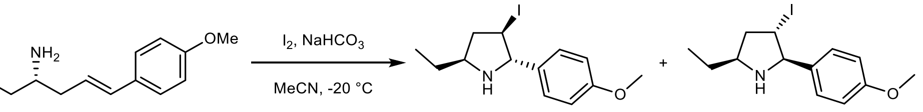

3w (2*S*,3*R*,5*S*)-**11** (2*R*,3*S*,5*S*)-**11** (minor)

To a dry 10-mL Schlenk tube equipped with a magnetic stirrer bar were added compound **3w** (0.062 g, 0.30 mmol), $NaHCO_3$ (0.025 g, 0.30 mmol) and dry acetonitrile (1.5 mL), followed by the addition of iodine (0.381 g, 1.5 mmol) at -20 °C. The mixture was stirred at -20 °C for 10 hours, then quenched with saturated $Na_2S_2O_3$ aqueous solution (7 mL) and diluted with DCM (7 mL) at -20 °C. After stirring for 5 min, to the reaction mixture was added water (7 mL). The aqueous layer was extracted with DCM (10 mL × 3). The combined organic layers were dried over Na_2SO_4, filtered, concentrated via rotary evaporation, and submitted to ^1H NMR analysis to determine the dr value (3.7:1), followed by purification via column chromatography on silica gel (petroleum ether : ethyl acetate = 5:1) to give compound (2*S*,3*R*,5*S*)-**11** (R_f = 0.3, petroleum ether : ethyl acetate = 5:1, 0.0642 g, 65% yield, 94% ee) and (2*R*,3*S*,5*S*)-**11** (R_f = 0.5, petroleum ether : ethyl acetate = 5:1, 0.0182 g, 18% yield, 93% ee). The total yield of the two diastereomers was 83%. The enantiomeric excesses (ee) of product **11** were determined by chiral HPLC analysis without further derivatization.

(2*S*,3*R*,5*S*)-**11**: Yellow oil; $[\alpha]_D^{25}$ = -39.8 (*c* = 0.10, $CHCl_3$); ^1H NMR (400 MHz, $CDCl_3$) δ 7.31 (d, *J* = 7.2 Hz, 2H), 6.87 (d, *J* = 7.2 Hz, 2H), 4.33 (d, *J* = 8.8 Hz, 1H), 4.03-3.93 (m, 1H), 3.79 (s, 3H), 3.36-3.27 (m, 1H), 2.76-2.67 (m, 1H), 2.10 (brs, 1H), 2.01-1.95 (m 1H), 1.67-1.46 (m, 2H), 0.94 (t, *J* = 7.2 Hz, 3H); ^{13}C NMR (100 MHz, $CDCl_3$) δ 159.4, 132.7, 128.1, 114.1, 71.5, 60.1, 55.4, 45.5, 30.5, 29.9, 11.3; HRMS (ESI): *m/z* Calcd. For $C_{13}H_{19}INO$ $(M + H)^+$: 332.0506; Found: 332.0508.

(2*R*,3*S*,5*S*)-**11**: Yellow oil; $[\alpha]_D^{25}$ = +45.5 (*c* = 0.20, $CHCl_3$); ^1H NMR (400 MHz, $CDCl_3$) δ 7.34 (d, *J* = 8.4 Hz, 2H), 6.87 (d, *J* = 8.4 Hz, 2H), 4.47 (d, *J* = 7.2 Hz, 1H), 4.05-3.96 (m, 1H), 3.80 (s, 3H), 3.43-3.34 (m, 1H), 2.45-2.35 (m, 1H), 2.21-2.06 (m, 2H), 1.69-1.48 (m, 2H), 0.97 (t, *J* = 7.2 Hz, 3H); ^{13}C NMR (100 MHz, $CDCl_3$) δ 159.4, 132.9, 128.2, 114.0, 73.2, 59.8, 55.4, 44.1, 30.3, 29.2, 11.4; HRMS (ESI): *m/z* Calcd. For $C_{13}H_{19}INO$ $(M + H)^+$: 332.0506; Found: 332.0507.

1.10 Determination of the Absolute Configuration of Compound (2*S*,3*R*,5*S*)-11 by X-ray Analysis

The absolute configuration of the major diastereomer of compound **11** was determined as (2*S*,3*R*,5*S*) by X-ray analysis of its *N*-Ts derivative (2*S*,3*R*,5*S*)-NTs-**11**. Compound (2*S*,3*R*,5*S*)-NTs-**11** was prepared by the following procedure.

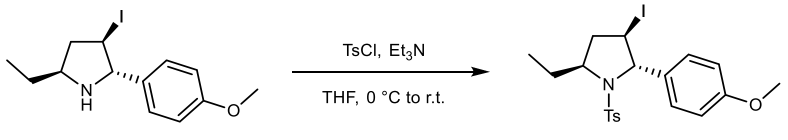

(2*S*,3*R*,5*S*)-**11** (2*S*,3*R*,5*S*)-NTs-**11**

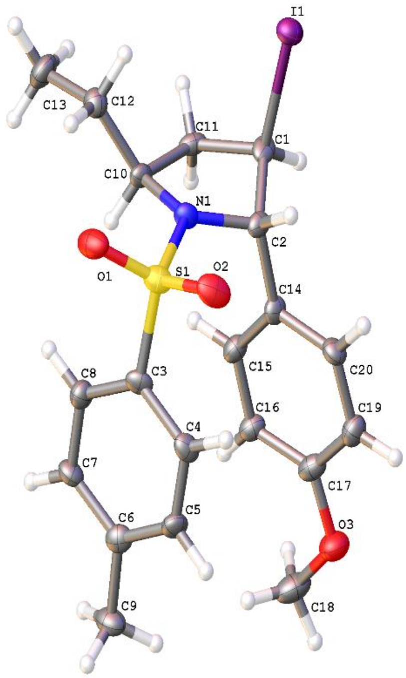

Supplementary Fig. 3. Synthesis of (2*S*,3*R*,5*S*)-NTs-**11** for X-ray analysis to determine the absolute configuration of compound **11** (CCDC 2235307)

Synthesis of compound (2*S*,3*R*,5*S*)-NTs-11 (Supplementary Fig. 3)

To a stirred solution of compound (2*S*,3*R*,5*S*)-**11** (0.020 g, 0.060 mmol) in THF (0.06 mL) was added triethylamine (9 mg, 0.090 mmol). After cooling to 0 °C, tosyl chloride (0.014 g, 0.072 mmol) dissolved in THF (0.06 mL) was added dropwise and the reaction mixture was allowed to warm up to room temperature. Upon being stirred for 15.5 hours, the reaction was diluted with water (5 mL) and extracted with DCM (5 mL × 3). The combined organic layers were dried over anhydrous Na_2SO_4,

filtered, concentrated, and purified by column chromatography on silica gel (petroleum ether : ethyl acetate = 10:1) to afford compound (2*S*,3*R*,5*S*)-NTs-**11** (0.021 g, 72% yield) as a white solid.

(2*S*,3*R*,5*S*)-NTs-**11**: White solid; M.p. 98-99 °C; $[\alpha]_D^{25}$ = -79.8 (*c* = 0.10, MeOH); ^{1}H NMR (400 MHz, $CDCl_3$) δ 7.53 (d, *J* = 8.4 Hz, 2H), 7.15 (d, *J* = 8.0 Hz, 2H), 7.07 (d, *J* = 8.8 Hz, 2H), 6.73 (d, *J* = 8.8 Hz, 2H), 5.21 (d, *J* = 3.2 Hz, 1H), 4.25-4.13 (m, 2H), 3.78 (s, 3H), 2.81-2.69 (m, 1H), 2.38 (s, 3H), 2.34-2.21 (m, 2H), 2.01-1.87 (m, 1H), 0.91 (t, *J* = 7.2 Hz, 3H); ^{13}C NMR (100 MHz, $CDCl_3$) δ 159.4, 142.8, 139.4, 131.8, 129.2, 128.0, 127.2, 114.0, 76.0, 63.7, 55.4, 38.7, 27.1, 25.4, 21.6, 11.1; HRMS (ESI): *m/z* Calcd. For $C_{20}H_{25}INO_3S$ $(M + H)^+$: 486.0594; Found: 486.0598.

The single crystal of compound (2*S*,3*R*,5*S*)-NTs-**11** was obtained by recrystallization from a mixed solvent of tetrachloromethane and *n*-pentane.

Data collection and structure solution for (2*S*,3*R*,5*S*)-NTs-11 (Supplementary Fig. 3, CCDC 2235307)

A colorless crystal with approximate dimensions 0.07 × 0.07 × 0.05 mm^3 was selected for the X-ray analysis. All measurements were performed on a Bruker D8 Venture diffractometer with GaKα (λ = 1.34139 Å) radiation. The crystal was kept at 213 K during data collection. Using *Olex*[27], the crystal structure was solved with the *ShelXT*[28] structure solution program using Intrinsic Phasing and refined with the *ShelXL*[29] refinement package using Least Squares minimization. The absolute configuration was established on the basis of the absolute structure parameter.

Supplementary Table 3. Crystal data and structure refinement for (2*S*,3*R*,5*S*)-NTs-**11**

Identification code	mj22414_0m
Empirical formula	$C_{20}H_{24}INO_3S$
Formula weight	485.36
Temperature/K	213.00
Crystal system	orthorhombic
Space group	$P2_12_12_1$
a/Å	6.0922(3)
b/Å	21.6718(8)
c/Å	30.8864(12)
α/°	90
β/°	90
γ/°	90
Volume/Å^3	4077.9(3)
Z	8
ρ_{calc}g/cm^3	1.581
μ/mm^{-1}	9.200
F(000)	1952.0
Crystal size/mm^3	0.07 × 0.07 × 0.05
Radiation	GaKα (λ = 1.34139)
2Θ range for data collection/°	4.978 to 109.962
Index ranges	-7 ≤ h ≤ 6, -26 ≤ k ≤ 26, -37 ≤ l ≤ 37
Reflections collected	41892
Independent reflections	7762 [R_{int} = 0.0991, R_{sigma} = 0.0805]
Data/restraints/parameters	7762/0/475
Goodness-of-fit on F^2	1.025
Final R indexes [I>=2σ (I)]	R_1 = 0.0527, wR_2 = 0.0977
Final R indexes [all data]	R_1 = 0.0924, wR_2 = 0.1114
Largest diff. peak/hole / e Å^{-3}	0.60/-0.72
Flack parameter	0.079(8)

1.11 Procedure for the Synthesis of Compound 12 (Fig. 4a)

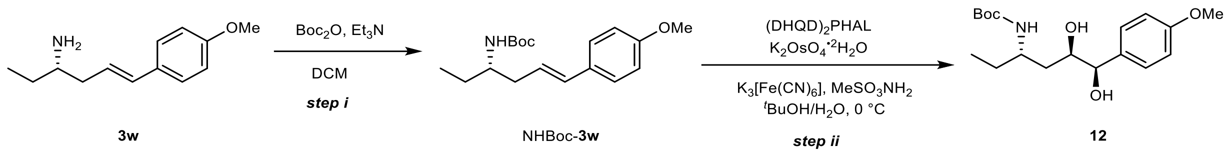

***Step i.* Synthesis of compound NHBoc-3w**

To a dry 10-mL round-bottom flask equipped with a magnetic stirred bar were added **3w** (0.200 g, 0.98 mmol), DCM (3.5 mL) and triethylamine (Et_3N) (0.296 g, 2.93 mmol). After cooling to 0 °C, *tert*-butyl dicarbonate (Boc_2O) (0.532 g, 2.44 mmol) was added and the mixture was allowed to warm up to room temperature. Upon stirring for 24 hours, the mixture was diluted with water (10 mL) and extracted with DCM (10 mL × 3). The combined organic layers were dried over anhydrous Na_2SO_4, filtered, concentrated, and purified via column chromatography on silica gel (petroleum ether : ethyl acetate = 10:1) to afford compound NHBoc-**3w** (0.245 g, 82% yield) as a white solid.

NHBoc-**3w**: White solid; M.p. 115-116 °C; $[\alpha]_D^{25}$ = -16.0 (c = 0.10, MeOH); ^{1}H NMR (400 MHz, $CDCl_3$) δ 7.27 (d, J = 8.8 Hz, 2H), 6.82 (d, J = 8.8 Hz, 2H), 6.36 (d, J = 15.6 Hz, 1H), 6.11-5.92 (m, 1H), 4.39 (d, J = 7.2 Hz, 0.89H for major amide isomer), 4.18 (brs, 0.11H for minor amide isomer), 3.79 (s, 3H), 3.70-3.54 (m, 0.89H for major amide isomer), 3.54-3.36 (m, 0.11H for minor amide isomer), 2.45-2.20 (m, 2H), 1.64-1.50 (m, 1H), 1.49-1.32 (m, 10H), 0.94 (t, J = 7.2 Hz, 3H); ^{13}C NMR (100 MHz, $CDCl_3$) δ 159.0, 155.8, 132.1, 130.4, 127.3, 124.1, 114.0, 79.1, 55.4, 52.0, 38.4, 28.5, 27.6, 10.5; HRMS (ESI): *m/z* Calcd. For $C_{18}H_{28}NO_3$ $(M + H)^+$: 306.2064; Found: 306.2065.

***Step ii.* Synthesis of compound 12**

To a 5-mL vial equipped with a magnetic stirrer bar were added $K_2OsO_4 \cdot 2H_2O$ (2.5 mg, 0.007 mmol), hydroquinidine 1,4-phthalazinediyl ether [$(DHQD)_2PHAL$] (12 mg, 0.015 mmol), $K_3[Fe(CN)_6]$ (0.324 g, 0.98 mmol), K_2CO_3 (0.126 g, 0.91 mmol), methanesulfonamide (0.031 g, 0.33 mmol), ^{t}BuOH (1 mL) and H_2O (1 mL). After stirring at room temperature for 30 min, compound NHBoc-**3w** (0.100 g, 0.33 mmol) was added at 0 °C. Upon being stirred at 0 °C for 20.5 hours, the reaction mixture was diluted with water (5 mL) and extracted with DCM (5 mL × 3). The combined organic layers were dried over anhydrous Na_2SO_4, filtered, concentrated, and submitted to ^{1}H NMR analysis to determine the dr value (> 20:1 dr), followed by purification via column chromatography on silica gel (dichloromethane : ethyl acetate = 5:1) to afford compound **12** (0.105 g, 94% yield) as a white solid.

The absolute configuration of **12** was tentatively assigned as (3*S*,5*R*,6*R*) according to the reported empirical model for asymmetric dihydroxylation of olefins[30].

12: White solid; M.p. 78-80 °C; $[\alpha]_D^{25}$ = -49.9 (*c* = 0.10, MeOH); ^1H NMR (400 MHz, $CDCl_3$) δ 7.24 (d, *J* = 8.8 Hz, 2H), 6.86 (d, *J* = 8.8 Hz, 2H), 4.78 (d, *J* = 3.2 Hz, 1H), 4.36 (d, *J* = 8.0 Hz, 2H), 3.79 (s, 3H), 3.70-3.54 (m, 2H), 3.53-3.47 (m, 1H), 1.51-1.37 (m, 11H), 1.35-1.22 (m, 1H), 1.01-0.91 (m, 1H), 0.87 (t, *J* = 7.6 Hz, 3H); ^{13}C NMR (100 MHz, $CDCl_3$) δ 159.4, 157.7, 132.8, 128.5, 113.9, 80.2, 77.6, 72.9, 55.3, 49.3, 39.5, 28.6, 28.4, 10.9; HRMS (ESI): *m/z* Calcd. For $C_{18}H_{29}NO_5Na$ (M + Na)$^+$: 362.1938; Found: 362.1946.

1.12 Procedure for the Synthesis of Compound 14 (Fig. 4a)

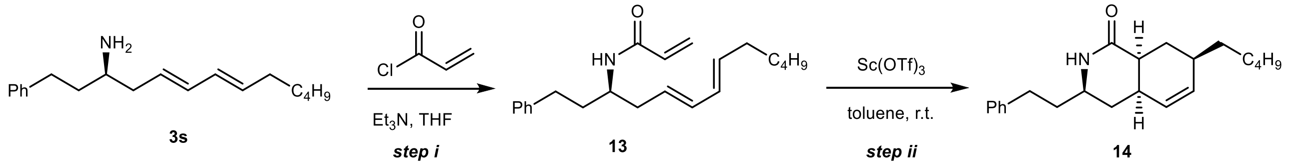

Step i. Synthesis of compound 13

To a stirred solution of compound **3s** (0.095 g, 0.35 mmol) in dry THF (1.0 mL) was added triethylamine (0.071 g, 0.70 mmol). After the mixture was stirred at 0 °C for 5 min, acryloyl chloride (0.048 g, 0.53 mmol) was added. Upon being stirred at 0 °C for 1 hour, the mixture was diluted with water (10 mL) and extracted with DCM (10 mL × 3). The combined organic layers were dried over anhydrous Na_2SO_4, filtered, concentrated, and purified via column chromatography on silica gel (petroleum ether : ethyl acetate = 6:1) to afford the compound **13** (0.0853 g, 75% yield) as a white solid.

13: White solid; M.p. 63-64 °C; $[\alpha]_D^{25}$ = +46.0 (c = 0.10, MeOH); ^{1}H NMR (400 MHz, $CDCl_3$) δ 7.31-7.23 (m, 2H), 7.21-7.13 (m, 3H), 6.26 (dd, J = 17.2, 1.6 Hz, 1H), 6.12-5.93 (m, 3H), 5.66-5.54 (m, 3H), 5.54-5.44 (m, 1H), 4.21-4.09 (m, 1H), 2.66 (t, J = 8.4 Hz, 2H), 2.31 (t, J = 7.2 Hz, 2H), 2.04 (q, J = 6.8 Hz, 2H), 1.92-1.81 (m, 1H), 1.80-1.68 (m, 1H), 1.44-1.20 (m, 6H), 0.89 (t, J = 6.8 Hz, 3H); ^{13}C NMR (100 MHz, $CDCl_3$) δ 165.2, 141.8, 134.2, 134.0, 131.2, 129.9, 128.5, 128.4, 126.4, 126.3, 126.0, 49.2, 38.0, 36.2, 32.7, 32.6, 31.5, 29.1, 22.6, 14.2; HRMS (ESI): *m/z* Calcd. For $C_{22}H_{32}NO$ (M + H)$^+$: 326.2478; Found: 326.2482.

Step ii. Synthesis of compound 14

To a 2-mL vial equipped with a magnetic stirred bar were added compound **13** (0.030 g, 0.092 mmol), $Sc(OTf)_3$ (9.1 mg, 0.018 mmol) in a glove box. To the sealed vial was added dry toluene (0.3 mL). Upon being stirred at room temperature for 25 hours, the mixture was diluted with water (5 mL) and extracted with ethyl acetate (5 mL × 3). The combined organic layers were dried over anhydrous Na_2SO_4, filtered, concentrated, and purified via column chromatography on silica gel (petroleum ether : ethyl acetate = 1:1) to afford compound **14** (0.0224 g, 75% yield) as a white solid. The diastereomeric ratio (dr value) was determined as > 20:1 by ^{1}H NMR analysis of the crude reaction mixture.

14: White solid; M.p. 125-127 °C; $[\alpha]_D^{25}$ = -22.1 (c = 0.10, MeOH); ^{1}H NMR (400 MHz, DMSO-d_6) δ 7.50 (s, 1H), 7.27 (t, J = 7.6 Hz, 2H), 7.21 (d, J = 6.8 Hz, 2H), 7.16 (, J = 7.2 Hz, 1H), 5.65-5.53

(m, 2H), 3.35-3.23 (m, 1H), 2.70-2.53 (m, 2H), 2.41-2.31 (m, 1H), 2.31-2.22 (m, 1H), 2.16-2.04 (m, 1H), 2.02-1.92 (m, 1H), 1.89-1.81 (m, 1H), 1.81-1.71 (m, 1H), 1.66-1.53 (m, 1H), 1.37-1.00 (m, 10H), 0.86 (t, J = 7.2 Hz, 3H); ^{13}C NMR (100 MHz, DMSO-d_6) δ 173.7, 141.8, 132.3, 129.2, 128.3, 125.7, 51.7, 39.8, 37.6, 35.7, 35.6, 32.6, 31.8, 31.5, 30.4, 29.5, 25.7, 22.1, 14.0; HRMS (ESI): *m/z* Calcd. For $C_{22}H_{32}NO$ $(M + H)^+$: 326.2478; Found: 326.2482.

The absolute configuration of compound **14** was tentatively determined by NMR analysis (Supplementary Figs. 4-6). The ^{1}H NMR signals of the bicyclic skeleton of compound **14** were assigned according to ^{1}H-^{1}H COSY and ^{1}H-^{13}C HSQC analysis (Supplementary Figs. 4 and 5). The ^{1}H-^{1}H NOESY spectroscopy was also collected as shown in Supplementary Fig. 6. There was a strong correlation between the H-1′ and the H-3′, indicating H-1′ and H-3′ are on the same side of the six-membered ring. There was obvious correlations between H-3′ and H-8′, which indicated H-3′ and H-8′ were located on same sides. There was a strong correlation between H-8′ and H-6′, suggesting that the H-8′ and H-6′ were on the same side of the ring. Since the chiral center at C-1′ was assigned as *R*, the absolute configuration of compound **14** could be determined as (1*R*,3*R*,6*R*,8*S*).

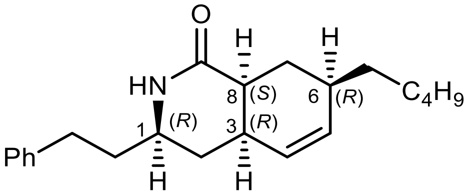

14

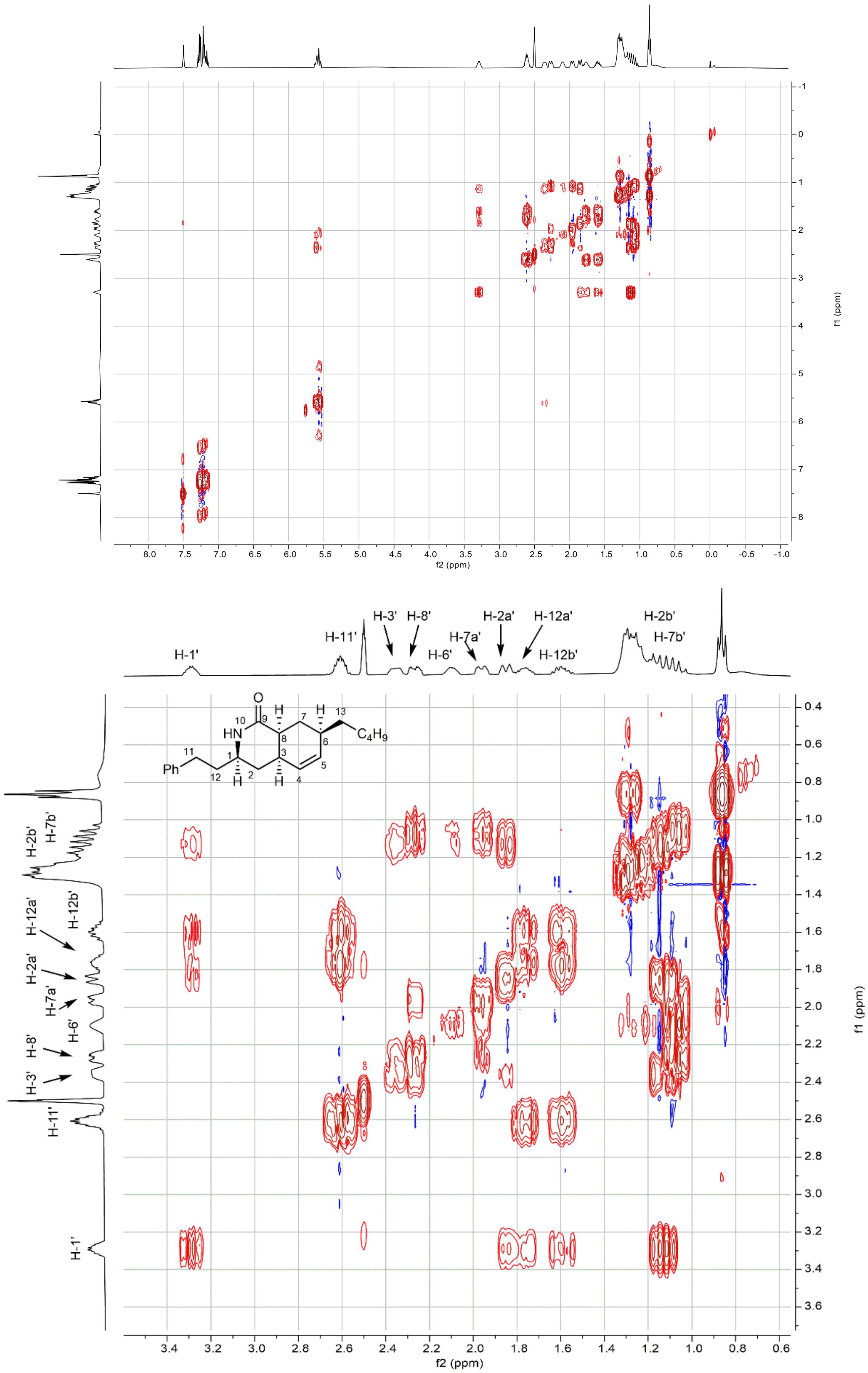

Supplementary Fig. 4. ^{1}H-^{1}H COSY of compound **14**

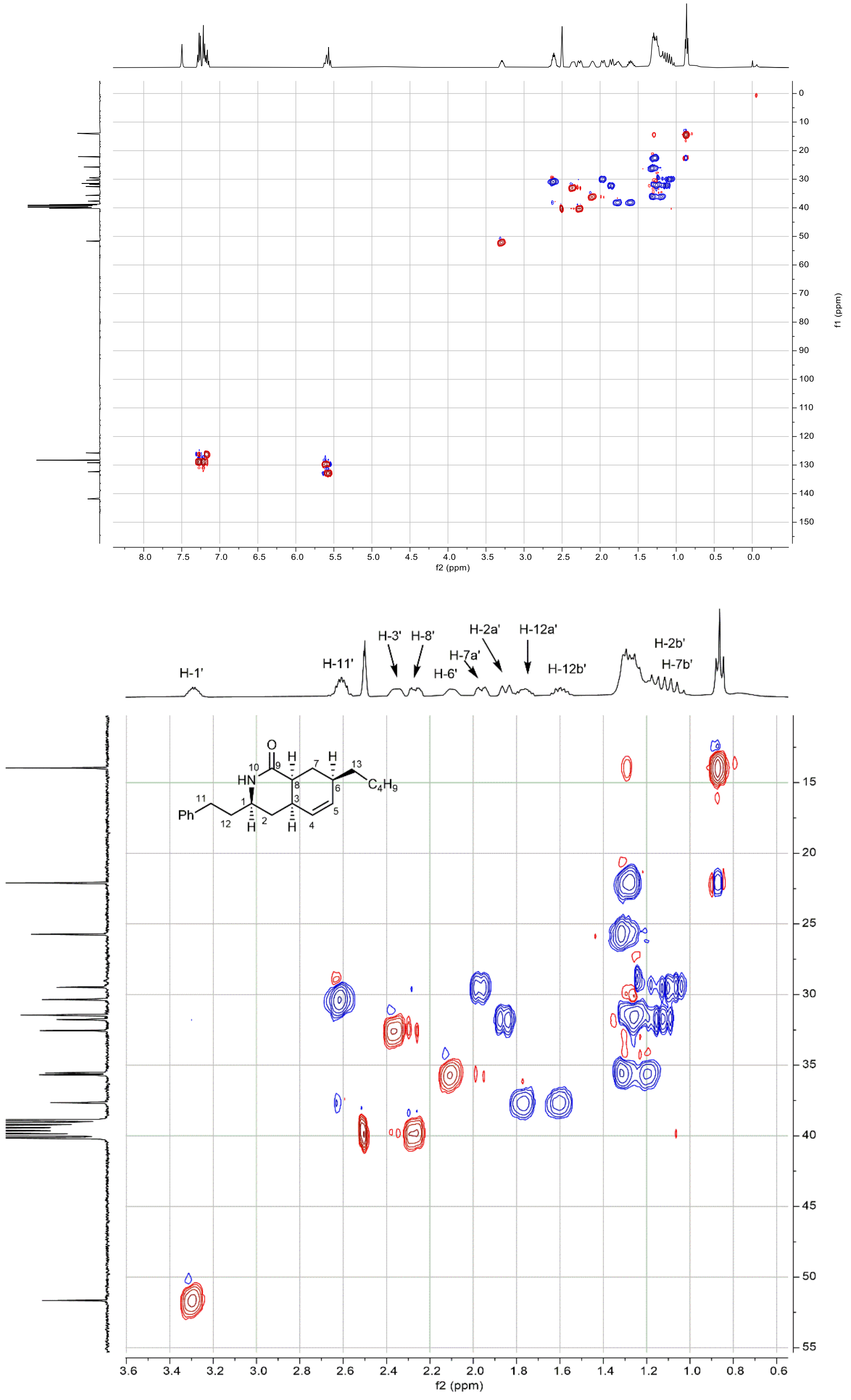

Supplementary Fig. 5. ^{1}H-^{13}C HSQC of compound **14**

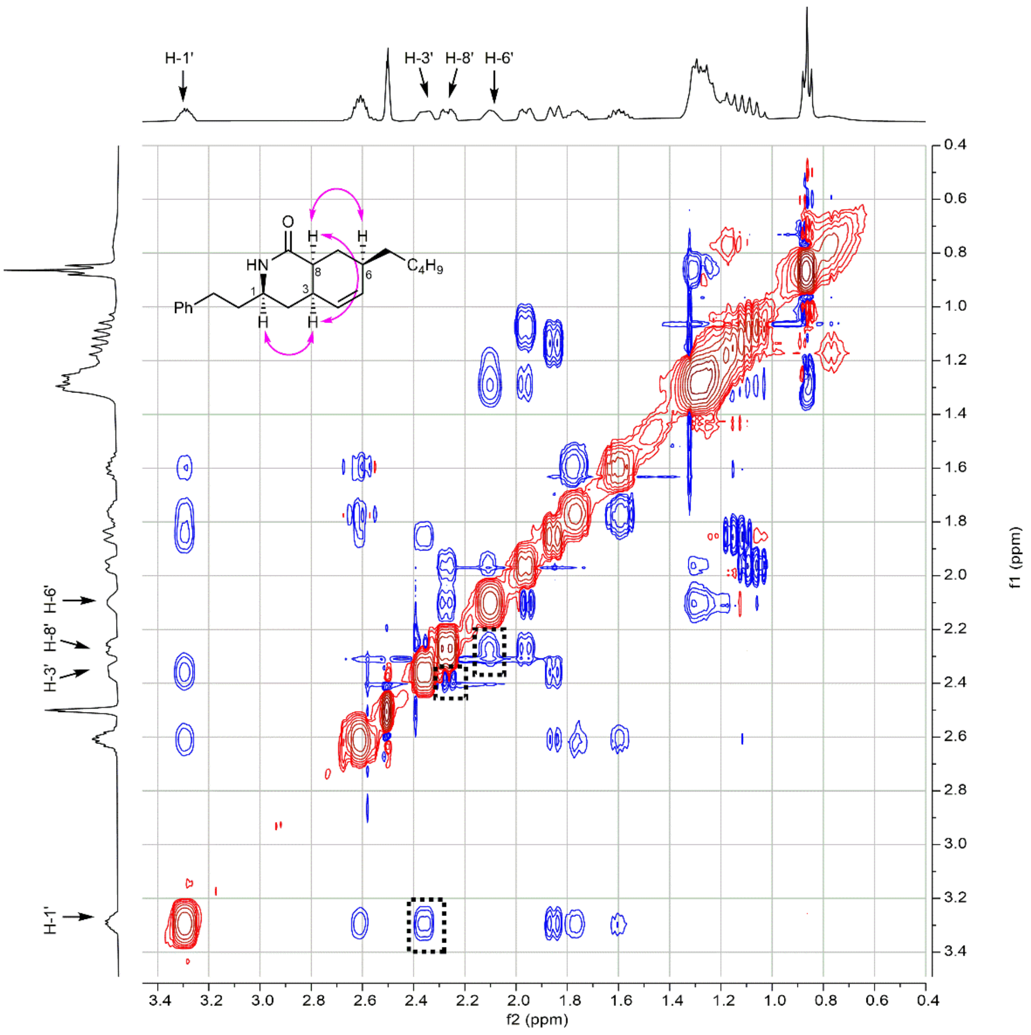

Supplementary Fig. 6. ^{1}H-^{1}H NOESY of compound **14**

1.13 Procedure for the Synthesis of Compound 17 (Fig. 4a)

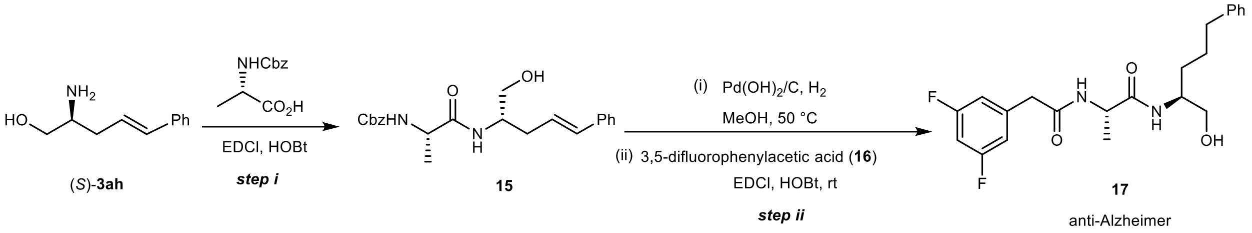

Step i. **Synthesis of compound 15**

To 5-mL vial equipped with a magnetic stirrer bar were added (*S*)-**3ah** (0.080 g, 0.45 mmol), *N*-carbobenzyloxy-L-alanine (0.088 g, 0.39 mmol), 1-ethyl-3-(3-dimethylaminopropyl)carbodiimide hydrochloride (EDCl) (0.112 g, 0.58 mmol), 1-hydroxybenzotriazole (HOBt) (0.079 g, 0.58 mmol), *N*-methyl morphofine (NMM) (0.079 g, 0.78 mmol) and DCM (1.5 mL). Upon being stirred at room temperature for 18 hours, the mixture was quenched with water (10 mL) and extracted with DCM (10 mL × 3). The combined organic layers were washed with water (40 mL), dried over anhydrous Na_2SO_4, filtered, concentrated, and purified via column chromatography on silica gel (ethyl acetate) to afford compound **15** (0.132 g, 89% yield, 98:2 dr) as a white solid. The diastereomeric ratio (dr value) of **15** was determined by HPLC analysis without further derivatization.

15: White solid; M.p. 120-122 °C; $[\alpha]_D^{25}$ = -32.8 (*c* = 0.10, MeOH); ^{1}H NMR (400 MHz, $CDCl_3$) δ 7.37-7.24 (m, 9H), 7.23-7.16 (m, 1H), 6.59 (d, *J* = 4.4 Hz, 1H), 6.43 (d, *J* = 15.6 Hz, 1H), 6.20-6.07 (m, 1H), 5.44 (d, *J* = 6.4 Hz, 1H), 5.07 (d, *J* = 12.0 Hz,1H), 4.96 (d. *J* = 12.4 Hz, 1H), 4.28-4.14 (m, 1H), 4.08-3.97 (m, 1H), 3.76-3.58 (m, 2H), 3.03 (brs, 1H), 2.56-2.32 (m, 2H), 1.34 (d, *J* = 7.2 Hz, 3H); ^{13}C NMR (100 MHz, $CDCl_3$) δ 172.9, 156.3, 137.2, 136.2, 133.2, 128.7, 128.4, 128.2, 127.5, 126.3, 125.7, 67.2, 64.4, 51.7, 51.0, 34.8, 18.4; HRMS (ESI): *m/z* Calcd. For $C_{22}H_{27}N_2O_4$ $(M + H)^+$: 383.1965 ; Found: 383.1971.

Step ii. **Synthesis of compound 17**

To a dry 5-mL vial equipped with a magnetic stirrer bar were added compound **15** (0.120 g, 0.31 mmol), $Pd(OH)_2/C$ (20% on carbon, wetted with ca. 50% water, 0.036 g). The sealed vial was evacuated and refilled with H_2 three times, followed by addition of methanol (1.2 mL) via a syringe. Upon stirring at 50 °C under H_2 (balloon) atmosphere for 4 hours, the reaction mixture was filtered through a pad of Celite and washed with DCM (30 mL). The combined filtrates were concentrated under reduced pressure to give the hydrogenated intermediate as a white solid that was used for the following reaction without further purification. The solid was dissolved with dry THF (1.0 mL) and

dry DMF (0.5 mL). To the solution were added 3,5-difluorophenylacetic acid (**16**) (0.047 g, 0.27 mmol), 1-ethyl-3-(3-dimethylaminopropyl)carbodiimide hydrochloride (EDCl) (0.078 g, 0.41 mmol), 1-hydroxybenzotriazole (HOBt) (0.055 g, 0.41 mmol), and *N*-methyl morpholine (NMM) (0.055 g, 0.54 mmol). Upon being stirred at room temperature for 24 hours, the reaction mixture was quenched with water (10 mL) and extracted with DCM (10 mL × 3). The combined organic layers were washed with water (30 mL × 3), dried over anhydrous Na_2SO_4, filtered, concentrated, and purified via column chromatography on silica gel (dichloromethane : methanol = 30:1) to afford the compound **17** (0.108 g, 99% yield) as a white solid.

17: White solid; M.p. 157-158 °C; $[\alpha]_D^{25}$ = -52.1 (*c* = 0.10, MeOH); ^{1}H NMR (400 MHz, CD_3OD) δ 7.21 (t, *J* = 7.6 Hz, 2H), 7.17-7.08 (m, 3H), 6.95-6.87 (m, 2H), 6.84-6.75 (m, 1H), 4.34 (q, *J* = 7.2 Hz, 1H), 3.94-3.84 (m, 1H), 3.55 (s, 2H), 3.46 (d, *J* = 5.6 Hz, 2H), 2.68-2.46 (m, 2H), 1.73-1.53 (m, 3H), 1.48-1.38 (m, 1H), 1.35 (d, *J* = 7.2 Hz, 3H); ^{13}C NMR (100 MHz, CD_3OD) δ 174.9, 172.3, 164.4 (dd, *J* = 245.3, 13.0 Hz), 143.5, 141.0 (t, *J* = 9.7 Hz), 129.5, 129.3, 126.7, 113.2 (dd, *J* = 18.5, 6.7 Hz) 103.0 (t, *J* = 25.6 Hz), 65.1, 52.4, 50.7, 42.7 (t, *J* = 1.8 Hz), 36.5, 31.6, 28.9, 18.2; ^{19}F NMR (376 MHz, CD_3OD) δ -112.1; HRMS (ESI): *m/z* Calcd. For $C_{22}H_{27}F_2N_2O_3$ $(M + H)^+$: 405.1984; Found: 405.1980.

2. Supplementary Discussion

2.1 Investigation on the Aza-Cope Rearrangement of Intermediate 21 (Fig. 4d)

(i) Synthesis of compound 25

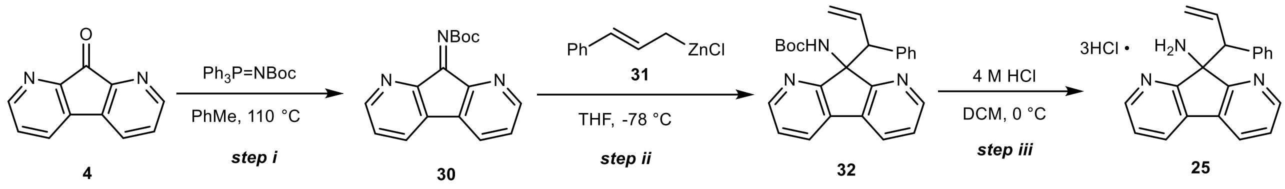

Step i. **Synthesis of compound 30**

To a 10-mL Schlenk tube equipped with a magnetic stirrer bar were added 1,8-diazafluoran-9-one (**4**) (0.500 g, 2.75 mmol) and *N*-Boc-imino-(triphenyl)phosphorane (1.09 g, 2.89 mmol). The sealed tube was evacuated and refilled with N_2 three times, followed by addition of dry toluene (3.0 mL). Upon stirring at 110 °C for 48 hours, the resulting mixture was concentrated via rotary evaporator under reduced pressure and purified via flash chromatography on silica gel (petroleum ether : ethyl acetate : triethylamine = 2:1:0.06) to give the compound **30** (0.349 g, 45% yield) as a yellow solid.

30: Yellow solid; M.p. 176-177 °C; ^{1}H NMR (400 MHz, $CDCl_3$) δ 8.61 (brs, 1H), 8.53 (brs, 1H), 7.88 (d, J = 8.0 Hz, 2H), 7.35 (dd, J = 6.4, 5.2 Hz, 2H), 1.65 (s, 9H); ^{13}C NMR (100 MHz, $CDCl_3$) δ 161.0, 160.3, 154.2, 150.7, 149.8, 148.9, 135.8, 135.0, 128.4, 126.5, 83.3, 28.3; HRMS (ESI): *m/z* Calcd. For $C_{16}H_{16}N_3O_2$ $(M + H)^+$: 282.1237; Found: 282.1241.

Step ii. **Synthesis of compound 32**

To a stirred solution of compound **30** (0.45 g, 1.60 mmol) in THF (5.0 mL) was add a solution of freshly prepared allylic zinc chloride **31**[31] (4.8 mL, 0.5 M solution in THF, 2.4 mmol) dropwise at -78 °C under nitrogen over 5 min. Upon being stirred at -78 °C overnight, the reaction mixture was quenched with saturated NH_4Cl (15 mL) and extracted with ethyl acetate (15 mL × 3). The combined organic layers were dried over anhydrous Na_2SO_4, filtered, concentrated, and purified via flash chromatography on silica gel (petroleum ether / ethyl acetate = 2:1) to give the compound **32** (0.319 g, 50% yield) as a white solid.

32: White solid; M.p. 189-190 °C; ^{1}H NMR (400 MHz, $CDCl_3$) δ 8.58 (d, J = 4.8 Hz, 1H), 8.54 (d, J = 4.4 Hz, 1H), 7.57 (d, J = 7.6 Hz, 1H), 7.49 (d, J = 7.6 Hz, 1H), 7.21 (dd, J = 7.6, 5.2 Hz, 1H), 7.10 (dd, J = 7.6, 5.2 Hz, 1H), 6.95-6.79 (m, 2H), 6.72 (dd, J = 8.0, 7.2 Hz, 2H), 6.23 (d, J = 7.6 Hz, 2H), 5.79 (brs, 1H), 5.52-5.40 (m, 2H), 4.17 (d, J = 10.8 Hz, 1H), 1.24 (s, 7H for major isomer), 0.73

(s, 2H for minor isomer); ^{13}C NMR (100 MHz, $CDCl_3$) δ 165.1, 164.3, 153.5, 148.4, 148.2, 136.9, 135.9, 133.2, 132.3, 128.3, 127.6, 127.5, 127.2, 126.6, 123.2, 122.9, 120.2, 79.7, 66.5, 58.0, 28.1; HRMS (ESI): *m/z* Calcd. For $C_{25}H_{26}N_3O_2$ $(M + H)^+$: 400.2020; Found: 400.2019.

Step iii. **Synthesis of compound 25**

To a 25-mL Schlenk tube equipped with a magnetic stirrer bar was added compound **32** (0.100 g, 0.25 mmol). The sealed tube was evacuated and refilled with N_2 three times, followed by addition of dry DCM (2.0 mL). After cooling to 0 °C, a HCl solution (4 M in dioxane, 2.0 mL) was added. Upon stirring at 0 °C for 1 hour, the mixture was concentrated in vacuo to afford compound **25** as a brick red solid (0.102 g, 99% yield), which was used for next step without further purification.

25: brick red solid; M.p. decomposed at 173-174 °C; ^{1}H NMR (400 MHz, D_2O) δ 8.66 (d, J = 5.2 1H), 8.58 (d, J = 4.8 Hz, 1H), 7.99 (d, J = 8.0 Hz, 1H), 7.89 (d, J = 8.0 Hz, 1H), 7.56 (dd, J = 7.2, 5.2 Hz, 1H), 7.43 (dd, J = 7.2, 5.2 Hz, 1H), 6.95 (t, J = 7.2 Hz, 1H), 6.88-6.74 (m, 3H), 6.44 (d, J = 7.6 Hz, 2H), 5.65 (d, J = 16.8 Hz, 1H), 5.57 (d, J = 10.0 Hz, 1H), 4.47 (d, J = 10.4 Hz, 1H); ^{13}C NMR (100 MHz, D_2O) δ 158.4, 157.6, 148.94, 148.92, 135.5, 133.6, 133.2, 132.8, 130.3, 128.03, 127.96, 127.5, 125.9, 125.7, 122.1, 65.0, 55.7; HRMS (ESI): *m/z* Calcd. For $C_{20}H_{18}N_3$ $(M + H)^+$: 300.1495; Found: 300.1495.

(ii) Aza-Cope rearrangement of compound 21b

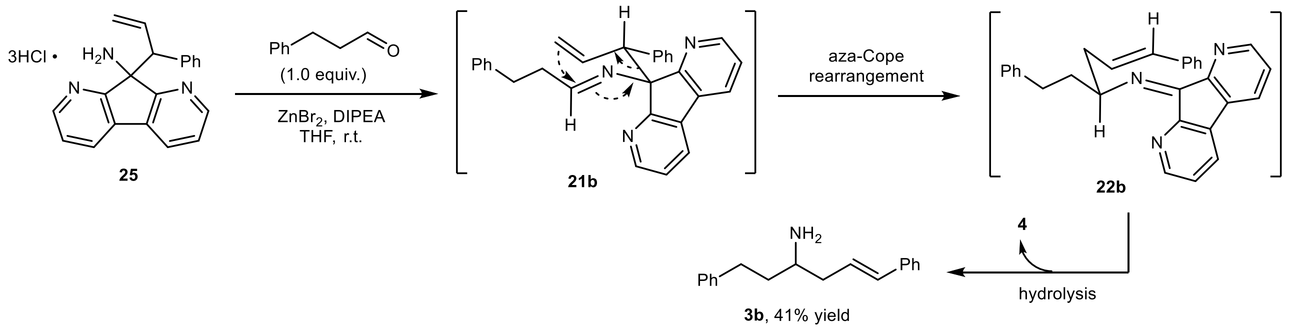

To a 25-mL Schlenk tube charged with a magnetic stirrer bar was added compound **25** (0.102 g, 0.25 mml). The sealed tube was evacuated and refilled with N_2 three times, followed by addition of dry THF (1.0 mL) and *N*,*N*-diisopropylethylamine (DIPEA) (0.101 g, 0.78 mmol). After the mixture was stirred at room temperature for 10 min, a solution of $ZnBr_2$ (0.056 g, 0.25 mmol) in THF (0.5 mL) and 3-phenylpropionaldehyde (0.034 g, 0.25 mmol) were added via a syringe. Upon stirring at room temperature for 1 hour, NH_2OH (1.5 mL, 50 wt% in water) was added to quench the reaction and the

resulting mixture was stirred for 1 hour. The mixture was diluted with water (2 mL) and extracted with ethyl acetate (5 mL × 3). The combined organic layers were dried over anhydrous Na_2SO_4, filtered, concentrated, and purified via column chromatography on silica gel (petroleum ether / ethyl acetate / dichloromethane / triethylamine = 2:1:0.3:0.03, the silica gel column was eluted with 1% v/v solution of Et_3N in petroleum ether before sample loading) to afford the compound **3b** (0.026 g, 41% yield) as a yellow oil.

2.2 Synthesis of Alkyl Amine 1d-*d*

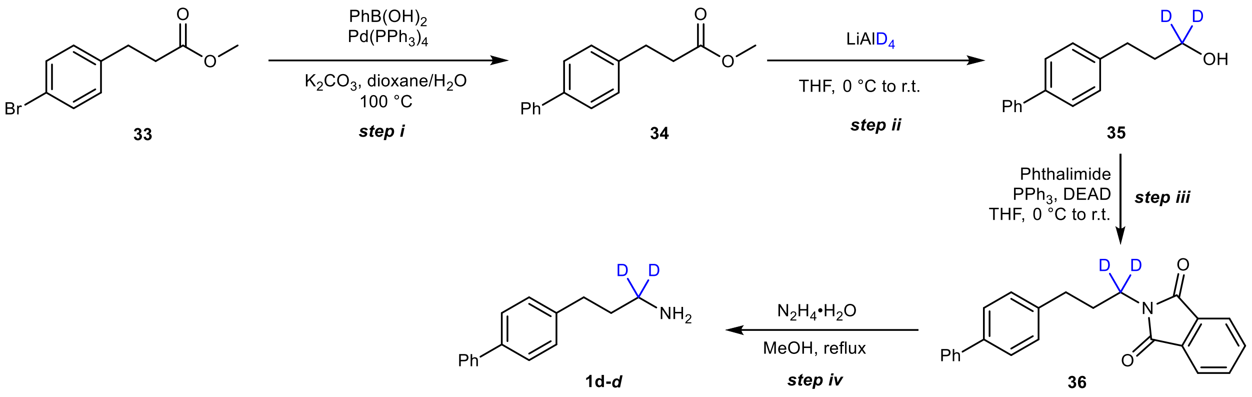

Step i. Synthesis of compound 34

To a 250-mL round-bottom flask equipped with a magnetic stirrer bar were added phenylboronic acid (3.76 g, 30.82 mmol), $Pd(PPh_3)_4$ (0.590 g, 0.51 mmol) and K_2CO_3 (8.50 g, 61.59 mmol). The sealed flask was evacuated and refilled with N_2 three times, followed by addition of dioxane (40 mL), H_2O (5 mL) and compound **33** (5.00 g, 20.58 mmol) via a syringe. The reaction mixture was stirred at 100 °C for 12 hours. After cooling to room temperature, the reaction mixture was diluted with water (40 mL), and extracted with ethyl acetate water (40 mL × 3). The combined organic layers were dried over anhydrous Na_2SO_4, filtered, concentrated, and purified via column chromatography on silica gel (petroleum ether : ethyl acetate = 10:1) to give compound **34** (4.20 g, 85% yield) as a white solid.

34: White solid; M.p. 51-53 °C; ^{1}H NMR (400 MHz, $CDCl_3$) δ 7.58 (dd, J = 7.2, 1.2 Hz, 2H), 7.53 (dd, J = 8.4, 2.0 Hz, 2H), 7.43 (t, J = 8.6 Hz, 2H), 7.37-7.31 (m, 1H), 7.28 (d, J = 8.0 Hz, 2H), 3.69 (s, 3H), 3.01 (t, J = 7.6 Hz, 2H), 2.68 (t, J = 7.6 Hz, 2H); ^{13}C NMR (100 MHz, $CDCl_3$) δ 173.5, 141.1, 139.7, 139.4, 128.9, 128.8, 127.4, 127.3, 127.1, 51.8, 35.8, 30.7; HRMS (ESI): *m/z* Calcd. For $C_{16}H_{17}O_2$ $(M + H)^+$: 241.1223; Found: 241.1227.

Step ii. Synthesis of compound 35

To a stirred solution of **34** (4.10 g, 17.08 mmol) in dry THF (34 mL) was slowly added $LiAlD_4$ (0.785 g, 18.69 mmol) over 2.5 min at 0 °C and the reaction mixture was allowed to warm up to room temperature. Upon being stirred at room temperature for 2 hours, the reaction was quenched by dropwise addition of water (0.8 mL), 10 % NaOH aqueous solution (1.6 mL) and water (2.4 mL) at 0 °C. The resulting mixture was filtered and washed with DCM. The combined filtrates were

concentrated via rotary evaporator to remove the solvent and purified via column chromatography on silica gel (petroleum ether / ethyl acetate = 5:1) to afford compound **35** (3.65 g, 99% yield) as a white solid.

35: White solid; M.p. 69-70 °C; ^{1}H NMR (400 MHz, $CDCl_3$) δ 7.60 (d, *J* = 8.0 Hz, 2H), 7.54 (d, *J* = 8.0 Hz, 2H), 7.45 (t, *J* = 7.6 Hz, 2H), 7.38-7.32 (m, 1H), 7.29 (d, *J* = 8.0 Hz, 2H), 2.77 (t, *J* = 8.0 Hz, 2H), 1.94 (t, *J* = 8.0 Hz, 2H); ^{13}C NMR (100 MHz, $CDCl_3$) δ 141.2, 141.1, 139.0, 129.0, 128.9, 127.3, 127.2, 127.1, 61.7 (quintet, *J* = 21.0 Hz), 34.1, 31.8; HRMS (ESI): *m/z* Calcd. For $C_{15}H_{15}D_2O$ $(M + H)^+$: 215.1399; Found: 215.1401.

***Step iii*. Synthesis of compound 36**

To a dry 250-mL round-bottom flask equipped with a magnetic stirrer bar were added compound **35** (3.75 g, 17.52 mmol), phthalimide (2.84 g, 19.32 mmol) and triphenylphosphine (5.06 g, 19.32 mmol). The sealed flask was evacuated and refilled with N_2 three times, followed by addition of dry THF (55 mL) via a syringe. After cooling to 0 °C, diethyl azodicarboxylate (DEAD) (4.58 g, 26.32 mmol) was slowly added over 4 min via syringe. Upon being stirred at room temperature for 12 hours, the reaction was quenched with water (100 mL), and extracted with ethyl acetate (100 mL × 3). The combined organic layers were dried over anhydrous Na_2SO_4, filtered, concentrated, and purified via column chromatography on silica gel (petroleum ether / ethyl acetate = 10:1) to afford compound **36** (5.85 g, 97% yield) as a white solid.

36: White solid; M.p. 95-96 °C; ^{1}H NMR (400 MHz, $CDCl_3$) δ 7.83 (dd, *J* = 5.6, 3.2 Hz, 2H), 7.68 (dd, *J* = 5.6, 3.2 Hz, 2H), 7.54 (dd, *J* = 8.4, 1.6 Hz, 2H), 7.48 (dd, *J* = 6.0, 1.6 Hz, 2H), 7.41 (td, *J* = 7.2, 1.6 Hz, 2H), 7.35-7.29 (m, 1H), 7.27 (dd, *J* = 8.0, 1.6 Hz, 2H), 2.74 (t, *J* = 8.0 Hz, 2H), 2.08 (t, *J* = 8.0 Hz, 2H); ^{13}C NMR (100 MHz, $CDCl_3$) δ 168.5, 141.0, 140.2, 139.0, 133.9, 132.2, 128.80, 128.75, 127.2, 127.09, 127.05, 123.2, 37.4 (quintet, *J* = 20.7 Hz), 32.9, 29.6; HRMS (ESI): *m/z* Calcd. For $C_{23}H_{18}D_2NO_2$ $(M + H)^+$: 344.1614; Found: 344.1617.

Step iv*. Synthesis of compound 1d-*d

To a 250-mL three-neck round-bottom flask equipped with a magnetic stirrer bar and a condenser were added compound **36** (5.85 g, 17.06 mmol), hydrated hydrazine (85 wt%, 2.92 mL, 51.16 mmol) and methanol (85 mL). Upon reflux for 2 hours, conc. HCl (13.6 mL) was added via a syringe and the reaction mixture was stirred for 1 hour. After cooling to room temperature, the reaction mixture was

concentrated via rotary evaporator. The resulting residue was dissolved in 4 M NaOH aqueous solution (150 mL) at 0 °C, and extracted with ether (150 mL × 3). The organic layers were combined, washed with brine (150 mL), dried over anhydrous Na_2SO_4, filtered, concentrated via rotary evaporation, and purified via column chromatography on silica gel (dichloromethane : methanol : 2.9 M ammonia solution in ethanol = 30:1:1) to give compound **1d-*d*** (3.44 g, 95% yield) as a colorless oil.

1d-*d*: Colorless oil; ^{1}H NMR (400 MHz, $CDCl_3$) δ 7.62 (d, *J* = 7.6 Hz, 2H), 7.55 (d, *J* = 8.4 Hz, 2H), 7.45 (t, *J* = 7.6 Hz, 2H), 7.35 (t, *J* = 7.2 Hz, 1H), 7.29 (d, *J* = 8.4 Hz, 2H), 2.72 (t, *J* = 8.0 Hz, 2H), 1.81 (t, *J* = 8.0 Hz, 2H), 1.14 (brs, 2H); ^{13}C NMR (100 MHz, $CDCl_3$) δ 141.3, 141.0, 138.7, 128.8, 128.7, 127.05, 126.99, 126.94, 41.0 (quintet, *J* = 20.2 Hz), 35.2, 32.8; HRMS (ESI): *m/z* Calcd. For $C_{15}H_{16}D_2N$ $(M + H)^+$: 214.1559; Found: 214.1563.

2.3 Kinetic Isotope Effect Studies

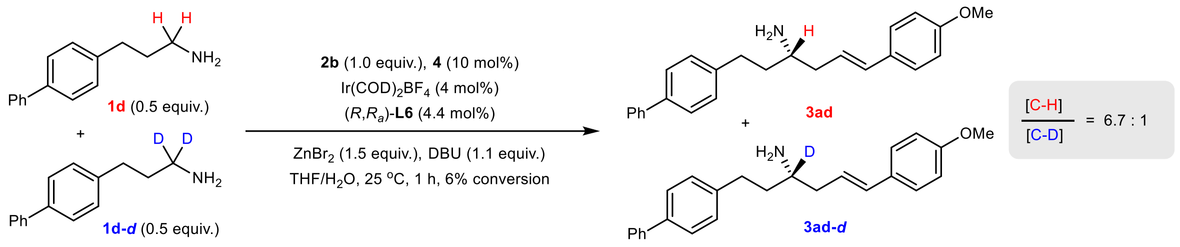

In a glove box, to a dry 5-mL vial equipped with a magnetic stirrer bar were charged with $Ir(COD)_2BF_4$ (5.9 mg, 0.012 mmol), (R,R_a)-**L6** (8.8 mg, 0.0132 mmol) and THF (0.2 mL). The vial was sealed and taken out of the glove box. The mixture was stirred at 50 °C for 30 min and then cooled down to room temperature, which was used as the solution of Ir catalyst. In the glove box, to an 8 mL vial equipped with a magnetic stirrer bar were added 1,8-diazafluoran-9-one (**4**) (5.5 mg, 0.03 mmol), dry THF (0.20 mL), primary amine **1d** (0.0319 g, 0.15 mmol), primary α,α-d_2-amine **1d-*d*** (99%, 0.0331 g, 0.15 mmol), and $ZnBr_2$ (0.101 g, 0.45 mmol, dissolved in 0.30 mL THF). The Schlenk tube was sealed and taken out of the glove box. After being stirred at room temperature for 30 min, to the mixture were added allylic carbonate **2b** (0.067 g, 0.30 mmol, dissolved in 0.4 mL THF), 1,8-diazabicyclo[5.4.0]undec-7-ene (DBU) (0.050 g, 0.33 mmol), the pre-prepared solution of Ir catalyst, THF (0.35 mL) and water (0.050 mL). Upon stirring at room temperature for 1 hour, $NH_2OH{\cdot}HCl$ solution (0.041 g, 0.59 mmol, dissolved in 0.5 mL 1 M HCl aqueous solution) was added to quench the reaction, followed by addition of ammonium hydroxide (10 mL, 25-28 wt% in water) and extraction with DCM (20 mL × 3). The combined organic layers were dried over Na_2SO_4, filtered, and concentrated under reduced pressure and purified via column chromatography on silica gel (petroleum ether : ethyl acetate : dichloromethane : triethylamine = 50:50:10:1, the silica gel column was eluted with 1% v/v solution of Et_3N in petroleum ether before sample loading) to afford a mixture of compounds **3ad** and **3ad-*d*** as pale yellow solids. The conversion of allylic carbonate **2b** was determined as 6% by ^1H NMR analysis of the crude reaction mixture. The ratio of products **3ad** and **3ad-*d*** was determined as 6.7:1 by ^1H NMR analysis of the purified products (Supplementary Fig. 7).

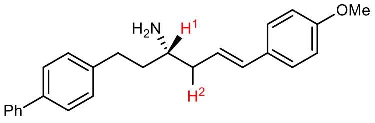

3ad

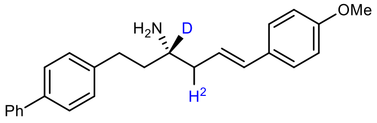

3ad-*d*

$$\frac{[\text{C-H}]}{[\text{C-D}]} = \frac{\textbf{3ad}\ (\text{H}^1)}{\textbf{3ad}\ (\text{H}^2) + \textbf{3ad-}\textbf{\textit{d}}\ (\text{H}^2) - \textbf{3ad}\ (\text{H}^1)} = \frac{0.87}{1-0.87} = 6.7:1$$

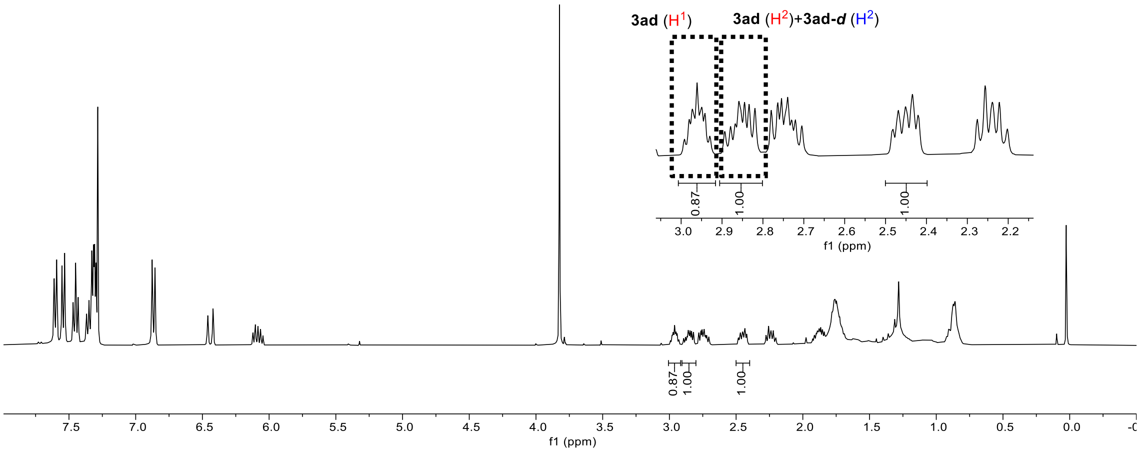

Supplementary Fig. 7. The ratio of products **3ad** and **3ad-*d*** determined by ^{1}H NMR

2.4 Investigation on the Influence of $ZnBr_2$

(i) Synthesis of imine 37

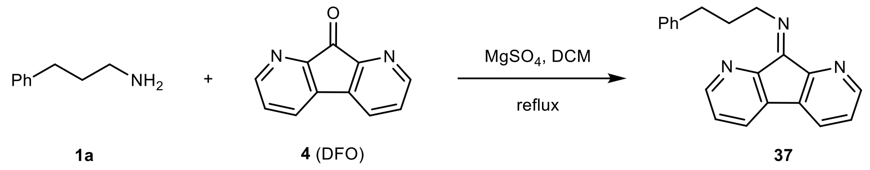

To a dry 50-mL round-bottom flask equipped with a magnetic stirrer bar were added DFO (0.728 g, 4.00 mmol), $MgSO_4$ (1.44 g, 12.00 mmol), dry DCM (15 mL) and **1a** (0.546 g, 4.04 mmol). The flask was equipped with a condenser and stirred at reflux for 22 hours. After being cooled down to room temperature, the reaction mixture was filtered through a pad of Celite and washed with DCM. The combined filtrates were concentrated under reduced pressure to give compound **37** (1.13 g, 94% yield) as bluish green oil.

37: Bluish green oil; ^{1}H NMR (400 MHz, $CDCl_3$) δ 8.63 (d, J = 4.0 Hz, 1H), 8.58 (d, J = 4.4 Hz, 1H), 7.92 (d, J = 7.6 Hz, 1H), 7.86 (d, J = 7.6 Hz, 1H), 7.34-7.21 (m, 6H), 7.20-7.12 (m, 1H), 4.84 (t, J = 7.2 Hz, 2H), 2.87 (t, J = 7.2 Hz, 2H), 2.39-2.27 (m, 2H); ^{13}C NMR (100 MHz, $CDCl_3$) δ 159.6, 154.8, 151.4, 150.5, 149.2, 142.6, 135.3, 132.9, 128.6, 128.3, 127.8, 125.7, 124.9, 124.4, 53.7, 34.1, 32.8; HRMS (ESI): *m/z* Calcd. For $C_{20}H_{18}N_3$ $(M + H)^+$: 300.1495; Found: 300.1492.

(ii) Synthesis of Catalyst 38

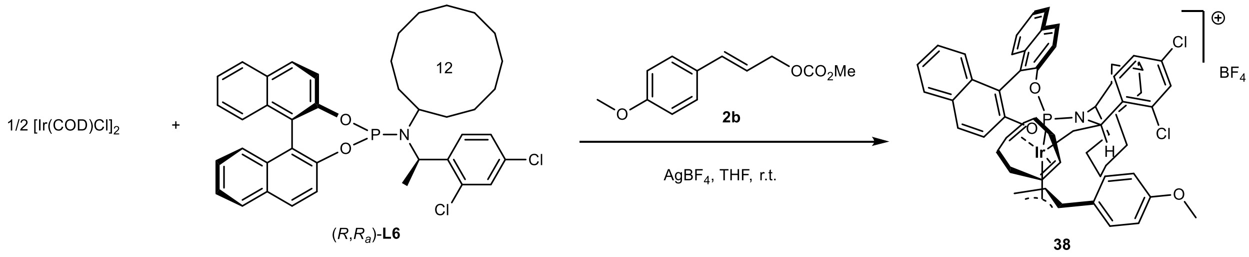

Iridium complex **38** was prepared according to the corresponding literature procedure[32]:

To a dry 10-mL Schlenk tube equipped with a magnetic stirrer bar were added $[Ir(COD)Cl]_2$ (0.134 g, 0.20 mmol), (R,R_a)-**L6** (0.268 g, 0.40 mmol) and dry THF (4 mL) in a glove box. After stirring at room temperature for 30 min, $AgBF_4$ (0.082 g, 0.42 mmol) and **2b** (0.356 g, 1.60 mmol) were added. The reaction mixture was stirred at room temperature for 21 hours. The formed precipitate was removed by filtration and the filtrate was concentrated and purified via column chromatography

on silica gel (DCM : *iso*-propanol = 97:3) to give the compound **38** (0.372 g, 77% yield) as a golden solid.

38: Golden solid; M.p. decomposed at 177-178 °C; $[\alpha]_D^{25}$ = +5.9 (*c* = 0.16, MeOH); ^{1}H NMR (400 MHz, CD_2Cl_2) δ 8.26-8.14 (m, 2H), 8.09-7.99 (m, 2H), 7.79-7.61 (m, 4H), 7.61-7.51 (m, 2H), 7.51-7.45 (m, 1H), 7.45-7.38 (m, 2H), 7.38-7.23 (m, 4H), 6.99 (d, *J* = 8.4 Hz, 2H), 5.95-5.79 (m, 1H), 5.17-4.96 (m, 2H), 4.63-4.52 (m, 1H), 4.25-4.13 (m, 1H), 4.04-3.93 (m, 1H), 3.85 (s, 3H), 3.61-3.51 (m, 1H), 3.07-2.78 (m, 3H), 2.58-2.42 (m, 2H), 2.40-2.28 (m, 1H), 2.24-2.10 (m, 2H), 1.96-1.82 (m, 1H), 1.79-1.53 (m, 4H), 1.48-0.23 (m, 22H); ^{13}C NMR (100 MHz, CD_2Cl_2) δ 161.7, 161.6, 148.6, 148.4, 148.14, 148.06, 139.4, 139.2, 134.4, 133.7, 133.2, 132.8, 132.6, 132.5, 131.0, 130.9, 130.1, 129.3, 129.1, 128.4, 127.9, 127.8, 127.6, 127.5, 126.9, 126.8, 125.84, 125.77, 122.3, 122.24, 122.15, 122.1, 121.19, 121.16, 121.10, 121.08, 115.82, 115.80, 101.6, 94.6, 94.5, 92.69, 92.66, 91.6, 91.4, 90.32, 90.27, 84.92, 84.86, 56.1, 40.2, 35.3, 33.8, 28.7, 27.5, 25.4, 25.2, 24.2, 23.5, 23.0, 22.8, 21.8; ^{31}P NMR (162 MHz, CD_2Cl_2) δ 118.6 (brs). HRMS (ESI): *m/z* Calcd. For $C_{58}H_{64}Cl_2IrNO_3P$ $(M - BF_4^-)^+$: 1116.3615; Found: 1116.3596.

(iii) Investigation on the impact of $ZnBr_2$

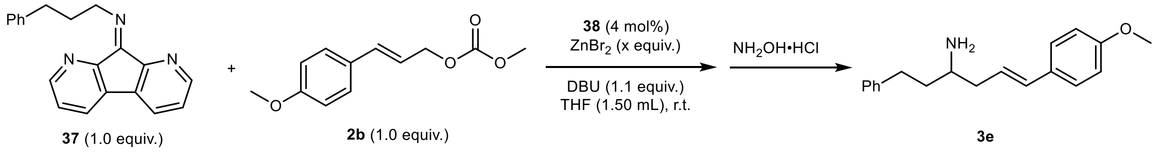

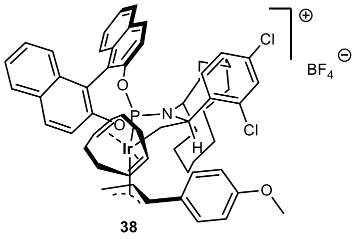

entry	$ZnBr_2$ (x equiv.)	yield (%)	ee (%)
1	1.1	61	95
2	0	54	99

To a 10-mL Schlenk tube equipped with a magnetic stirrer bar was added catalyst **38** (0.0144 g, 0.012 mmol). The sealed tube was evacuated and refilled with N_2 three times, followed by addition of imine **37** (0.090 g, 0.30 mmol, dissolve in 0.50 mL THF), **2b** (0.067 g, 0.30 mmol, dissolved in 0.50 mL THF), $ZnBr_2$ (0.074 g, 0.33 mmol, dissolved in 0.50 mL THF for entry 1), THF (0.50 mL for entry 2), and DBU (0.050 g, 0.33 mmol). Upon stirring at room temperature for 36 hours, $NH_2OH{\cdot}HCl$ (0.104 g, 1.50 mmol, dissolved in 1.5 mL of 1 M HCl aqueous solution) was added to quench the reaction. After stirring at room temperature for 1 hour, saturated Na_2CO_3 aqueous solution was added

till pH 9~10. To the resulting mixture was added H_2O (10 mL). The mixture was extracted with DCM (10 mL × 3). The combined organic layers were dried over anhydrous Na_2SO_4, filtered, concentrated, and purified via column chromatography on silica gel (petroleum ether : ethyl acetate : dichloromethane : triethylamine = 50:50:10: 1, the silica gel column was eluted with 1% v/v solution of Et_3N in petroleum ether before sample loading) to afford the compound **3e** (0.0514 g, 61% yield, 95% ee for entry 1; 0.0454 g, 54% yield, 99% ee for entry 2) as a brown solid.

***Discussions*:** In the absence of additive $ZnBr_2$, the reaction between imine **37** and allylic acetate **2b** still went smoothly to give the α-C–H allylic alkylation product **3e** with a similar yield and enantioselectivity. The comparison experiments demonstrated that $ZnBr_2$ has little influence on the asymmetric allylic substitution and subsequent aza-Cope rearrangement. The additive $ZnBr_2$ is supposed to acted as a Lewis acid, promoting the condensation of alkyl amine **1** with diazafluorenone **4** to form an imine that initiated the transformation. Additionally, $ZnBr_2$ also likely inhibited the undesired allylic amination of the primary amine **1** by coordinating with the NH_2 group.

2.5 Determination of pK_a Values by Calculation

Computational Methods**:** All calculations were carried out using the Gaussian 09 computational program[33]. The geometries were optimized by using the B3LYP density functional method[34] and 6-31G(d) basis sets for all atoms in the gas phase. The frequency analysis was performed at the same level of theory, so that to verify that all of the structures optimized did not have any imaginary frequency and to compute the Gibbs free-energy data. Single-point-energy calculations were performed on the basis of the optimized geometries by using the m06-2x method[35] with the 6-311++G(3df,2p) basis sets for all atoms, in which the solvation effect of dimethyl sulfoxide (DMSO) was treated by the SMD model[36]. In the Fukui-function analysis, electron-density distributions were obtained at the B3LYP/6-31+G(d) level, and the isosurfaces of Fukui functions were generated using the Multiwfn software[37].

Calculation Strategy**:** The *p*Ka values of the target acids were computed by using the proton-transfer method as reported in the literature[38]. Compound **39** with a reported pK_a of 22.1 (in DMSO)[39] was used as the reference acid in this method. The proton-transfer reaction in DMSO between the target acid (HA) and **39** was designed as shown below. The pK_a value of HA was derived on the basis of the free-energy change (ΔG_{soln}) of the proton-transfer reaction and the reported pK_a value of **39**[39], as shown in the equation below.

$$\text{HA} + \mathbf{39}^{\ominus} \xrightarrow[\text{DMSO}]{298.15\text{ K}} \text{A}^{\ominus} + \mathbf{39} \qquad \triangle G_{soln}$$

$$pK_a(\text{HA}) = \frac{\Delta G_{soln}}{RT\ln(10)} + pK_a(\mathbf{39}) = \frac{\Delta G_{soln}}{RT\ln(10)} + 22.1$$

Results**:** The calculated pK_a values of the α C–H bonds for compounds **1c**, **18c** and **18c′** were shown in the following figure (Supplementary Fig. 8). The calculated ΔG_{soln} values in the above proton-transfer reaction for **1c**, **18c**, and **18c′** were 47.6, -13.3, and -10.9 kcal/mol, respectively. Based on the reported pK_a (22.1)[39] of the α-amino C–H bond of 9-NH_2-fluorene (**39**), the pK_a values of the α C–H bonds for **1c**, **18c** and **18c′** were predicted as 57, 12.5 and 14 respectively.

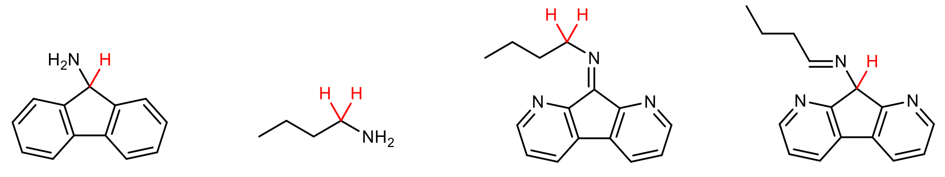

39, pK_a = 22.1 (DMSO) **1c**, pK_a = 57 **18c**, pK_a = 12.5 **18c'**, pK_a = 14

Supplementary Fig. 8 The calculated pK_a values of compounds **1c**, **18c**, and **18c′**

***Discussions*:** The α-amino C−H bond of *n*-butylamine (**1c**) is extremely low (pK_a 57). The formation of imine **18c** with 1,8-diazafluoren-9-one (DFO) dramatically improved the acidity of the C−H bond up to 10^{44} times (pK_a 12.5 of **18c** vs 57 of **1c**), demonstrating the extraordinary capability of DFO for the activation of the α-amino C−H bonds of alkyl amines.

Supplementary Table 4. Electronic energies and Gibbs free energies at the B3LYP/6-31G* level of theory in the gas phase, and singlet-point energies at the m06-2x/6-311++G(3df,2p) level of theory in DMSO. Energetic data are reported in Hartree.

**Species**	E^{B3}	G^{B3}	$E^{\mathrm{m06\text{-}2x}}$
**39**	-556.7862571	-556.616667	-556.7230991
**39**⁻	-556.216464	-556.062128	-556.2268571
**1c**	-213.8084986	-213.68852	-213.7665263
**1c**⁻	-213.1351031	-213.032477	-213.1923732
**18c**	-744.9005288	-744.673087	-744.8102311
**18c**⁻	-744.3577971	-744.144715	-744.3361323
**18c′**	-744.9015328	-744.674753	-744.8134764
**18c′**⁻	-744.3577972	-744.144715	-744.3361328

3. Supplementary Figures

3.1 NMR Spectra

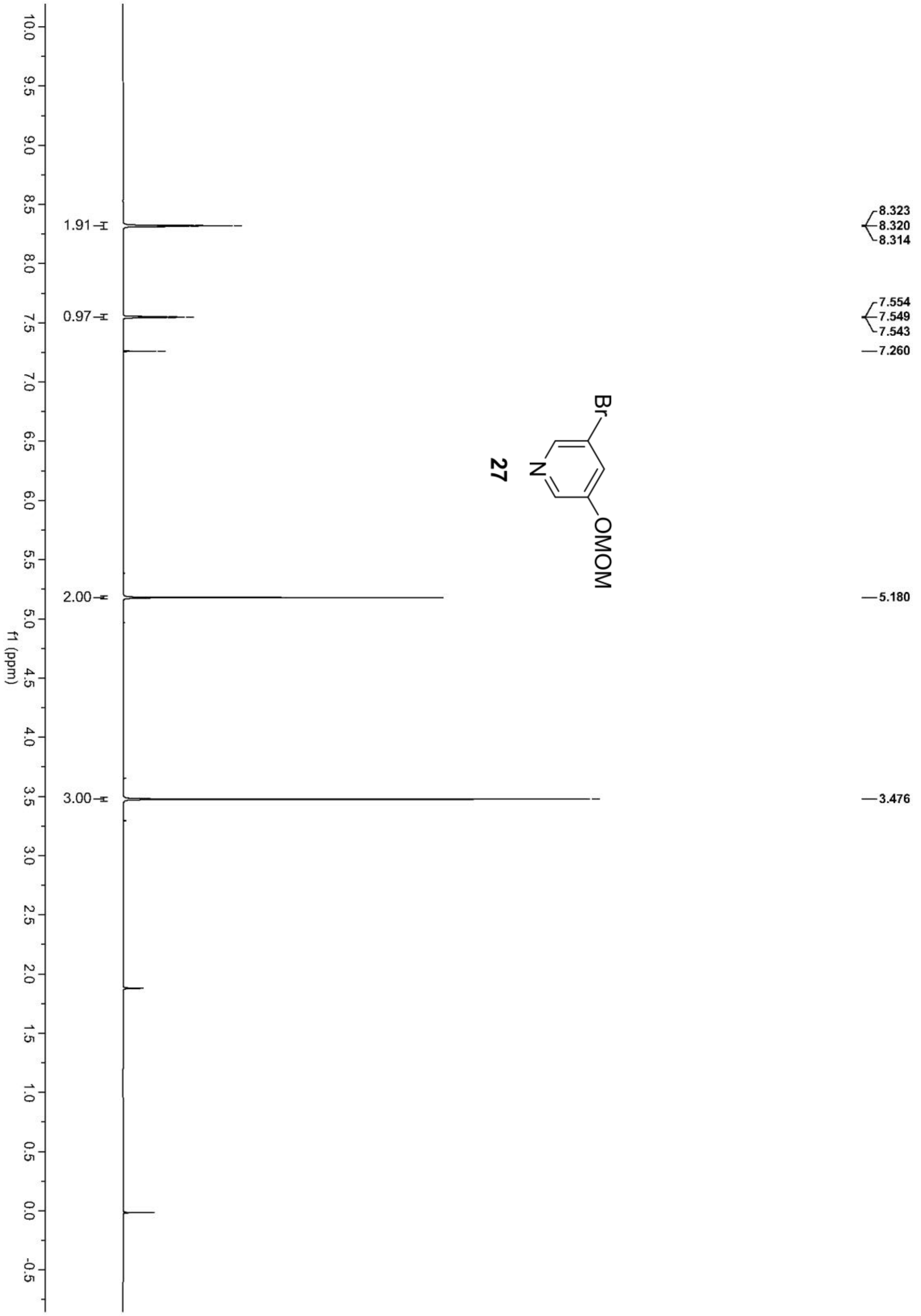

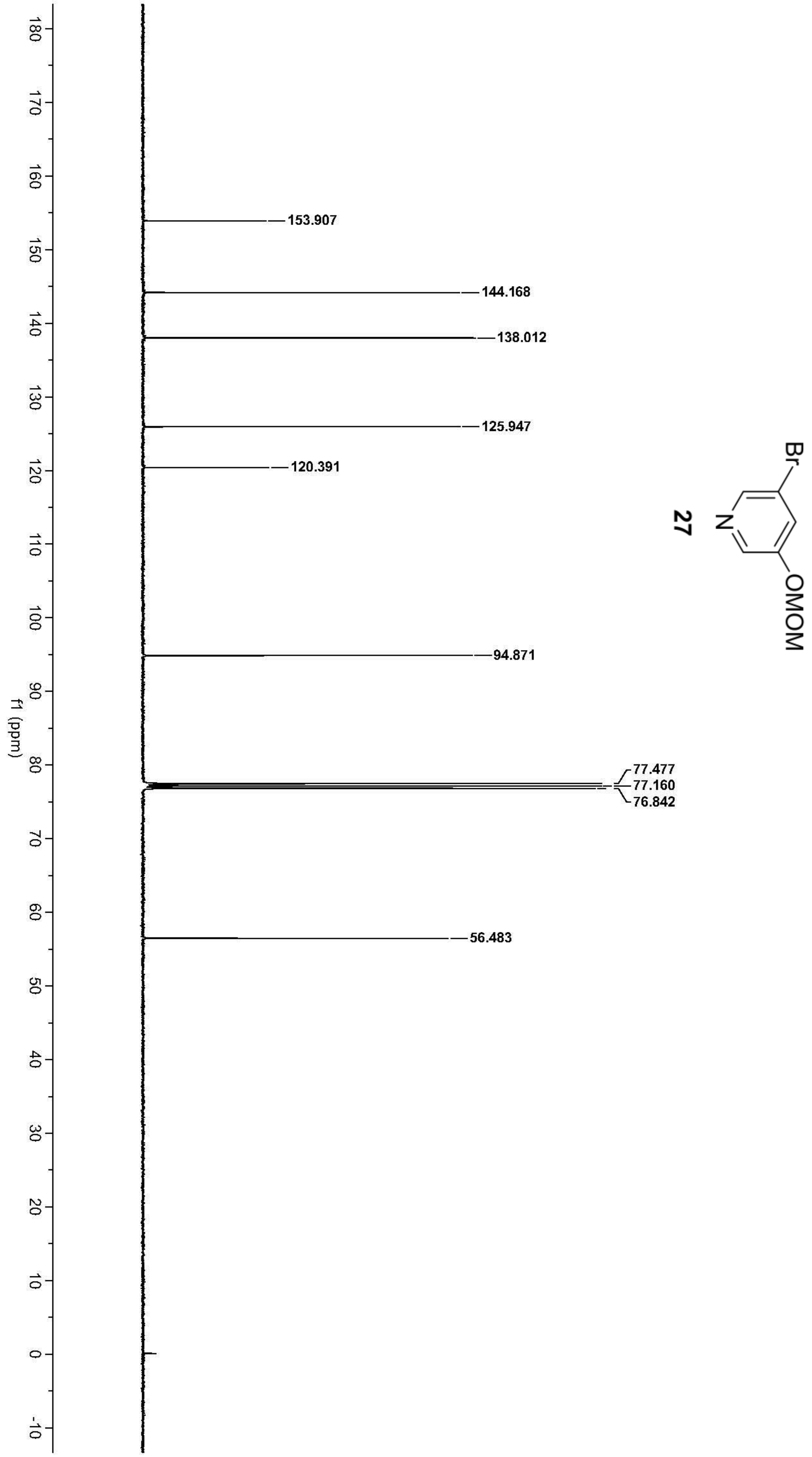
153.907
144.168
138.012
125.947
120.391
94.871
77.477
77.160
76.842
56.483
f1 (ppm)
Br
N
OMOM
27

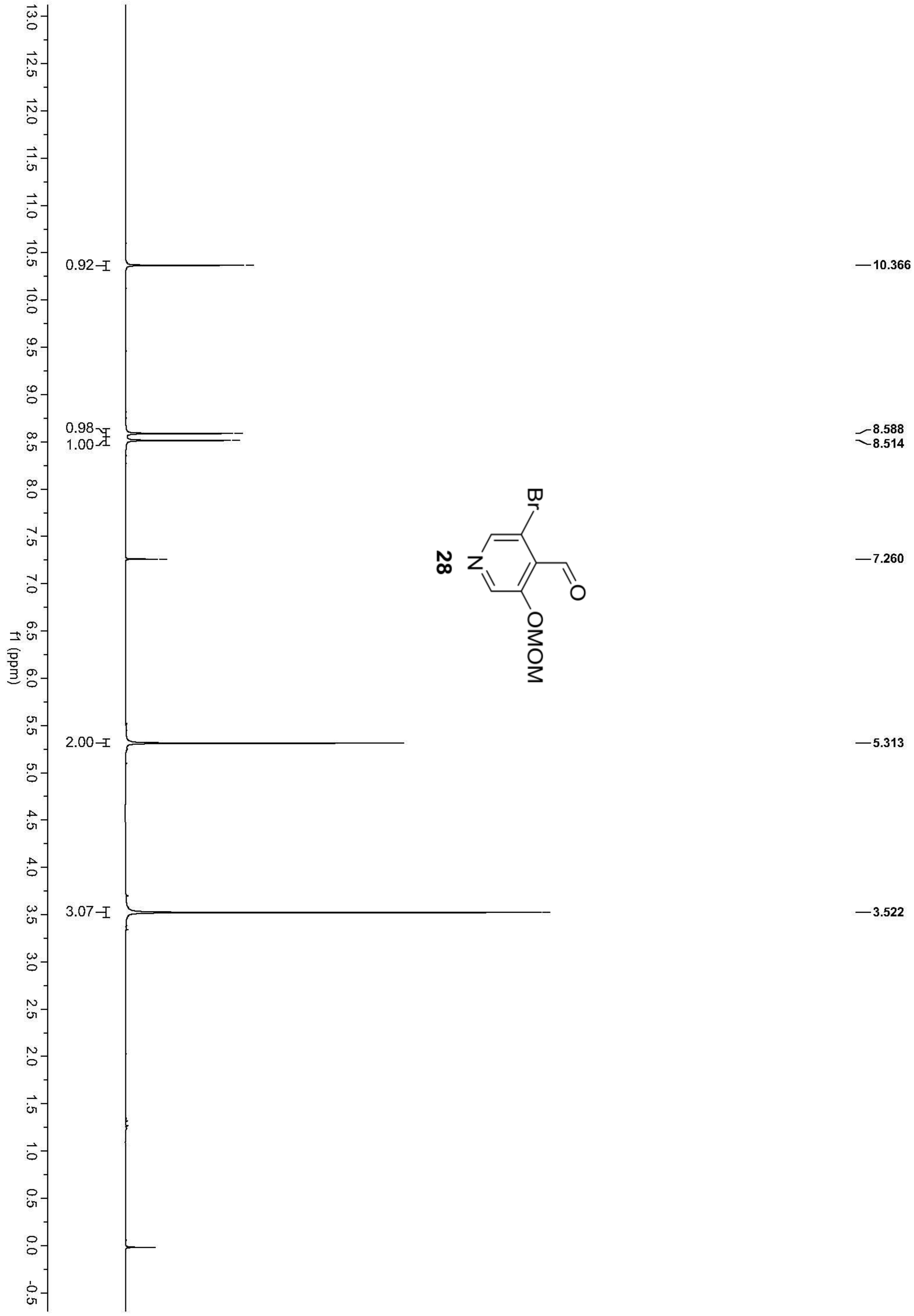

10.366
8.588
8.514
7.260
5.313
3.522
0.92
0.98
1.00
2.00
3.07
f1 (ppm)
Br
N
O
OMOM
28

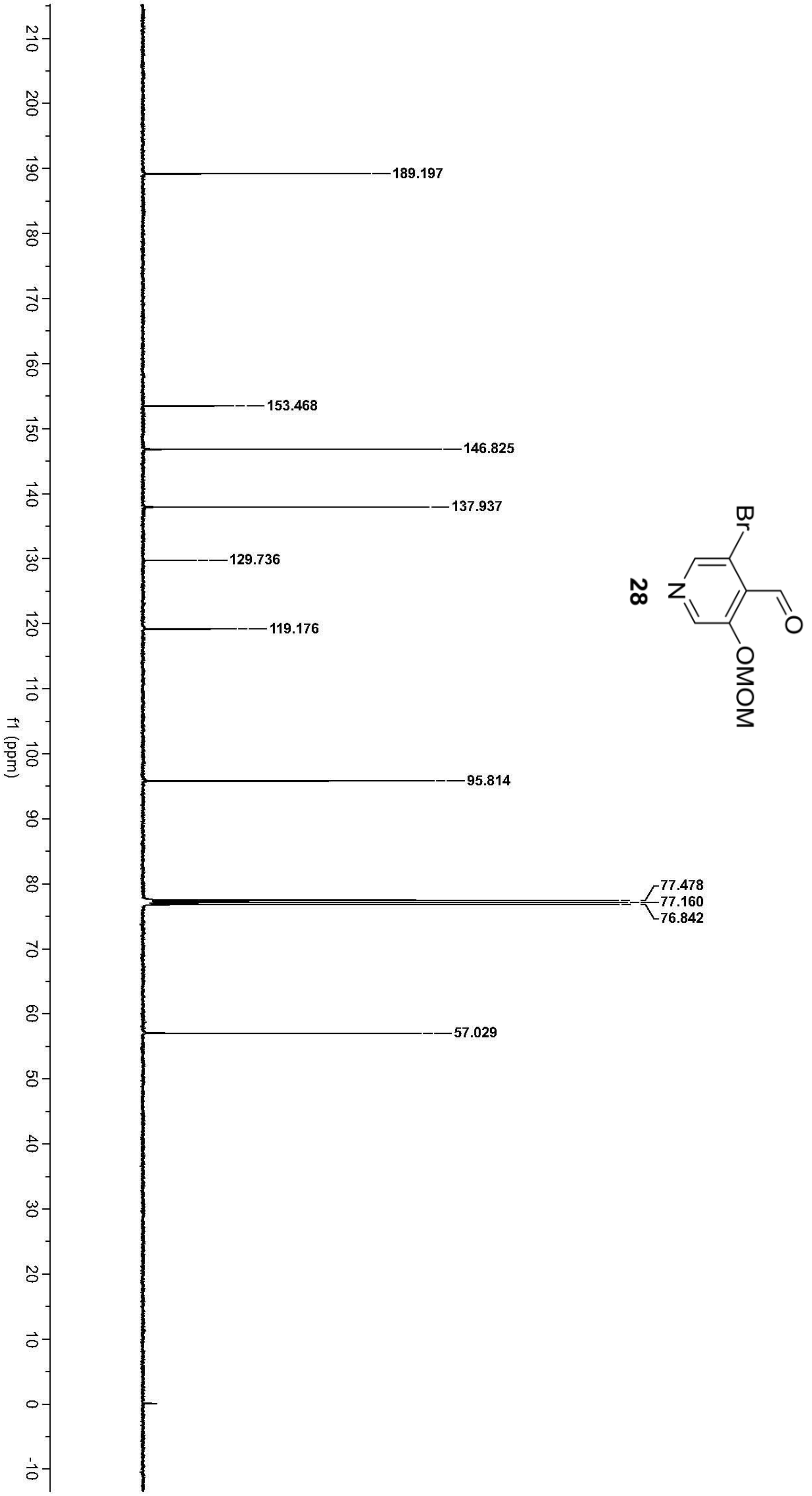

189.197
153.468
146.825
137.937
129.736
119.176
95.814
77.478
77.160
76.842
57.029
f1 (ppm)
Br
N
OMOM
O
28

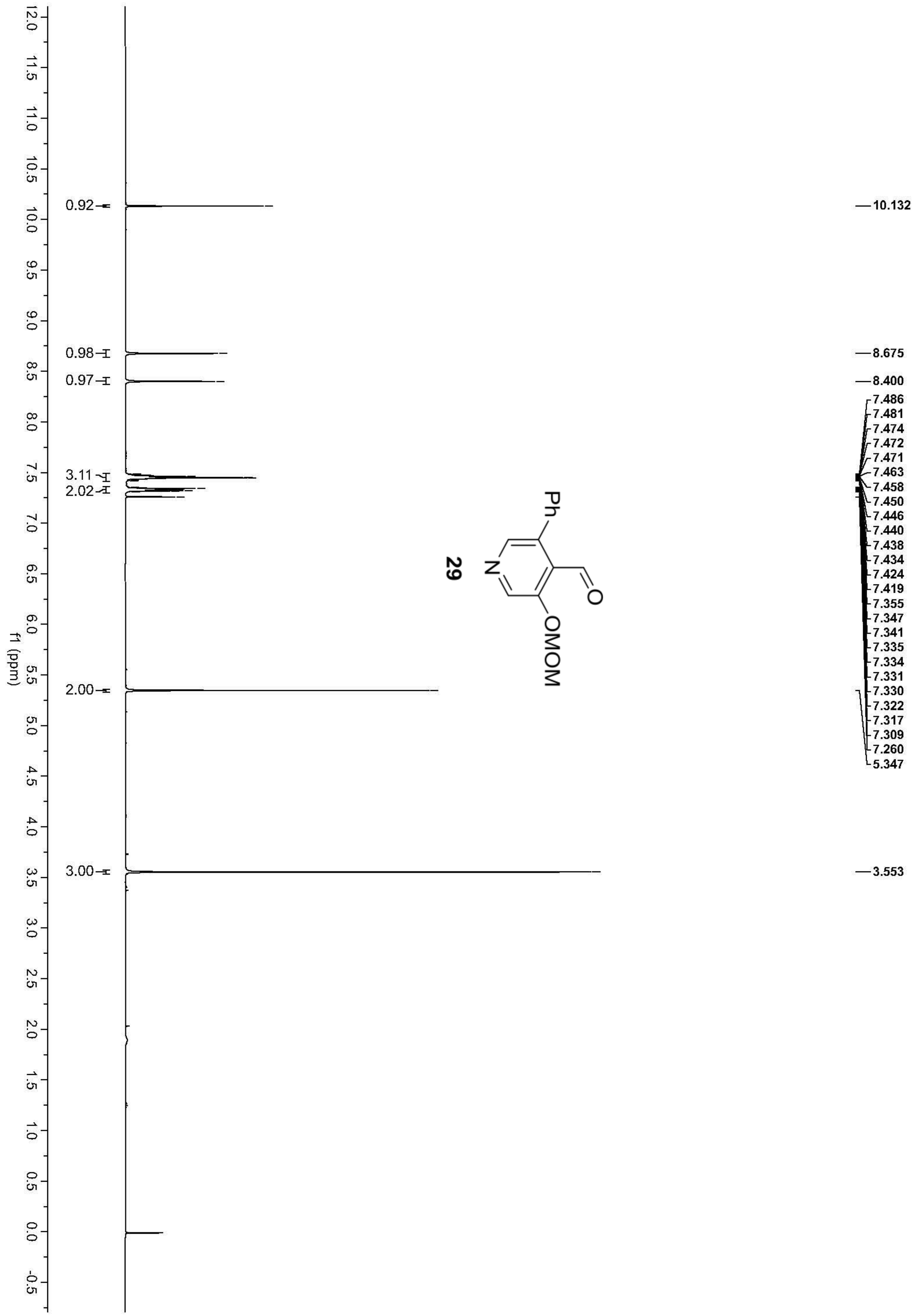
f1 (ppm)
12.0 11.5 11.0 10.5 10.0 9.5 9.0 8.5 8.0 7.5 7.0 6.5 6.0 5.5 5.0 4.5 4.0 3.5 3.0 2.5 2.0 1.5 1.0 0.5 0.0 -0.5
0.92
0.98
0.97
3.11
2.02
2.00
3.00
10.132
8.675
8.400
7.486
7.481
7.474
7.472
7.471
7.463
7.458
7.450
7.446
7.440
7.438
7.434
7.424
7.419
7.355
7.347
7.341
7.335
7.334
7.331
7.330
7.322
7.317
7.309
7.260
5.347
3.553
Ph
N
O
OMOM
29

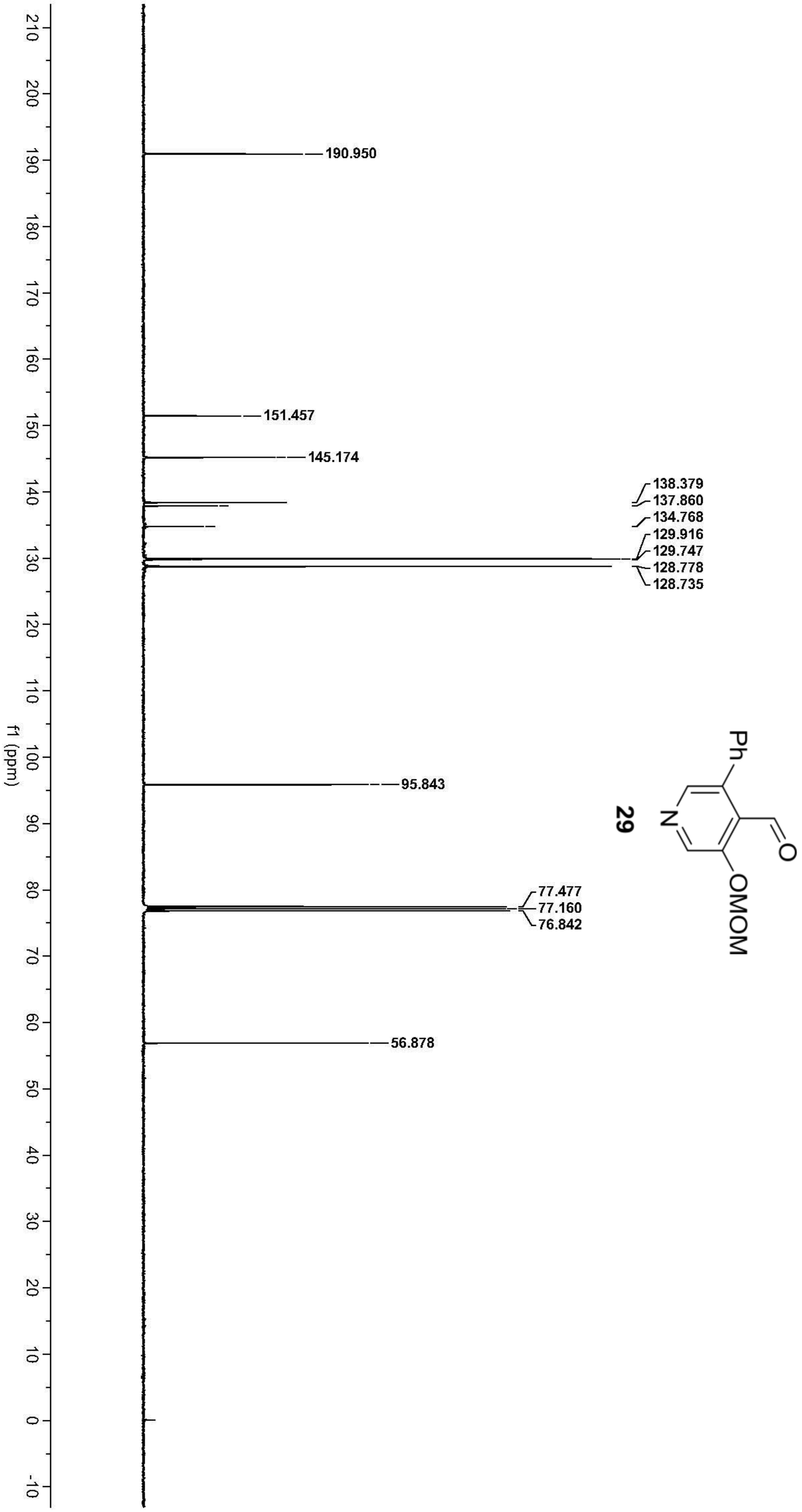
190.950
151.457
145.174
138.379
137.860
134.768
129.916
129.747
128.778
128.735
95.843
77.477
77.160
76.842
56.878
f1 (ppm)
Ph
N
O
OMOM
29

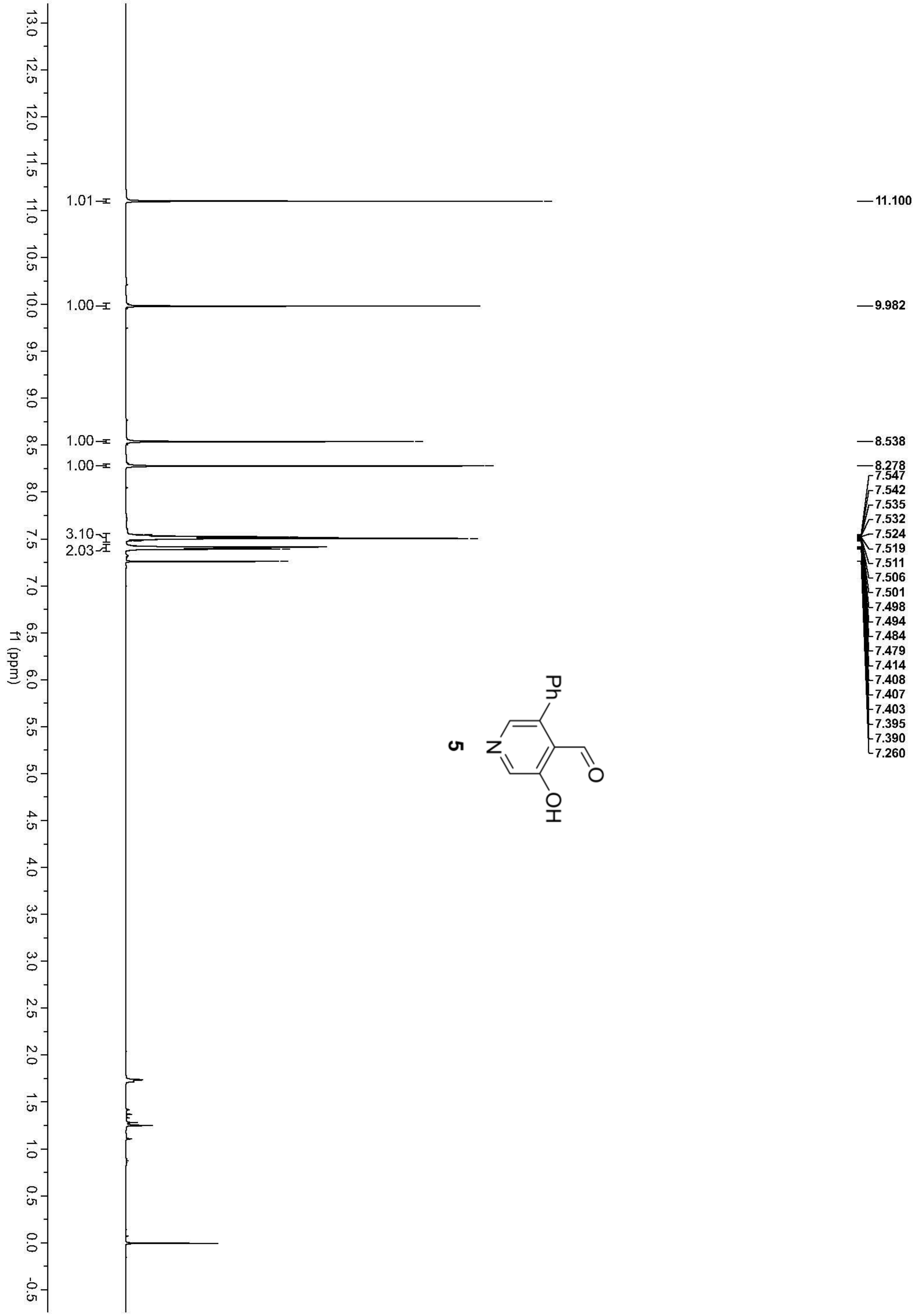

11.100
9.982
8.538
8.278
7.547
7.542
7.535
7.532
7.524
7.519
7.511
7.506
7.501
7.498
7.494
7.484
7.479
7.414
7.408
7.407
7.403
7.395
7.390
7.260
1.01
1.00
1.00
1.00
3.10
2.03
f1 (ppm)
Ph
N
OH
O
5

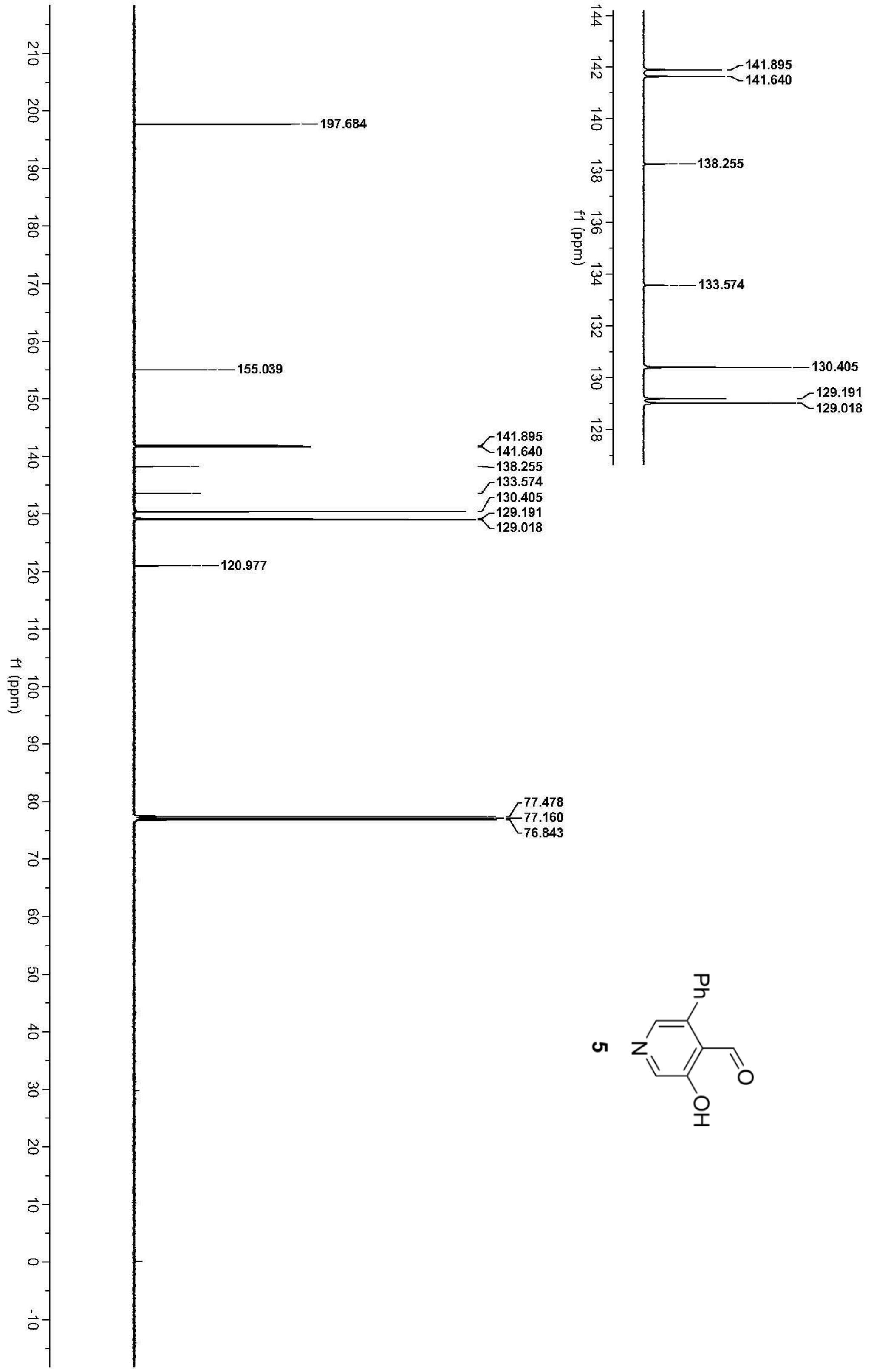
197.684
155.039
141.895
141.640
138.255
133.574
130.405
129.191
129.018
120.977
77.478
77.160
76.843
f1 (ppm)
Ph
N
OH
O
5

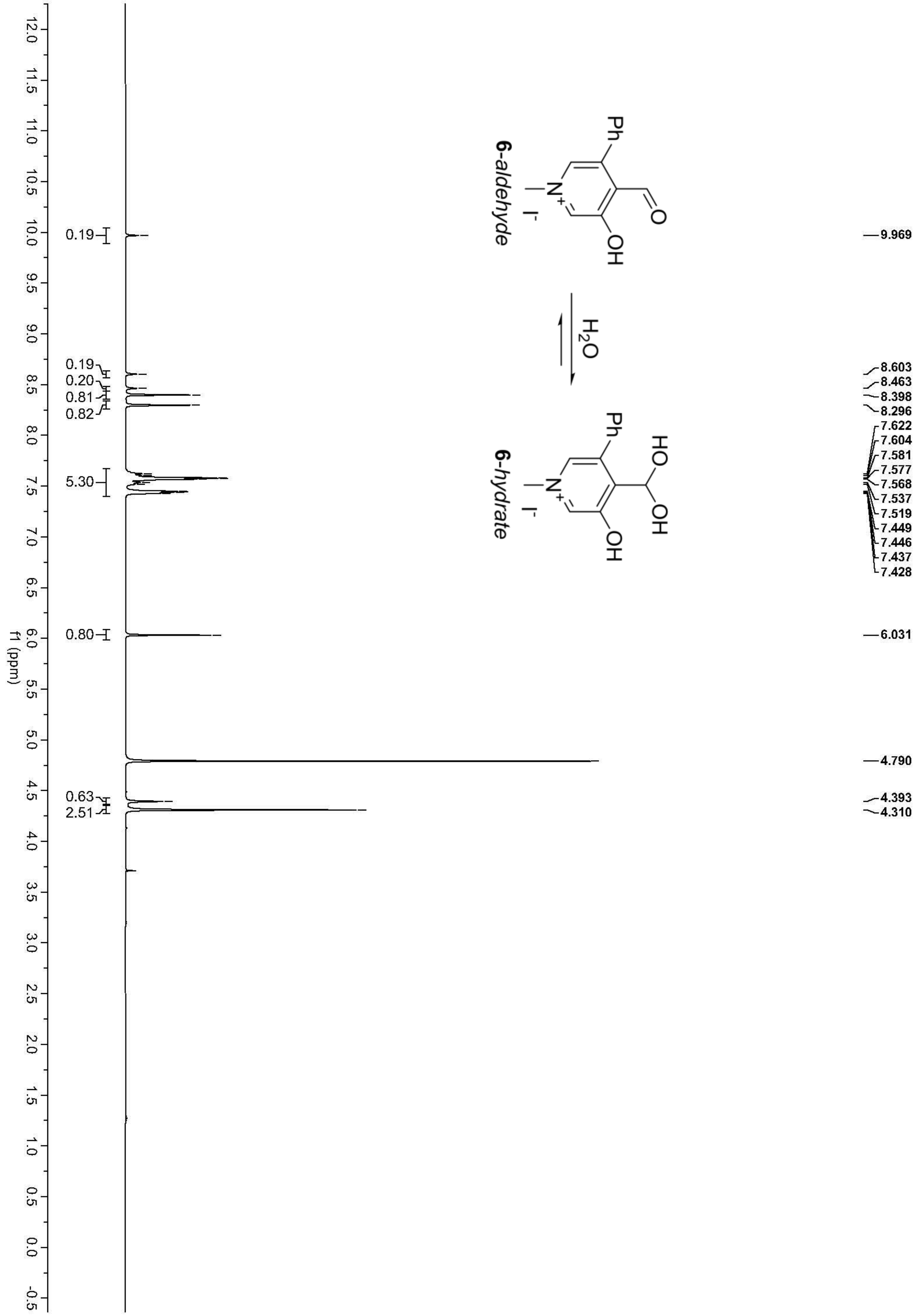
6-aldehyde
6-hydrate
H2O
Ph
OH
HO
N+
I-
9.969
8.603
8.463
8.398
8.296
7.622
7.604
7.581
7.577
7.568
7.537
7.519
7.449
7.446
7.437
7.428
6.031
4.790
4.393
4.310
0.19
0.19
0.20
0.81
0.82
5.30
0.80
0.63
2.51
f1 (ppm)

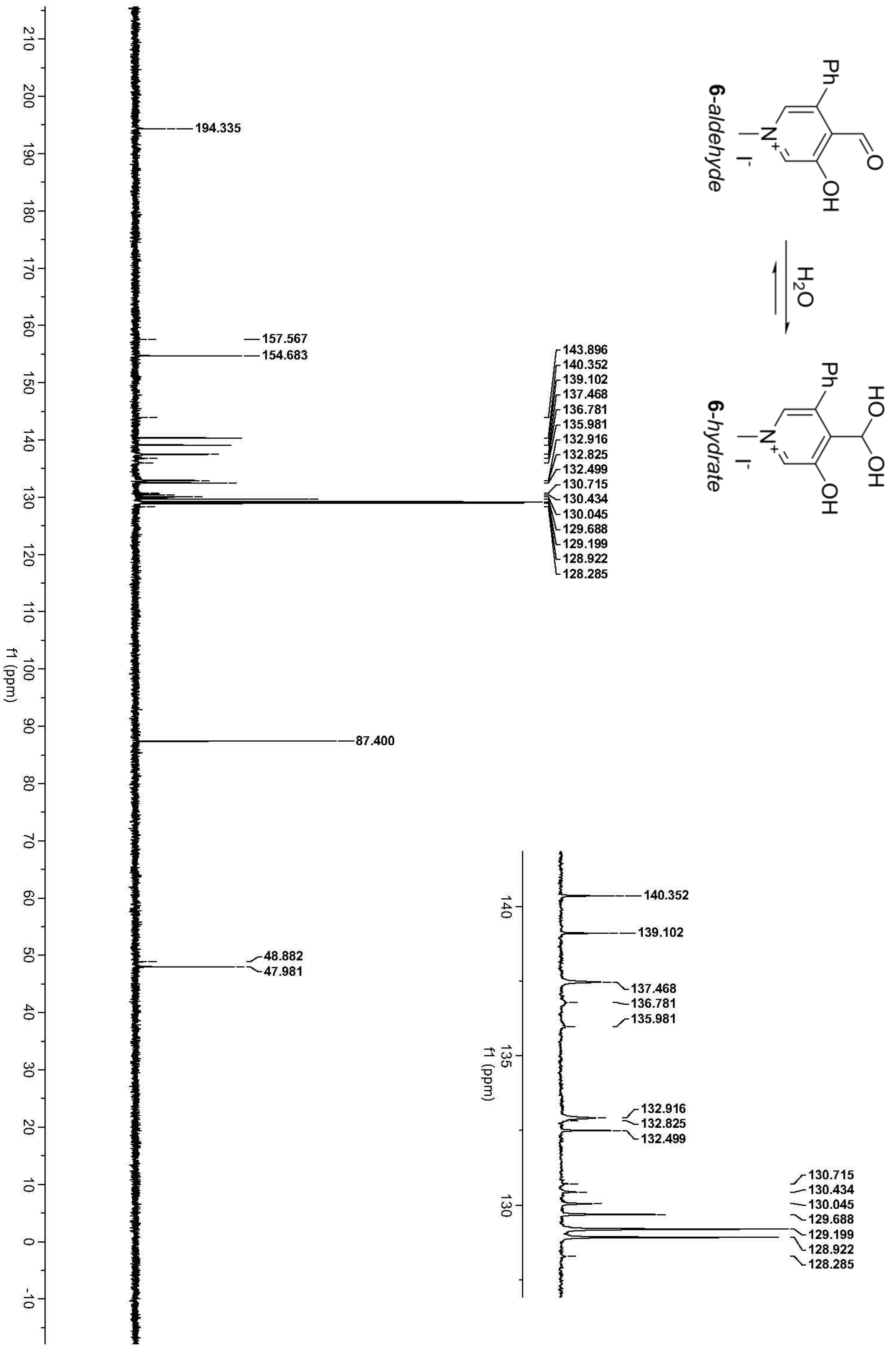
6-aldehyde
H_2O
6-hydrate
Ph
OH
HO
N^+
I^-
194.335
157.567
154.683
143.896
140.352
139.102
137.468
136.781
135.981
132.916
132.825
132.499
130.715
130.434
130.045
129.688
129.199
128.922
128.285
87.400
48.882
47.981
f1 (ppm)

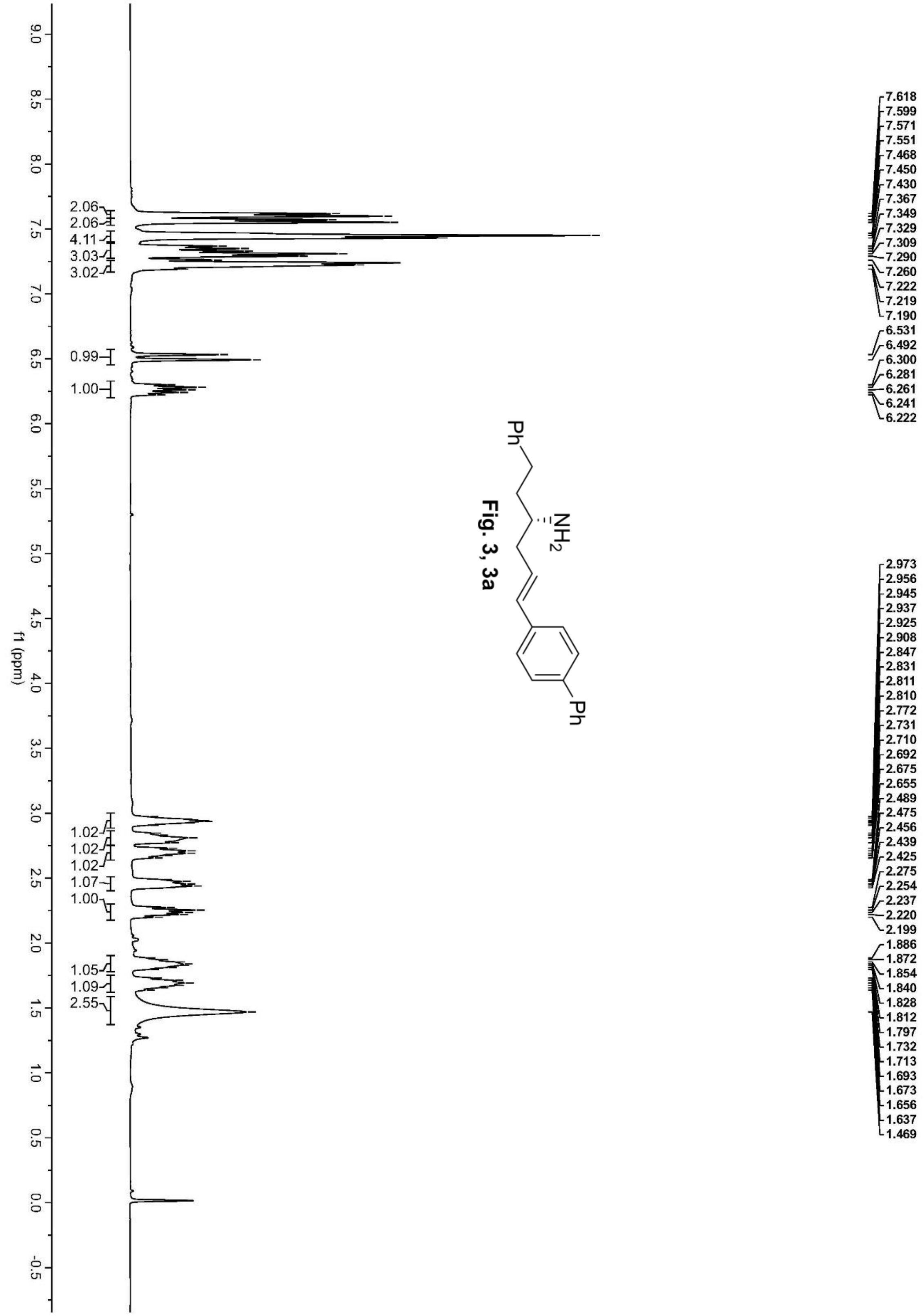

Ph
NH2
Ph
9.0
8.5
8.0
7.5
7.0
6.5
6.0
5.5
5.0
4.5
4.0
3.5
3.0
2.5
2.0
1.5
1.0
0.5
0.0
-0.5
f1 (ppm)
2.06
2.06
4.11
3.03
3.02
0.99
1.00
1.02
1.02
1.02
1.07
1.00
1.05
1.09
2.55
7.618
7.599
7.571
7.551
7.468
7.450
7.430
7.367
7.349
7.329
7.309
7.290
7.260
7.222
7.219
7.190
6.531
6.492
6.300
6.281
6.261
6.241
6.222
2.973
2.956
2.945
2.937
2.925
2.908
2.847
2.831
2.811
2.810
2.772
2.731
2.710
2.692
2.675
2.655
2.489
2.475
2.456
2.439
2.425
2.275
2.254
2.237
2.220
2.199
1.886
1.872
1.854
1.840
1.828
1.812
1.797
1.732
1.713
1.693
1.673
1.656
1.637
1.469

Fig. 3, 3a

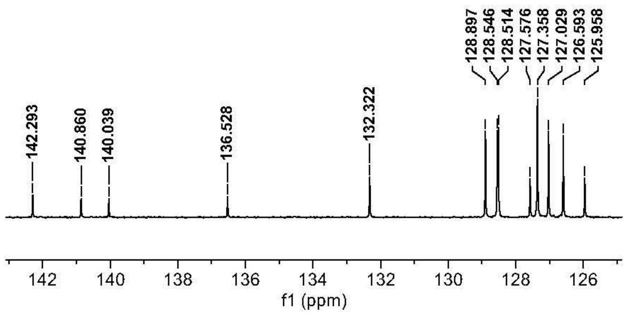

142.293
140.860
140.039
136.528
132.322
128.897
128.546
128.514
127.576
127.358
127.029
126.593
125.958
f1 (ppm)

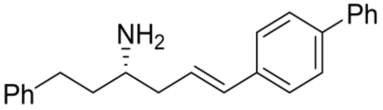

NH2
Ph
Ph

Fig. 3, 3a

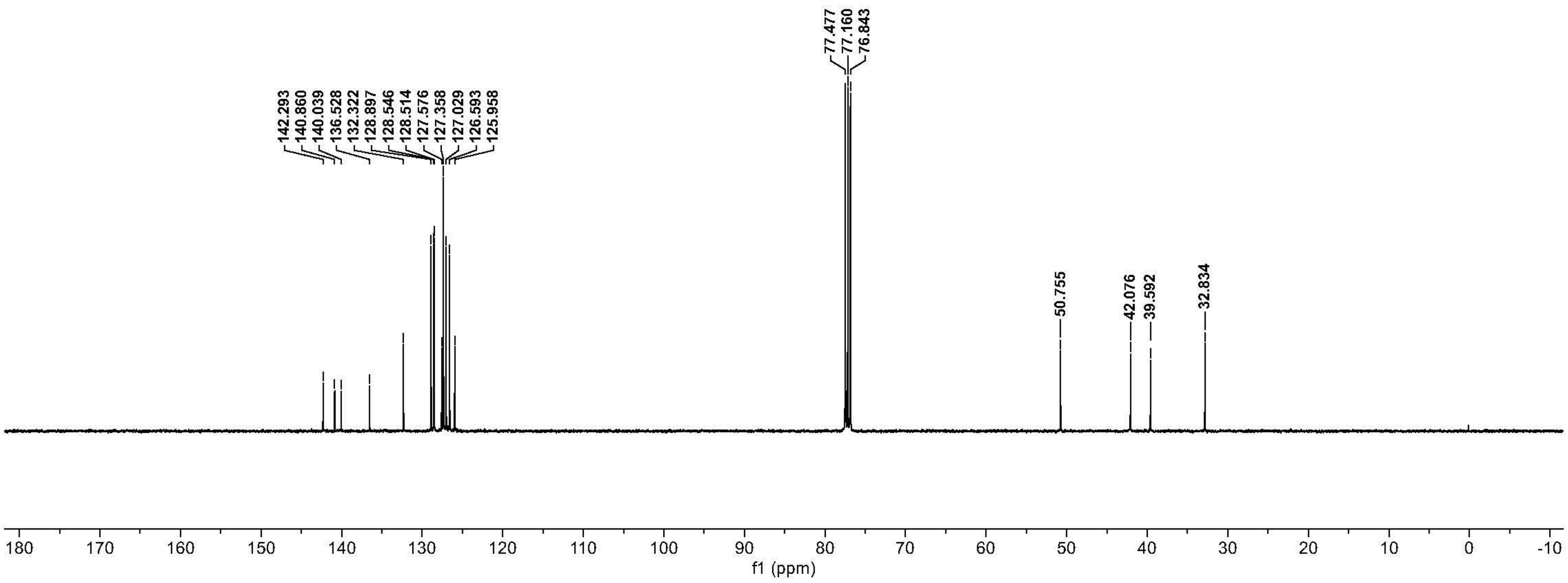

142.293
140.860
140.039
136.528
132.322
128.897
128.546
128.514
127.576
127.358
127.029
126.593
125.958
77.477
77.160
76.843
50.755
42.076
39.592
32.834
f1 (ppm)

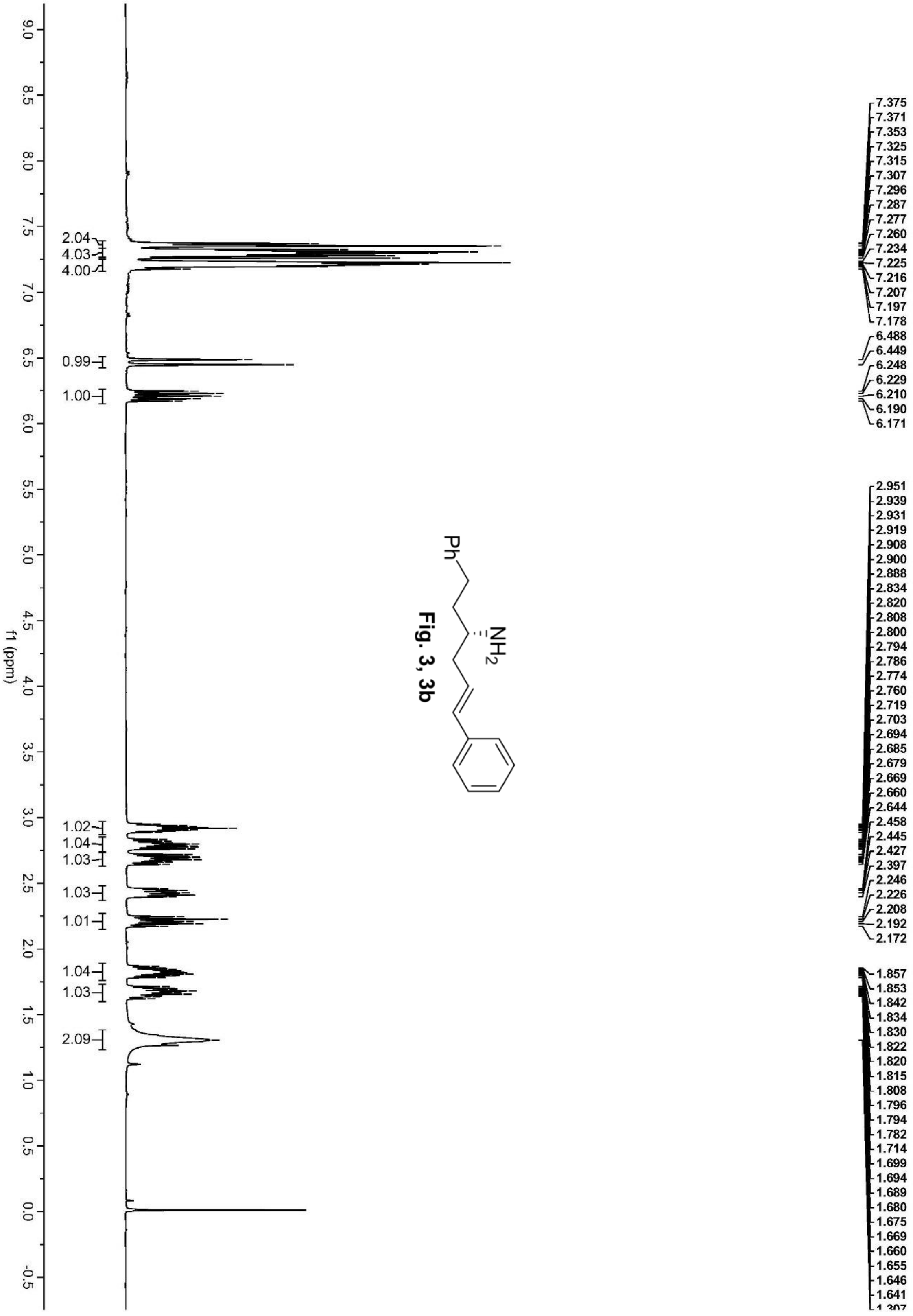
NH2
Ph
f1 (ppm)
2.04
4.03
4.00
0.99
1.00
1.02
1.04
1.03
1.03
1.01
1.04
1.03
2.09
7.375
7.371
7.353
7.325
7.315
7.307
7.296
7.287
7.277
7.260
7.234
7.225
7.216
7.207
7.197
7.178
6.488
6.449
6.248
6.229
6.210
6.190
6.171
2.951
2.939
2.931
2.919
2.908
2.900
2.888
2.834
2.820
2.808
2.800
2.794
2.786
2.774
2.760
2.719
2.703
2.694
2.685
2.679
2.669
2.660
2.644
2.458
2.445
2.427
2.397
2.246
2.226
2.208
2.192
2.172
1.857
1.853
1.842
1.834
1.830
1.822
1.820
1.815
1.808
1.796
1.794
1.782
1.714
1.699
1.694
1.689
1.680
1.675
1.669
1.660
1.655
1.646
1.641

Fig. 3, 3b

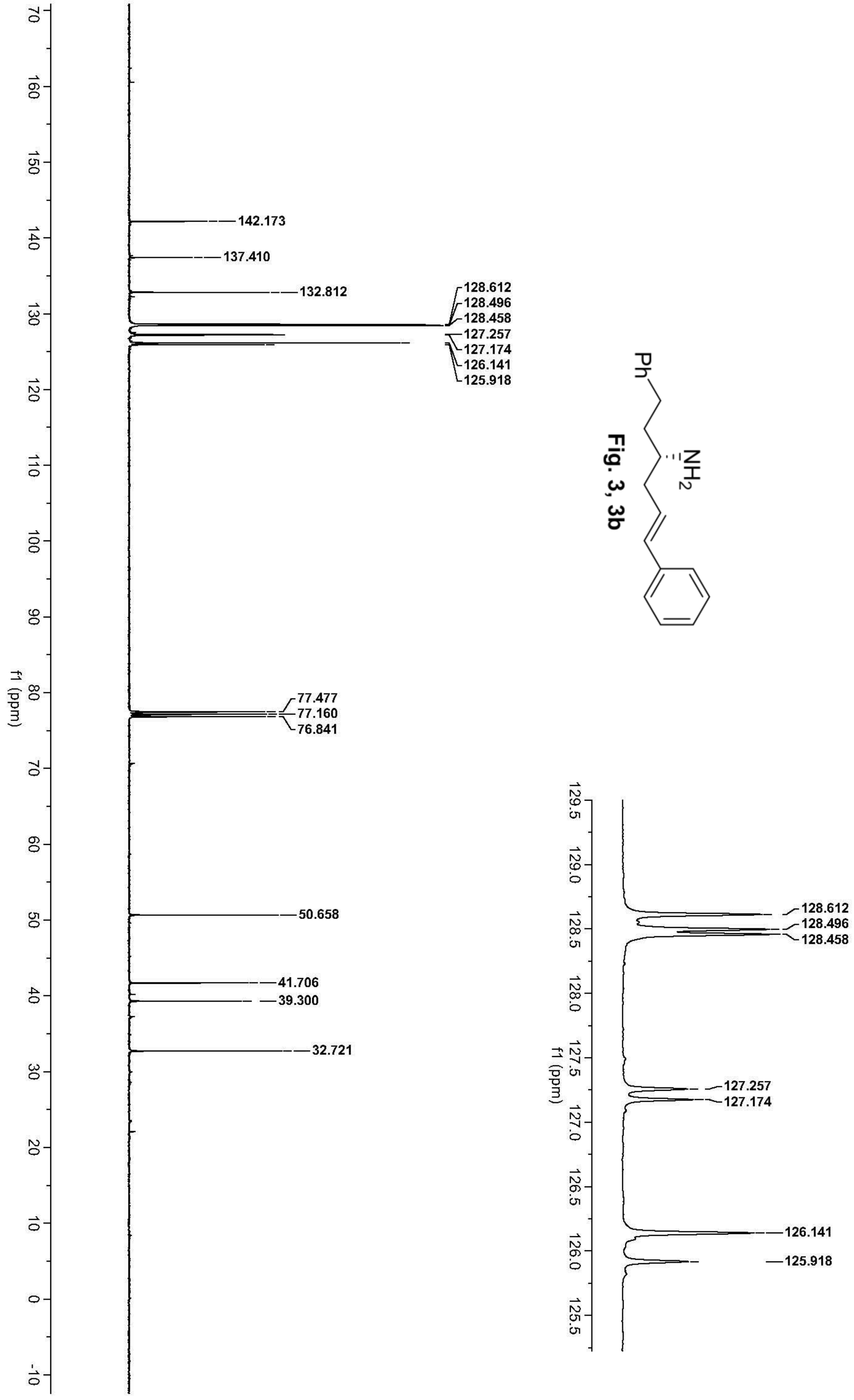

142.173
137.410
132.812
128.612
128.496
128.458
127.257
127.174
126.141
125.918
77.477
77.160
76.841
50.658
41.706
39.300
32.721
f1 (ppm)
Ph
NH_2

Fig. 3, 3b

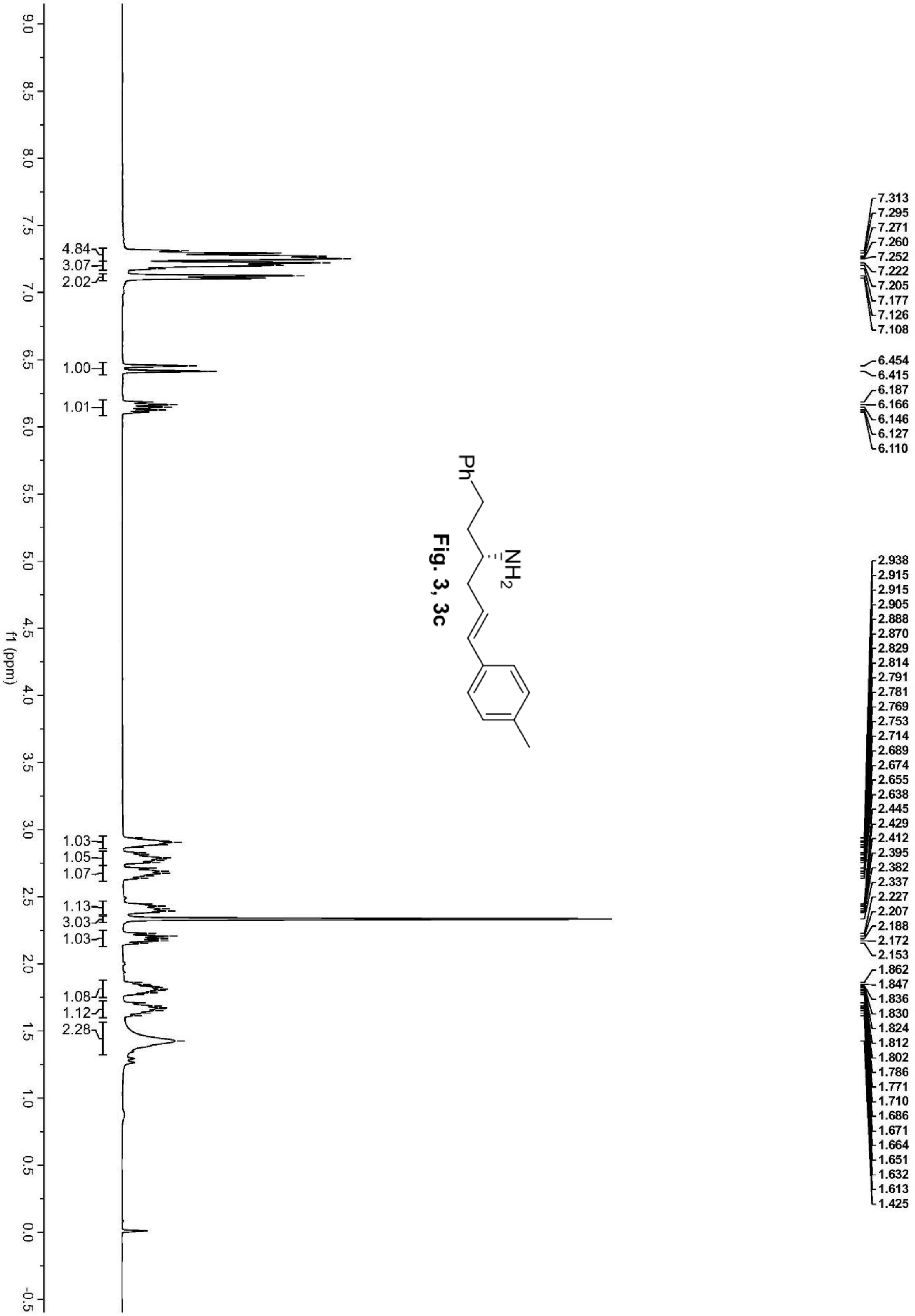

Fig. 3, 3c

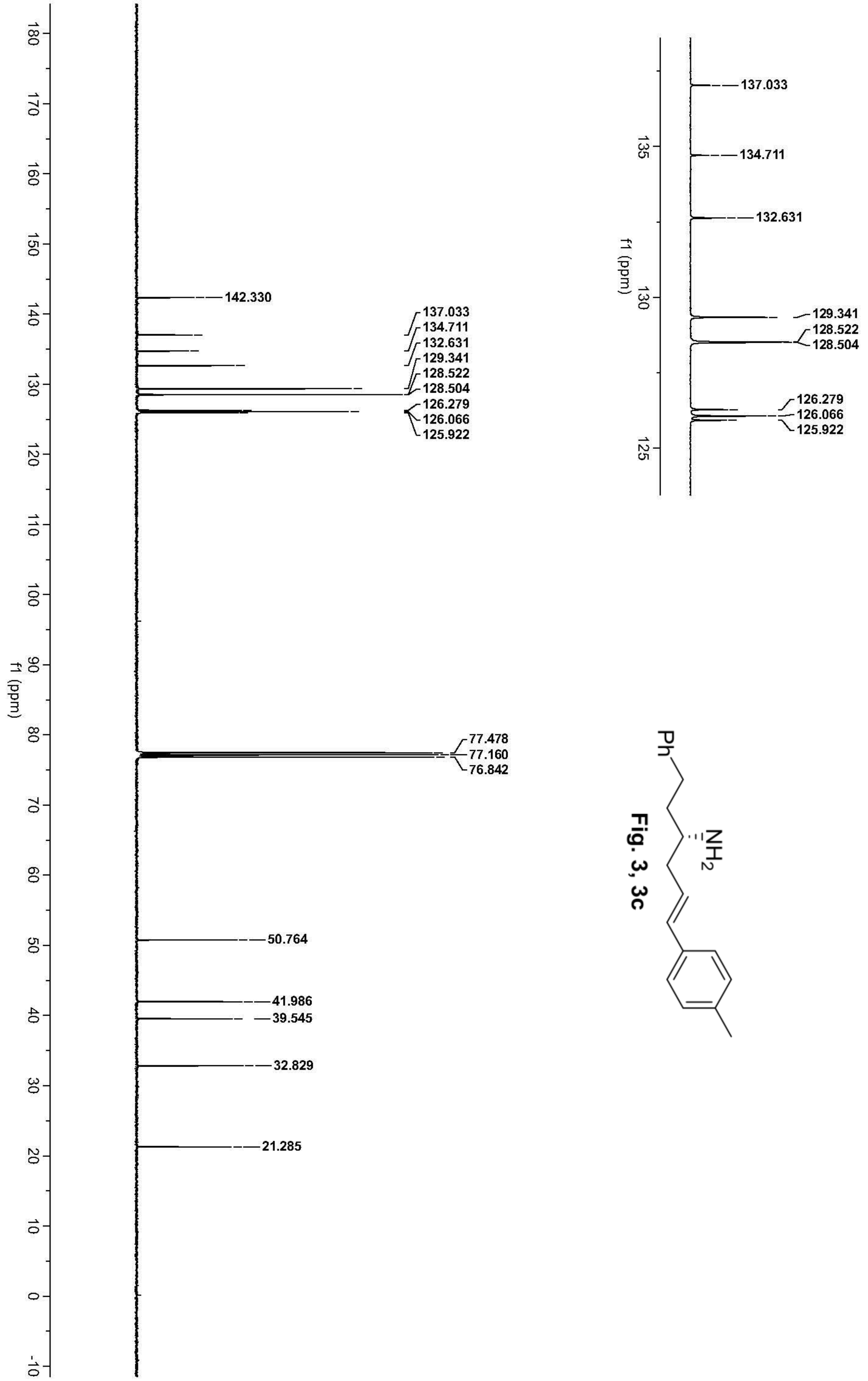
142.330
137.033
134.711
132.631
129.341
128.522
128.504
126.279
126.066
125.922
77.478
77.160
76.842
50.764
41.986
39.545
32.829
21.285
f1 (ppm)
Ph
NH2

Fig. 3, 3c

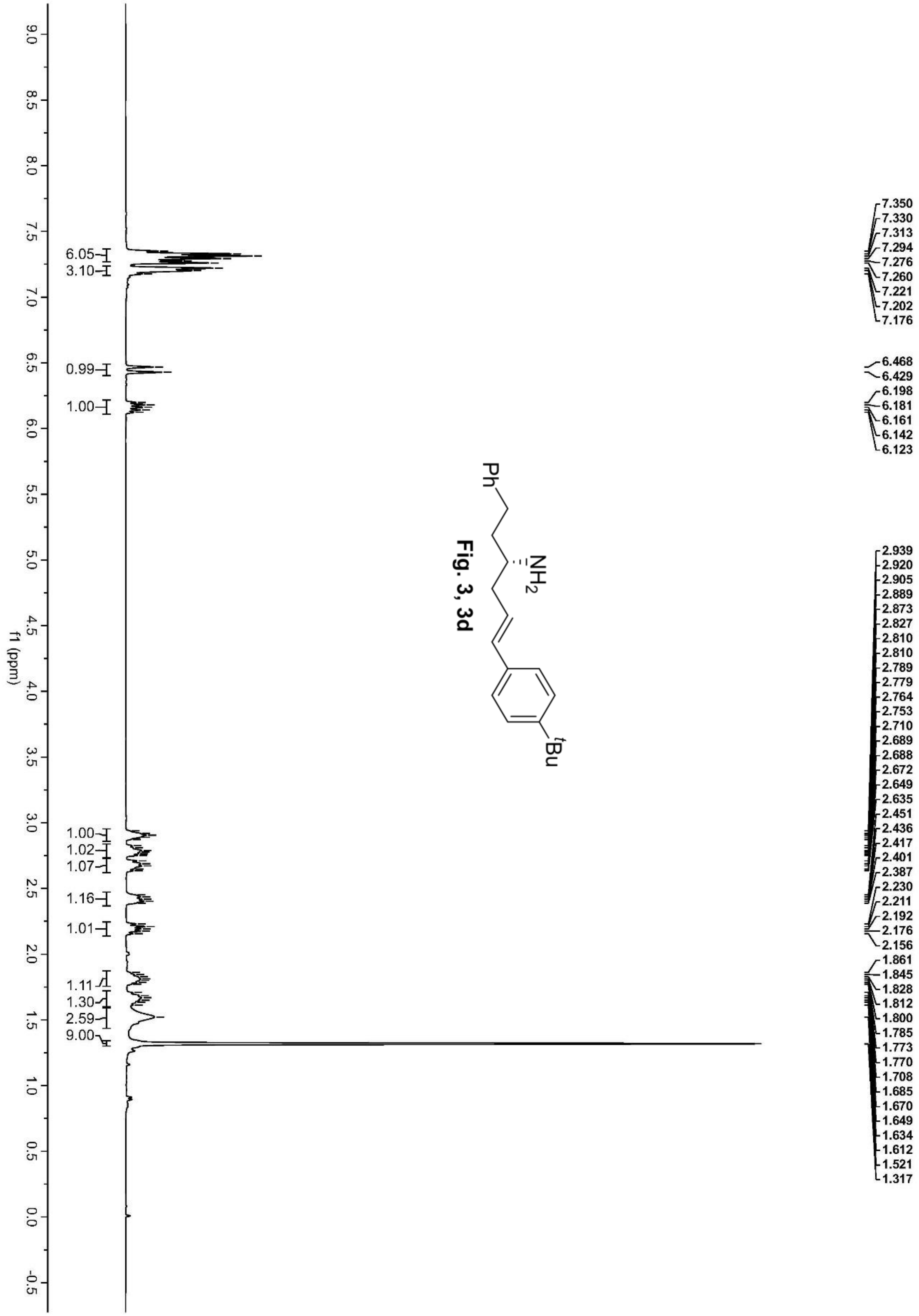

f1 (ppm)
9.0
8.5
8.0
7.5
7.0
6.5
6.0
5.5
5.0
4.5
4.0
3.5
3.0
2.5
2.0
1.5
1.0
0.5
0.0
-0.5
6.05
3.10
0.99
1.00
1.00
1.02
1.07
1.16
1.01
1.11
1.30
2.59
9.00
7.350
7.330
7.313
7.294
7.276
7.260
7.221
7.202
7.176
6.468
6.429
6.198
6.181
6.161
6.142
6.123
2.939
2.920
2.905
2.889
2.873
2.827
2.810
2.810
2.789
2.779
2.764
2.753
2.710
2.689
2.688
2.672
2.649
2.635
2.451
2.436
2.417
2.401
2.387
2.230
2.211
2.192
2.176
2.156
1.861
1.845
1.828
1.812
1.800
1.785
1.773
1.770
1.708
1.685
1.670
1.649
1.634
1.612
1.521
1.317
Ph
NH2
tBu

Fig. 3, 3d

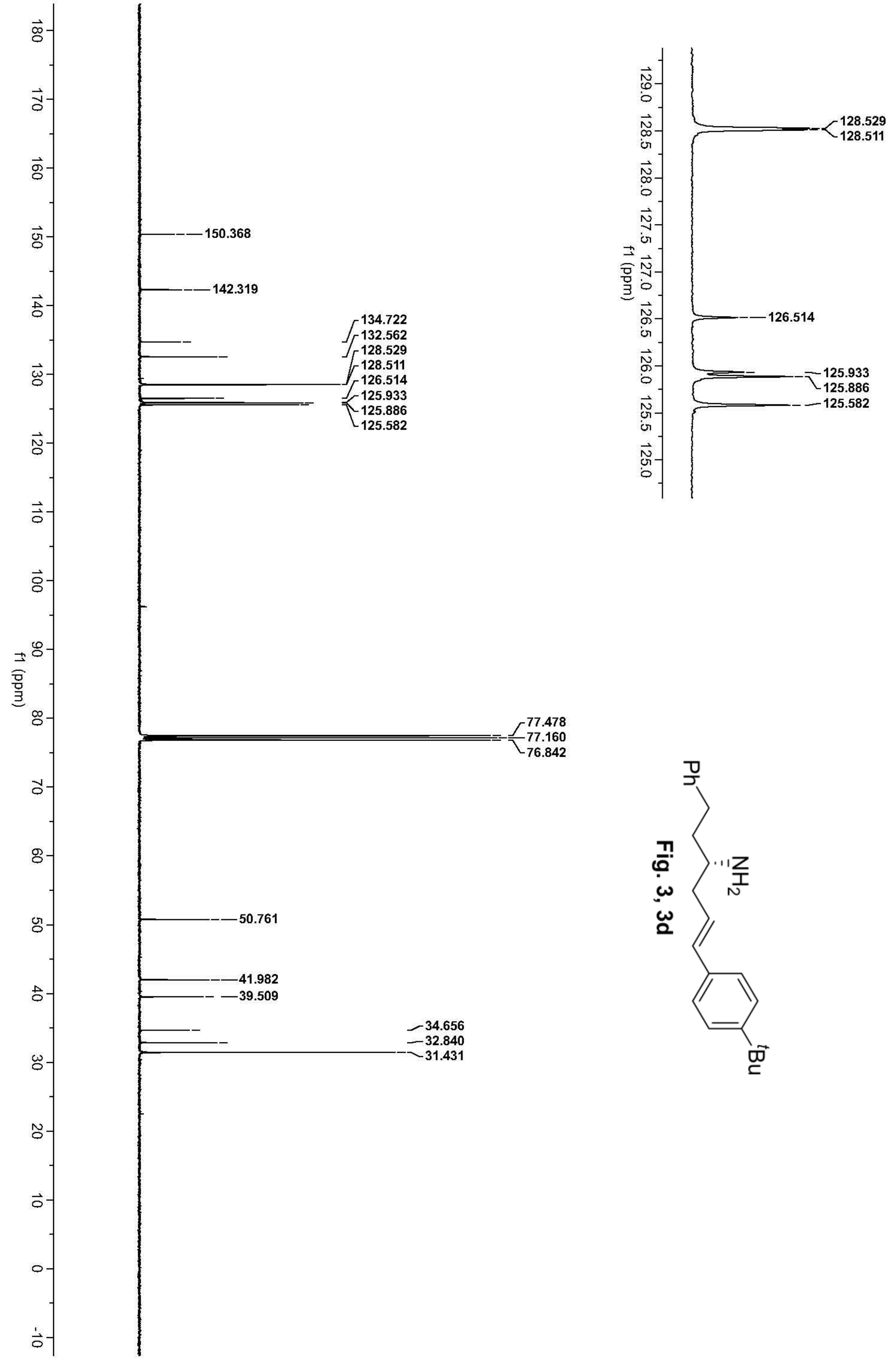
150.368
142.319
134.722
132.562
128.529
128.511
126.514
125.933
125.886
125.582
77.478
77.160
76.842
50.761
41.982
39.509
34.656
32.840
31.431
f1 (ppm)
128.529
128.511
126.514
125.933
125.886
125.582
f1 (ppm)
Ph
NH2
tBu

Fig. 3, 3d

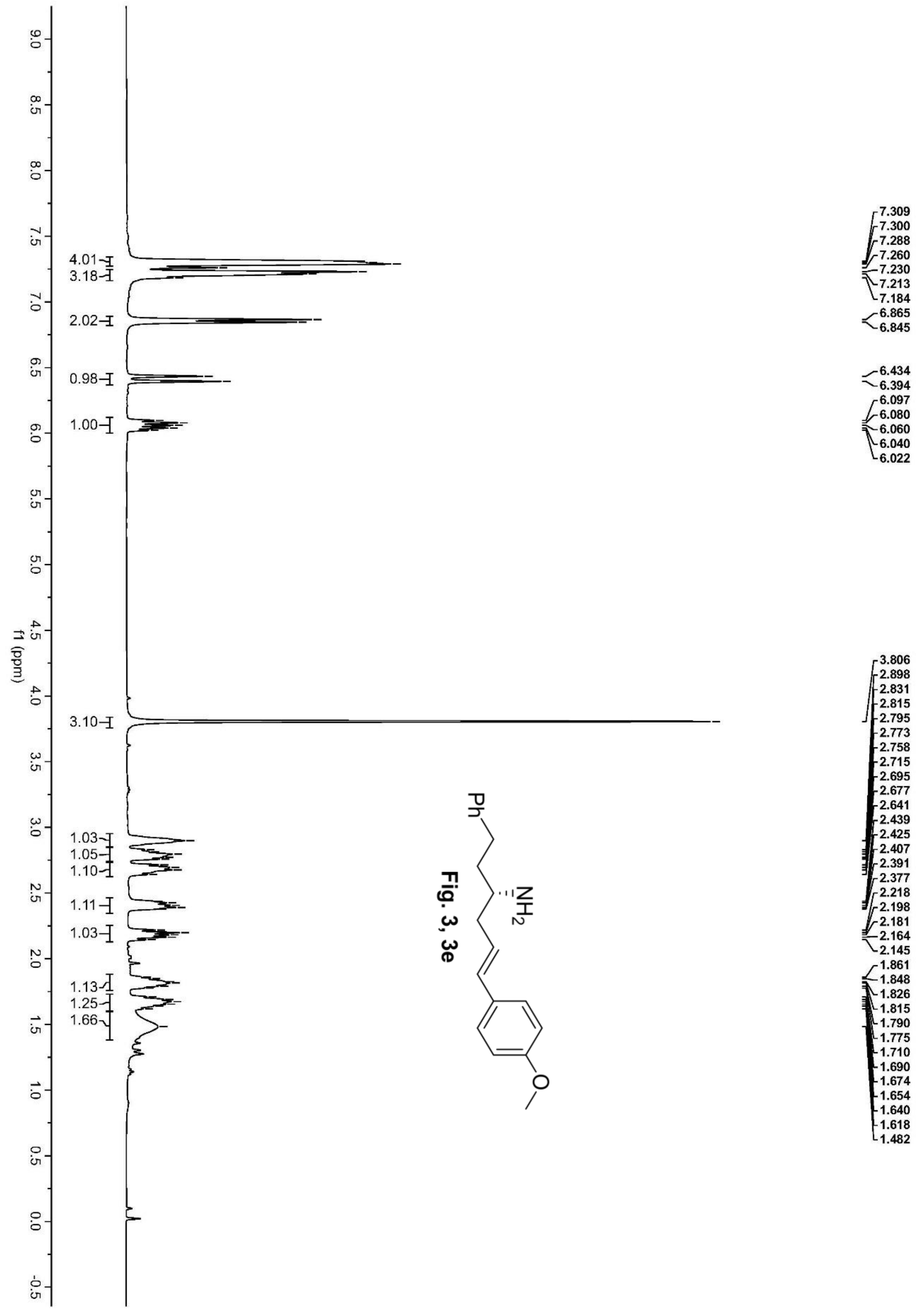

7.309
7.300
7.288
7.260
7.230
7.213
7.184
6.865
6.845
6.434
6.394
6.097
6.080
6.060
6.040
6.022
3.806
2.898
2.831
2.815
2.795
2.773
2.758
2.715
2.695
2.677
2.641
2.439
2.425
2.407
2.391
2.377
2.218
2.198
2.181
2.164
2.145
1.861
1.848
1.826
1.815
1.790
1.775
1.710
1.690
1.674
1.654
1.640
1.618
1.482
4.01
3.18
2.02
0.98
1.00
3.10
1.03
1.05
1.10
1.11
1.03
1.13
1.25
1.66
Ph
NH2
O
f1 (ppm)
9.0
8.5
8.0
7.5
7.0
6.5
6.0
5.5
5.0
4.5
4.0
3.5
3.0
2.5
2.0
1.5
1.0
0.5
0.0
-0.5

Fig. 3, 3e

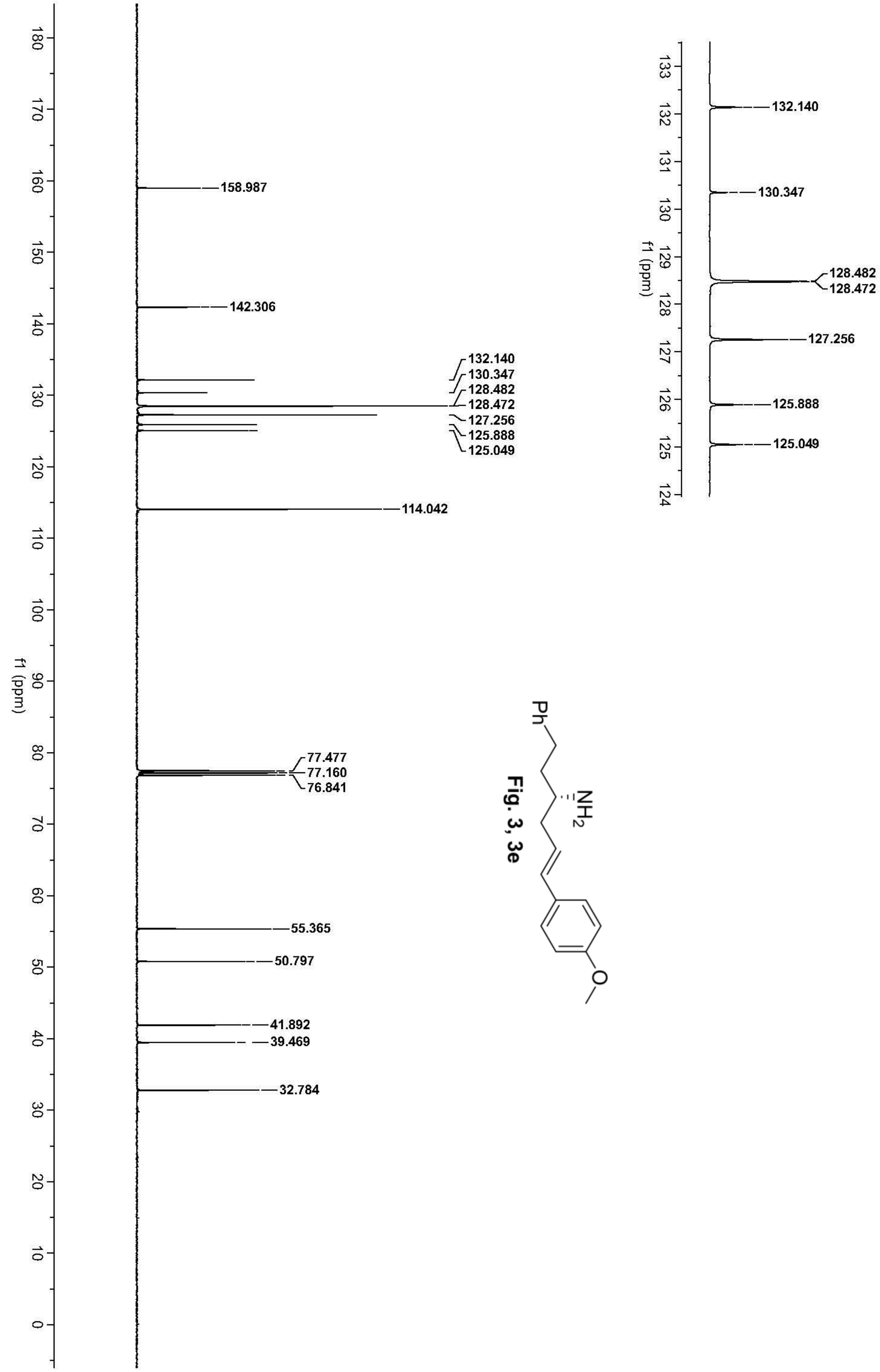
158.987
142.306
132.140
130.347
128.482
128.472
127.256
125.888
125.049
114.042
77.477
77.160
76.841
55.365
50.797
41.892
39.469
32.784
f1 (ppm)
Ph
NH2
Fig. 3, 3e

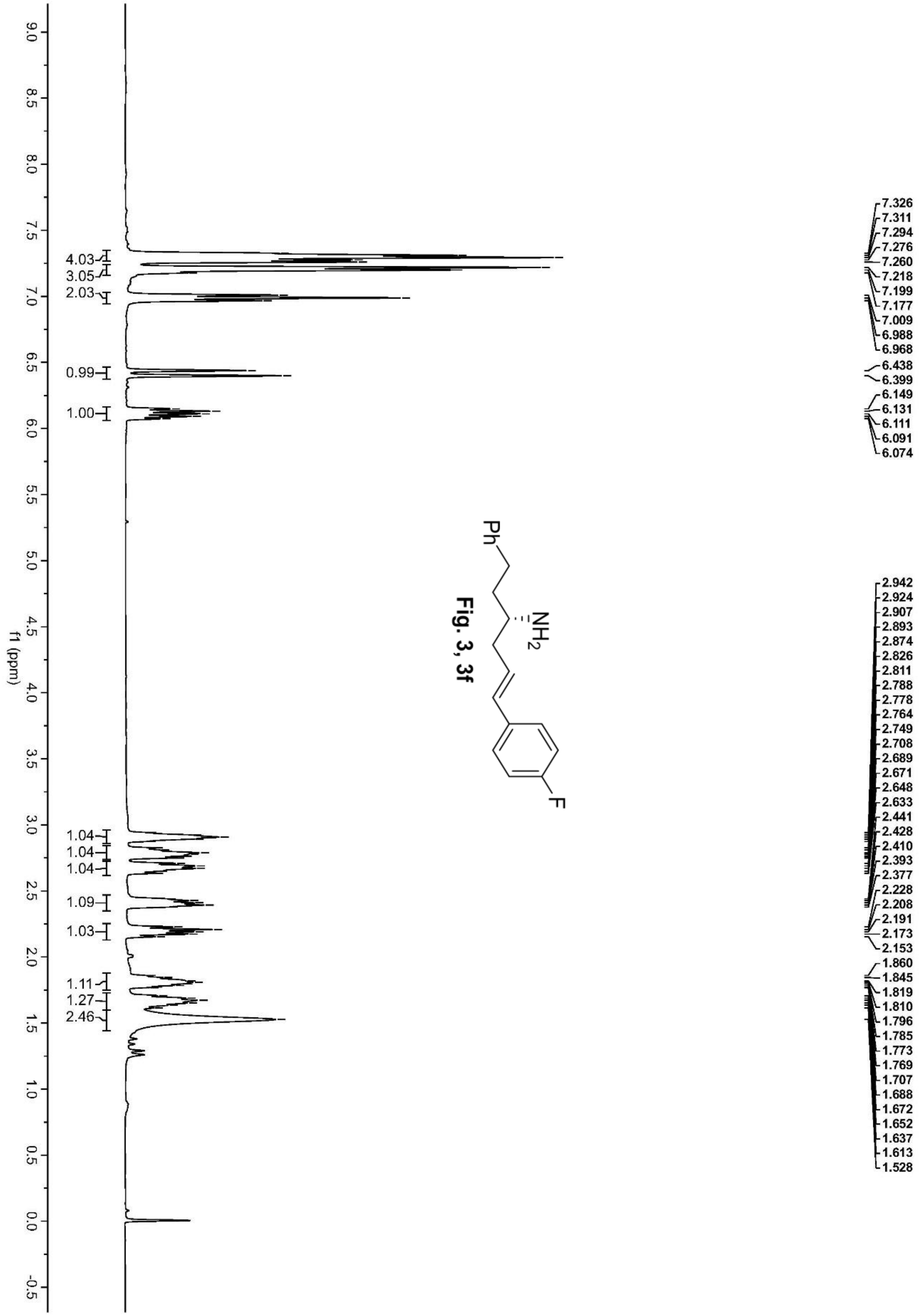
7.326
7.311
7.294
7.276
7.260
7.218
7.199
7.177
7.009
6.988
6.968
6.438
6.399
6.149
6.131
6.111
6.091
6.074
2.942
2.924
2.907
2.893
2.874
2.826
2.811
2.788
2.778
2.764
2.749
2.708
2.689
2.671
2.648
2.633
2.441
2.428
2.410
2.393
2.377
2.228
2.208
2.191
2.173
2.153
1.860
1.845
1.819
1.810
1.796
1.785
1.773
1.769
1.707
1.688
1.672
1.652
1.637
1.613
1.528
4.03
3.05
2.03
0.99
1.00
1.04
1.04
1.04
1.09
1.03
1.11
1.27
2.46
Ph
NH2
F
f1 (ppm)
Fig. 3, 3f

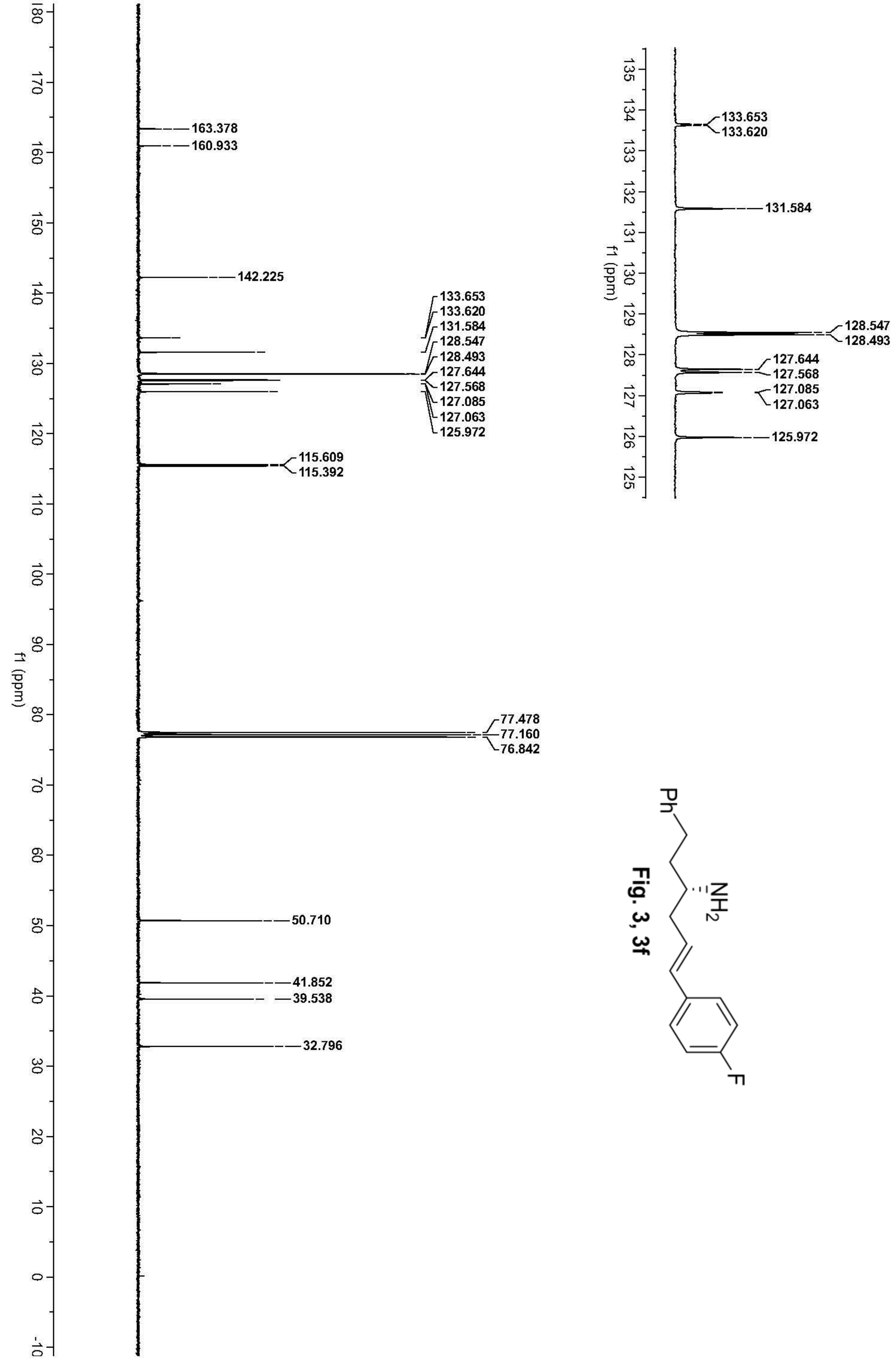
163.378
160.933
142.225
133.653
133.620
131.584
128.547
128.493
127.644
127.568
127.085
127.063
125.972
115.609
115.392
77.478
77.160
76.842
50.710
41.852
39.538
32.796
f1 (ppm)
Ph
NH2
F

Fig. 3, 3f

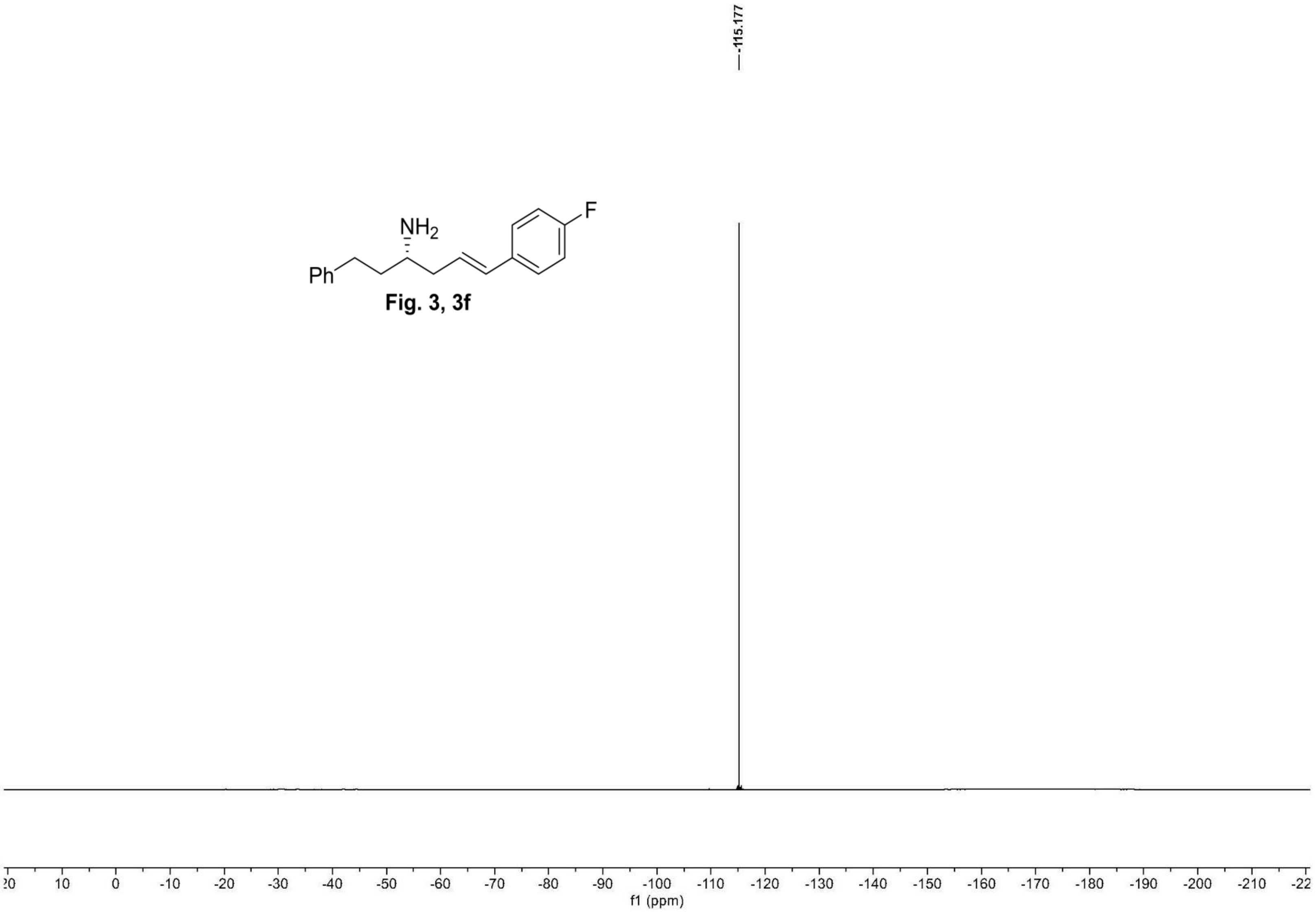
NH2
F
Ph
-115.177
20
10
0
-10
-20
-30
-40
-50
-60
-70
-80
-90
-100
-110
-120
-130
-140
-150
-160
-170
-180
-190
-200
-210
-22
f1 (ppm)

Fig. 3, 3f

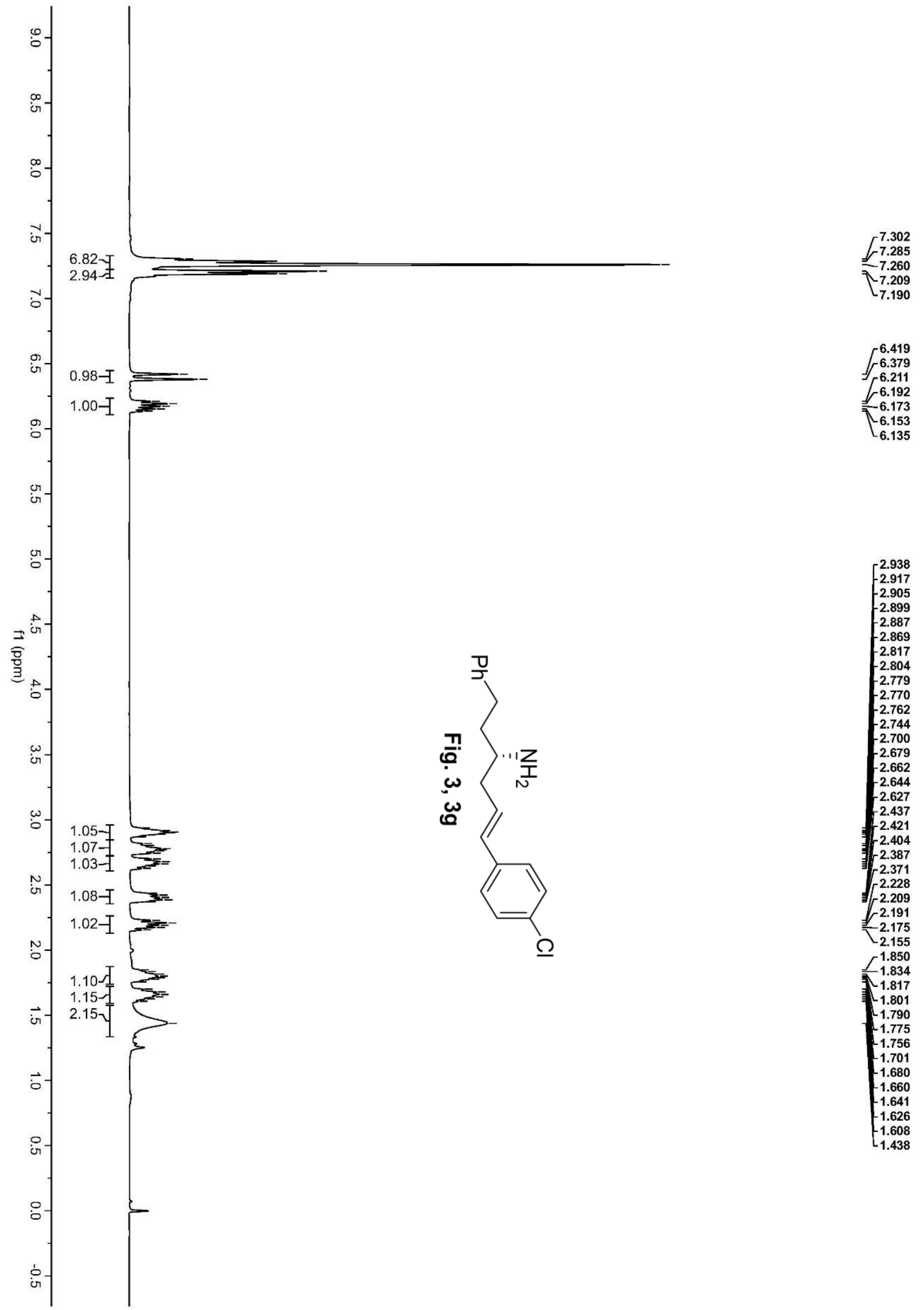

Fig. 3, 3g

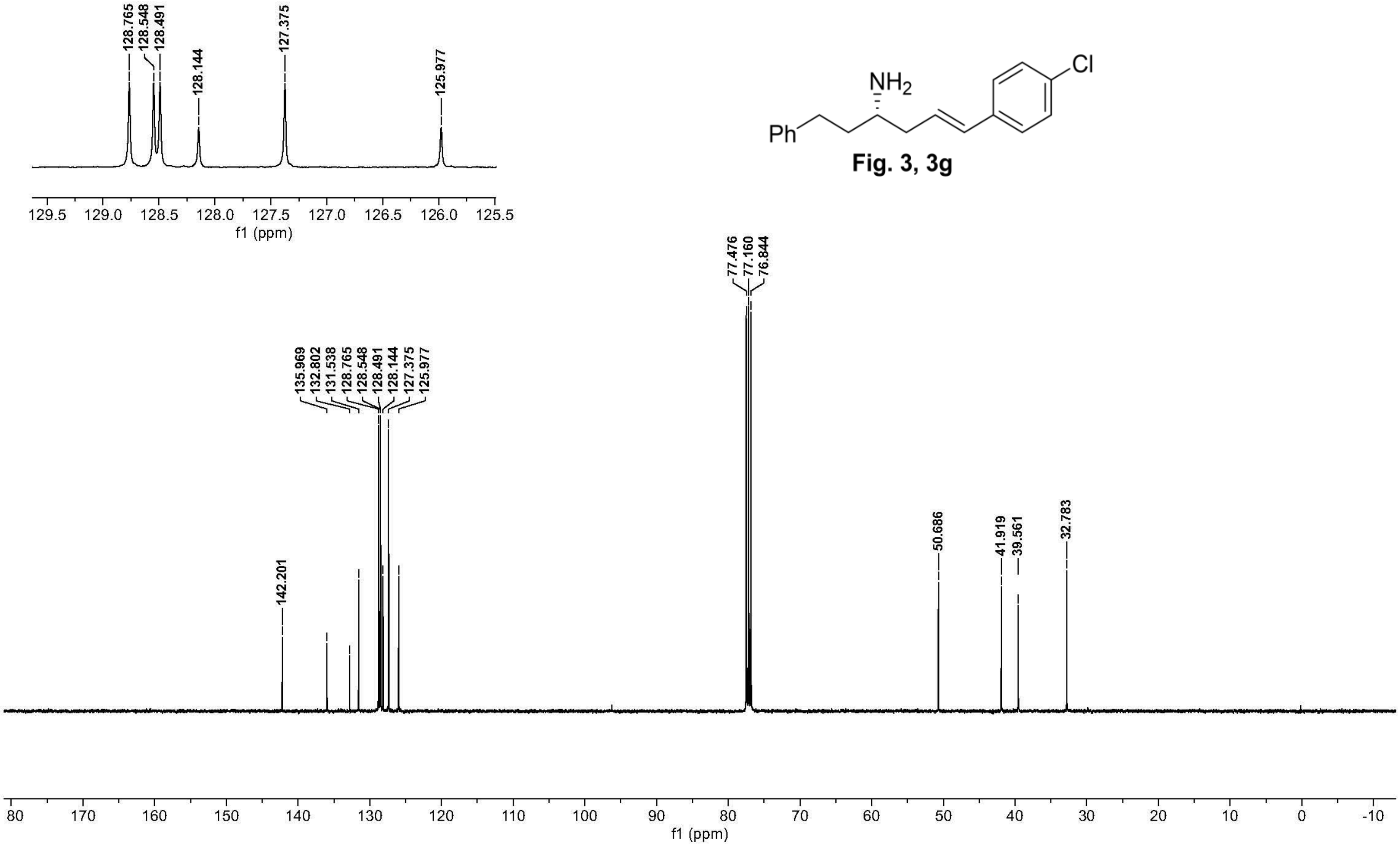
128.765
128.548
128.491
128.144
127.375
125.977
129.5 129.0 128.5 128.0 127.5 127.0 126.5 126.0 125.5
f1 (ppm)
NH2
Cl
Ph
77.476
77.160
76.844
135.969
132.802
131.538
128.765
128.548
128.491
128.144
127.375
125.977
142.201
50.686
41.919
39.561
32.783
80 170 160 150 140 130 120 110 100 90 80 70 60 50 40 30 20 10 0 -10
f1 (ppm)

Fig. 3, 3g

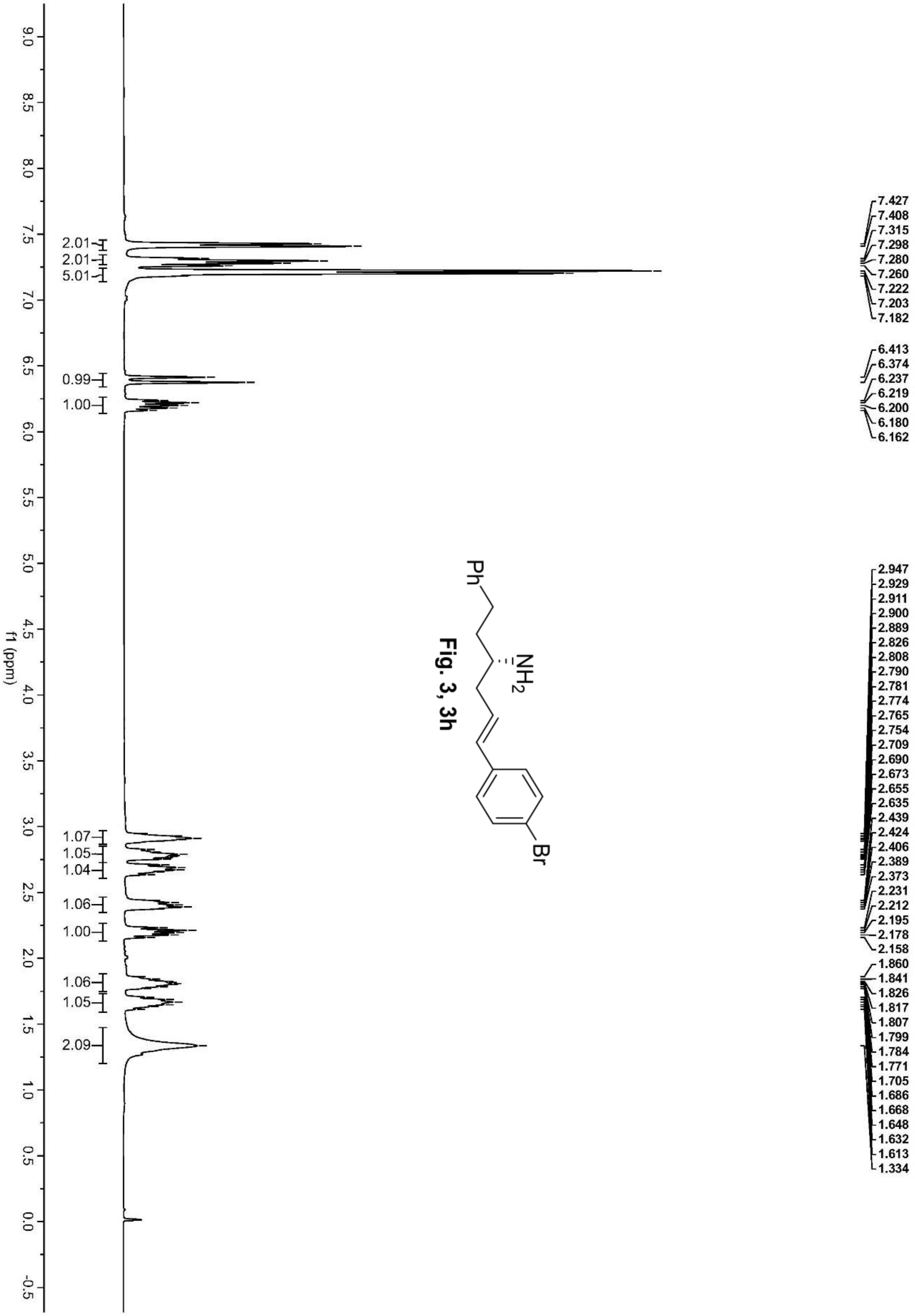

7.427
7.408
7.315
7.298
7.280
7.260
7.222
7.203
7.182
6.413
6.374
6.237
6.219
6.200
6.180
6.162
2.947
2.929
2.911
2.900
2.889
2.826
2.808
2.790
2.781
2.774
2.765
2.754
2.709
2.690
2.673
2.655
2.635
2.439
2.424
2.406
2.389
2.373
2.231
2.212
2.195
2.178
2.158
1.860
1.841
1.826
1.817
1.807
1.799
1.784
1.771
1.705
1.686
1.668
1.648
1.632
1.613
1.334
2.01
2.01
5.01
0.99
1.00
1.07
1.05
1.04
1.06
1.00
1.06
1.05
2.09
Ph
NH2
Br
9.0
8.5
8.0
7.5
7.0
6.5
6.0
5.5
5.0
4.5
4.0
3.5
3.0
2.5
2.0
1.5
1.0
0.5
0.0
-0.5
f1 (ppm)

Fig. 3, 3h

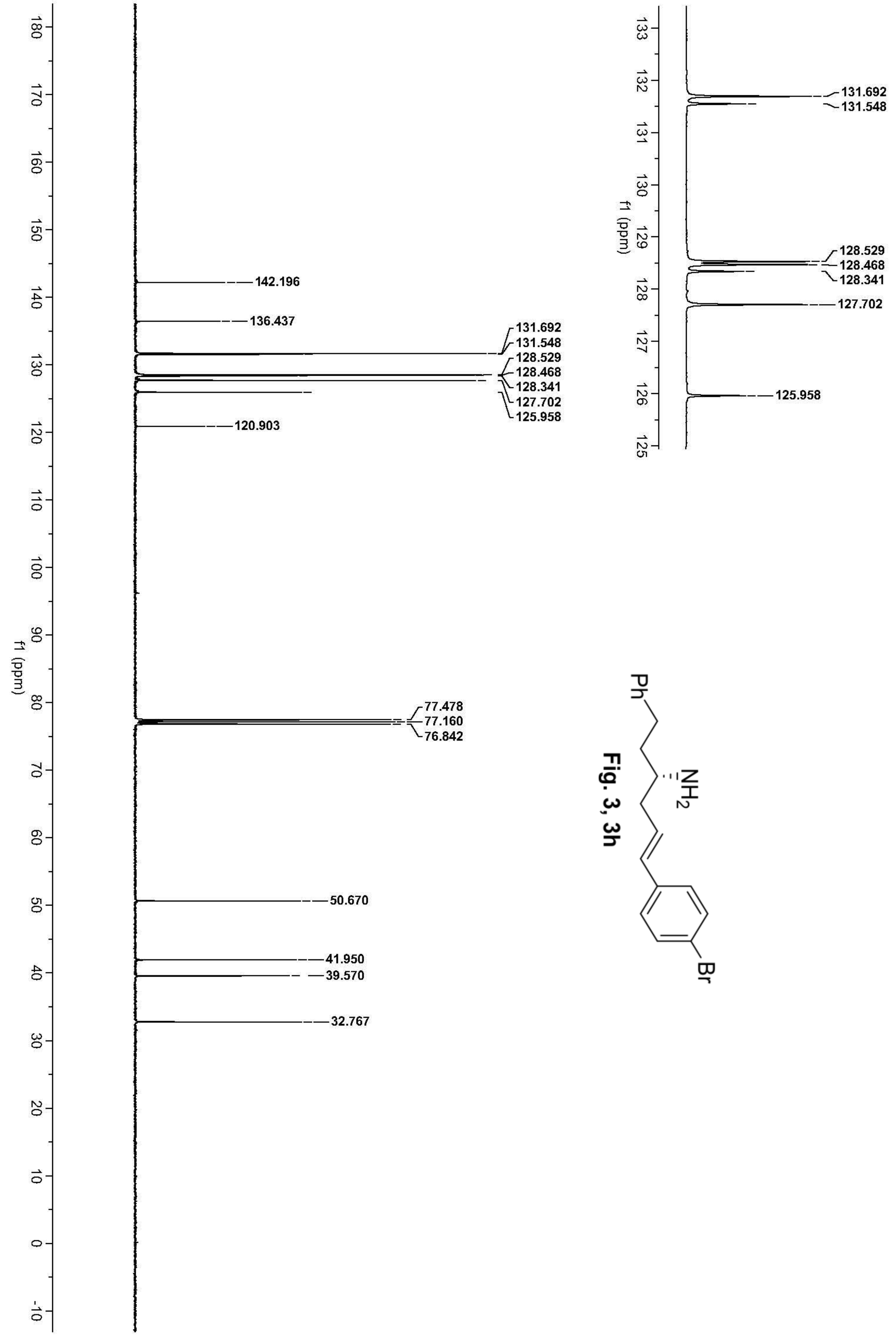

142.196
136.437
131.692
131.548
128.529
128.468
128.341
127.702
125.958
120.903
77.478
77.160
76.842
50.670
41.950
39.570
32.767
f1 (ppm)
Ph
NH2
Br

Fig. 3, 3h

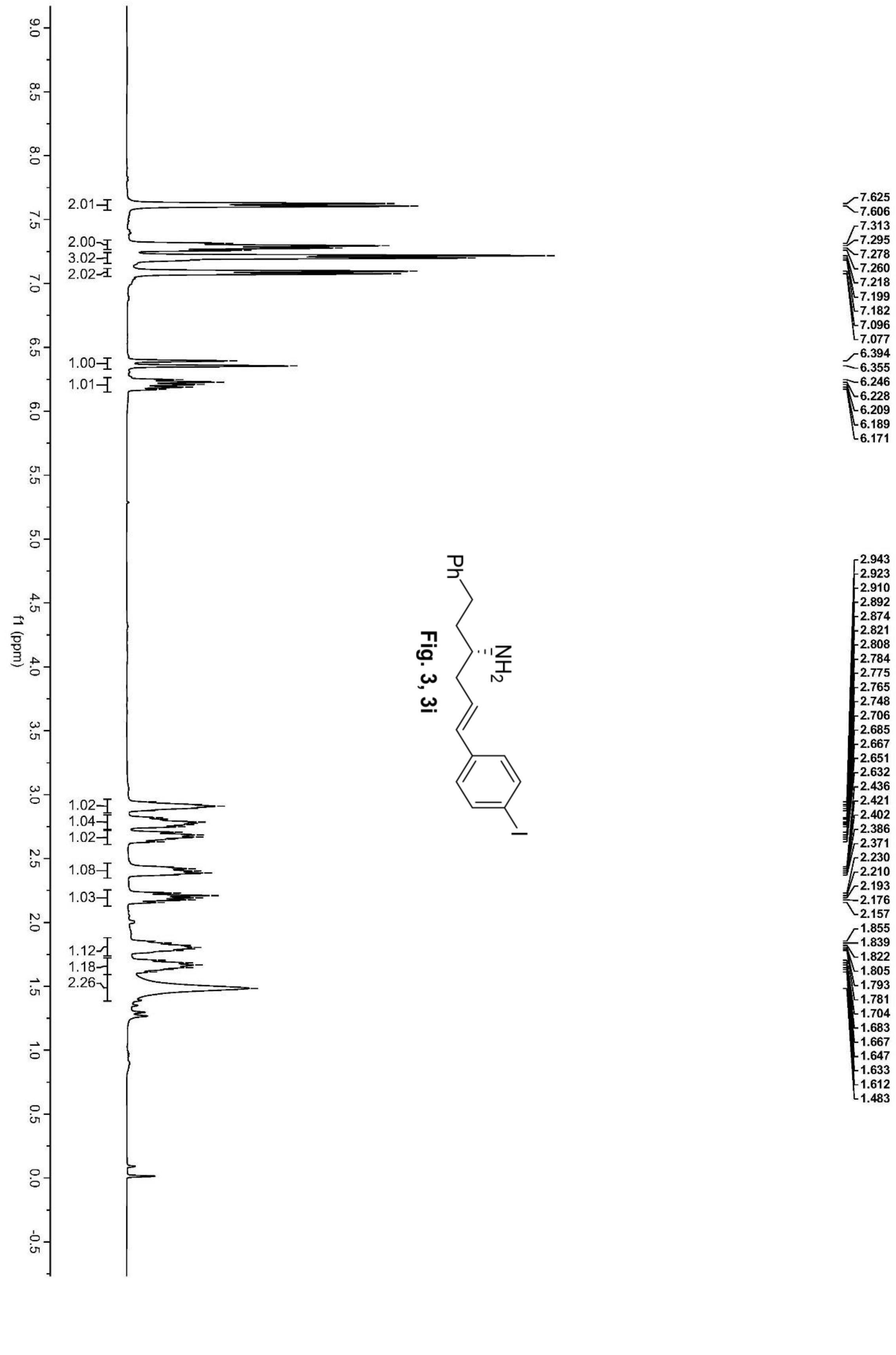
7.625
7.606
7.313
7.295
7.278
7.260
7.218
7.199
7.182
7.096
7.077
6.394
6.355
6.246
6.228
6.209
6.189
6.171
2.943
2.923
2.910
2.892
2.874
2.821
2.808
2.784
2.775
2.765
2.748
2.706
2.685
2.667
2.651
2.632
2.436
2.421
2.402
2.386
2.371
2.230
2.210
2.193
2.176
2.157
1.855
1.839
1.822
1.805
1.793
1.781
1.704
1.683
1.667
1.647
1.633
1.612
1.483
Ph
NH2
I
2.01
2.00
3.02
2.02
1.00
1.01
1.02
1.04
1.02
1.08
1.03
1.12
1.18
2.26
f1 (ppm)

Fig. 3, 3i

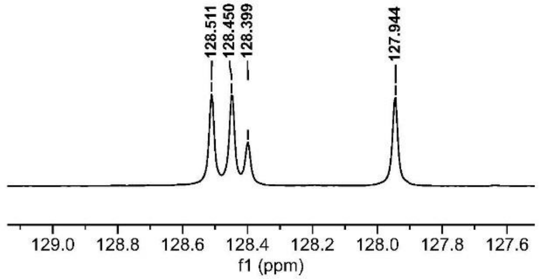
128.511
128.450
128.399
127.944
129.0
128.8
128.6
128.4
128.2
128.0
127.8
127.6
f1 (ppm)

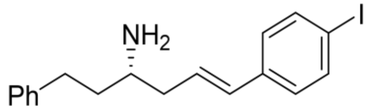
NH2
Ph
I

Fig. 3, 3i

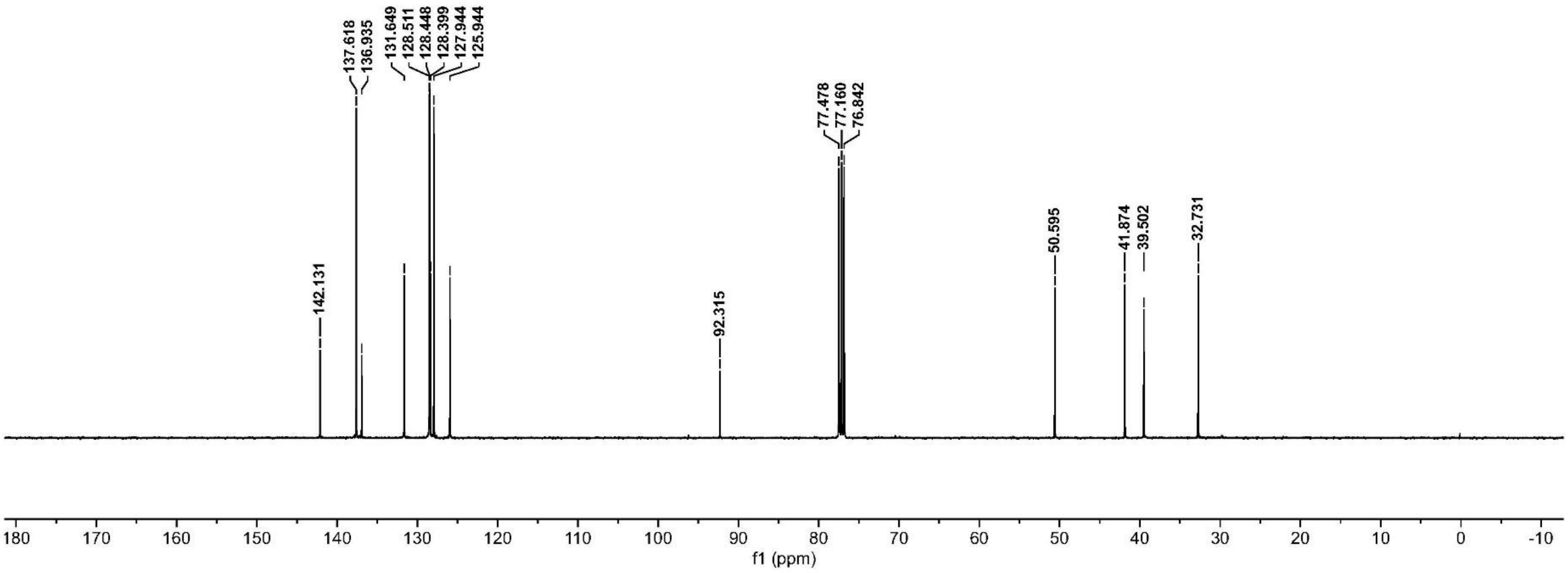
142.131
137.618
136.935
131.649
128.511
128.448
128.399
127.944
125.944
92.315
77.478
77.160
76.842
50.595
41.874
39.502
32.731
180
170
160
150
140
130
120
110
100
90
80
70
60
50
40
30
20
10
0
-10
f1 (ppm)

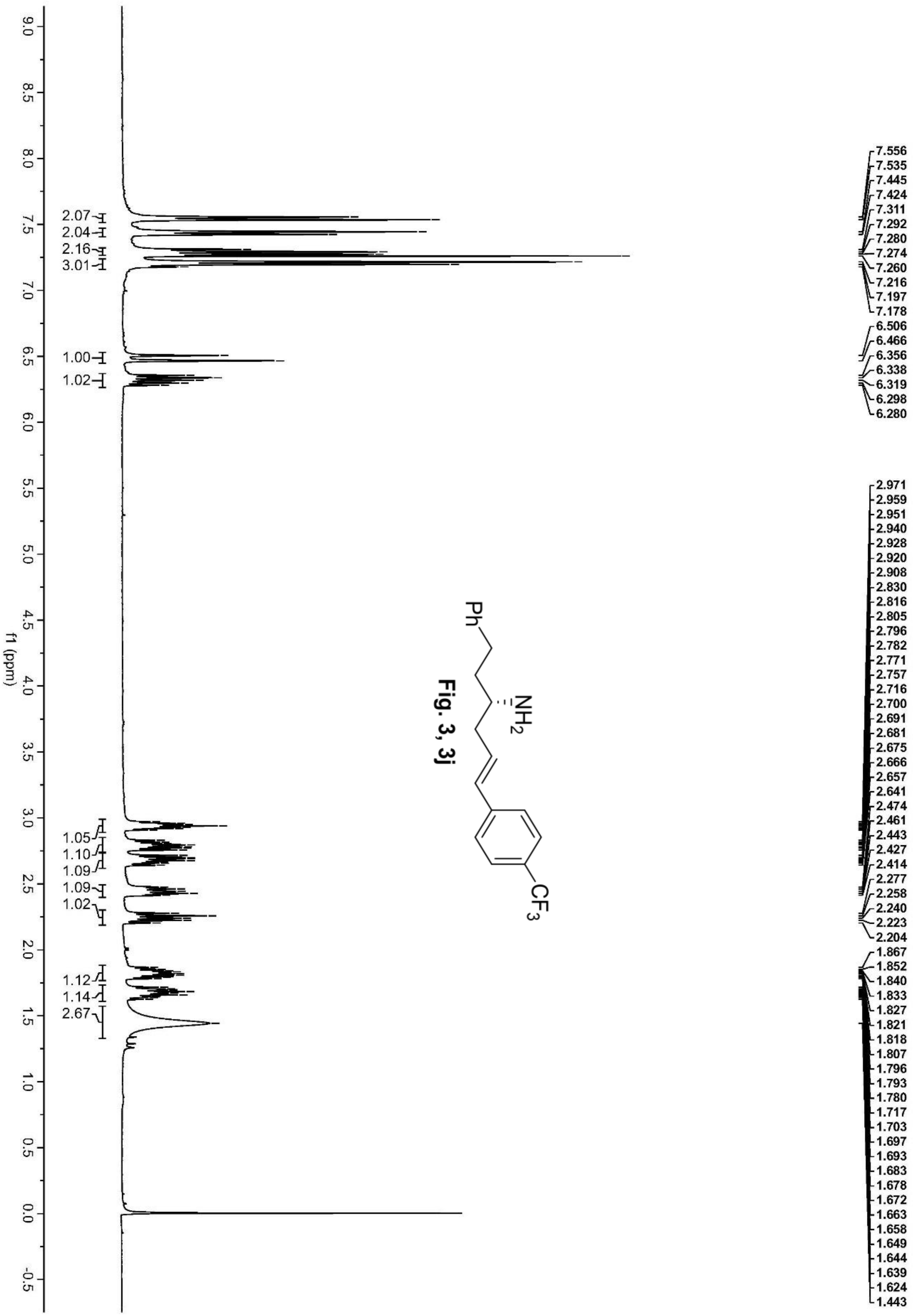
f1 (ppm)
9.0
8.5
8.0
7.5
7.0
6.5
6.0
5.5
5.0
4.5
4.0
3.5
3.0
2.5
2.0
1.5
1.0
0.5
0.0
-0.5
2.07
2.04
2.16
3.01
1.00
1.02
1.05
1.10
1.09
1.09
1.02
1.12
1.14
2.67
Ph
NH2
CF3
7.556
7.535
7.445
7.424
7.311
7.292
7.280
7.274
7.260
7.216
7.197
7.178
6.506
6.466
6.356
6.338
6.319
6.298
6.280
2.971
2.959
2.951
2.940
2.928
2.920
2.908
2.830
2.816
2.805
2.796
2.782
2.771
2.757
2.716
2.700
2.691
2.681
2.675
2.666
2.657
2.641
2.474
2.461
2.443
2.427
2.414
2.277
2.258
2.240
2.223
2.204
1.867
1.852
1.840
1.833
1.827
1.821
1.818
1.807
1.796
1.793
1.780
1.717
1.703
1.697
1.693
1.683
1.678
1.672
1.663
1.658
1.649
1.644
1.639
1.624
1.443
Fig. 3, 3j

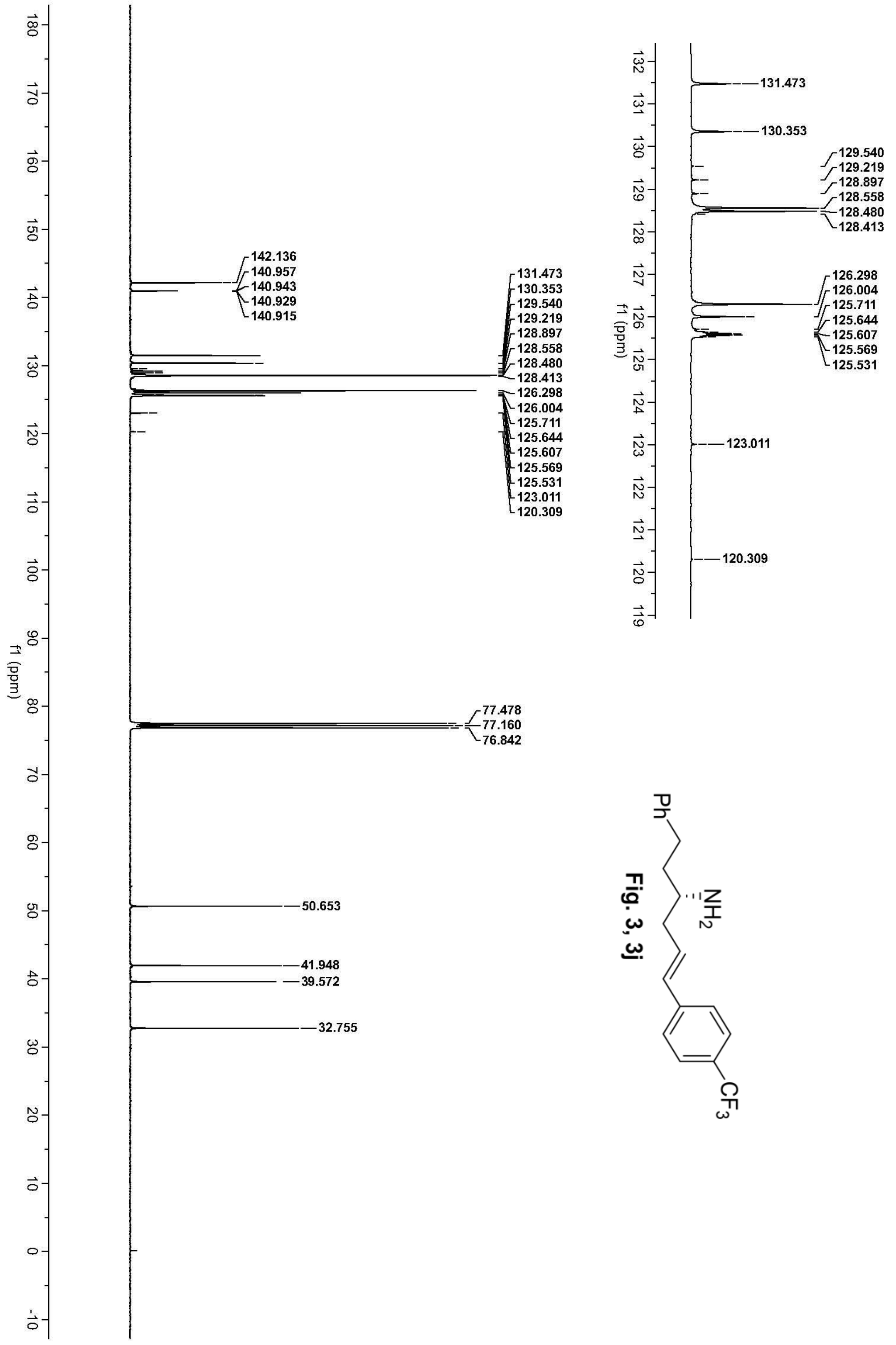
f1 (ppm)
142.136
140.957
140.943
140.929
140.915
131.473
130.353
129.540
129.219
128.897
128.558
128.480
128.413
126.298
126.004
125.711
125.644
125.607
125.569
125.531
123.011
120.309
77.478
77.160
76.842
50.653
41.948
39.572
32.755
Ph
NH2
CF3

Fig. 3, 3j

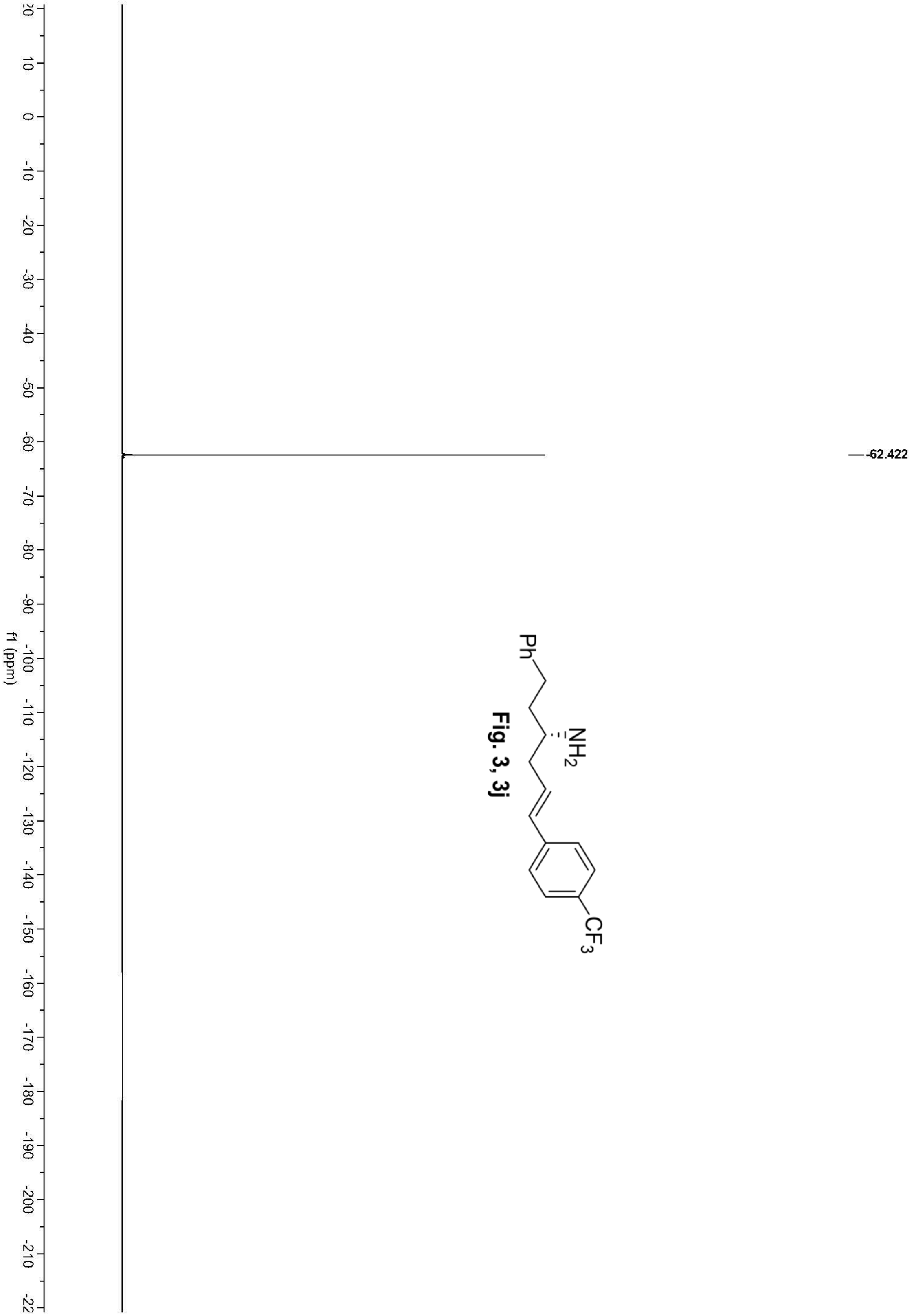
-62.422
Ph
NH2
CF3
f1 (ppm)
10
0
-10
-20
-30
-40
-50
-60
-70
-80
-90
-100
-110
-120
-130
-140
-150
-160
-170
-180
-190
-200
-210

Fig. 3, 3j

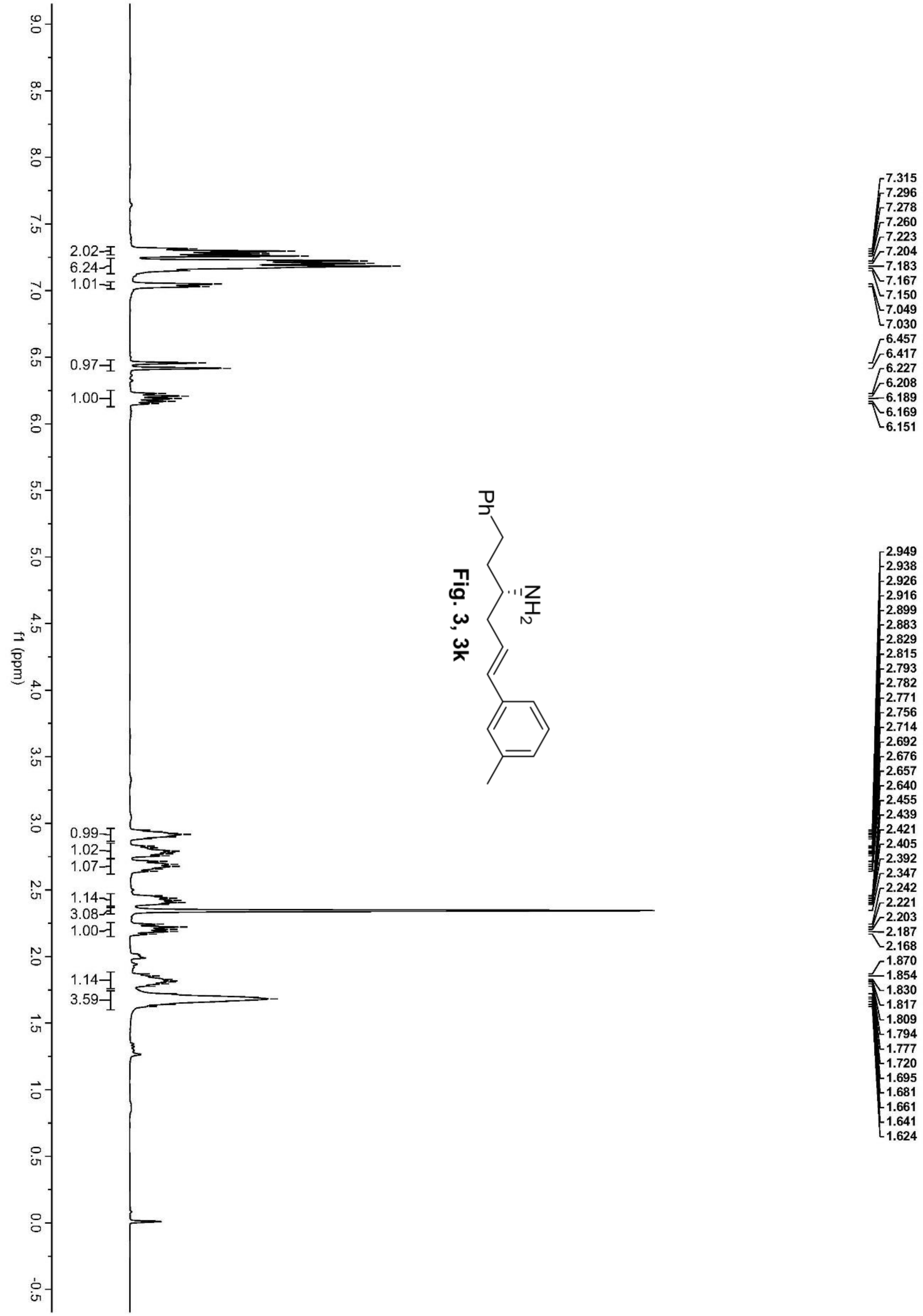

f1 (ppm)
9.0
8.5
8.0
7.5
7.0
6.5
6.0
5.5
5.0
4.5
4.0
3.5
3.0
2.5
2.0
1.5
1.0
0.5
0.0
-0.5
2.02
6.24
1.01
0.97
1.00
0.99
1.02
1.07
1.14
3.08
1.00
1.14
3.59
7.315
7.296
7.278
7.260
7.223
7.204
7.183
7.167
7.150
7.049
7.030
6.457
6.417
6.227
6.208
6.189
6.169
6.151
2.949
2.938
2.926
2.916
2.899
2.883
2.829
2.815
2.793
2.782
2.771
2.756
2.714
2.692
2.676
2.657
2.640
2.455
2.439
2.421
2.405
2.392
2.347
2.242
2.221
2.203
2.187
2.168
1.870
1.854
1.830
1.817
1.809
1.794
1.777
1.720
1.695
1.681
1.661
1.641
1.624
Ph
NH_2

Fig. 3, 3k

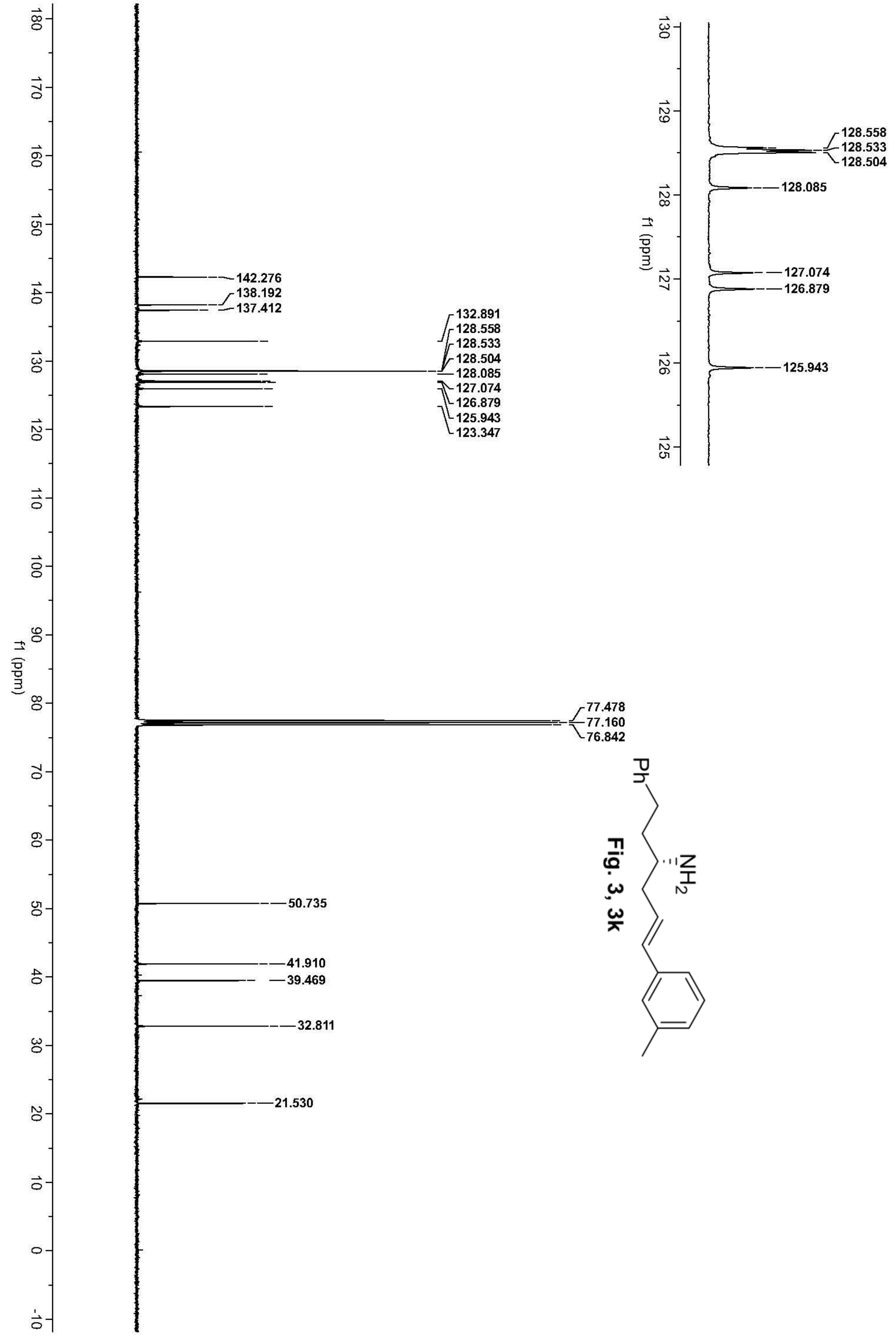

142.276
138.192
137.412
132.891
128.558
128.533
128.504
128.085
127.074
126.879
125.943
123.347
77.478
77.160
76.842
50.735
41.910
39.469
32.811
21.530
f1 (ppm)
Ph
NH2

Fig. 3, 3k

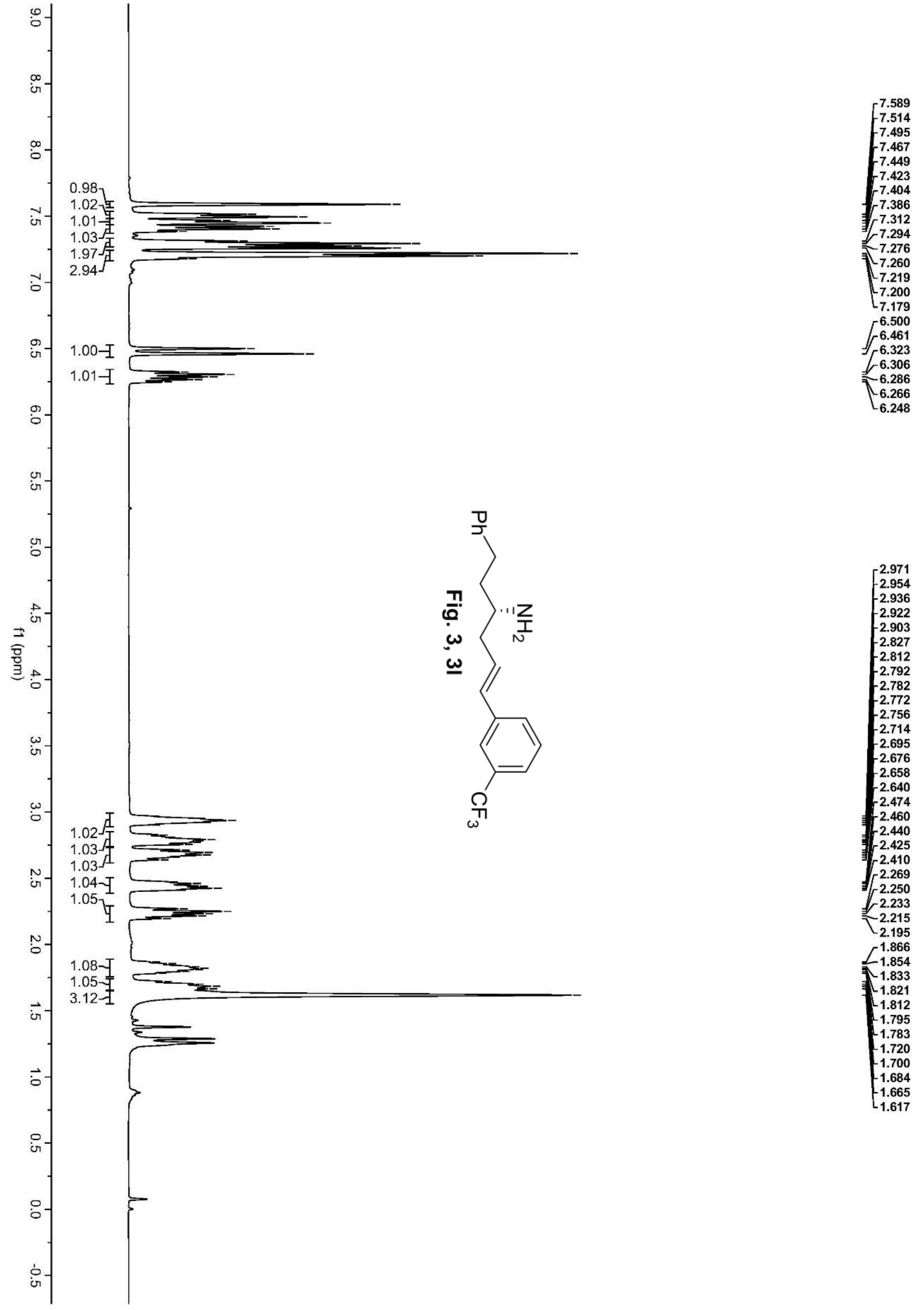
Ph
NH2
CF3
Fig. 3, 3l
f1 (ppm)

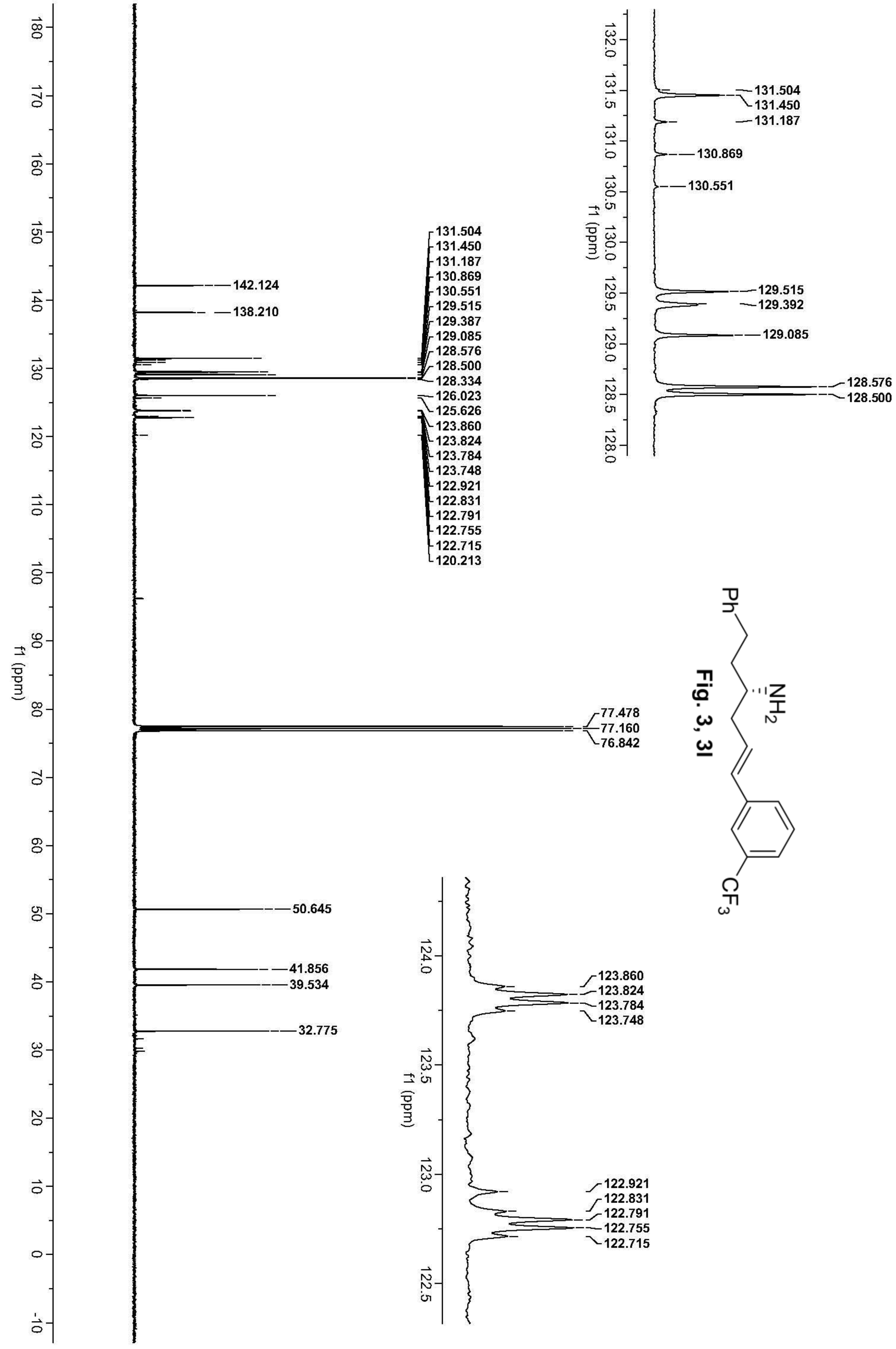

142.124
138.210
131.504
131.450
131.187
130.869
130.551
129.515
129.387
129.085
128.576
128.500
128.334
126.023
125.626
123.860
123.824
123.784
123.748
122.921
122.831
122.791
122.755
122.715
120.213
77.478
77.160
76.842
50.645
41.856
39.534
32.775
129.392
f1 (ppm)
Ph
NH2
CF3

Fig. 3, 3l

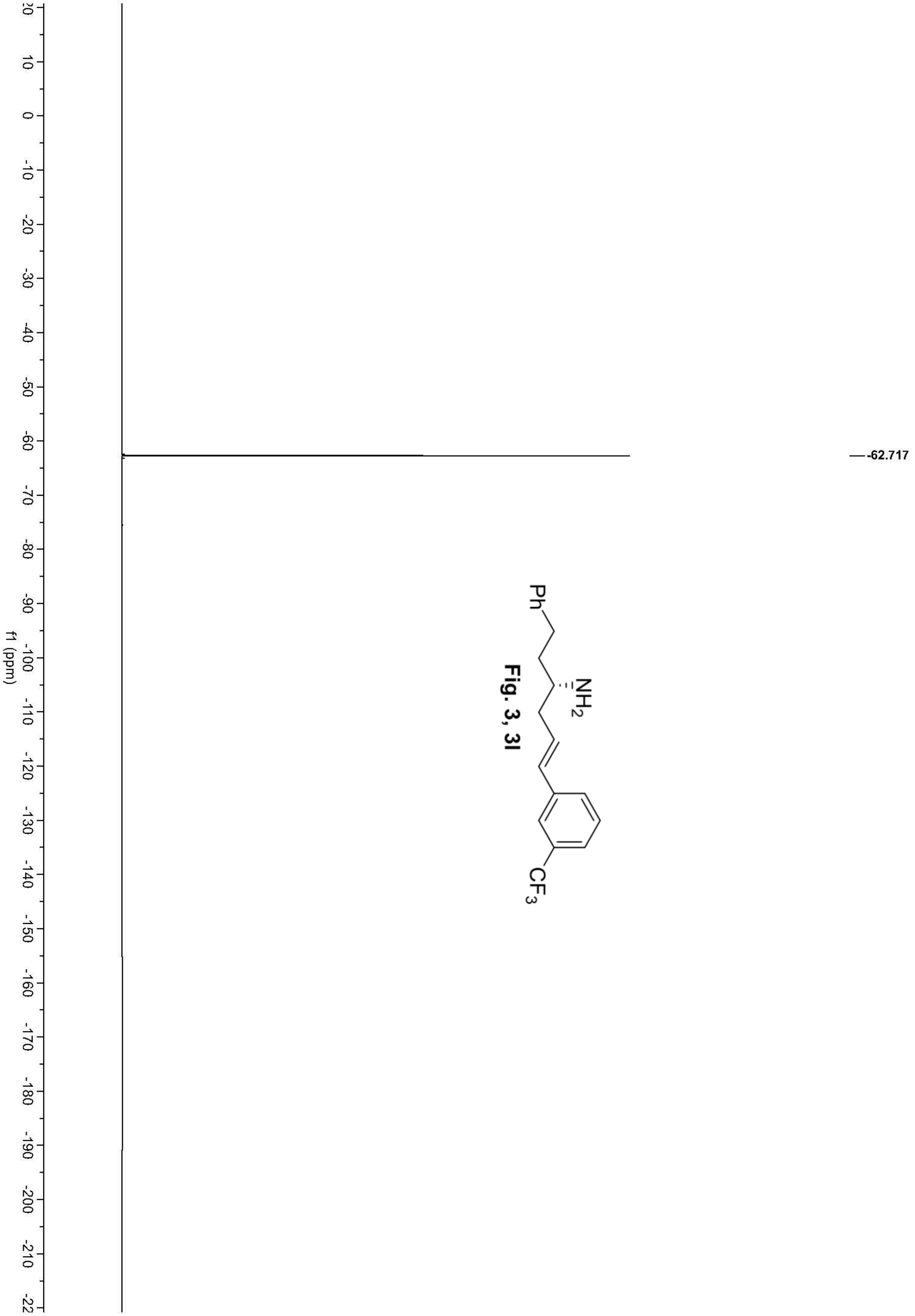

Fig. 3, 3l

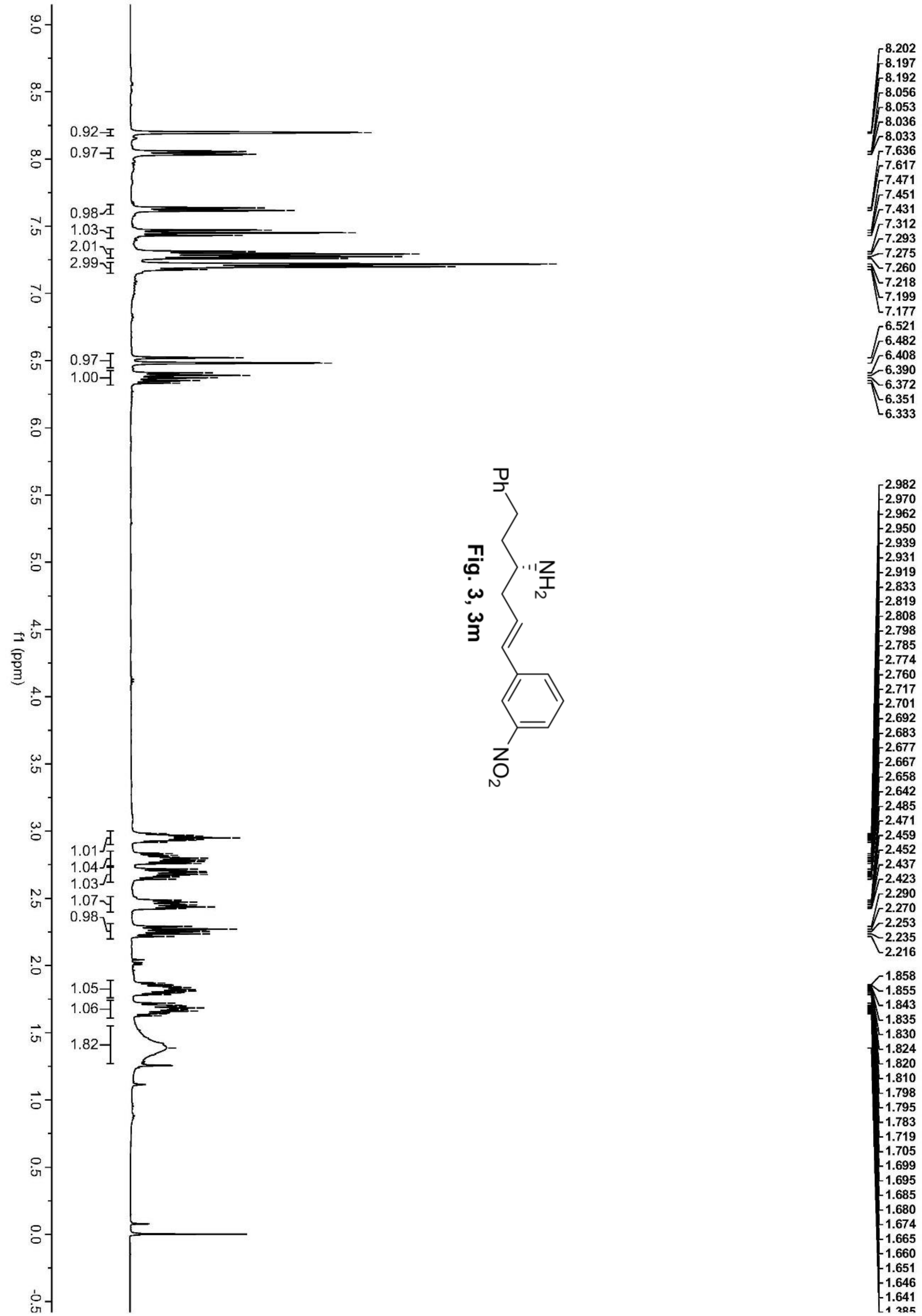

Fig. 3, 3m

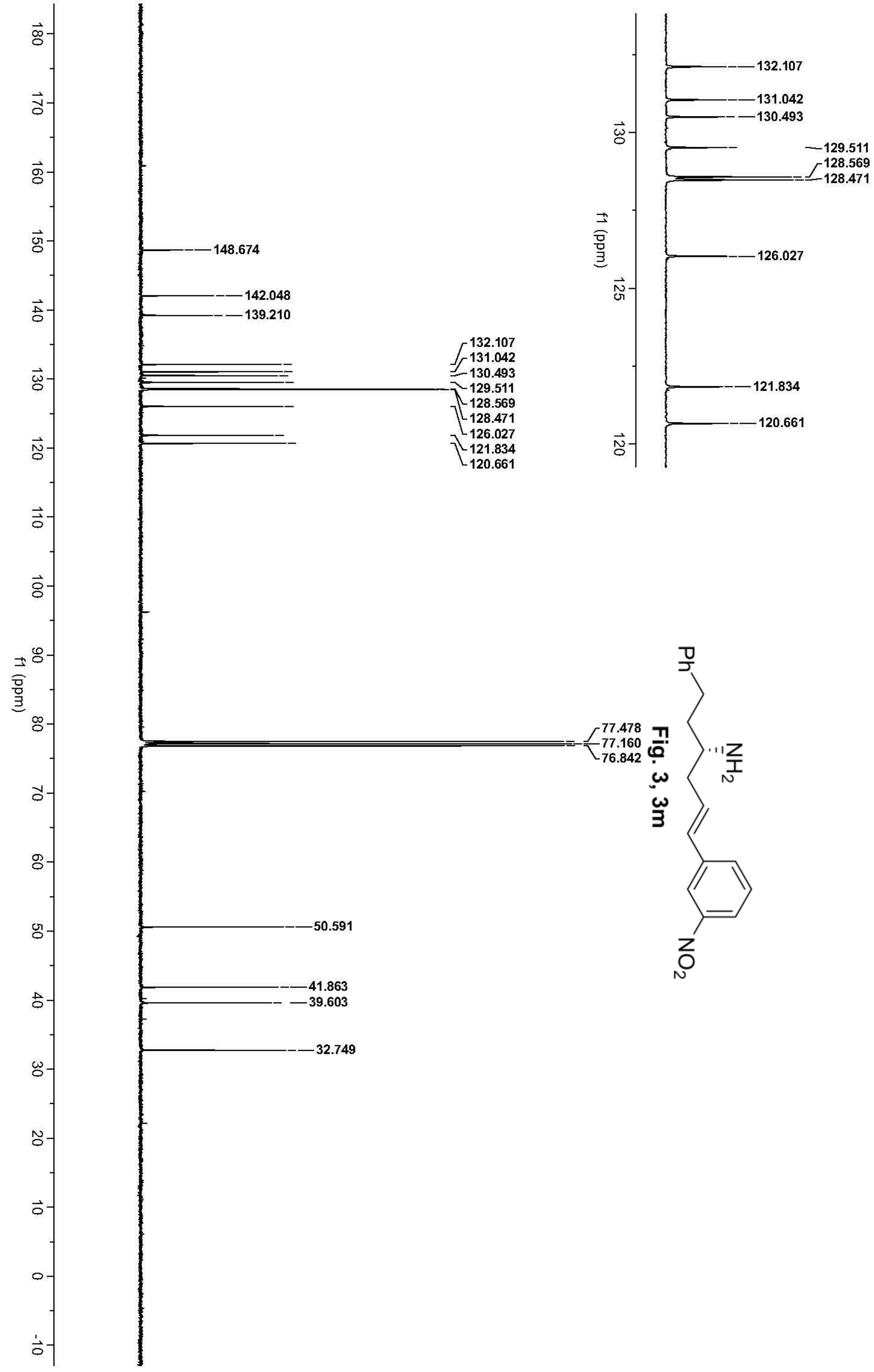
148.674
142.048
139.210
132.107
131.042
130.493
129.511
128.569
128.471
126.027
121.834
120.661
77.478
77.160
76.842
50.591
41.863
39.603
32.749
f1 (ppm)
Ph
NH2
NO2

Fig. 3, 3m

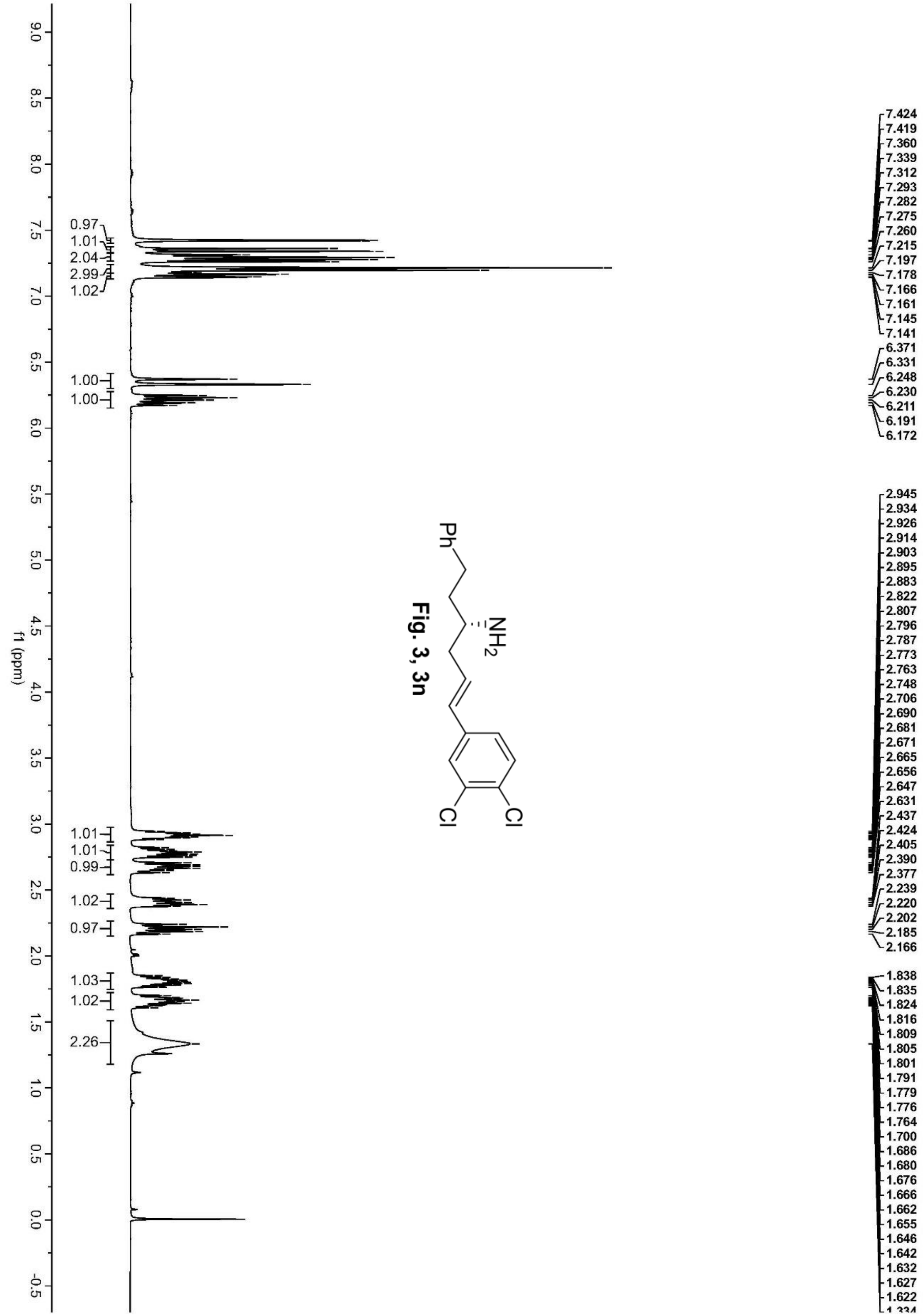
f1 (ppm)
9.0
8.5
8.0
7.5
7.0
6.5
6.0
5.5
5.0
4.5
4.0
3.5
3.0
2.5
2.0
1.5
1.0
0.5
0.0
-0.5
0.97
1.01
2.04
2.99
1.02
1.00
1.00
1.01
1.01
0.99
1.02
0.97
1.03
1.02
2.26
Ph
NH2
Cl
Cl
7.424
7.419
7.360
7.339
7.312
7.293
7.282
7.275
7.260
7.215
7.197
7.178
7.166
7.161
7.145
7.141
6.371
6.331
6.248
6.230
6.211
6.191
6.172
2.945
2.934
2.926
2.914
2.903
2.895
2.883
2.822
2.807
2.796
2.787
2.773
2.763
2.748
2.706
2.690
2.681
2.671
2.665
2.656
2.647
2.631
2.437
2.424
2.405
2.390
2.377
2.239
2.220
2.202
2.185
2.166
1.838
1.835
1.824
1.816
1.809
1.805
1.801
1.791
1.779
1.776
1.764
1.700
1.686
1.680
1.676
1.666
1.662
1.655
1.646
1.642
1.632
1.627
1.622

Fig. 3, 3n

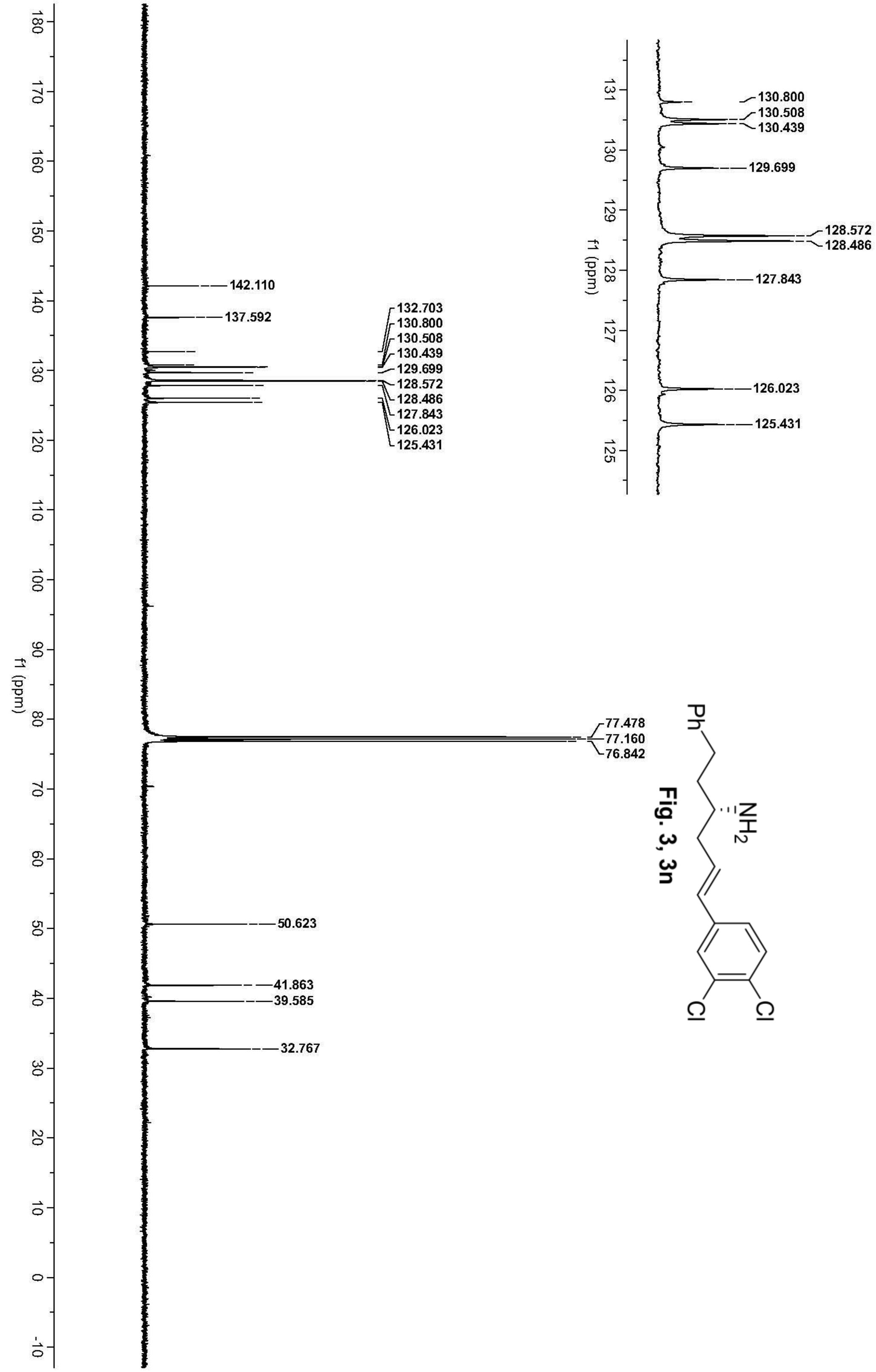
142.110
137.592
132.703
130.800
130.508
130.439
129.699
128.572
128.486
127.843
126.023
125.431
77.478
77.160
76.842
50.623
41.863
39.585
32.767
f1 (ppm)
Ph
NH_2
Cl
Cl

Fig. 3, 3n

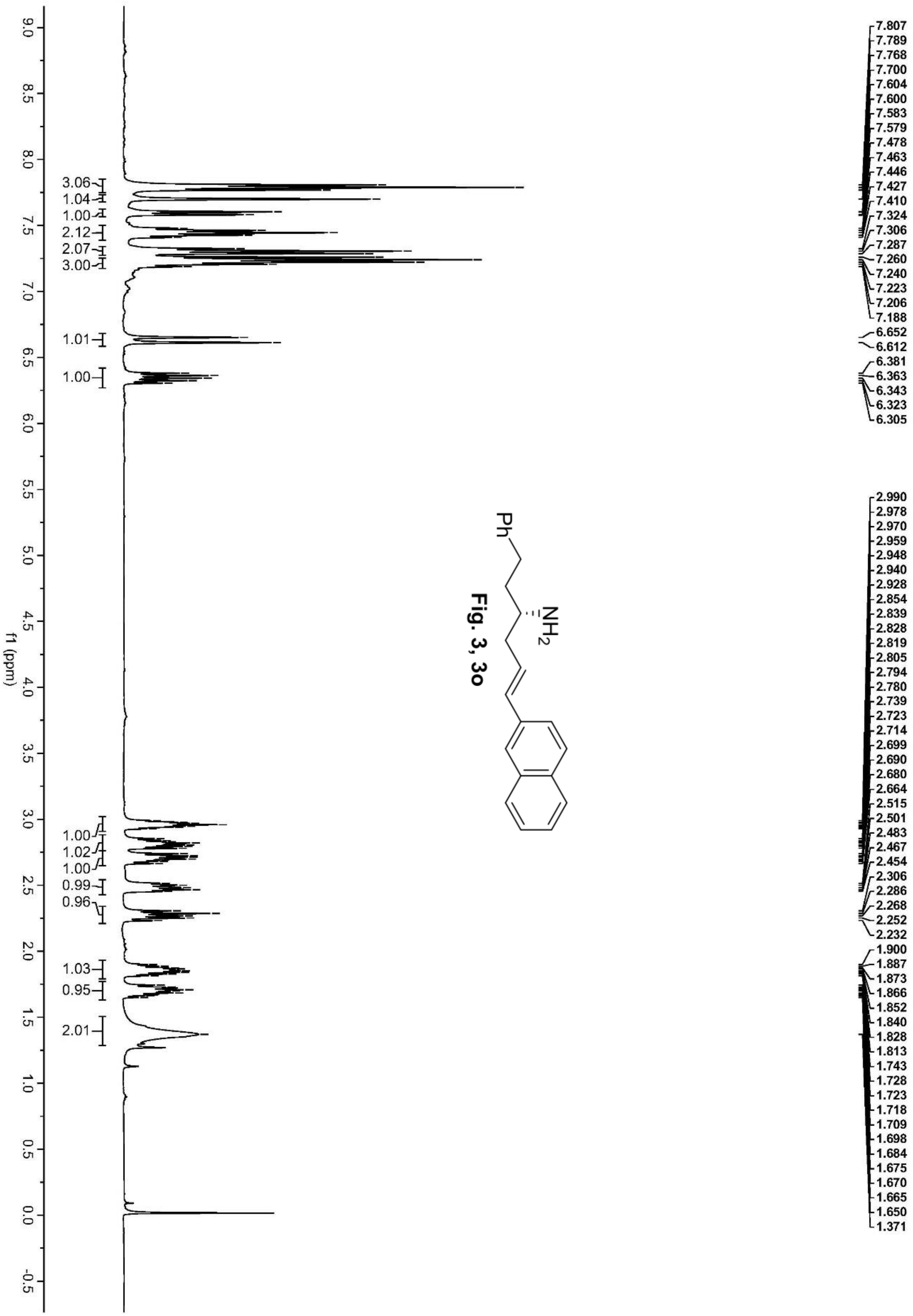

Ph
NH2
f1 (ppm)
9.0 8.5 8.0 7.5 7.0 6.5 6.0 5.5 5.0 4.5 4.0 3.5 3.0 2.5 2.0 1.5 1.0 0.5 0.0 -0.5
3.06 1.04 1.00 2.12 2.07 3.00 1.01 1.00 1.00 1.02 1.00 0.99 0.96 1.03 0.95 2.01
7.807 7.789 7.768 7.700 7.604 7.600 7.583 7.579 7.478 7.463 7.446 7.427 7.410 7.324 7.306 7.287 7.260 7.240 7.223 7.206 7.188
6.652 6.612 6.381 6.363 6.343 6.323 6.305
2.990 2.978 2.970 2.959 2.948 2.940 2.928 2.854 2.839 2.828 2.819 2.805 2.794 2.780 2.739 2.723 2.714 2.699 2.690 2.680 2.664 2.515 2.501 2.483 2.467 2.454 2.306 2.286 2.268 2.252 2.232
1.900 1.887 1.873 1.866 1.852 1.840 1.828 1.813 1.743 1.728 1.723 1.718 1.709 1.698 1.684 1.675 1.670 1.665 1.650 1.371

Fig. 3, 3o

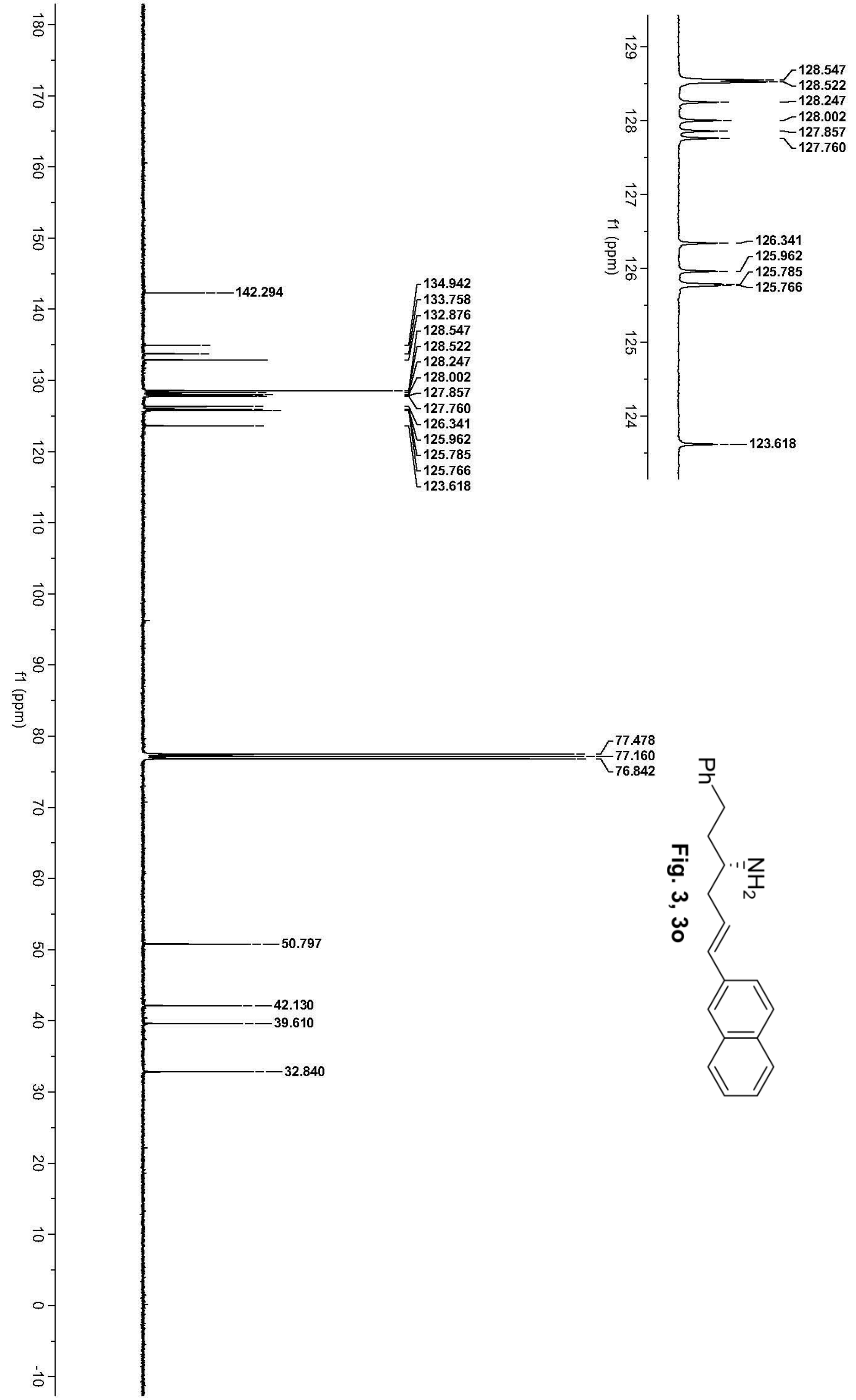
142.294
134.942
133.758
132.876
128.547
128.522
128.247
128.002
127.857
127.760
126.341
125.962
125.785
125.766
123.618
77.478
77.160
76.842
50.797
42.130
39.610
32.840
f1 (ppm)
Ph
NH2

Fig. 3, 3o

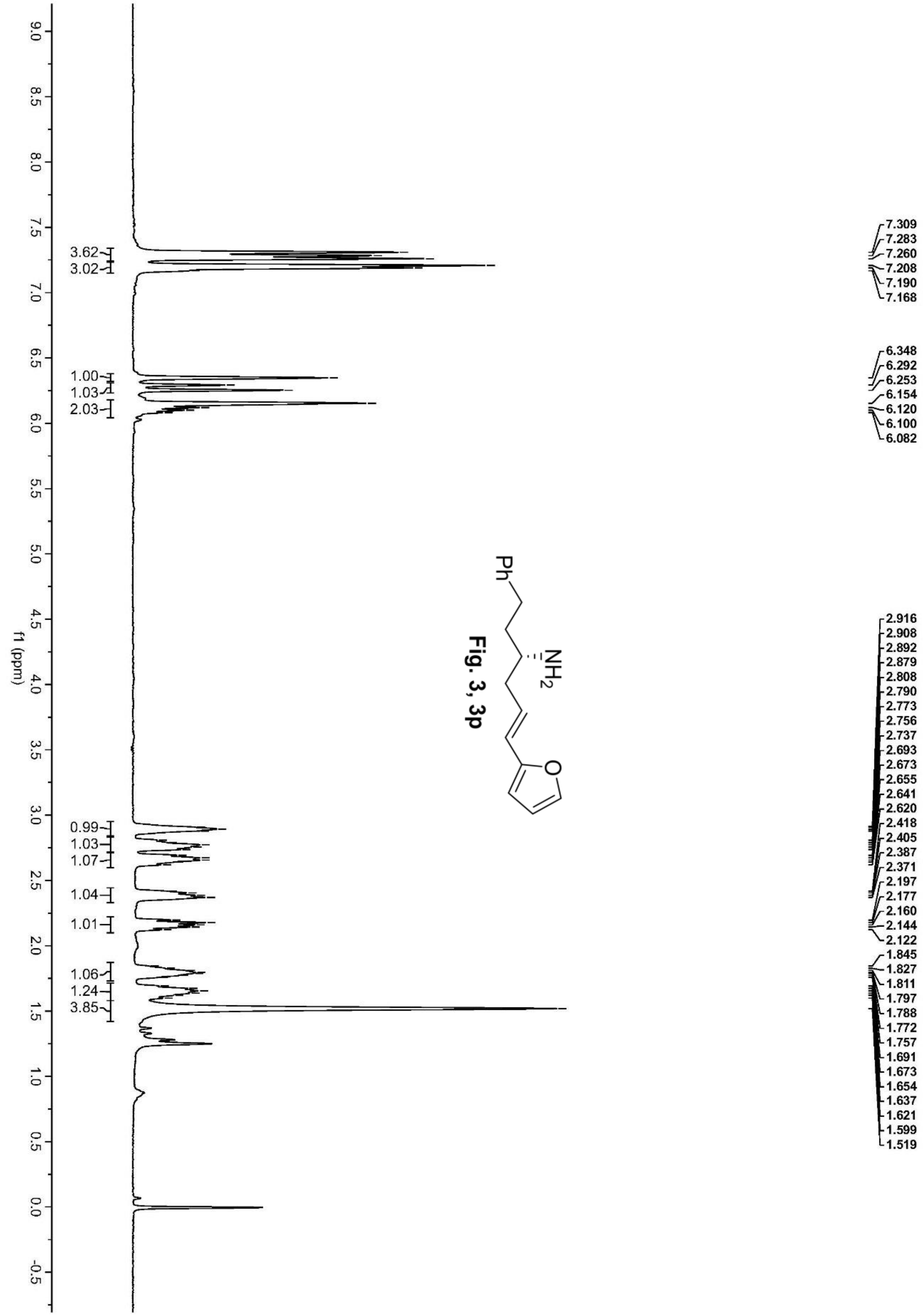

7.309
7.283
7.260
7.208
7.190
7.168
6.348
6.292
6.253
6.154
6.120
6.100
6.082
2.916
2.908
2.892
2.879
2.808
2.790
2.773
2.756
2.737
2.693
2.673
2.655
2.641
2.620
2.418
2.405
2.387
2.371
2.197
2.177
2.160
2.144
2.122
1.845
1.827
1.811
1.797
1.788
1.772
1.757
1.691
1.673
1.654
1.637
1.621
1.599
1.519
Ph
NH2
O
3.62
3.02
1.00
1.03
2.03
0.99
1.03
1.07
1.04
1.01
1.06
1.24
3.85
f1 (ppm)

Fig. 3, 3p

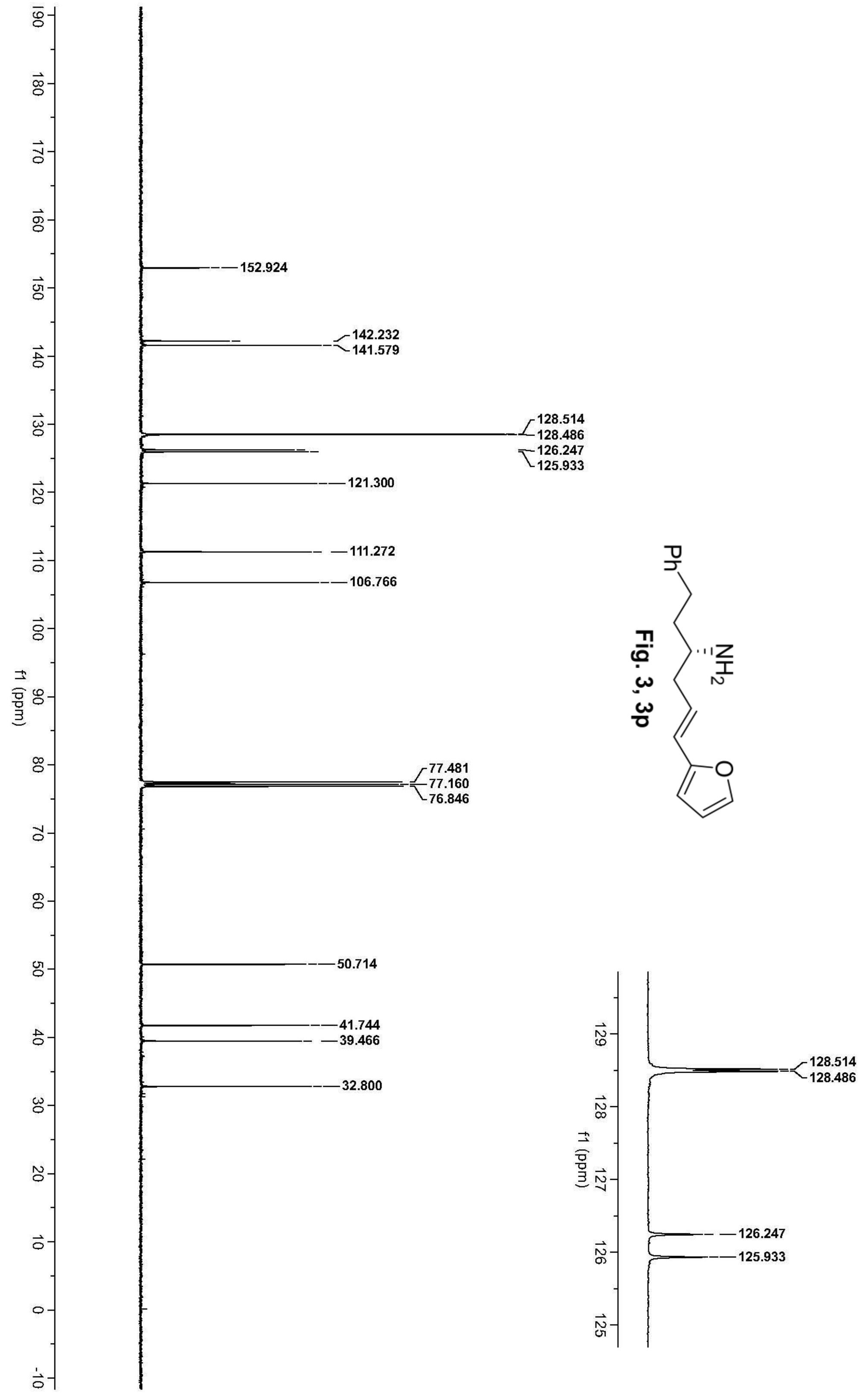
152.924
142.232
141.579
128.514
128.486
126.247
125.933
121.300
111.272
106.766
77.481
77.160
76.846
50.714
41.744
39.466
32.800
f1 (ppm)
Ph
NH2
O
129
128
127
126
125
128.514
128.486
126.247
125.933
f1 (ppm)

Fig. 3, 3p

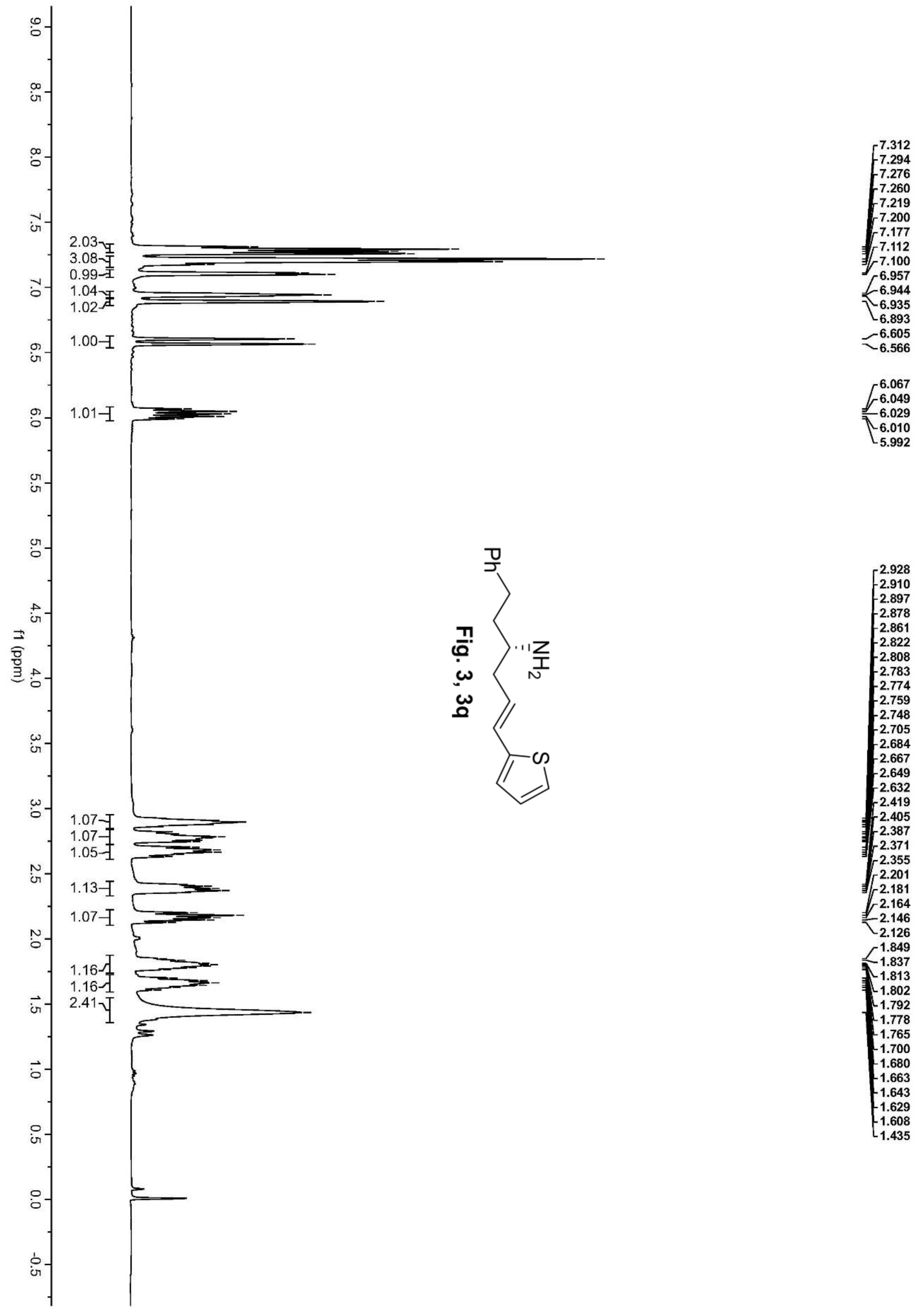
7.312
7.294
7.276
7.260
7.219
7.200
7.177
7.112
7.100
6.957
6.944
6.935
6.893
6.605
6.566
6.067
6.049
6.029
6.010
5.992
2.928
2.910
2.897
2.878
2.861
2.822
2.808
2.783
2.774
2.759
2.748
2.705
2.684
2.667
2.649
2.632
2.419
2.405
2.387
2.371
2.355
2.201
2.181
2.164
2.146
2.126
1.849
1.837
1.813
1.802
1.792
1.778
1.765
1.700
1.680
1.663
1.643
1.629
1.608
1.435
2.03
3.08
0.99
1.04
1.02
1.00
1.01
1.07
1.07
1.05
1.13
1.07
1.16
1.16
2.41
f1 (ppm)
Ph
NH2
S
Fig. 3, 3q

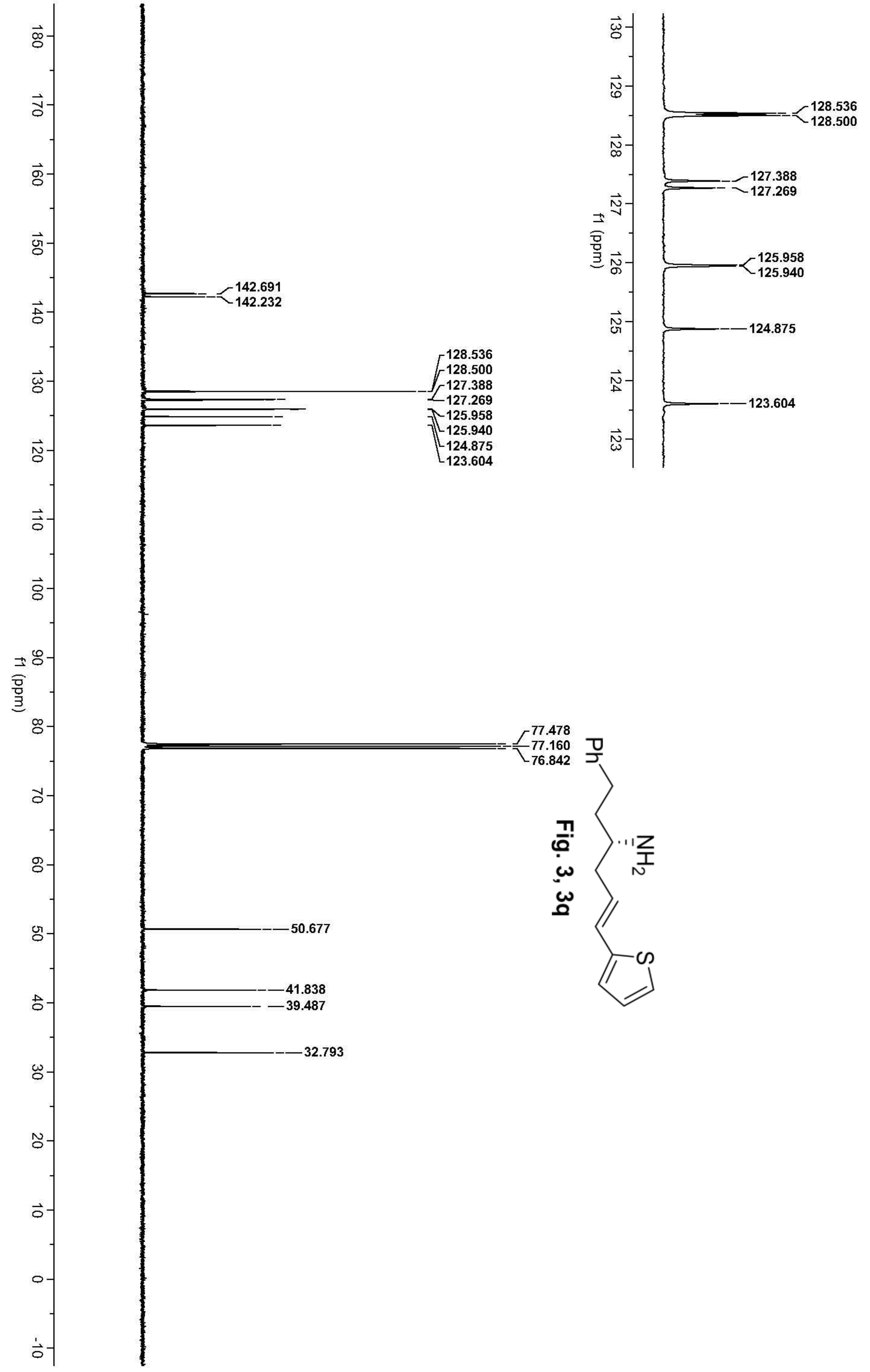

142.691
142.232
128.536
128.500
127.388
127.269
125.958
125.940
124.875
123.604
77.478
77.160
76.842
50.677
41.838
39.487
32.793
f1 (ppm)
Ph
NH2
S

Fig. 3, 3q

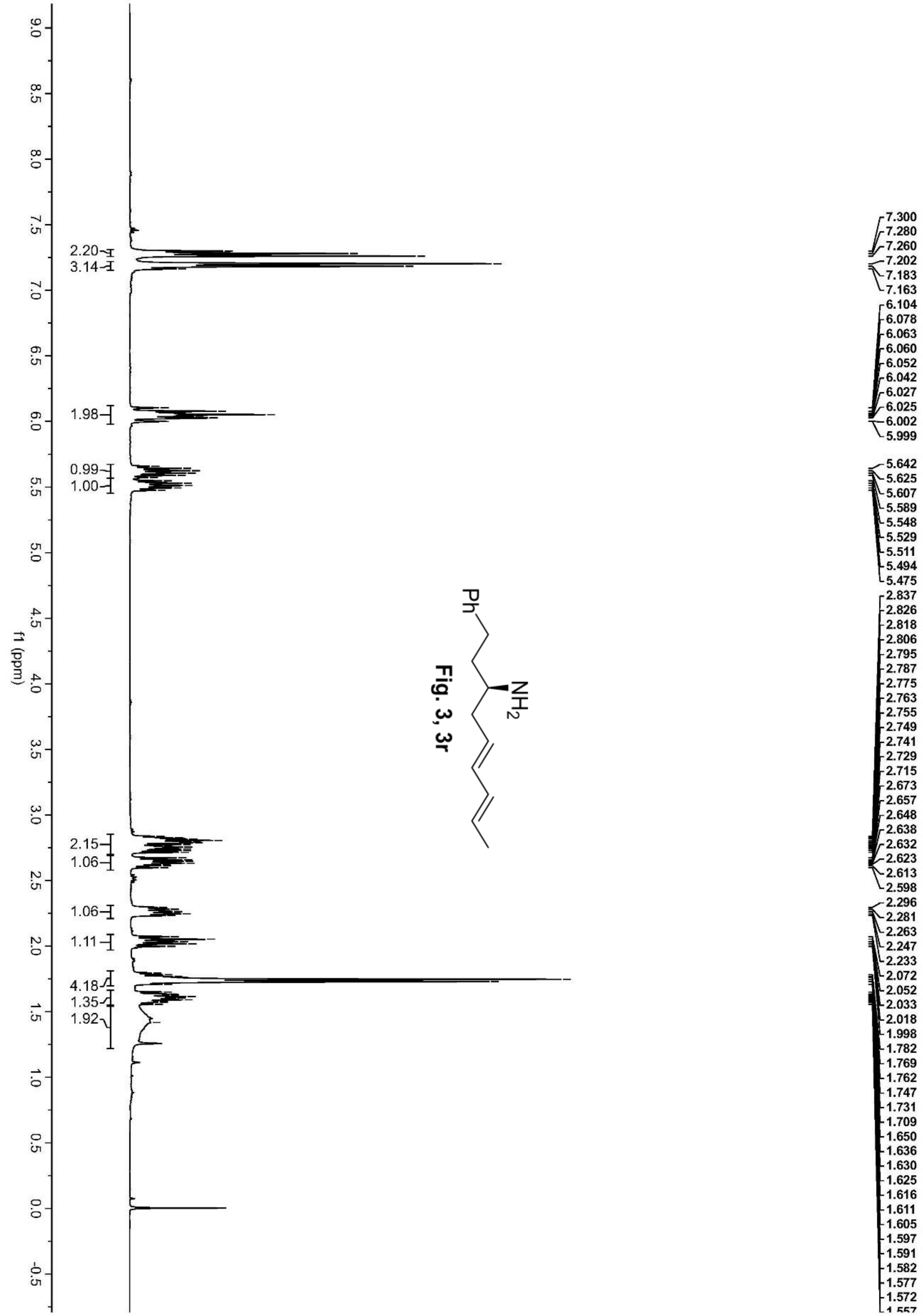

9.0
8.5
8.0
7.5
7.0
6.5
6.0
5.5
5.0
4.5
4.0
3.5
3.0
2.5
2.0
1.5
1.0
0.5
0.0
-0.5
f1 (ppm)
2.20
3.14
1.98
0.99
1.00
2.15
1.06
1.06
1.11
4.18
1.35
1.92
Ph
NH2
7.300
7.280
7.260
7.202
7.183
7.163
6.104
6.078
6.063
6.060
6.052
6.042
6.027
6.025
6.002
5.999
5.642
5.625
5.607
5.589
5.548
5.529
5.511
5.494
5.475
2.837
2.826
2.818
2.806
2.795
2.787
2.775
2.763
2.755
2.749
2.741
2.729
2.715
2.673
2.657
2.648
2.638
2.632
2.623
2.613
2.598
2.296
2.281
2.263
2.247
2.233
2.072
2.052
2.033
2.018
1.998
1.782
1.769
1.762
1.747
1.731
1.709
1.650
1.636
1.630
1.625
1.616
1.611
1.605
1.597
1.591
1.582
1.577
1.572

Fig. 3, 3r

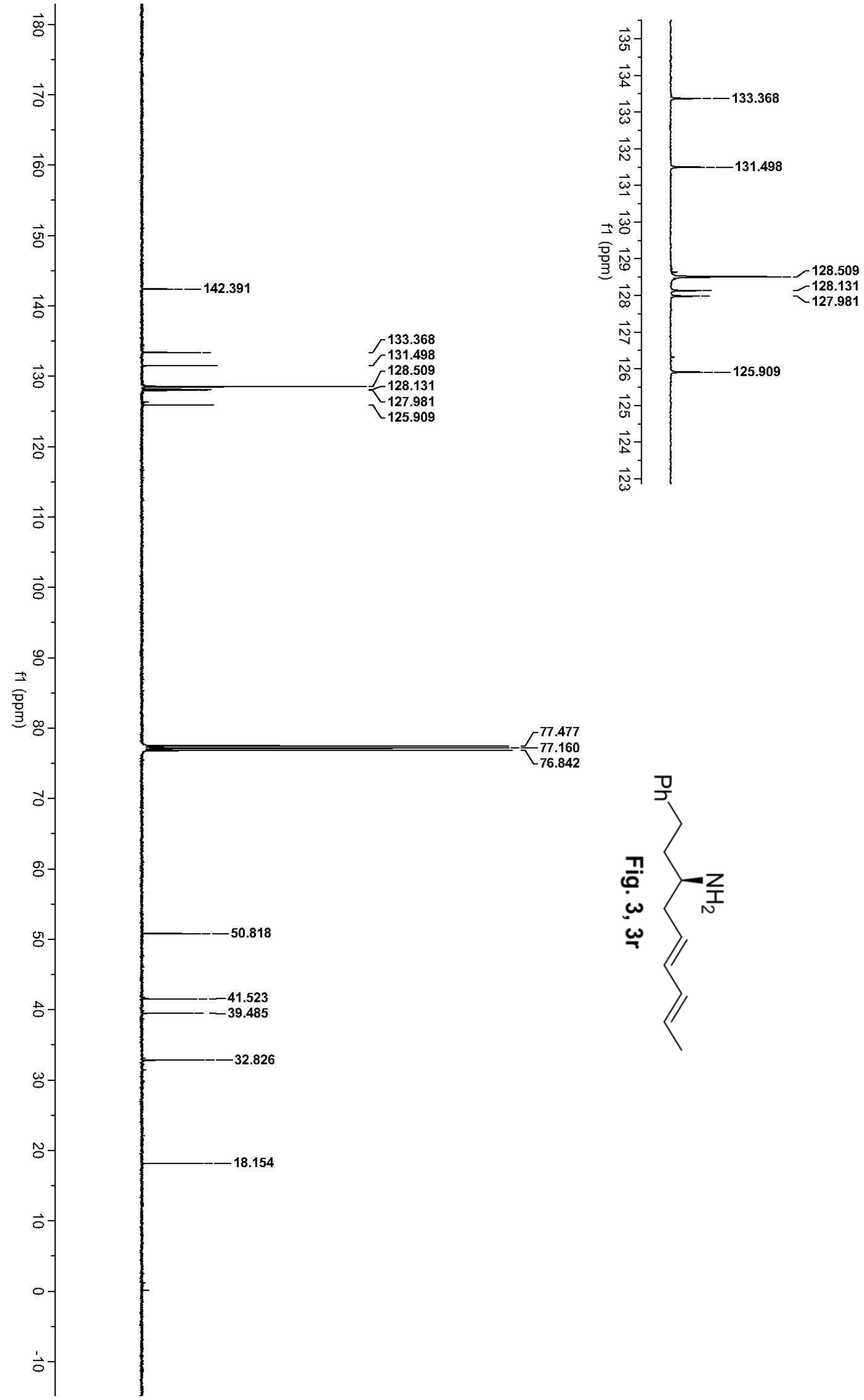

142.391
133.368
131.498
128.509
128.131
127.981
125.909
77.477
77.160
76.842
50.818
41.523
39.485
32.826
18.154
f1 (ppm)
Ph
NH2

Fig. 3, 3r

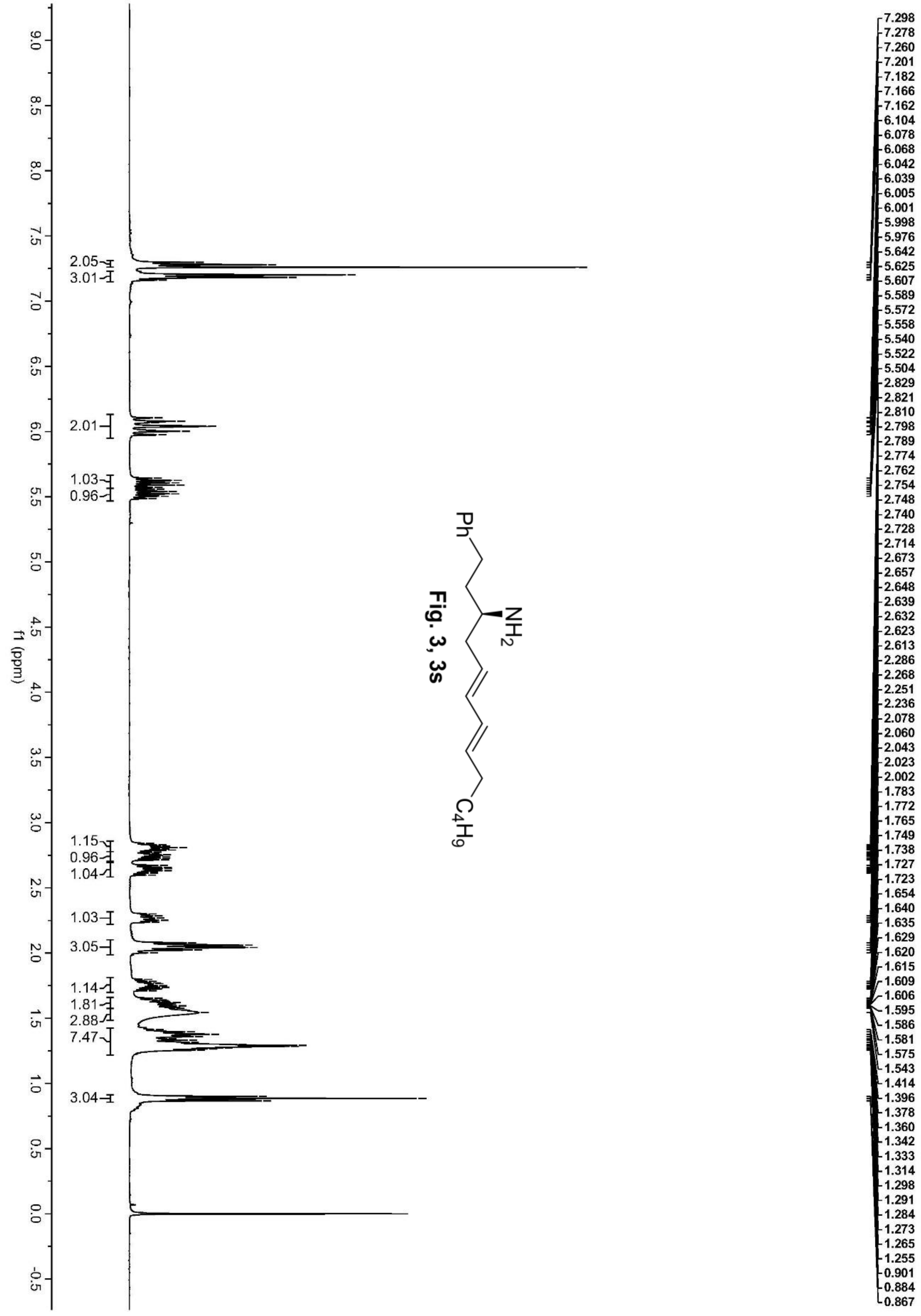
7.298
7.278
7.260
7.201
7.182
7.166
7.162
6.104
6.078
6.068
6.042
6.039
6.005
6.001
5.998
5.976
5.642
5.625
5.607
5.589
5.572
5.558
5.540
5.522
5.504
2.829
2.821
2.810
2.798
2.789
2.774
2.762
2.754
2.748
2.740
2.728
2.714
2.673
2.657
2.648
2.639
2.632
2.623
2.613
2.286
2.268
2.251
2.236
2.078
2.060
2.043
2.023
2.002
1.783
1.772
1.765
1.749
1.738
1.727
1.723
1.654
1.640
1.635
1.629
1.620
1.615
1.609
1.606
1.595
1.586
1.581
1.575
1.543
1.414
1.396
1.378
1.360
1.342
1.333
1.314
1.298
1.291
1.284
1.273
1.265
1.255
0.901
0.884
0.867
Ph
NH2
C4H9
2.05
3.01
2.01
1.03
0.96
1.15
0.96
1.04
1.03
3.05
1.14
1.81
2.88
7.47
3.04
9.0
8.5
8.0
7.5
7.0
6.5
6.0
5.5
5.0
4.5
4.0
3.5
3.0
2.5
2.0
1.5
1.0
0.5
0.0
-0.5
f1 (ppm)

Fig. 3, 3s

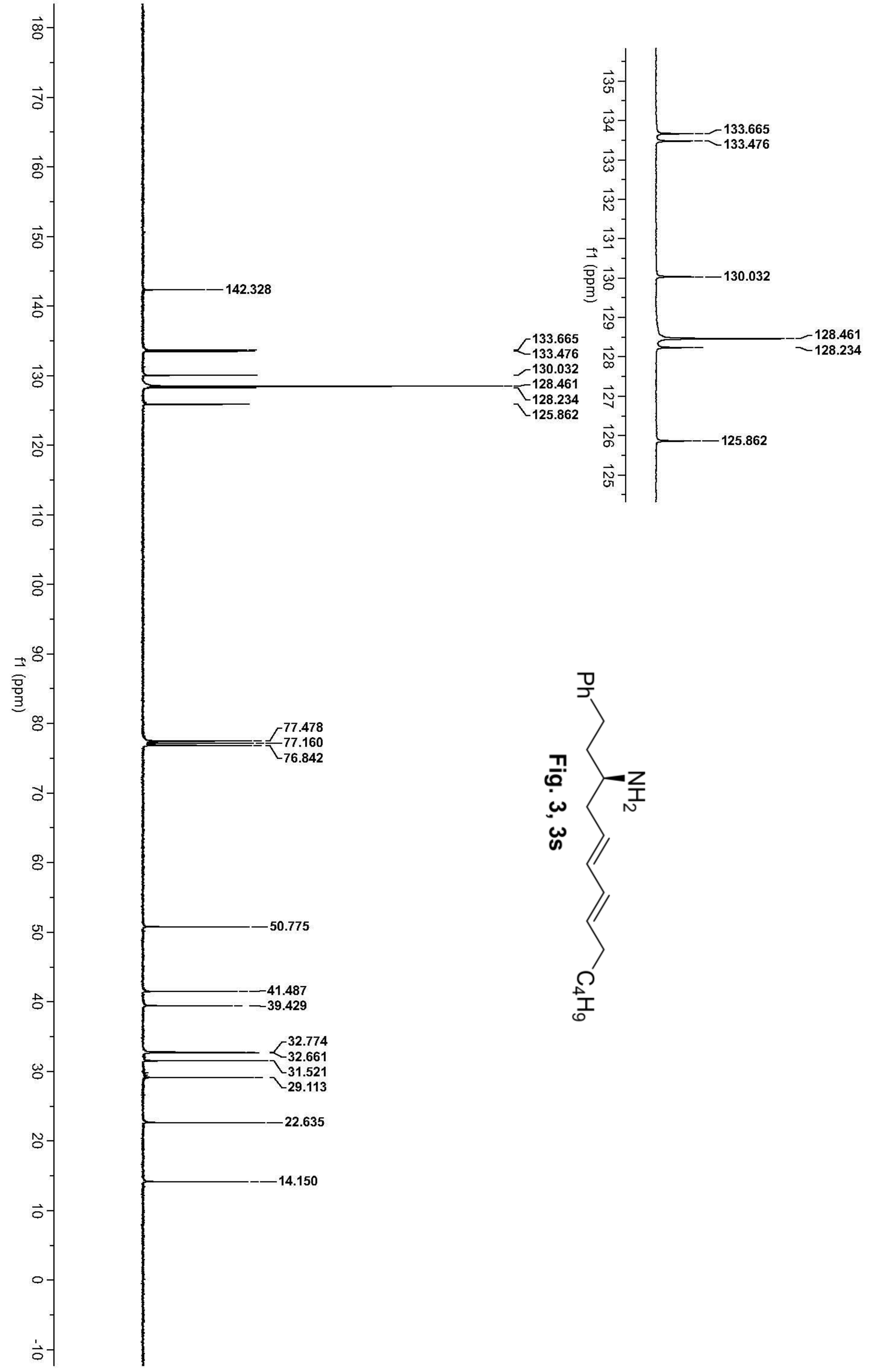
142.328
133.665
133.476
130.032
128.461
128.234
125.862
77.478
77.160
76.842
50.775
41.487
39.429
32.774
32.661
31.521
29.113
22.635
14.150
f1 (ppm)
Ph
NH_2
C_4H_9

Fig. 3, 3s

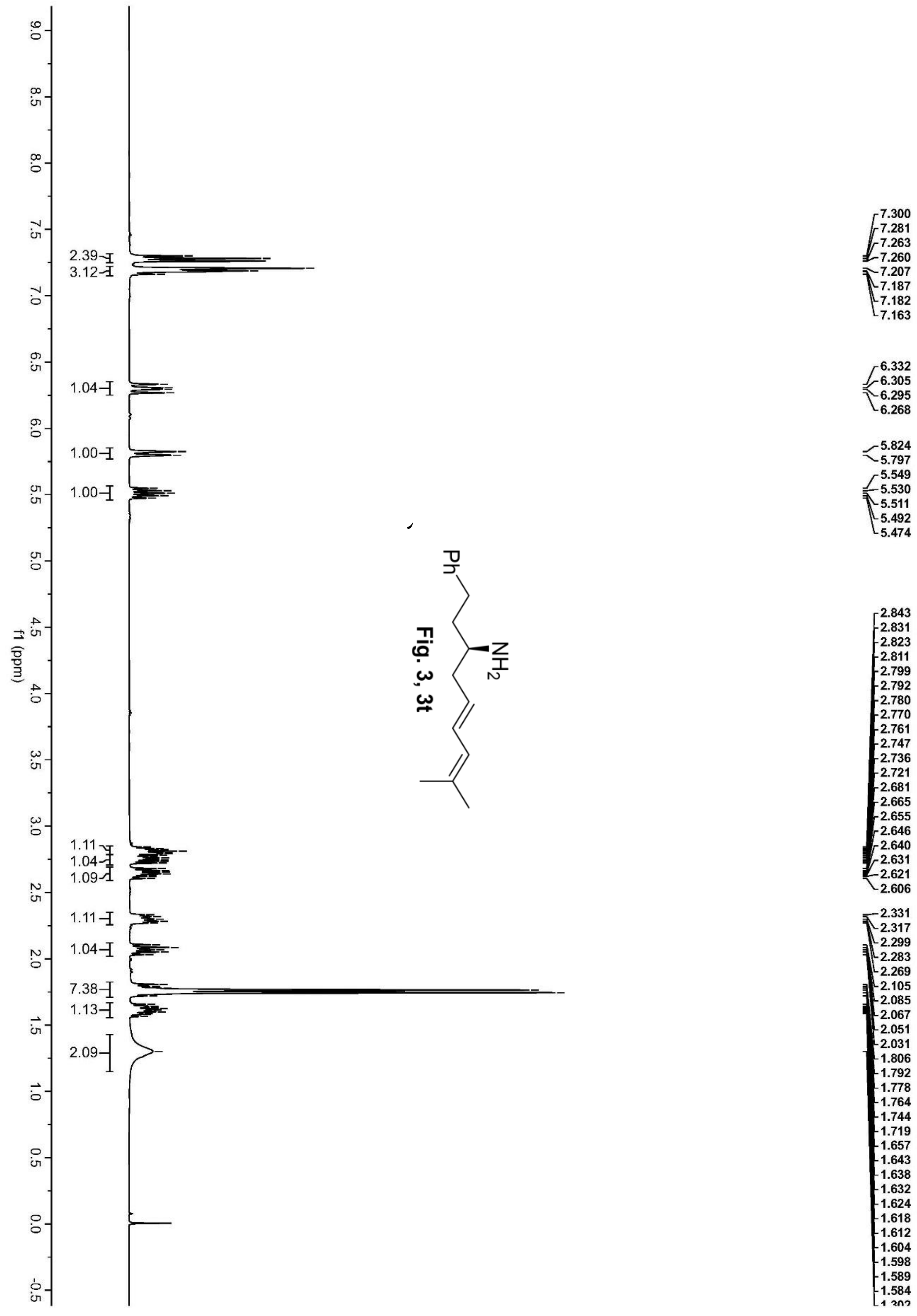

Ph
NH2
Fig. 3, 3t
f1 (ppm)
7.300
7.281
7.263
7.260
7.207
7.187
7.182
7.163
6.332
6.305
6.295
6.268
5.824
5.797
5.549
5.530
5.511
5.492
5.474
2.843
2.831
2.823
2.811
2.799
2.792
2.780
2.770
2.761
2.747
2.736
2.721
2.681
2.665
2.655
2.646
2.640
2.631
2.621
2.606
2.331
2.317
2.299
2.283
2.269
2.105
2.085
2.067
2.051
2.031
1.806
1.792
1.778
1.764
1.744
1.719
1.657
1.643
1.638
1.632
1.624
1.618
1.612
1.604
1.598
1.589
1.584
2.39
3.12
1.04
1.00
1.00
1.11
1.04
1.09
1.11
1.04
7.38
1.13
2.09

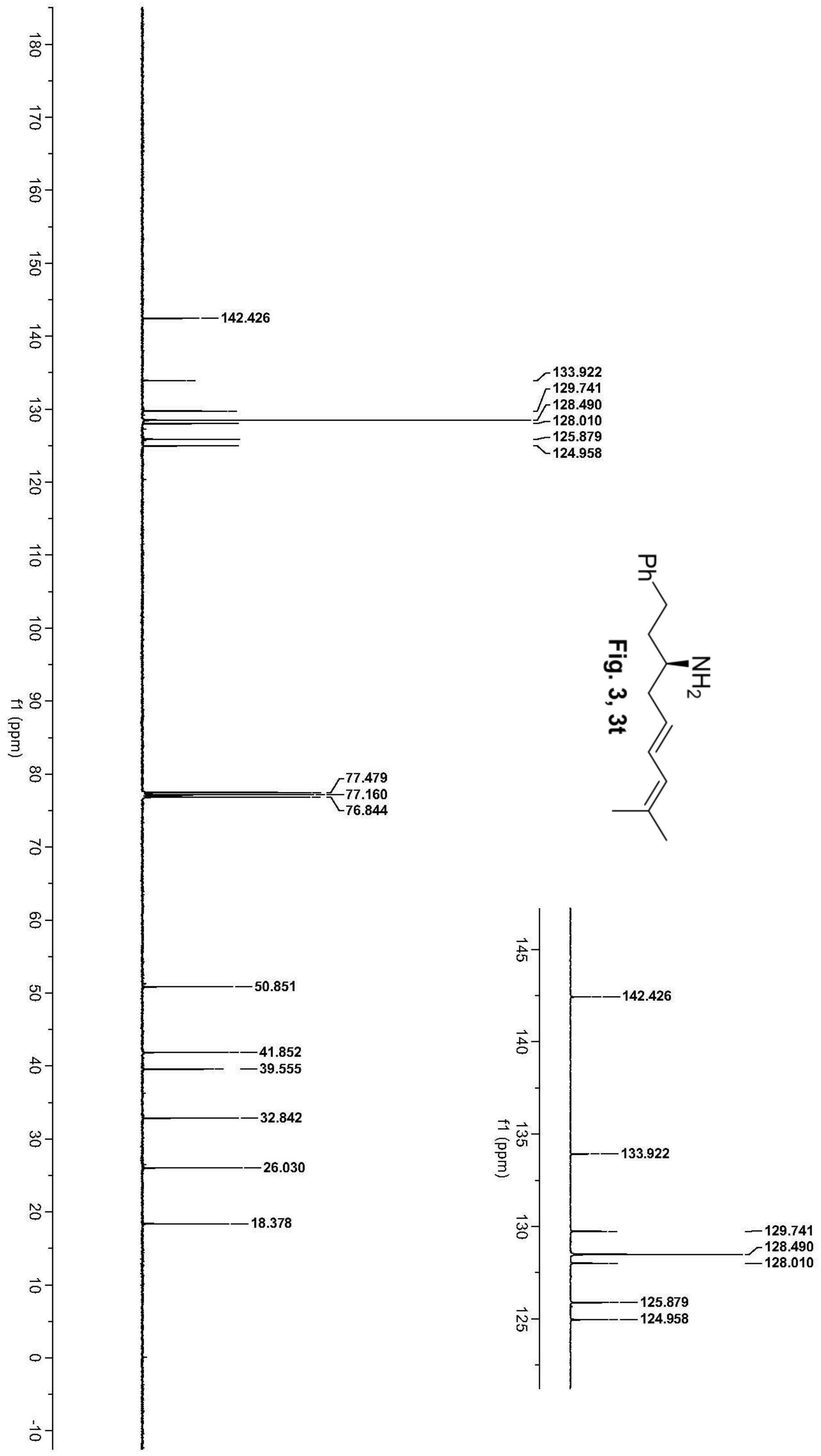
142.426
133.922
129.741
128.490
128.010
125.879
124.958
77.479
77.160
76.844
50.851
41.852
39.555
32.842
26.030
18.378
f1 (ppm)
Ph
NH2

Fig. 3, 3t

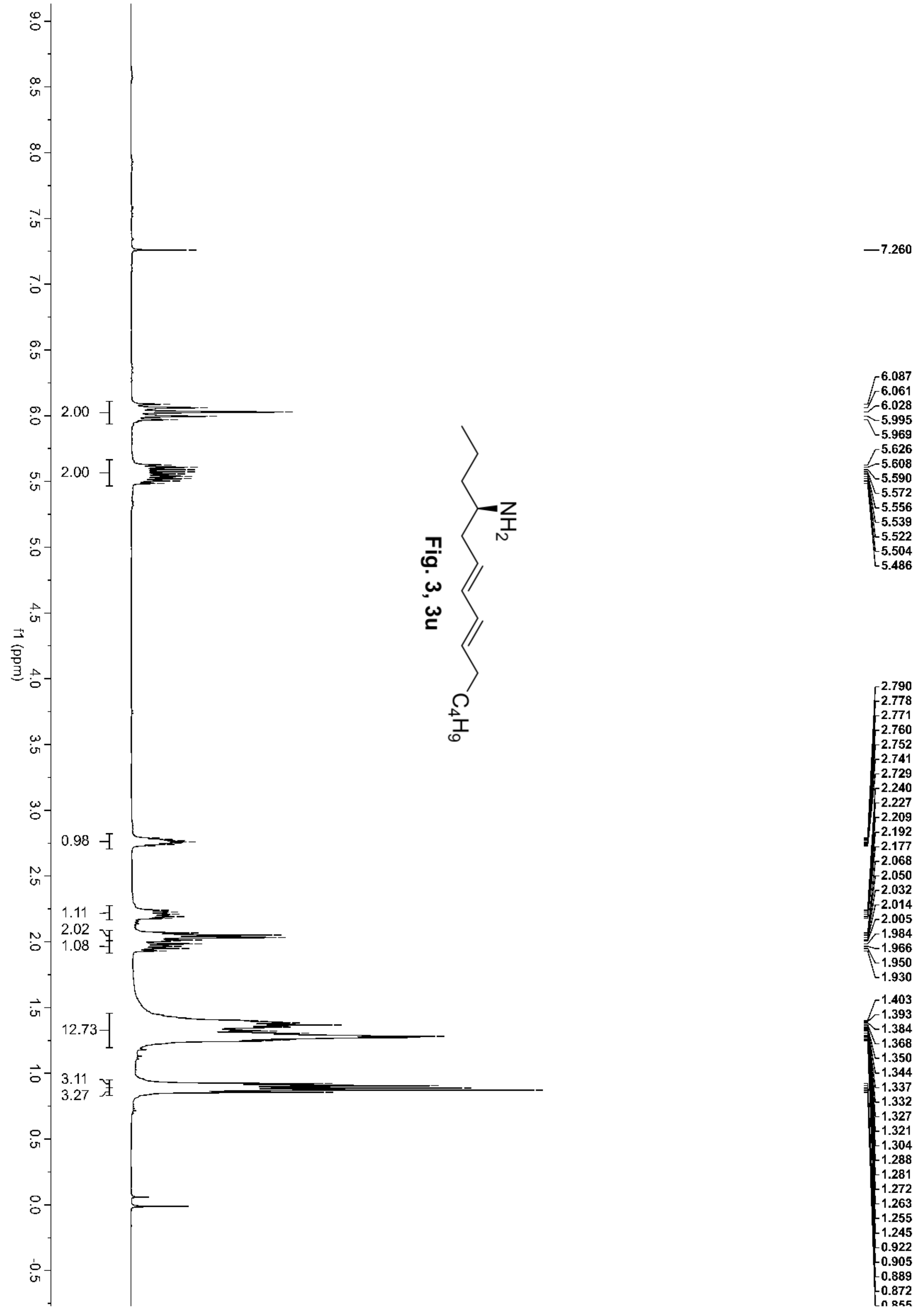

Fig. 3, 3u

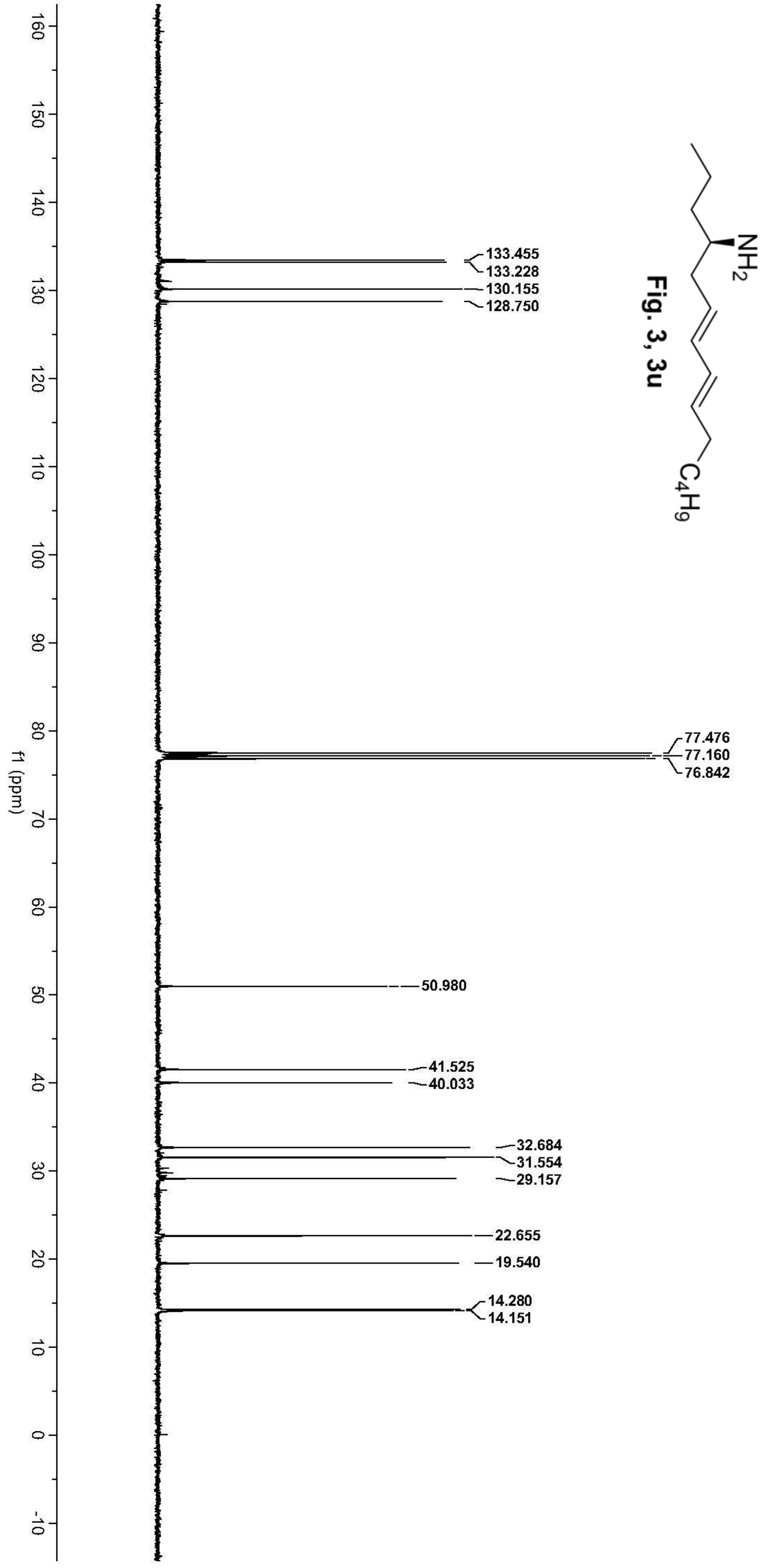
133.455
133.228
130.155
128.750
77.476
77.160
76.842
50.980
41.525
40.033
32.684
31.554
29.157
22.655
19.540
14.280
14.151
NH2
C4H9
f1 (ppm)
160
150
140
130
120
110
100
90
80
70
60
50
40
30
20
10
0
-10
Fig. 3, 3u

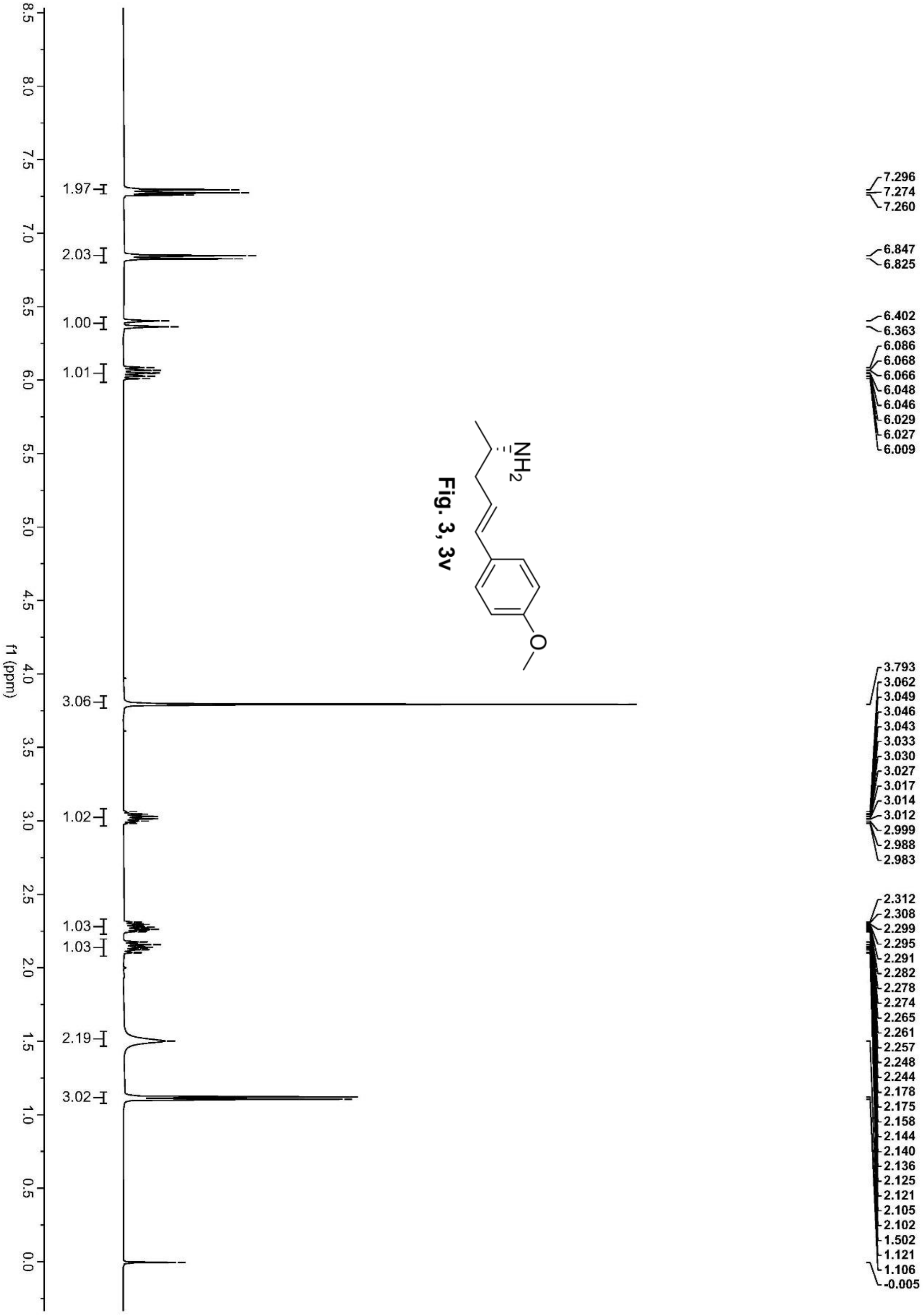

NH2
f1 (ppm)
8.5
8.0
7.5
7.0
6.5
6.0
5.5
5.0
4.5
4.0
3.5
3.0
2.5
2.0
1.5
1.0
0.5
0.0
1.97
2.03
1.00
1.01
3.06
1.02
1.03
1.03
2.19
3.02
7.296
7.274
7.260
6.847
6.825
6.402
6.363
6.086
6.068
6.066
6.048
6.046
6.029
6.027
6.009
3.793
3.062
3.049
3.046
3.043
3.033
3.030
3.027
3.017
3.014
3.012
2.999
2.988
2.983
2.312
2.308
2.299
2.295
2.291
2.282
2.278
2.274
2.265
2.261
2.257
2.248
2.244
2.178
2.175
2.158
2.144
2.140
2.136
2.125
2.121
2.105
2.102
1.502
1.121
1.106
-0.005

Fig. 3, 3v

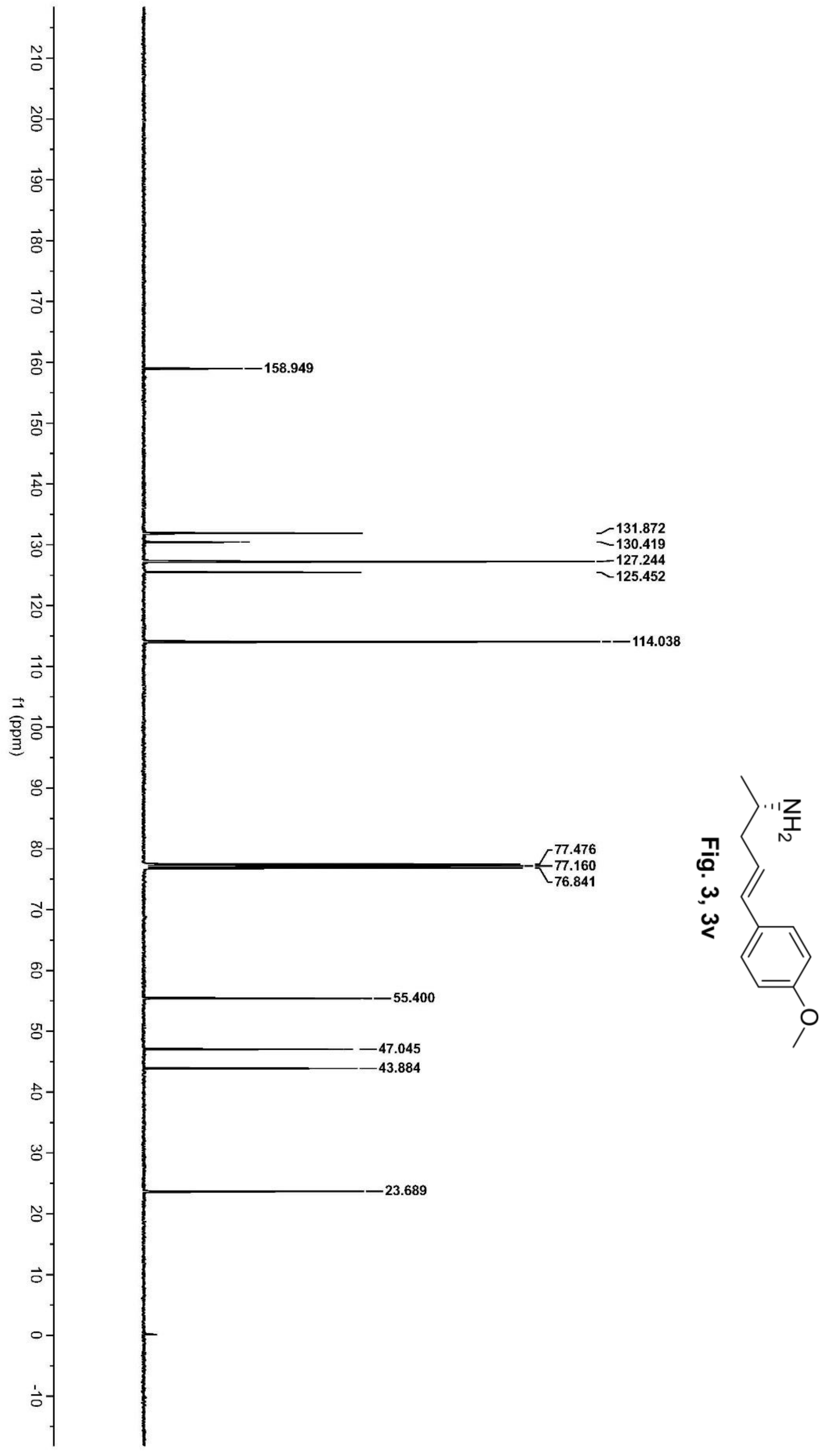

158.949
131.872
130.419
127.244
125.452
114.038
77.476
77.160
76.841
55.400
47.045
43.884
23.689
NH_2
O
f1 (ppm)

Fig. 3, 3v

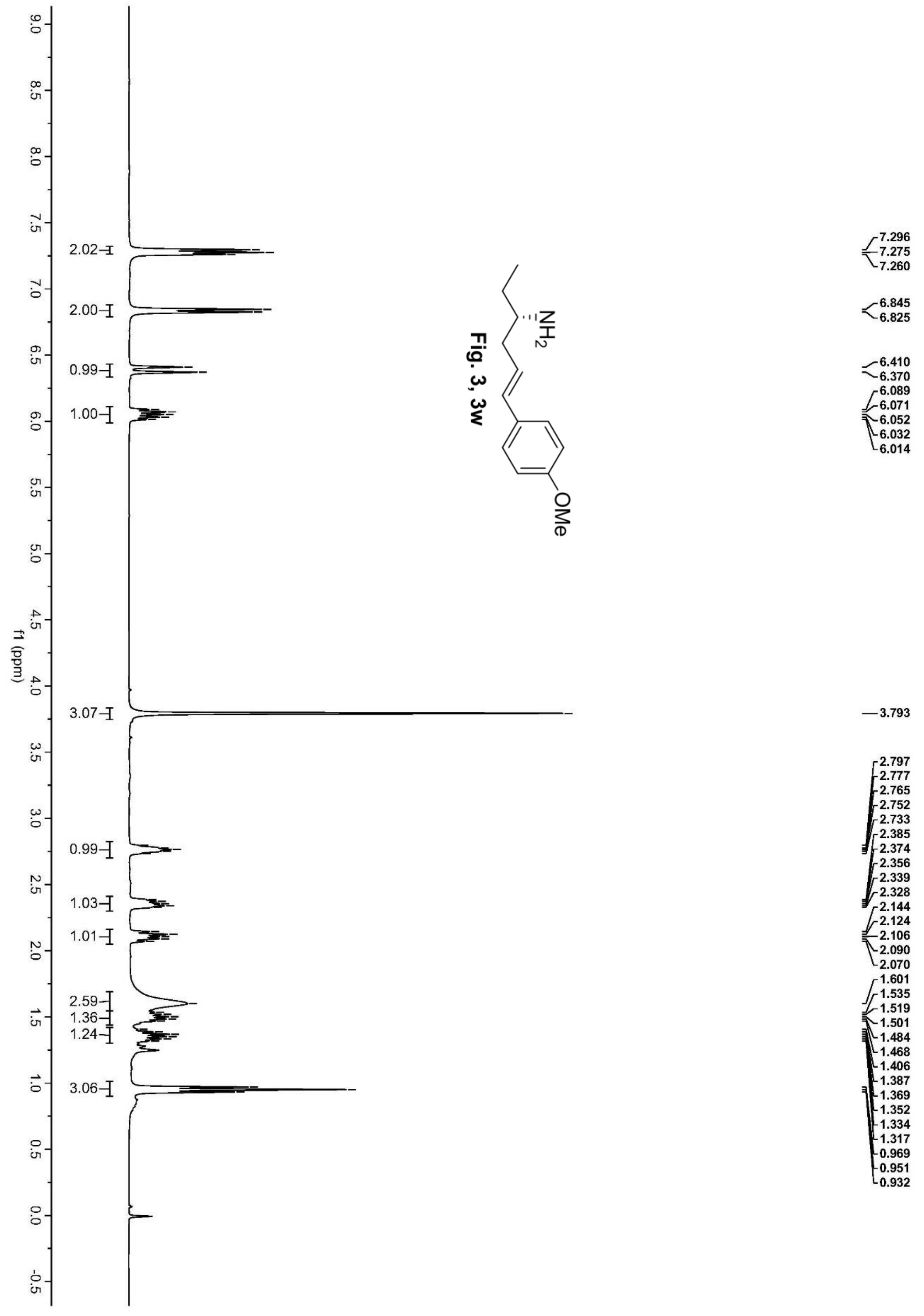
NH2
OMe
f1 (ppm)
9.0 8.5 8.0 7.5 7.0 6.5 6.0 5.5 5.0 4.5 4.0 3.5 3.0 2.5 2.0 1.5 1.0 0.5 0.0 -0.5
2.02 2.00 0.99 1.00 3.07 0.99 1.03 1.01 2.59 1.36 1.24 3.06
7.296 7.275 7.260
6.845 6.825
6.410 6.370 6.089 6.071 6.052 6.032 6.014
3.793
2.797 2.777 2.765 2.752 2.733 2.385 2.374 2.356 2.339 2.328 2.144 2.124 2.106 2.090 2.070 1.601 1.535 1.519 1.501 1.484 1.468 1.406 1.387 1.369 1.352 1.334 1.317 0.969 0.951 0.932
Fig. 3, 3w

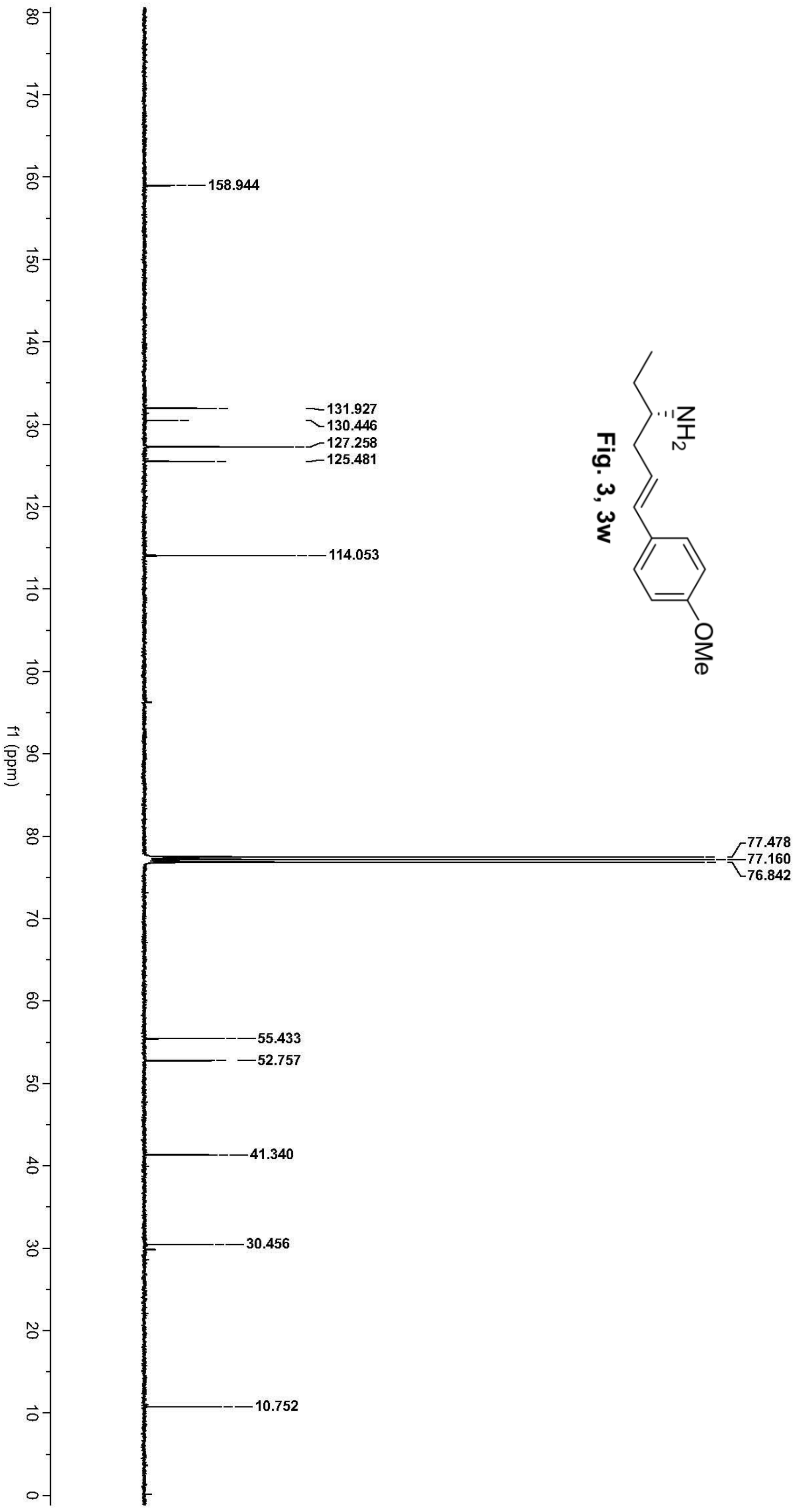
158.944
131.927
130.446
127.258
125.481
114.053
77.478
77.160
76.842
55.433
52.757
41.340
30.456
10.752
NH2
OMe
f1 (ppm)
180
170
160
150
140
130
120
110
100
90
80
70
60
50
40
30
20
10
0

Fig. 3, 3w

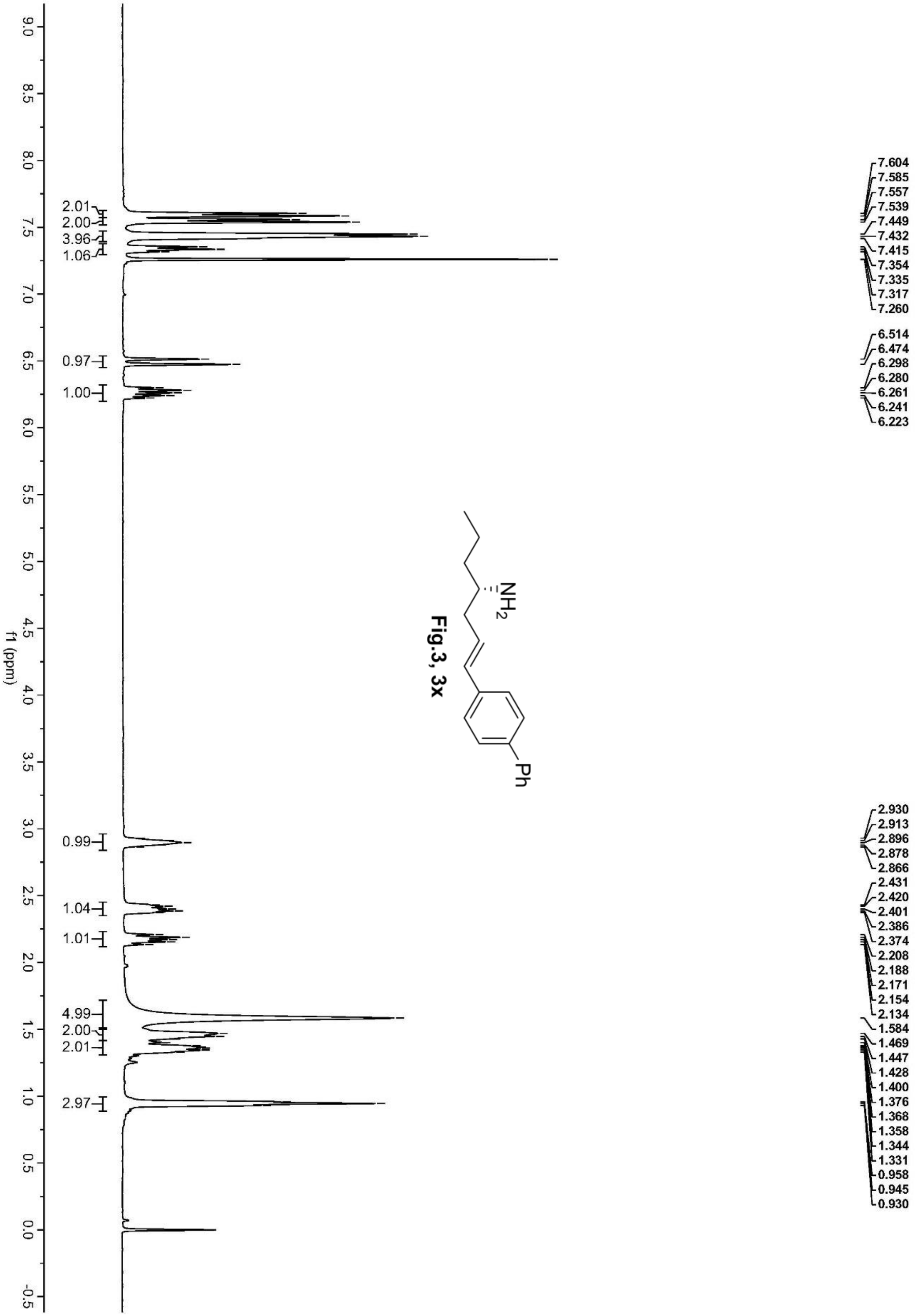

Fig.3, 3x

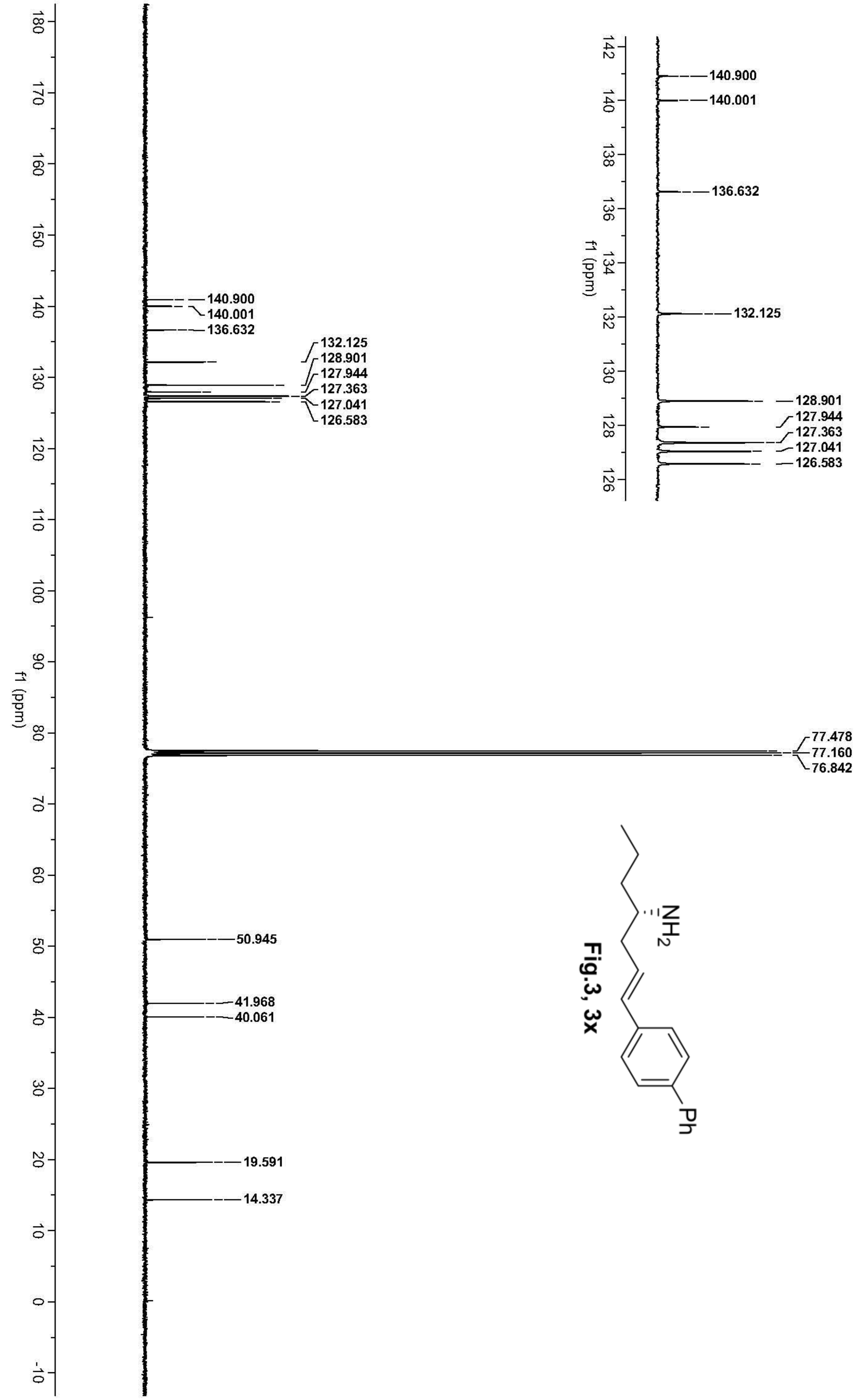
140.900
140.001
136.632
132.125
128.901
127.944
127.363
127.041
126.583
77.478
77.160
76.842
50.945
41.968
40.061
19.591
14.337
f1 (ppm)
NH_2
Ph

Fig.3, 3x

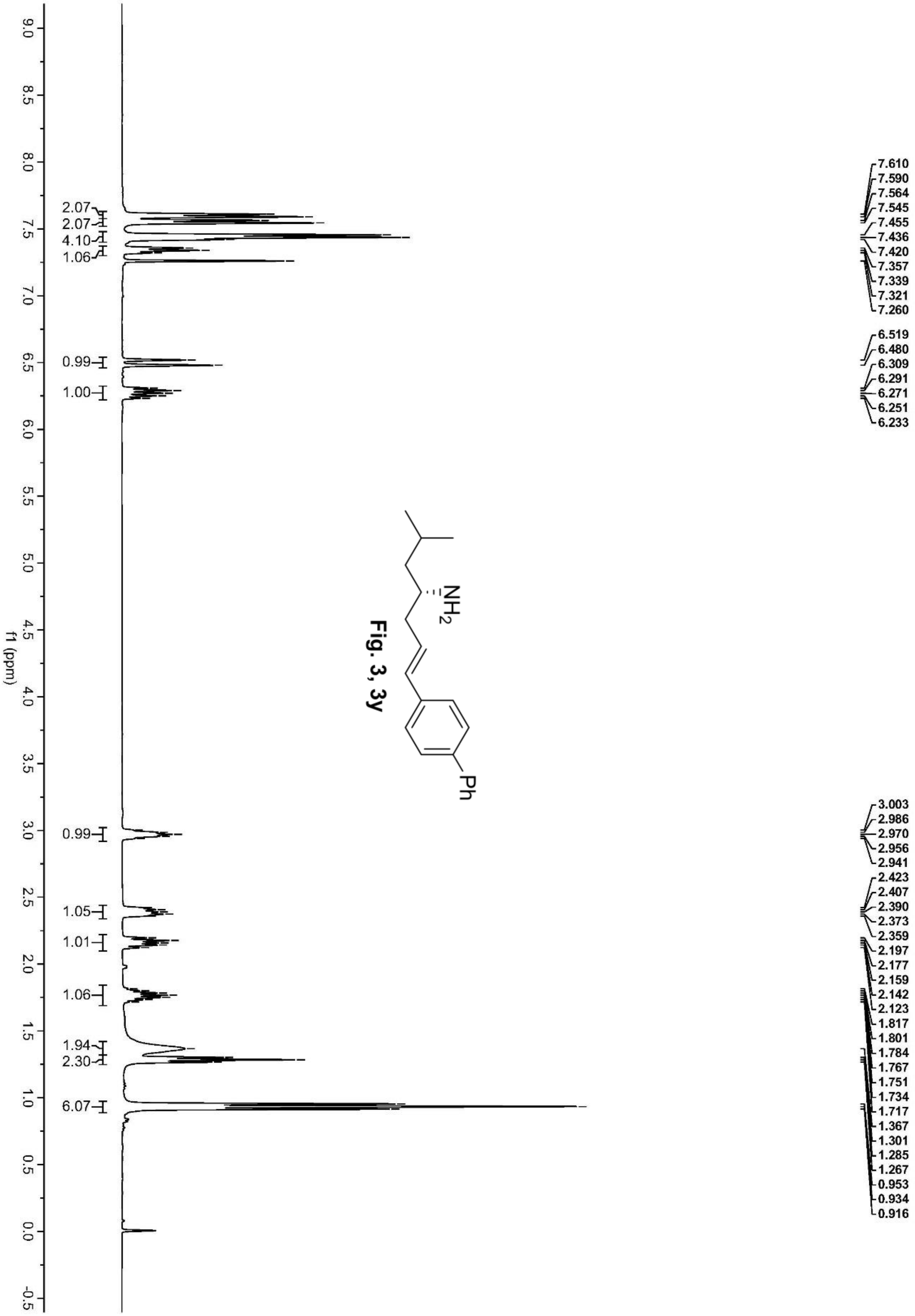

7.610
7.590
7.564
7.545
7.455
7.436
7.420
7.357
7.339
7.321
7.260
6.519
6.480
6.309
6.291
6.271
6.251
6.233
3.003
2.986
2.970
2.956
2.941
2.423
2.407
2.390
2.373
2.359
2.197
2.177
2.159
2.142
2.123
1.817
1.801
1.784
1.767
1.751
1.734
1.717
1.367
1.301
1.285
1.267
0.953
0.934
0.916
2.07
2.07
4.10
1.06
0.99
1.00
0.99
1.05
1.01
1.06
1.94
2.30
6.07
NH2
Ph
Fig. 3, 3y
f1 (ppm)

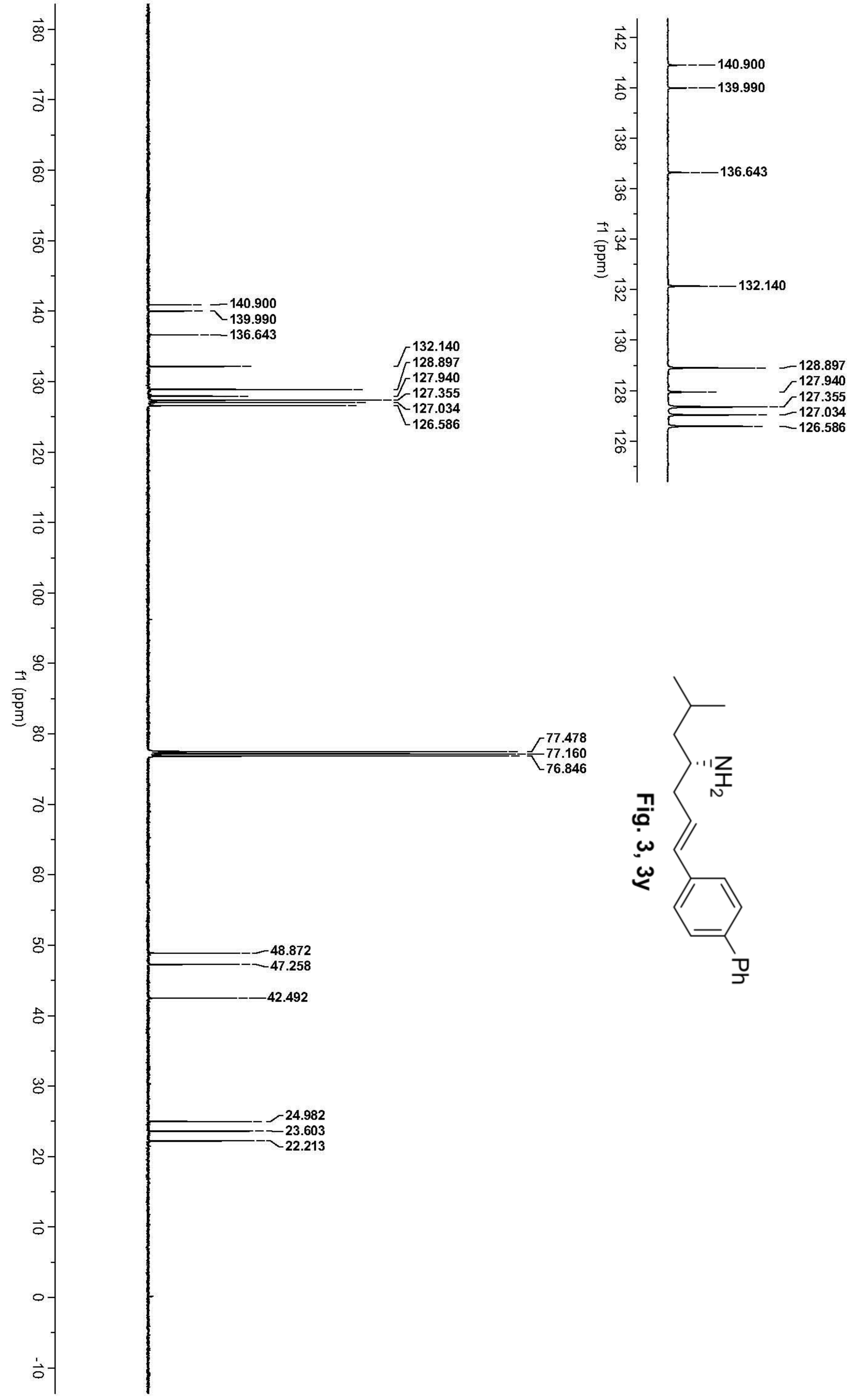
140.900
139.990
136.643
132.140
128.897
127.940
127.355
127.034
126.586
77.478
77.160
76.846
48.872
47.258
42.492
24.982
23.603
22.213
f1 (ppm)
NH2
Ph

Fig. 3, 3y

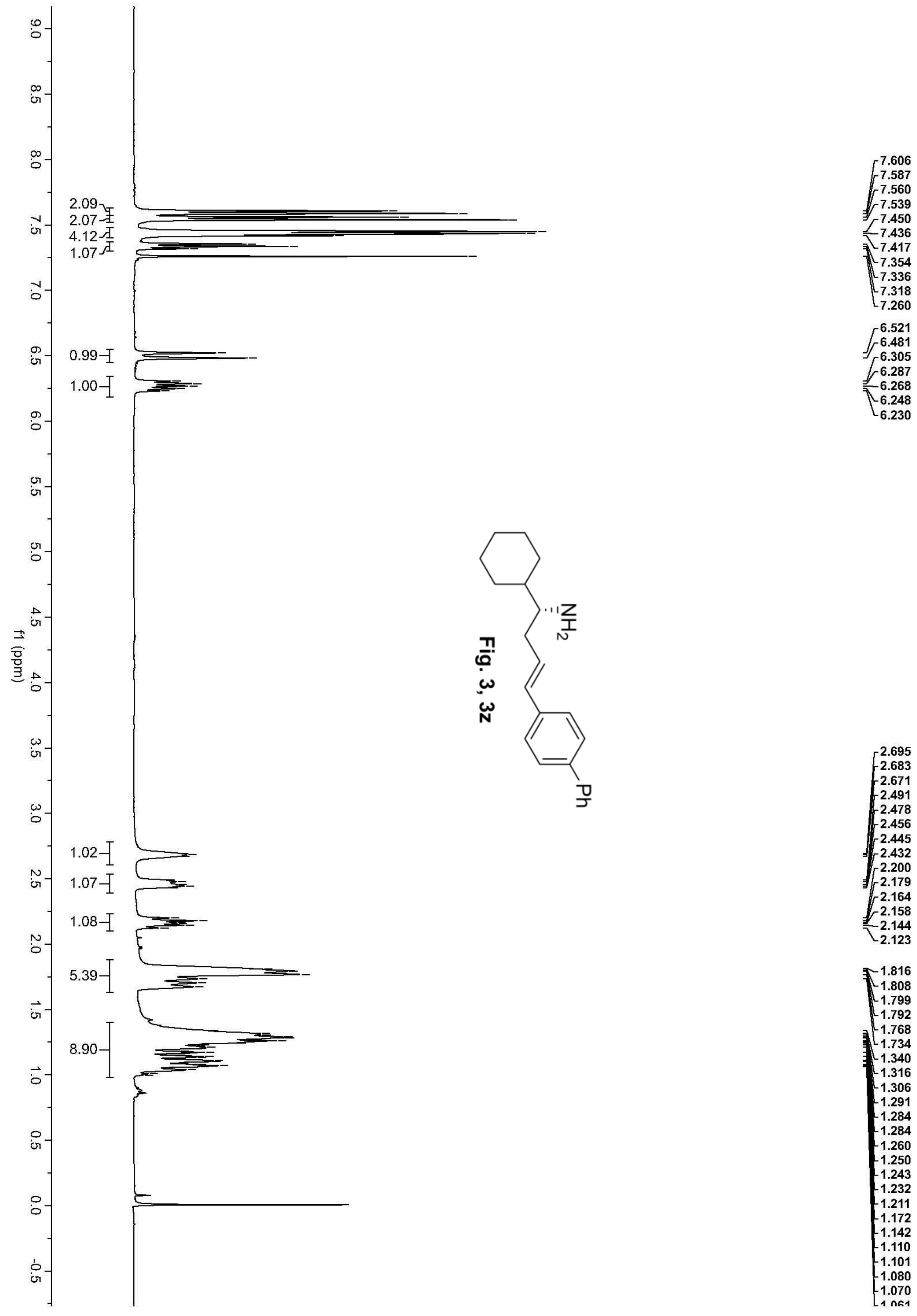
7.606
7.587
7.560
7.539
7.450
7.436
7.417
7.354
7.336
7.318
7.260
6.521
6.481
6.305
6.287
6.268
6.248
6.230
2.695
2.683
2.671
2.491
2.478
2.456
2.445
2.432
2.200
2.179
2.164
2.158
2.144
2.123
1.816
1.808
1.799
1.792
1.768
1.734
1.340
1.316
1.306
1.291
1.284
1.284
1.260
1.250
1.243
1.232
1.211
1.172
1.142
1.110
1.101
1.080
1.070
2.09
2.07
4.12
1.07
0.99
1.00
1.02
1.07
1.08
5.39
8.90
NH2
Ph
f1 (ppm)

Fig. 3, 3z

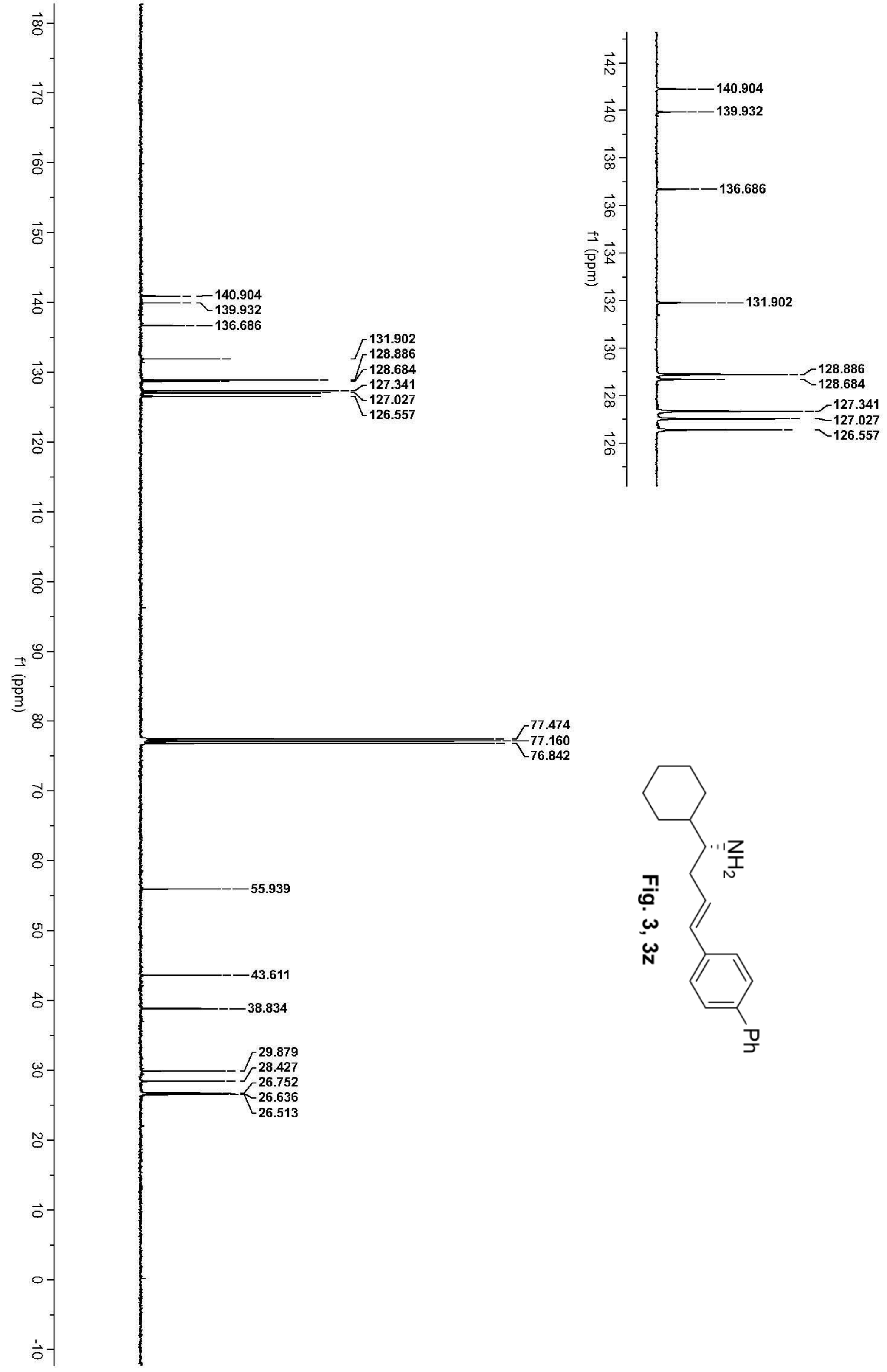
140.904
139.932
136.686
131.902
128.886
128.684
127.341
127.027
126.557
77.474
77.160
76.842
55.939
43.611
38.834
29.879
28.427
26.752
26.636
26.513
f1 (ppm)
NH2
Ph

Fig. 3, 3z

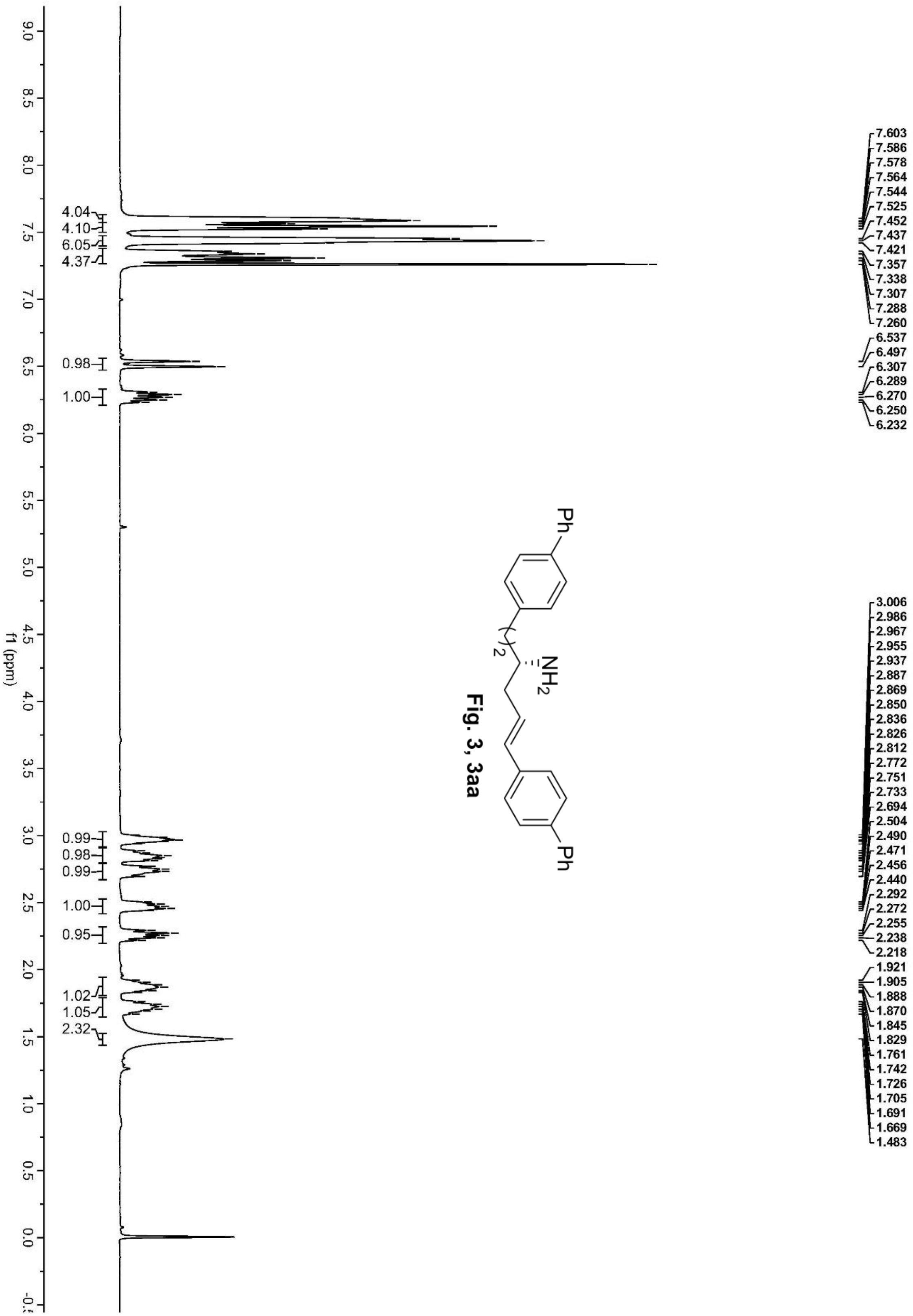
7.603
7.586
7.578
7.564
7.544
7.525
7.452
7.437
7.421
7.357
7.338
7.307
7.288
7.260
6.537
6.497
6.307
6.289
6.270
6.250
6.232
3.006
2.986
2.967
2.955
2.937
2.887
2.869
2.850
2.836
2.826
2.812
2.772
2.751
2.733
2.694
2.504
2.490
2.471
2.456
2.440
2.292
2.272
2.255
2.238
2.218
1.921
1.905
1.888
1.870
1.845
1.829
1.761
1.742
1.726
1.705
1.691
1.669
1.483
4.04
4.10
6.05
4.37
0.98
1.00
0.99
0.98
0.99
1.00
0.95
1.02
1.05
2.32
f1 (ppm)
Ph
NH2
2
Ph

Fig. 3, 3aa

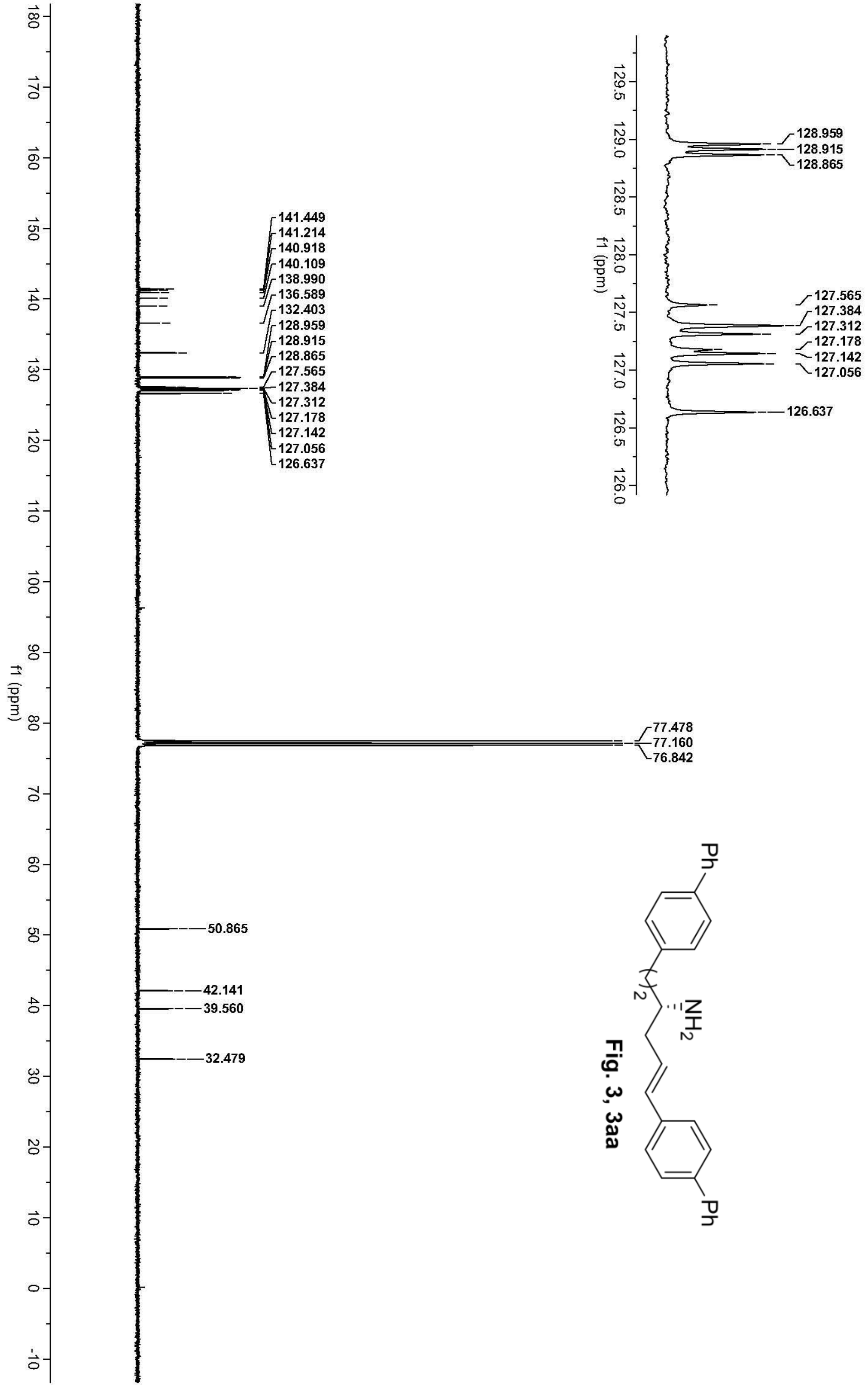
141.449
141.214
140.918
140.109
138.990
136.589
132.403
128.959
128.915
128.865
127.565
127.384
127.312
127.178
127.142
127.056
126.637
77.478
77.160
76.842
50.865
42.141
39.560
32.479
f1 (ppm)
Ph
NH2
Ph

Fig. 3, 3aa

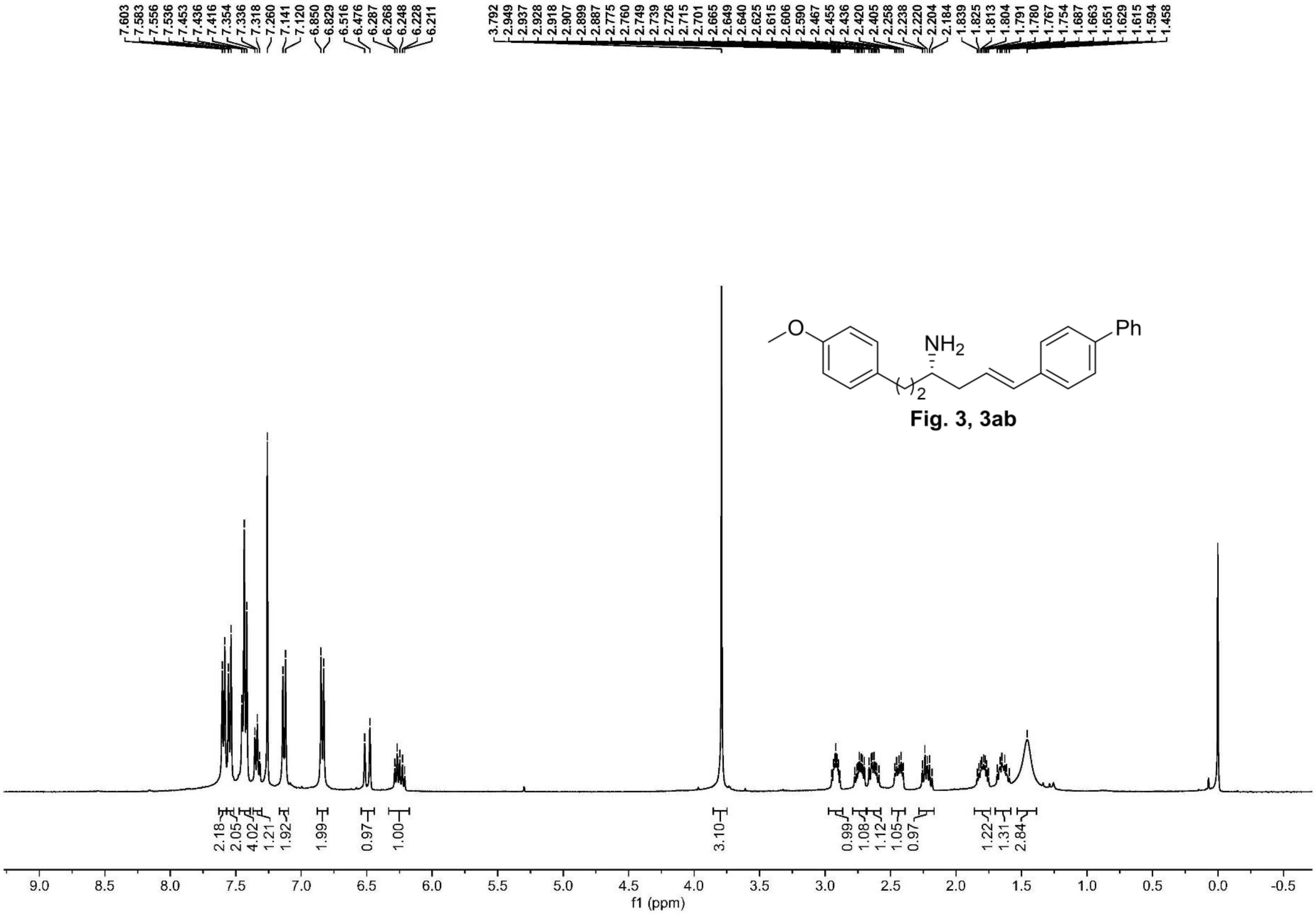

Fig. 3, 3ab

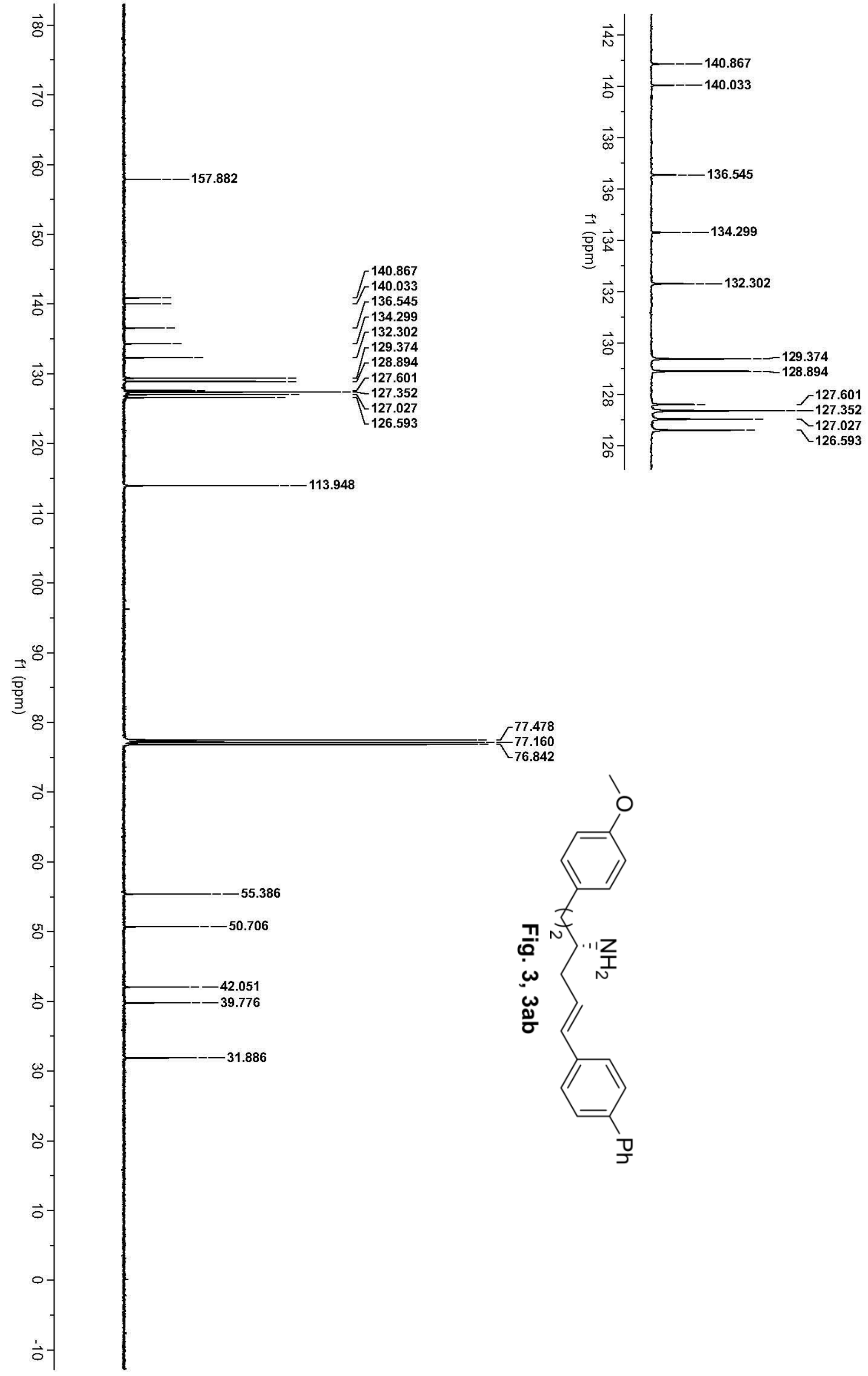

157.882
140.867
140.033
136.545
134.299
132.302
129.374
128.894
127.601
127.352
127.027
126.593
113.948
77.478
77.160
76.842
55.386
50.706
42.051
39.776
31.886
f1 (ppm)
NH2
O
Ph
2

Fig. 3, 3ab

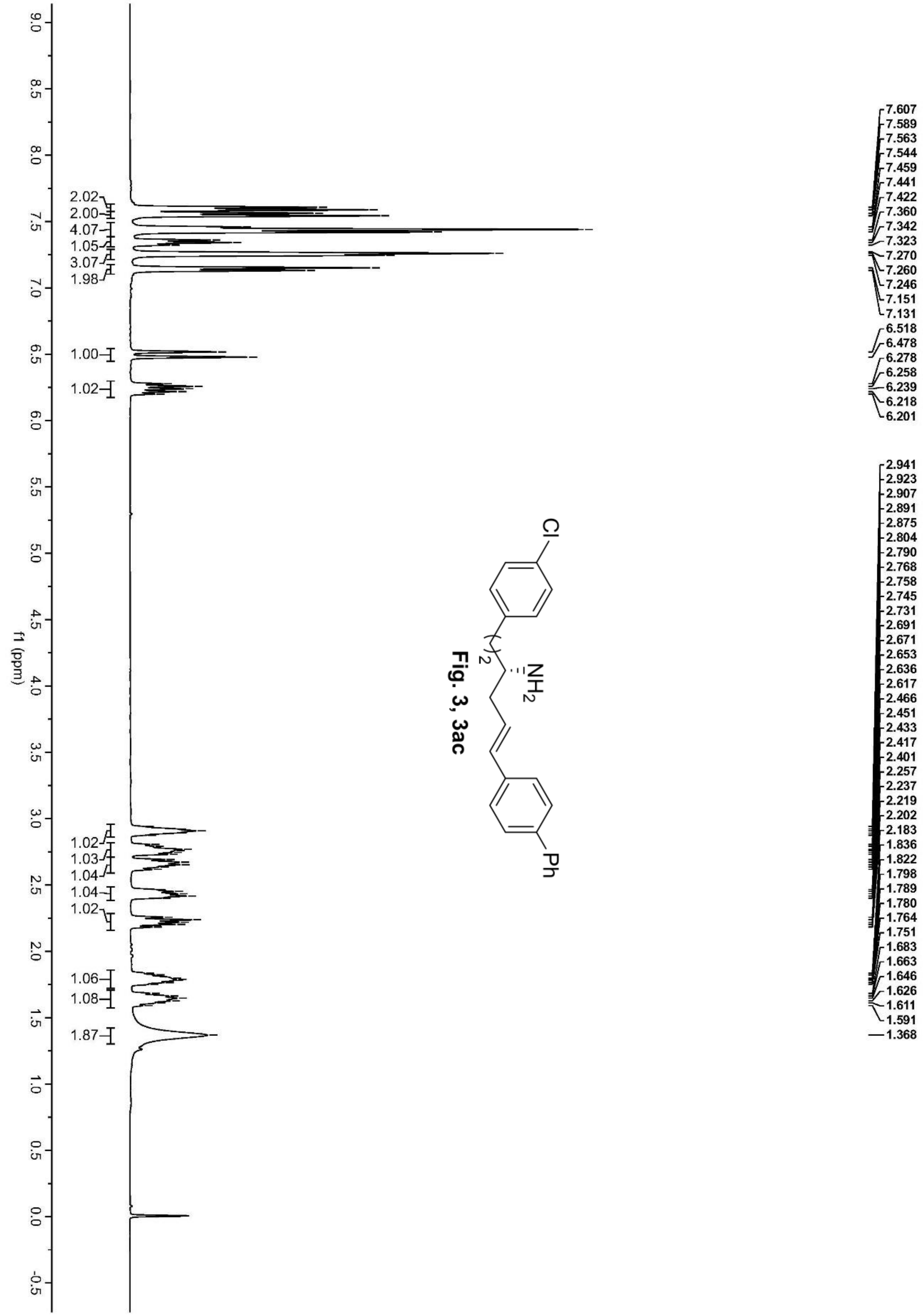
7.607
7.589
7.563
7.544
7.459
7.441
7.422
7.360
7.342
7.323
7.270
7.260
7.246
7.151
7.131
6.518
6.478
6.278
6.258
6.239
6.218
6.201
2.941
2.923
2.907
2.891
2.875
2.804
2.790
2.768
2.758
2.745
2.731
2.691
2.671
2.653
2.636
2.617
2.466
2.451
2.433
2.417
2.401
2.257
2.237
2.219
2.202
2.183
1.836
1.822
1.798
1.789
1.780
1.764
1.751
1.683
1.663
1.646
1.626
1.611
1.591
1.368
f1 (ppm)
Cl
NH2
Ph

Fig. 3, 3ac

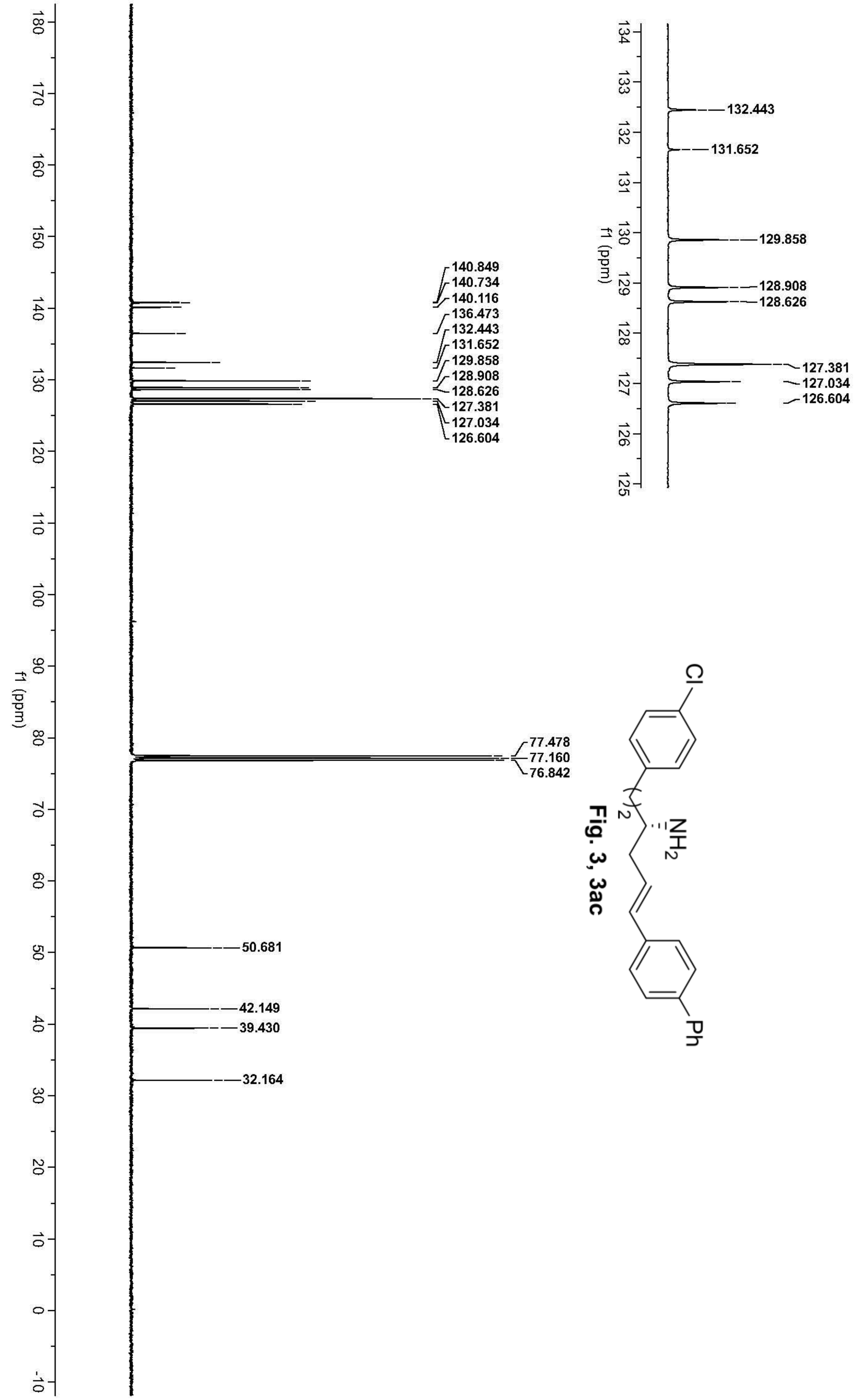
132.443
131.652
129.858
128.908
128.626
127.381
127.034
126.604
f1 (ppm)
140.849
140.734
140.116
136.473
77.478
77.160
76.842
50.681
42.149
39.430
32.164
Cl
NH2
Ph
f1 (ppm)
Fig. 3, 3ac

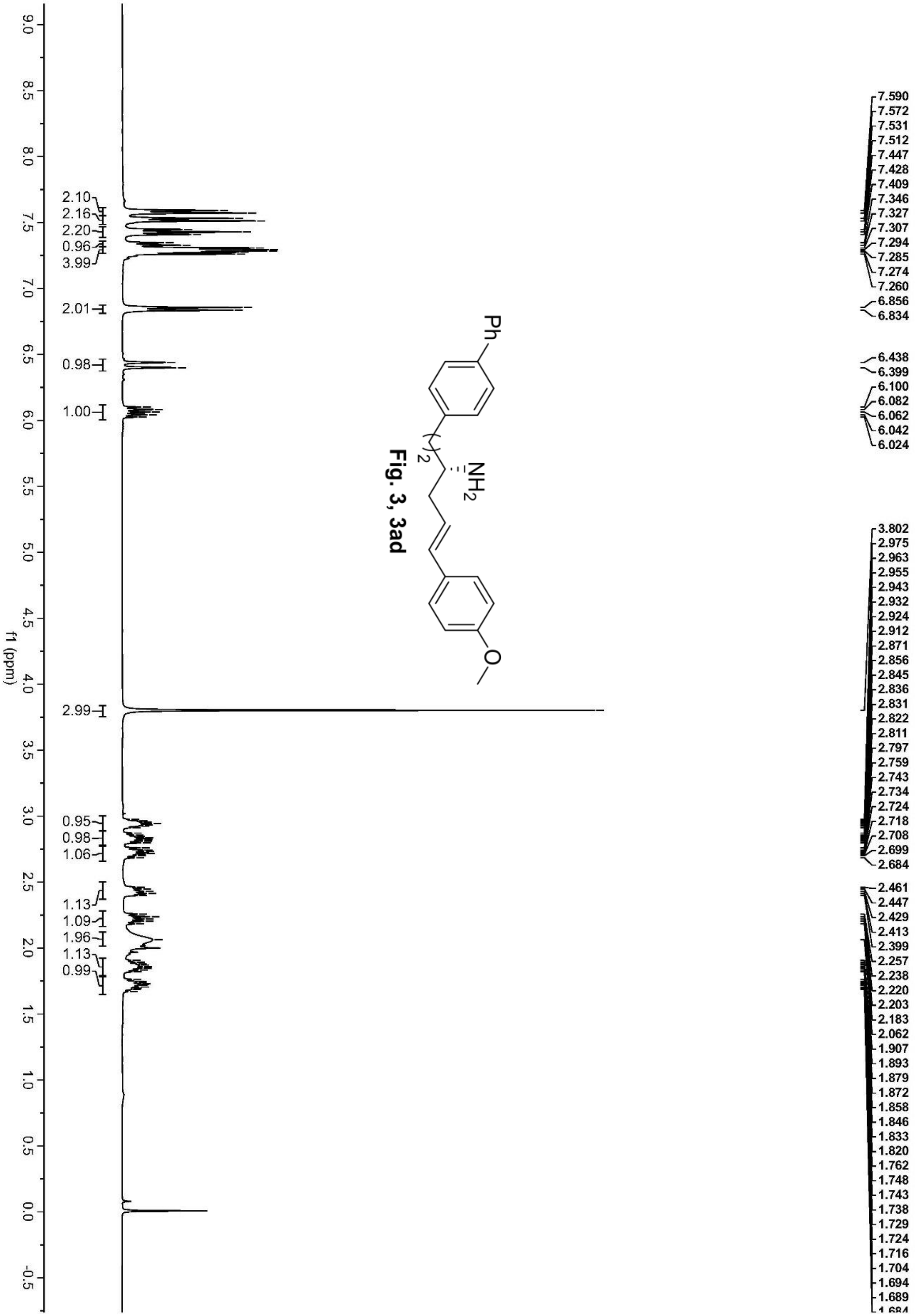
9.0
8.5
8.0
7.5
7.0
6.5
6.0
5.5
5.0
4.5
4.0
3.5
3.0
2.5
2.0
1.5
1.0
0.5
0.0
-0.5
f1 (ppm)
2.10
2.16
2.20
0.96
3.99
2.01
0.98
1.00
2.99
0.95
0.98
1.06
1.13
1.09
1.96
1.13
0.99
Ph
NH2
2
7.590
7.572
7.531
7.512
7.447
7.428
7.409
7.346
7.327
7.307
7.294
7.285
7.274
7.260
6.856
6.834
6.438
6.399
6.100
6.082
6.062
6.042
6.024
3.802
2.975
2.963
2.955
2.943
2.932
2.924
2.912
2.871
2.856
2.845
2.836
2.831
2.822
2.811
2.797
2.759
2.743
2.734
2.724
2.718
2.708
2.699
2.684
2.461
2.447
2.429
2.413
2.399
2.257
2.238
2.220
2.203
2.183
2.062
1.907
1.893
1.879
1.872
1.858
1.846
1.833
1.820
1.762
1.748
1.743
1.738
1.729
1.724
1.716
1.704
1.694
1.689

Fig. 3, 3ad

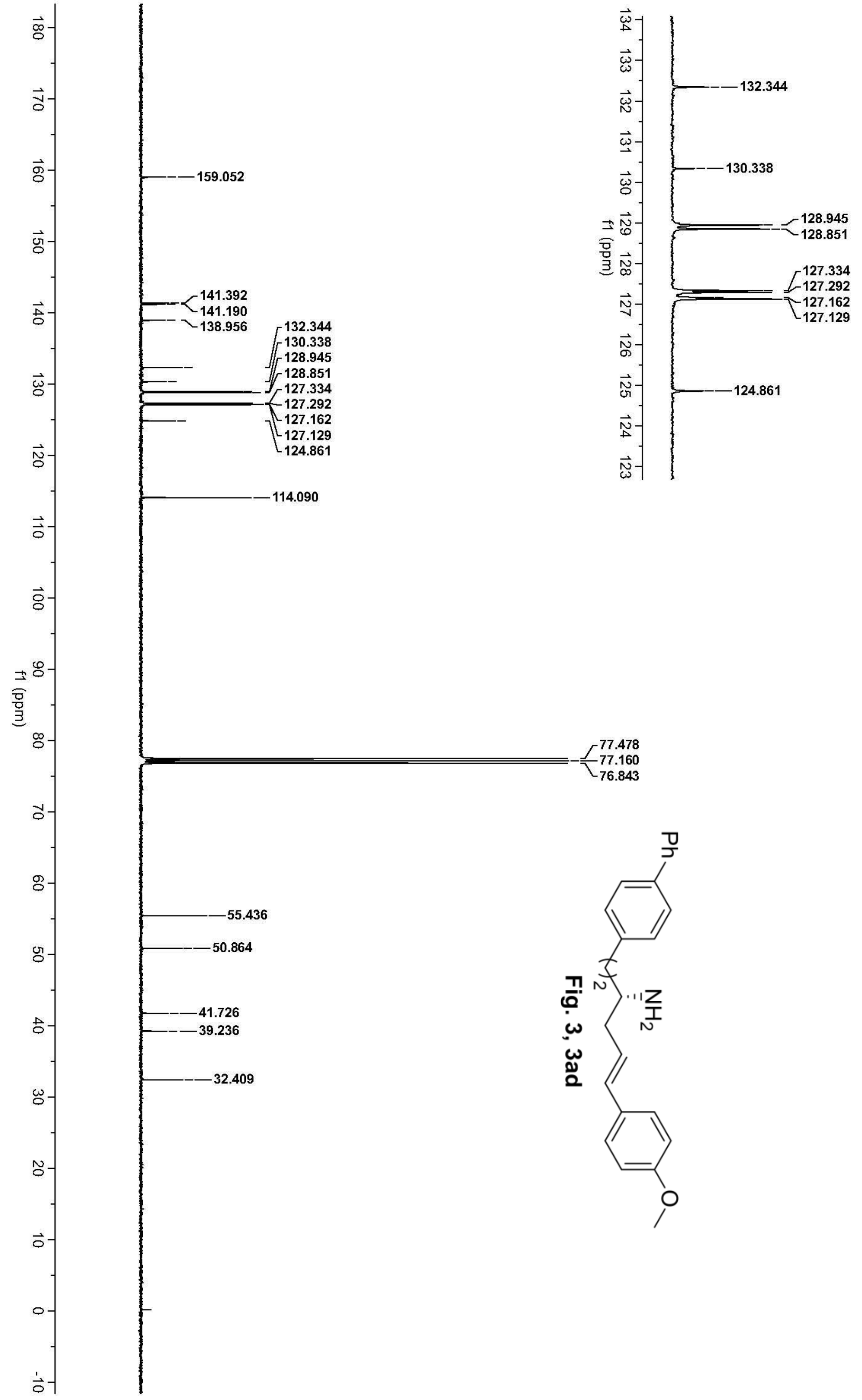
159.052
141.392
141.190
138.956
132.344
130.338
128.945
128.851
127.334
127.292
127.162
127.129
124.861
114.090
77.478
77.160
76.843
55.436
50.864
41.726
39.236
32.409
f1 (ppm)
Ph
NH2
2
O

Fig. 3, 3ad

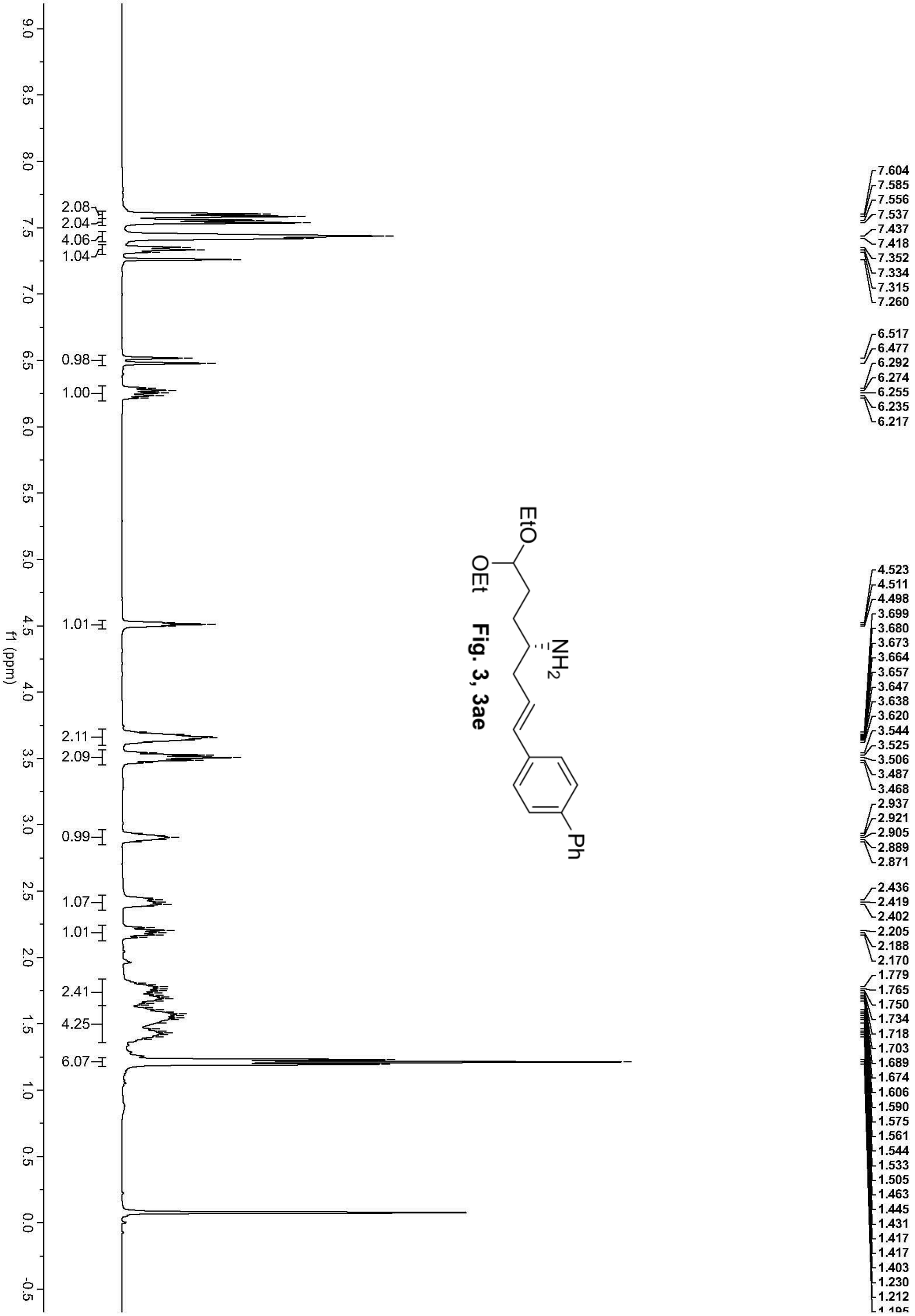
7.604
7.585
7.556
7.537
7.437
7.418
7.352
7.334
7.315
7.260
6.517
6.477
6.292
6.274
6.255
6.235
6.217
4.523
4.511
4.498
3.699
3.680
3.673
3.664
3.657
3.647
3.638
3.620
3.544
3.525
3.506
3.487
3.468
2.937
2.921
2.905
2.889
2.871
2.436
2.419
2.402
2.205
2.188
2.170
1.779
1.765
1.750
1.734
1.718
1.703
1.689
1.674
1.606
1.590
1.575
1.561
1.544
1.533
1.505
1.463
1.445
1.431
1.417
1.417
1.403
1.230
1.212
2.08
2.04
4.06
1.04
0.98
1.00
1.01
2.11
2.09
0.99
1.07
1.01
2.41
4.25
6.07
f1 (ppm)
EtO
OEt
NH2
Ph

Fig. 3, 3ae

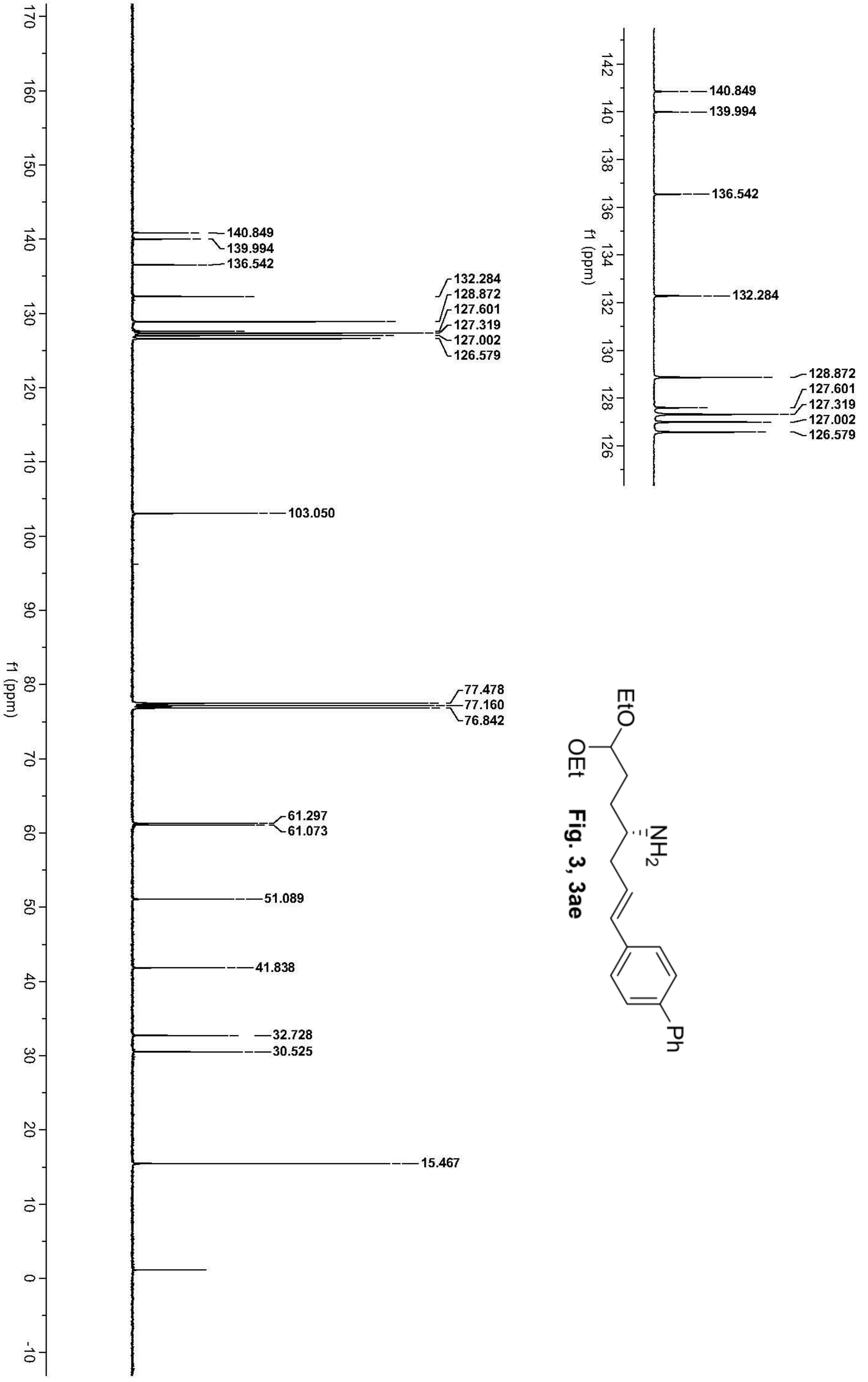
140.849
139.994
136.542
132.284
128.872
127.601
127.319
127.002
126.579
103.050
77.478
77.160
76.842
61.297
61.073
51.089
41.838
32.728
30.525
15.467
f1 (ppm)
EtO
OEt
NH2
Ph

Fig. 3, 3ae

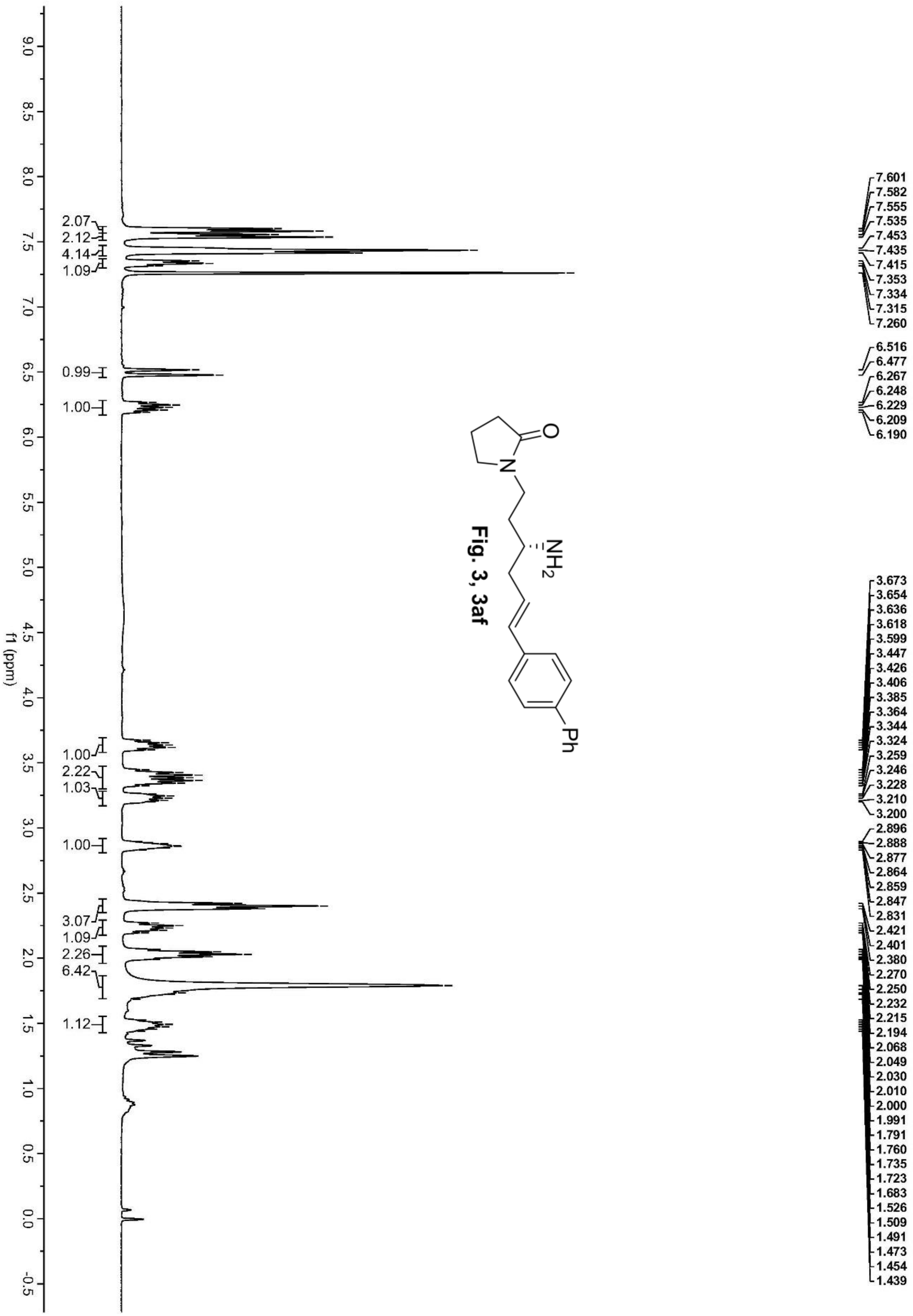

7.601
7.582
7.555
7.535
7.453
7.435
7.415
7.353
7.334
7.315
7.260
6.516
6.477
6.267
6.248
6.229
6.209
6.190
3.673
3.654
3.636
3.618
3.599
3.447
3.426
3.406
3.385
3.364
3.344
3.324
3.259
3.246
3.228
3.210
3.200
2.896
2.888
2.877
2.864
2.859
2.847
2.831
2.421
2.401
2.380
2.270
2.250
2.232
2.215
2.194
2.068
2.049
2.030
2.010
2.000
1.991
1.791
1.760
1.735
1.723
1.683
1.526
1.509
1.491
1.473
1.454
1.439
2.07
2.12
4.14
1.09
0.99
1.00
1.00
2.22
1.03
1.00
3.07
1.09
2.26
6.42
1.12
O
N
NH2
Ph
9.0
8.5
8.0
7.5
7.0
6.5
6.0
5.5
5.0
4.5
4.0
3.5
3.0
2.5
2.0
1.5
1.0
0.5
0.0
-0.5
f1 (ppm)

Fig. 3, 3af

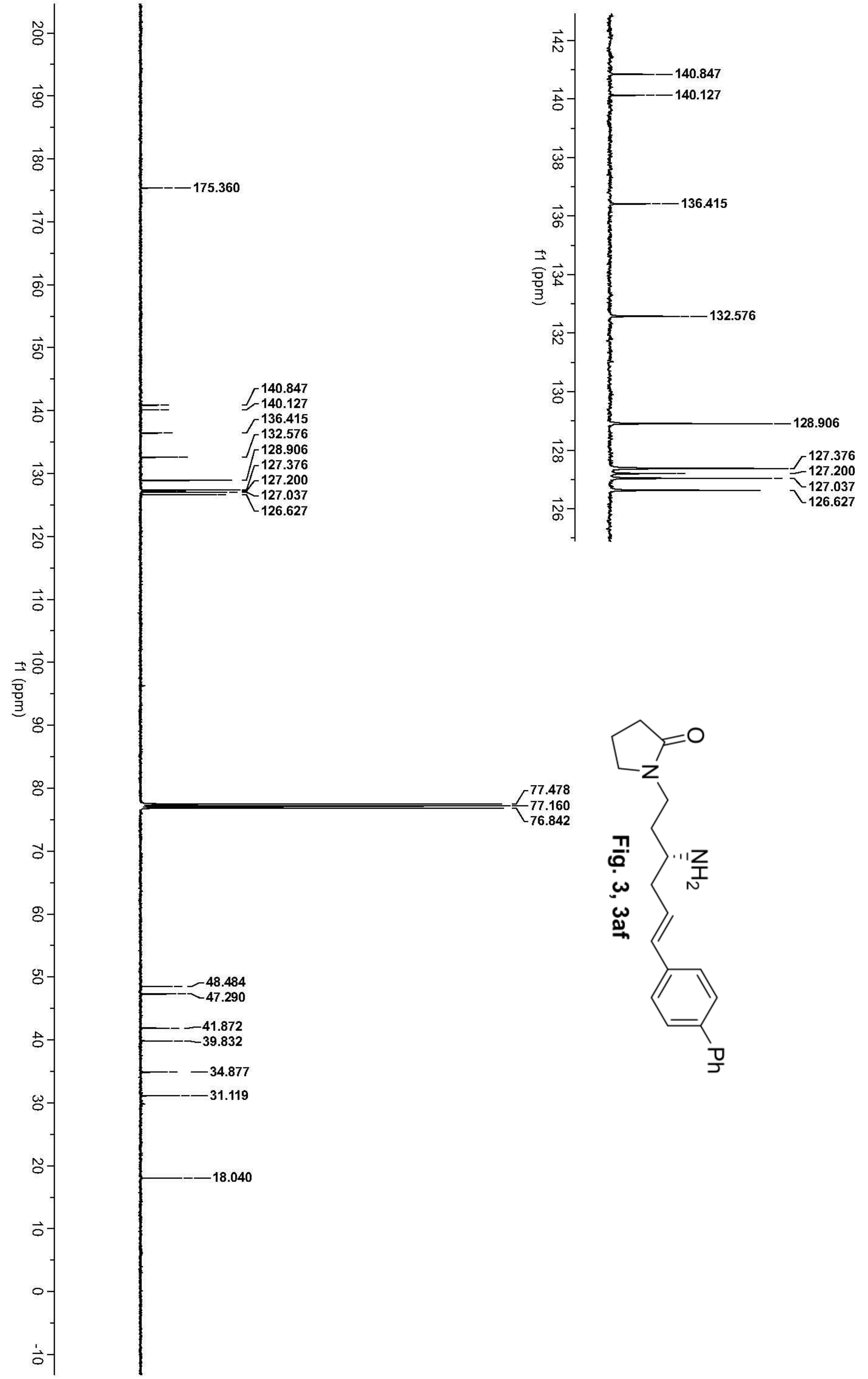
175.360
140.847
140.127
136.415
132.576
128.906
127.376
127.200
127.037
126.627
77.478
77.160
76.842
48.484
47.290
41.872
39.832
34.877
31.119
18.040
f1 (ppm)
O
N
NH2
Ph

Fig. 3, 3af

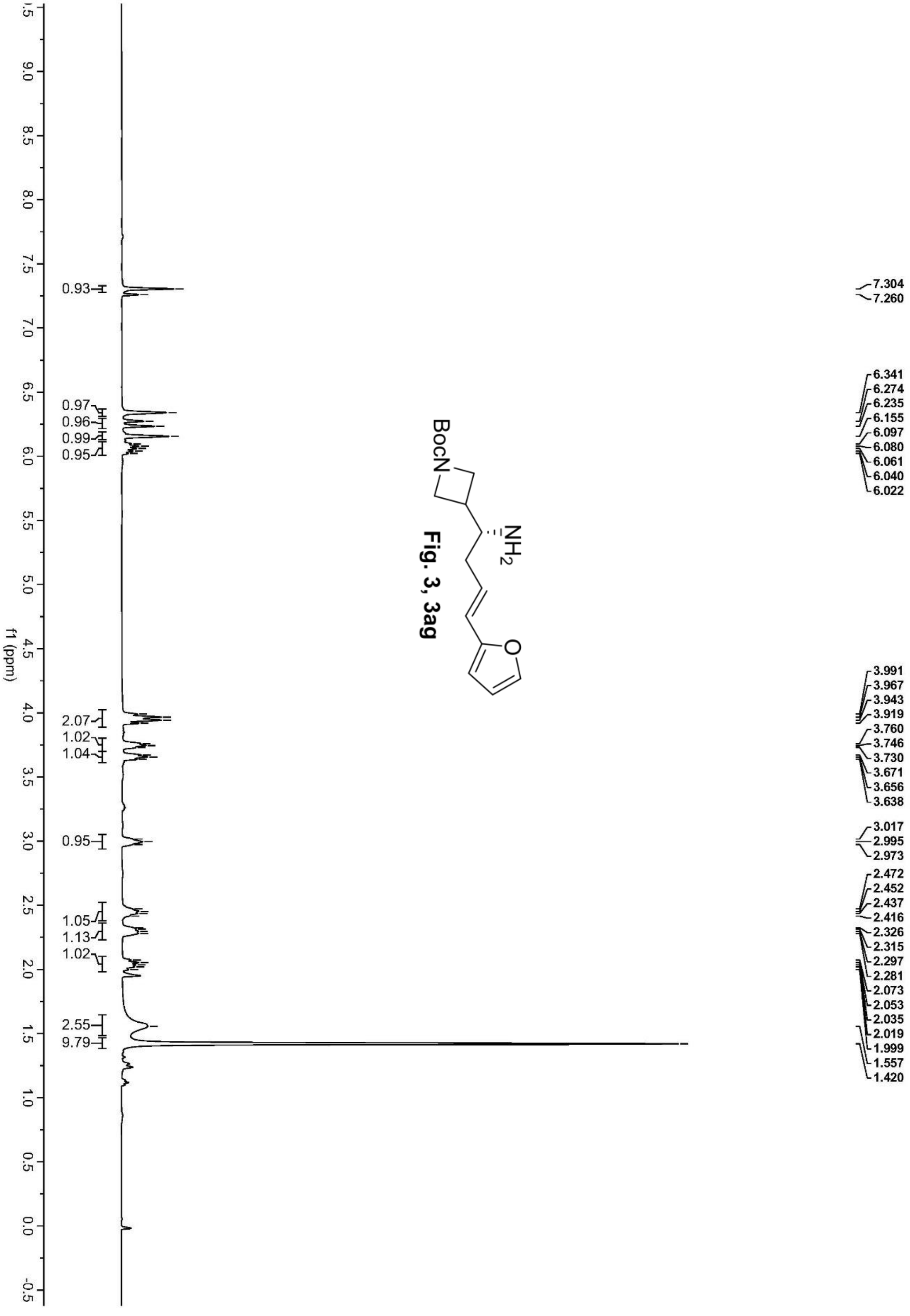
BocN
NH2
7.304
7.260
6.341
6.274
6.235
6.155
6.097
6.080
6.061
6.040
6.022
3.991
3.967
3.943
3.919
3.760
3.746
3.730
3.671
3.656
3.638
3.017
2.995
2.973
2.472
2.452
2.437
2.416
2.326
2.315
2.297
2.281
2.073
2.053
2.035
2.019
1.999
1.557
1.420
0.93
0.97
0.96
0.99
0.95
2.07
1.02
1.04
0.95
1.05
1.13
1.02
2.55
9.79
f1 (ppm)

Fig. 3, 3ag

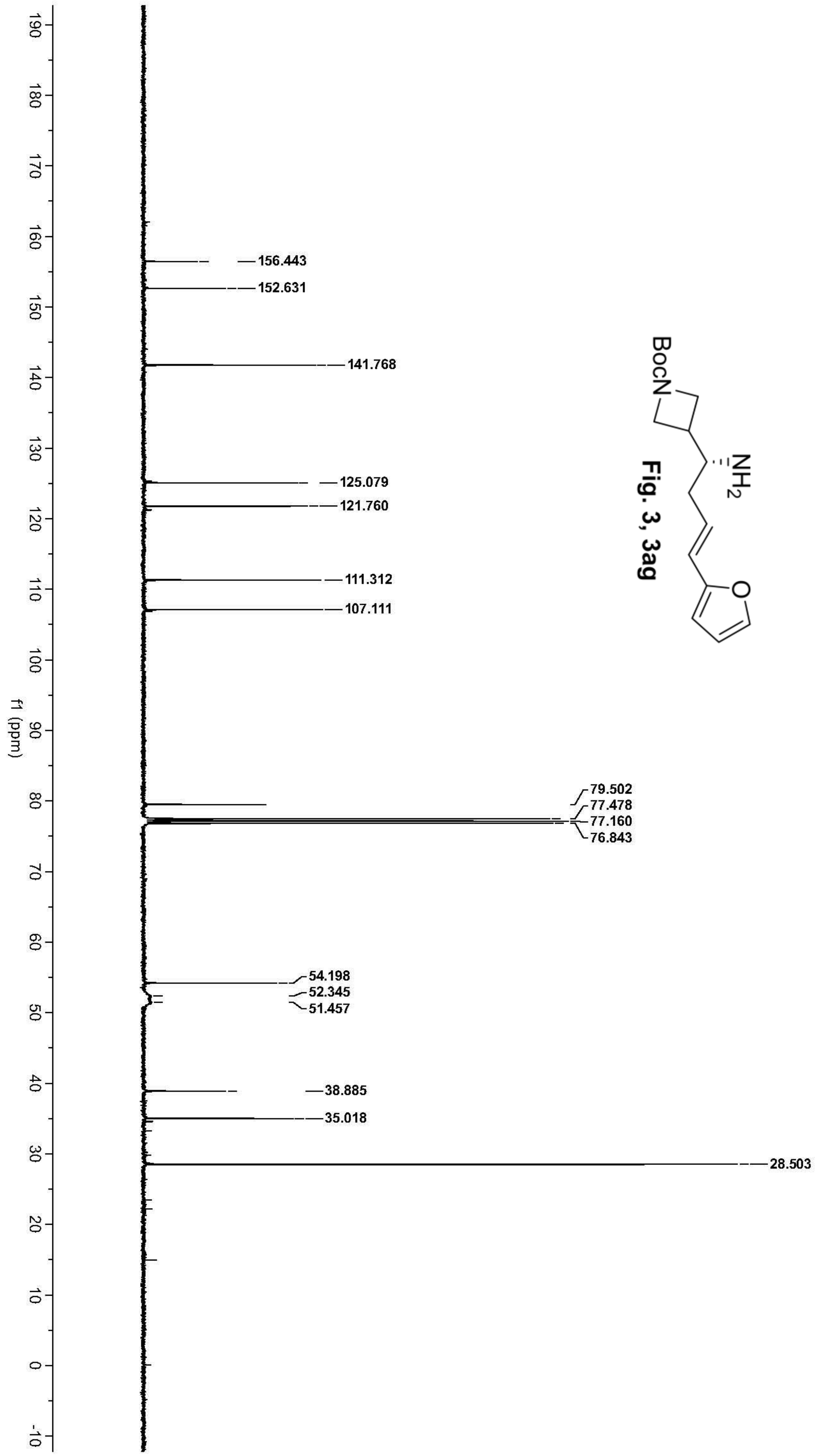
156.443
152.631
141.768
125.079
121.760
111.312
107.111
79.502
77.478
77.160
76.843
54.198
52.345
51.457
38.885
35.018
28.503
f1 (ppm)
BocN
NH2
O

Fig. 3, 3ag

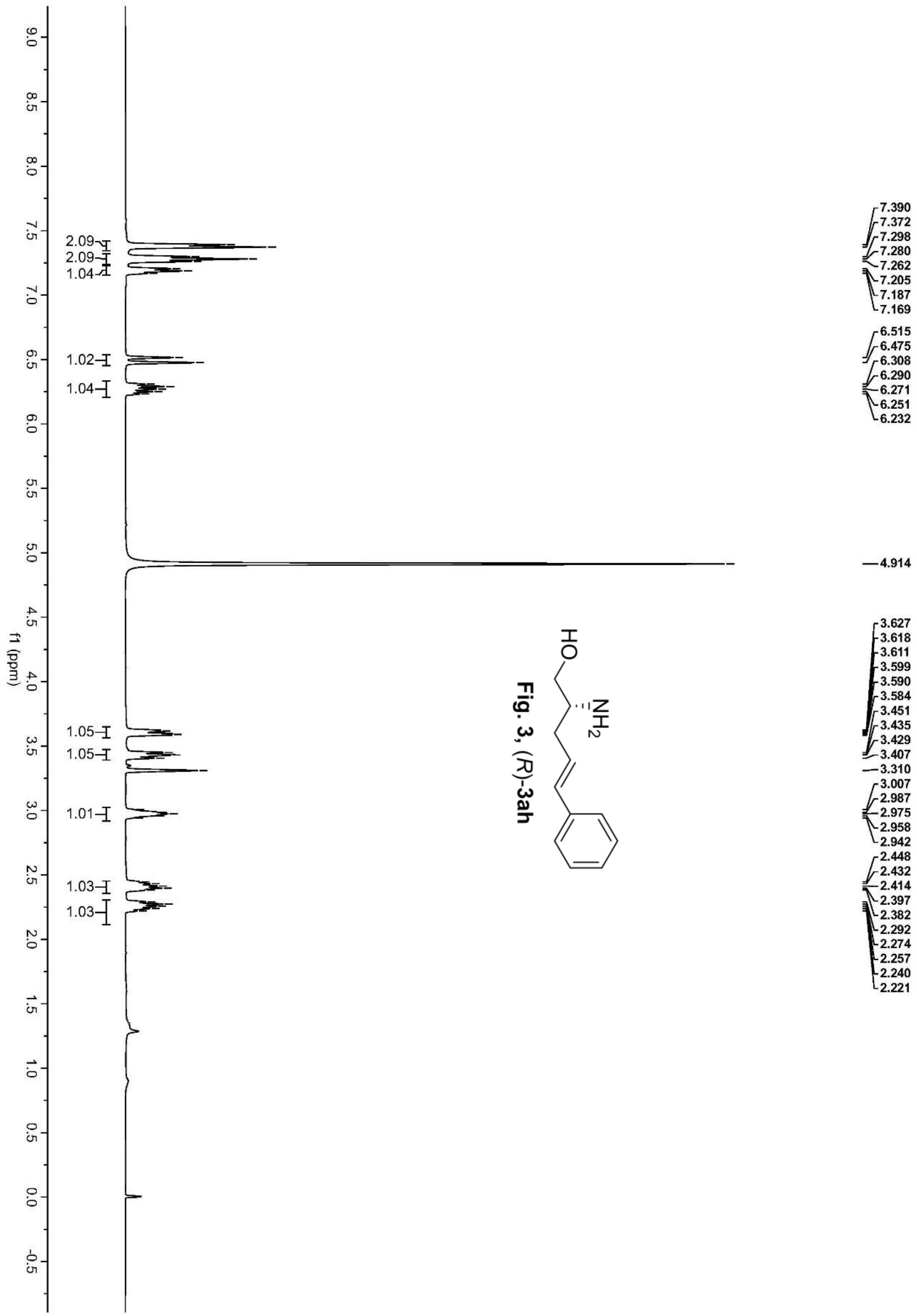

7.390
7.372
7.298
7.280
7.262
7.205
7.187
7.169
6.515
6.475
6.308
6.290
6.271
6.251
6.232
4.914
3.627
3.618
3.611
3.599
3.590
3.584
3.451
3.435
3.429
3.407
3.310
3.007
2.987
2.975
2.958
2.942
2.448
2.432
2.414
2.397
2.382
2.292
2.274
2.257
2.240
2.221
2.09
2.09
1.04
1.02
1.04
1.05
1.05
1.01
1.03
1.03
HO
NH2
f1 (ppm)

Fig. 3, (*R*)-3ah

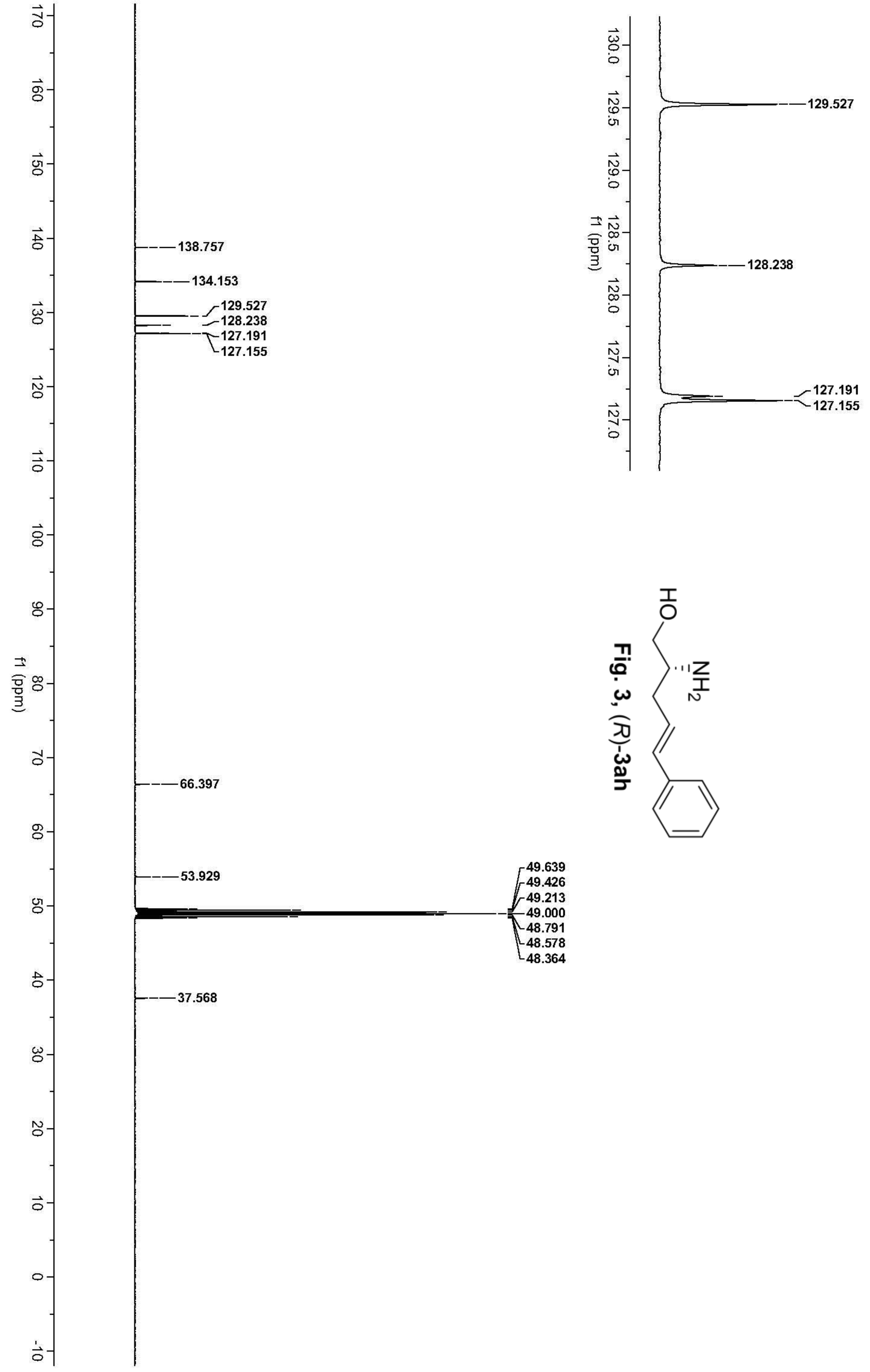
138.757
134.153
129.527
128.238
127.191
127.155
66.397
53.929
49.639
49.426
49.213
49.000
48.791
48.578
48.364
37.568
f1 (ppm)
HO
NH_2

Fig. 3, (*R*)-3ah

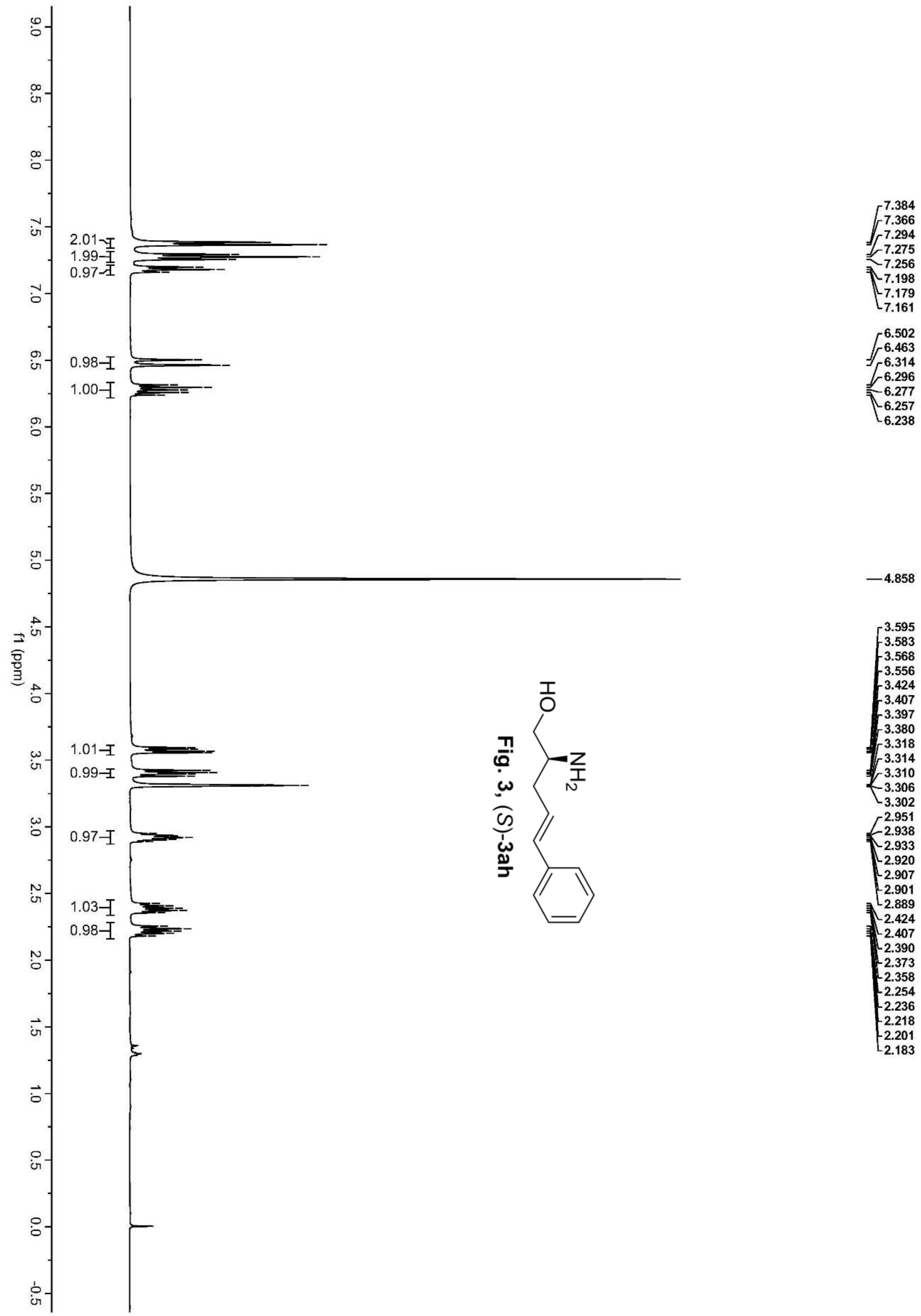

f1 (ppm)
2.01
1.99
0.97
0.98
1.00
1.01
0.99
0.97
1.03
0.98
7.384
7.366
7.294
7.275
7.256
7.198
7.179
7.161
6.502
6.463
6.314
6.296
6.277
6.257
6.238
4.858
3.595
3.583
3.568
3.556
3.424
3.407
3.397
3.380
3.318
3.314
3.310
3.306
3.302
2.951
2.938
2.933
2.920
2.907
2.901
2.889
2.424
2.407
2.390
2.373
2.358
2.254
2.236
2.218
2.201
2.183
HO
NH2

Fig. 3, (S)-3ah

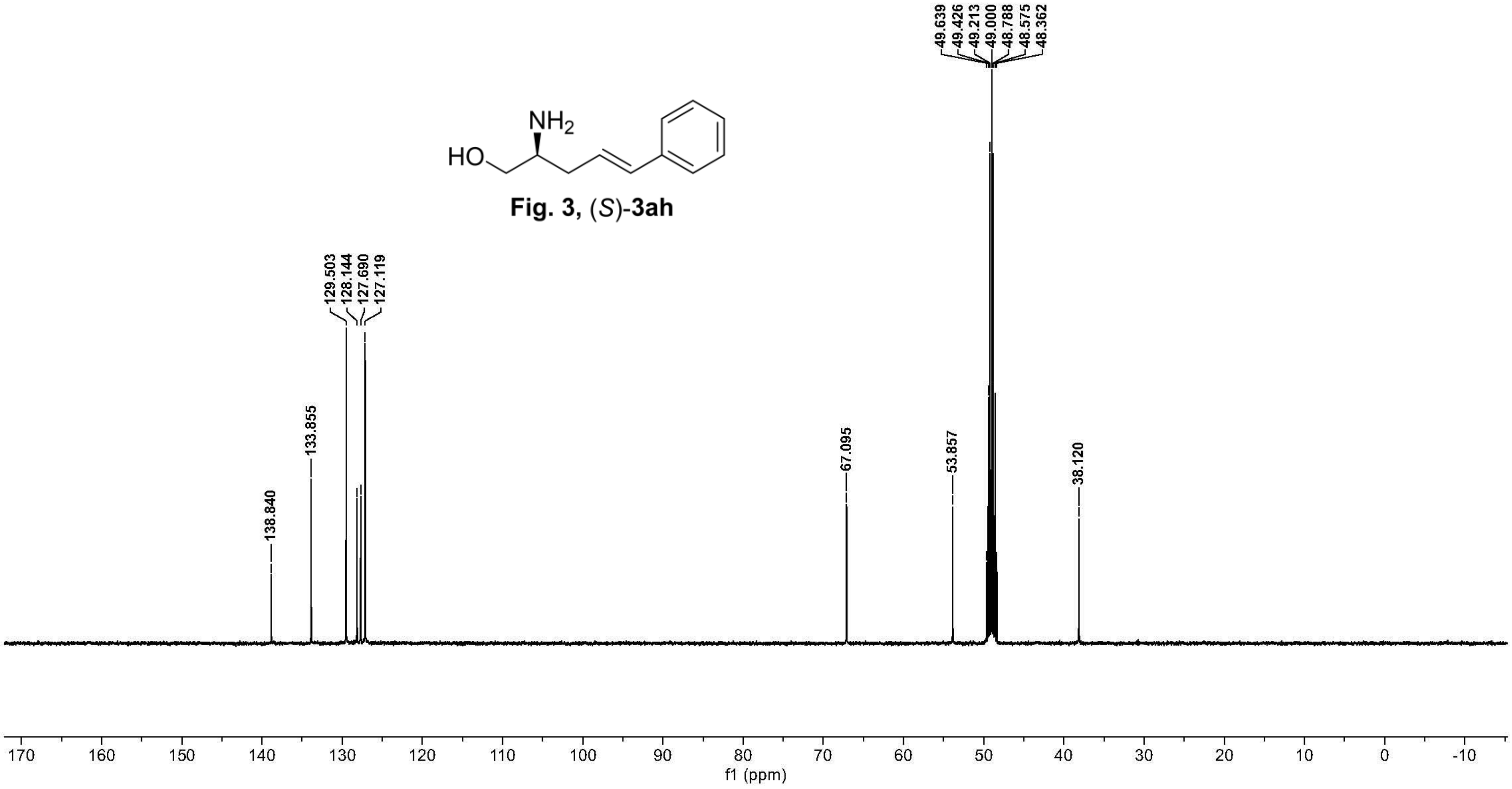
138.840
133.855
129.503
128.144
127.690
127.119
67.095
53.857
49.639
49.426
49.213
49.000
48.788
48.575
48.362
38.120
NH_2
HO
170 160 150 140 130 120 110 100 90 80 70 60 50 40 30 20 10 0 -10
f1 (ppm)

Fig. 3, (*S*)-**3ah**

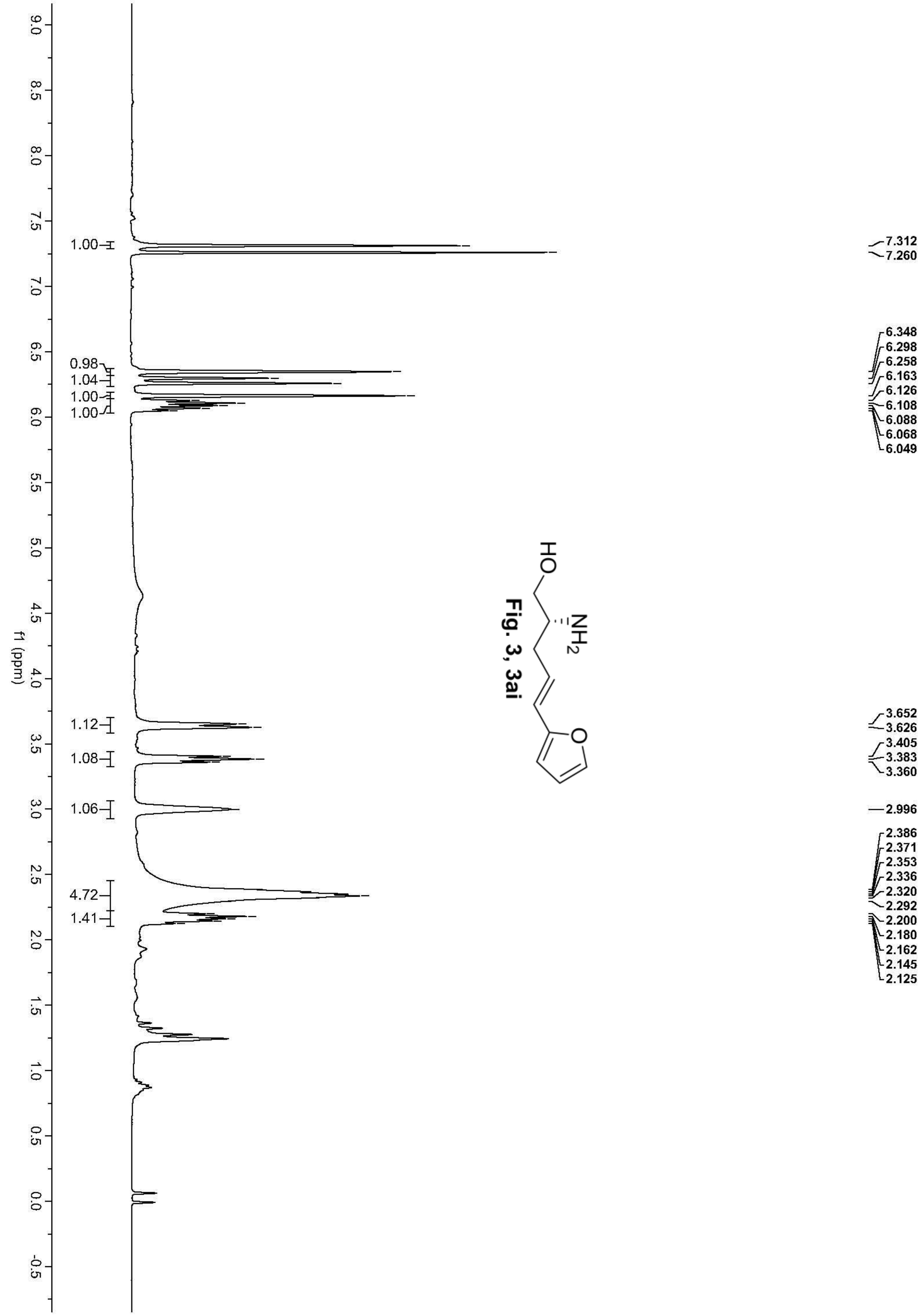
7.312
7.260
6.348
6.298
6.258
6.163
6.126
6.108
6.088
6.068
6.049
3.652
3.626
3.405
3.383
3.360
2.996
2.386
2.371
2.353
2.336
2.320
2.292
2.200
2.180
2.162
2.145
2.125
1.00
0.98
1.04
1.00
1.00
1.12
1.08
1.06
4.72
1.41
HO
NH2
O
9.0
8.5
8.0
7.5
7.0
6.5
6.0
5.5
5.0
4.5
4.0
3.5
3.0
2.5
2.0
1.5
1.0
0.5
0.0
-0.5
f1 (ppm)

Fig. 3, 3ai

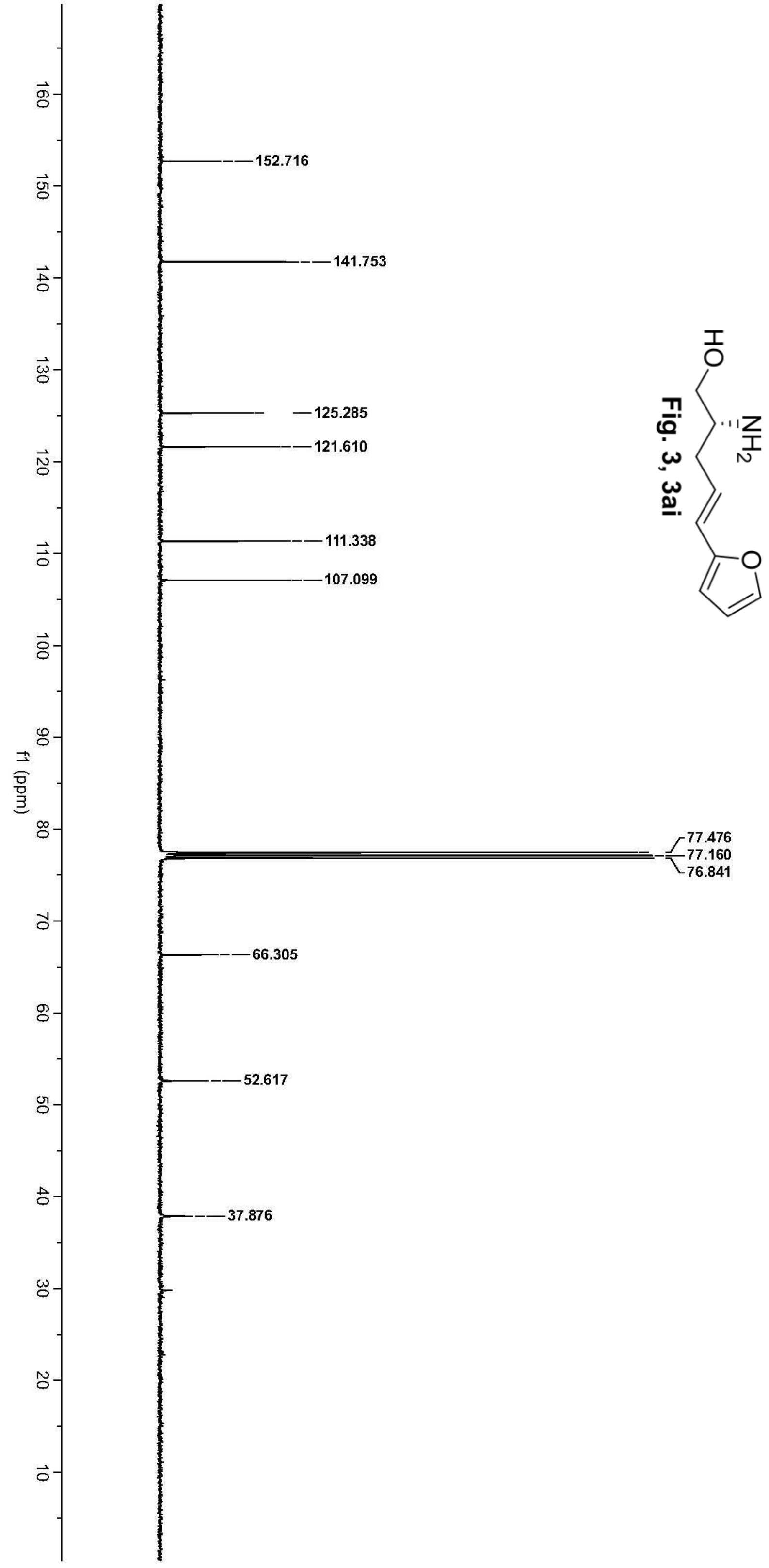
152.716
141.753
125.285
121.610
111.338
107.099
77.476
77.160
76.841
66.305
52.617
37.876
f1 (ppm)
HO
NH2
O

Fig. 3, 3ai

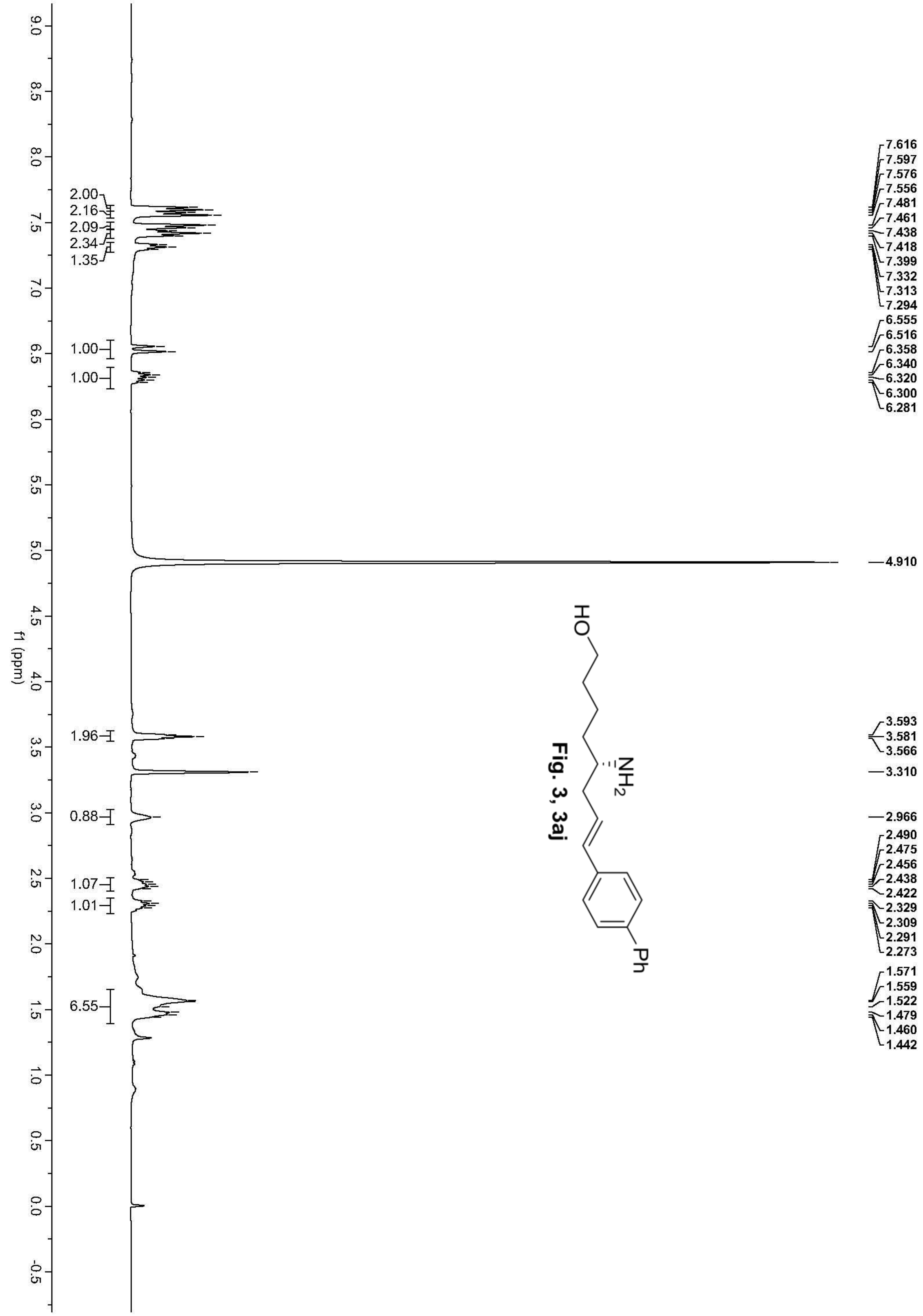

7.616
7.597
7.576
7.556
7.481
7.461
7.438
7.418
7.399
7.332
7.313
7.294
6.555
6.516
6.358
6.340
6.320
6.300
6.281
4.910
3.593
3.581
3.566
3.310
2.966
2.490
2.475
2.456
2.438
2.422
2.329
2.309
2.291
2.273
1.571
1.559
1.522
1.479
1.460
1.442
2.00
2.16
2.09
2.34
1.35
1.00
1.00
1.96
0.88
1.07
1.01
6.55
HO
NH2
Ph
f1 (ppm)

Fig. 3, 3aj

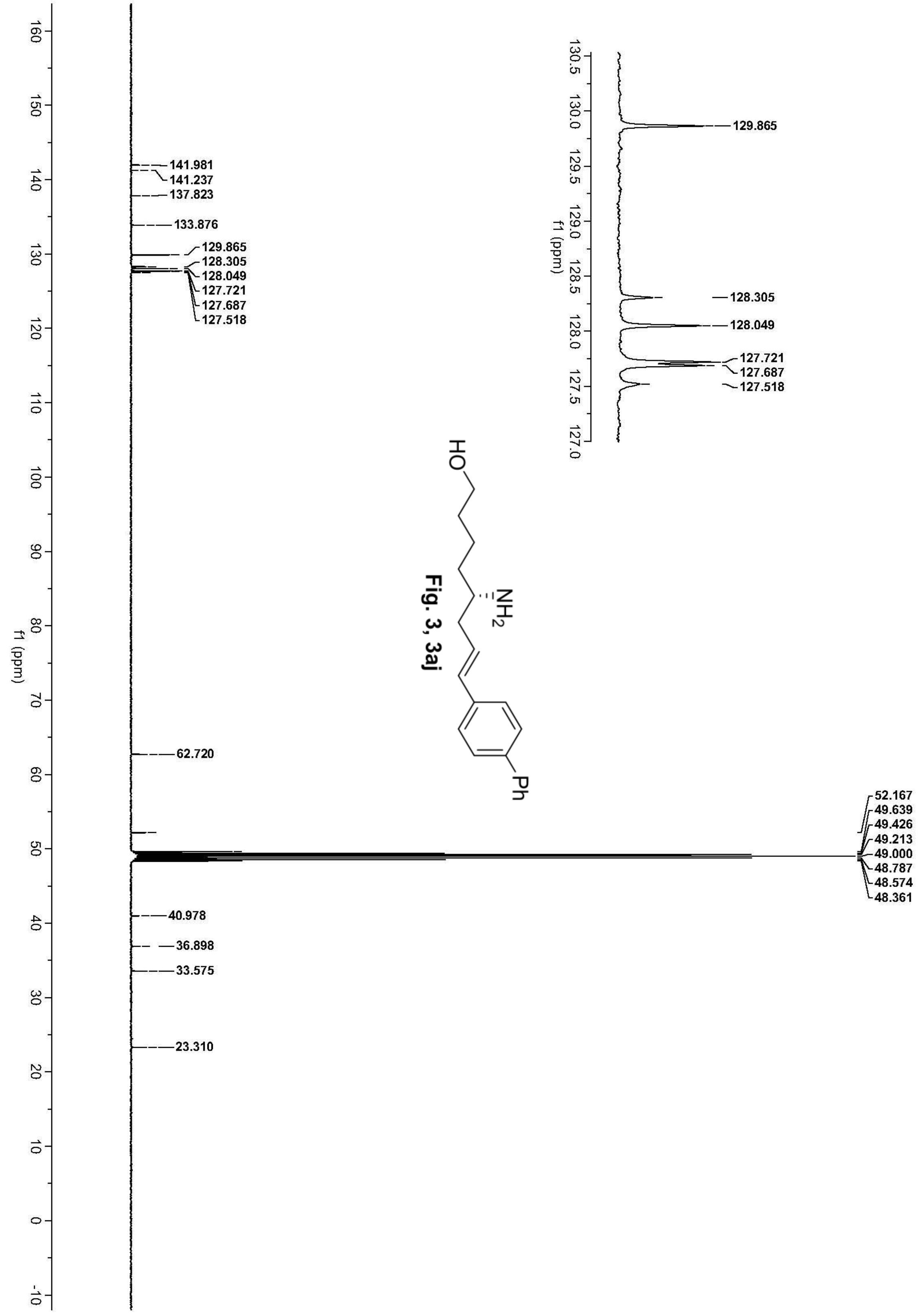

141.981
141.237
137.823
133.876
129.865
128.305
128.049
127.721
127.687
127.518
62.720
52.167
49.639
49.426
49.213
49.000
48.787
48.574
48.361
40.978
36.898
33.575
23.310
f1 (ppm)
HO
NH2
Ph

Fig. 3, 3aj

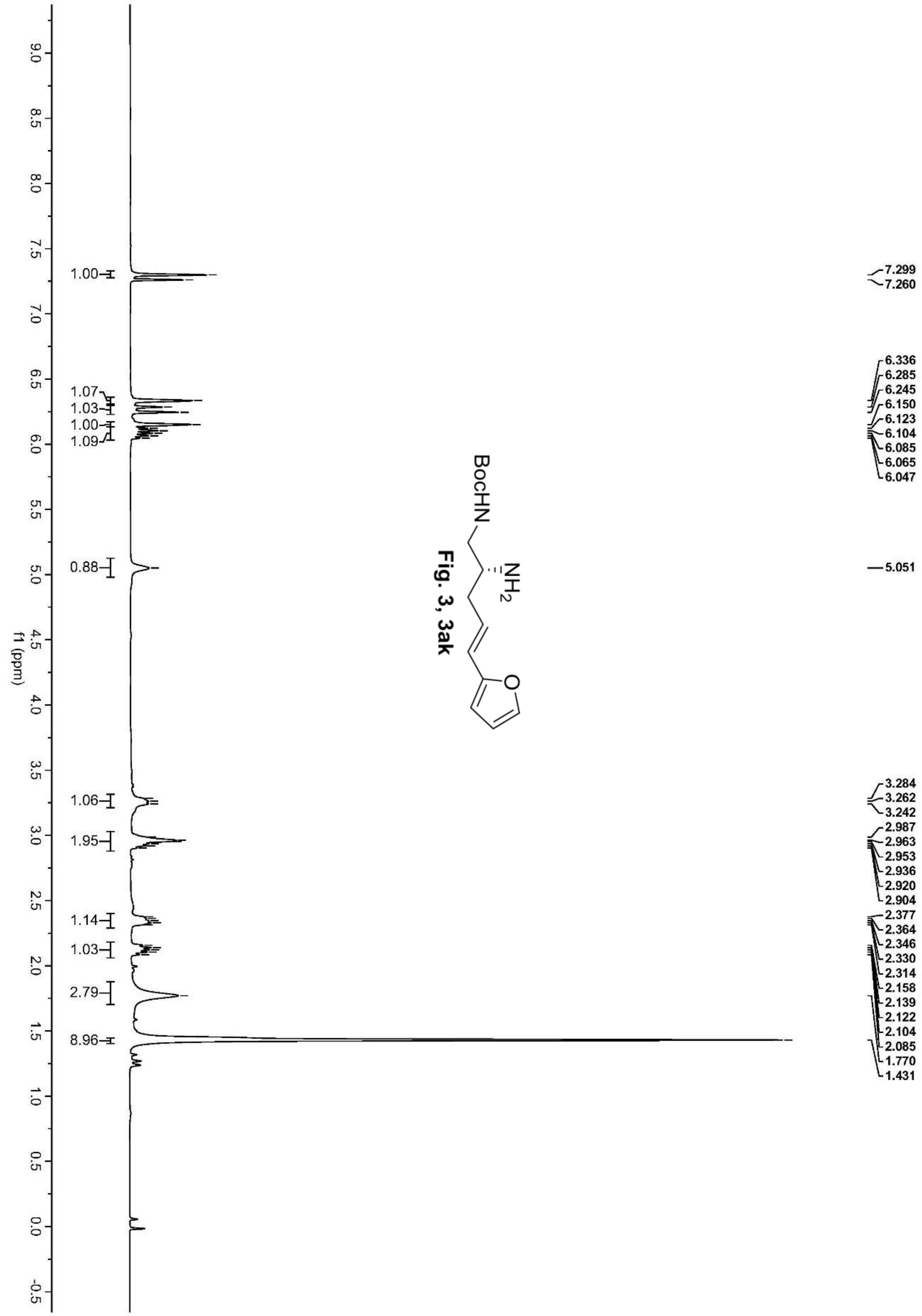

Fig. 3, 3ak

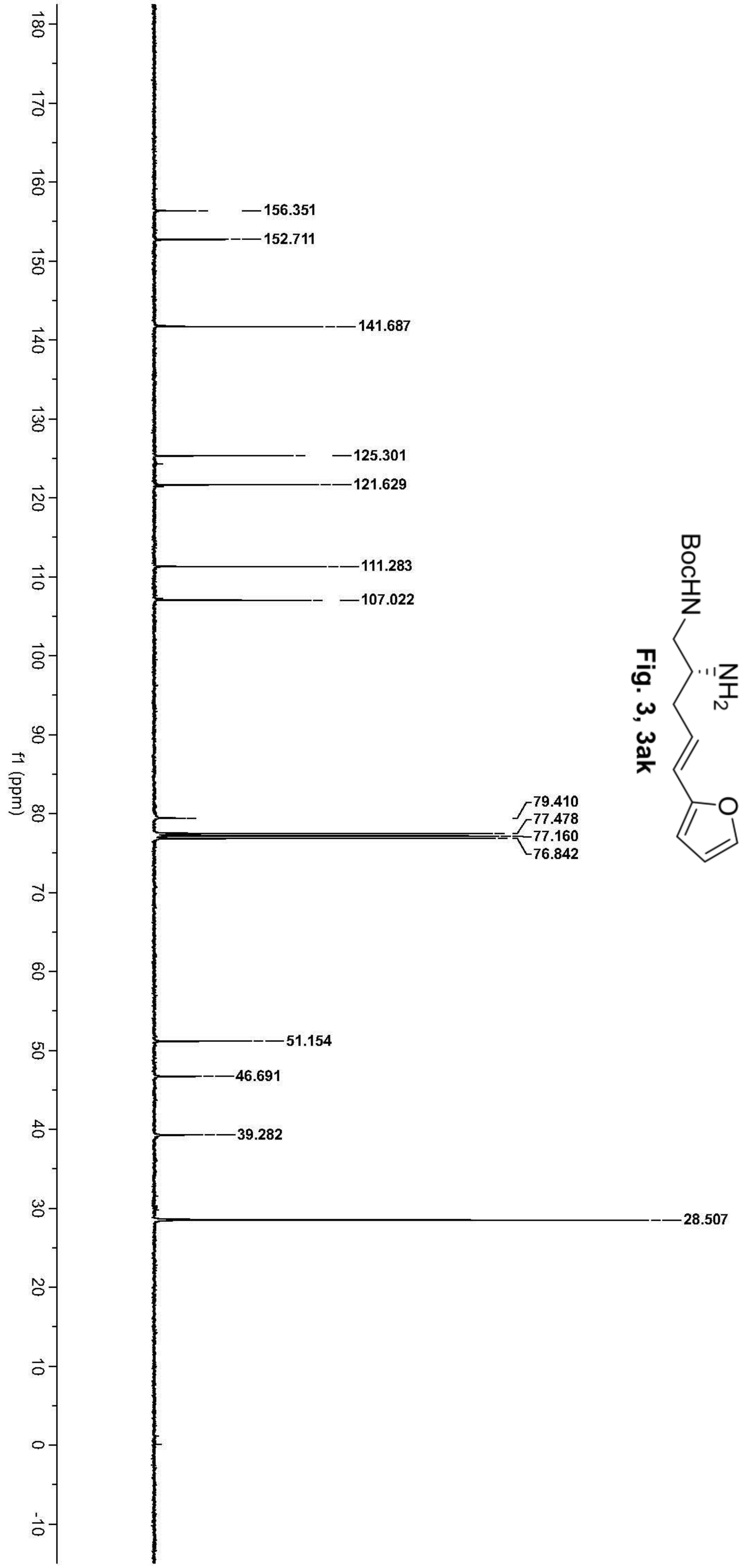
156.351
152.711
141.687
125.301
121.629
111.283
107.022
79.410
77.478
77.160
76.842
51.154
46.691
39.282
28.507
f1 (ppm)
BocHN
NH2
O

Fig. 3, 3ak

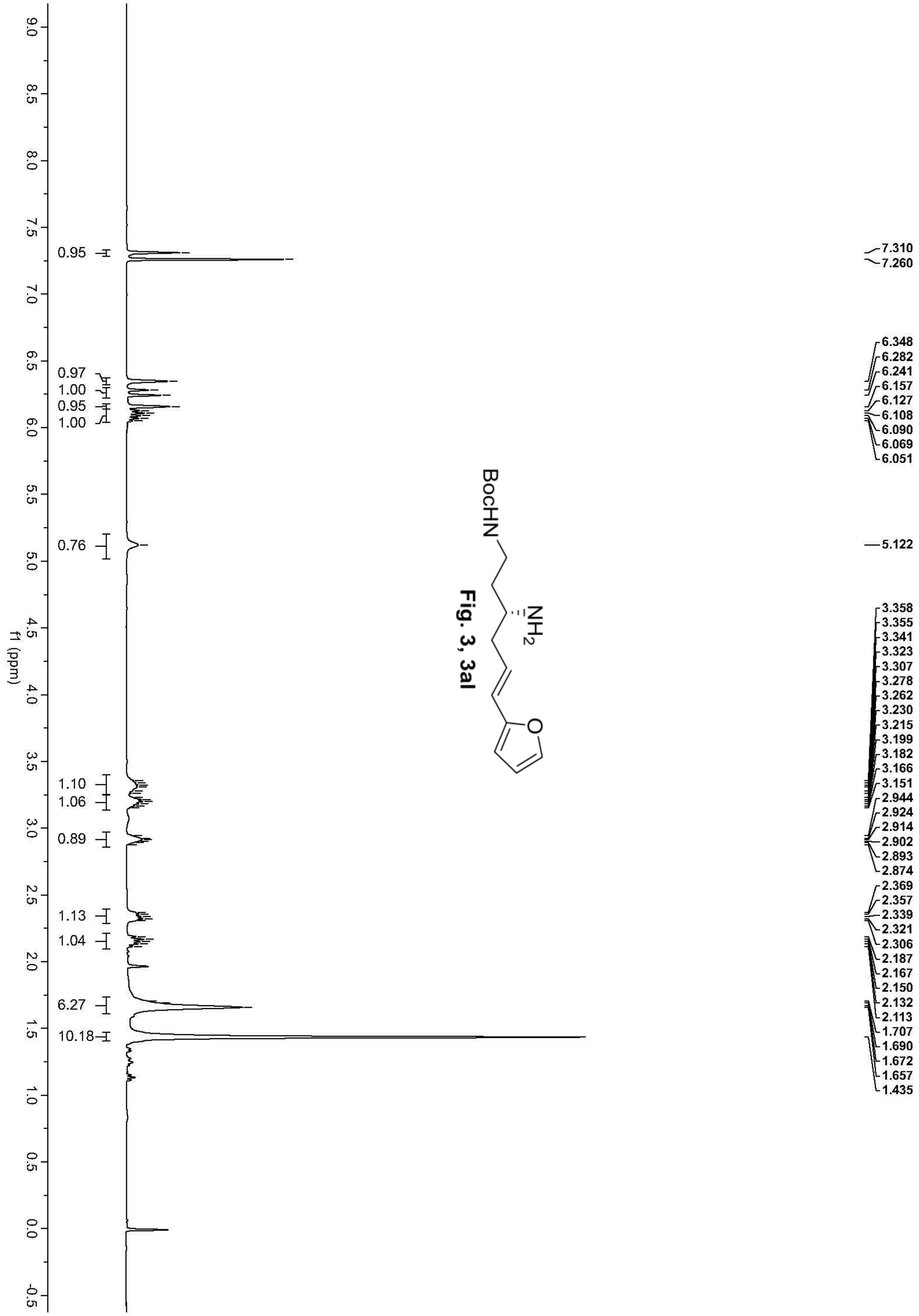
BocHN
NH2
7.310
7.260
6.348
6.282
6.241
6.157
6.127
6.108
6.090
6.069
6.051
5.122
3.358
3.355
3.341
3.323
3.307
3.278
3.262
3.230
3.215
3.199
3.182
3.166
3.151
2.944
2.924
2.914
2.902
2.893
2.874
2.369
2.357
2.339
2.321
2.306
2.187
2.167
2.150
2.132
2.113
1.707
1.690
1.672
1.657
1.435
0.95
0.97
1.00
0.95
1.00
0.76
1.10
1.06
0.89
1.13
1.04
6.27
10.18
f1 (ppm)

Fig. 3, 3al

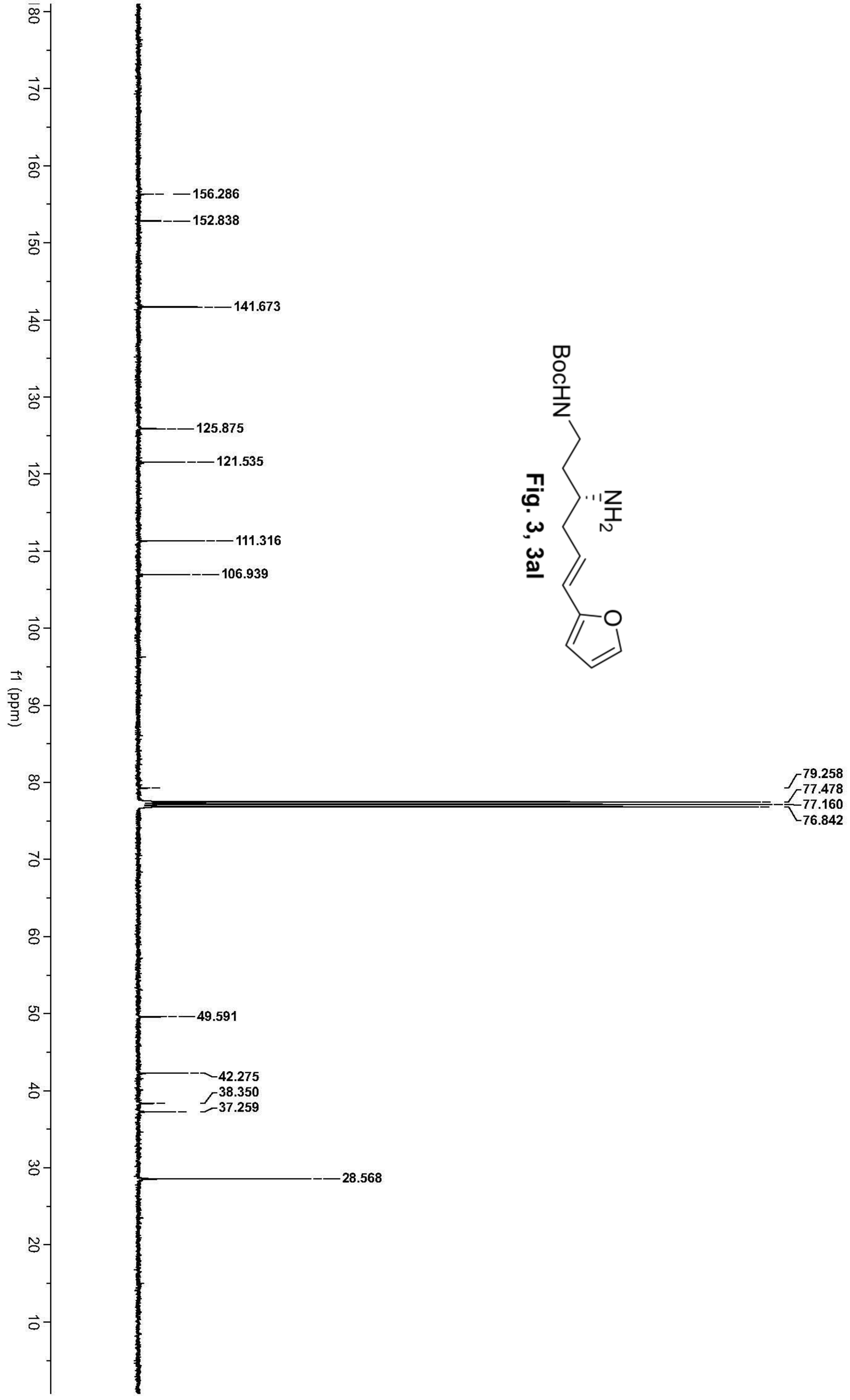
156.286
152.838
141.673
125.875
121.535
111.316
106.939
79.258
77.478
77.160
76.842
49.591
42.275
38.350
37.259
28.568
f1 (ppm)
BocHN
NH2
O

Fig. 3, 3al

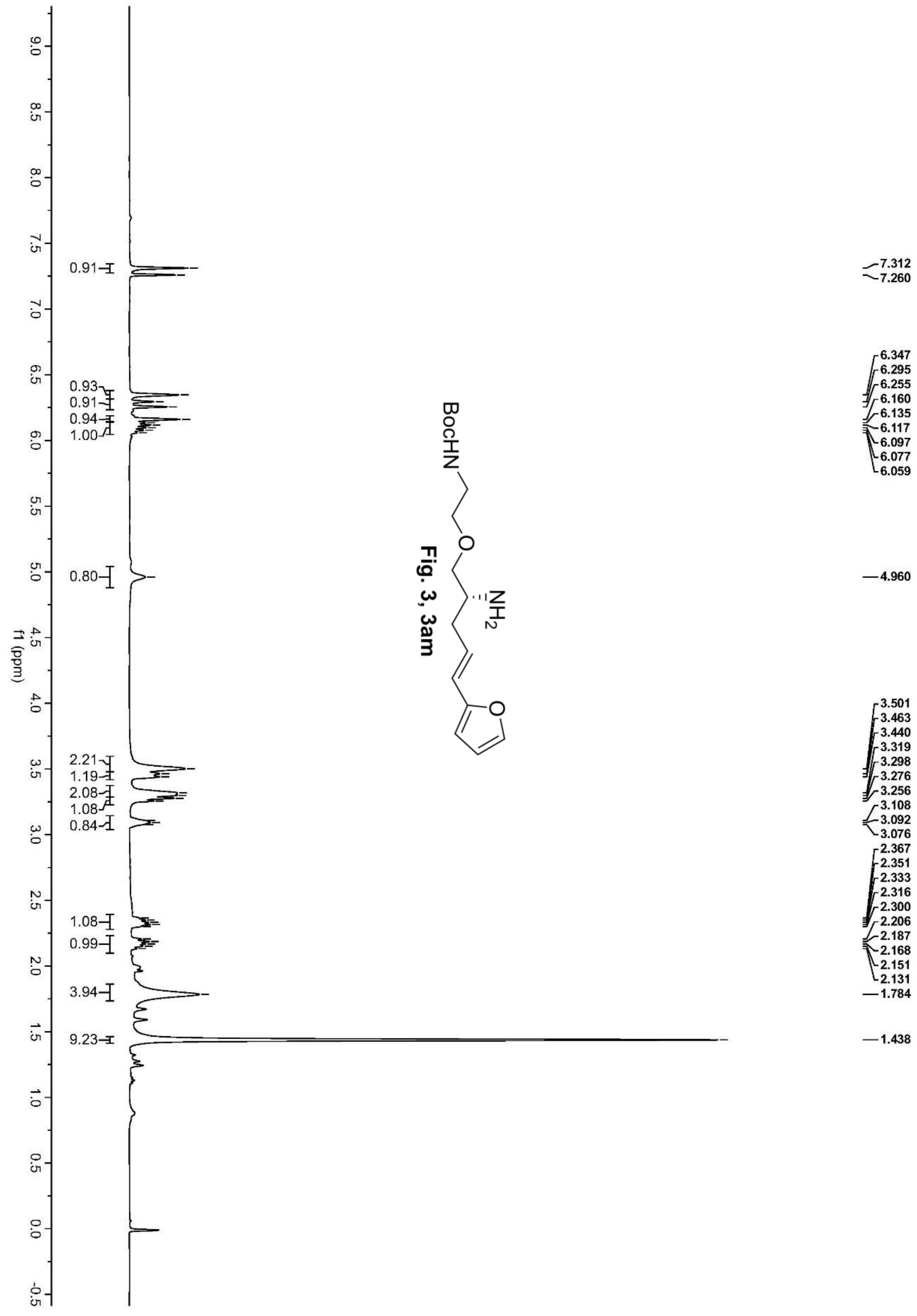
BocHN
NH2
f1 (ppm)
9.0
8.5
8.0
7.5
7.0
6.5
6.0
5.5
5.0
4.5
4.0
3.5
3.0
2.5
2.0
1.5
1.0
0.5
0.0
-0.5
0.91
0.93
0.91
0.94
1.00
0.80
2.21
1.19
2.08
1.08
0.84
1.08
0.99
3.94
9.23
7.312
7.260
6.347
6.295
6.255
6.160
6.135
6.117
6.097
6.077
6.059
4.960
3.501
3.463
3.440
3.319
3.298
3.276
3.256
3.108
3.092
3.076
2.367
2.351
2.333
2.316
2.300
2.206
2.187
2.168
2.151
2.131
1.784
1.438
Fig. 3, 3am

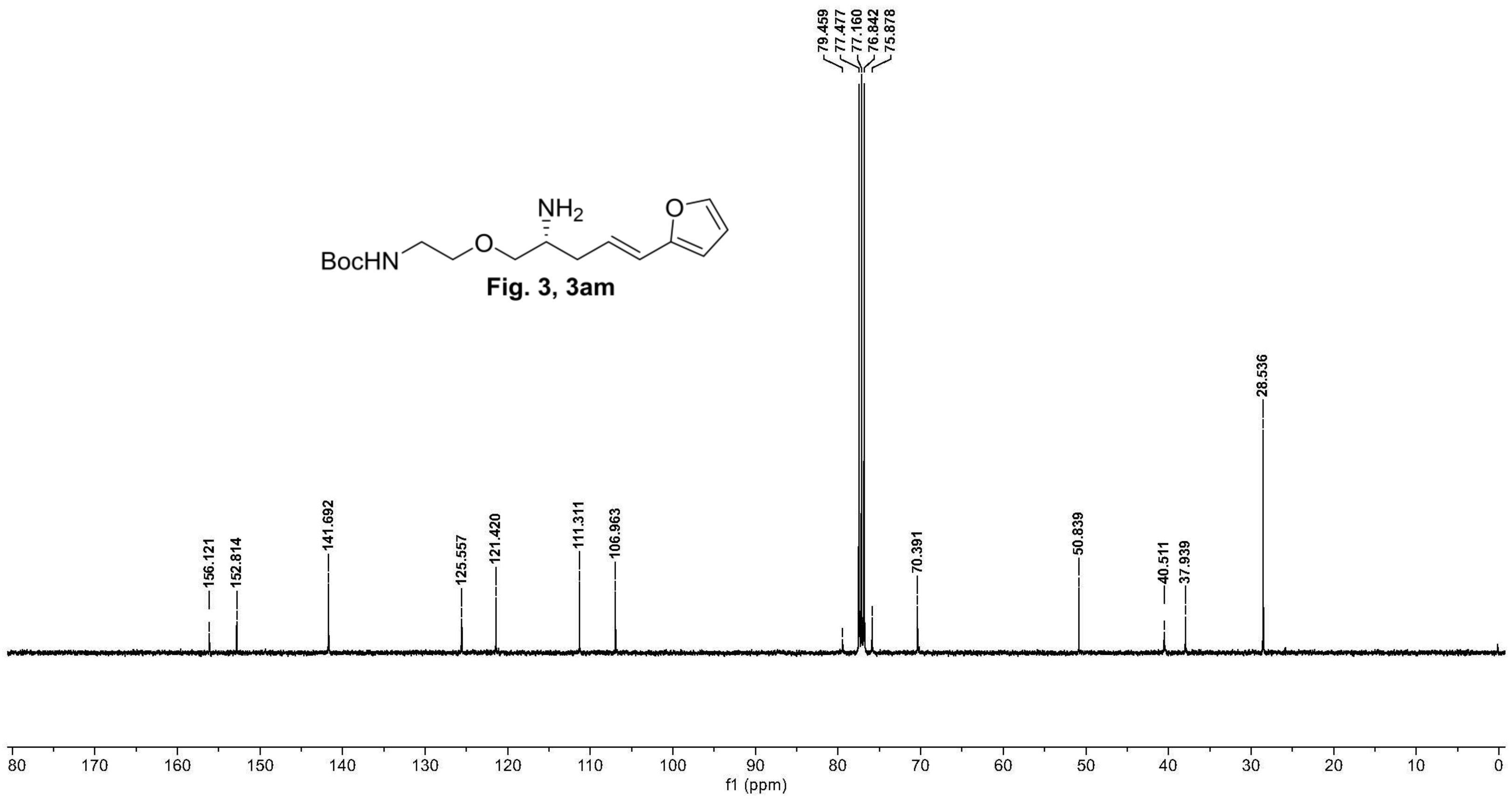

BocHN
O
NH2
O
79.459
77.477
77.160
76.842
75.878
156.121
152.814
141.692
125.557
121.420
111.311
106.963
70.391
50.839
40.511
37.939
28.536
80
170
160
150
140
130
120
110
100
90
80
70
60
50
40
30
20
10
0
f1 (ppm)

Fig. 3, 3am

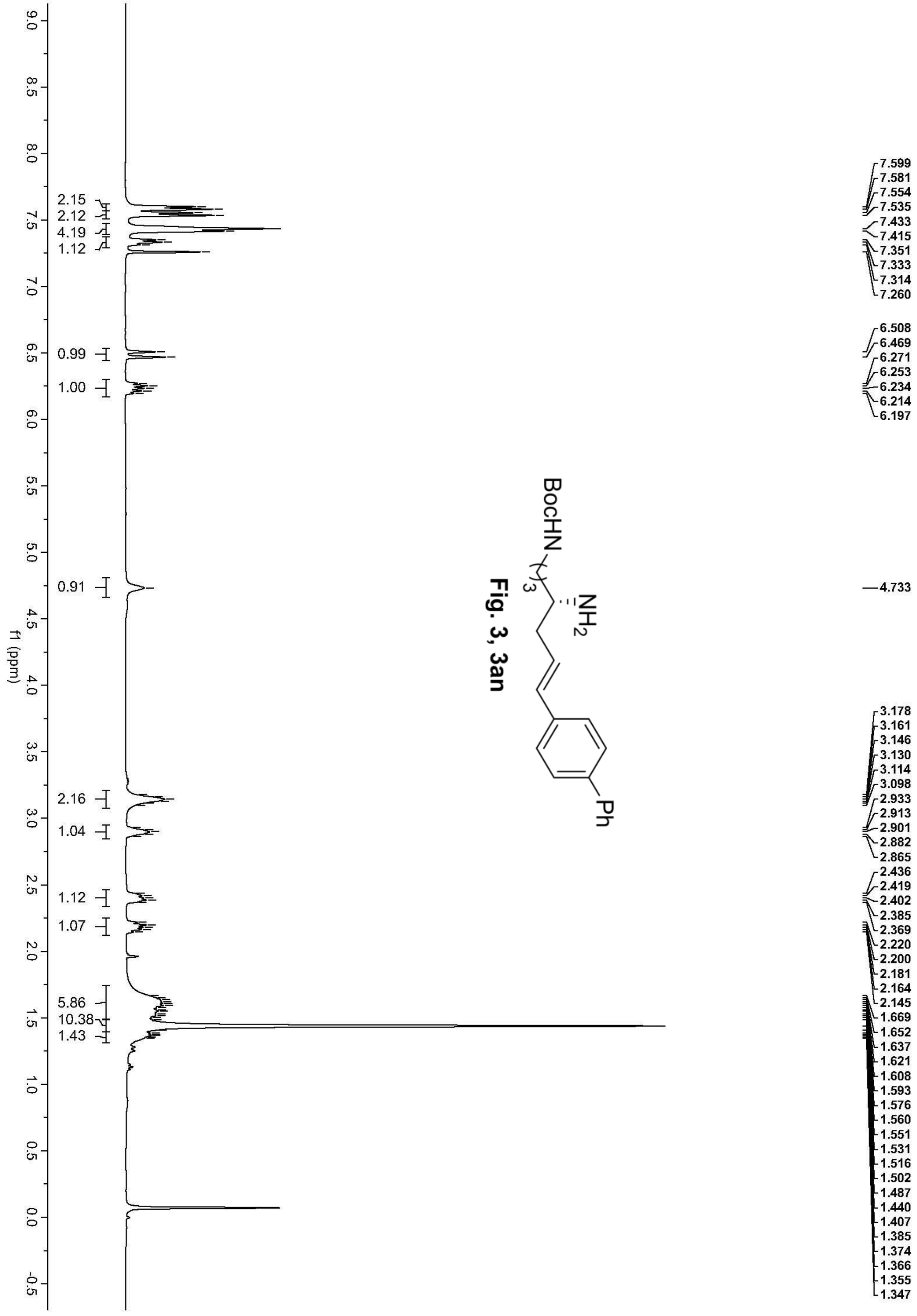

BocHN
NH2
Ph
3
f1 (ppm)
9.0
8.5
8.0
7.5
7.0
6.5
6.0
5.5
5.0
4.5
4.0
3.5
3.0
2.5
2.0
1.5
1.0
0.5
0.0
-0.5
2.15
2.12
4.19
1.12
0.99
1.00
0.91
2.16
1.04
1.12
1.07
5.86
10.38
1.43
7.599
7.581
7.554
7.535
7.433
7.415
7.351
7.333
7.314
7.260
6.508
6.469
6.271
6.253
6.234
6.214
6.197
4.733
3.178
3.161
3.146
3.130
3.114
3.098
2.933
2.913
2.901
2.882
2.865
2.436
2.419
2.402
2.385
2.369
2.220
2.200
2.181
2.164
2.145
1.669
1.652
1.637
1.621
1.608
1.593
1.576
1.560
1.551
1.531
1.516
1.502
1.487
1.440
1.407
1.385
1.374
1.366
1.355
1.347

Fig. 3, 3an

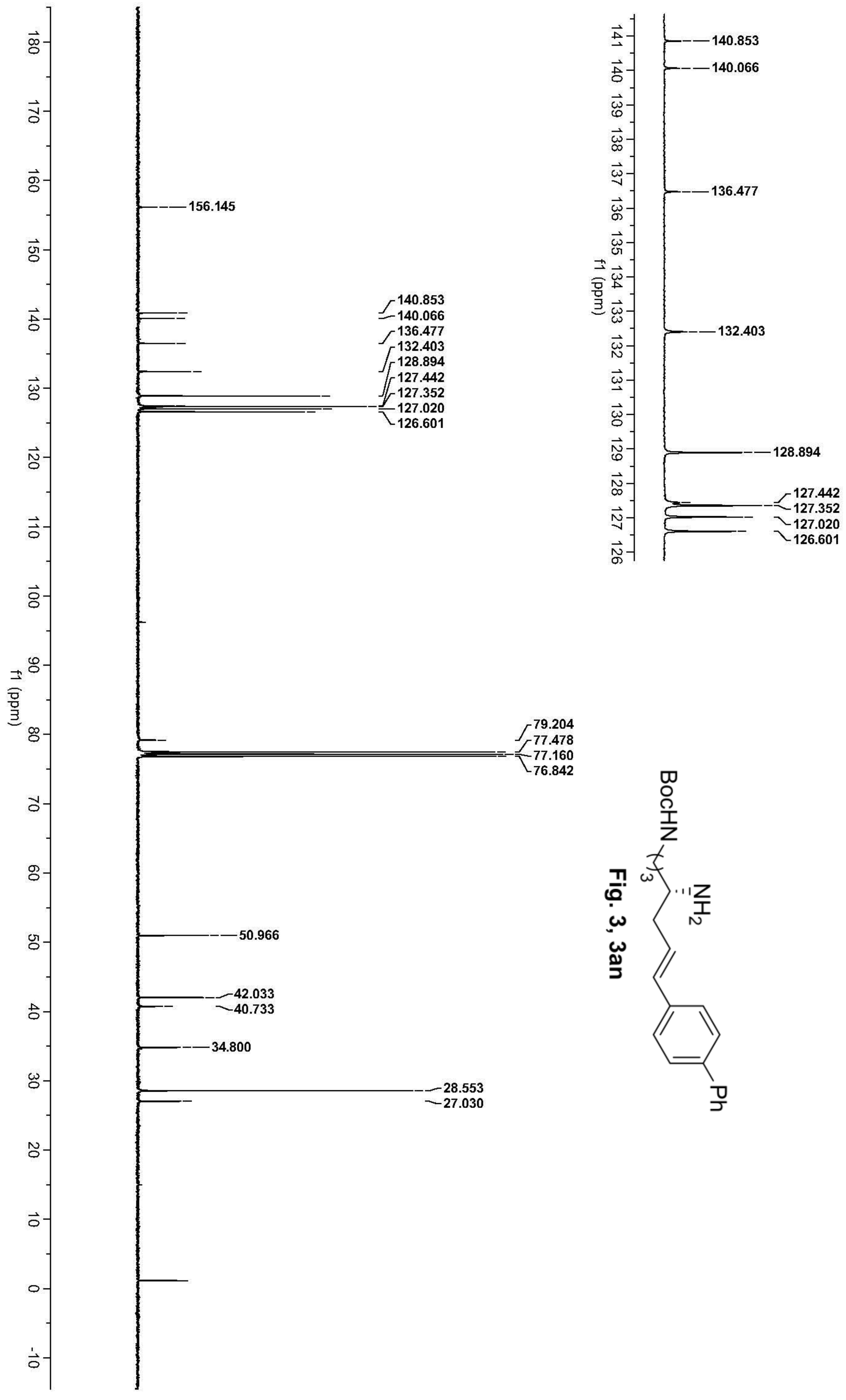

156.145
140.853
140.066
136.477
132.403
128.894
127.442
127.352
127.020
126.601
79.204
77.478
77.160
76.842
50.966
42.033
40.733
34.800
28.553
27.030
f1 (ppm)
BocHN
NH2
Ph

Fig. 3, 3an

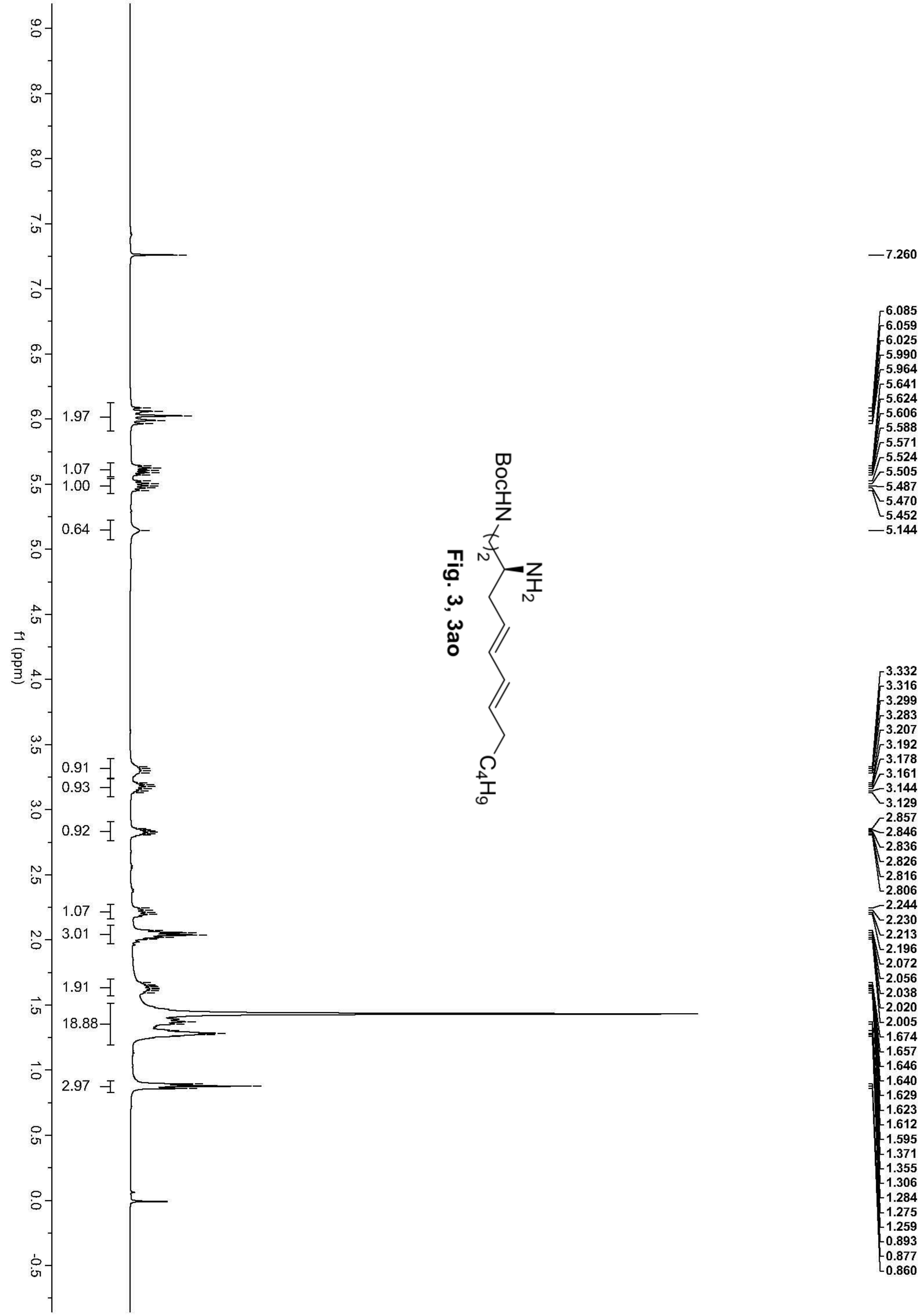
BocHN
NH2
C4H9
f1 (ppm)
1.97
1.07
1.00
0.64
0.91
0.93
0.92
1.07
3.01
1.91
18.88
2.97
7.260
6.085
6.059
6.025
5.990
5.964
5.641
5.624
5.606
5.588
5.571
5.524
5.505
5.487
5.470
5.452
5.144
3.332
3.316
3.299
3.283
3.207
3.192
3.178
3.161
3.144
3.129
2.857
2.846
2.836
2.826
2.816
2.806
2.244
2.230
2.213
2.196
2.072
2.056
2.038
2.020
2.005
1.674
1.657
1.646
1.640
1.629
1.623
1.612
1.595
1.371
1.355
1.306
1.284
1.275
1.259
0.893
0.877
0.860

Fig. 3, 3ao

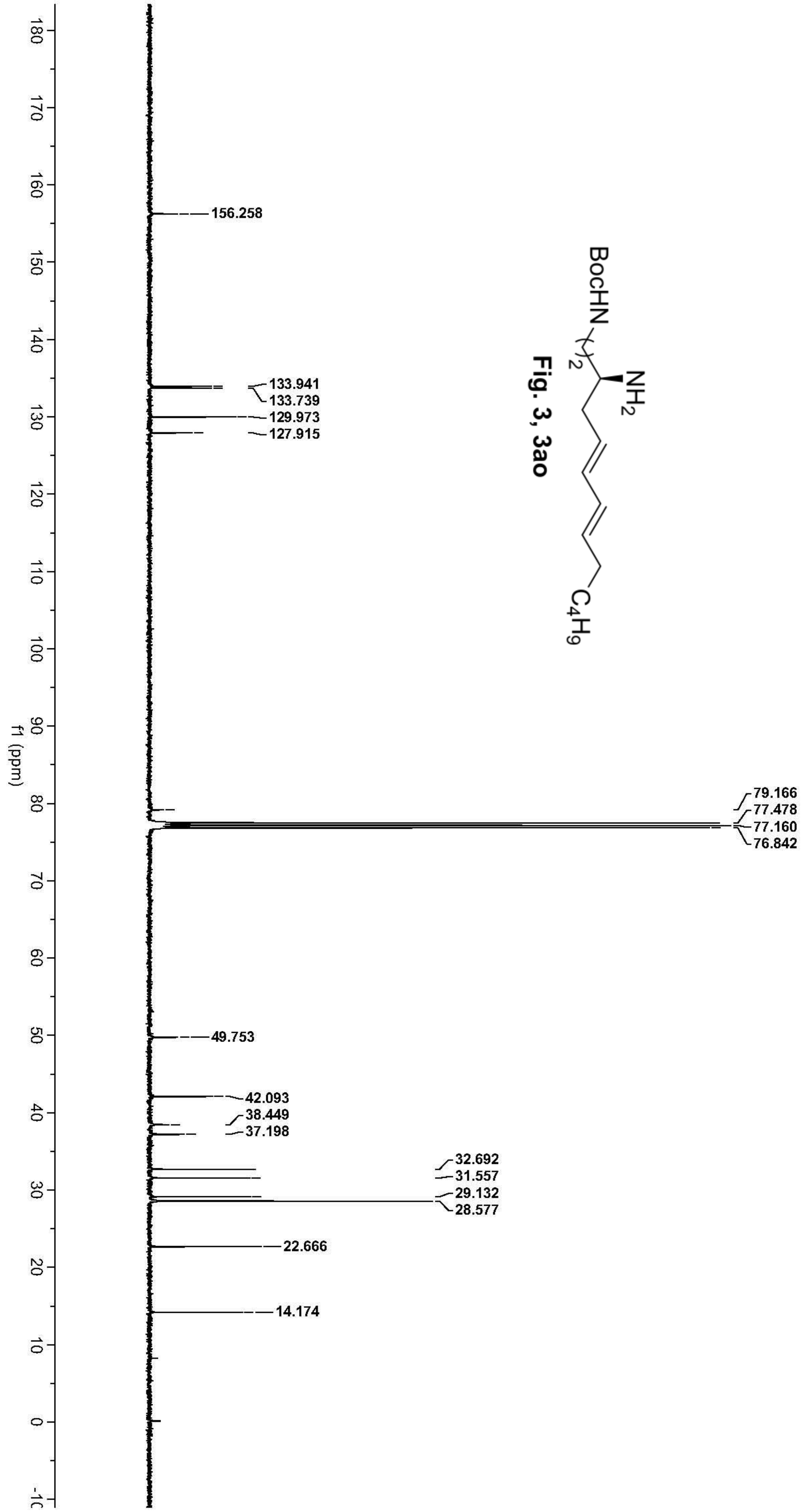
156.258
133.941
133.739
129.973
127.915
79.166
77.478
77.160
76.842
49.753
42.093
38.449
37.198
32.692
31.557
29.132
28.577
22.666
14.174
f1 (ppm)
BocHN
NH2
C4H9
Fig. 3, 3ao

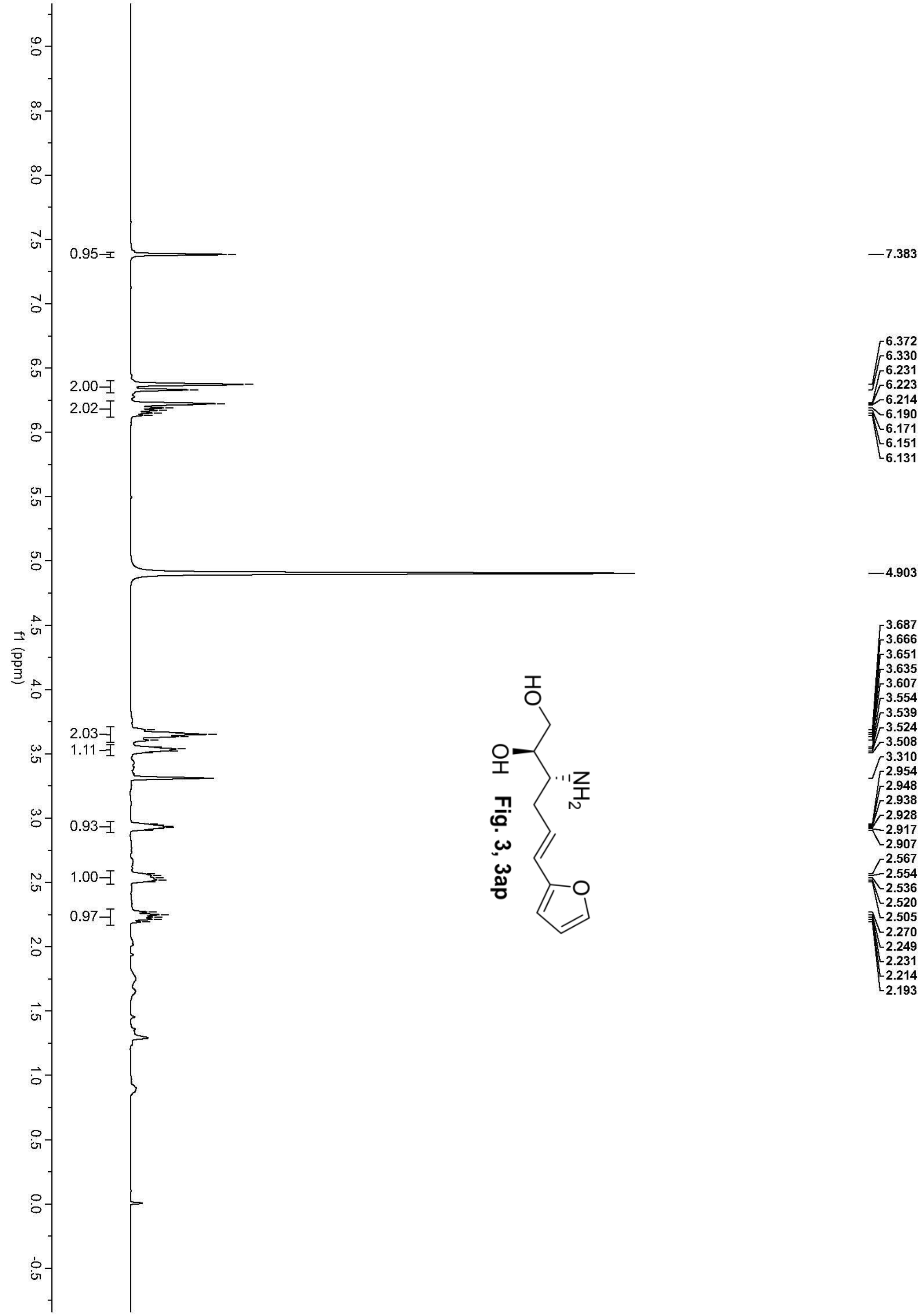

7.383
6.372
6.330
6.231
6.223
6.214
6.190
6.171
6.151
6.131
4.903
3.687
3.666
3.651
3.635
3.607
3.554
3.539
3.524
3.508
3.310
2.954
2.948
2.938
2.928
2.917
2.907
2.567
2.554
2.536
2.520
2.505
2.270
2.249
2.231
2.214
2.193
0.95
2.00
2.02
2.03
1.11
0.93
1.00
0.97
9.0
8.5
8.0
7.5
7.0
6.5
6.0
5.5
5.0
4.5
4.0
3.5
3.0
2.5
2.0
1.5
1.0
0.5
0.0
-0.5
f1 (ppm)
HO
OH
NH2
O

Fig. 3, 3ap

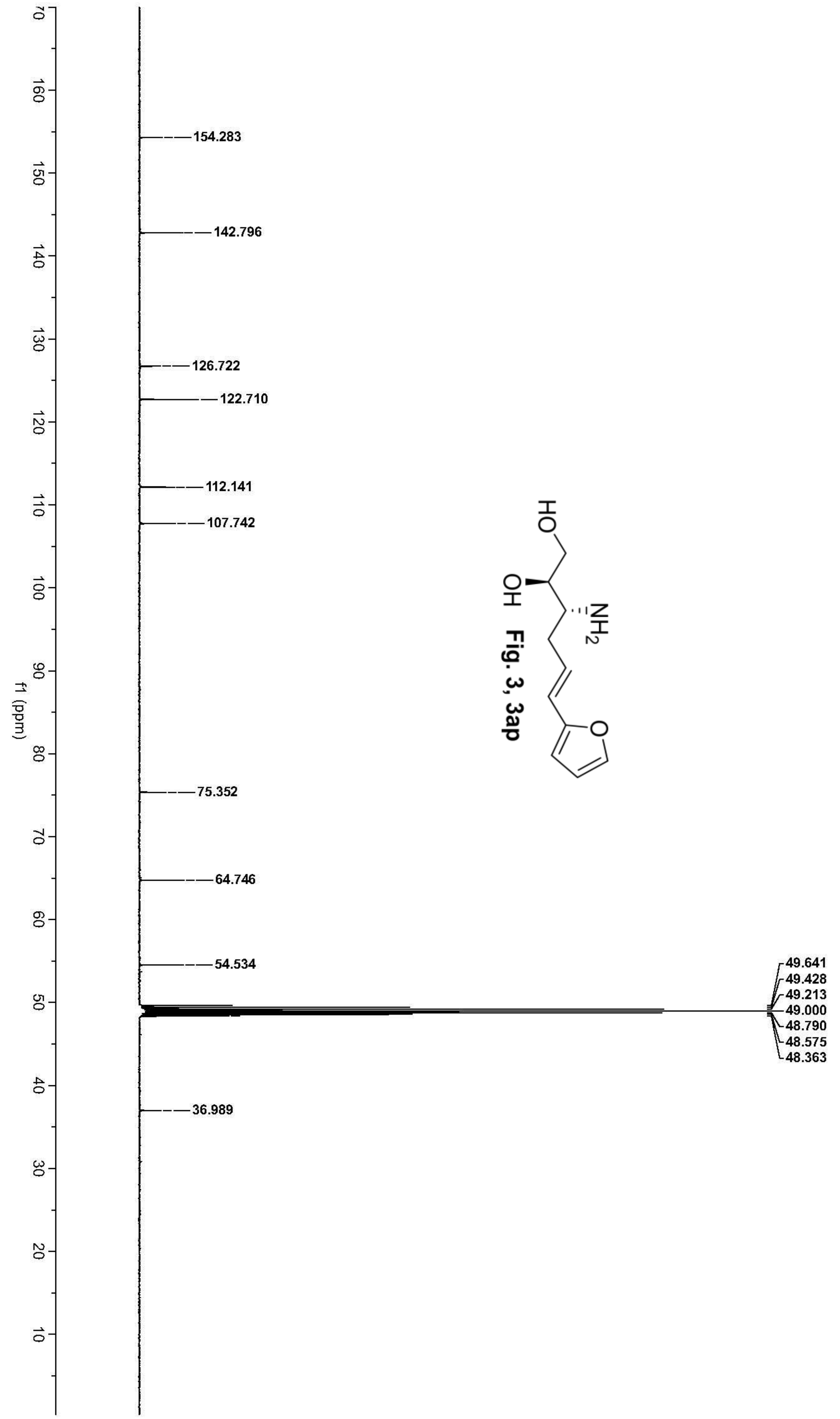
154.283
142.796
126.722
122.710
112.141
107.742
75.352
64.746
54.534
49.641
49.428
49.213
49.000
48.790
48.575
48.363
36.989
f1 (ppm)
HO
OH
NH2
O

Fig. 3, 3ap

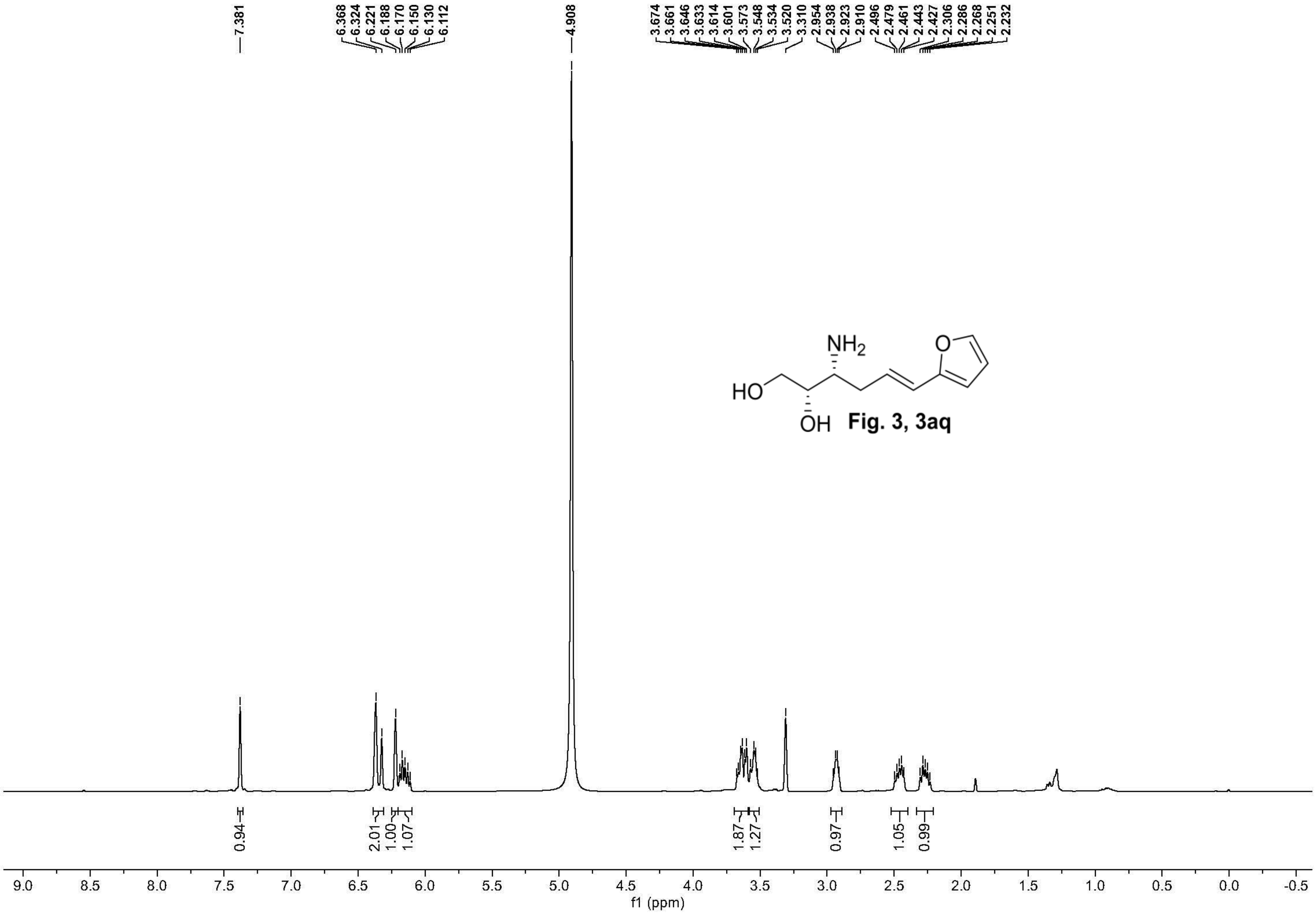
7.381
6.368
6.324
6.221
6.188
6.170
6.150
6.130
6.112
4.908
3.674
3.661
3.646
3.633
3.614
3.601
3.573
3.548
3.534
3.520
3.310
2.954
2.938
2.923
2.910
2.496
2.479
2.461
2.443
2.427
2.306
2.286
2.268
2.251
2.232
NH2
HO
OH
0.94
2.01
1.00
1.07
1.87
1.27
0.97
1.05
0.99
9.0
8.5
8.0
7.5
7.0
6.5
6.0
5.5
5.0
4.5
4.0
3.5
3.0
2.5
2.0
1.5
1.0
0.5
0.0
-0.5
f1 (ppm)

Fig. 3, 3aq

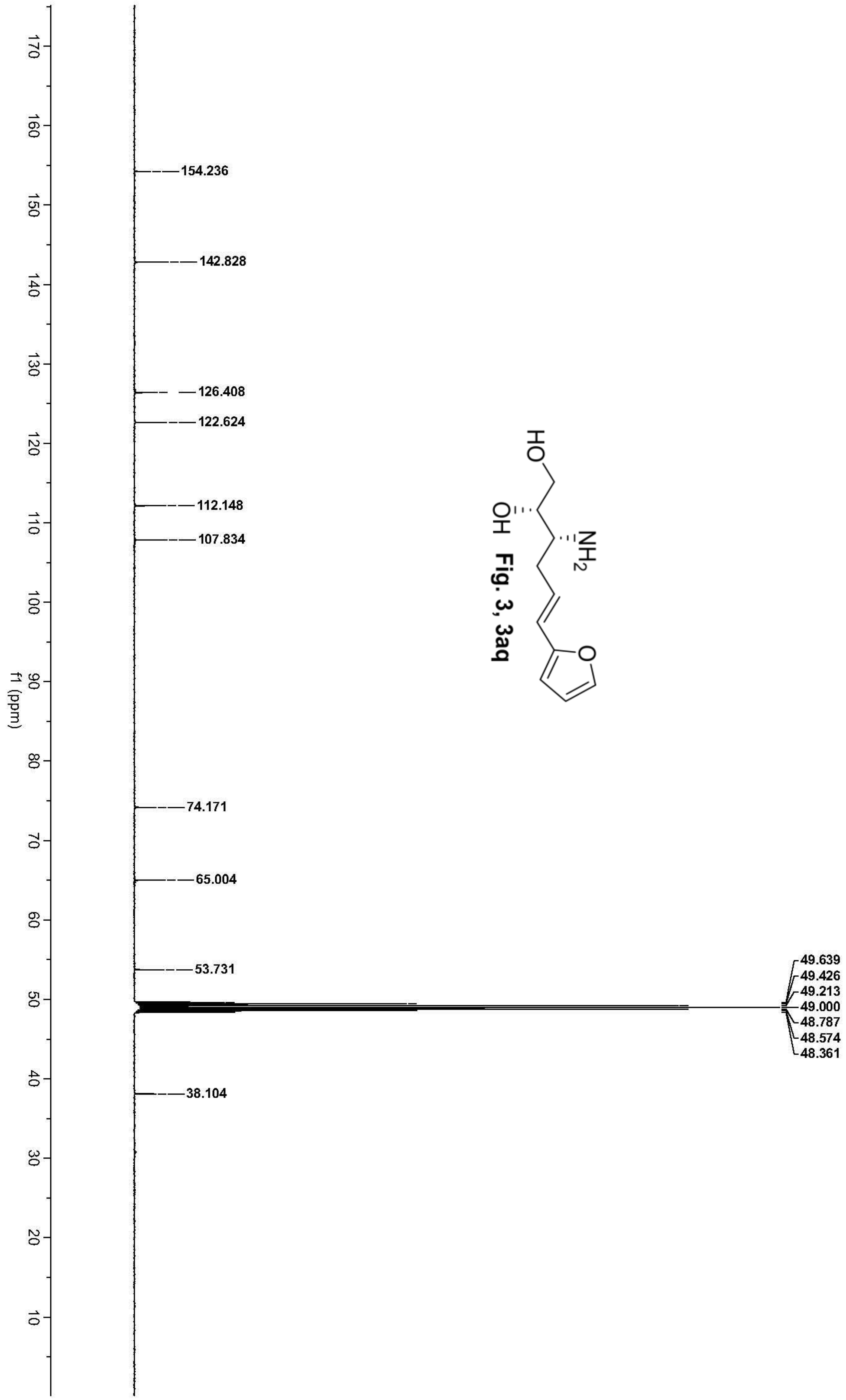

154.236
142.828
126.408
122.624
112.148
107.834
74.171
65.004
53.731
49.639
49.426
49.213
49.000
48.787
48.574
48.361
38.104
f1 (ppm)
HO
OH
NH2
O
Fig. 3, 3aq

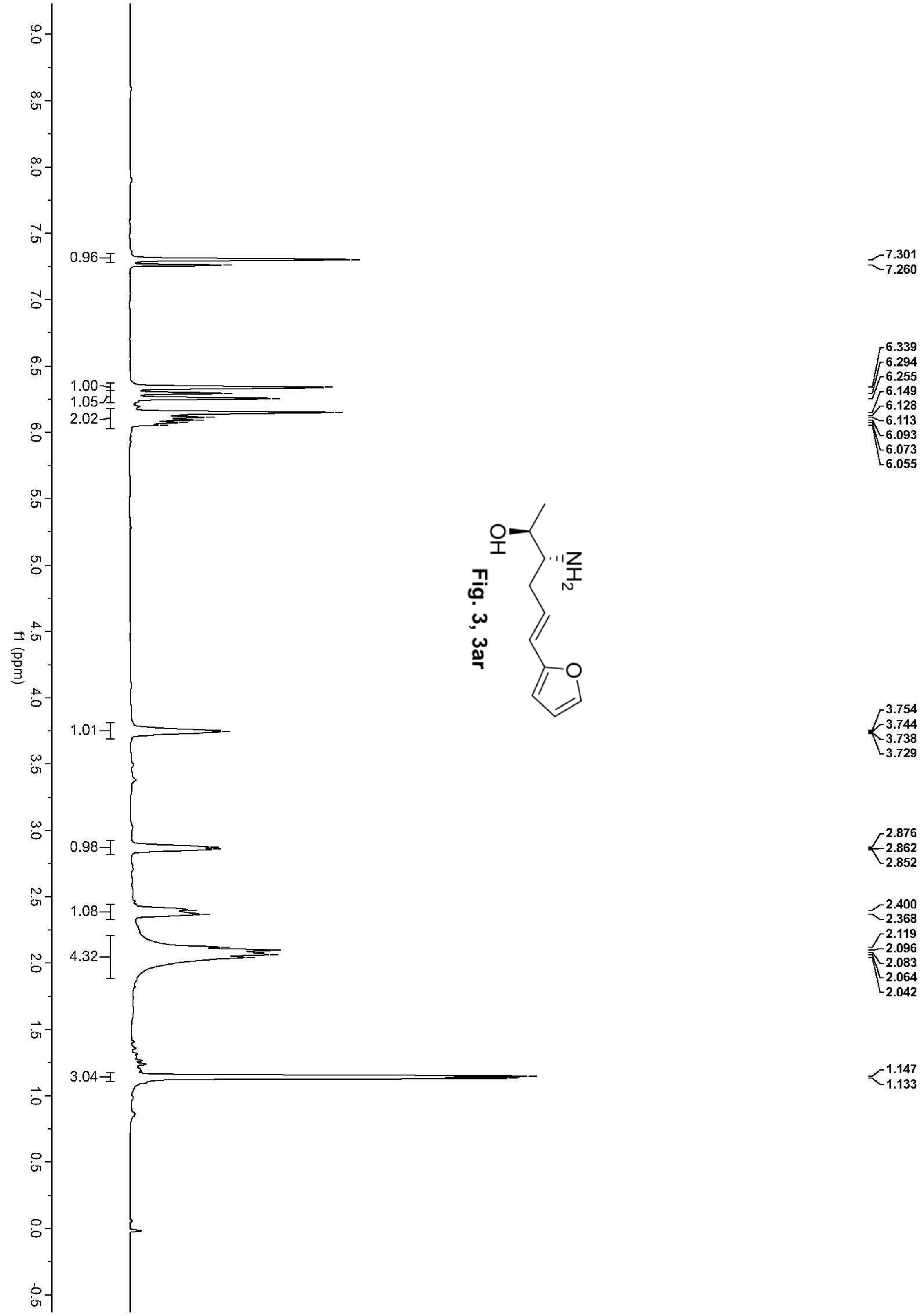
7.301
7.260
6.339
6.294
6.255
6.149
6.128
6.113
6.093
6.073
6.055
3.754
3.744
3.738
3.729
2.876
2.862
2.852
2.400
2.368
2.119
2.096
2.083
2.064
2.042
1.147
1.133
0.96
1.00
1.05
2.02
1.01
0.98
1.08
4.32
3.04
f1 (ppm)
NH2
OH
Fig. 3, 3ar

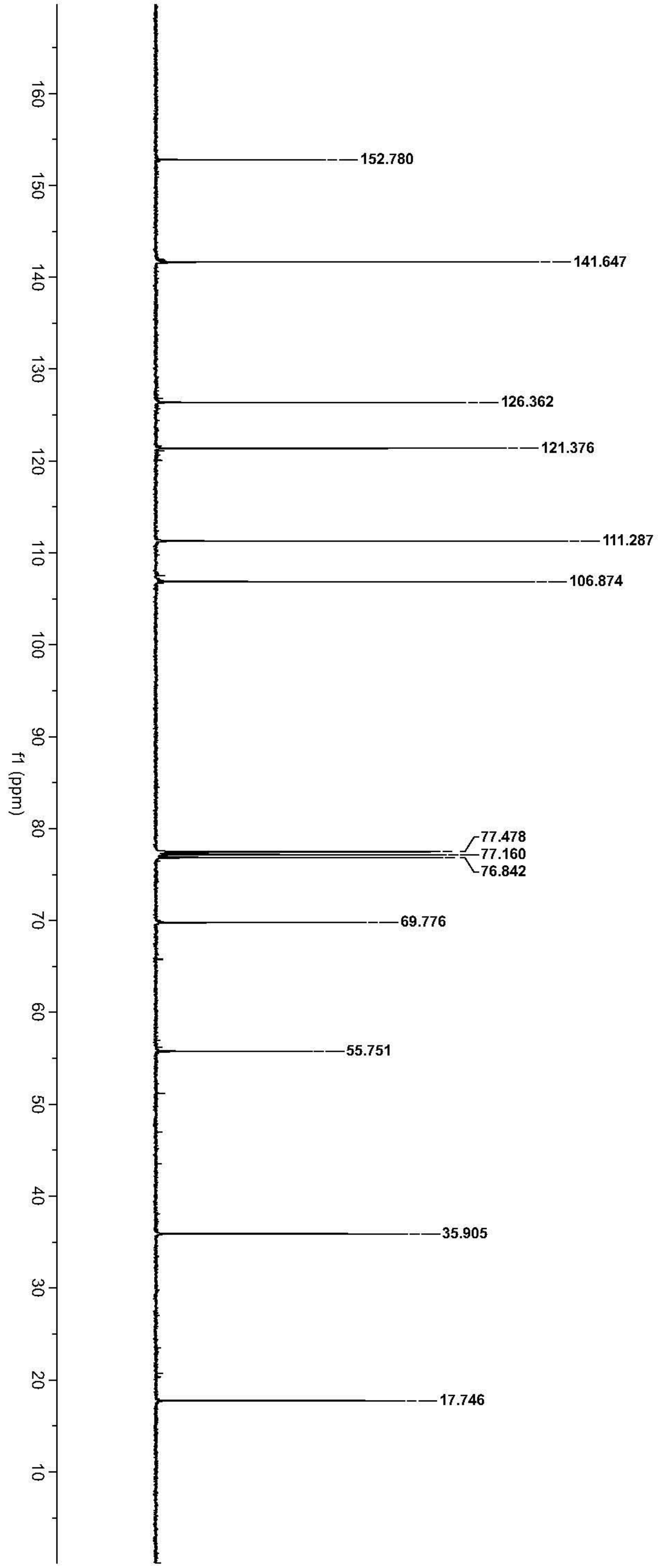

160
150
140
130
120
110
100
90
80
70
60
50
40
30
20
10
f1 (ppm)
152.780
141.647
126.362
121.376
111.287
106.874
77.478
77.160
76.842
69.776
55.751
35.905
17.746

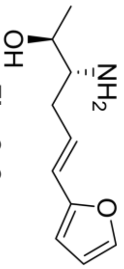

NH2
OH
O

Fig. 3, 3ar

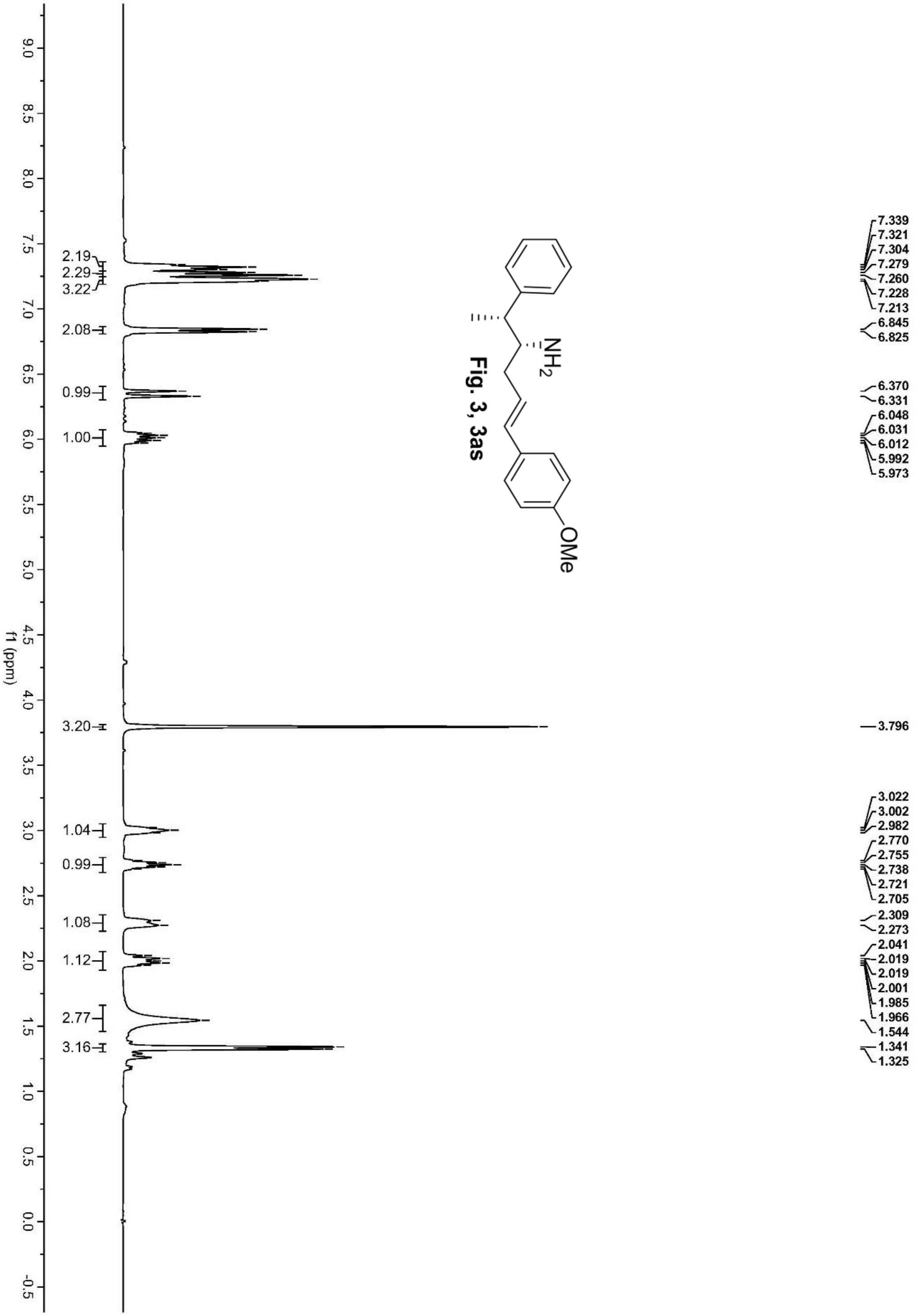

NH2
OMe
f1 (ppm)
9.0
8.5
8.0
7.5
7.0
6.5
6.0
5.5
5.0
4.5
4.0
3.5
3.0
2.5
2.0
1.5
1.0
0.5
0.0
-0.5
2.19
2.29
3.22
2.08
0.99
1.00
3.20
1.04
0.99
1.08
1.12
2.77
3.16
7.339
7.321
7.304
7.279
7.260
7.228
7.213
6.845
6.825
6.370
6.331
6.048
6.031
6.012
5.992
5.973
3.796
3.022
3.002
2.982
2.770
2.755
2.738
2.721
2.705
2.309
2.273
2.041
2.019
2.019
2.001
1.985
1.966
1.544
1.341
1.325

Fig. 3, 3as

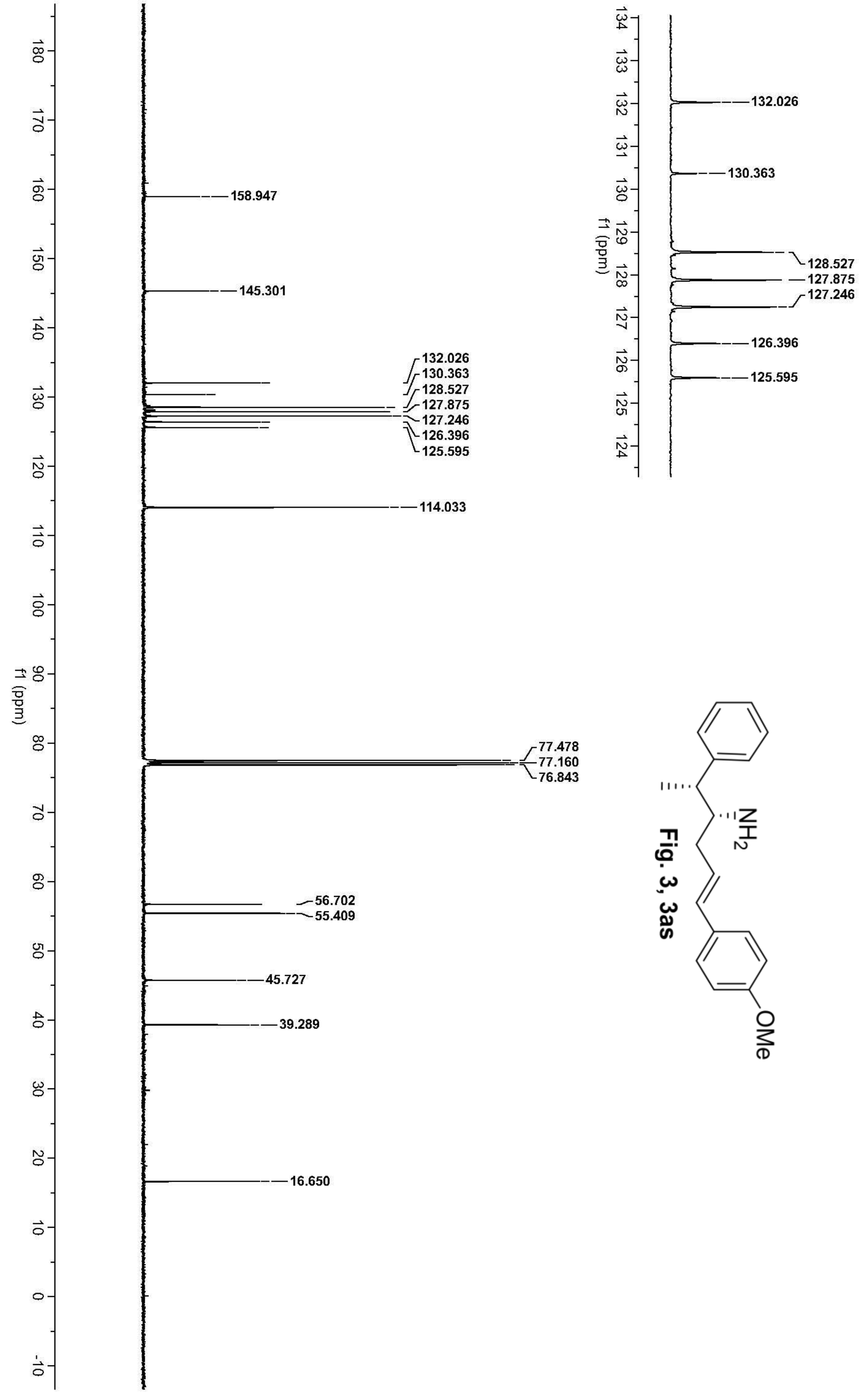
158.947
145.301
132.026
130.363
128.527
127.875
127.246
126.396
125.595
114.033
77.478
77.160
76.843
56.702
55.409
45.727
39.289
16.650
f1 (ppm)
NH_2
OMe

Fig. 3, 3as

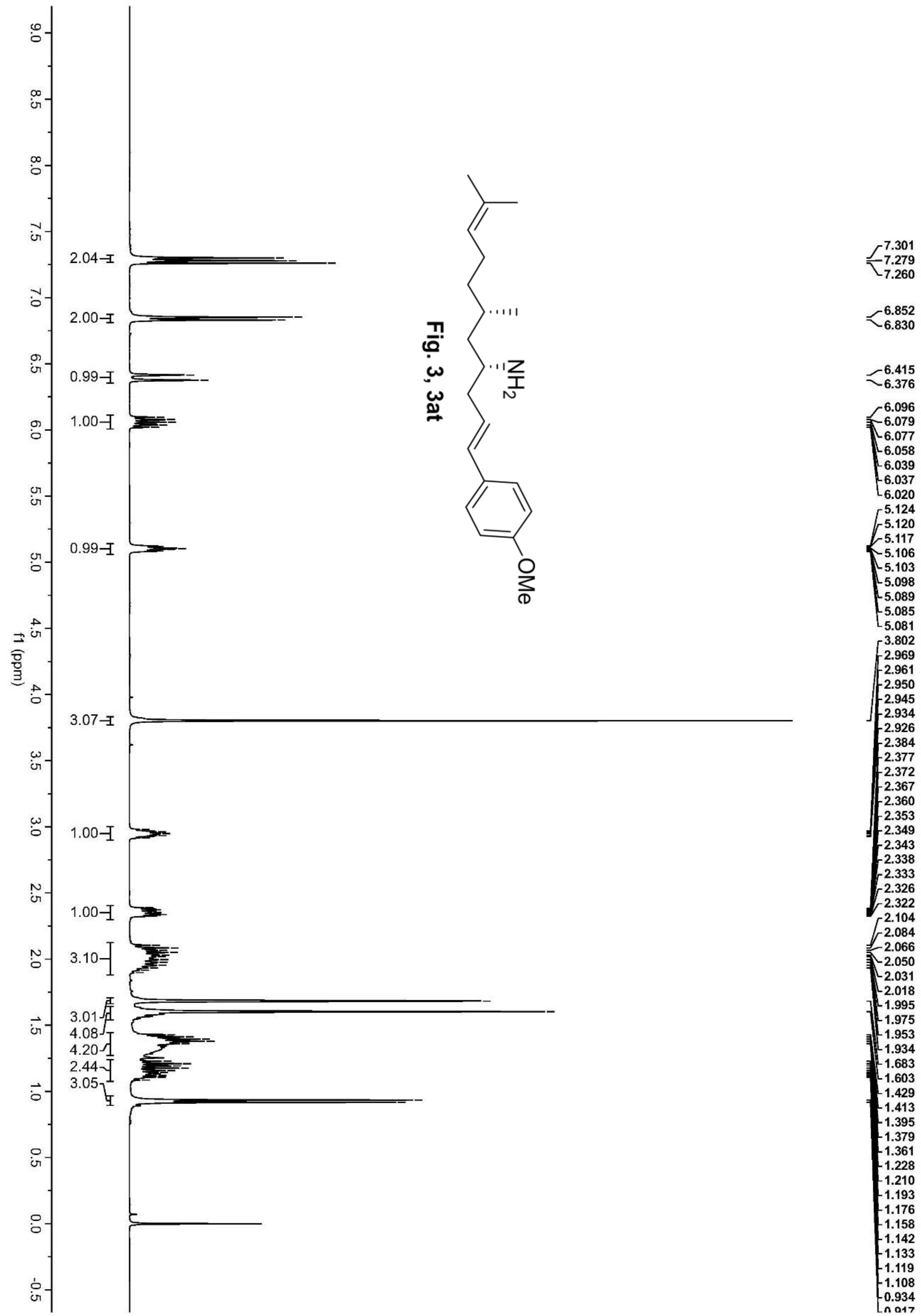
NH2
OMe
f1 (ppm)
2.04
2.00
0.99
1.00
0.99
3.07
1.00
1.00
3.10
3.01
4.08
4.20
2.44
3.05
7.301
7.279
7.260
6.852
6.830
6.415
6.376
6.096
6.079
6.077
6.058
6.039
6.037
6.020
5.124
5.120
5.117
5.106
5.103
5.098
5.089
5.085
5.081
3.802
2.969
2.961
2.950
2.945
2.934
2.926
2.384
2.377
2.372
2.367
2.360
2.353
2.349
2.343
2.338
2.333
2.326
2.322
2.104
2.084
2.066
2.050
2.031
2.018
1.995
1.975
1.953
1.934
1.683
1.603
1.429
1.413
1.395
1.379
1.361
1.228
1.210
1.193
1.176
1.158
1.142
1.133
1.119
1.108
0.934

Fig. 3, 3at

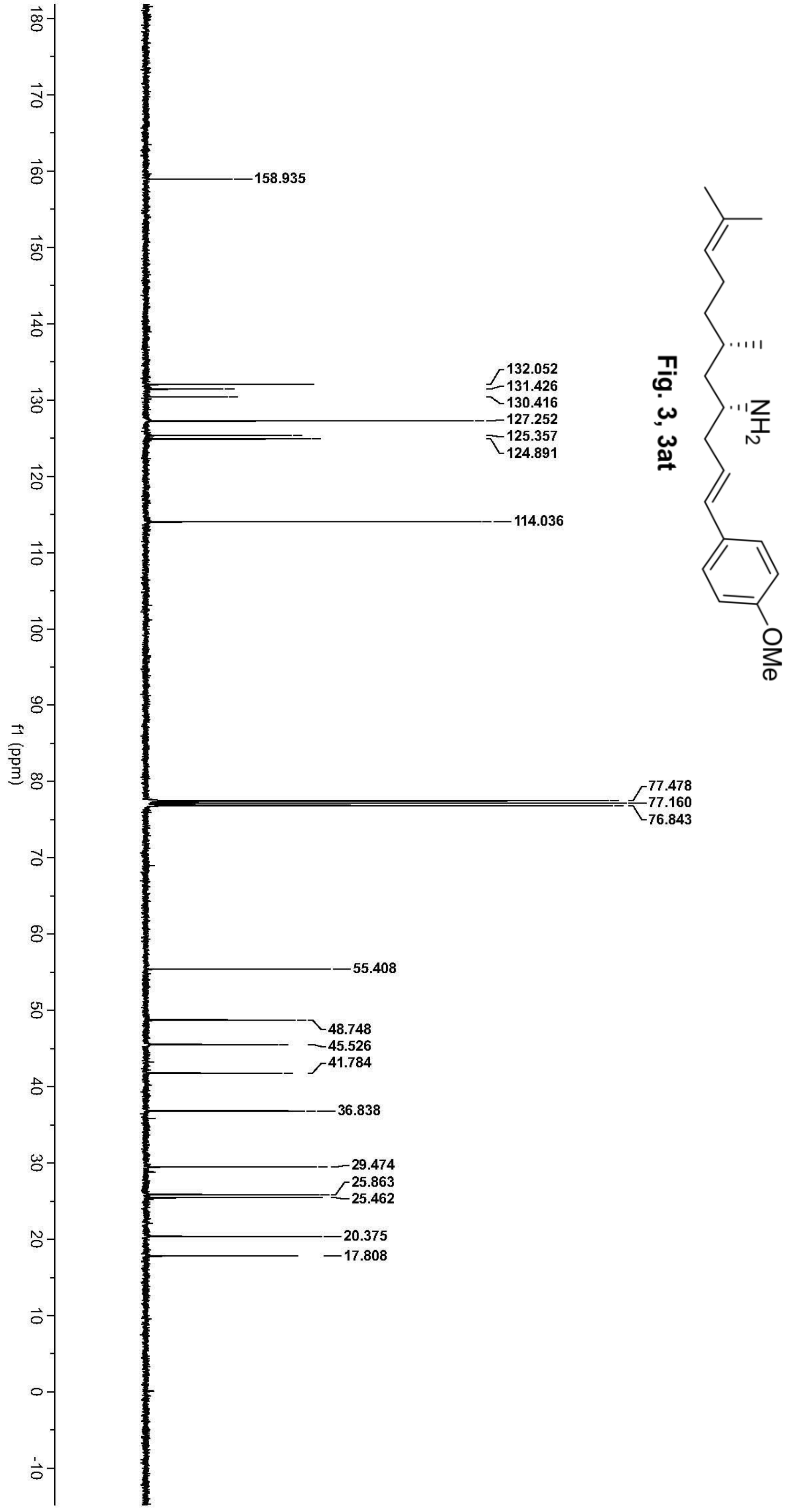
Fig. 3, 3at
NH2
OMe
158.935
132.052
131.426
130.416
127.252
125.357
124.891
114.036
77.478
77.160
76.843
55.408
48.748
45.526
41.784
36.838
29.474
25.863
25.462
20.375
17.808
f1 (ppm)

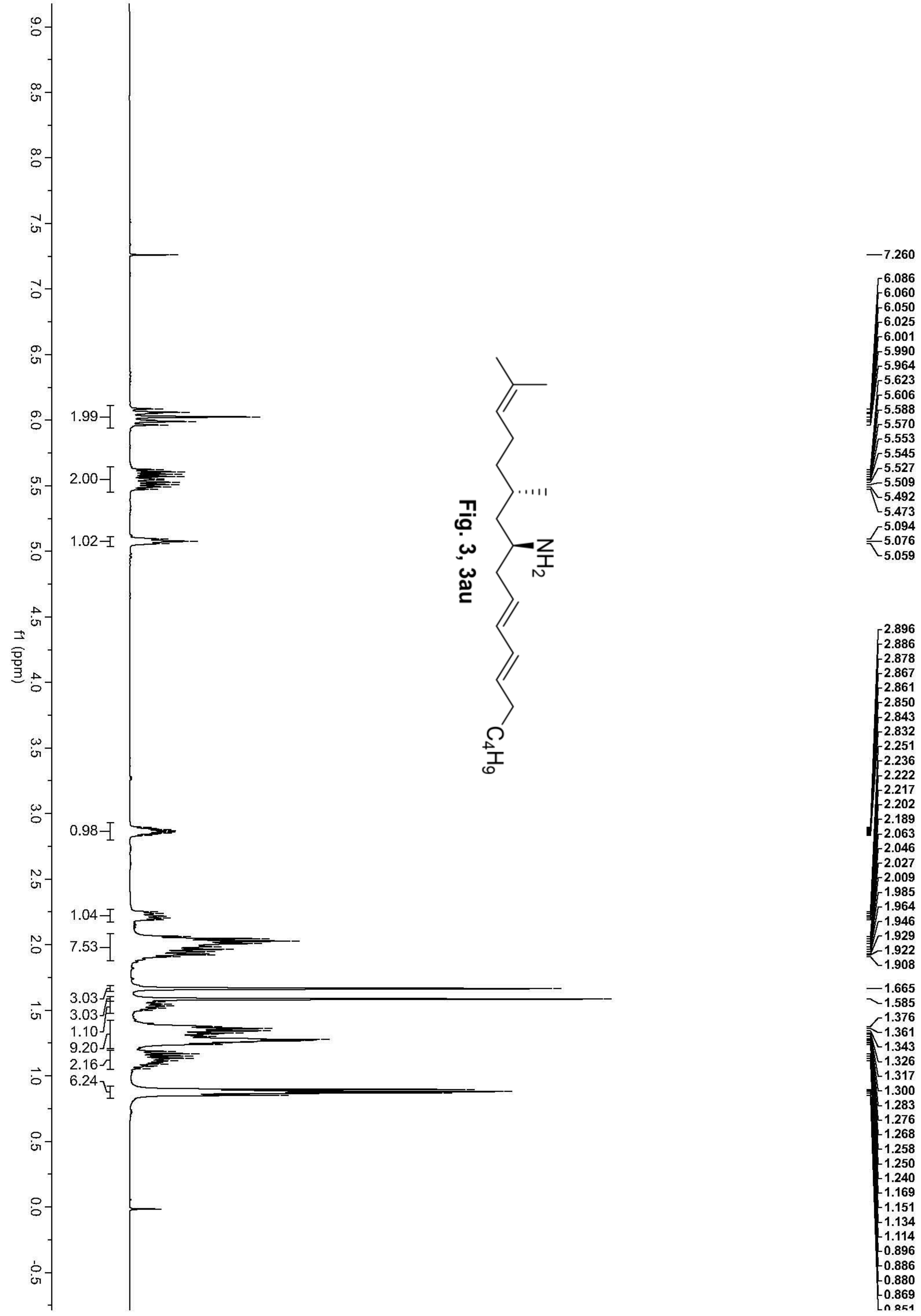
NH2
C4H9
f1 (ppm)
1.99
2.00
1.02
0.98
1.04
7.53
3.03
3.03
1.10
9.20
2.16
6.24
7.260
6.086
6.060
6.050
6.025
6.001
5.990
5.964
5.623
5.606
5.588
5.570
5.553
5.545
5.527
5.509
5.492
5.473
5.094
5.076
5.059
2.896
2.886
2.878
2.867
2.861
2.850
2.843
2.832
2.251
2.236
2.222
2.217
2.202
2.189
2.063
2.046
2.027
2.009
1.985
1.964
1.946
1.929
1.922
1.908
1.665
1.585
1.376
1.361
1.343
1.326
1.317
1.300
1.283
1.276
1.268
1.258
1.250
1.240
1.169
1.151
1.134
1.114
0.896
0.886
0.880
0.869

Fig. 3, 3au

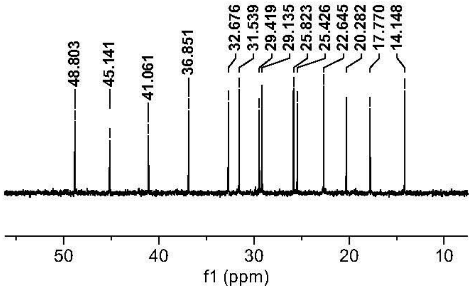
48.803
45.141
41.061
36.851
32.676
31.539
29.419
29.135
25.823
25.426
22.645
20.282
17.770
14.148
50 40 30 20 10
f1 (ppm)

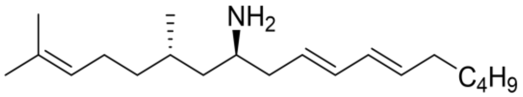
NH2
C4H9

Fig. 3, 3au

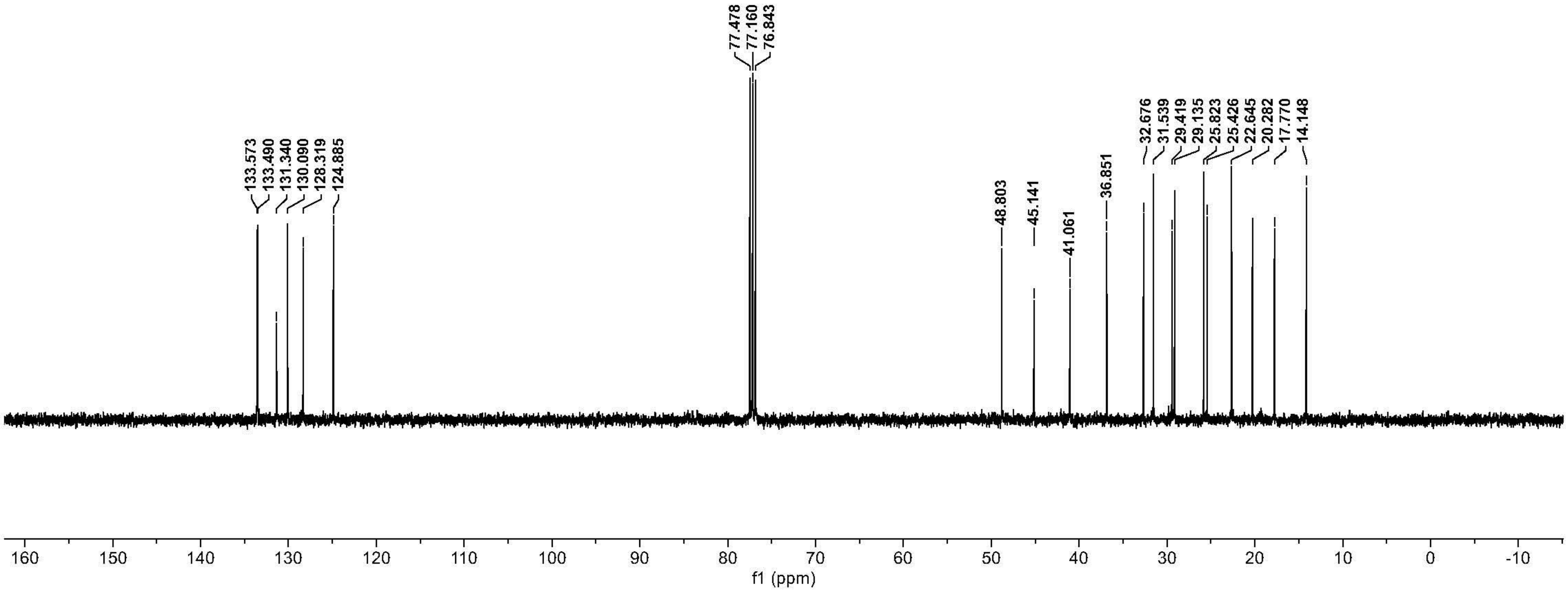
133.573
133.490
131.340
130.090
128.319
124.885
77.478
77.160
76.843
48.803
45.141
41.061
36.851
32.676
31.539
29.419
29.135
25.823
25.426
22.645
20.282
17.770
14.148
160 150 140 130 120 110 100 90 80 70 60 50 40 30 20 10 0 -10
f1 (ppm)

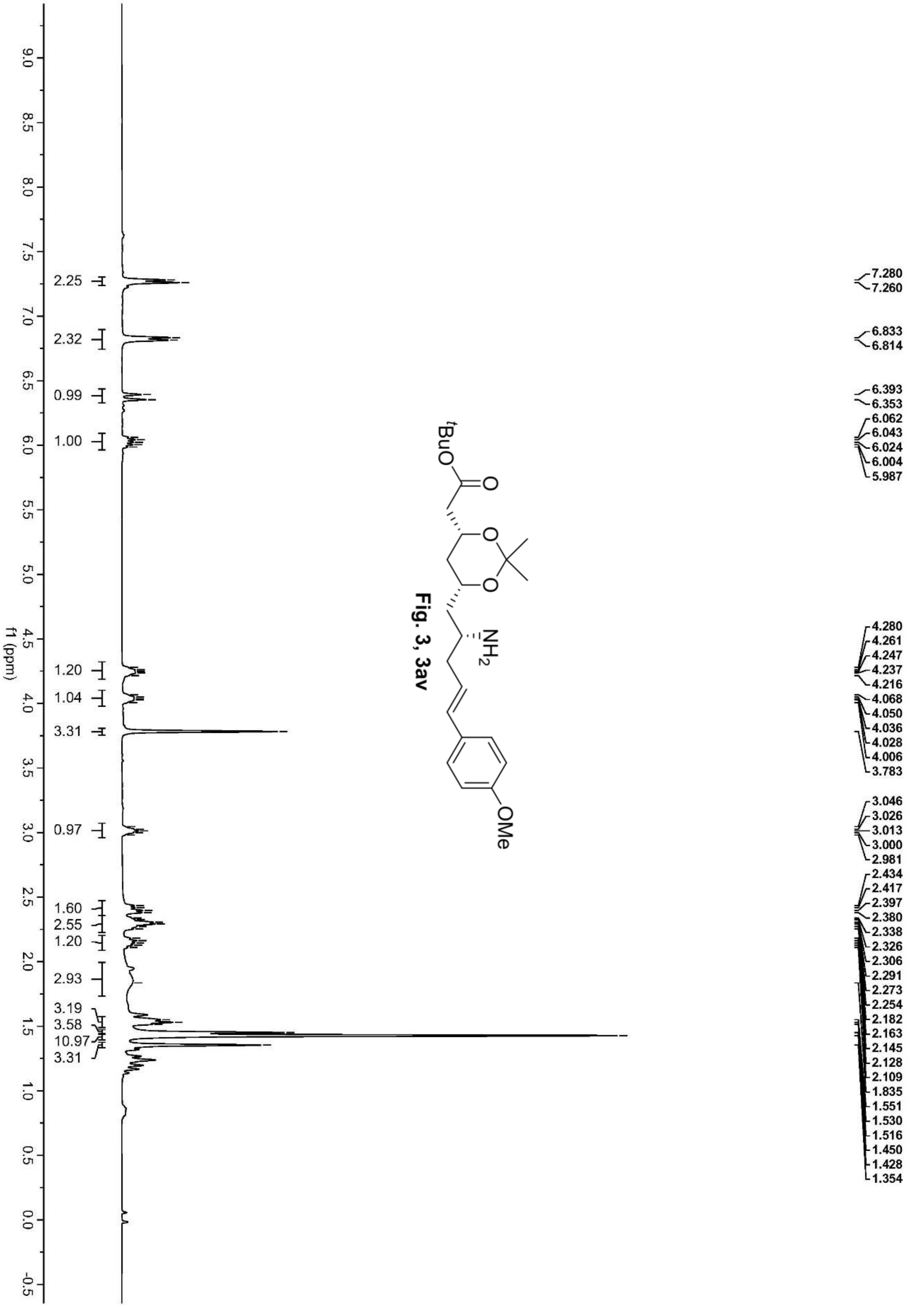

Fig. 3, 3av

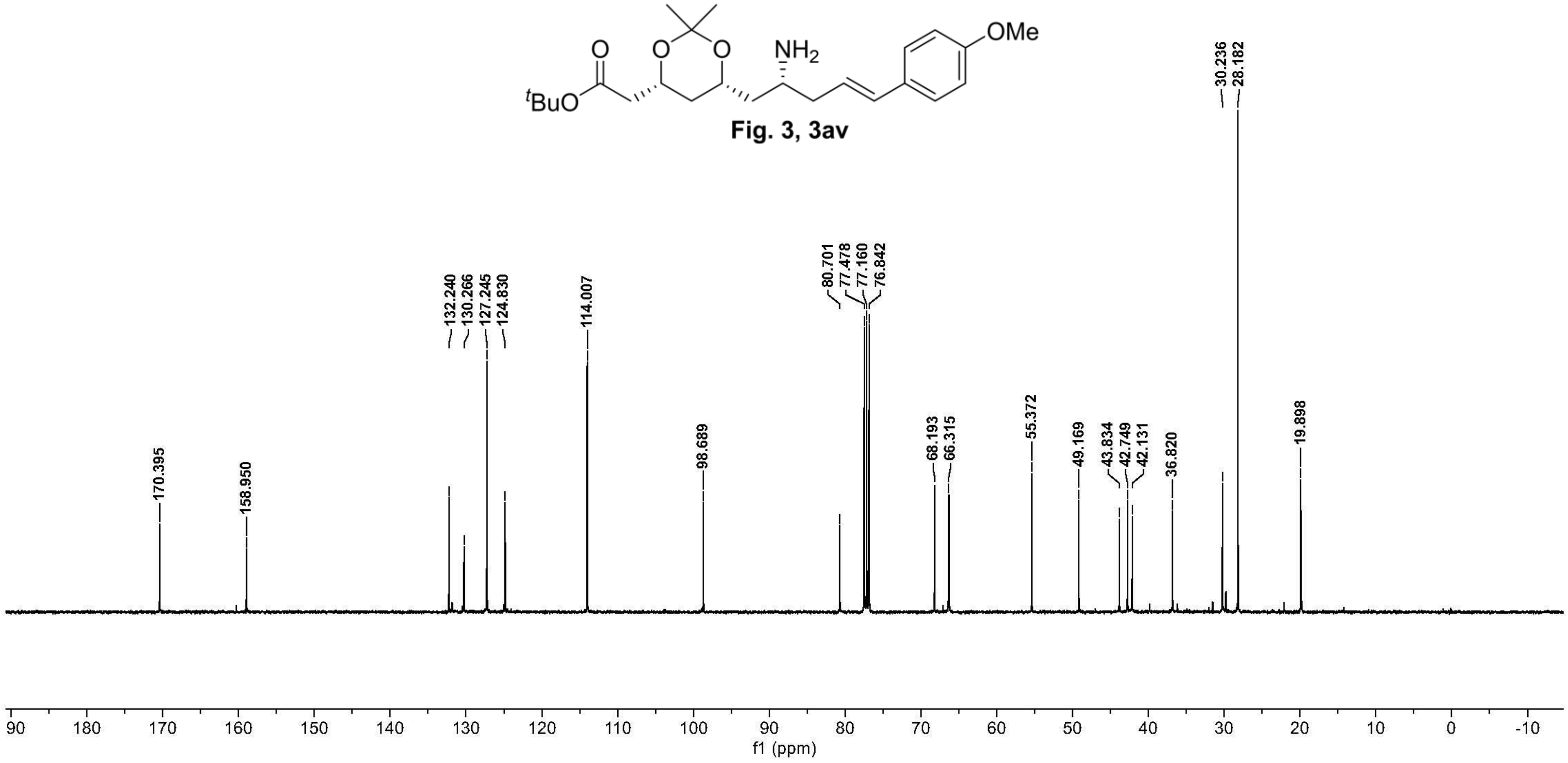
^{t}BuO
O
O
O
NH2
OMe
170.395
158.950
132.240
130.266
127.245
124.830
114.007
98.689
80.701
77.478
77.160
76.842
68.193
66.315
55.372
49.169
43.834
42.749
42.131
36.820
30.236
28.182
19.898
f1 (ppm)

Fig. 3, 3av

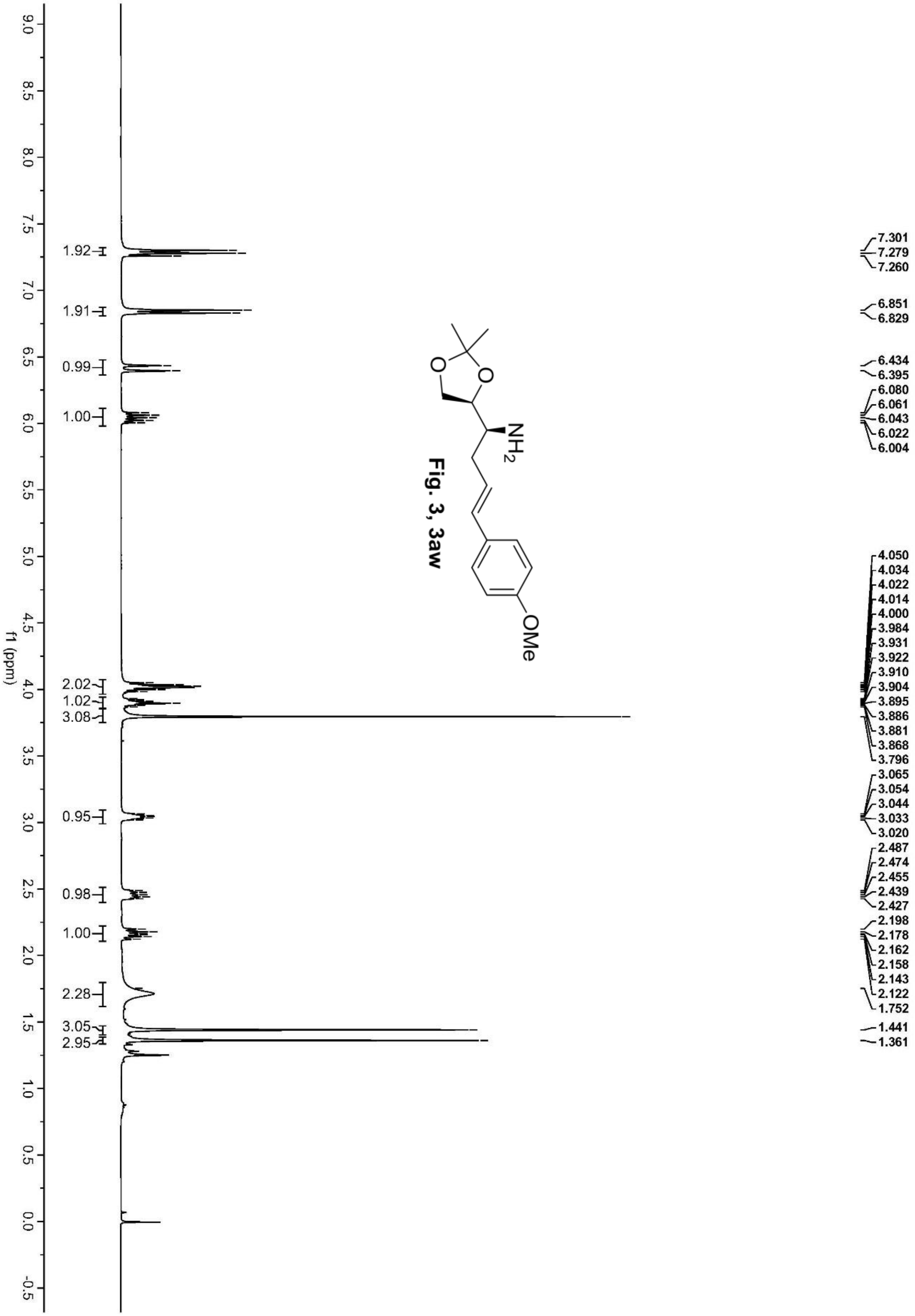

Fig. 3, 3aw
NH2
OMe
f1 (ppm)
9.0 8.5 8.0 7.5 7.0 6.5 6.0 5.5 5.0 4.5 4.0 3.5 3.0 2.5 2.0 1.5 1.0 0.5 0.0 -0.5
1.92 1.91 0.99 1.00 2.02 1.02 3.08 0.95 0.98 1.00 2.28 3.05 2.95
7.301 7.279 7.260
6.851 6.829
6.434 6.395
6.080 6.061 6.043 6.022 6.004
4.050 4.034 4.022 4.014 4.000 3.984 3.931 3.922 3.910 3.904 3.895 3.886 3.881 3.868 3.796
3.065 3.054 3.044 3.033 3.020
2.487 2.474 2.455 2.439 2.427
2.198 2.178 2.162 2.158 2.143 2.122 1.752
1.441
1.361

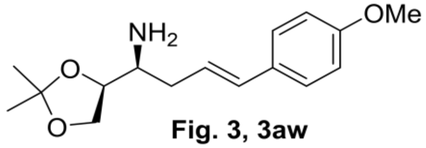
NH2
OMe
O
O

Fig. 3, 3aw

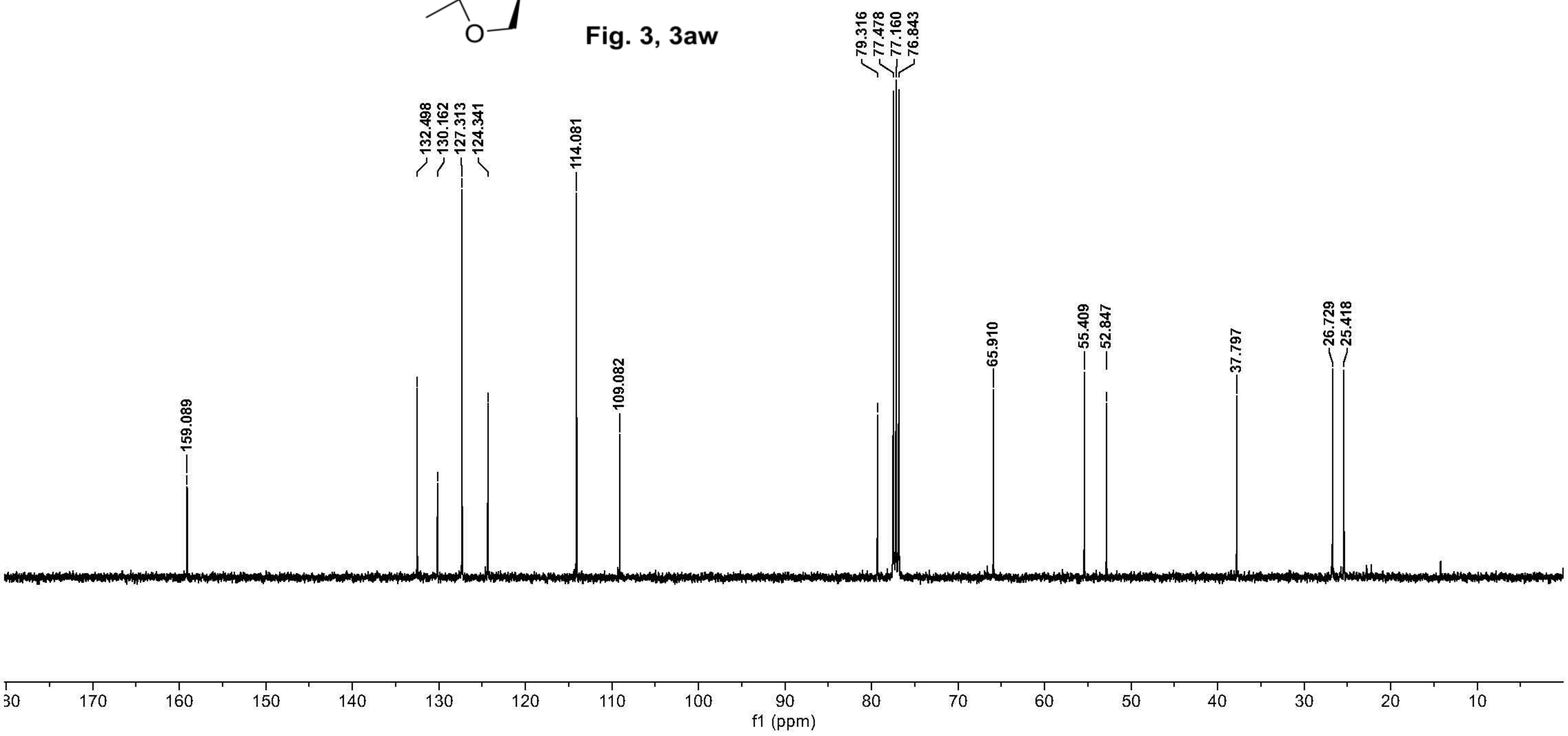
159.089
132.498
130.162
127.313
124.341
114.081
109.082
79.316
77.478
77.160
76.843
65.910
55.409
52.847
37.797
26.729
25.418
170
160
150
140
130
120
110
100
90
80
70
60
50
40
30
20
10
f1 (ppm)

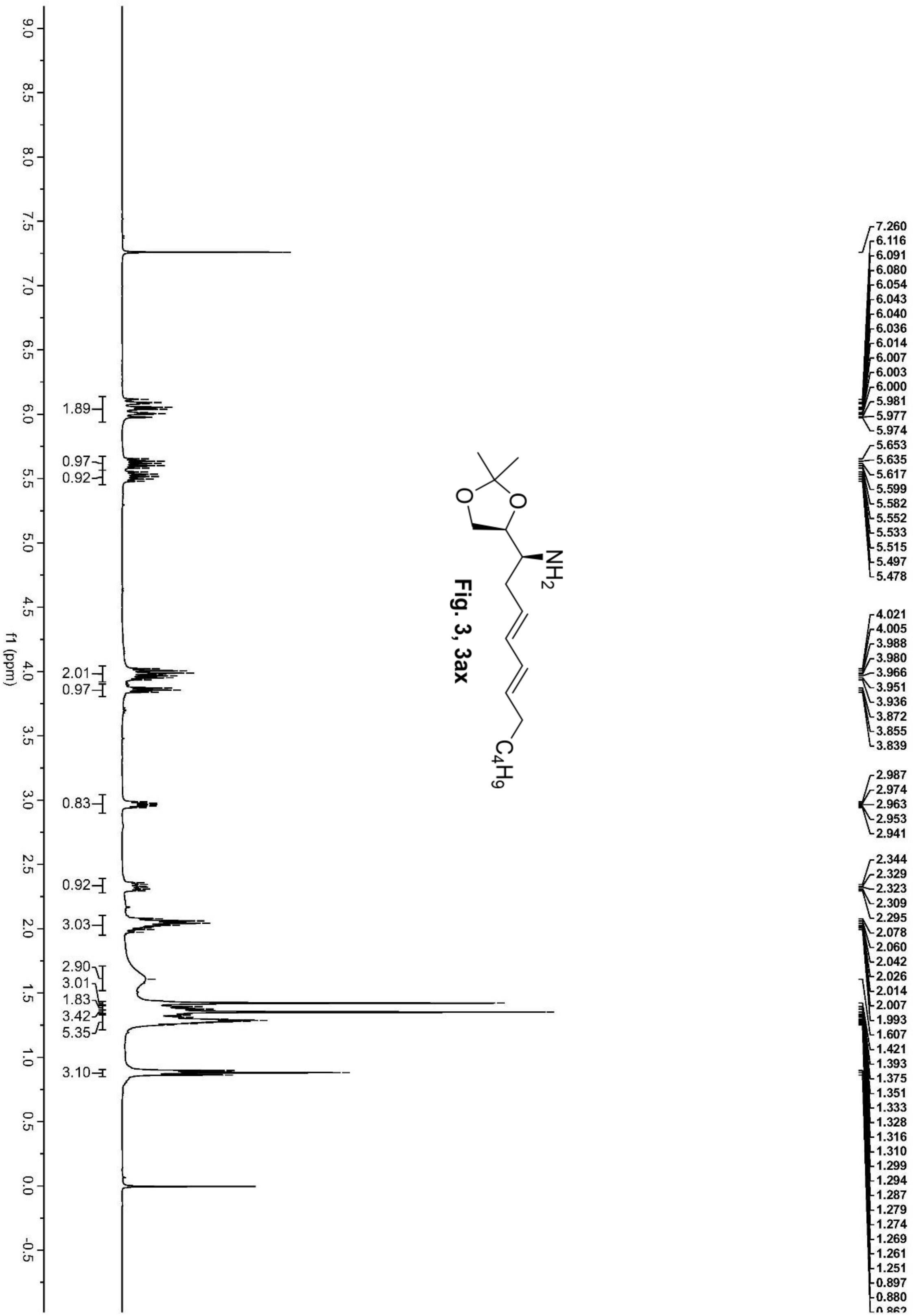

9.0
8.5
8.0
7.5
7.0
6.5
6.0
5.5
5.0
4.5
4.0
3.5
3.0
2.5
2.0
1.5
1.0
0.5
0.0
-0.5
f1 (ppm)
1.89
0.97
0.92
2.01
0.97
0.83
0.92
3.03
2.90
3.01
1.83
3.42
5.35
3.10
O
O
NH2
C4H9
7.260
6.116
6.091
6.080
6.054
6.043
6.040
6.036
6.014
6.007
6.003
6.000
5.981
5.977
5.974
5.653
5.635
5.617
5.599
5.582
5.552
5.533
5.515
5.497
5.478
4.021
4.005
3.988
3.980
3.966
3.951
3.936
3.872
3.855
3.839
2.987
2.974
2.963
2.953
2.941
2.344
2.329
2.323
2.309
2.295
2.078
2.060
2.042
2.026
2.014
2.007
1.993
1.607
1.421
1.393
1.375
1.351
1.333
1.328
1.316
1.310
1.299
1.294
1.287
1.279
1.274
1.269
1.261
1.251
0.897
0.880

Fig. 3, 3ax

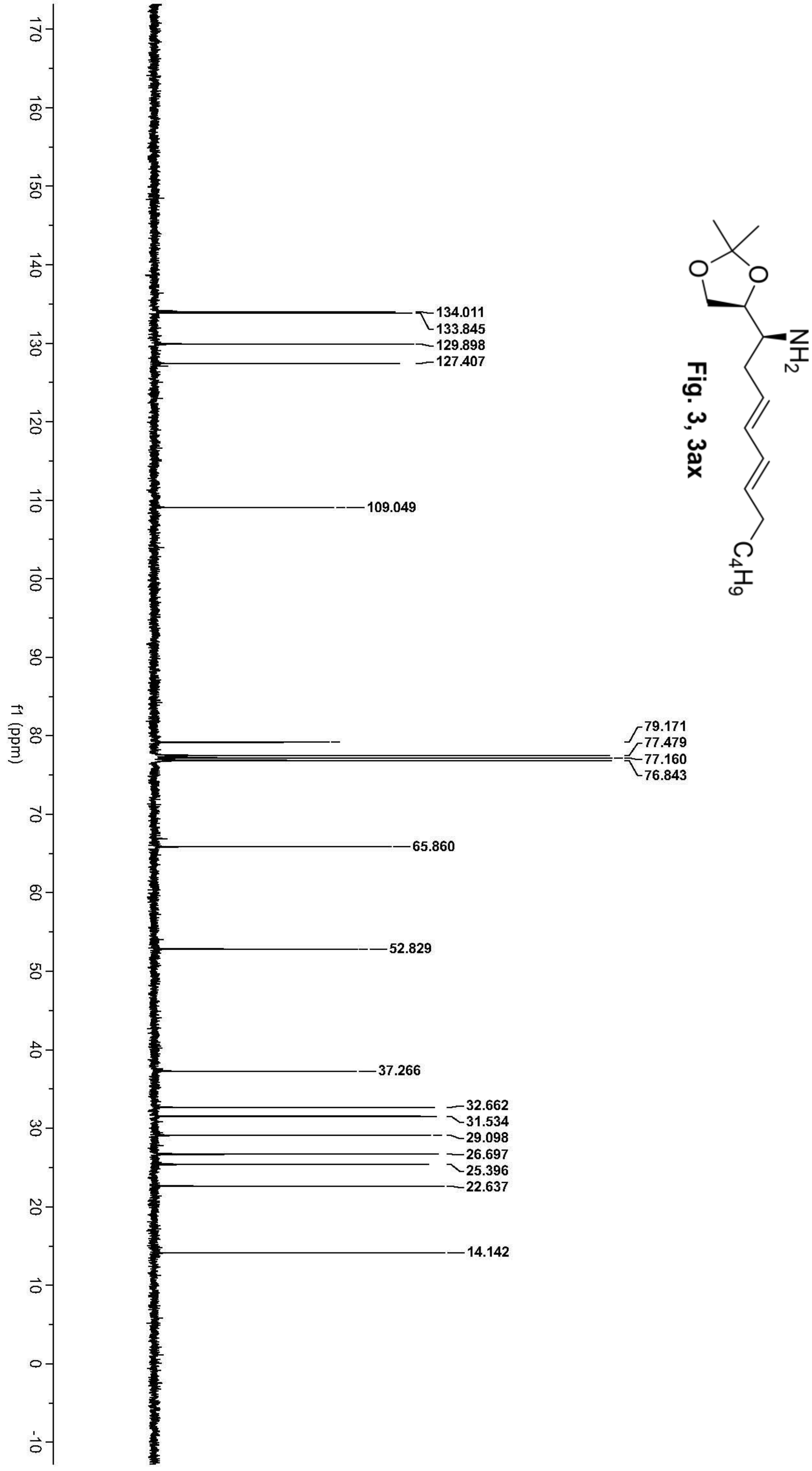

134.011
133.845
129.898
127.407
109.049
79.171
77.479
77.160
76.843
65.860
52.829
37.266
32.662
31.534
29.098
26.697
25.396
22.637
14.142
f1 (ppm)
NH2
C4H9
O
O

Fig. 3, 3ax

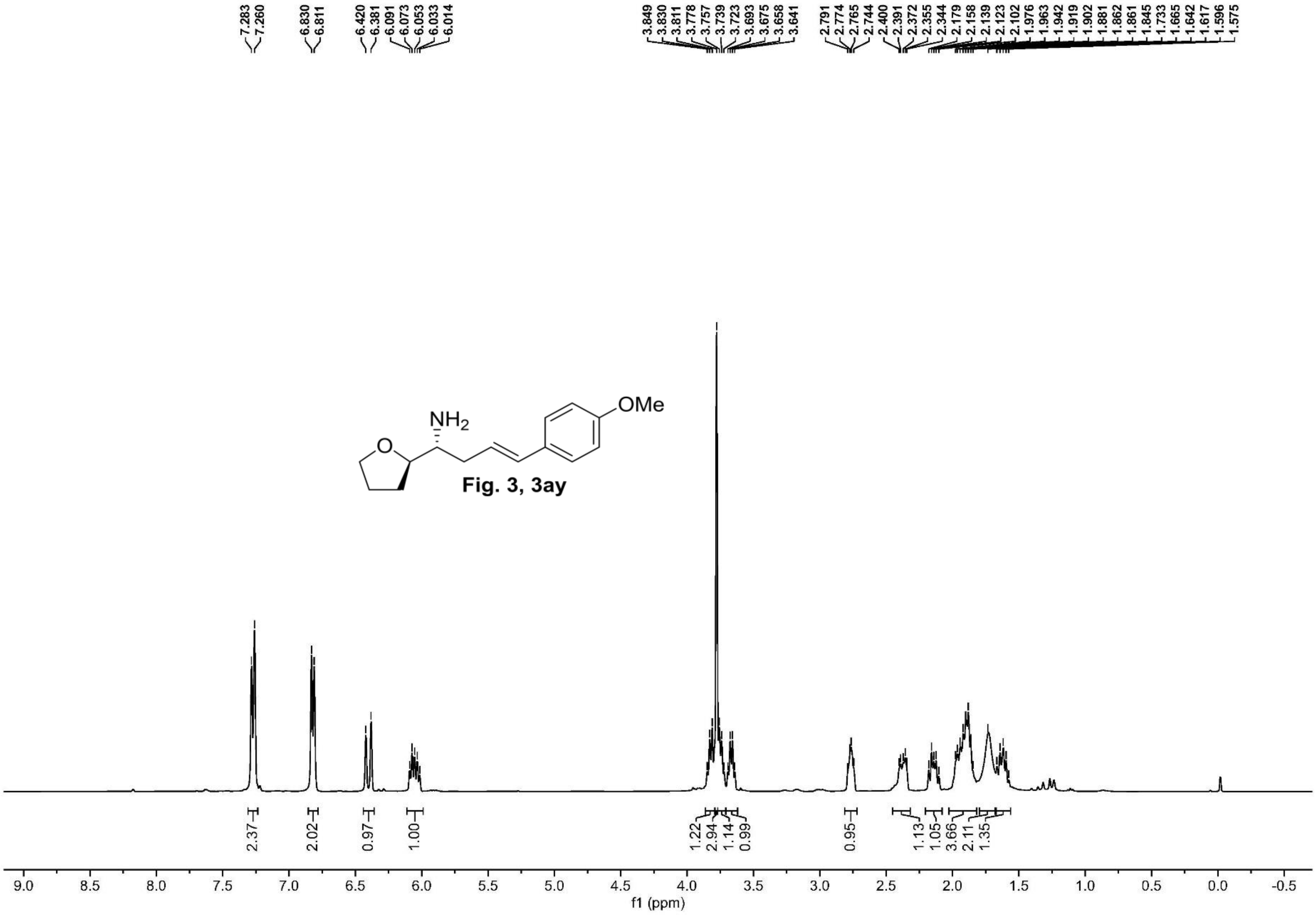
7.283
7.260
6.830
6.811
6.420
6.381
6.091
6.073
6.053
6.033
6.014
3.849
3.830
3.811
3.778
3.757
3.739
3.723
3.693
3.675
3.658
3.641
2.791
2.774
2.765
2.744
2.400
2.391
2.372
2.355
2.344
2.179
2.158
2.139
2.123
2.102
1.976
1.963
1.942
1.919
1.902
1.881
1.862
1.861
1.845
1.733
1.665
1.642
1.617
1.596
1.575
NH2
O
OMe
2.37
2.02
0.97
1.00
1.22
2.94
1.14
0.99
0.95
1.13
1.05
3.66
2.11
1.35
9.0 8.5 8.0 7.5 7.0 6.5 6.0 5.5 5.0 4.5 4.0 3.5 3.0 2.5 2.0 1.5 1.0 0.5 0.0 -0.5
f1 (ppm)
Fig. 3, 3ay

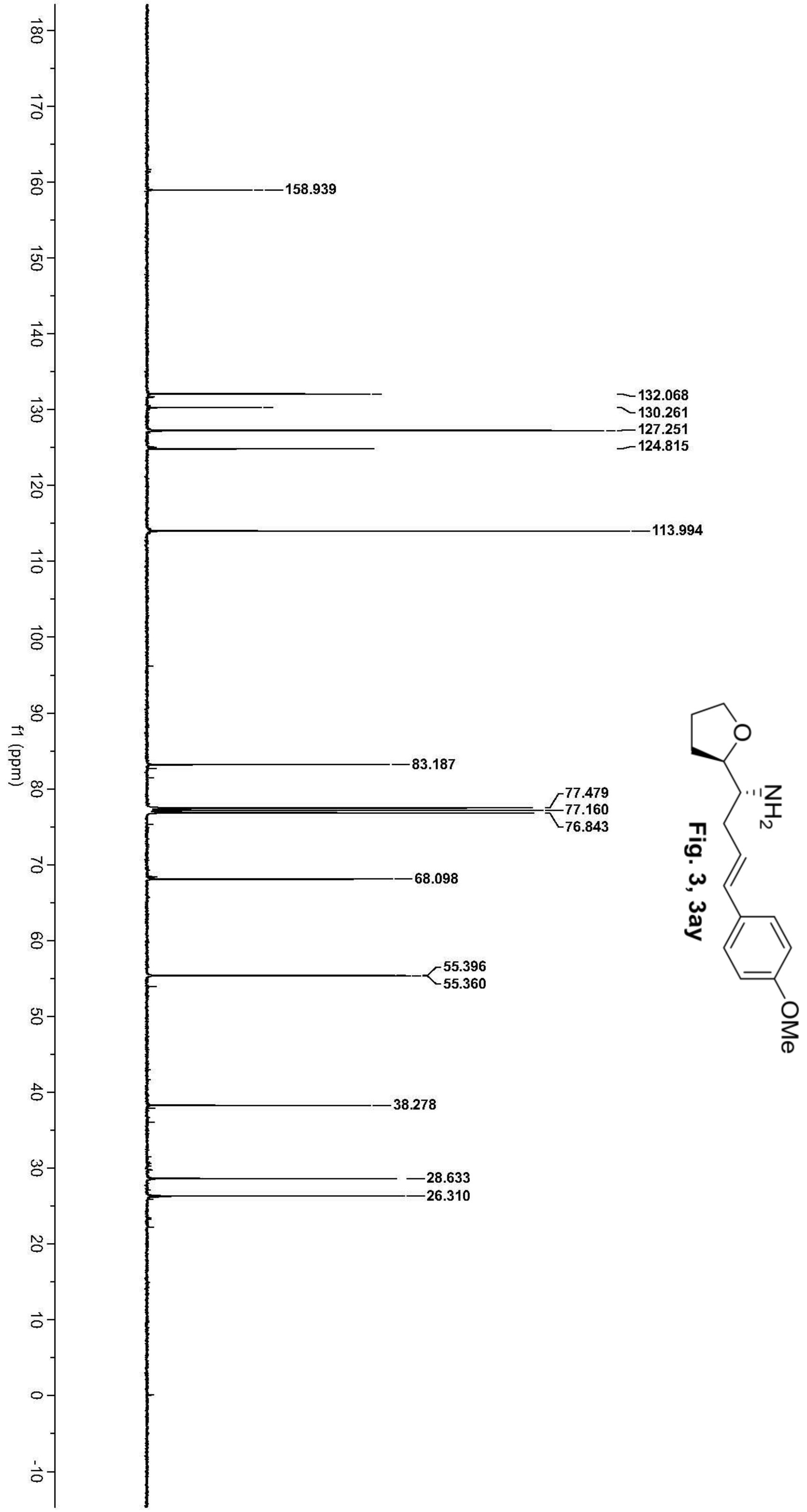
158.939
132.068
130.261
127.251
124.815
113.994
83.187
77.479
77.160
76.843
68.098
55.396
55.360
38.278
28.633
26.310
f1 (ppm)
NH2
O
OMe

Fig. 3, 3ay

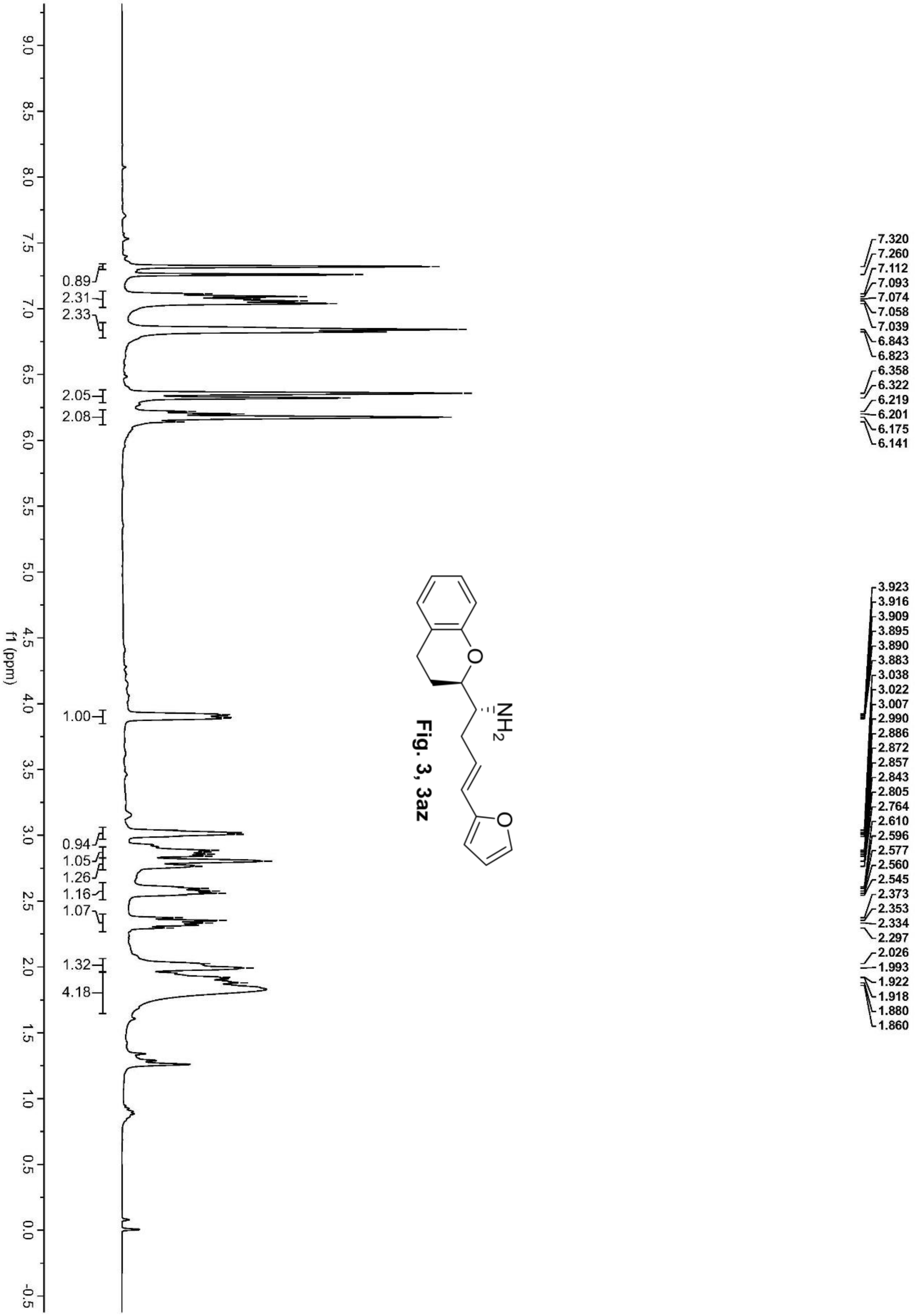

9.0
8.5
8.0
7.5
7.0
6.5
6.0
5.5
5.0
4.5
4.0
3.5
3.0
2.5
2.0
1.5
1.0
0.5
0.0
-0.5
f1 (ppm)
0.89
2.31
2.33
2.05
2.08
1.00
0.94
1.05
1.26
1.16
1.07
1.32
4.18
7.320
7.260
7.112
7.093
7.074
7.058
7.039
6.843
6.823
6.358
6.322
6.219
6.201
6.175
6.141
3.923
3.916
3.909
3.895
3.890
3.883
3.038
3.022
3.007
2.990
2.886
2.872
2.857
2.843
2.805
2.764
2.610
2.596
2.577
2.560
2.545
2.373
2.353
2.334
2.297
2.026
1.993
1.922
1.918
1.880
1.860
O
NH2

Fig. 3, 3az

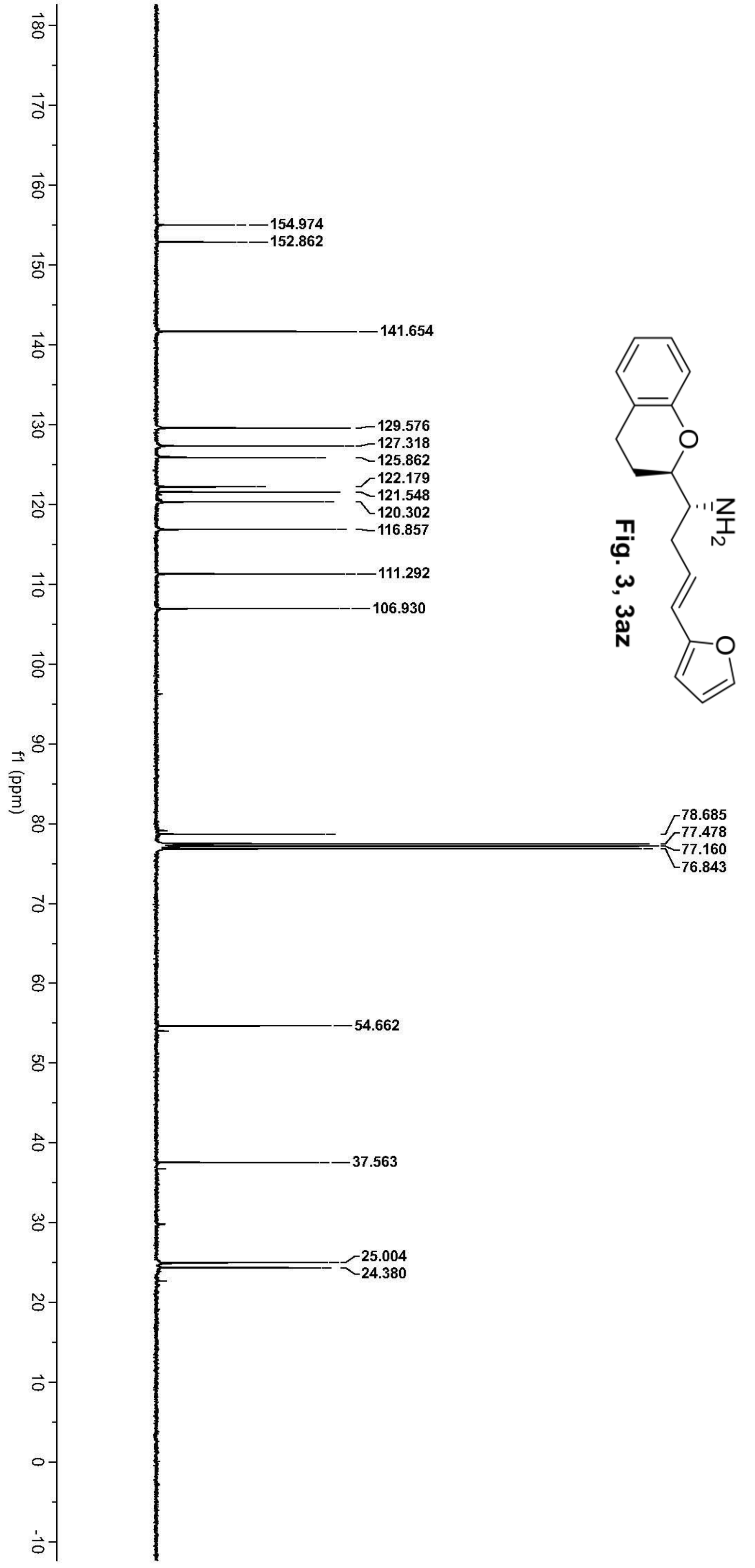
154.974
152.862
141.654
129.576
127.318
125.862
122.179
121.548
120.302
116.857
111.292
106.930
78.685
77.478
77.160
76.843
54.662
37.563
25.004
24.380
NH_2
O
f1 (ppm)
180
170
160
150
140
130
120
110
100
90
80
70
60
50
40
30
20
10
0
-10

Fig. 3, 3az

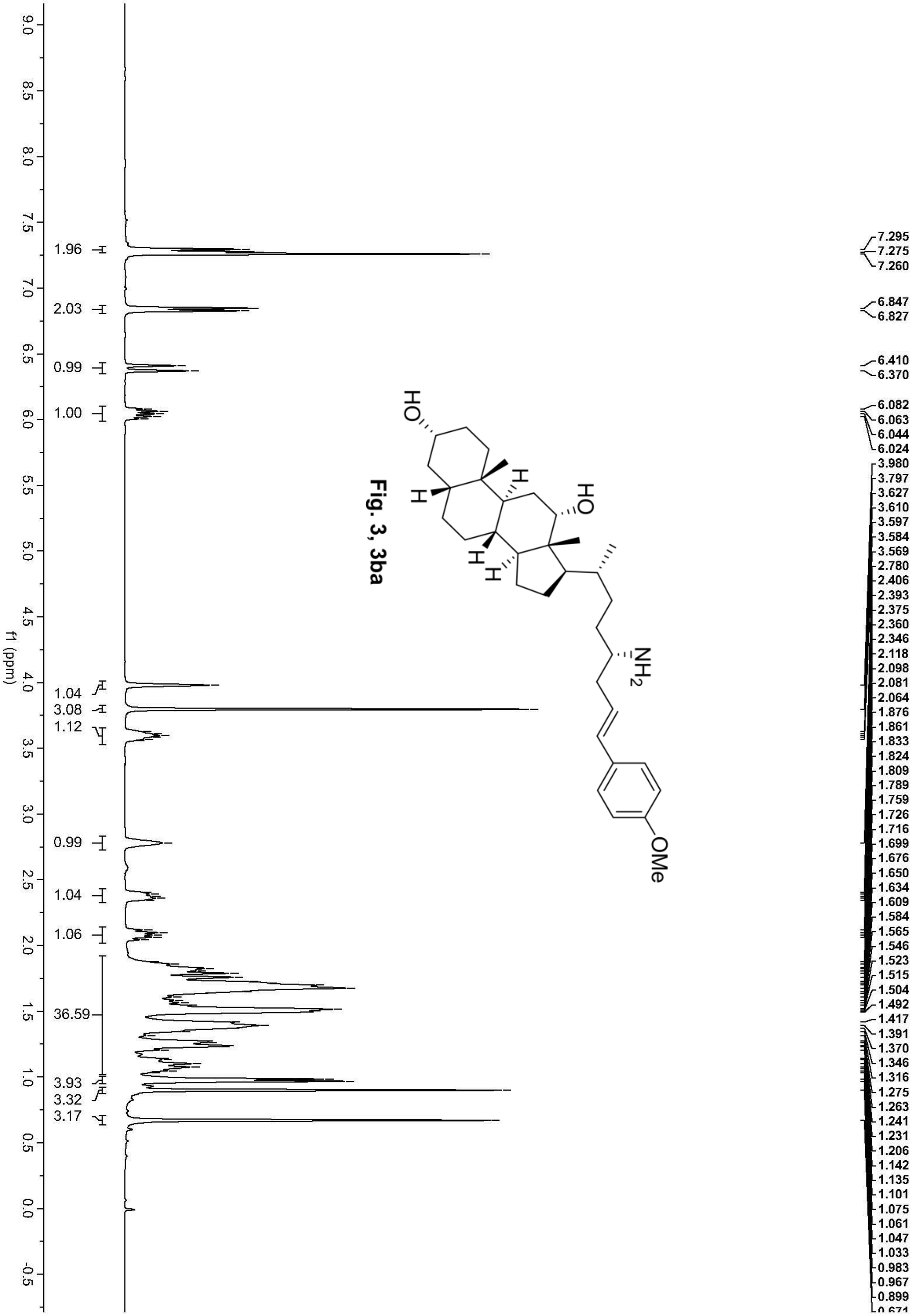
9.0
8.5
8.0
7.5
7.0
6.5
6.0
5.5
5.0
4.5
4.0
3.5
3.0
2.5
2.0
1.5
1.0
0.5
0.0
-0.5
f1 (ppm)
1.96
2.03
0.99
1.00
1.04
3.08
1.12
0.99
1.04
1.06
36.59
3.93
3.32
3.17
HO
H
H
HO
H
H
NH_2
OMe
7.295
7.275
7.260
6.847
6.827
6.410
6.370
6.082
6.063
6.044
6.024
3.980
3.797
3.627
3.610
3.597
3.584
3.569
2.780
2.406
2.393
2.375
2.360
2.346
2.118
2.098
2.081
2.064
1.876
1.861
1.833
1.824
1.809
1.789
1.759
1.726
1.716
1.699
1.676
1.650
1.634
1.609
1.584
1.565
1.546
1.523
1.515
1.504
1.492
1.417
1.391
1.370
1.346
1.316
1.275
1.263
1.241
1.231
1.206
1.142
1.135
1.101
1.075
1.061
1.047
1.033
0.983
0.967
0.899

Fig. 3, 3ba

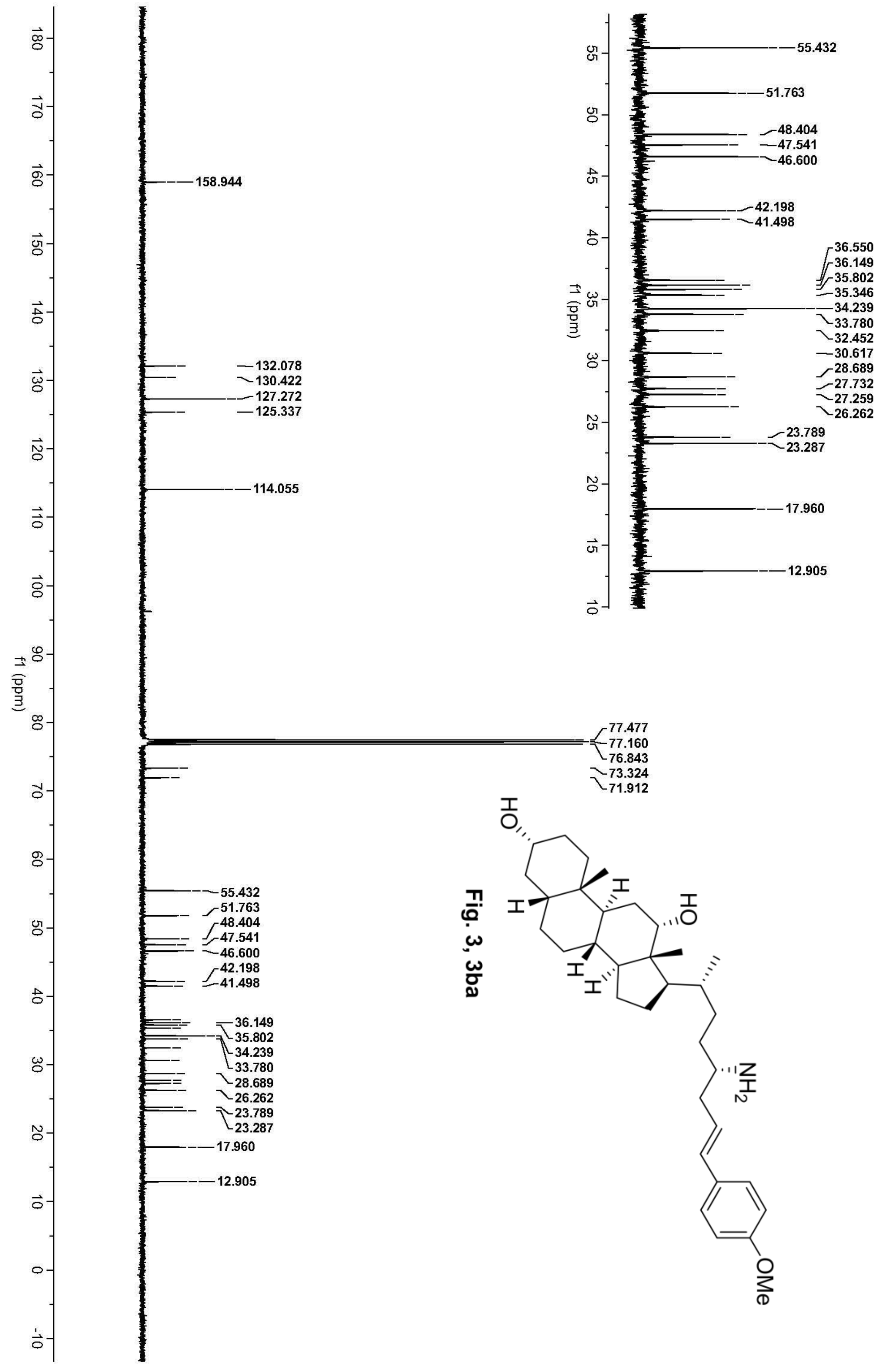
158.944
132.078
130.422
127.272
125.337
114.055
77.477
77.160
76.843
73.324
71.912
55.432
51.763
48.404
47.541
46.600
42.198
41.498
36.550
36.149
35.802
35.346
34.239
33.780
32.452
30.617
28.689
27.732
27.259
26.262
23.789
23.287
17.960
12.905
f1 (ppm)
HO
H
NH2
OMe

Fig. 3, 3ba

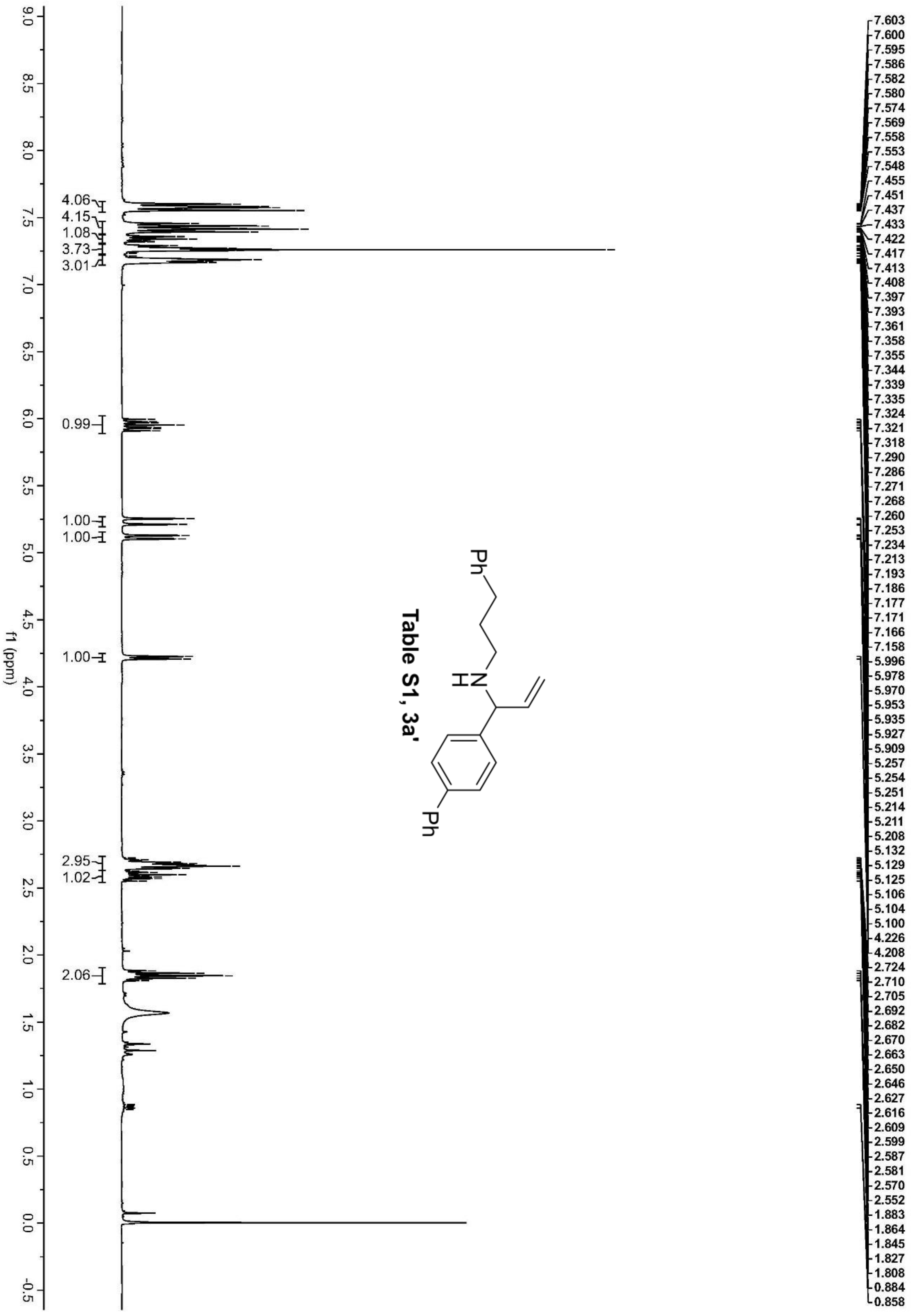
Table S1, 3a'
Ph
N
H
Ph
f1 (ppm)
9.0
8.5
8.0
7.5
7.0
6.5
6.0
5.5
5.0
4.5
4.0
3.5
3.0
2.5
2.0
1.5
1.0
0.5
0.0
-0.5
4.06
4.15
1.08
3.73
3.01
0.99
1.00
1.00
1.00
2.95
1.02
2.06
7.603
7.600
7.595
7.586
7.582
7.580
7.574
7.569
7.558
7.553
7.548
7.455
7.451
7.437
7.433
7.422
7.417
7.413
7.408
7.397
7.393
7.361
7.358
7.355
7.344
7.339
7.335
7.324
7.321
7.318
7.290
7.286
7.271
7.268
7.260
7.253
7.234
7.213
7.193
7.186
7.177
7.171
7.166
7.158
5.996
5.978
5.970
5.953
5.935
5.927
5.909
5.257
5.254
5.251
5.214
5.211
5.208
5.132
5.129
5.125
5.106
5.104
5.100
4.226
4.208
2.724
2.710
2.705
2.692
2.682
2.670
2.663
2.650
2.646
2.627
2.616
2.609
2.599
2.587
2.581
2.570
2.552
1.883
1.864
1.845
1.827
1.808
0.884
0.858

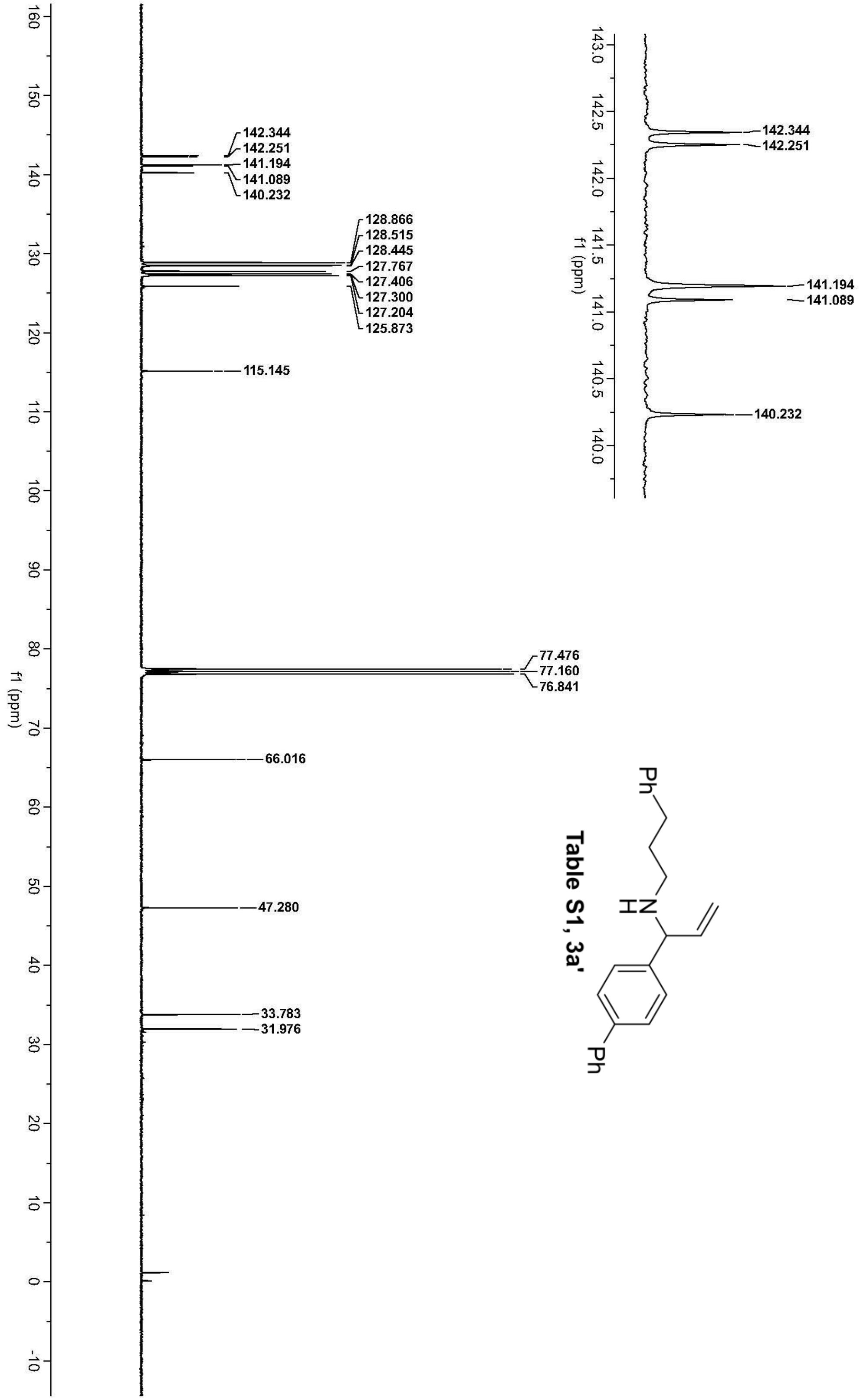
142.344
142.251
141.194
141.089
140.232
128.866
128.515
128.445
127.767
127.406
127.300
127.204
125.873
115.145
77.476
77.160
76.841
66.016
47.280
33.783
31.976
f1 (ppm)
Ph
N
H
Ph

Table S1, 3a'

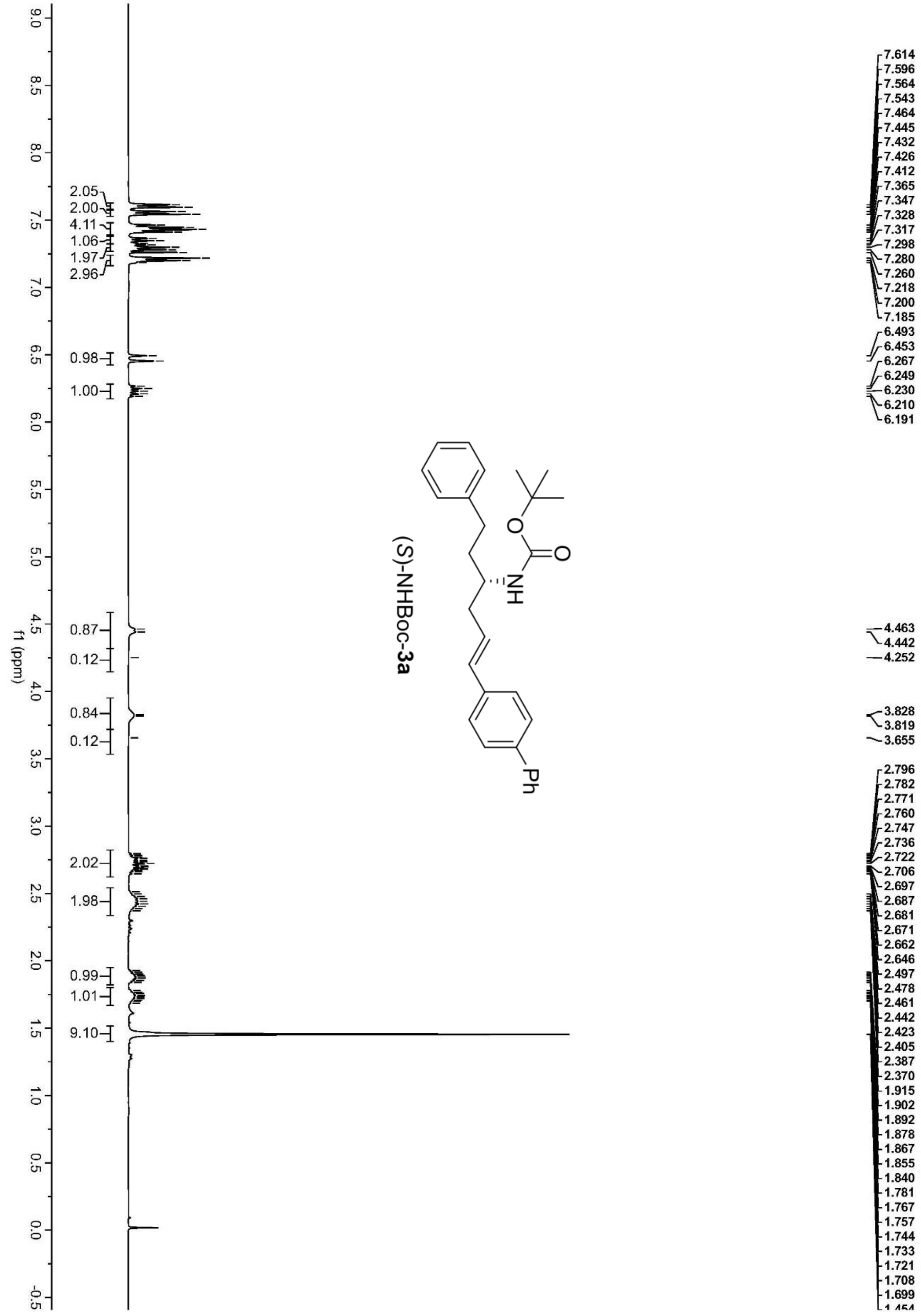
(S)-NHBoc-3a
NH
O
O
Ph
f1 (ppm)
9.0 8.5 8.0 7.5 7.0 6.5 6.0 5.5 5.0 4.5 4.0 3.5 3.0 2.5 2.0 1.5 1.0 0.5 0.0 -0.5
2.05 2.00 4.11 1.06 1.97 2.96 0.98 1.00 0.87 0.12 0.84 0.12 2.02 1.98 0.99 1.01 9.10
7.614 7.596 7.564 7.543 7.464 7.445 7.432 7.426 7.412 7.365 7.347 7.328 7.317 7.298 7.280 7.260 7.218 7.200 7.185
6.493 6.453 6.267 6.249 6.230 6.210 6.191
4.463 4.442 4.252
3.828 3.819 3.655
2.796 2.782 2.771 2.760 2.747 2.736 2.722 2.706 2.697 2.687 2.681 2.671 2.662 2.646 2.497 2.478 2.461 2.442 2.423 2.405 2.387 2.370
1.915 1.902 1.892 1.878 1.867 1.855 1.840 1.781 1.767 1.757 1.744 1.733 1.721 1.708 1.699

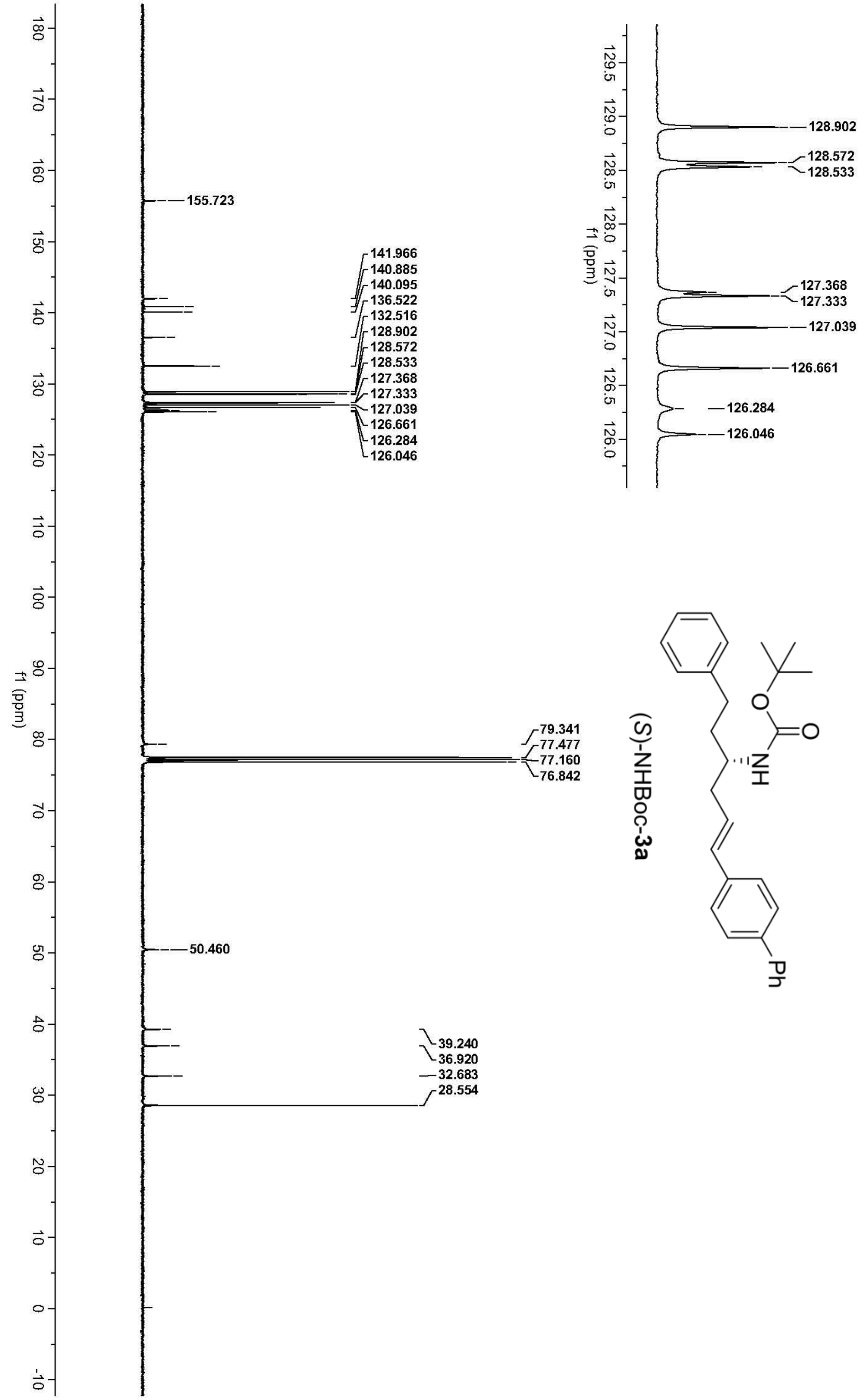
(S)-NHBoc-3a
NH
O
O
Ph
155.723
141.966
140.885
140.095
136.522
132.516
128.902
128.572
128.533
127.368
127.333
127.039
126.661
126.284
126.046
79.341
77.477
77.160
76.842
50.460
39.240
36.920
32.683
28.554
f1 (ppm)

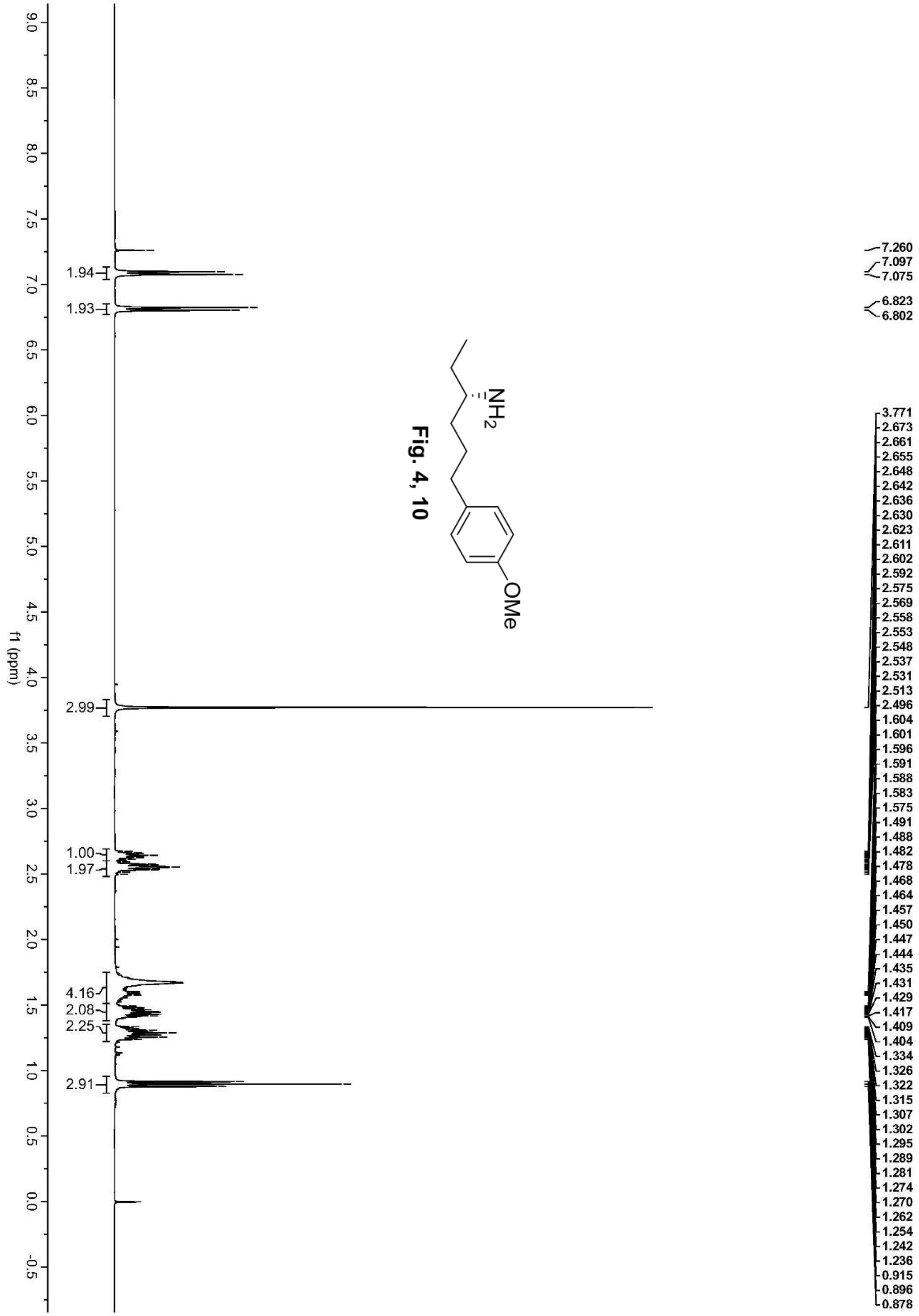
9.0
8.5
8.0
7.5
7.0
6.5
6.0
5.5
5.0
4.5
4.0
3.5
3.0
2.5
2.0
1.5
1.0
0.5
0.0
-0.5
f1 (ppm)
1.94
1.93
2.99
1.00
1.97
4.16
2.08
2.25
2.91
NH2
OMe
7.260
7.097
7.075
6.823
6.802
3.771
2.673
2.661
2.655
2.648
2.642
2.636
2.630
2.623
2.611
2.602
2.592
2.575
2.569
2.558
2.553
2.548
2.537
2.531
2.513
2.496
1.604
1.601
1.596
1.591
1.588
1.583
1.575
1.491
1.488
1.482
1.478
1.468
1.464
1.457
1.450
1.447
1.444
1.435
1.431
1.429
1.417
1.409
1.404
1.334
1.326
1.322
1.315
1.307
1.302
1.295
1.289
1.281
1.274
1.270
1.262
1.254
1.242
1.236
0.915
0.896
0.878
Fig. 4, 10

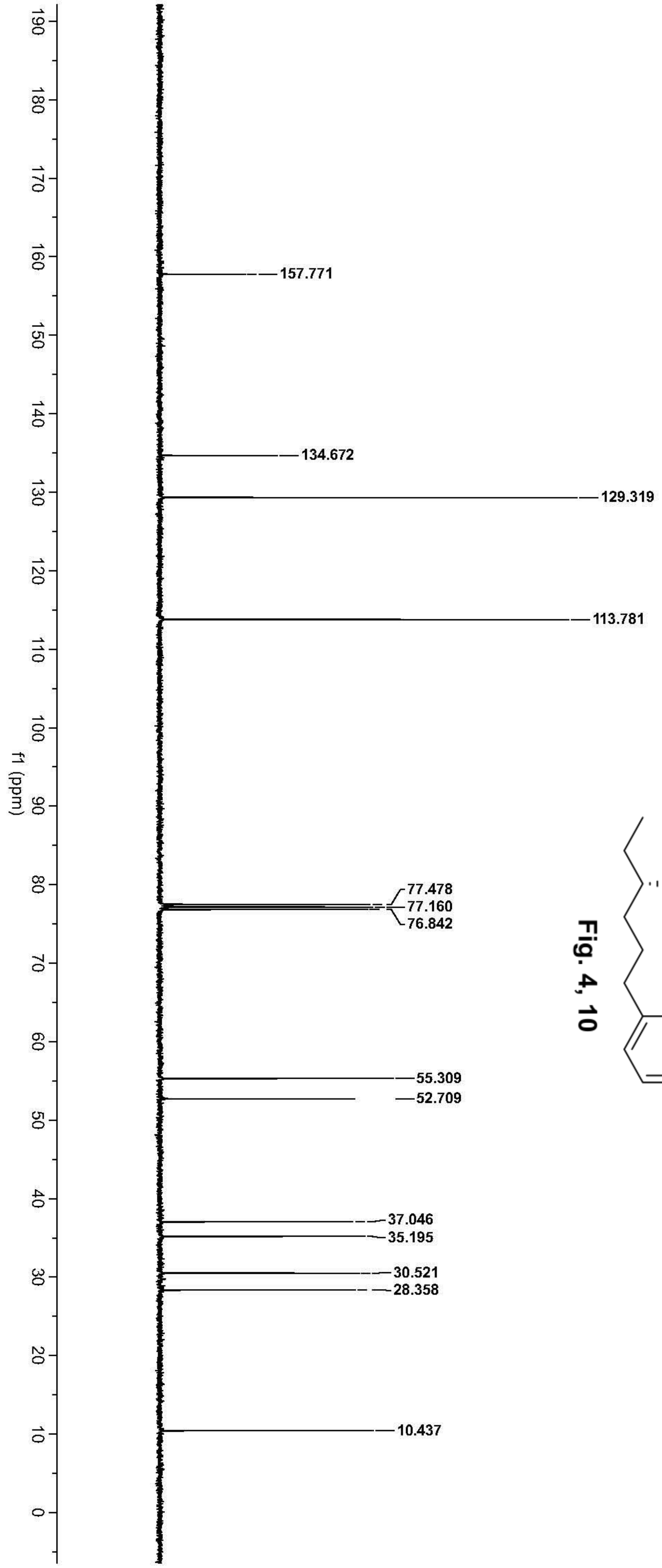
157.771
134.672
129.319
113.781
77.478
77.160
76.842
55.309
52.709
37.046
35.195
30.521
28.358
10.437
f1 (ppm)

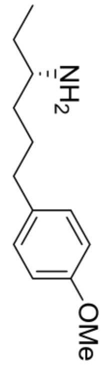
NH2
OMe

Fig. 4, 10

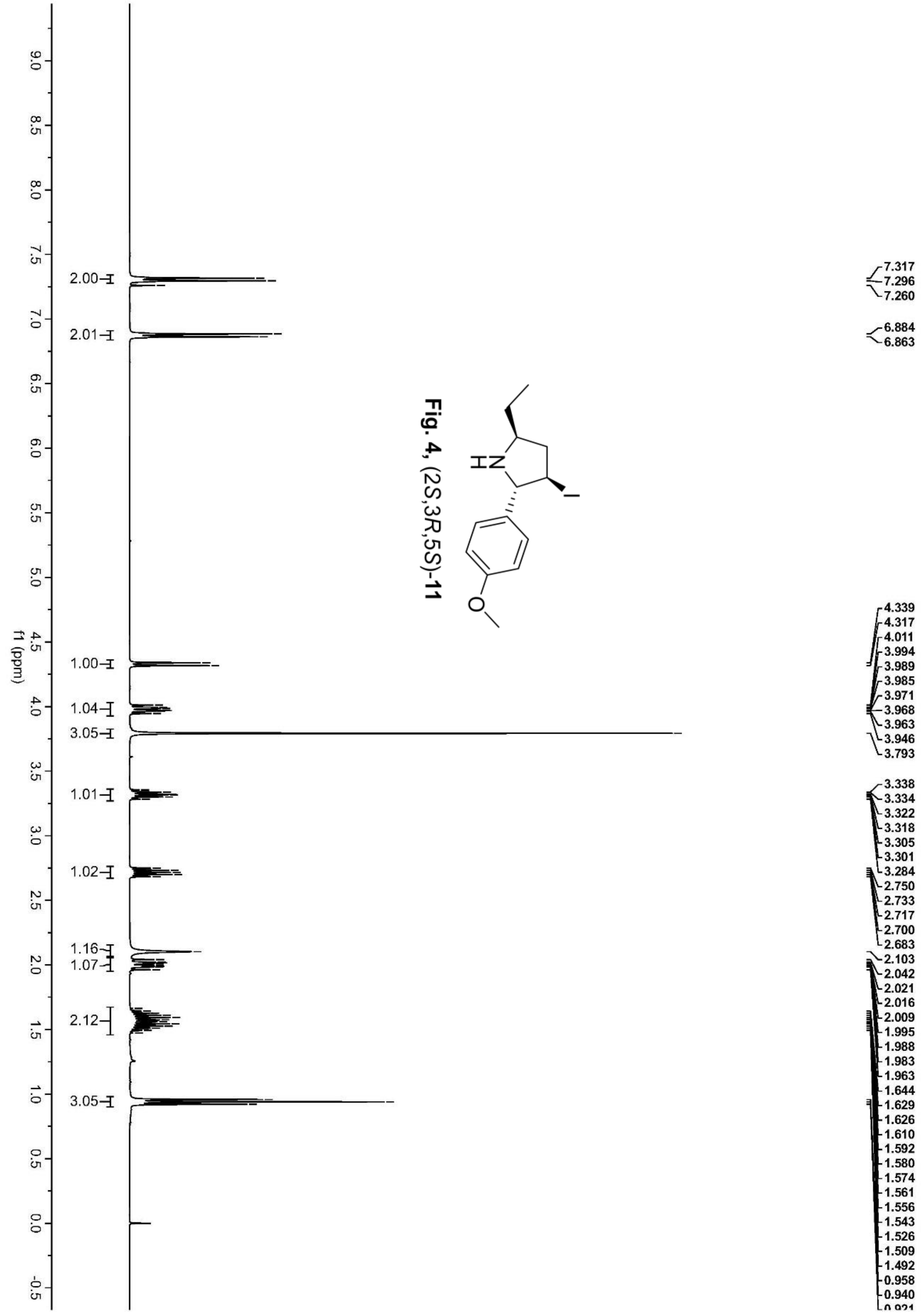

Fig. 4, (2*S*,3*R*,5*S*)-**11**

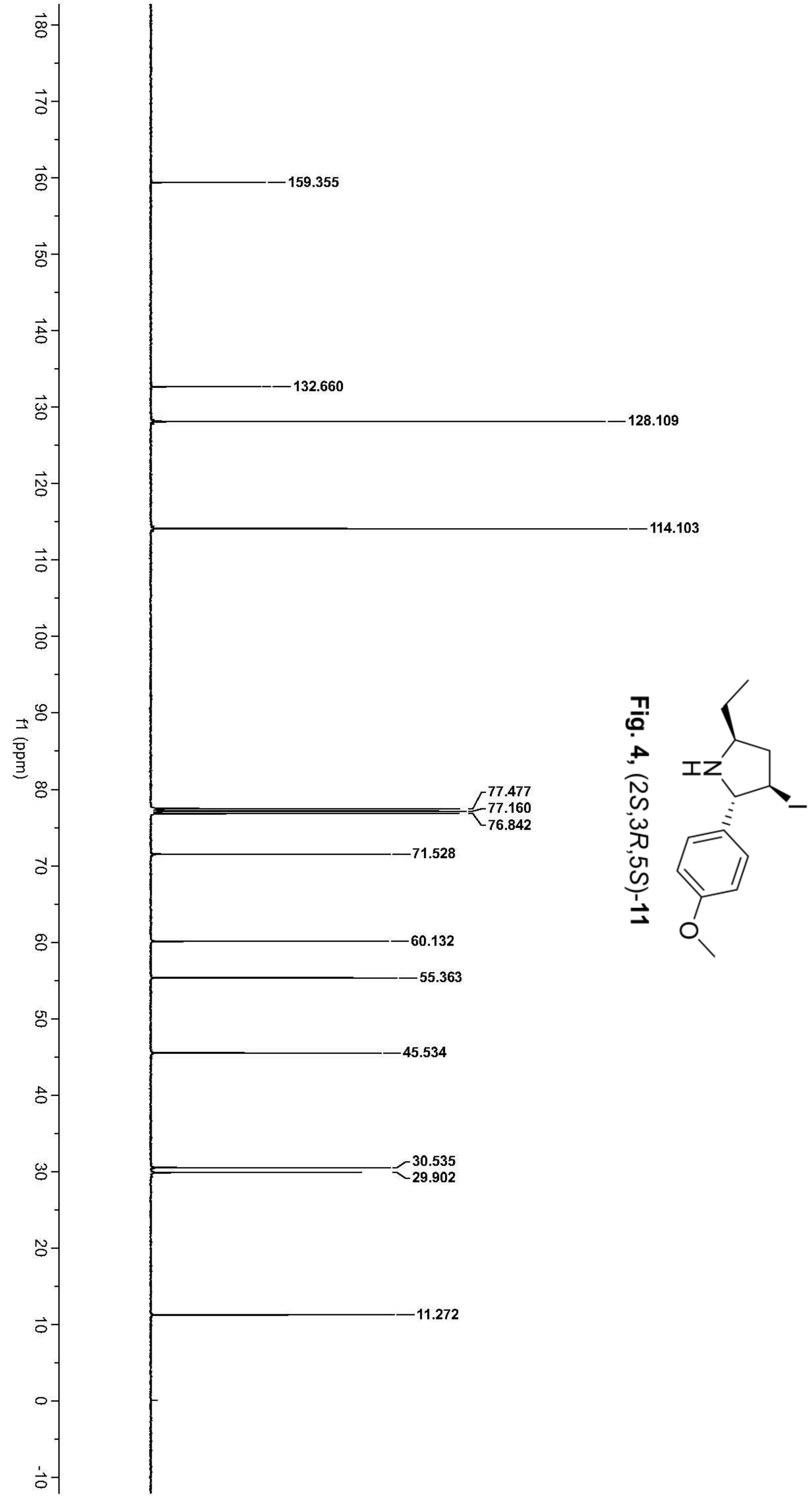

Fig. 4, (2*S*,3*R*,5*S*)-**11**

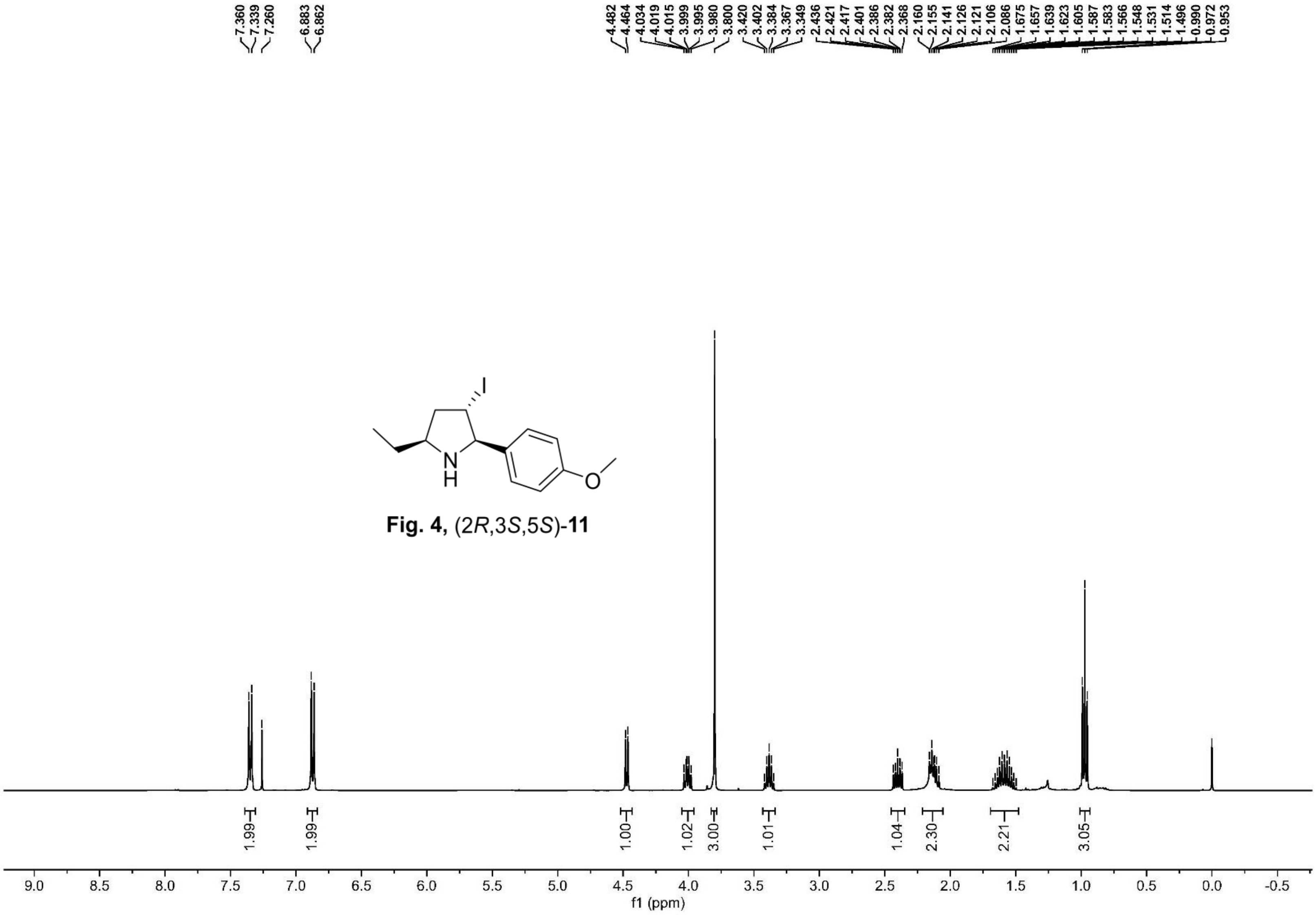

Fig. 4, (2*R*,3*S*,5*S*)-**11**

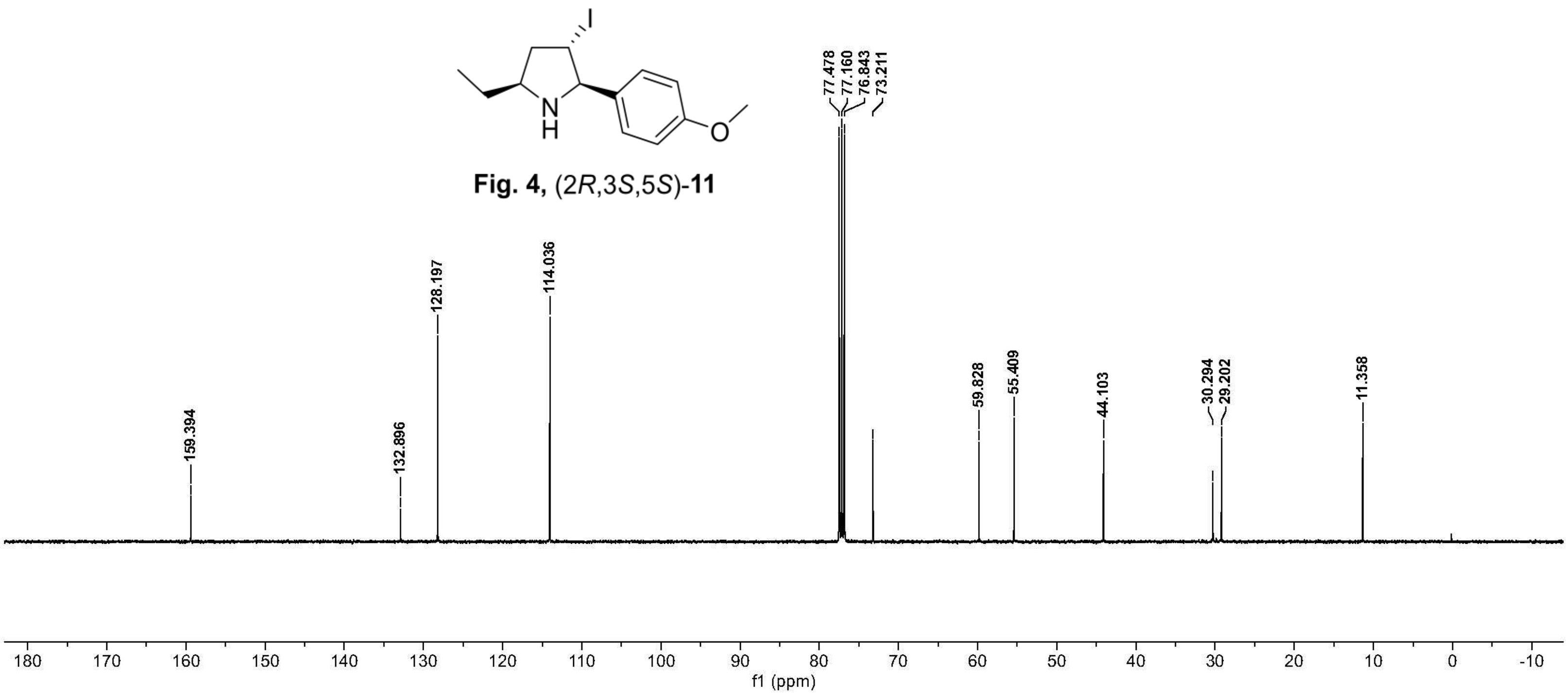

Fig. 4, (2*R*,3*S*,5*S*)-**11**

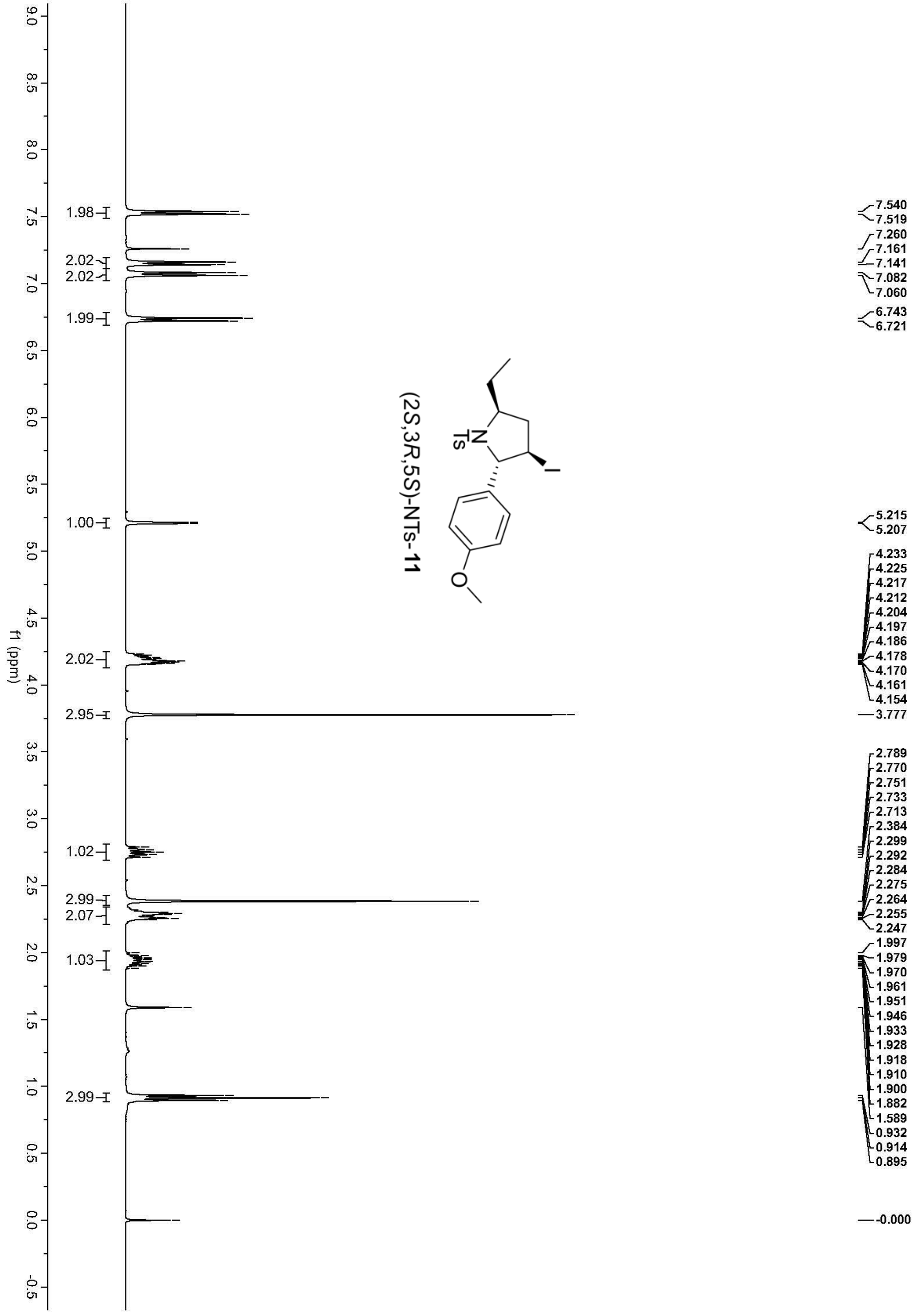
(2S,3R,5S)-NTs-11
N
Ts
I
O
f1 (ppm)
9.0
8.5
8.0
7.5
7.0
6.5
6.0
5.5
5.0
4.5
4.0
3.5
3.0
2.5
2.0
1.5
1.0
0.5
0.0
-0.5
1.98
2.02
2.02
1.99
1.00
2.02
2.95
1.02
2.99
2.07
1.03
2.99
7.540
7.519
7.260
7.161
7.141
7.082
7.060
6.743
6.721
5.215
5.207
4.233
4.225
4.217
4.212
4.204
4.197
4.186
4.178
4.170
4.161
4.154
3.777
2.789
2.770
2.751
2.733
2.713
2.384
2.299
2.292
2.284
2.275
2.264
2.255
2.247
1.997
1.979
1.970
1.961
1.951
1.946
1.933
1.928
1.918
1.910
1.900
1.882
1.589
0.932
0.914
0.895
-0.000

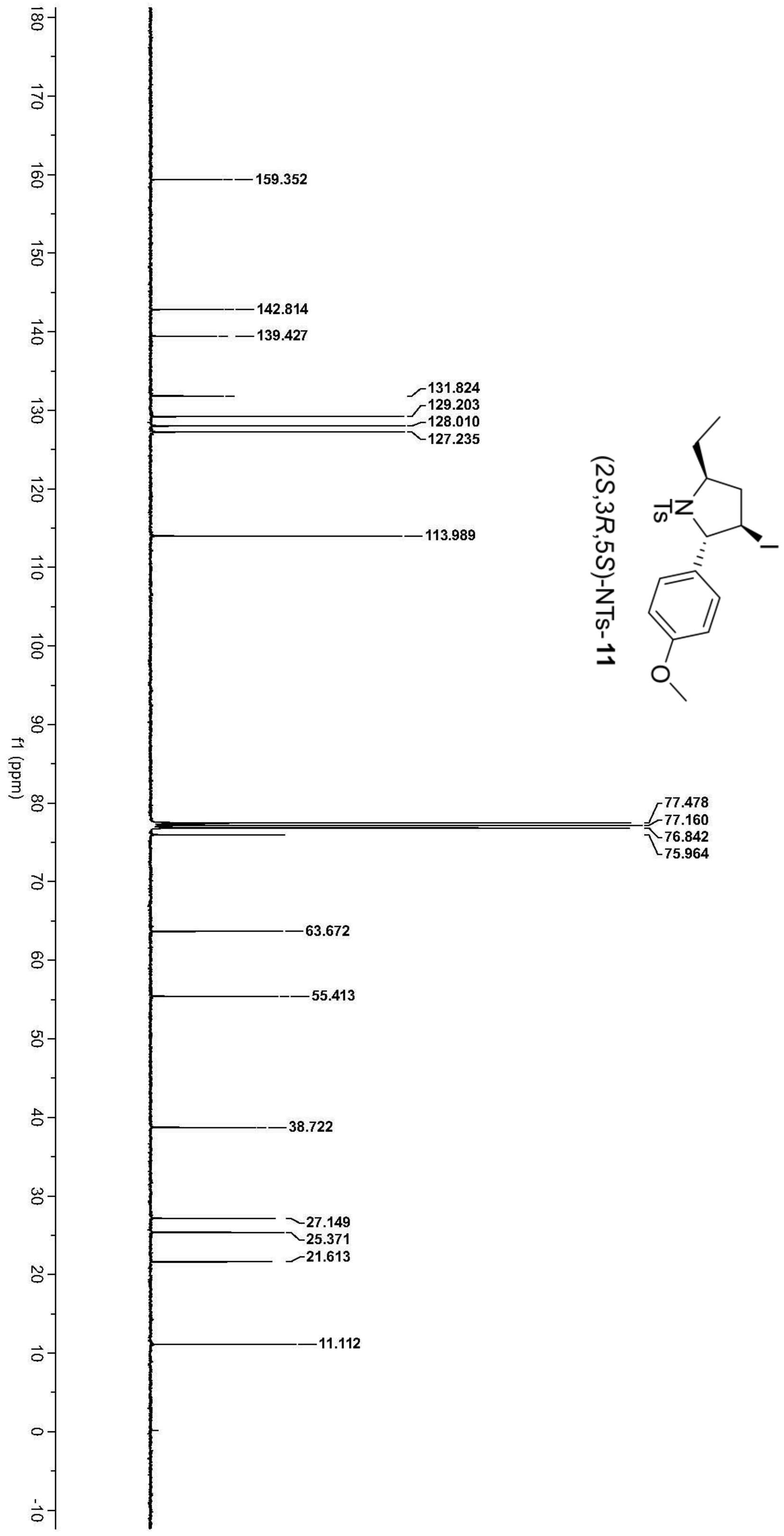
159.352
142.814
139.427
131.824
129.203
128.010
127.235
113.989
77.478
77.160
76.842
75.964
63.672
55.413
38.722
27.149
25.371
21.613
11.112
N
Ts
I
O
(2S,3R,5S)-NTs-11
f1 (ppm)
180
170
160
150
140
130
120
110
100
90
80
70
60
50
40
30
20
10
0
-10

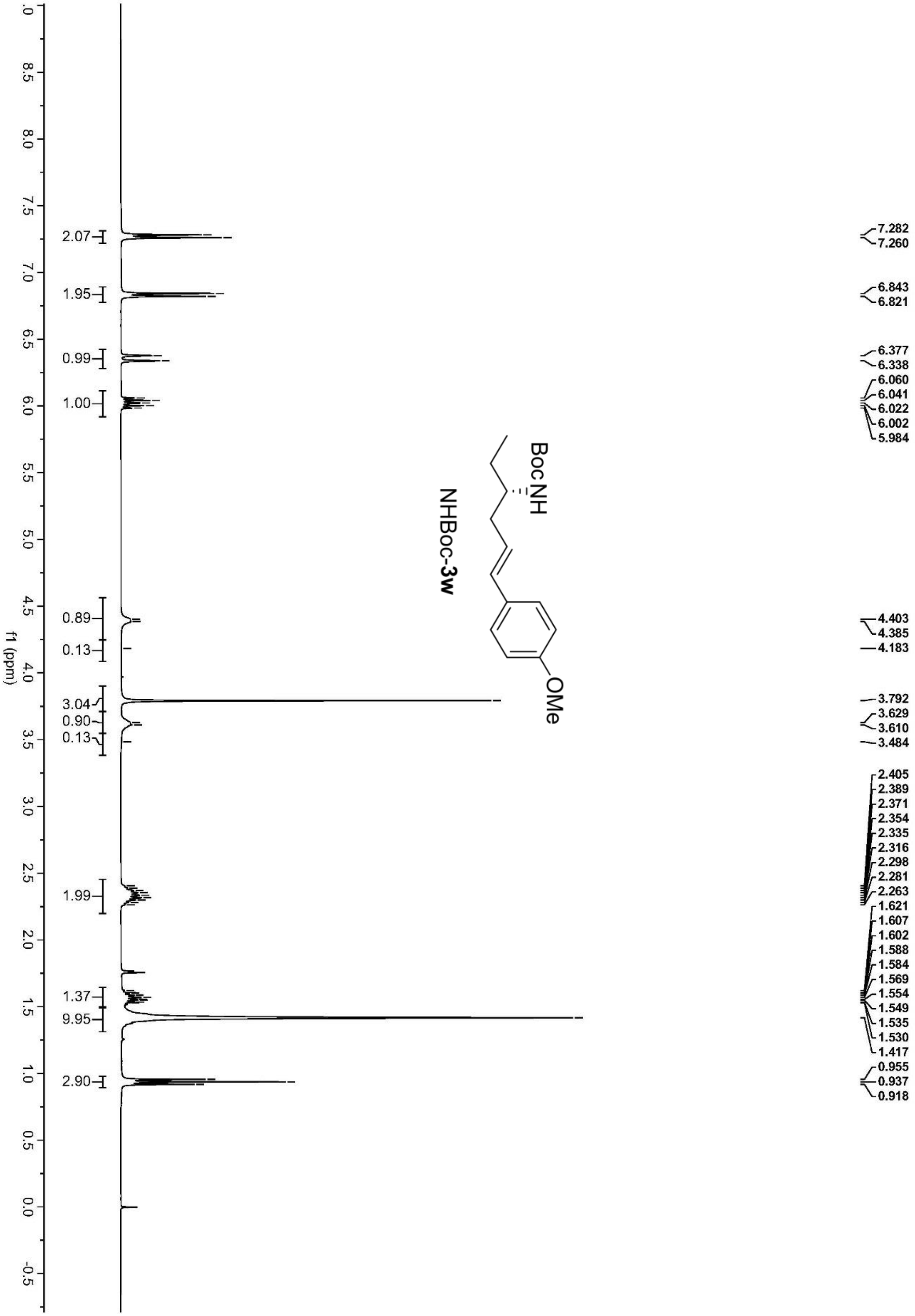
BocNH
OMe
NHBoc-3w
f1 (ppm)
2.07
1.95
0.99
1.00
0.89
0.13
3.04
0.90
0.13
1.99
1.37
9.95
2.90
7.282
7.260
6.843
6.821
6.377
6.338
6.060
6.041
6.022
6.002
5.984
4.403
4.385
4.183
3.792
3.629
3.610
3.484
2.405
2.389
2.371
2.354
2.335
2.316
2.298
2.281
2.263
1.621
1.607
1.602
1.588
1.584
1.569
1.554
1.549
1.535
1.530
1.417
0.955
0.937
0.918

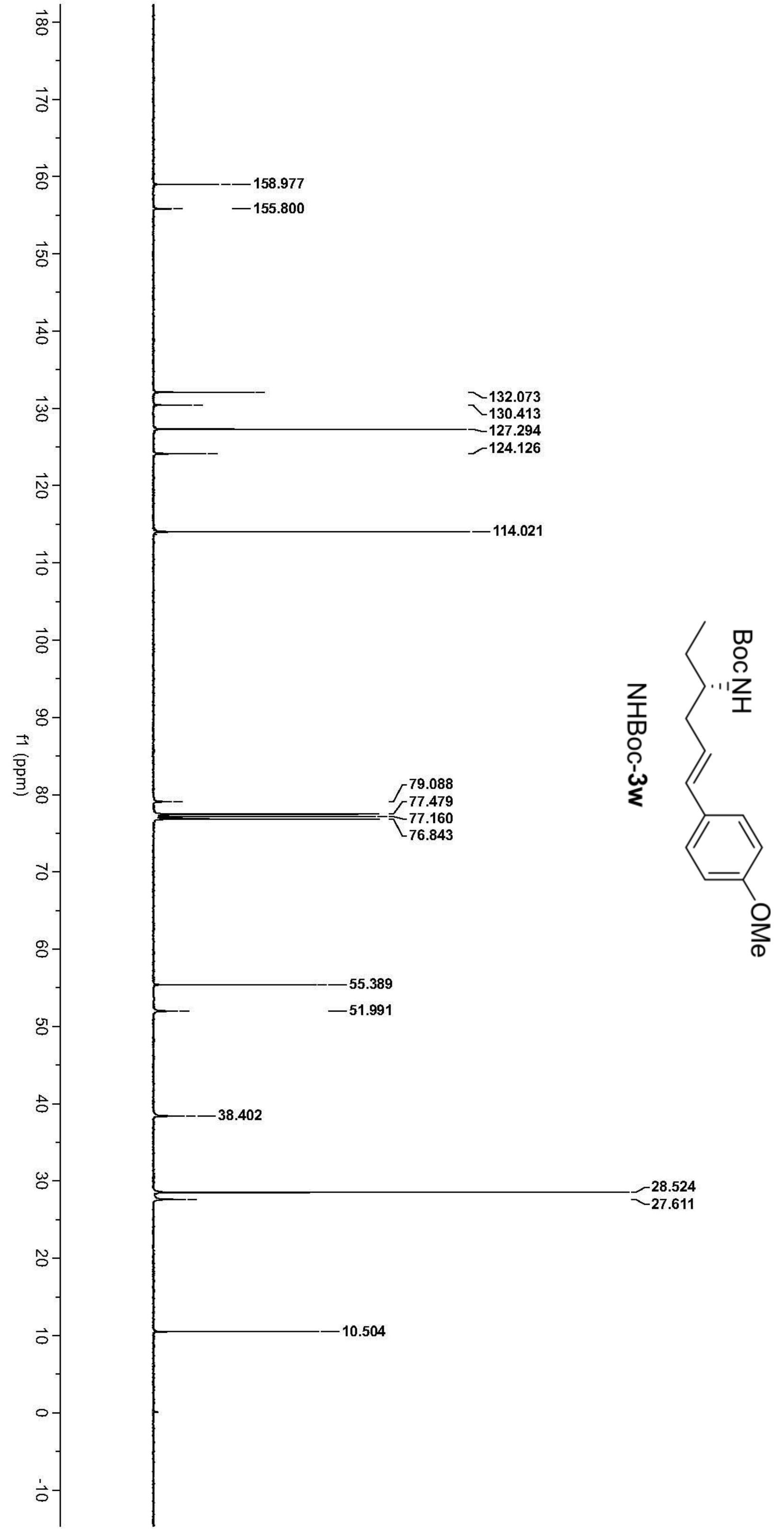
158.977
155.800
132.073
130.413
127.294
124.126
114.021
79.088
77.479
77.160
76.843
55.389
51.991
38.402
28.524
27.611
10.504
f1 (ppm)
BocNH
OMe
NHBoc-3w

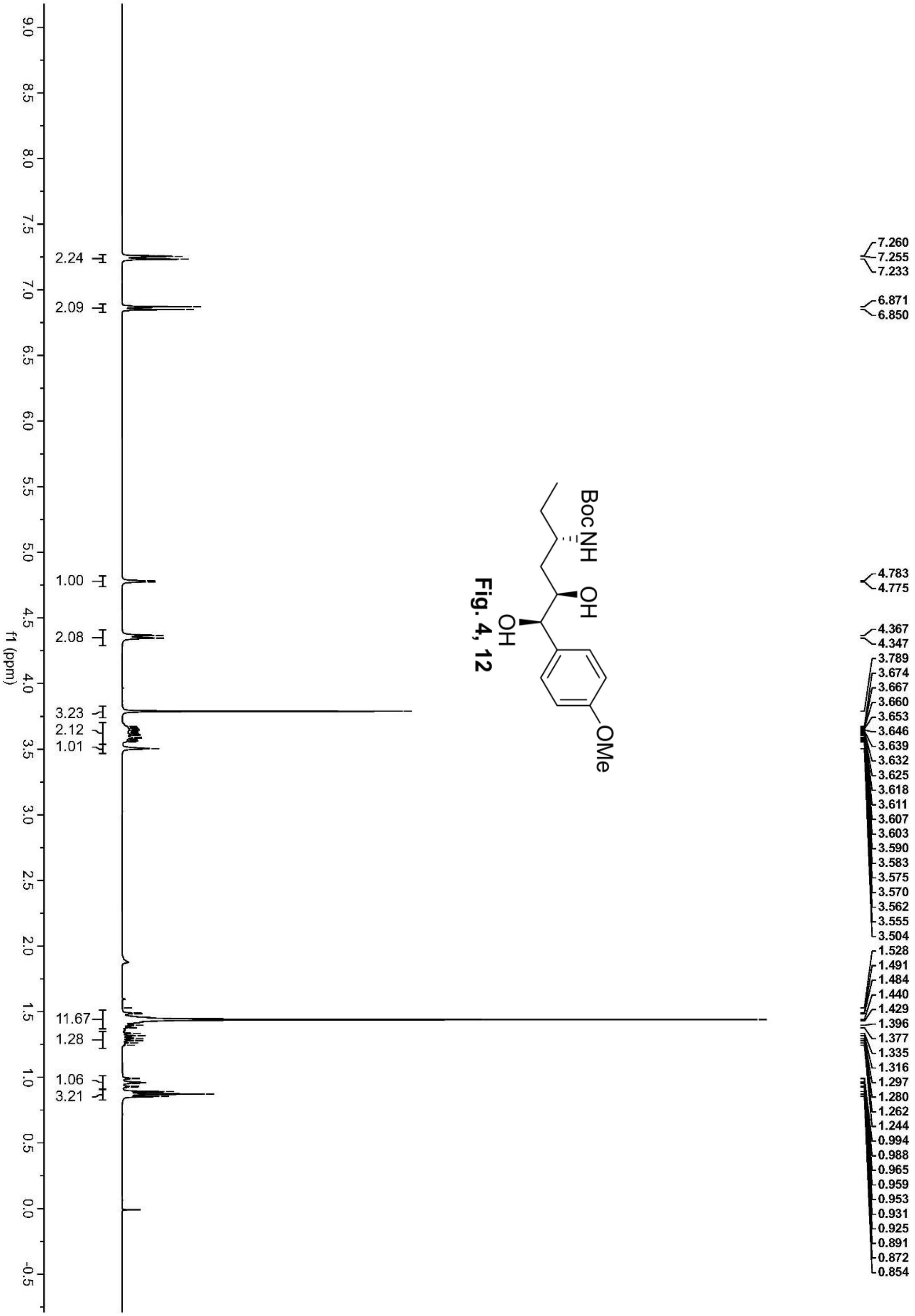

9.0
8.5
8.0
7.5
7.0
6.5
6.0
5.5
5.0
4.5
4.0
3.5
3.0
2.5
2.0
1.5
1.0
0.5
0.0
-0.5
f1 (ppm)
2.24
2.09
1.00
2.08
3.23
2.12
1.01
11.67
1.28
1.06
3.21
7.260
7.255
7.233
6.871
6.850
4.783
4.775
4.367
4.347
3.789
3.674
3.667
3.660
3.653
3.646
3.639
3.632
3.625
3.618
3.611
3.607
3.603
3.590
3.583
3.575
3.570
3.562
3.555
3.504
1.528
1.491
1.484
1.440
1.429
1.396
1.377
1.335
1.316
1.297
1.280
1.262
1.244
0.994
0.988
0.965
0.959
0.953
0.931
0.925
0.891
0.872
0.854
BocNH
OH
OH
OMe

Fig. 4, 12

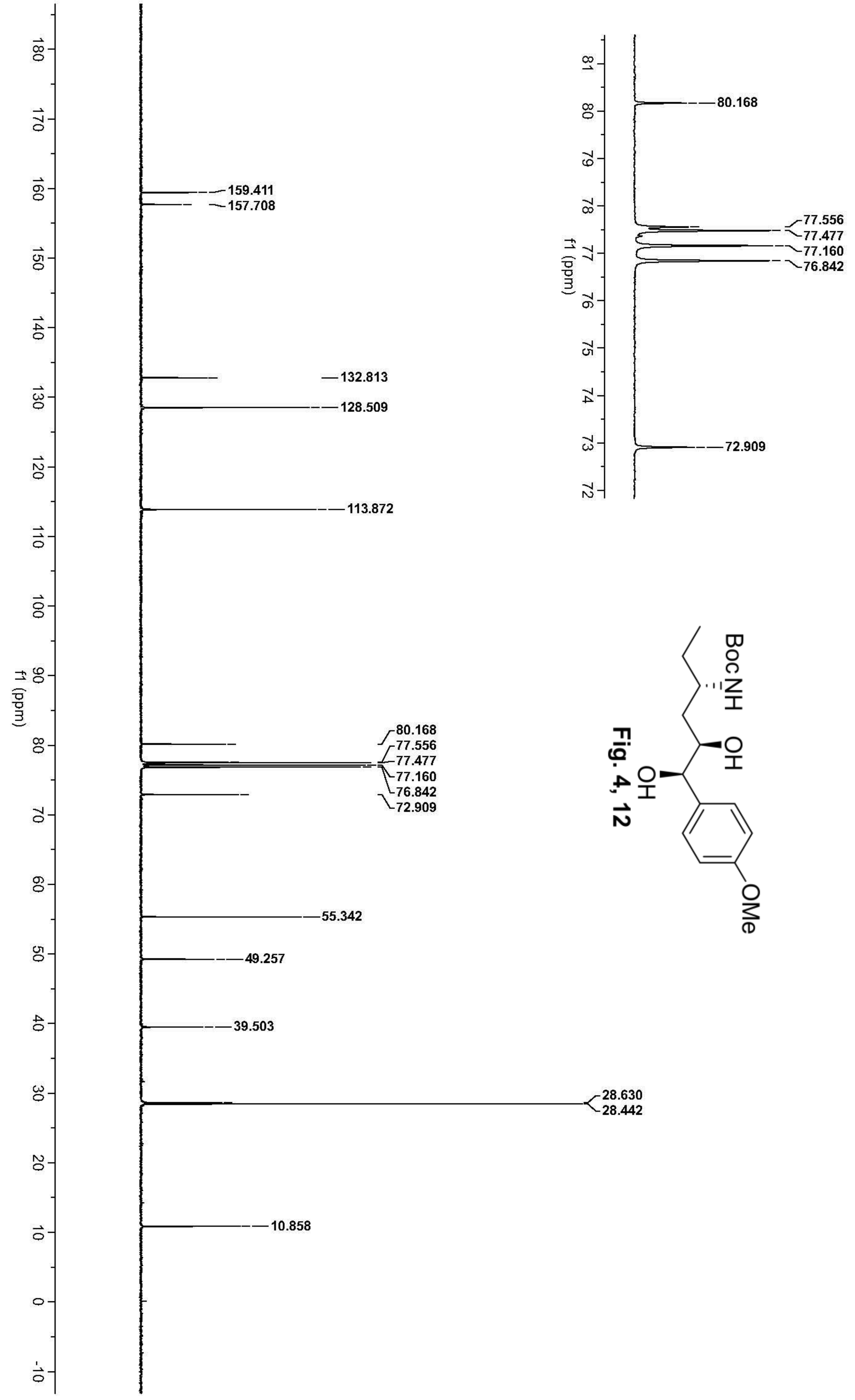
80.168
77.556
77.477
77.160
76.842
72.909
f1 (ppm)
159.411
157.708
132.813
128.509
113.872
55.342
49.257
39.503
28.630
28.442
10.858
BocNH
OH
OH
OMe

Fig. 4, 12

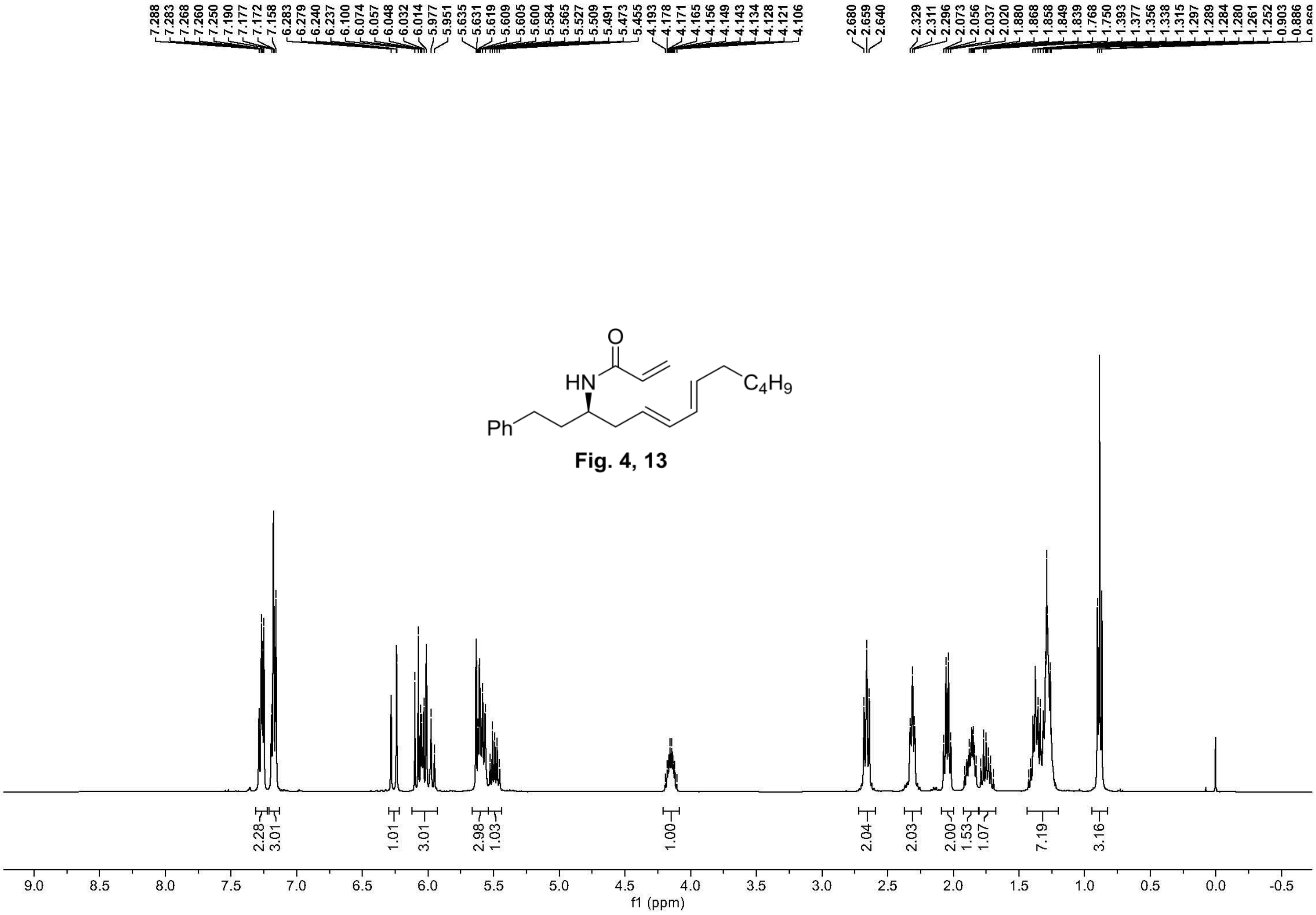
Ph
HN
O
C4H9
7.288
7.283
7.268
7.260
7.250
7.190
7.177
7.172
7.158
6.283
6.279
6.240
6.237
6.100
6.074
6.057
6.048
6.032
6.014
5.977
5.951
5.635
5.631
5.619
5.609
5.605
5.600
5.584
5.565
5.527
5.509
5.491
5.473
5.455
4.193
4.178
4.171
4.165
4.156
4.149
4.143
4.134
4.128
4.121
4.106
2.680
2.659
2.640
2.329
2.311
2.296
2.073
2.056
2.037
2.020
1.880
1.868
1.858
1.849
1.839
1.768
1.750
1.393
1.377
1.356
1.338
1.315
1.297
1.289
1.284
1.280
1.261
1.252
0.903
0.886
2.28
3.01
1.01
3.01
2.98
1.03
1.00
2.04
2.03
2.00
1.53
1.07
7.19
3.16
9.0
8.5
8.0
7.5
7.0
6.5
6.0
5.5
5.0
4.5
4.0
3.5
3.0
2.5
2.0
1.5
1.0
0.5
0.0
-0.5
f1 (ppm)

Fig. 4, 13

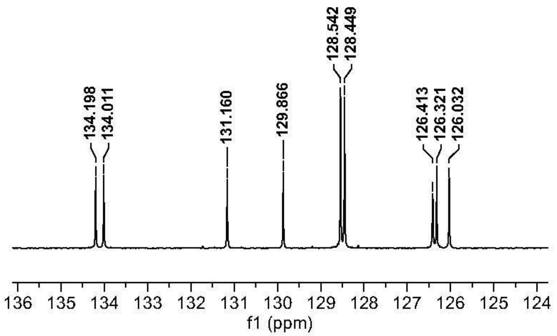
134.198
134.011
131.160
129.866
128.542
128.449
126.413
126.321
126.032
136 135 134 133 132 131 130 129 128 127 126 125 124
f1 (ppm)

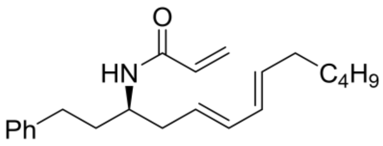
O
HN
Ph
C_4H_9

Fig. 4, 13

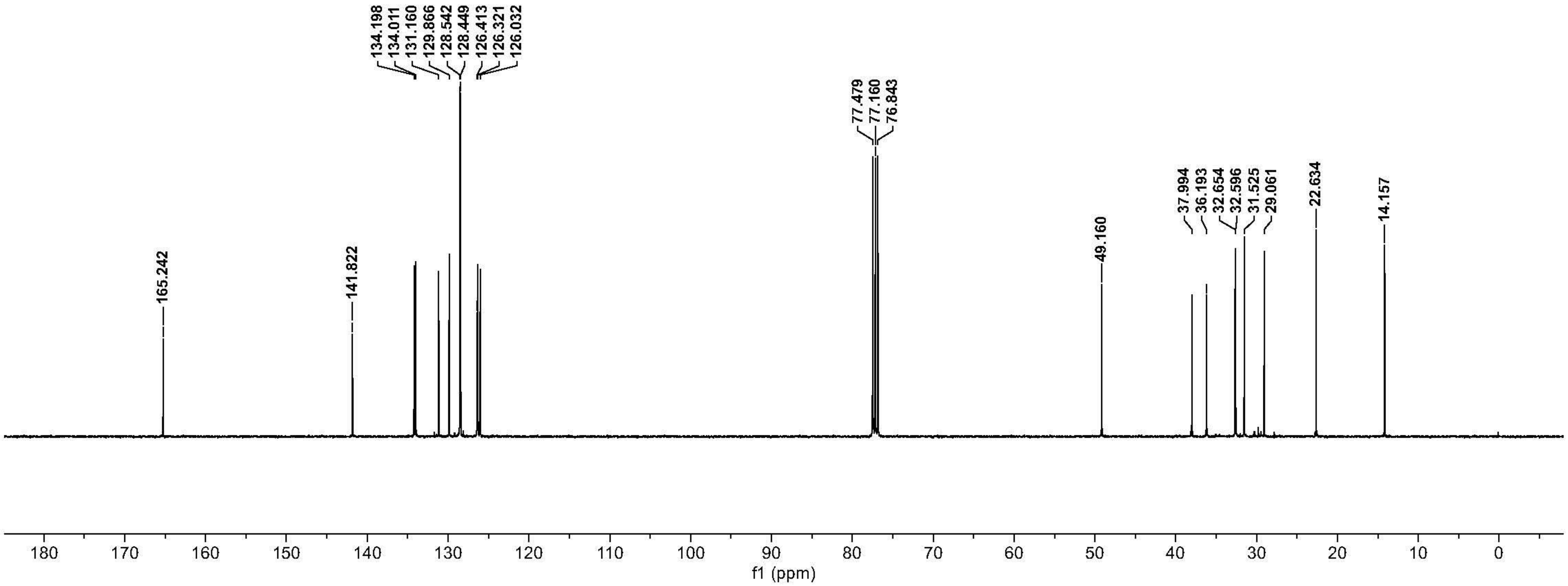
165.242
141.822
134.198
134.011
131.160
129.866
128.542
128.449
126.413
126.321
126.032
77.479
77.160
76.843
49.160
37.994
36.193
32.654
32.596
31.525
29.061
22.634
14.157
180 170 160 150 140 130 120 110 100 90 80 70 60 50 40 30 20 10 0
f1 (ppm)

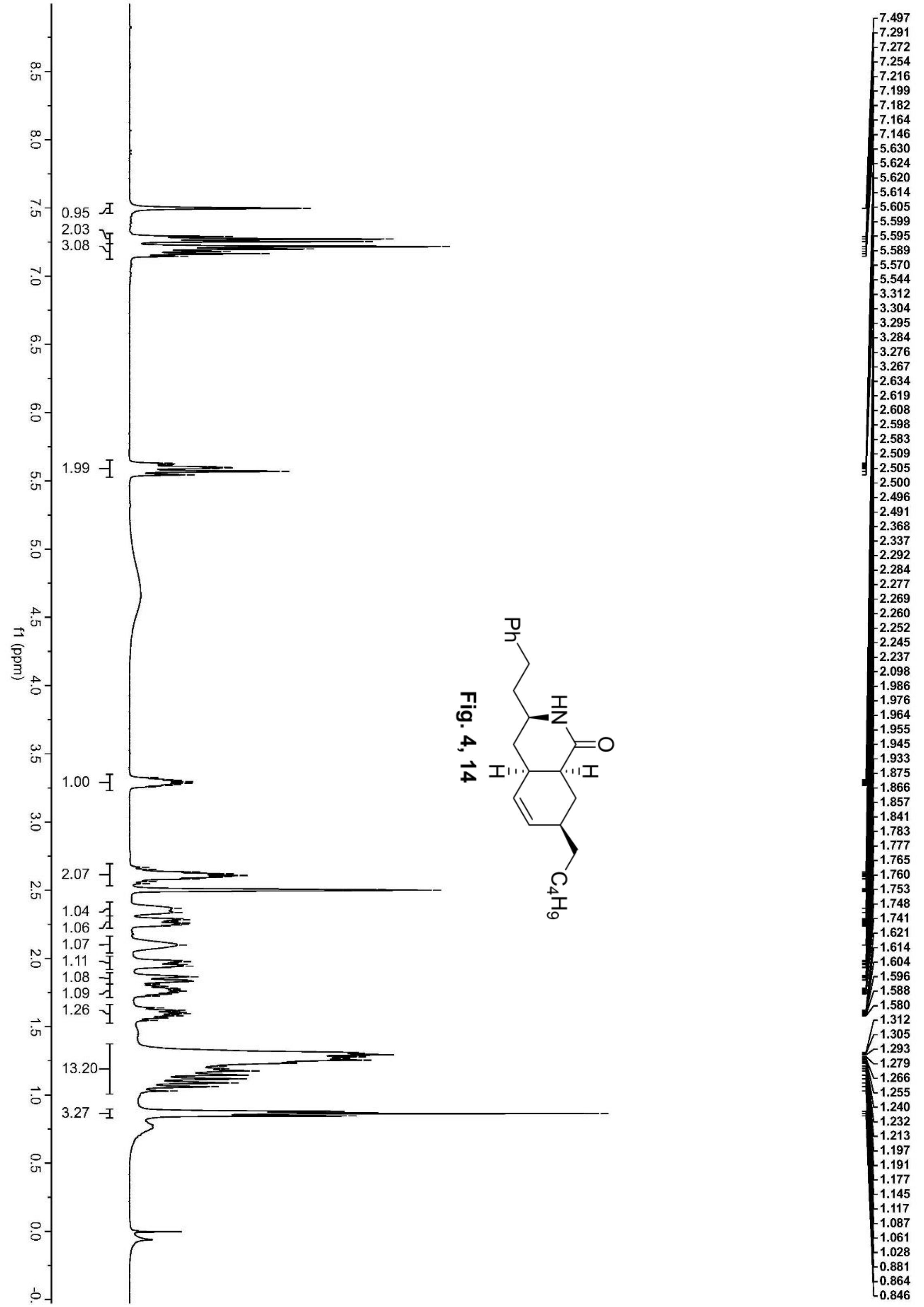
7.497
7.291
7.272
7.254
7.216
7.199
7.182
7.164
7.146
5.630
5.624
5.620
5.614
5.605
5.599
5.595
5.589
5.570
5.544
3.312
3.304
3.295
3.284
3.276
3.267
2.634
2.619
2.608
2.598
2.583
2.509
2.505
2.500
2.496
2.491
2.368
2.337
2.292
2.284
2.277
2.269
2.260
2.252
2.245
2.237
2.098
1.986
1.976
1.964
1.955
1.945
1.933
1.875
1.866
1.857
1.841
1.783
1.777
1.765
1.760
1.753
1.748
1.741
1.621
1.614
1.604
1.596
1.588
1.580
1.312
1.305
1.293
1.279
1.266
1.255
1.240
1.232
1.213
1.197
1.191
1.177
1.145
1.117
1.087
1.061
1.028
0.881
0.864
0.846
0.95
2.03
3.08
1.99
1.00
2.07
1.04
1.06
1.07
1.11
1.08
1.09
1.26
13.20
3.27
8.5
8.0
7.5
7.0
6.5
6.0
5.5
5.0
4.5
4.0
3.5
3.0
2.5
2.0
1.5
1.0
0.5
0.0
-0.
f1 (ppm)
Ph
HN
O
H
H
C_4H_9

Fig. 4, 14

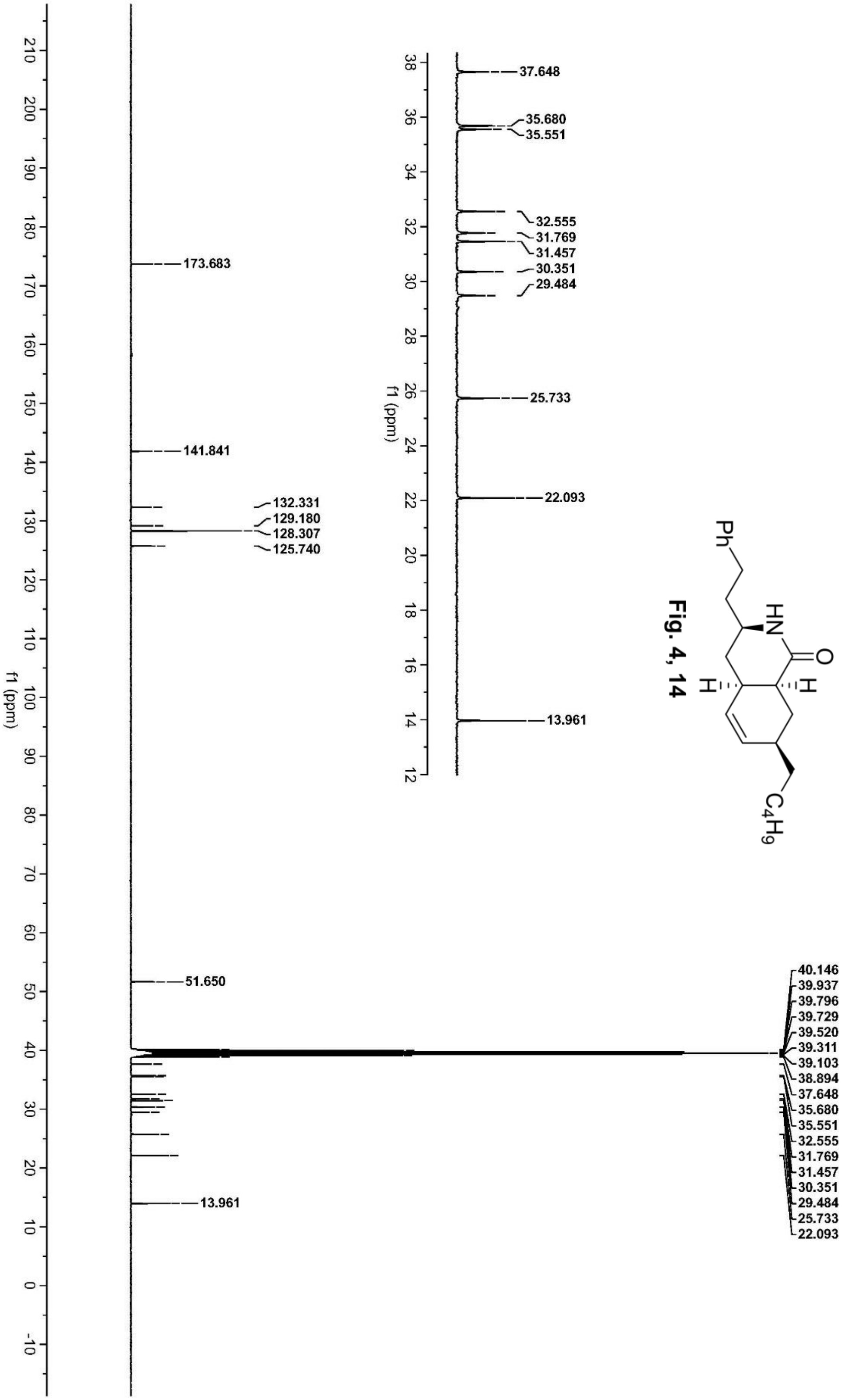
173.683
141.841
132.331
129.180
128.307
125.740
51.650
13.961
37.648
35.680
35.551
32.555
31.769
31.457
30.351
29.484
25.733
22.093
13.961
40.146
39.937
39.796
39.729
39.520
39.311
39.103
38.894
37.648
35.680
35.551
32.555
31.769
31.457
30.351
29.484
25.733
22.093
f1 (ppm)
f1 (ppm)
Ph
HN
O
H
H
C_4H_9

Fig. 4, 14

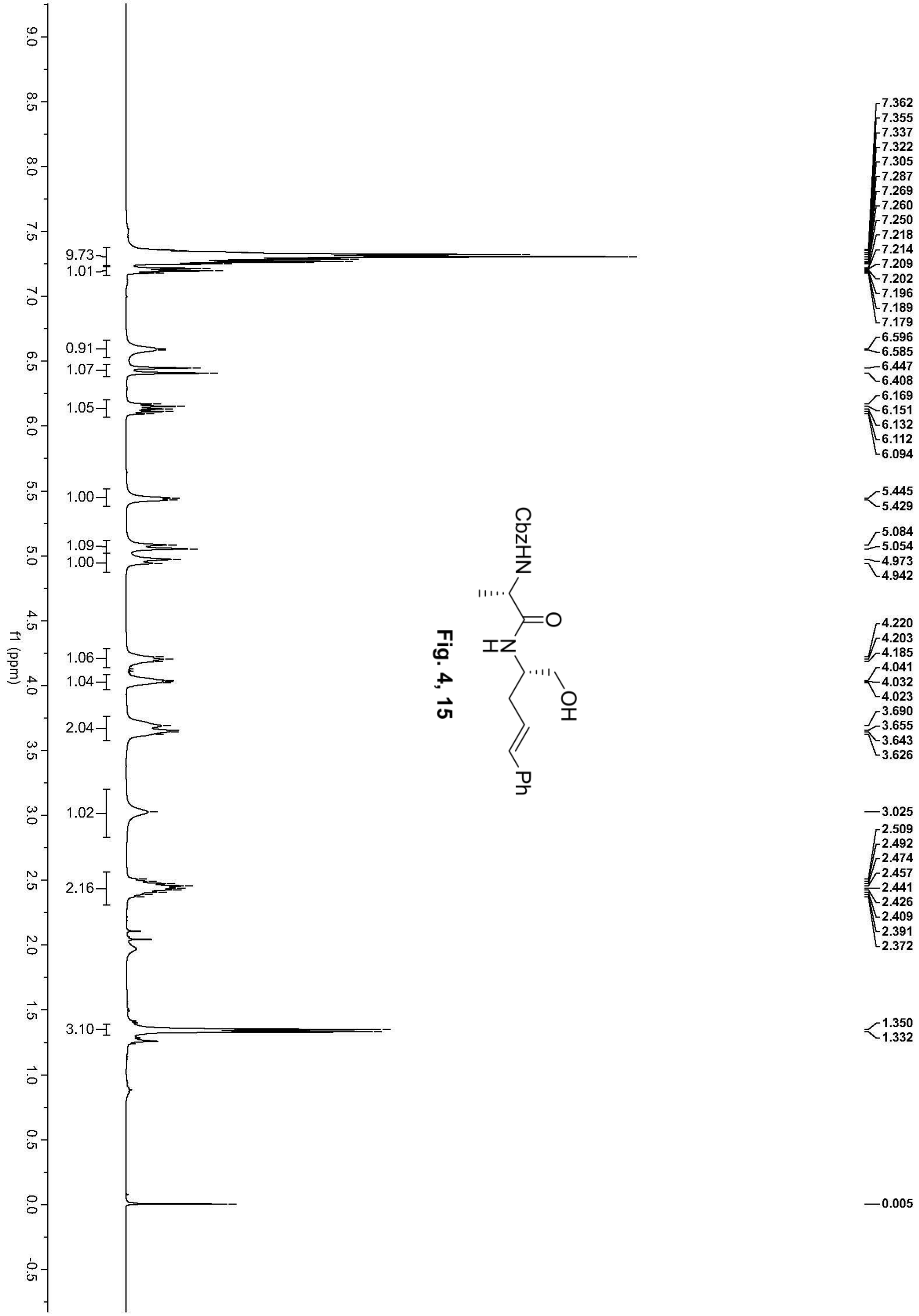

9.0
8.5
8.0
7.5
7.0
6.5
6.0
5.5
5.0
4.5
4.0
3.5
3.0
2.5
2.0
1.5
1.0
0.5
0.0
-0.5
f1 (ppm)
9.73
1.01
0.91
1.07
1.05
1.00
1.09
1.00
1.06
1.04
2.04
1.02
2.16
3.10
7.362
7.355
7.337
7.322
7.305
7.287
7.269
7.260
7.250
7.218
7.214
7.209
7.202
7.196
7.189
7.179
6.596
6.585
6.447
6.408
6.169
6.151
6.132
6.112
6.094
5.445
5.429
5.084
5.054
4.973
4.942
4.220
4.203
4.185
4.041
4.032
4.023
3.690
3.655
3.643
3.626
3.025
2.509
2.492
2.474
2.457
2.441
2.426
2.409
2.391
2.372
1.350
1.332
0.005
CbzHN
O
N
H
OH
Ph

Fig. 4, 15

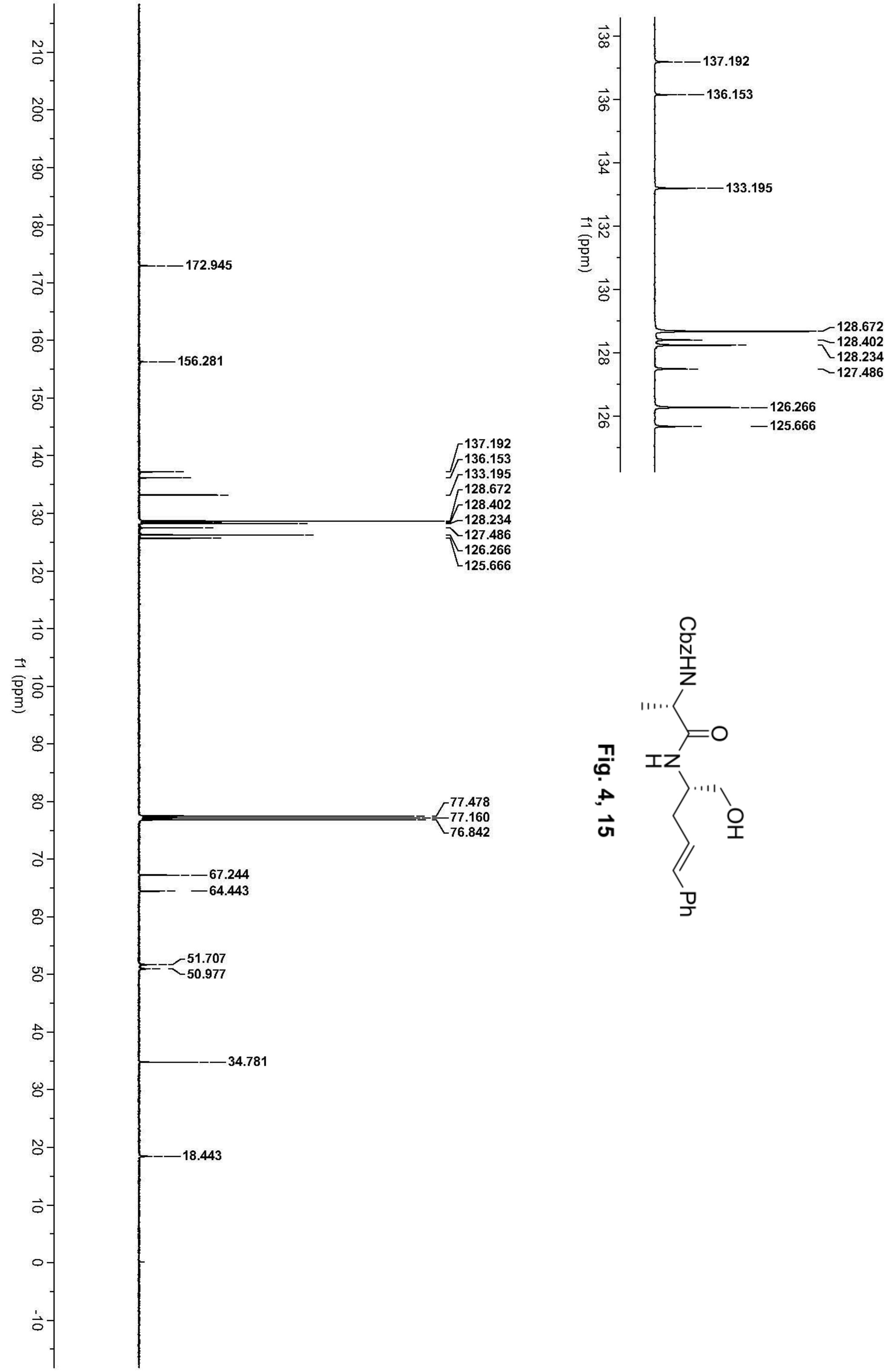

172.945
156.281
137.192
136.153
133.195
128.672
128.402
128.234
127.486
126.266
125.666
77.478
77.160
76.842
67.244
64.443
51.707
50.977
34.781
18.443
f1 (ppm)
CbzHN
O
N
H
OH
Ph

Fig. 4, 15

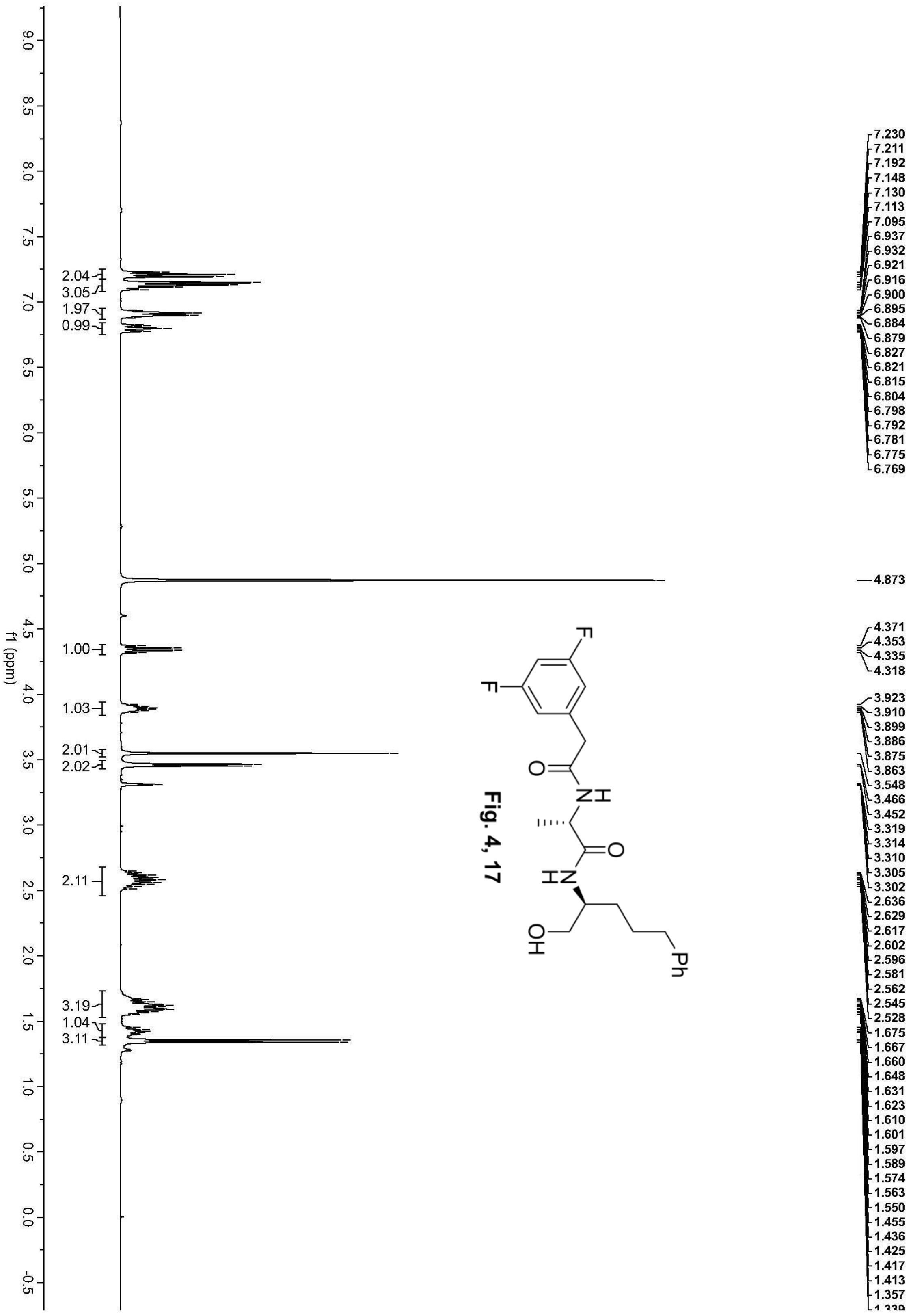

Fig. 4, 17

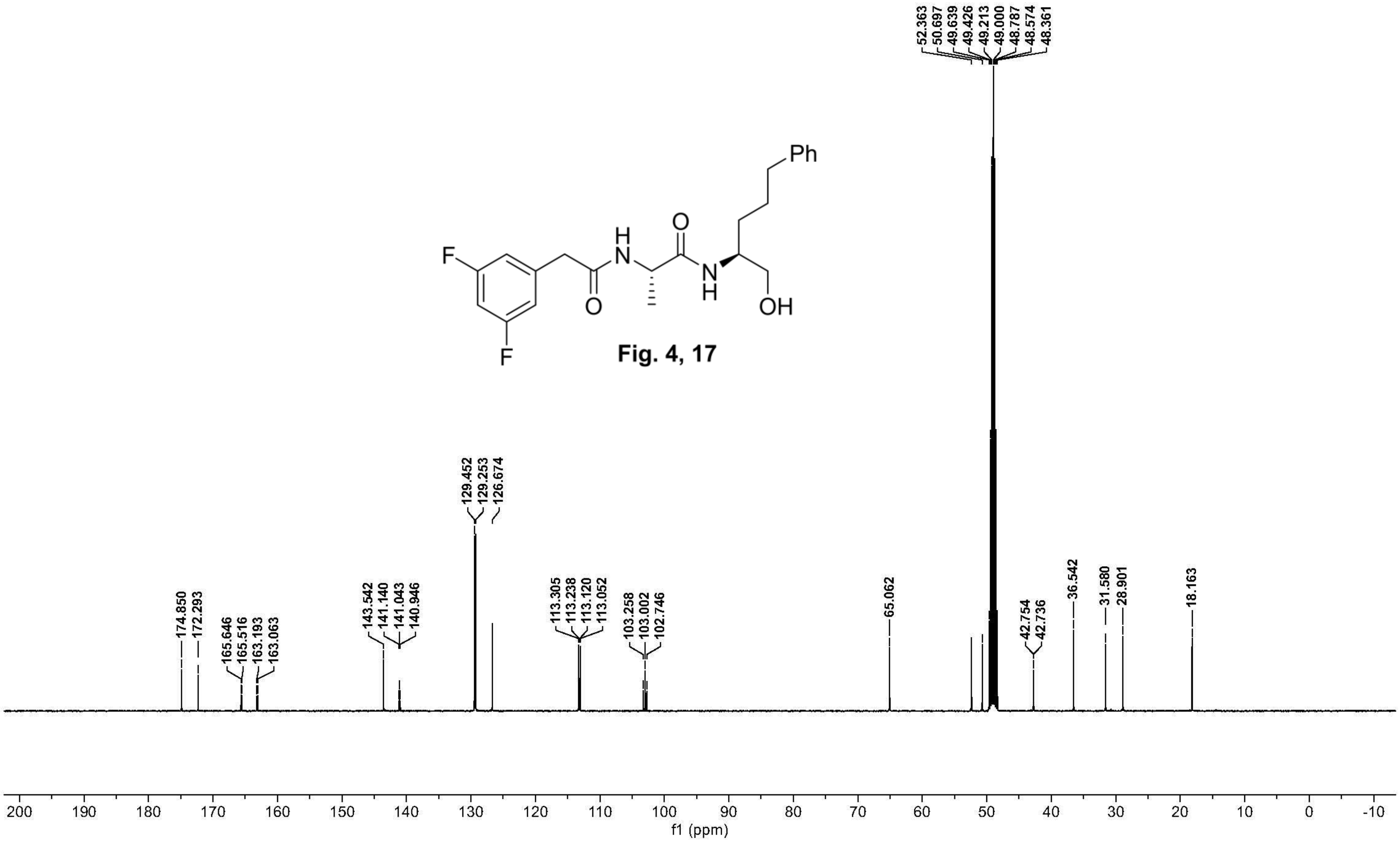
174.850
172.293
165.646
165.516
163.193
163.063
143.542
141.140
141.043
140.946
129.452
129.253
126.674
113.305
113.238
113.120
113.052
103.258
103.002
102.746
65.062
52.363
50.697
49.639
49.426
49.213
49.000
48.787
48.574
48.361
42.754
42.736
36.542
31.580
28.901
18.163
200 190 180 170 160 150 140 130 120 110 100 90 80 70 60 50 40 30 20 10 0 -10
f1 (ppm)
F
F
H
N
O
O
N
H
OH
Ph

Fig. 4, 17

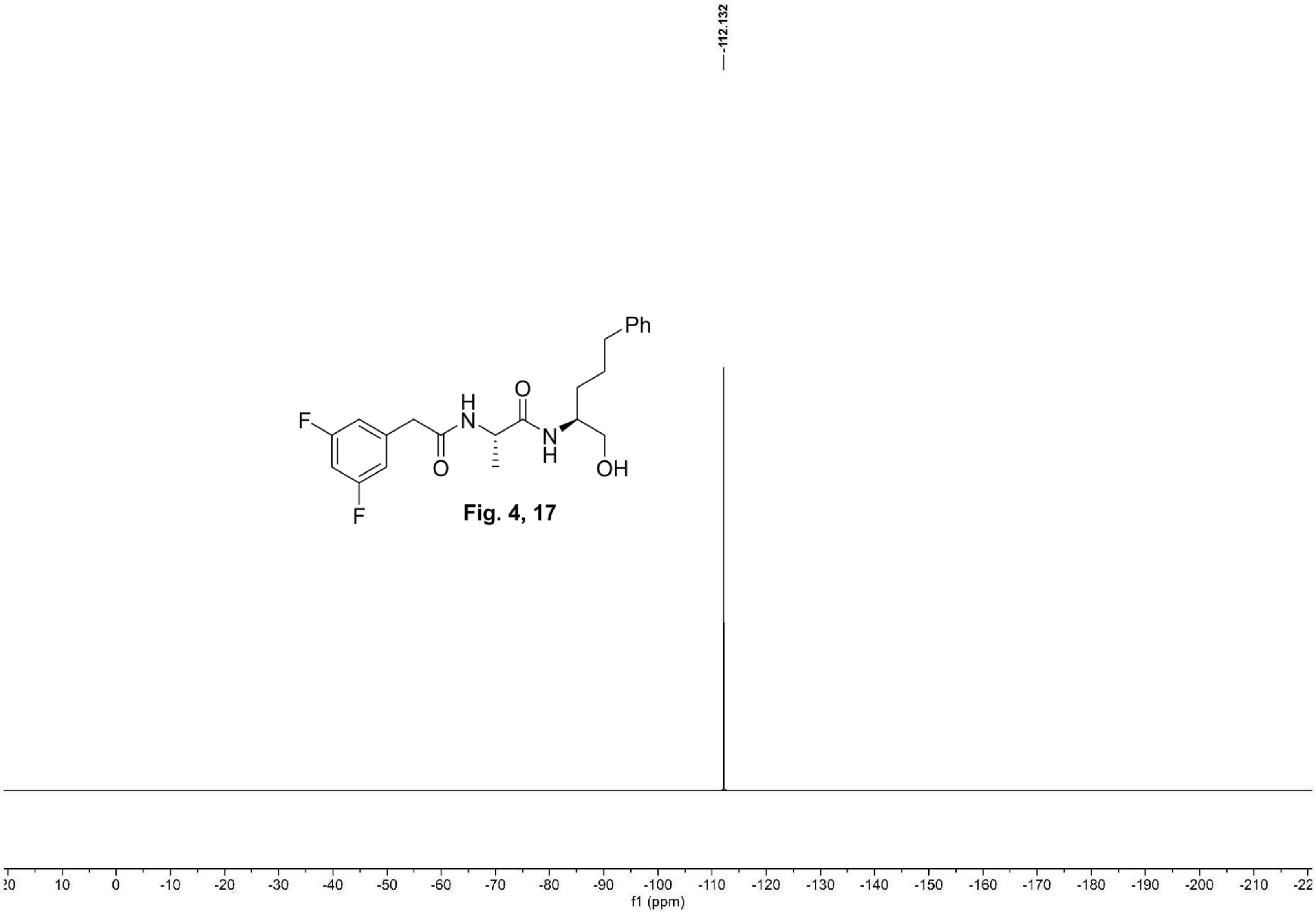
-112.132
F
F
H
N
O
O
N
H
OH
Ph
20 10 0 -10 -20 -30 -40 -50 -60 -70 -80 -90 -100 -110 -120 -130 -140 -150 -160 -170 -180 -190 -200 -210 -22
f1 (ppm)

Fig. 4, 17

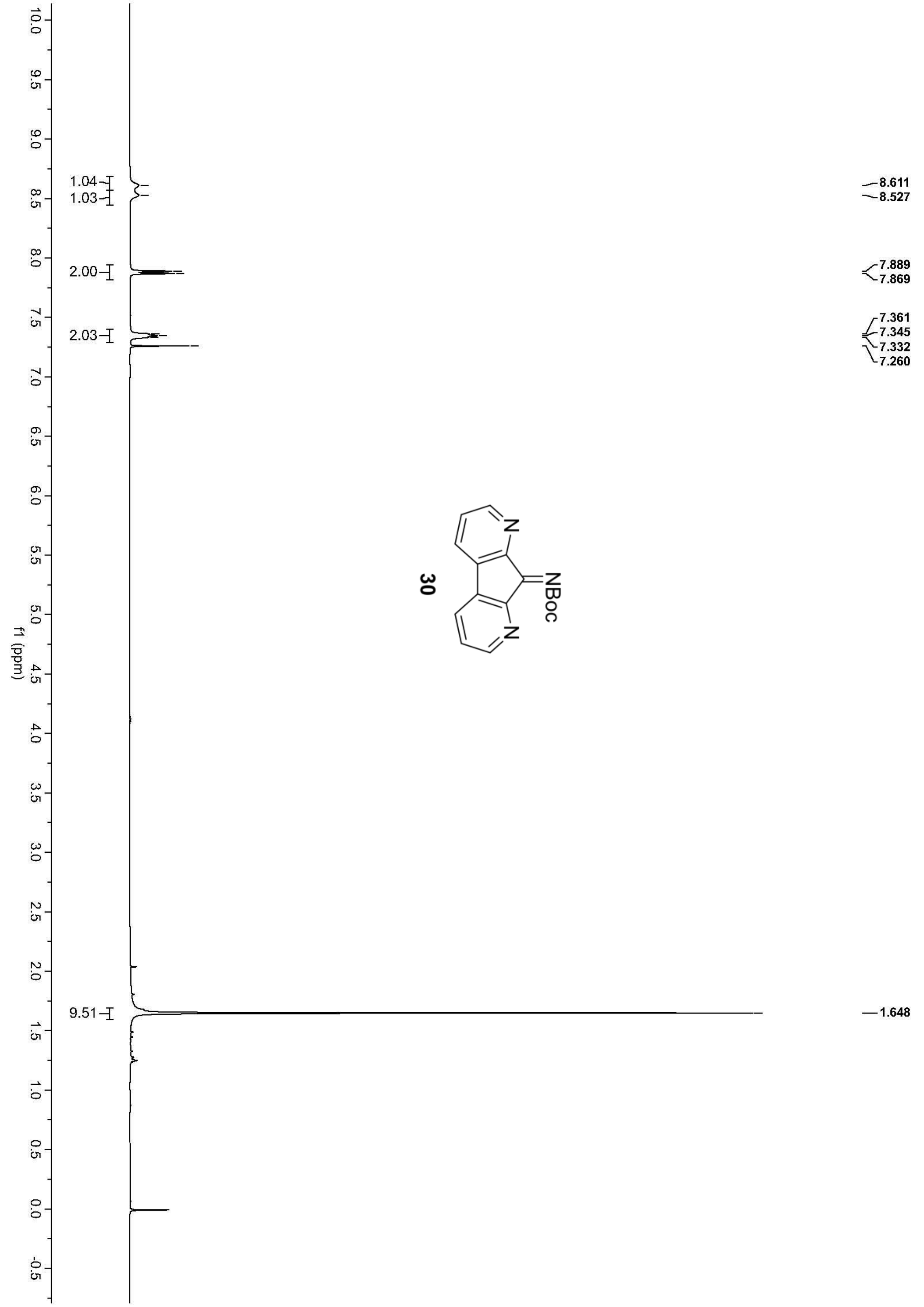

8.611
8.527
7.889
7.869
7.361
7.345
7.332
7.260
1.648
1.04
1.03
2.00
2.03
9.51
f1 (ppm)
N
N
NBoc
30

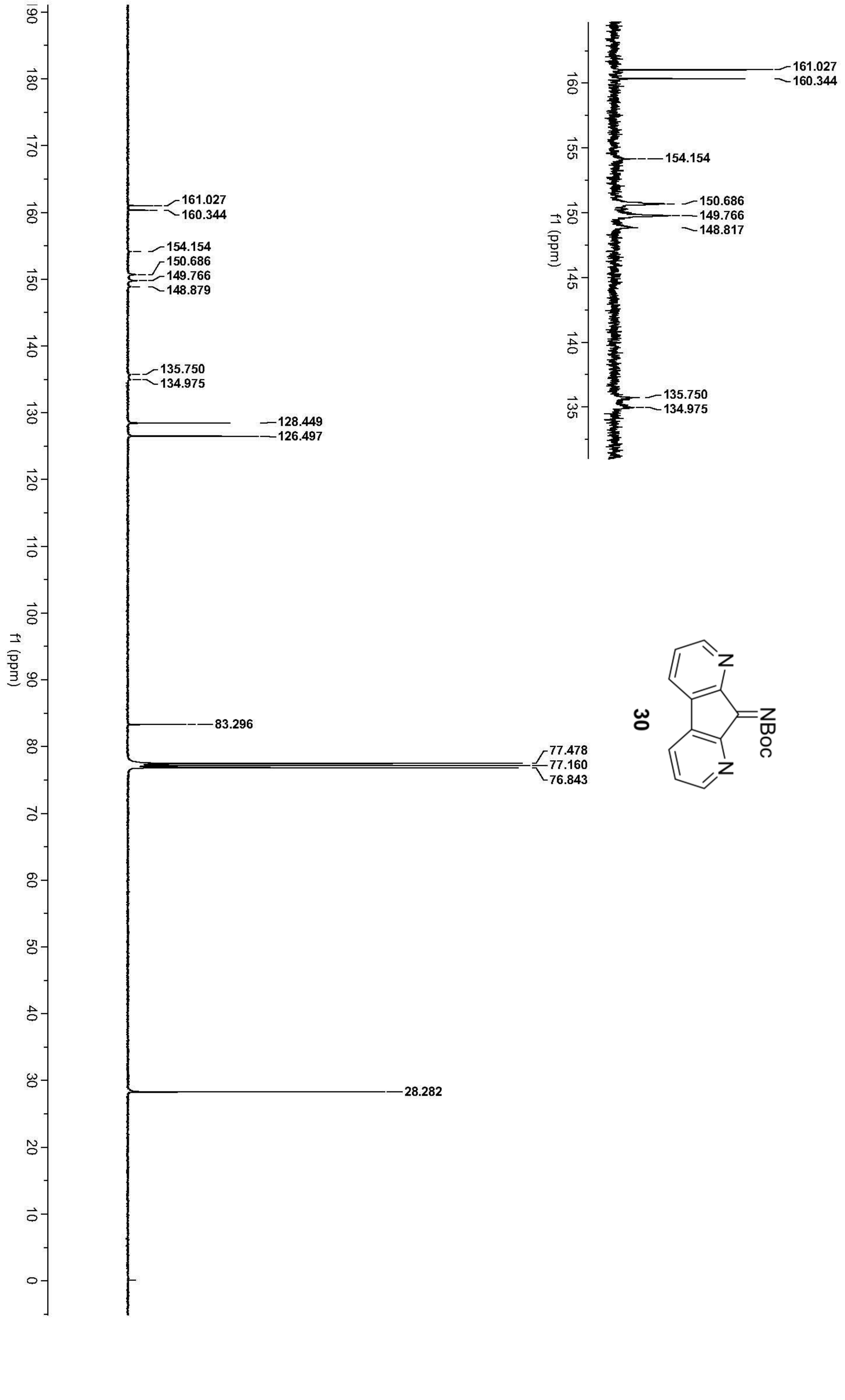
161.027
160.344
154.154
150.686
149.766
148.879
135.750
134.975
128.449
126.497
83.296
77.478
77.160
76.843
28.282
f1 (ppm)
148.817
NBoc
N
N
30

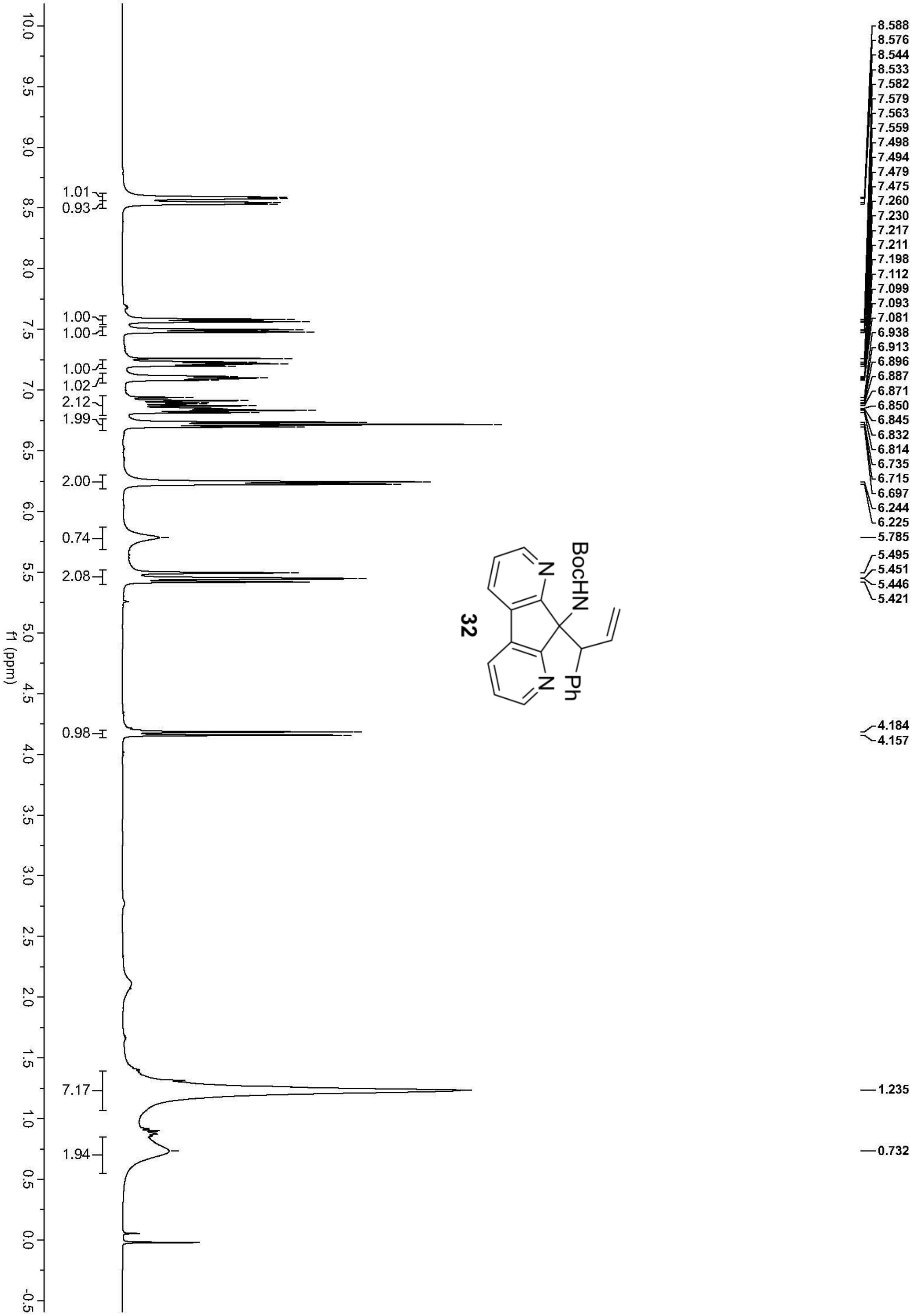
BocHN
N
N
Ph
32
f1 (ppm)
8.588
8.576
8.544
8.533
7.582
7.579
7.563
7.559
7.498
7.494
7.479
7.475
7.260
7.230
7.217
7.211
7.198
7.112
7.099
7.093
7.081
6.938
6.913
6.896
6.887
6.871
6.850
6.845
6.832
6.814
6.735
6.715
6.697
6.244
6.225
5.785
5.495
5.451
5.446
5.421
4.184
4.157
1.235
0.732
1.01
0.93
1.00
1.00
1.00
1.02
2.12
1.99
2.00
0.74
2.08
0.98
7.17
1.94

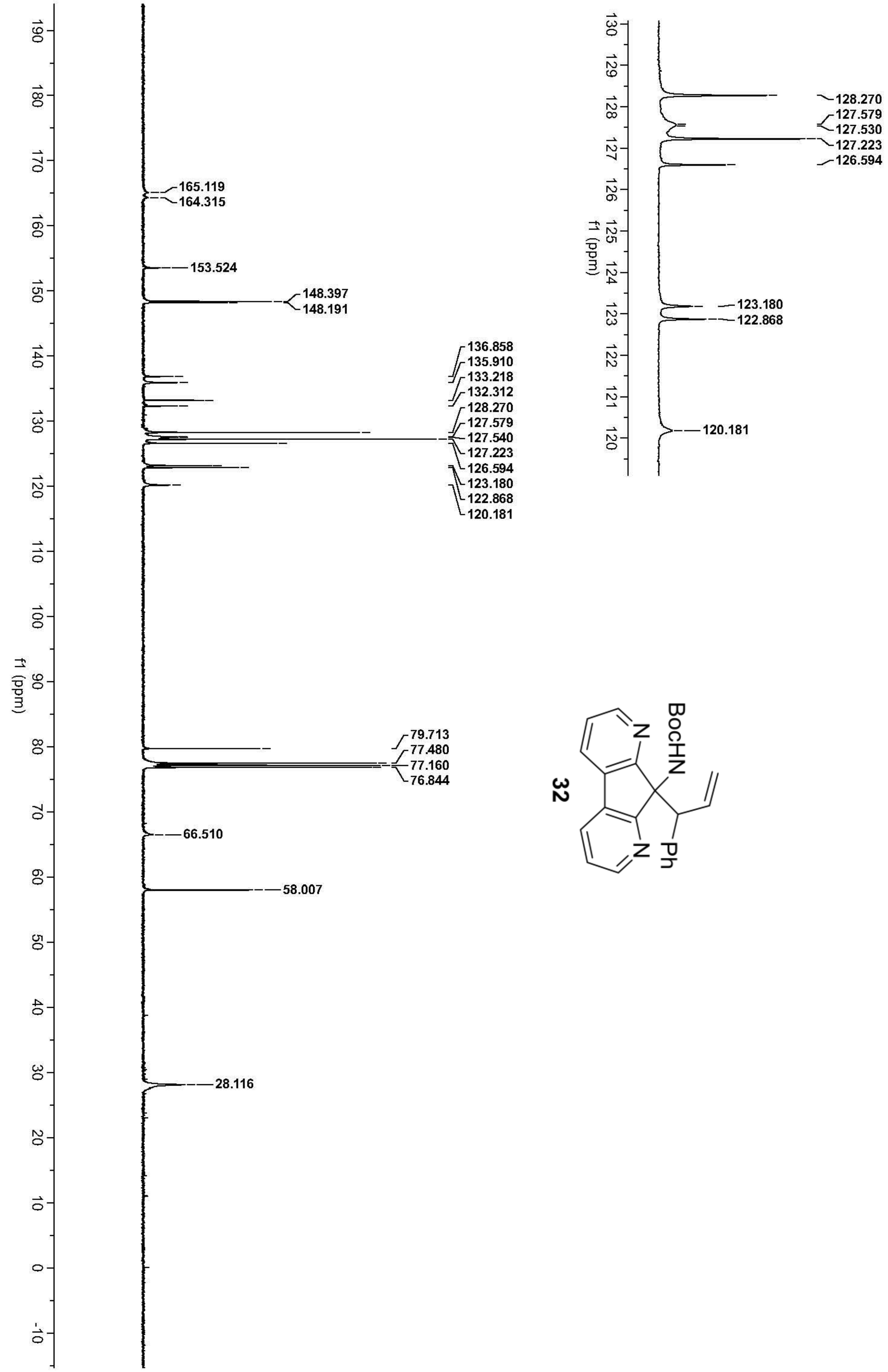
165.119
164.315
153.524
148.397
148.191
136.858
135.910
133.218
132.312
128.270
127.579
127.540
127.223
126.594
123.180
122.868
120.181
79.713
77.480
77.160
76.844
66.510
58.007
28.116
f1 (ppm)
128.270
127.579
127.530
127.223
126.594
123.180
122.868
120.181
f1 (ppm)
BocHN
N
N
Ph
32

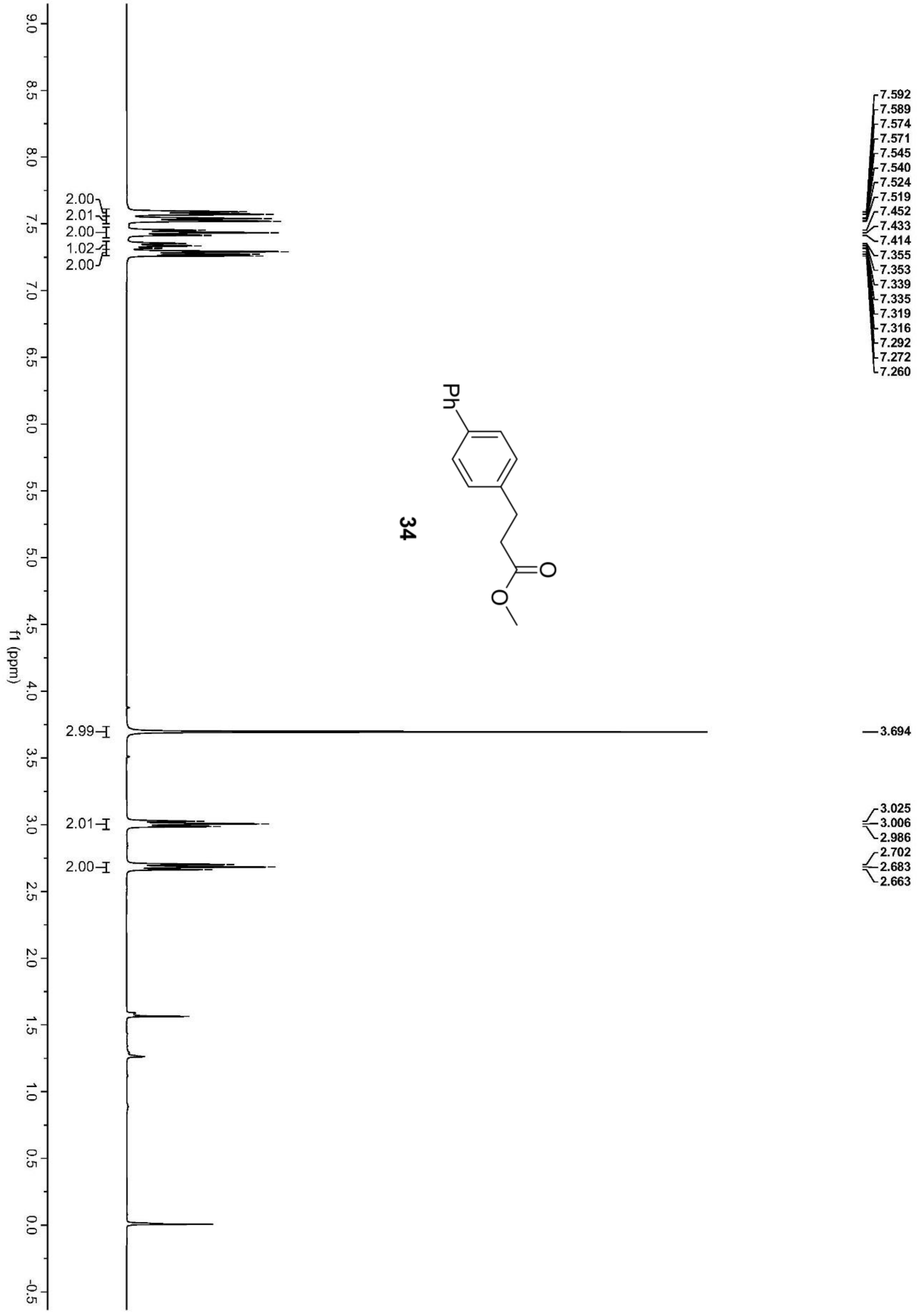

34
Ph
O
f1 (ppm)
9.0
8.5
8.0
7.5
7.0
6.5
6.0
5.5
5.0
4.5
4.0
3.5
3.0
2.5
2.0
1.5
1.0
0.5
0.0
-0.5
2.00
2.01
2.00
1.02
2.00
2.99
2.01
2.00
7.592
7.589
7.574
7.571
7.545
7.540
7.524
7.519
7.452
7.433
7.414
7.355
7.353
7.339
7.335
7.319
7.316
7.292
7.272
7.260
3.694
3.025
3.006
2.986
2.702
2.683
2.663

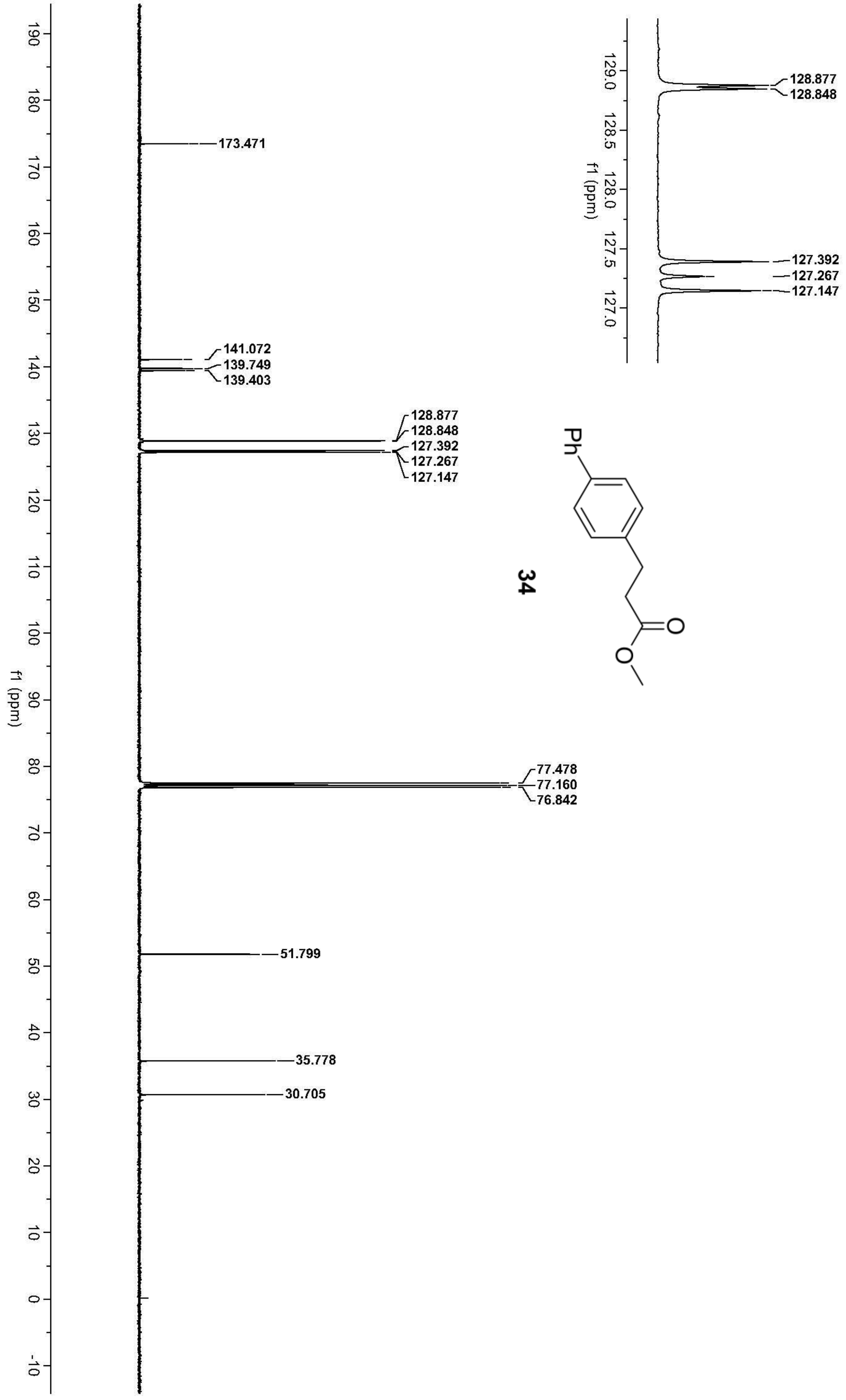
173.471
141.072
139.749
139.403
128.877
128.848
127.392
127.267
127.147
77.478
77.160
76.842
51.799
35.778
30.705
f1 (ppm)
Ph
O
O
34

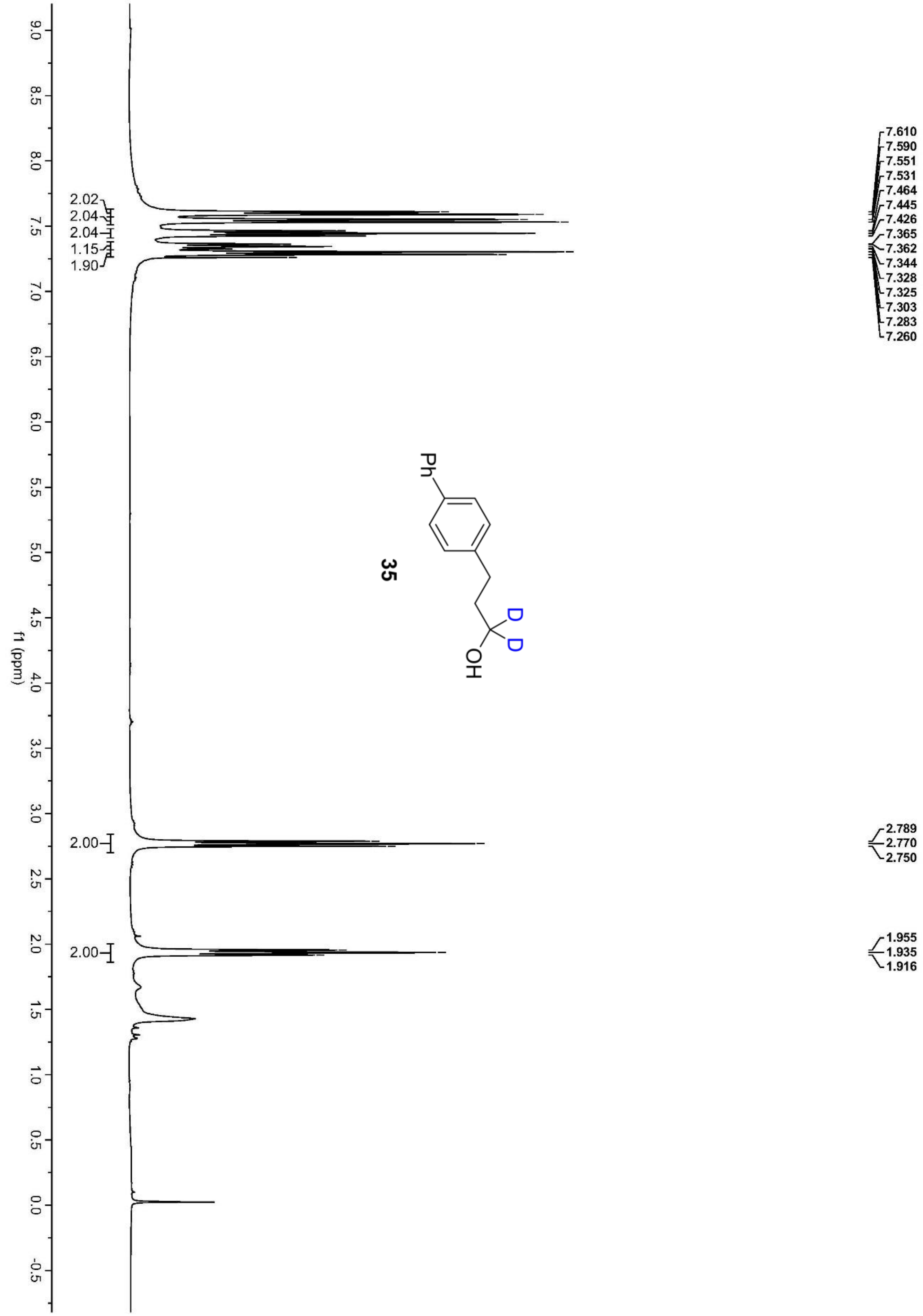
35
Ph
D D
OH
f1 (ppm)
7.610
7.590
7.551
7.531
7.464
7.445
7.426
7.365
7.362
7.344
7.328
7.325
7.303
7.283
7.260
2.789
2.770
2.750
1.955
1.935
1.916
2.02
2.04
2.04
1.15
1.90
2.00
2.00

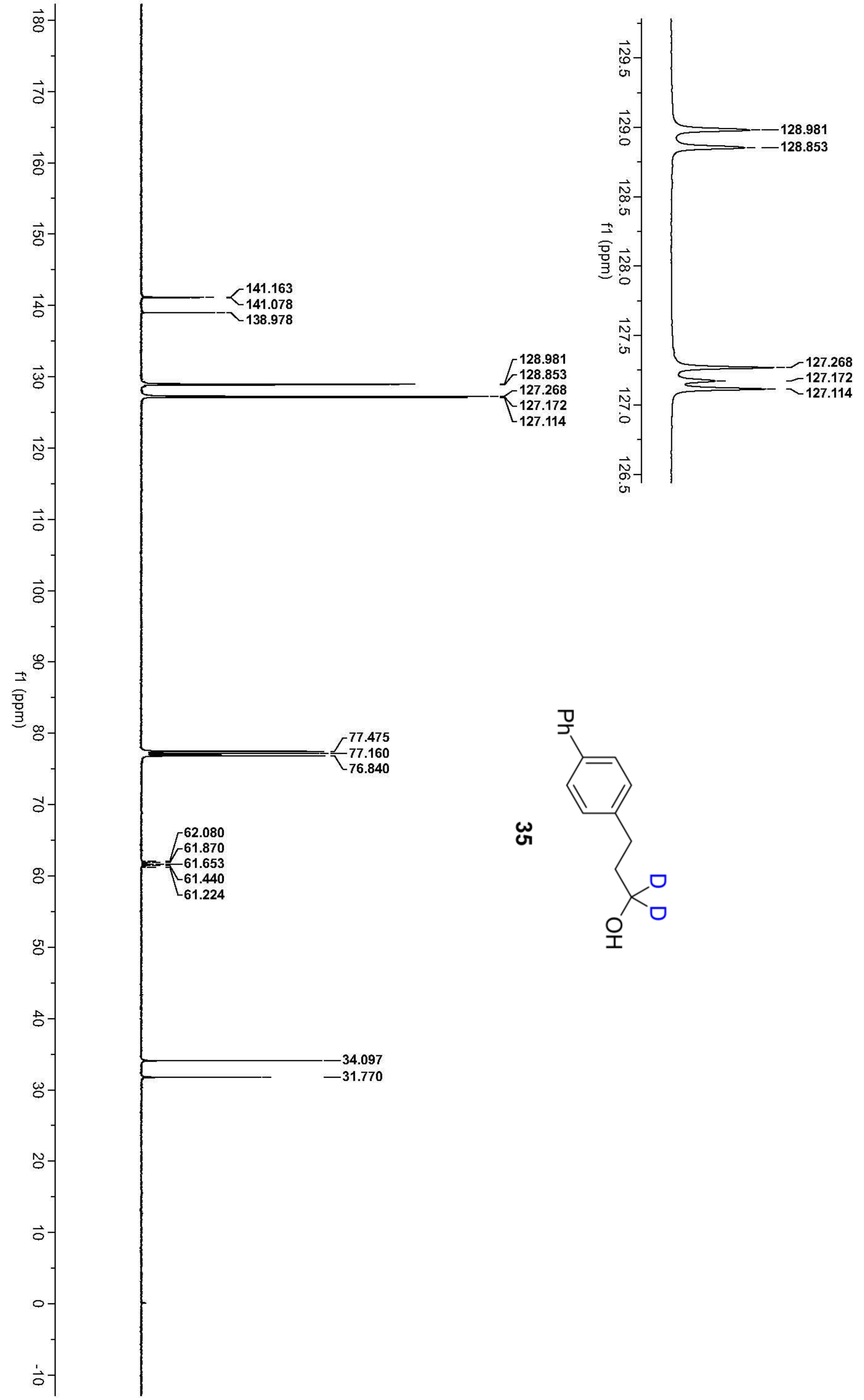

129.5
129.0
128.5
128.0
127.5
127.0
126.5
f1 (ppm)
128.981
128.853
127.268
127.172
127.114
141.163
141.078
138.978
128.981
128.853
127.268
127.172
127.114
77.475
77.160
76.840
62.080
61.870
61.653
61.440
61.224
34.097
31.770
180
170
160
150
140
130
120
110
100
90
80
70
60
50
40
30
20
10
0
-10
f1 (ppm)
Ph
D D
OH

35

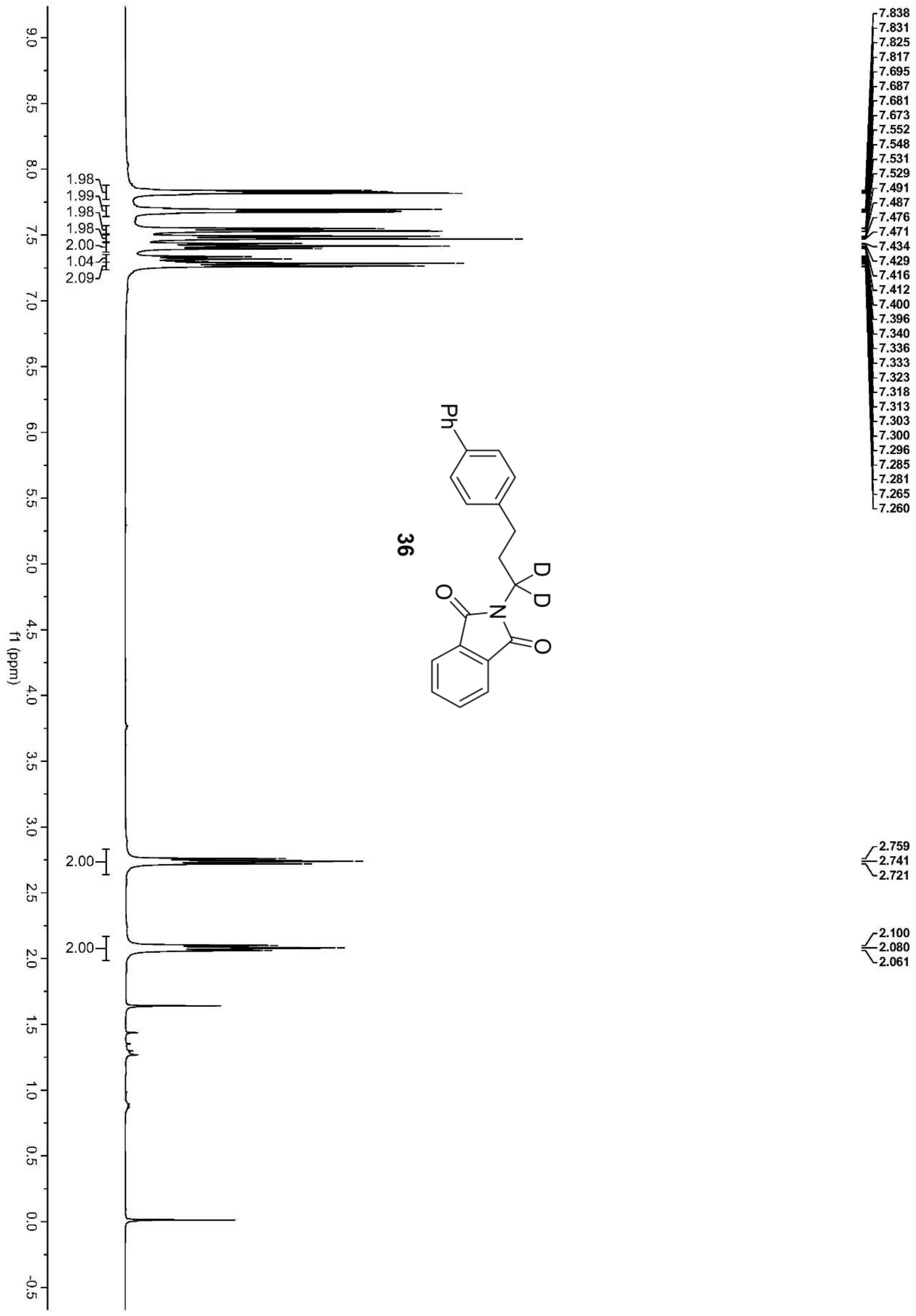

36
Ph
D D
O
N
O
f1 (ppm)
9.0 8.5 8.0 7.5 7.0 6.5 6.0 5.5 5.0 4.5 4.0 3.5 3.0 2.5 2.0 1.5 1.0 0.5 0.0 -0.5
1.98 1.99 1.98 1.98 2.00 1.04 2.09 2.00 2.00
7.838 7.831 7.825 7.817 7.695 7.687 7.681 7.673 7.552 7.548 7.531 7.529 7.491 7.487 7.476 7.471 7.434 7.429 7.416 7.412 7.400 7.396 7.340 7.336 7.333 7.323 7.318 7.313 7.303 7.300 7.296 7.285 7.281 7.265 7.260
2.759 2.741 2.721
2.100 2.080 2.061

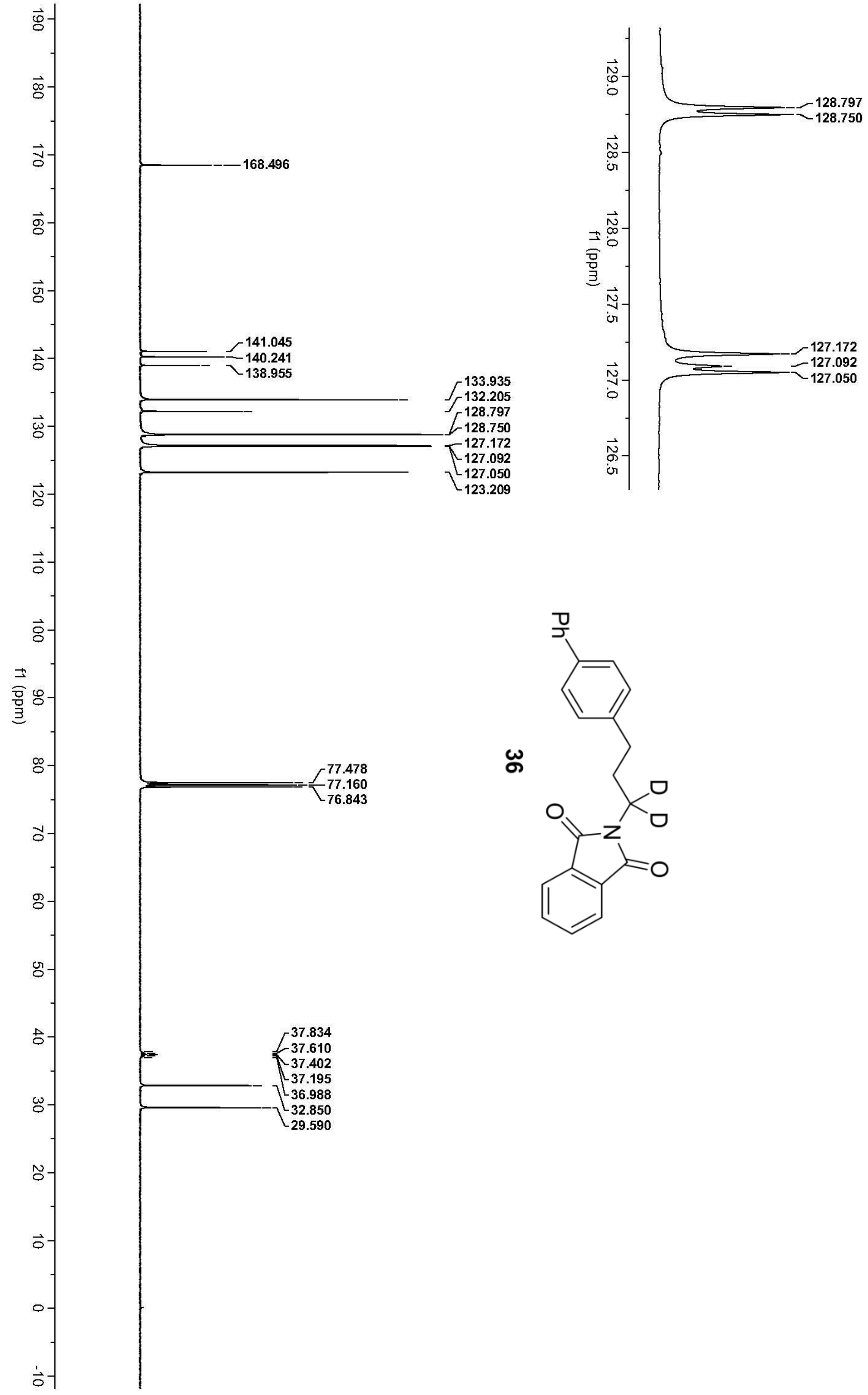

168.496
141.045
140.241
138.955
133.935
132.205
128.797
128.750
127.172
127.092
127.050
123.209
77.478
77.160
76.843
37.834
37.610
37.402
37.195
36.988
32.850
29.590
f1 (ppm)
129.0
128.5
128.0
127.5
127.0
126.5
Ph
D D
N
O
O
36

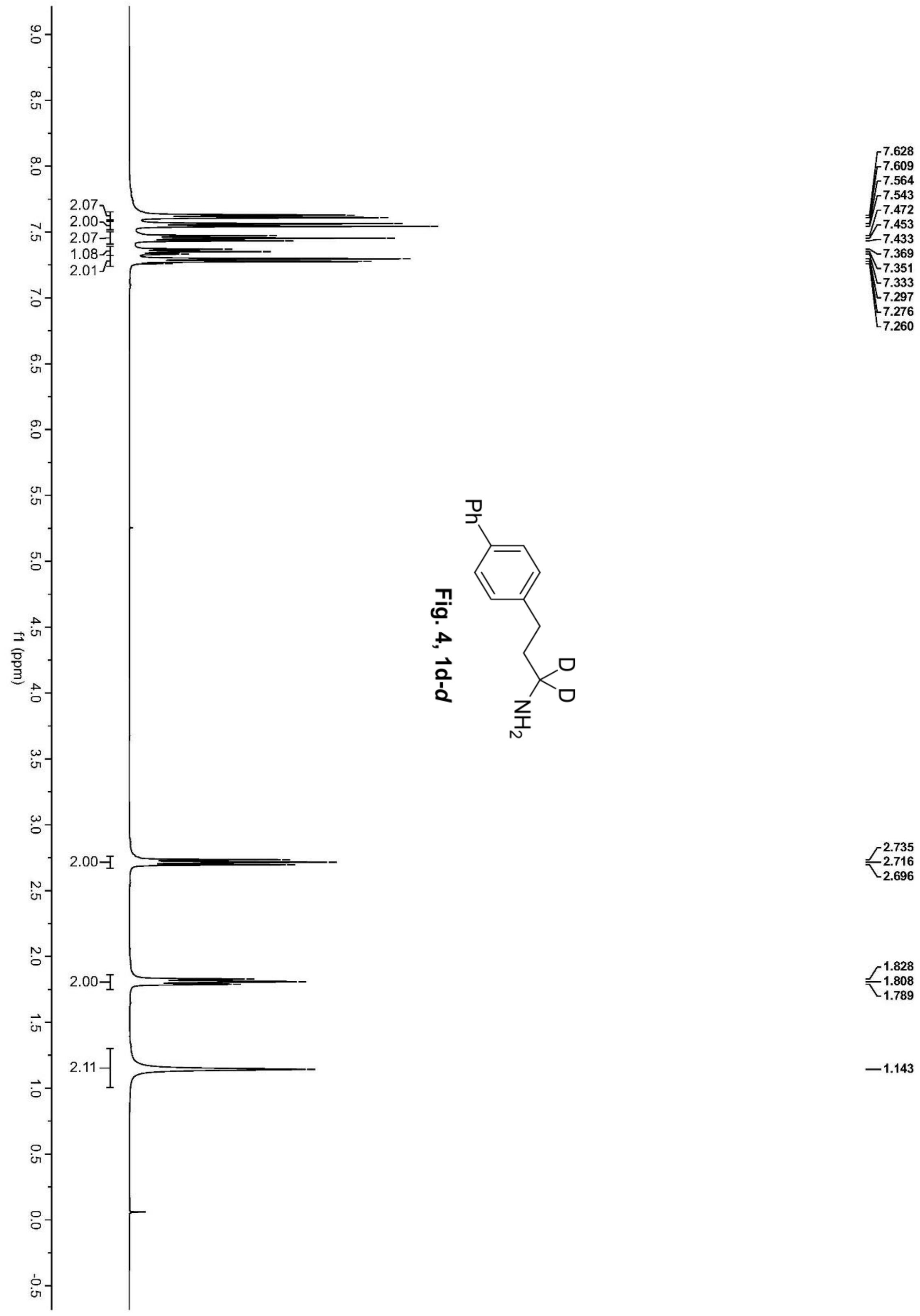

f1 (ppm)
Ph
D D
NH2
7.628
7.609
7.564
7.543
7.472
7.453
7.433
7.369
7.351
7.333
7.297
7.276
7.260
2.735
2.716
2.696
1.828
1.808
1.789
1.143
2.07
2.00
2.07
1.08
2.01
2.00
2.00
2.11

Fig. 4, 1d-*d*

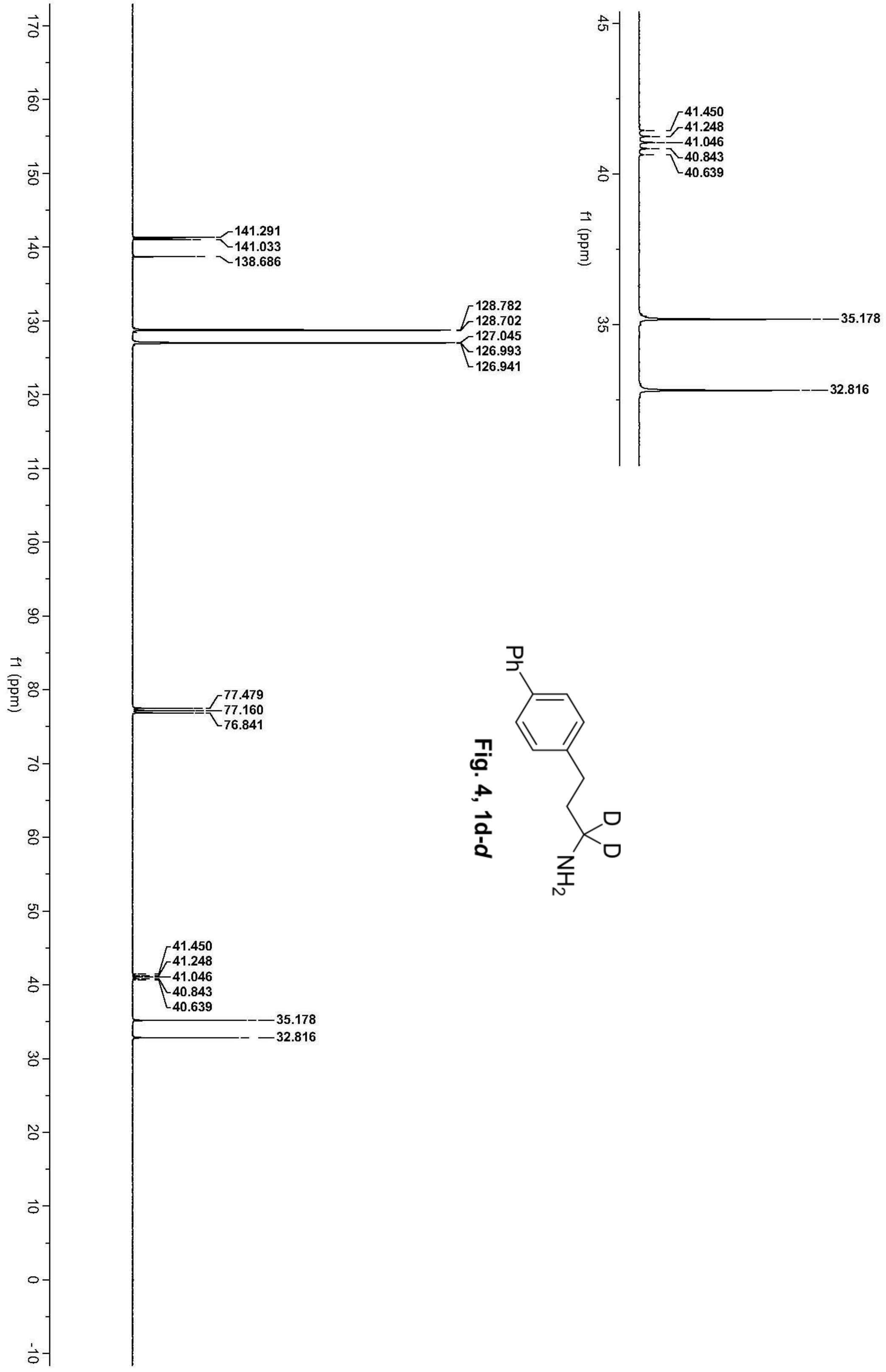

f1 (ppm)
170
160
150
140
130
120
110
100
90
80
70
60
50
40
30
20
10
0
-10
141.291
141.033
138.686
128.782
128.702
127.045
126.993
126.941
77.479
77.160
76.841
41.450
41.248
41.046
40.843
40.639
35.178
32.816
45
40
35
Ph
D
D
NH_2

Fig. 4, 1d-*d*

3.2 Chromatograms for the Determination of Enantiomeric Excesses and Diastereomeric Excesses

Fig 3, Compound 3a

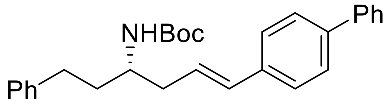

HPLC Conditions

Column: Chiralpak AD-H, Daicel Chemical Industries, Ltd.

Eluent: Hexanes/Isopropanol (95:5)

Flow rate: 1.0 mL/min

Detection: UV 254 nm

Racemic

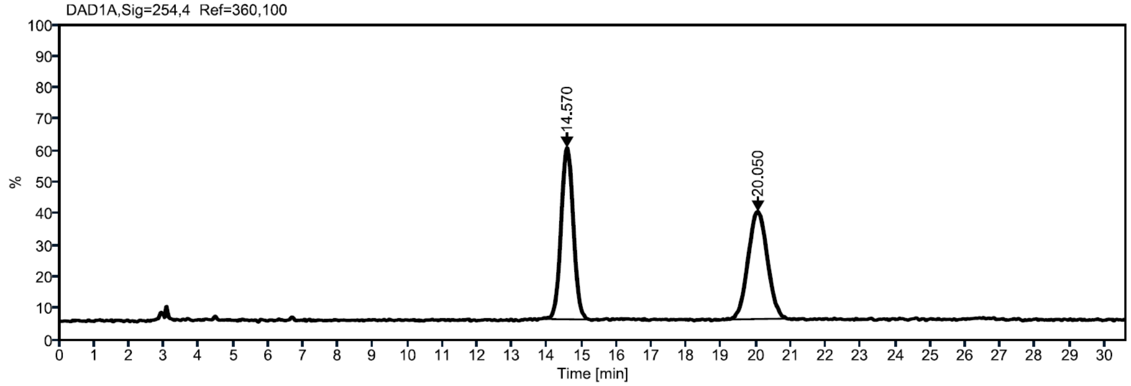

Signal: DAD1A,Sig=254,4 Ref=360,100

RT [min]	Type	Width [min]	Area	Height	Area%
14.570	MM m	0.37	191.21	7.82	50.27
20.050	MM m	0.47	189.15	4.90	49.73
		Sum	380.36		

Chiral

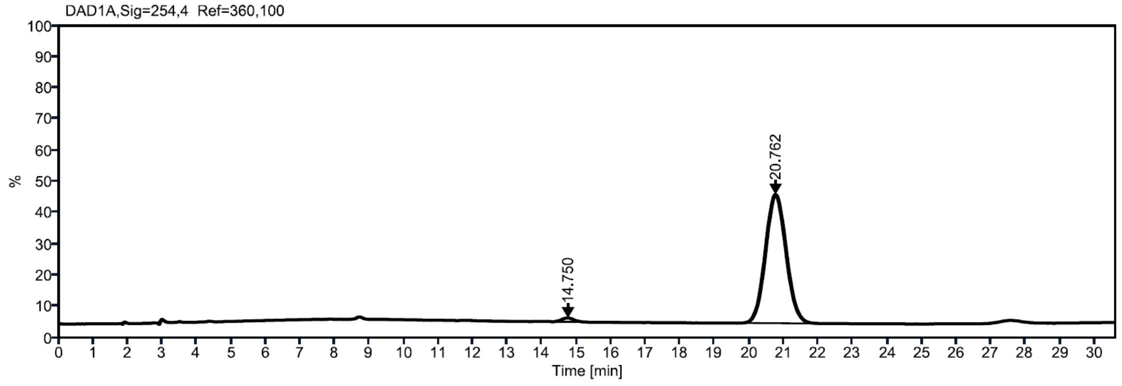

Signal: DAD1A,Sig=254,4 Ref=360,100

RT [min]	Type	Width [min]	Area	Height	Area%
14.750	MM m	0.28	10.13	0.46	1.50
20.762	MM m	0.64	666.65	15.99	98.50
		Sum	676.78		

Fig. 3, Compound 3b

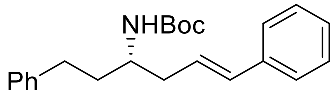

HPLC Conditions

Column: Chiralpak AD-H, Daicel Chemical Industries, Ltd.

Eluent: Hexanes/Isopropanol (95:5)

Flow rate: 1.0 mL/min

Detection: UV 254 nm

Racemic

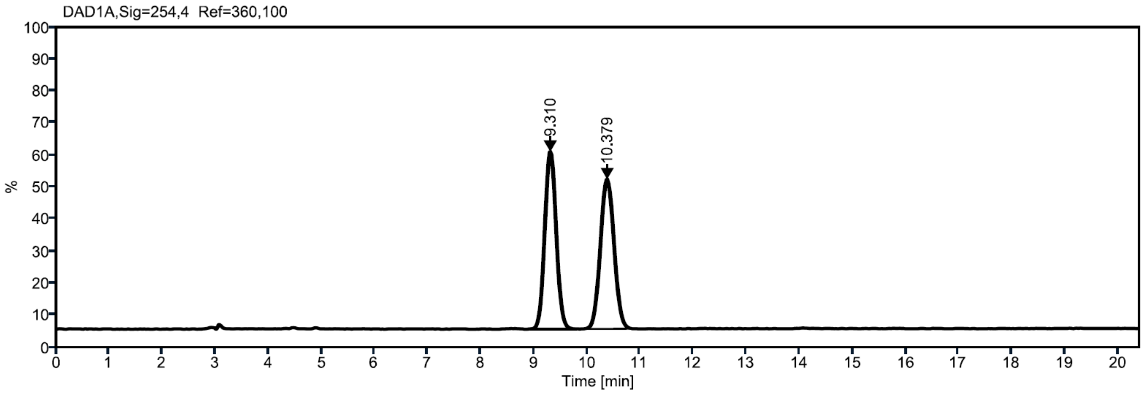

Signal: DAD1A,Sig=254,4 Ref=360,100

RT [min]	Type	Width [min]	Area	Height	Area%
9.310	MM m	0.23	310.21	20.93	49.53
10.379	MM m	0.28	316.13	17.65	50.47
		Sum	626.34		

Chiral

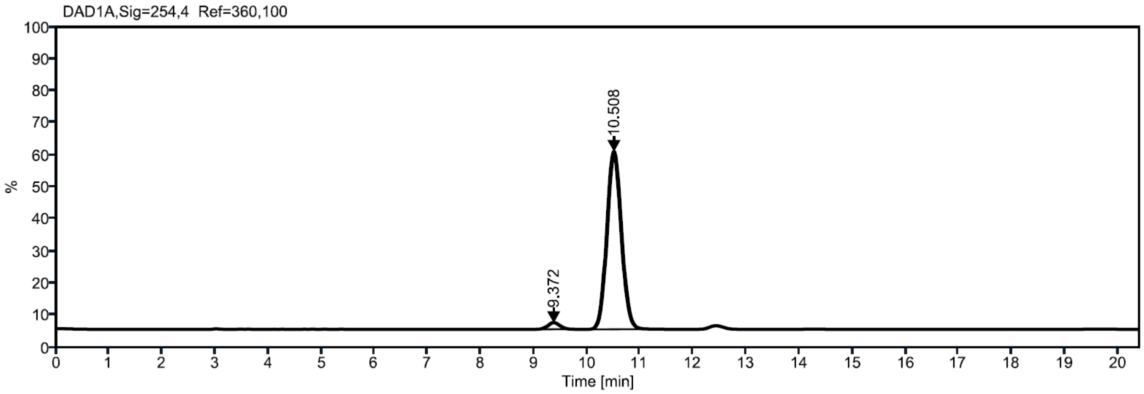

Signal: DAD1A,Sig=254,4 Ref=360,100

RT [min]	Type	Width [min]	Area	Height	Area%
9.372	MM m	0.24	175.62	11.34	2.93
10.508	MM m	0.30	5825.50	306.14	97.07
		Sum	6001.12		

Fig. 3, Compound 3c

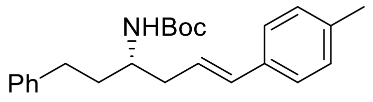

HPLC Conditions

Column: Chiralpak AD-H, Daicel Chemical Industries, Ltd.

Eluent: Hexanes/Isopropanol (95:5)

Flow rate: 1.0 mL/min

Detection: UV 254 nm

Racemic

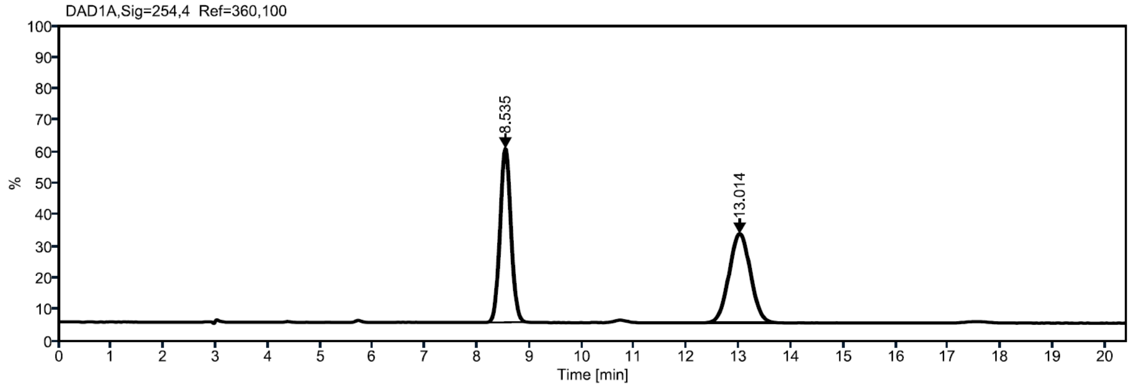

Signal: DAD1A,Sig=254,4 Ref=360,100

RT [min]	Type	Width [min]	Area	Height	Area%
8.535	MM m	0.23	423.12	28.85	50.15
13.014	MM m	0.44	420.52	14.79	49.85
		Sum	843.64		

Chiral

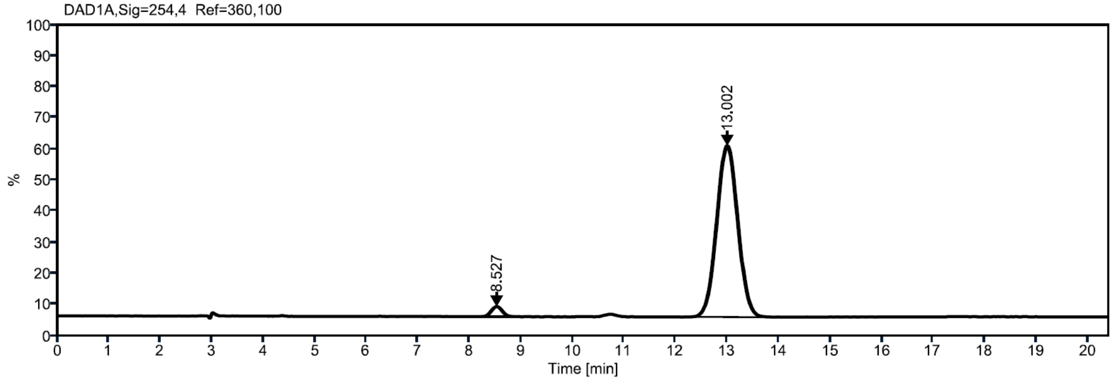

Signal: DAD1A,Sig=254,4 Ref=360,100

RT [min]	Type	Width [min]	Area	Height	Area%
8.527	MM m	0.22	16.71	1.14	2.98
13.002	MM m	0.44	544.61	19.14	97.02
		Sum	561.32		

Fig. 3, Compound 3d

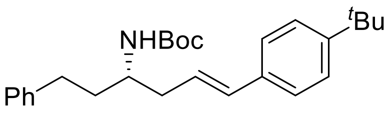

HPLC Conditions

Column: Chiralpak AD-H, Daicel Chemical Industries, Ltd.

Eluent: Hexanes/Isopropanol (95:5)

Flow rate: 1.0 mL/min

Detection: UV 254 nm

Racemic

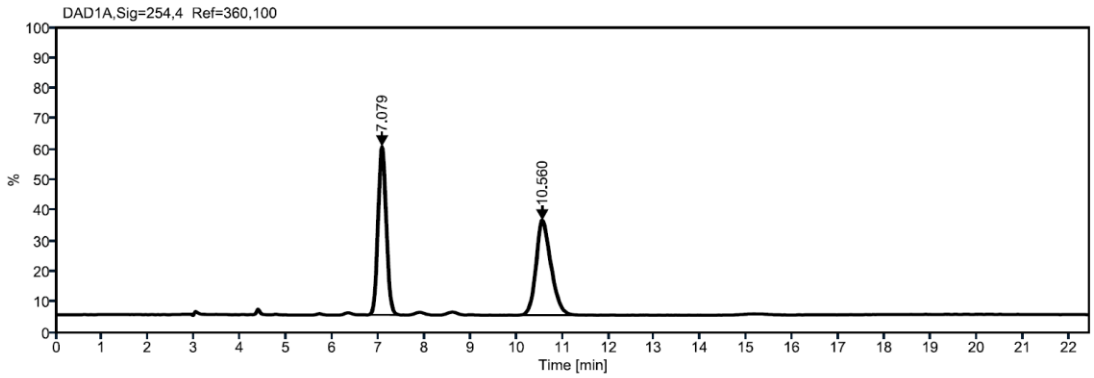

Signal: DAD1A,Sig=254,4 Ref=360,100

RT [min]	Type	Width [min]	Area	Height	Area%
7.079	MM m	0.19	224.07	18.05	49.43
10.560	MM m	0.33	229.25	10.18	50.57
		Sum	453.31		

Chiral

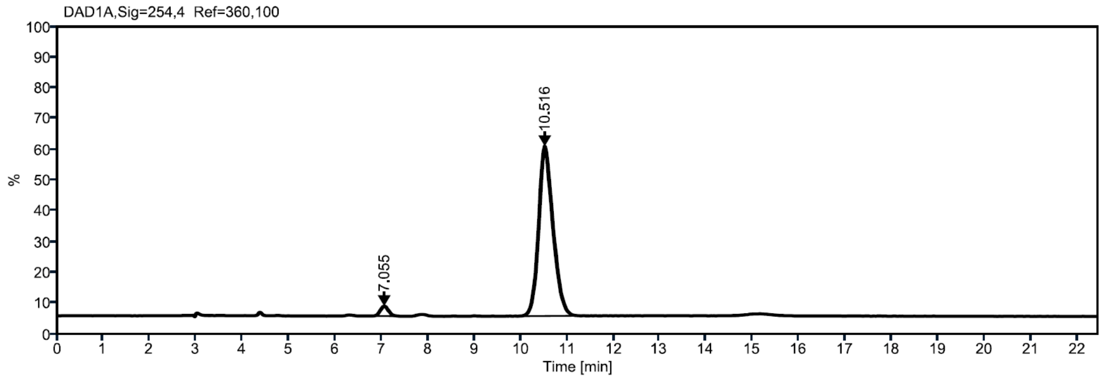

Signal: DAD1A,Sig=254,4 Ref=360,100

RT [min]	Type	Width [min]	Area	Height	Area%
7.055	MM m	0.19	14.66	1.18	3.28
10.516	MM m	0.32	432.72	20.06	96.72
		Sum	447.38		

Fig. 3, Compound 3e

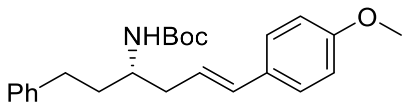

HPLC Conditions

Column: Chiralpak AD-H, Daicel Chemical Industries, Ltd.

Eluent: Hexanes/Isopropanol (95:5)

Flow rate: 1.0 mL/min

Detection: UV 254 nm

Racemic

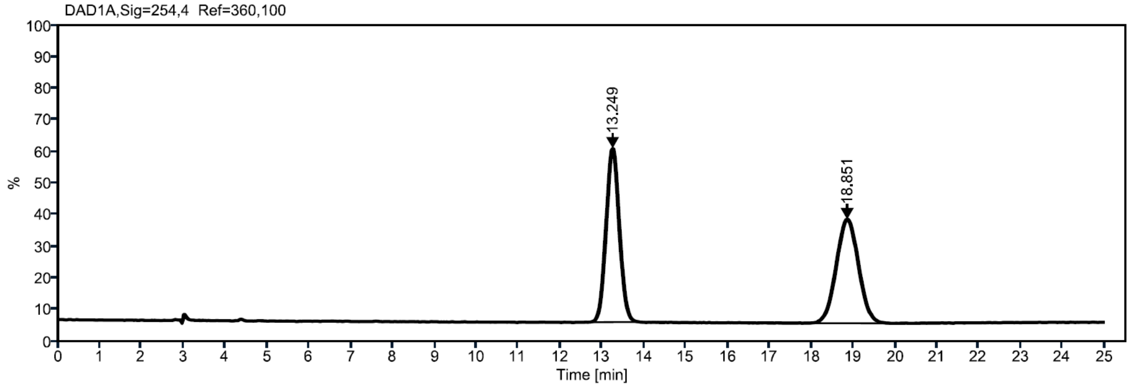

Signal: DAD1A,Sig=254,4 Ref=360,100

RT [min]	Type	Width [min]	Area	Height	Area%
13.249	MM m	0.34	238.17	10.79	49.99
18.851	MM m	0.55	238.24	6.45	50.01
		Sum	476.41		

Chiral

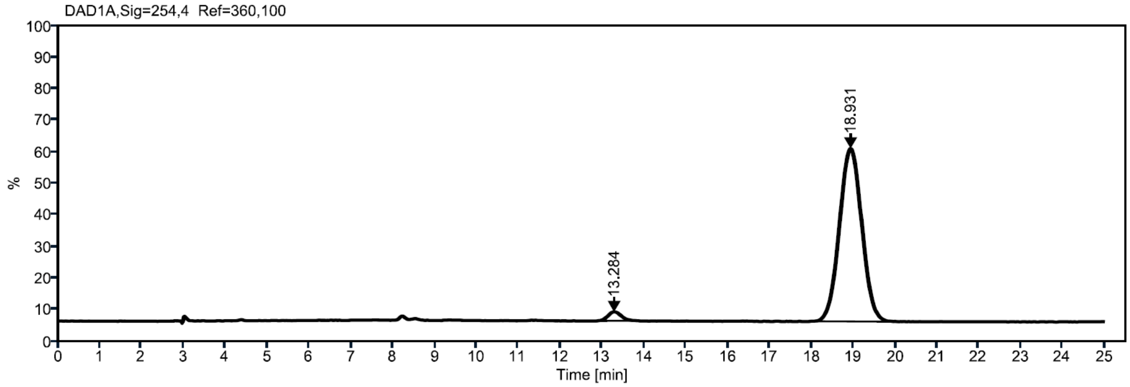

Signal: DAD1A,Sig=254,4 Ref=360,100

RT [min]	Type	Width [min]	Area	Height	Area%
13.284	MM m	0.28	15.20	0.69	2.98
18.931	MM m	0.58	494.23	13.29	97.02
		Sum	509.43		

Fig. 3, Compound 3f

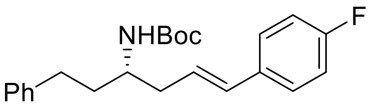

HPLC Conditions

Column: Chiralcel OD-3, Daicel Chemical Industries, Ltd.

Eluent: Hexanes/Isopropanol (95:5)

Flow rate: 1.0 mL/min

Detection: UV 254 nm

Racemic

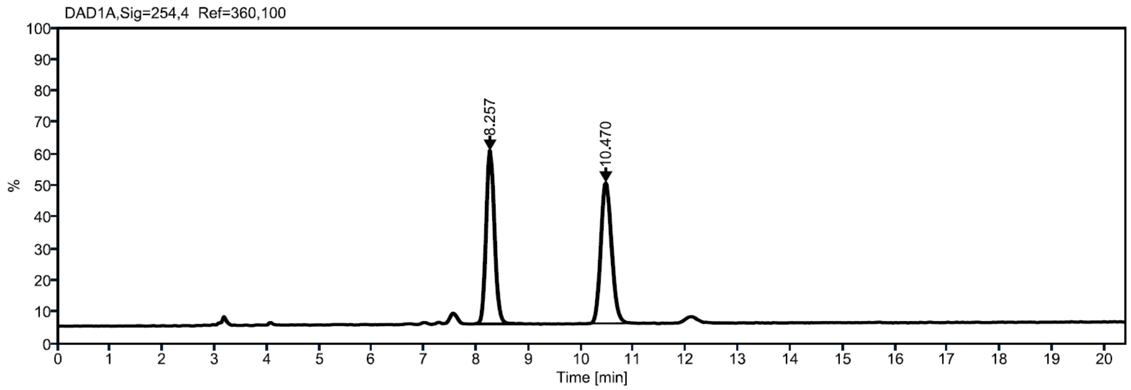

Signal: DAD1A,Sig=254,4 Ref=360,100

RT [min]	Type	Width [min]	Area	Height	Area%
8.257	MM m	0.17	350.52	31.75	48.58
10.470	MM m	0.22	371.04	25.87	51.42
		Sum	721.56		

Chiral

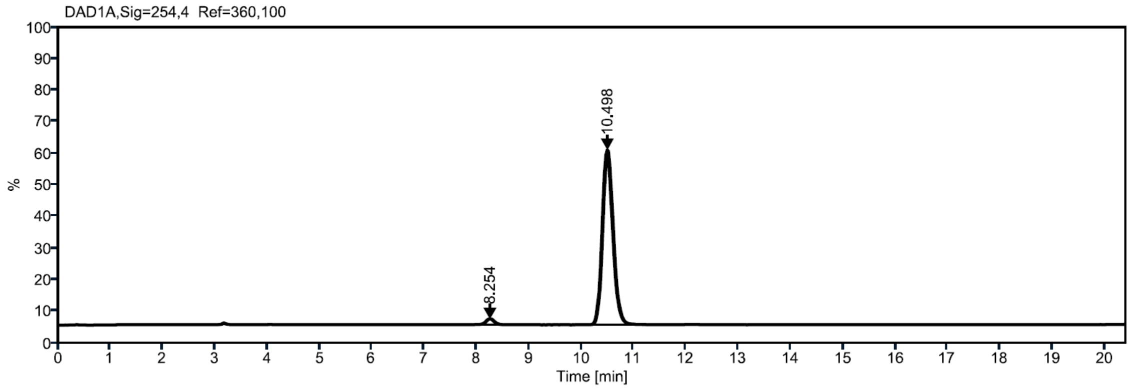

Signal: DAD1A,Sig=254,4 Ref=360,100

RT [min]	Type	Width [min]	Area	Height	Area%
8.254	MM m	0.17	85.74	7.53	2.65
10.498	MM m	0.22	3144.83	221.84	97.35
		Sum	3230.57		

Fig. 3, Compound 3g

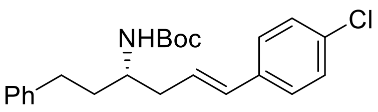

HPLC Conditions

Column: Chiralpak AD-H, Daicel Chemical Industries, Ltd.

Eluent: Hexanes/Isopropanol (95:5)

Flow rate: 1.0 mL/min

Detection: UV 254 nm

Racemic

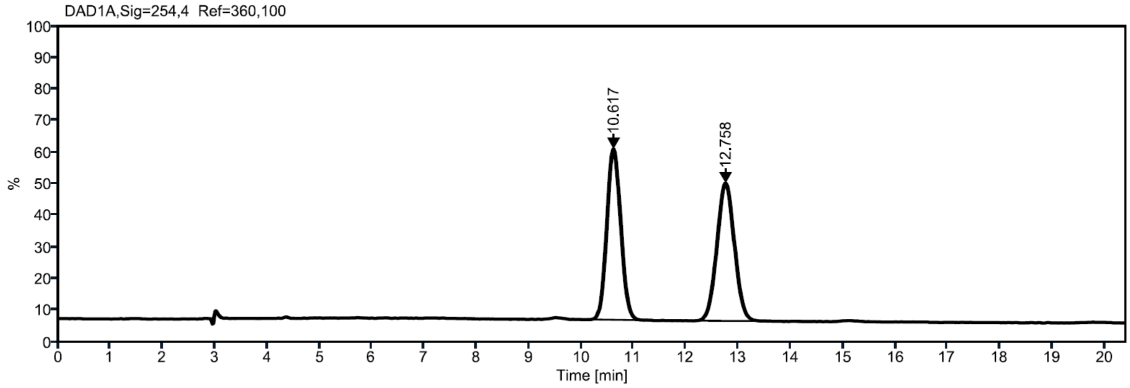

Signal: DAD1A,Sig=254,4 Ref=360,100

RT [min]	Type	Width [min]	Area	Height	Area%
10.617	MM m	0.29	149.68	8.07	49.58
12.758	MM m	0.37	152.24	6.50	50.42
		Sum	301.92		

Chiral

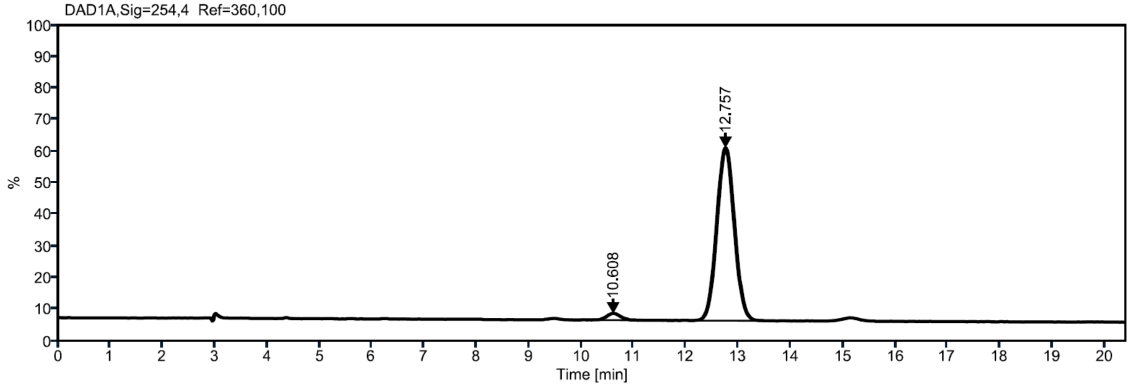

Signal: DAD1A,Sig=254,4 Ref=360,100

RT [min]	Type	Width [min]	Area	Height	Area%
10.608	MM m	0.24	9.97	0.55	2.93
12.757	MM m	0.37	330.18	14.10	97.07
		Sum	340.14		

Fig. 3, Compound 3h

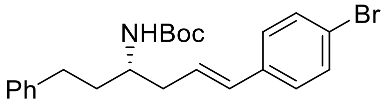

HPLC Conditions

Column: Chiralpak AD-H, Daicel Chemical Industries, Ltd.

Eluent: Hexanes/Isopropanol (95:5)

Flow rate: 1.0 mL/min

Detection: UV 254 nm

Racemic

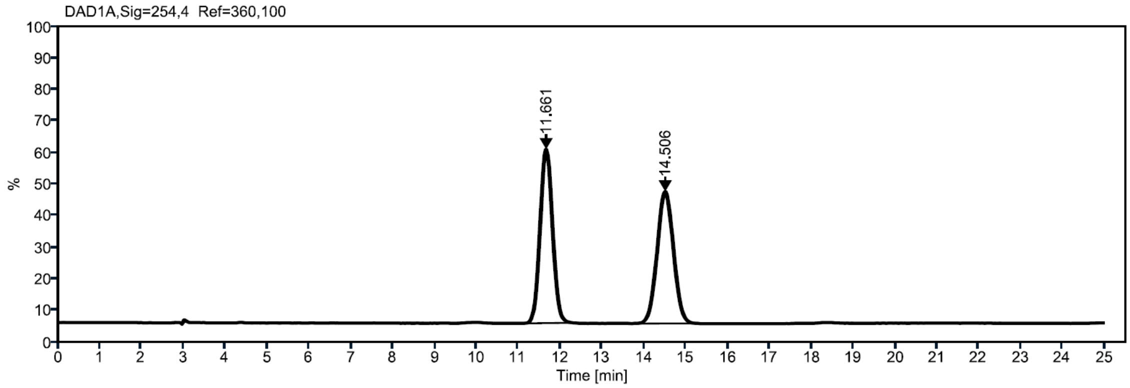

Signal: DAD1A,Sig=254,4 Ref=360,100

RT [min]	Type	Width [min]	Area	Height	Area%
11.661	MM m	0.32	452.44	21.94	49.93
14.506	MM m	0.42	453.73	16.66	50.07
		Sum	906.17		

Chiral

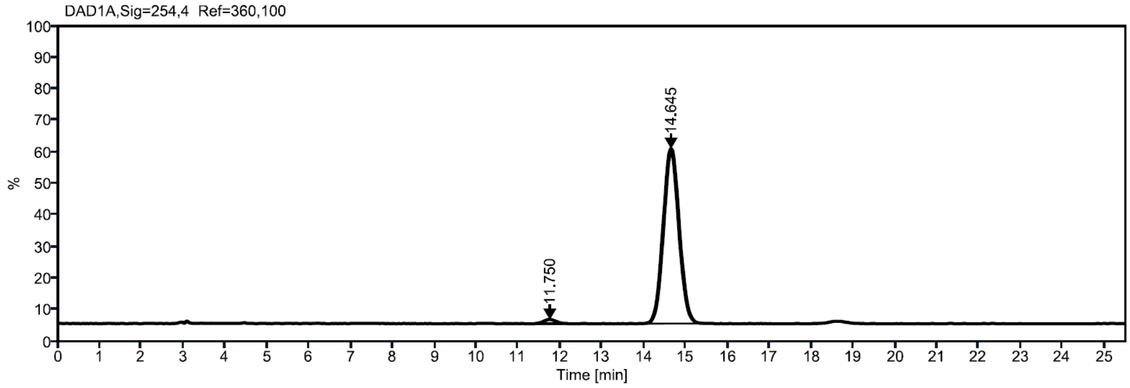

Signal: DAD1A,Sig=254,4 Ref=360,100

RT [min]	Type	Width [min]	Area	Height	Area%
11.750	MM m	0.25	21.38	1.05	1.82
14.645	VB	1.62	1154.87	44.08	98.18
		Sum	1176.25		

Fig. 3, Compound 3i

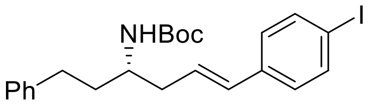

HPLC Conditions

Column: Chiralpak AD-H, Daicel Chemical Industries, Ltd.

Eluent: Hexanes/Isopropanol (95:5)

Flow rate: 1.0 mL/min

Detection: UV 254 nm

Racemic

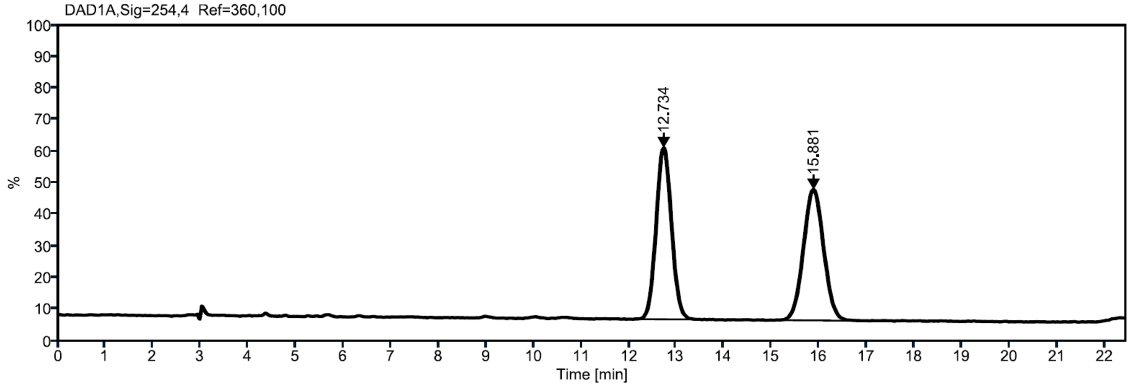

Signal: DAD1A,Sig=254,4 Ref=360,100

RT [min]	Type	Width [min]	Area	Height	Area%
12.734	MM m	0.34	120.69	5.36	49.83
15.881	MM m	0.43	121.49	4.09	50.17
		Sum	242.17		

Chiral

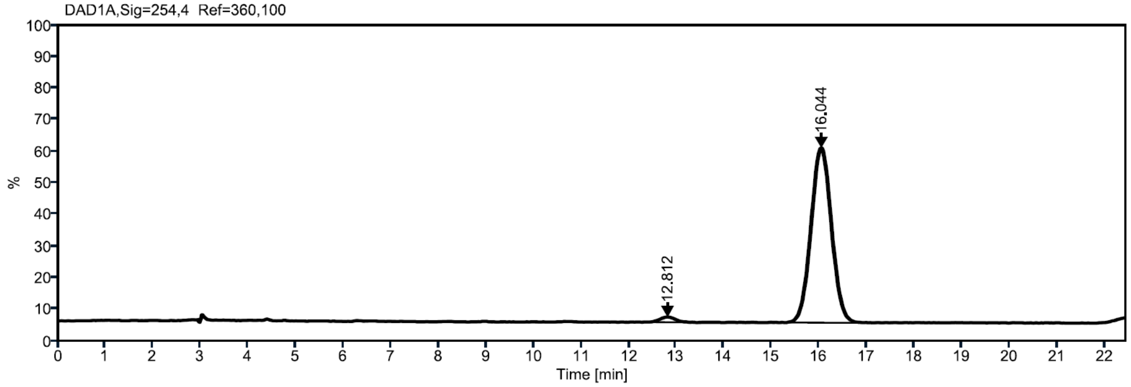

Signal: DAD1A,Sig=254,4 Ref=360,100

RT [min]	Type	Width [min]	Area	Height	Area%
12.812	MM m	0.28	6.61	0.29	2.21
16.044	MM m	0.46	291.99	9.73	97.79
		Sum	298.60		

Fig. 3, Compound 3j

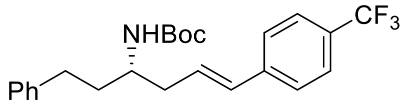

HPLC Conditions

Column: Chiralpak AD-H, Daicel Chemical Industries, Ltd.

Eluent: Hexanes/Isopropanol (95:5)

Flow rate: 1.0 mL/min

Detection: UV 254 nm

Racemic

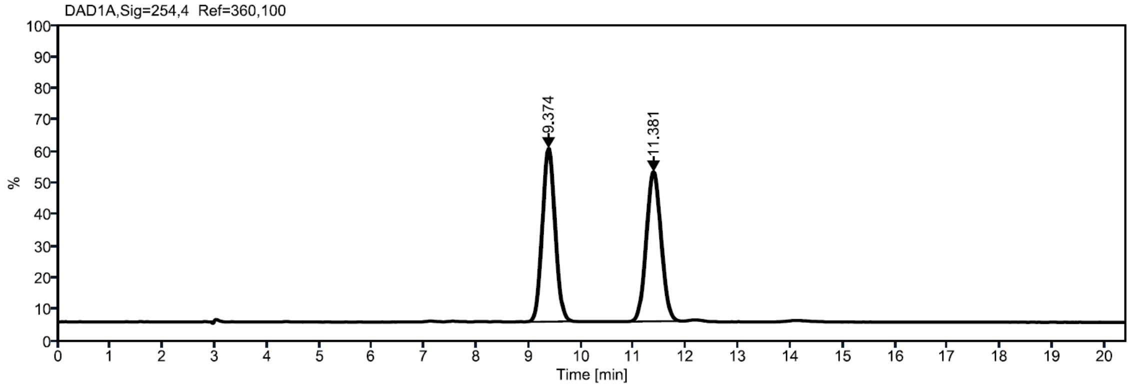

Signal: DAD1A,Sig=254,4 Ref=360,100

RT [min]	Type	Width [min]	Area	Height	Area%
9.374	MM m	0.26	434.68	25.52	50.00
11.381	MM m	0.31	434.71	22.05	50.00
		Sum	869.39		

Chiral

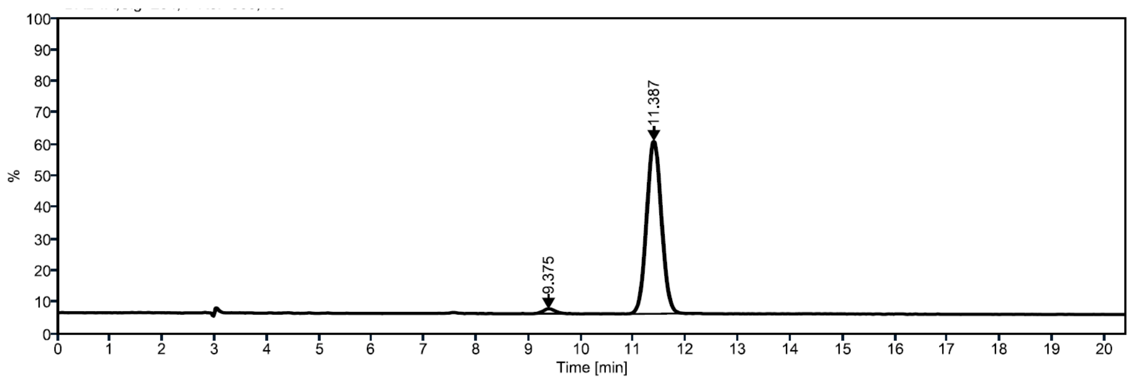

Signal: DAD1A,Sig=254,4 Ref=360,100

RT [min]	Type	Width [min]	Area	Height	Area%
9.375	MM m	0.20	5.24	0.31	2.27
11.387	MM m	0.30	225.20	11.37	97.73
		Sum	230.44		

Fig. 3, Compound 3k

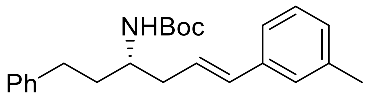

HPLC Conditions

Column: Chiralpak AD-H, Daicel Chemical Industries, Ltd.

Eluent: Hexanes/Isopropanol (97.5:2.5)

Flow rate: 1.0 mL/min

Detection: UV 254 nm

Racemic

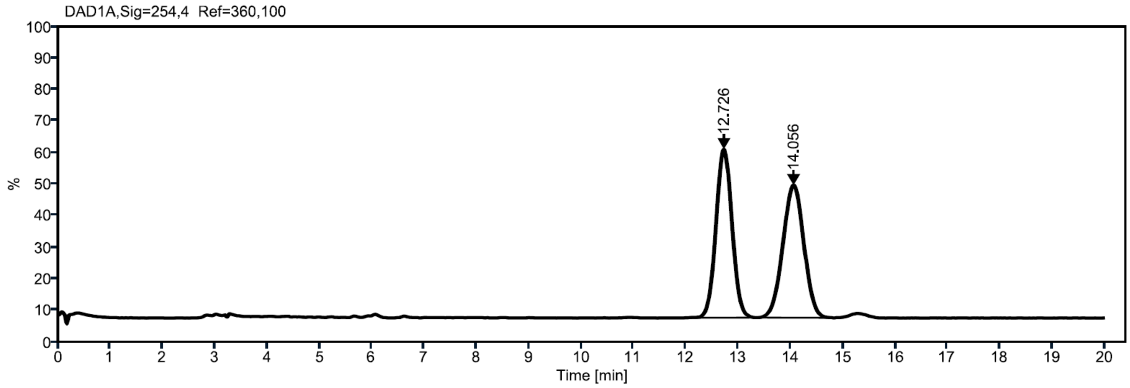

Signal: DAD1A,Sig=254,4 Ref=360,100

RT [min]	Type	Width [min]	Area	Height	Area%
12.726	MM m	0.34	244.22	11.41	49.28
14.056	MM m	0.43	251.39	8.98	50.72
		Sum	495.62		

Chiral

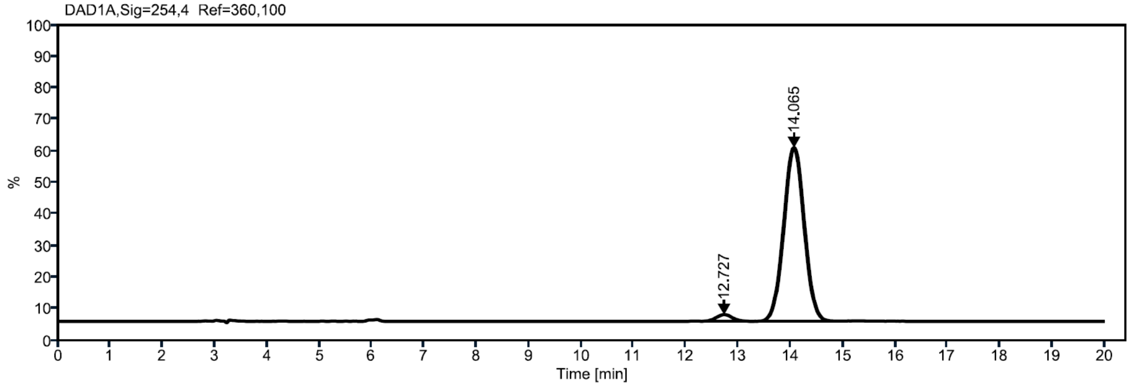

Signal: DAD1A,Sig=254,4 Ref=360,100

RT [min]	Type	Width [min]	Area	Height	Area%
12.727	MM m	0.28	19.83	0.96	2.84
14.065	MM m	0.42	677.92	24.85	97.16
		Sum	697.75		

Fig. 3, Compound 3l

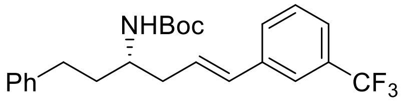

HPLC Conditions

Column: Chiralpak AD-H, Daicel Chemical Industries, Ltd.

Eluent: Hexanes/Isopropanol (95:5)

Flow rate: 1.0 mL/min

Detection: UV 254 nm

Racemic

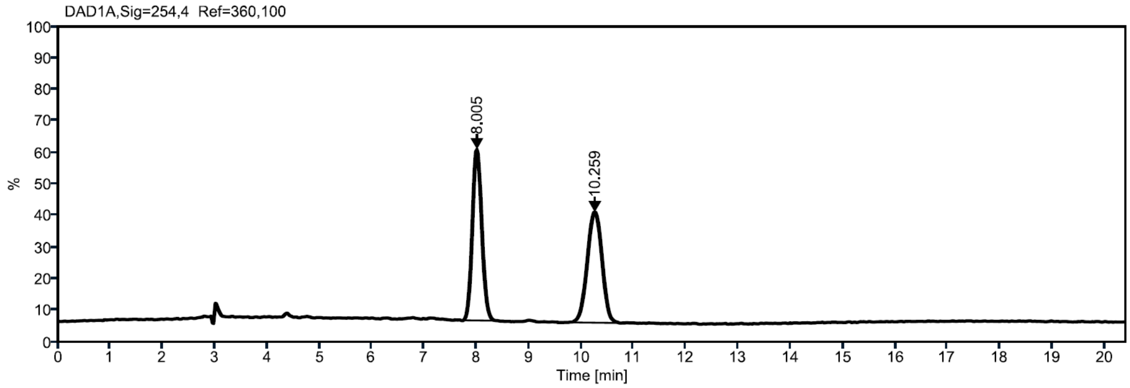

Signal: DAD1A,Sig=254,4 Ref=360,100

RT [min]	Type	Width [min]	Area	Height	Area%
8.005	MM m	0.19	49.58	3.97	49.74
10.259	MM m	0.31	50.09	2.57	50.26
		Sum	99.68		

Chiral

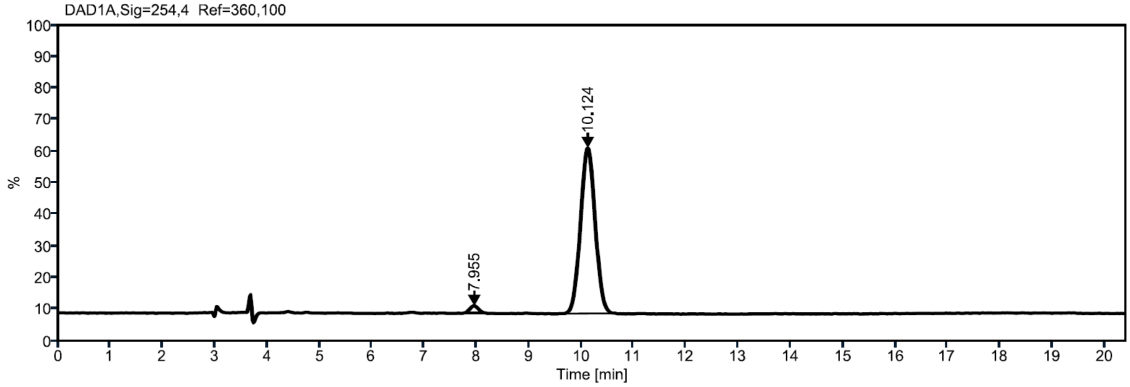

Signal: DAD1A,Sig=254,4 Ref=360,100

RT [min]	Type	Width [min]	Area	Height	Area%
7.955	MM m	0.16	3.06	0.28	2.31
10.124	MM m	0.30	129.36	6.60	97.69
		Sum	132.42		

Fig. 3, Compound 3m

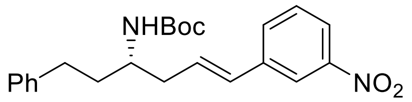

HPLC Conditions

Column: Chiralpak AD-H, Daicel Chemical Industries, Ltd.

Eluent: Hexanes/Isopropanol (95:5)

Flow rate: 1.0 mL/min

Detection: UV 254 nm

Racemic

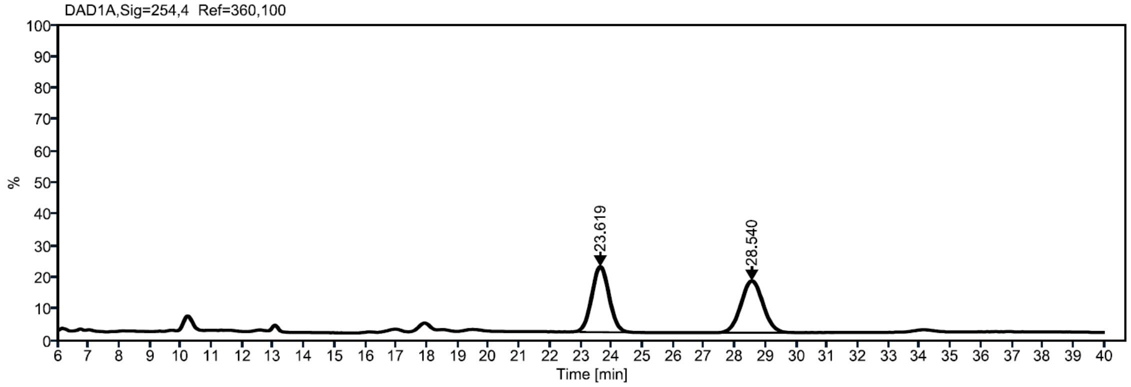

Signal: DAD1A,Sig=254,4 Ref=360,100

RT [min]	Type	Width [min]	Area	Height	Area%
23.619	MM m	0.60	389.00	9.92	50.05
28.540	MM m	0.70	388.26	7.84	49.95
		Sum	777.26		

Chiral

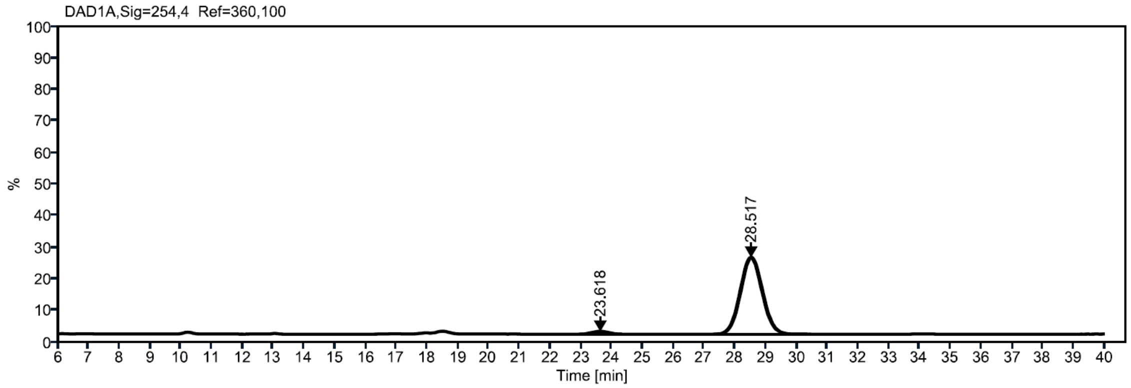

Signal: DAD1A,Sig=254,4 Ref=360,100

RT [min]	Type	Width [min]	Area	Height	Area%
23.618	MM m	0.51	141.99	3.64	2.83
28.517	MM m	0.77	4869.34	97.92	97.17
		Sum	5011.33		

Fig. 3, Compound 3n

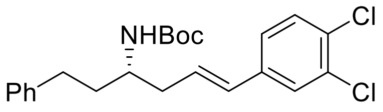

HPLC Conditions

Column: Chiralpak AD-H, Daicel Chemical Industries, Ltd.

Eluent: Hexanes/Isopropanol (95:5)

Flow rate: 1.0 mL/min

Detection: UV 254 nm

Racemic

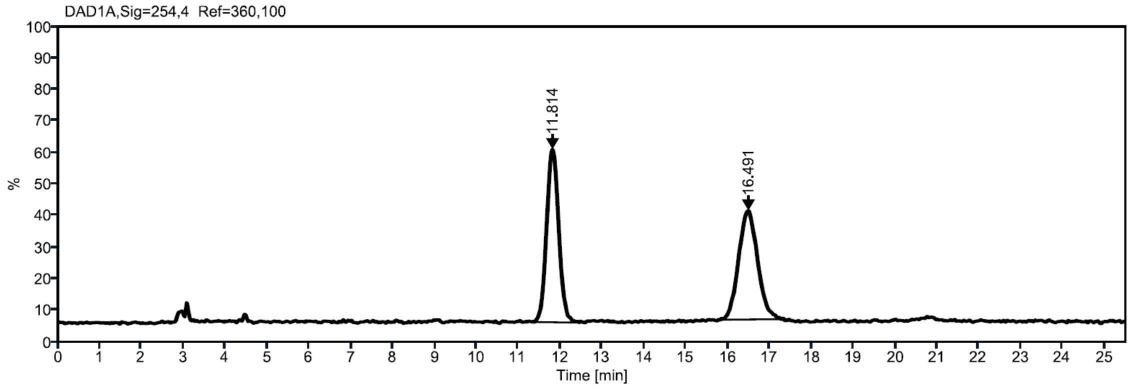

Signal: DAD1A,Sig=254,4 Ref=360,100

RT [min]	Type	Width [min]	Area	Height	Area%
11.814	MM m	0.30	117.62	5.85	50.33
16.491	MM m	0.39	116.09	3.69	49.67
		Sum	233.71		

Chiral

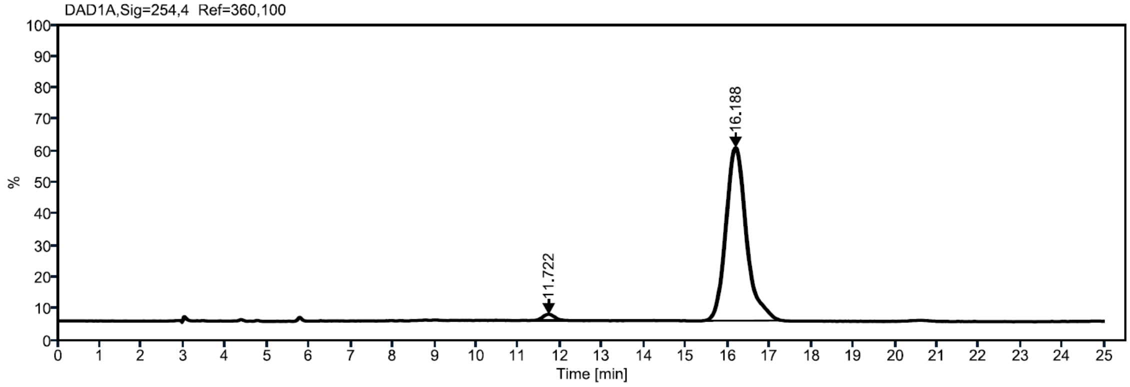

Signal: DAD1A,Sig=254,4 Ref=360,100

RT [min]	Type	Width [min]	Area	Height	Area%
11.722	MM m	0.24	8.85	0.47	1.95
16.188	MM m	0.52	446.14	13.09	98.05
		Sum	454.99		

Fig. 3, Compound 3o

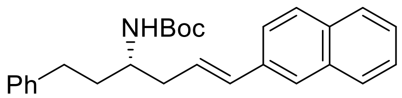

HPLC Conditions

Column: Chiralpak AD-H, Daicel Chemical Industries, Ltd.

Eluent: Hexanes/Isopropanol (95:5)

Flow rate: 1.0 mL/min

Detection: UV 254 nm

Racemic

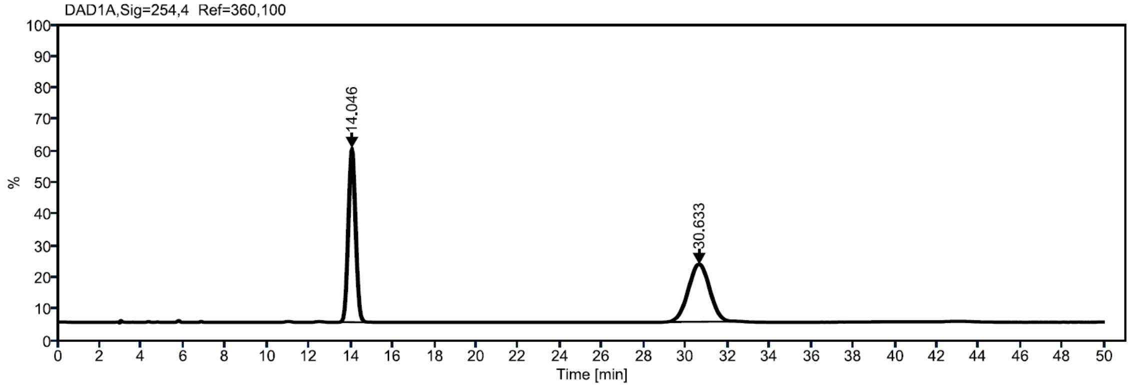

Signal: DAD1A,Sig=254,4 Ref=360,100

RT [min]	Type	Width [min]	Area	Height	Area%
14.046	MM m	0.37	862.16	36.05	50.19
30.633	MM m	0.86	855.61	11.95	49.81
		Sum	1717.77		

Chiral

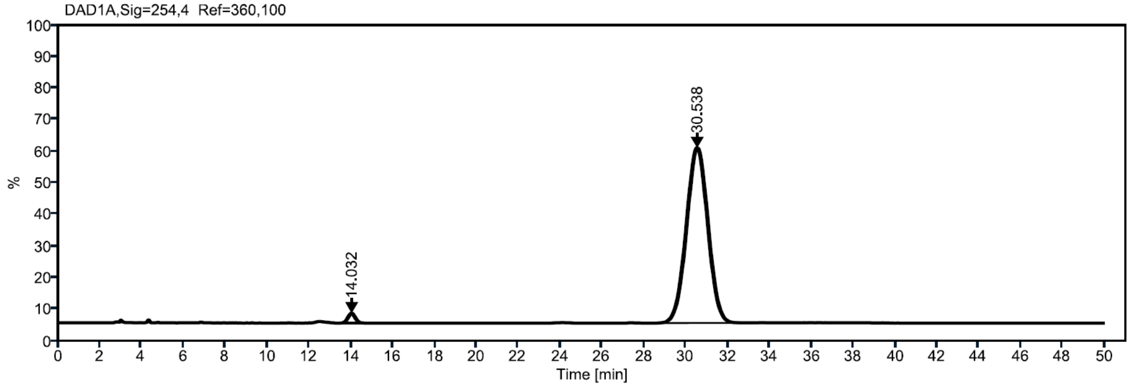

Signal: DAD1A,Sig=254,4 Ref=360,100

RT [min]	Type	Width [min]	Area	Height	Area%
14.032	MM m	0.33	52.54	2.20	1.86
30.538	MM m	1.08	2774.12	38.71	98.14
		Sum	2826.66		

Fig. 3, Compound 3p

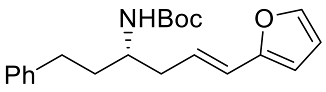

HPLC Conditions

Column: Chiralcel OD-3, Daicel Chemical Industries, Ltd.

Eluent: Hexanes/Isopropanol (95:5)

Flow rate: 1.0 mL/min

Detection: UV 254 nm

Racemic

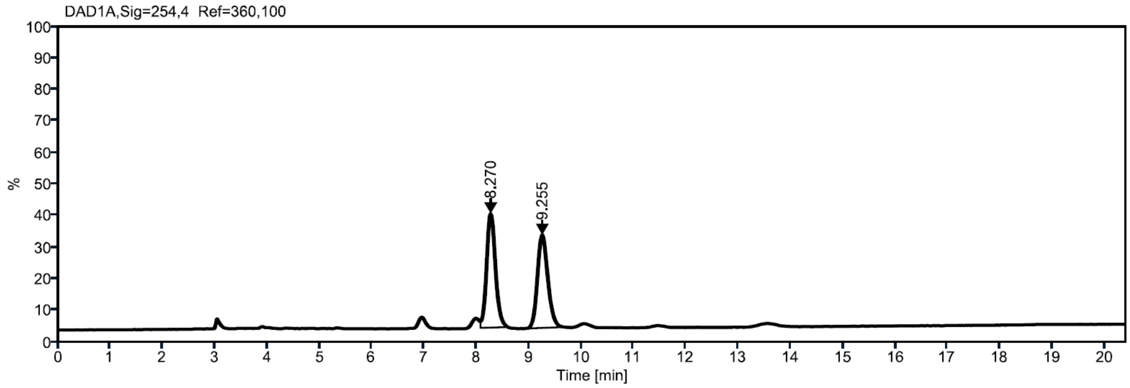

Signal:	DAD1A,Sig=254,4 Ref=360,100				
**RT [min]**	**Type**	**Width [min]**	**Area**	**Height**	**Area%**
8.270	MM m	0.18	142.99	12.33	50.70
9.255	MM m	0.21	139.06	10.04	49.30
		Sum	282.04		

Chiral

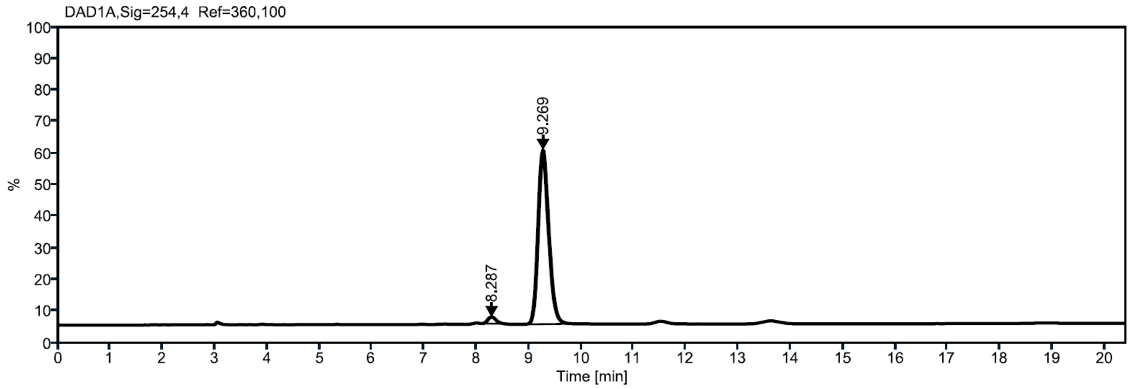

Signal:	DAD1A,Sig=254,4 Ref=360,100				
**RT [min]**	**Type**	**Width [min]**	**Area**	**Height**	**Area%**
8.287	MM m	0.17	24.91	2.33	2.84
9.269	MM m	0.22	853.13	60.55	97.16
		Sum	878.05		

Fig. 3, Compound 3q

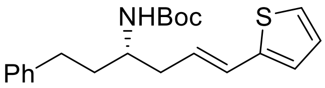

HPLC Conditions

Column: Chiralpak AD-H, Daicel Chemical Industries, Ltd.

Eluent: Hexanes/Isopropanol (95:5)

Flow rate: 1.0 mL/min

Detection: UV 254 nm

Racemic

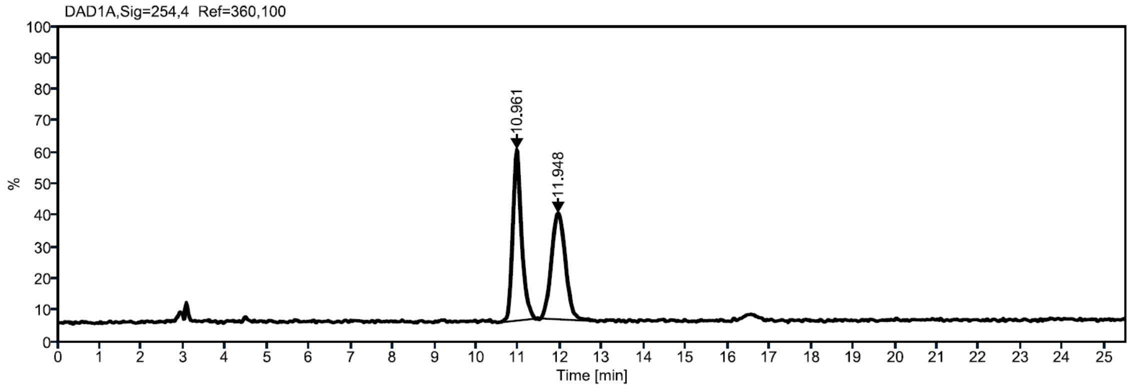

Signal: DAD1A,Sig=254,4 Ref=360,100

RT [min]	Type	Width [min]	Area	Height	Area%
10.961	MM m	0.22	75.00	5.15	50.18
11.948	MM m	0.31	74.48	3.19	49.82
		Sum	149.48		

Chiral

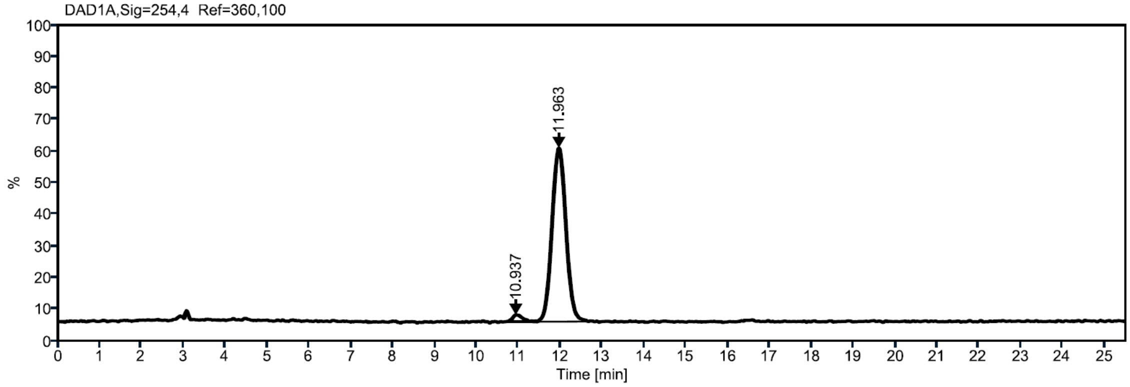

Signal: DAD1A,Sig=254,4 Ref=360,100

RT [min]	Type	Width [min]	Area	Height	Area%
10.937	MM m	0.21	7.43	0.44	2.91
11.963	MM m	0.35	248.30	10.71	97.09
		Sum	255.73		

Fig. 3, Compound 3r

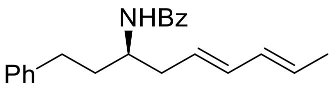

HPLC Conditions

Column: Chiralcel OD-H, Daicel Chemical Industries, Ltd.

Eluent: Hexanes/Isopropanol (90:10)

Flow rate: 1.0 mL/min

Detection: UV 230 nm

Racemic

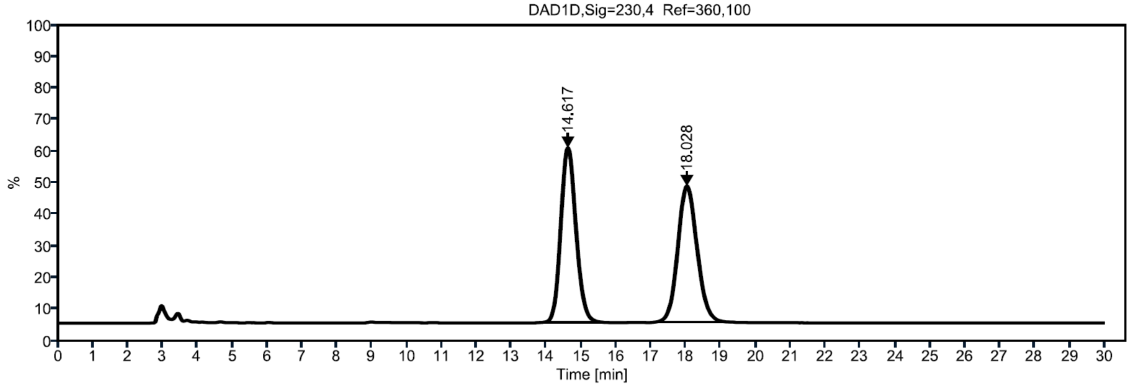

Signal: DAD1D,Sig=230,4 Ref=360,100

RT [min]	Type	Width [min]	Area	Height	Area%
14.617	MM m	0.47	2977.92	98.09	50.22
18.028	MM m	0.60	2952.03	76.49	49.78
		Sum	5929.94		

Chiral

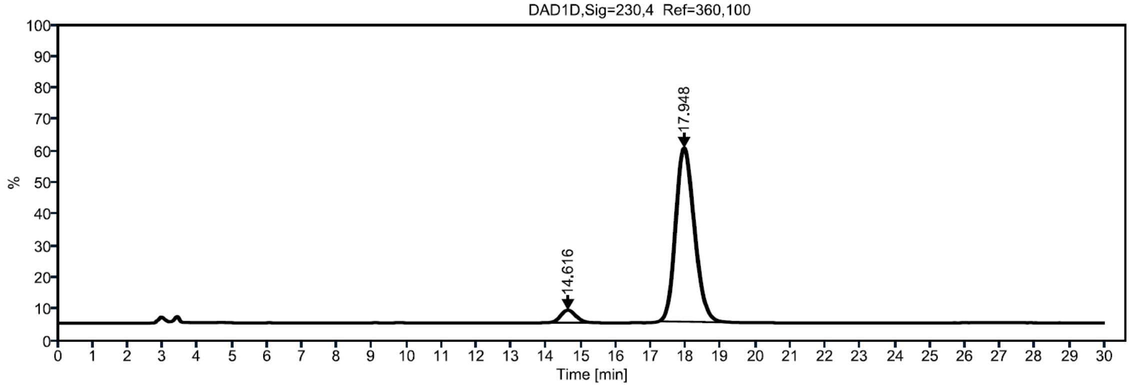

Signal: DAD1D,Sig=230,4 Ref=360,100

RT [min]	Type	Width [min]	Area	Height	Area%
14.616	MM m	0.47	564.43	18.69	5.52
17.948	MM m	0.58	9662.77	257.86	94.48
		Sum	10227.20		

Fig. 3, Compound 3s

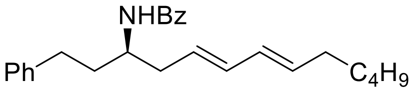

HPLC Conditions

Column: Chiralpak AD-H, Daicel Chemical Industries, Ltd.

Eluent: Hexanes/Isopropanol (97:3)

Flow rate: 1.0 mL/min

Detection: UV 210 nm

Racemic

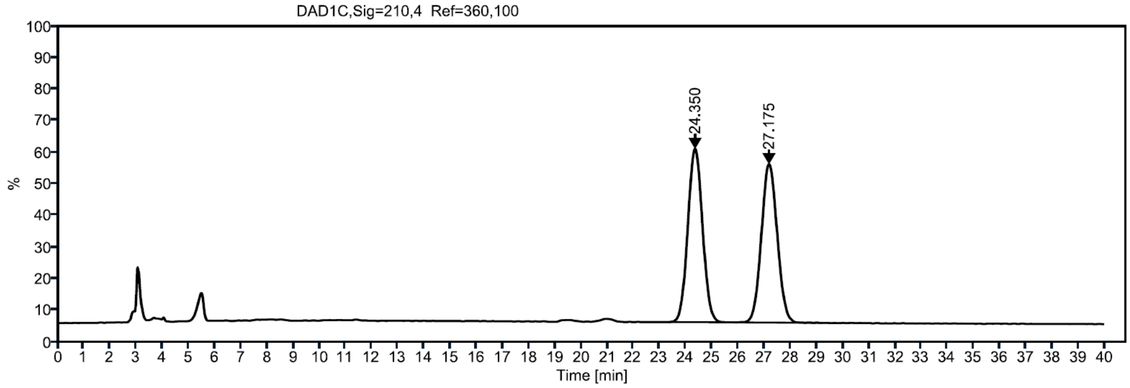

Signal: DAD1C,Sig=210,4 Ref=360,100

RT [min]	Type	Width [min]	Area	Height	Area%
24.350	MM m	0.60	4104.16	104.99	50.23
27.175	MM m	0.65	4067.05	95.87	49.77
		Sum	8171.21		

Chiral

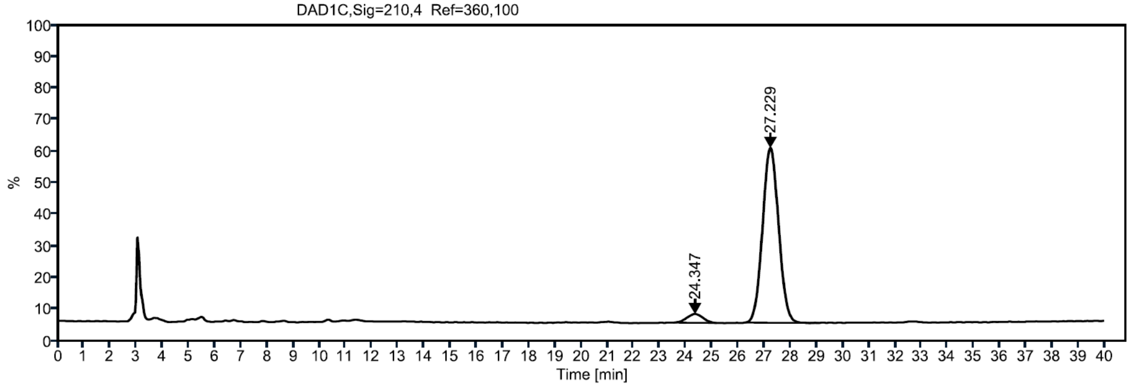

Signal: DAD1C,Sig=210,4 Ref=360,100

RT [min]	Type	Width [min]	Area	Height	Area%
24.347	MM m	0.53	188.74	4.43	4.82
27.229	MM m	0.68	3724.34	87.23	95.18
		Sum	3913.07		

Fig. 3, Compound 3t

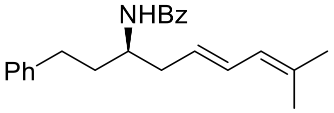

HPLC Conditions

Column: Chiralcel OD-H, Daicel Chemical Industries, Ltd.

Eluent: Hexanes/Isopropanol (90:10)

Flow rate: 1.0 mL/min

Detection: UV 254 nm

Racemic

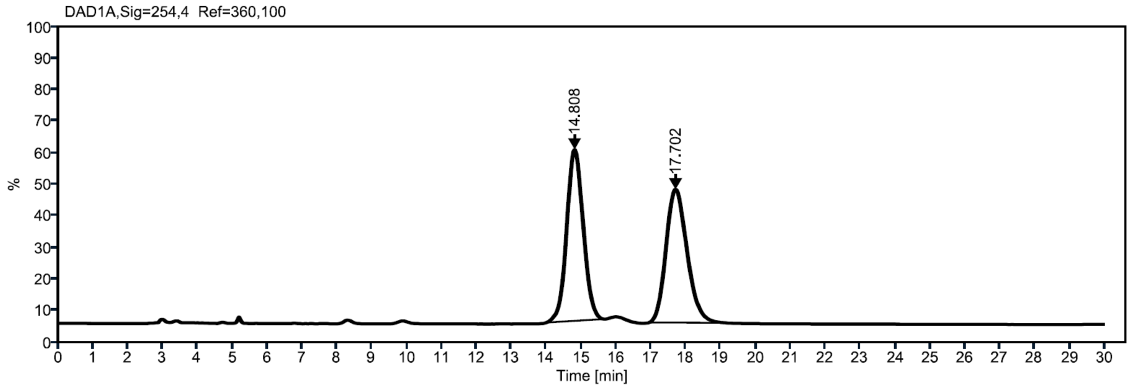

Signal: DAD1A,Sig=254,4 Ref=360,100

RT [min]	Type	Width [min]	Area	Height	Area%
14.808	MM m	0.51	1493.73	45.31	50.24
17.702	MM m	0.65	1479.30	35.23	49.76
		Sum	2973.03		

Chiral

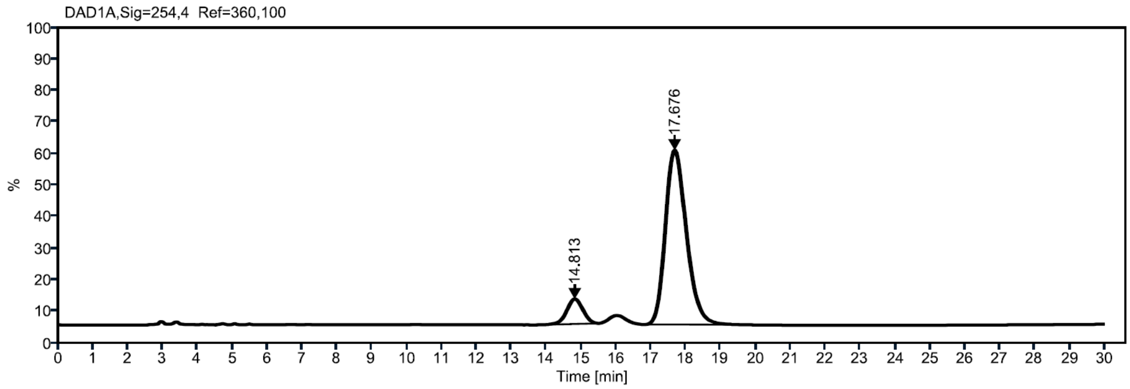

Signal: DAD1A,Sig=254,4 Ref=360,100

RT [min]	Type	Width [min]	Area	Height	Area%
14.813	MM m	0.50	351.18	10.91	9.89
17.676	MM m	0.65	3198.09	76.14	90.11
		Sum	3549.27		

Fig. 3, Compound 3u

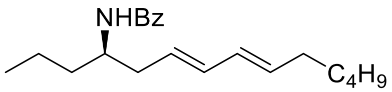

HPLC Conditions

Column: Chiralpak AD-H, Daicel Chemical Industries, Ltd.

Eluent: Hexanes/Isopropanol (97:3)

Flow rate: 1.0 mL/min

Detection: UV 254 nm

Racemic

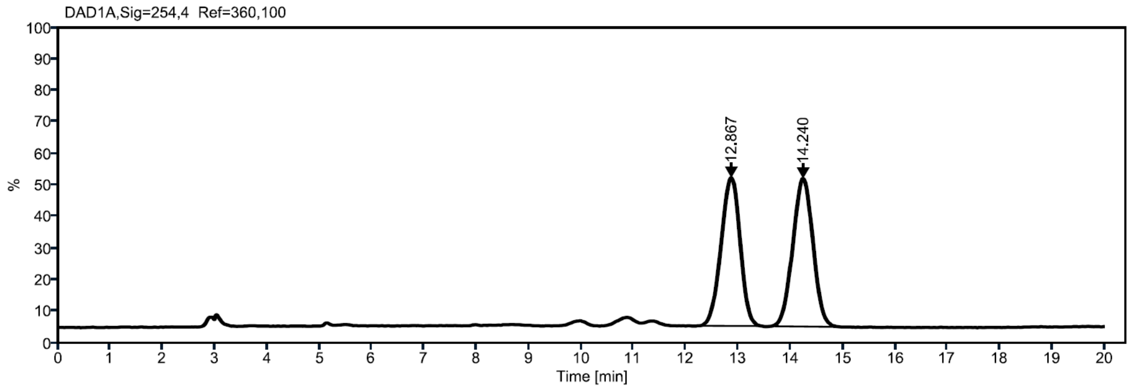

Signal: DAD1A,Sig=254,4 Ref=360,100

RT [min]	Type	Width [min]	Area	Height	Area%
12.867	MM m	0.39	515.95	20.31	48.57
14.240	MM m	0.42	546.22	20.28	51.43
		Sum	1062.17		

Chiral

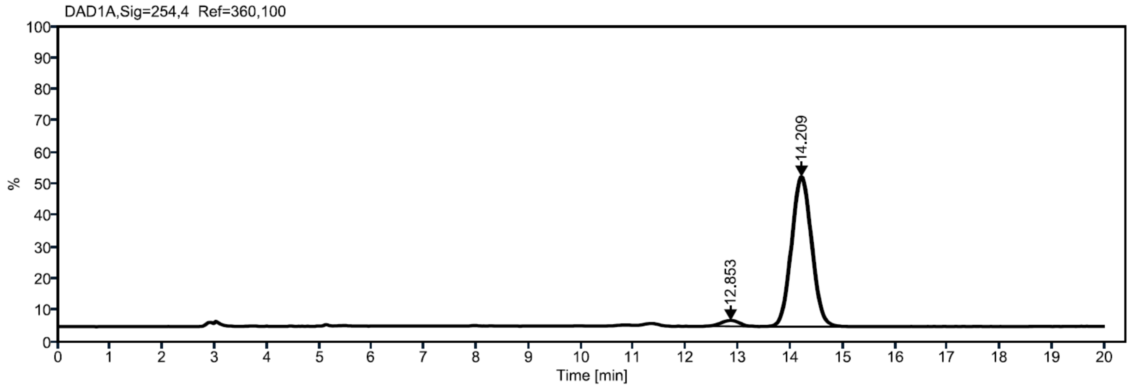

Signal: DAD1A,Sig=254,4 Ref=360,100

RT [min]	Type	Width [min]	Area	Height	Area%
12.853	MM m	0.32	46.43	1.90	3.46
14.209	MM m	0.42	1295.66	48.26	96.54
		Sum	1342.09		

Fig. 3, Compound 3v

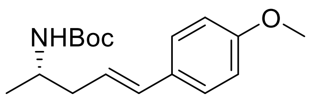

HPLC Conditions

Column: Chiralpak AD-H, Daicel Chemical Industries, Ltd.

Eluent: Hexanes/Isopropanol (90:10)

Flow rate: 1.0 mL/min

Detection: UV 254 nm

Racemic

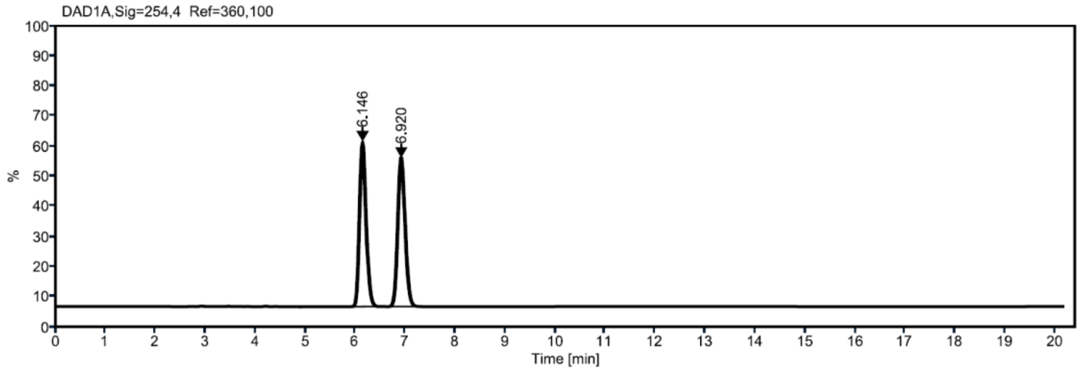

Signal: DAD1A,Sig=254,4 Ref=360,100

RT [min]	Type	Width [min]	Area	Height	Area%
6.146	MM m	0.14	2470.54	270.12	49.88
6.920	MM m	0.16	2482.23	243.17	50.12
		Sum	4952.77		

Chiral

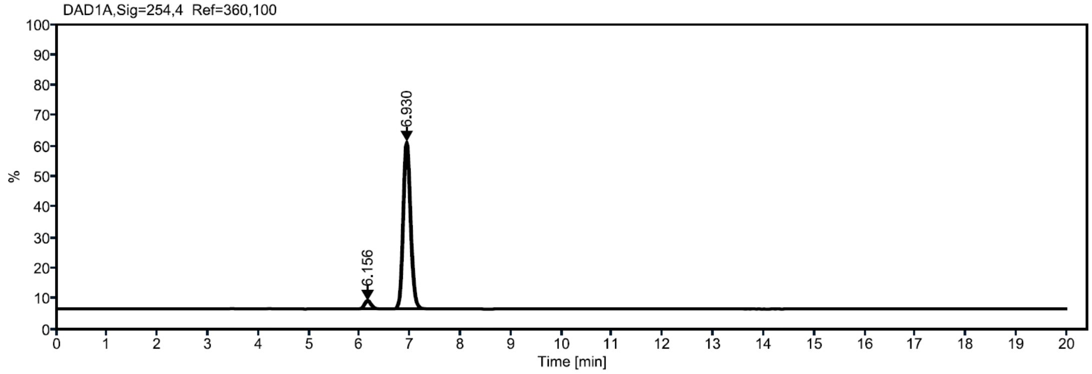

Signal: DAD1A,Sig=254,4 Ref=360,100

RT [min]	Type	Width [min]	Area	Height	Area%
6.156	MM m	0.14	346.77	38.01	4.31
6.930	MM m	0.16	7701.62	748.87	95.69
		Sum	8048.39		

Fig. 3, Compound 3w

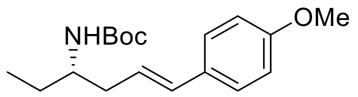

HPLC Conditions

Column: Chiralpak AD-H, Daicel Chemical Industries, Ltd.

Eluent: Hexanes/Isopropanol (95:5)

Flow rate: 1.0 mL/min

Detection: UV 254 nm

Racemic

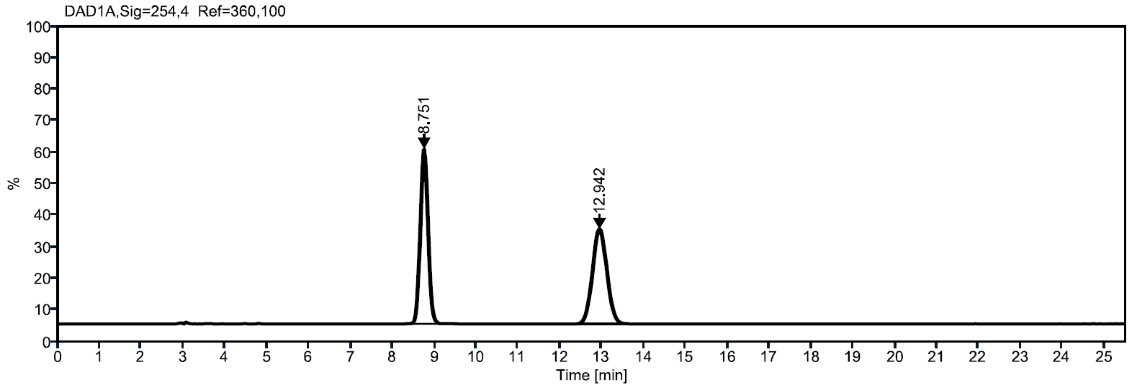

Signal: DAD1A,Sig=254,4 Ref=360,100

RT [min]	Type	Width [min]	Area	Height	Area%
8.751	MM m	0.20	884.90	68.26	49.92
12.942	MM m	0.38	887.80	37.05	50.08
		Sum	1772.70		

Chiral

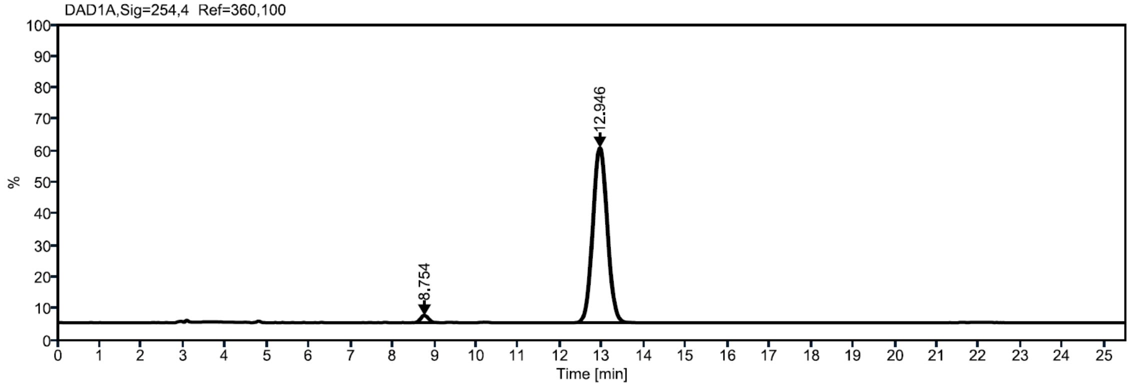

Signal: DAD1A,Sig=254,4 Ref=360,100

RT [min]	Type	Width [min]	Area	Height	Area%
8.754	MM m	0.20	25.16	1.98	2.16
12.946	MM m	0.37	1141.00	47.50	97.84
		Sum	1166.16		

Fig. 3, Compound 3x

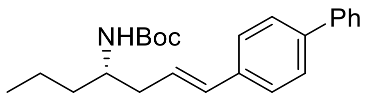

HPLC Conditions

Column: Chiralpak AD-H, Daicel Chemical Industries, Ltd.

Eluent: Hexanes/Isopropanol (90:10)

Flow rate: 1.0 mL/min

Detection: UV 254 nm

Racemic

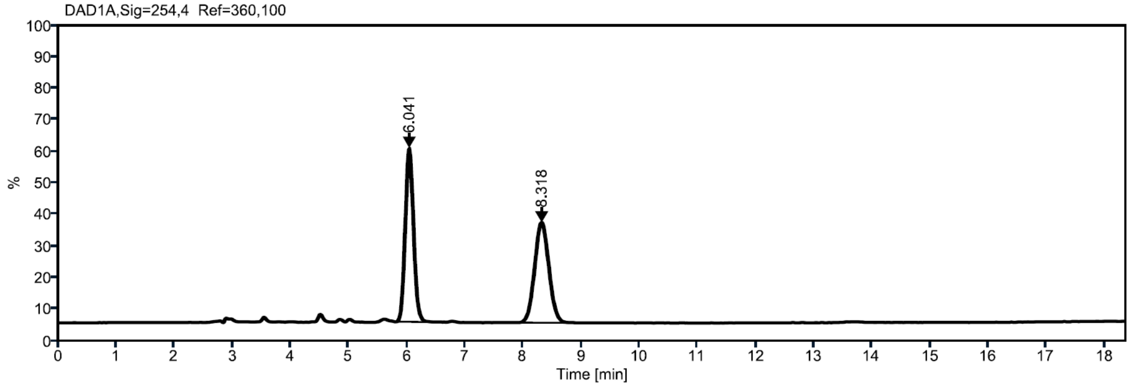

Signal: DAD1A,Sig=254,4 Ref=360,100

RT [min]	Type	Width [min]	Area	Height	Area%
6.041	MM m	0.15	111.51	11.21	50.35
8.318	MM m	0.26	109.97	6.47	49.65
		Sum	221.48		

Chiral

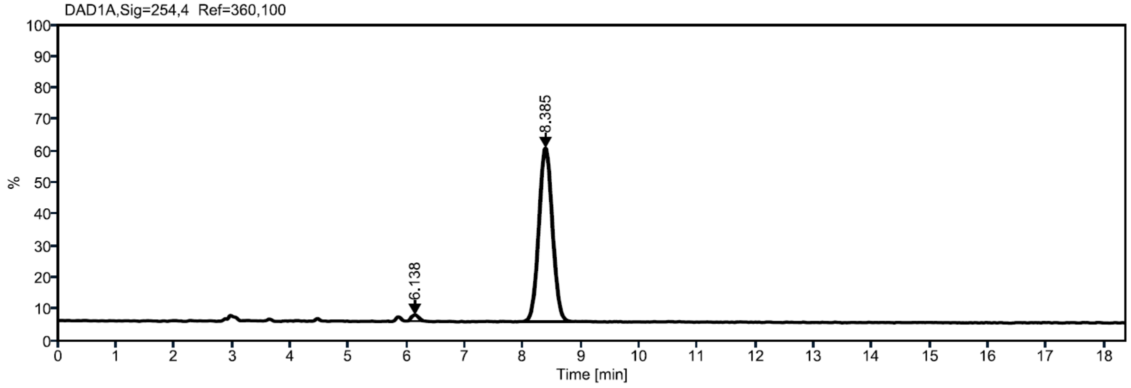

Signal: DAD1A,Sig=254,4 Ref=360,100

RT [min]	Type	Width [min]	Area	Height	Area%
6.138	MM m	0.14	6.70	0.74	2.02
8.385	MM m	0.25	324.71	20.06	97.98
		Sum	331.41		

Fig. 3, Compound 3y

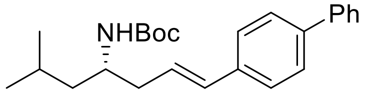

HPLC Conditions

Column: Chiralpak AD-H, Daicel Chemical Industries, Ltd.

Eluent: Hexanes/Isopropanol (95:5)

Flow rate: 1.0 mL/min

Detection: UV 254 nm

Racemic

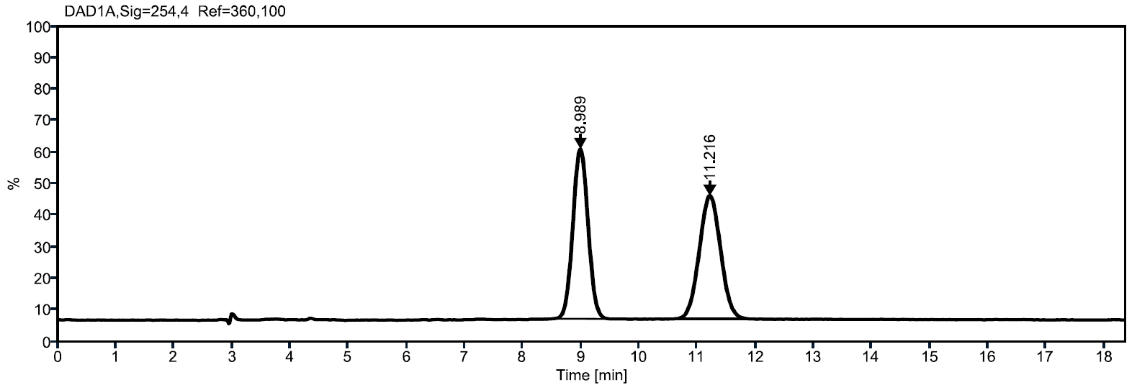

Signal: DAD1A,Sig=254,4 Ref=360,100

RT [min]	Type	Width [min]	Area	Height	Area%
8.989	MM m	0.28	195.73	10.74	49.57
11.216	MM m	0.39	199.10	7.79	50.43
		Sum	394.82		

Chiral

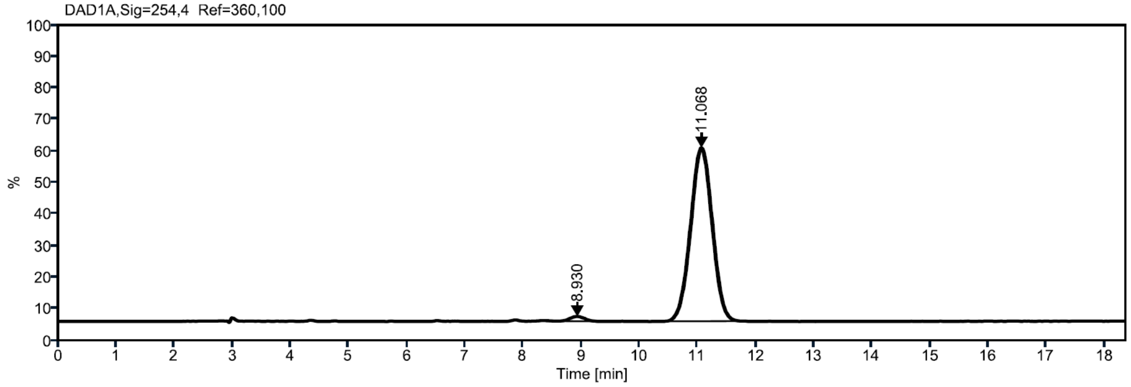

Signal: DAD1A,Sig=254,4 Ref=360,100

RT [min]	Type	Width [min]	Area	Height	Area%
8.930	MM m	0.23	10.17	0.59	1.79
11.068	MM m	0.41	559.19	21.60	98.21
		Sum	569.35		

Fig. 3, Compound 3z

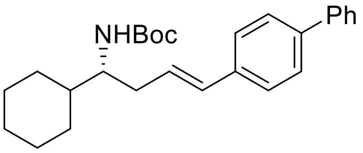

HPLC Conditions

Column: Chiralpak AD-H, Daicel Chemical Industries, Ltd.

Eluent: Hexanes/Isopropanol (95:5)

Flow rate: 1.0 mL/min

Detection: UV 254 nm

Racemic

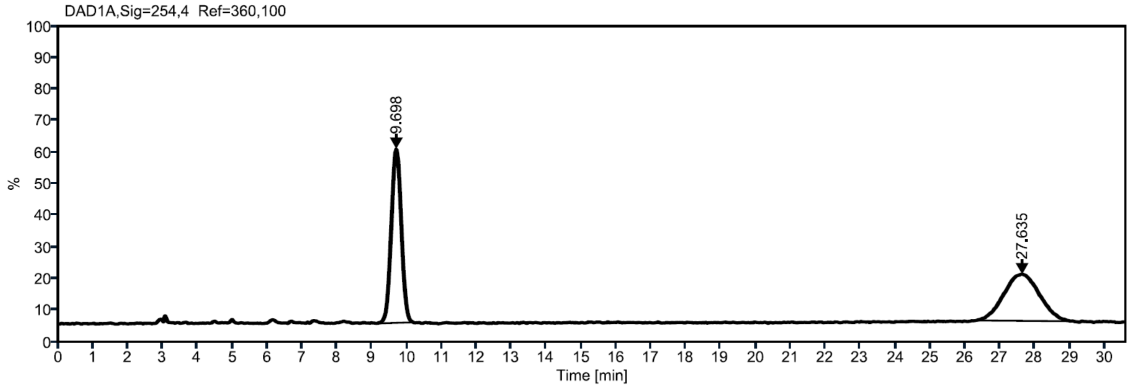

Signal: DAD1A,Sig=254,4 Ref=360,100

RT [min]	Type	Width [min]	Area	Height	Area%
9.698	MM m	0.31	277.99	13.96	50.96
27.635	MM m	0.84	267.50	3.76	49.04
		Sum	545.48		

Chiral

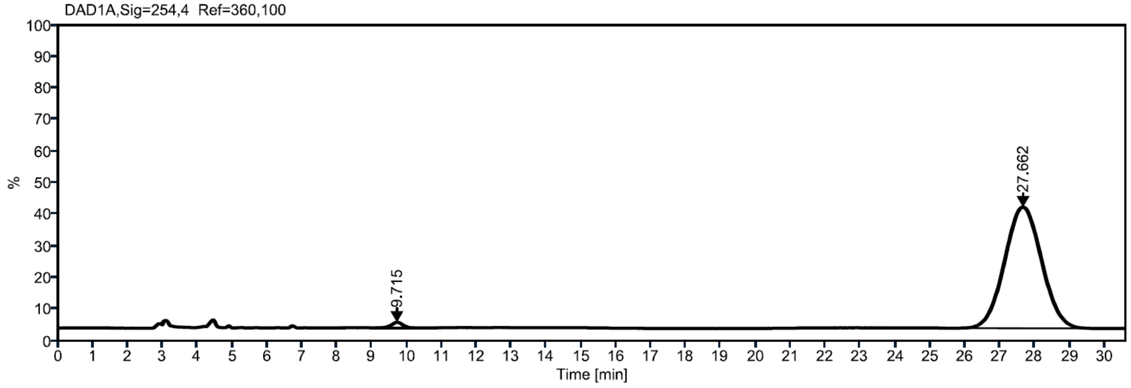

Signal: DAD1A,Sig=254,4 Ref=360,100

RT [min]	Type	Width [min]	Area	Height	Area%
9.715	MM m	0.31	24.44	1.06	1.42
27.662	MM m	1.07	1700.71	23.01	98.58
		Sum	1725.14		

Fig. 3, Compound 3aa

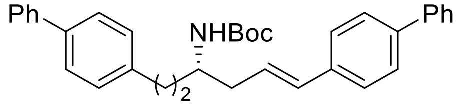

HPLC Conditions

Column: Chiralpak AD-H, Daicel Chemical Industries, Ltd.

Eluent: Hexanes/Isopropanol (95:5)

Flow rate: 1.0 mL/min

Detection: UV 254 nm

Racemic

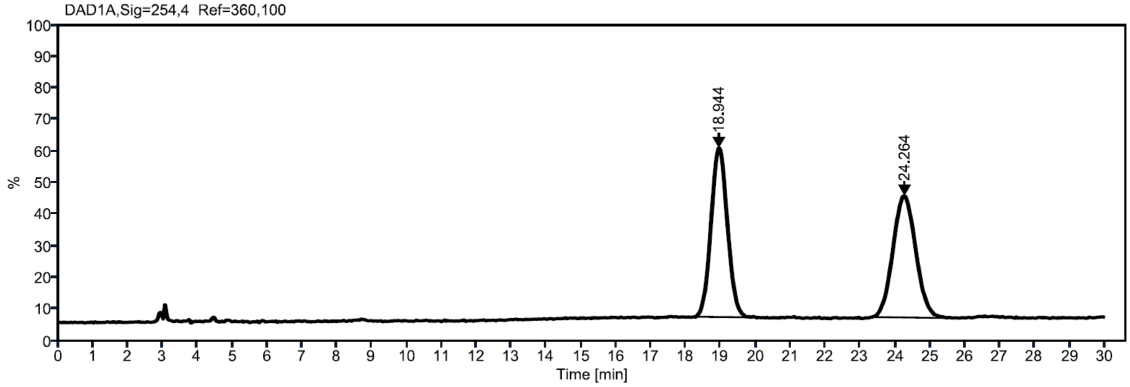

Signal: DAD1A,Sig=254,4 Ref=360,100

RT [min]	Type	Width [min]	Area	Height	Area%
18.944	MM m	0.48	215.17	6.59	49.86
24.264	MM m	0.55	216.39	4.74	50.14
		Sum	431.56		

Chiral

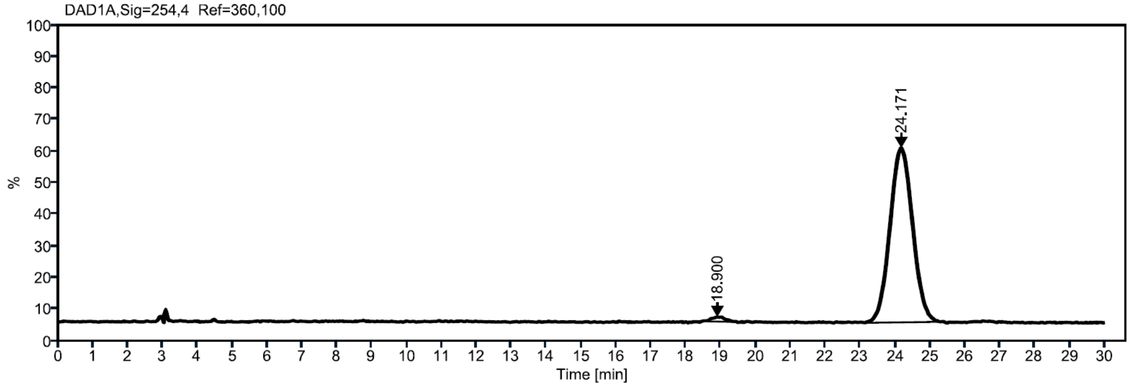

Signal: DAD1A,Sig=254,4 Ref=360,100

RT [min]	Type	Width [min]	Area	Height	Area%
18.900	MM m	0.36	6.69	0.23	1.72
24.171	BB	2.17	382.64	8.46	98.28
		Sum	389.33		

Fig. 3, Compound 3ab

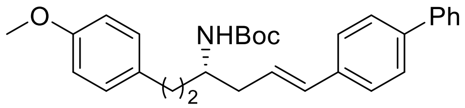

HPLC Conditions

Column: Chiralpak AD-H, Daicel Chemical Industries, Ltd.

Eluent: Hexanes/Isopropanol (95:5)

Flow rate: 1.0 mL/min

Detection: UV 254 nm

Racemic

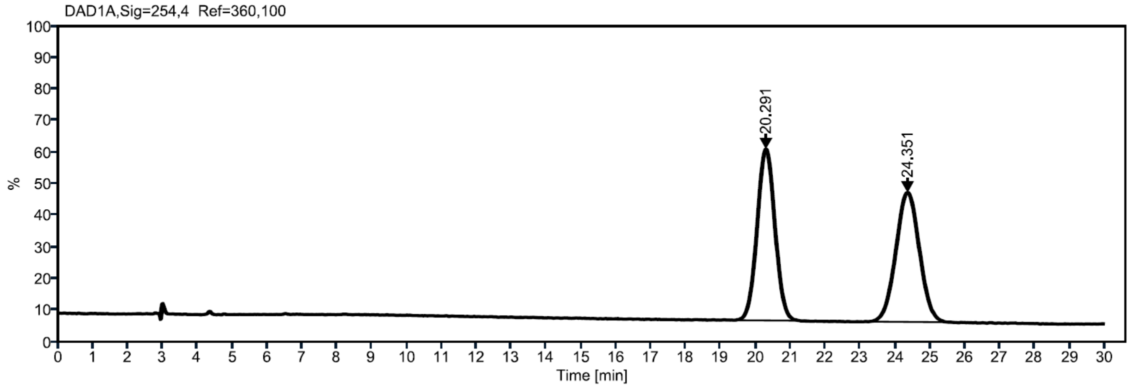

Signal: DAD1A,Sig=254,4 Ref=360,100

RT [min]	Type	Width [min]	Area	Height	Area%
20.291	MM m	0.55	242.68	6.82	50.10
24.351	MM m	0.64	241.75	5.14	49.90
		Sum	484.43		

Chiral

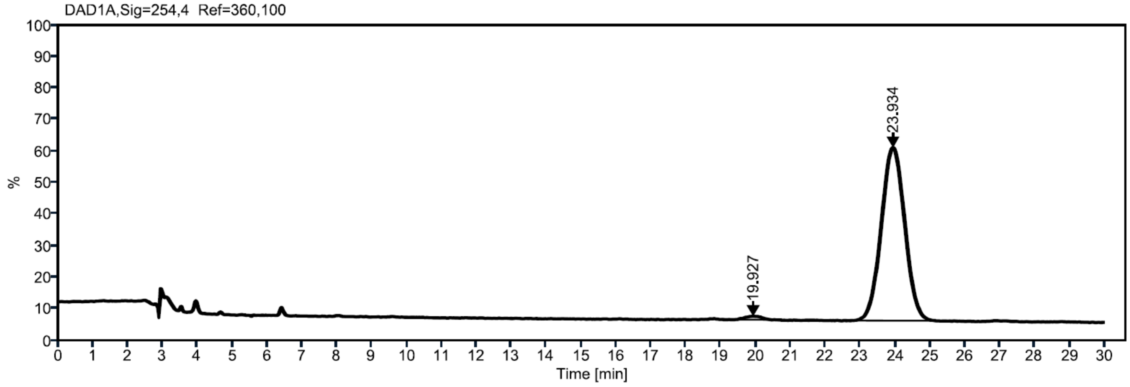

Signal: DAD1A,Sig=254,4 Ref=360,100

RT [min]	Type	Width [min]	Area	Height	Area%
19.927	MM m	0.39	4.41	0.14	1.41
23.934	MM m	0.67	307.47	6.56	98.59
		Sum	311.88		

Fig. 3, Compound 3ac

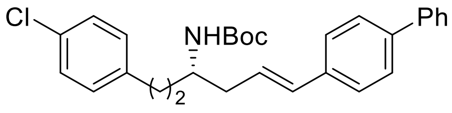

HPLC Conditions

Column: Chiralpak AD-H, Daicel Chemical Industries, Ltd.

Eluent: Hexanes/Isopropanol (95:5)

Flow rate: 1.0 mL/min

Detection: UV 254 nm

Racemic

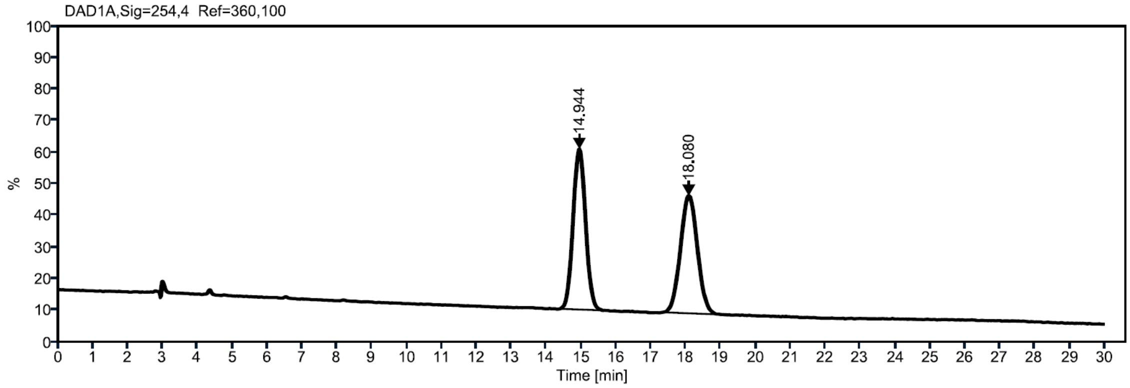

Signal: DAD1A,Sig=254,4 Ref=360,100

RT [min]	Type	Width [min]	Area	Height	Area%
14.944	MM m	0.38	157.30	6.31	50.04
18.080	MM m	0.51	157.05	4.62	49.96
		Sum	314.35		

Chiral

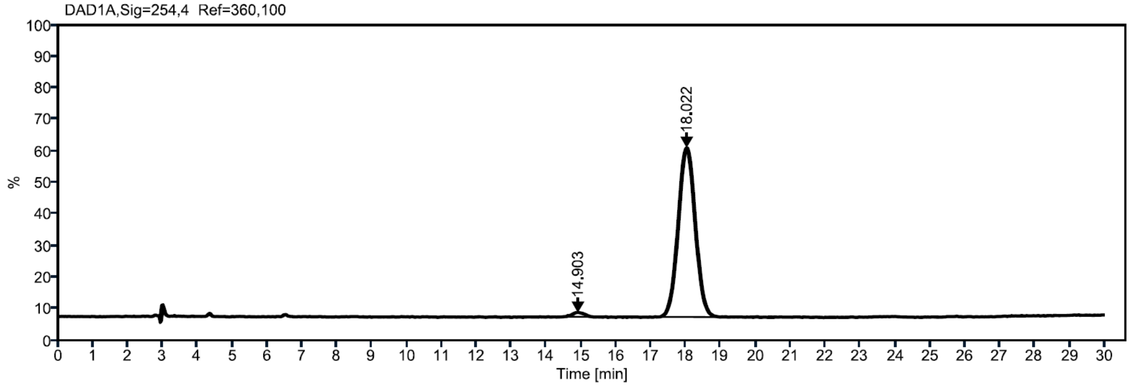

Signal: DAD1A,Sig=254,4 Ref=360,100

RT [min]	Type	Width [min]	Area	Height	Area%
14.903	MM m	0.28	3.80	0.16	1.79
18.022	MM m	0.51	209.14	6.14	98.21
		Sum	212.94		

Fig. 3, Compound 3ad

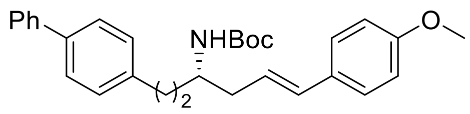

HPLC Conditions

Column: Chiralpak AD-H, Daicel Chemical Industries, Ltd.

Eluent: Hexanes/Isopropanol (95:5)

Flow rate: 1.0 mL/min

Detection: UV 254 nm

Racemic

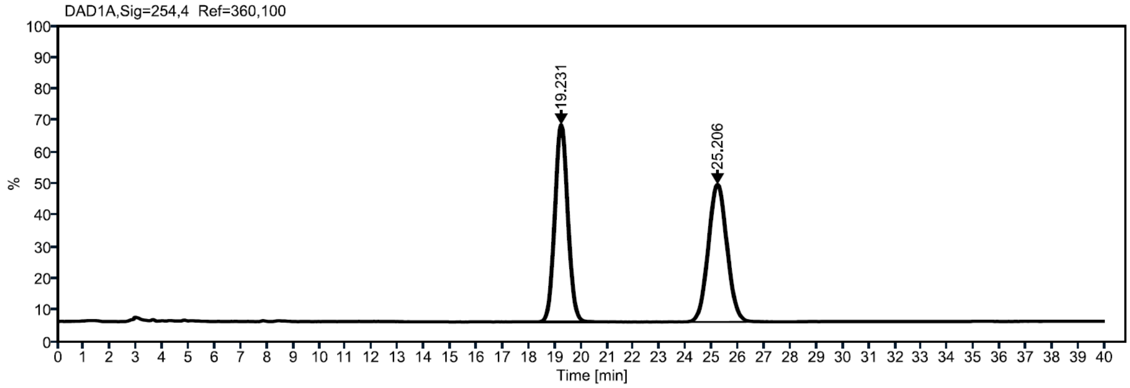

Signal: DAD1A,Sig=254,4 Ref=360,100

RT [min]	Type	Width [min]	Area	Height	Area%
19.231	MM m	0.54	2981.94	86.15	49.94
25.206	MM m	0.77	2988.83	60.07	50.06
		Sum	5970.77		

Chiral

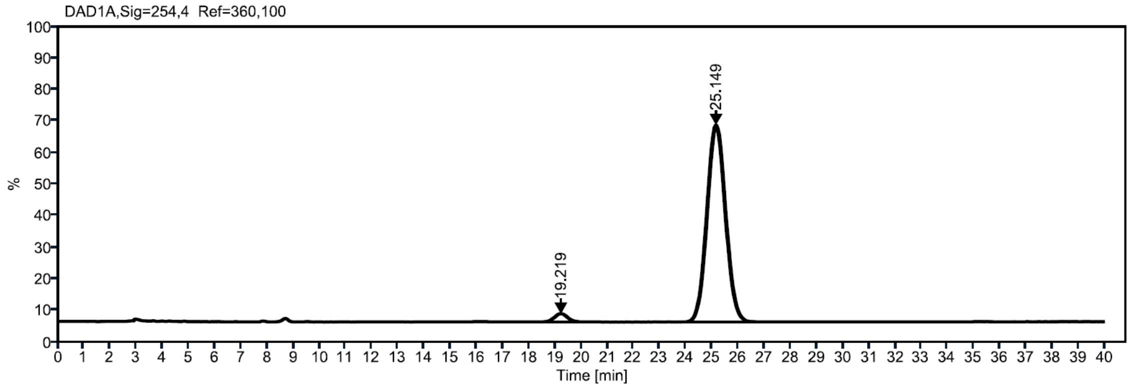

Signal: DAD1A,Sig=254,4 Ref=360,100

RT [min]	Type	Width [min]	Area	Height	Area%
19.219	MM m	0.45	172.48	5.01	2.84
25.149	MM m	0.77	5895.37	119.21	97.16
		Sum	6067.85		

Fig. 3, Compound 3ae

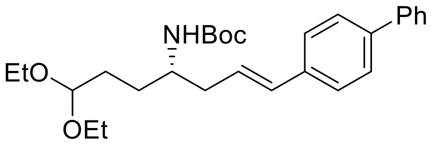

HPLC Conditions

Column: Chiralpak AD-H, Daicel Chemical Industries, Ltd.

Eluent: Hexanes/Isopropanol (95:5)

Flow rate: 1.0 mL/min

Detection: UV 254 nm

Racemic

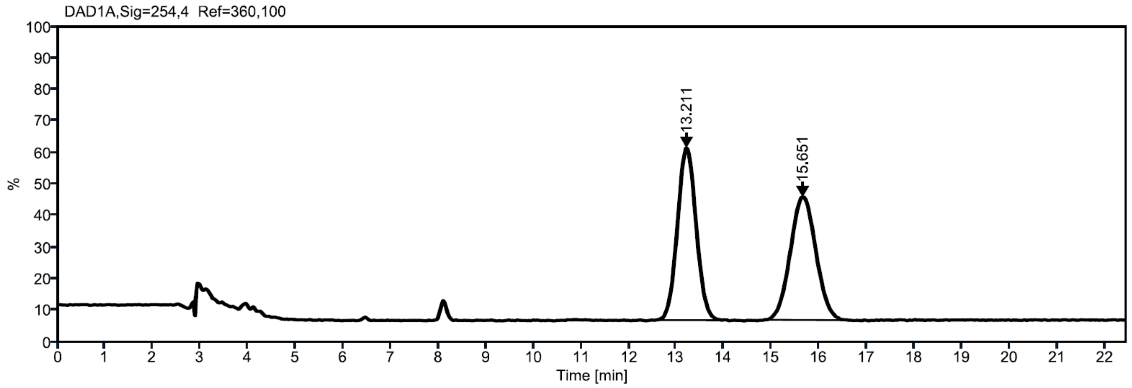

Signal: DAD1A,Sig=254,4 Ref=360,100

RT [min]	Type	Width [min]	Area	Height	Area%
13.211	MM m	0.41	151.33	5.52	50.63
15.651	MM m	0.49	147.53	3.94	49.37
		Sum	298.86		

Chiral

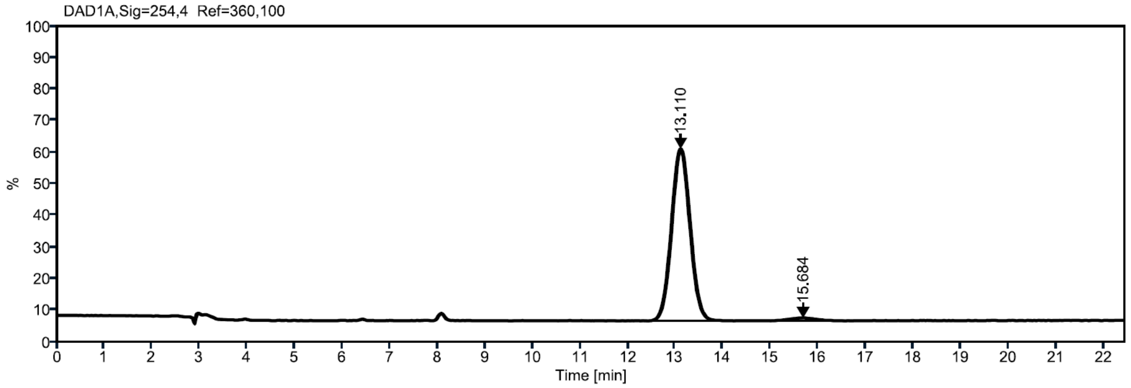

Signal: DAD1A,Sig=254,4 Ref=360,100

RT [min]	Type	Width [min]	Area	Height	Area%
13.110	MM m	0.42	434.69	16.05	97.82
15.684	MM m	0.42	9.67	0.28	2.18
		Sum	444.36		

Fig. 3, Compound 3af

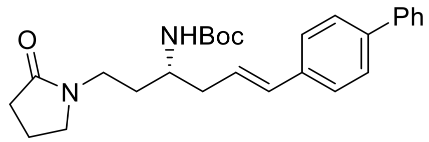

HPLC Conditions

Column: Chiralpak AD-H, Daicel Chemical Industries, Ltd.

Eluent: Hexanes/Isopropanol (90:10)

Flow rate: 1.0 mL/min

Detection: UV 254 nm

Racemic

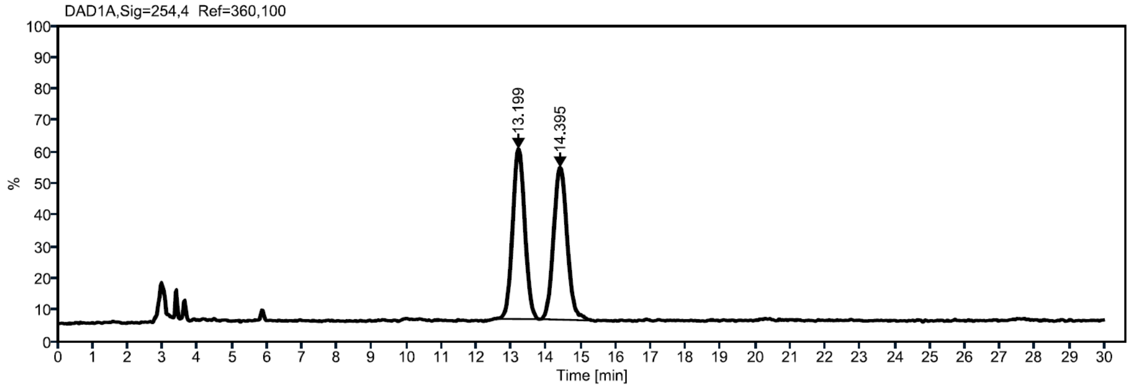

Signal: DAD1A,Sig=254,4 Ref=360,100

RT [min]	Type	Width [min]	Area	Height	Area%
13.199	MM m	0.34	111.28	4.57	50.07
14.395	MM m	0.40	110.97	4.08	49.93
		Sum	222.25		

Chiral

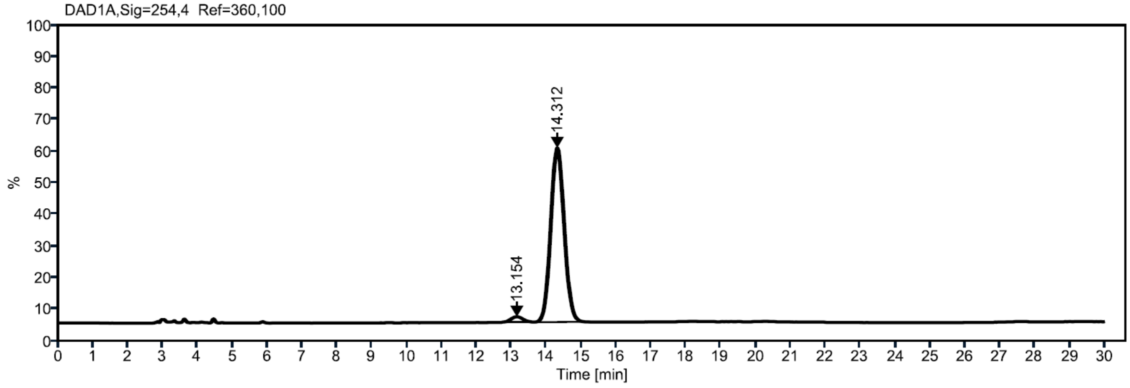

Signal: DAD1A,Sig=254,4 Ref=360,100

RT [min]	Type	Width [min]	Area	Height	Area%
13.154	MM m	0.30	30.96	1.32	2.65
14.312	MM m	0.42	1136.46	42.21	97.35
		Sum	1167.42		

Fig. 3, Compound 3ag

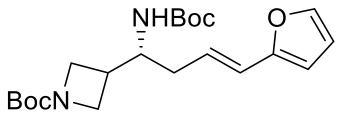

HPLC Conditions

Column: Chiralcel OD-H, Daicel Chemical Industries, Ltd.

Eluent: Hexanes/Isopropanol (95:5)

Flow rate: 1.0 mL/min

Detection: UV 254 nm

Racemic

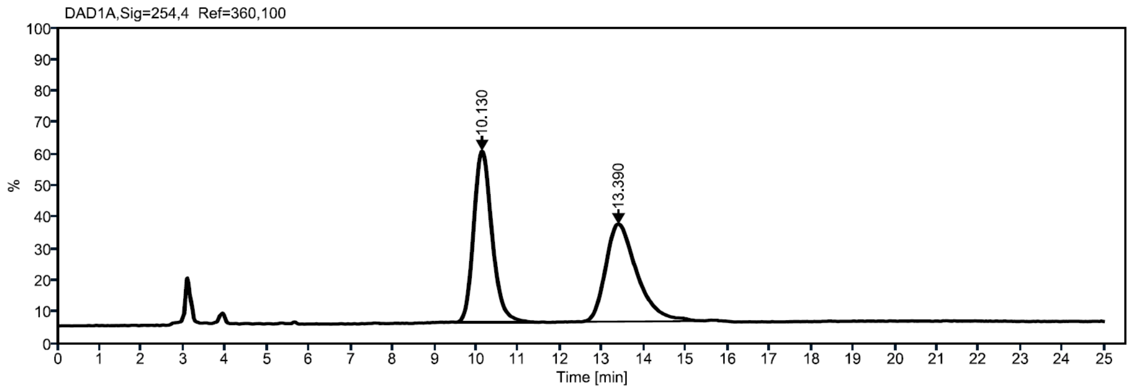

Signal: DAD1A,Sig=254,4 Ref=360,100

RT [min]	Type	Width [min]	Area	Height	Area%
10.130	BB	1.90	574.94	18.87	50.71
13.390	BB	2.93	558.76	10.72	49.29
		Sum	1133.70		

Chiral

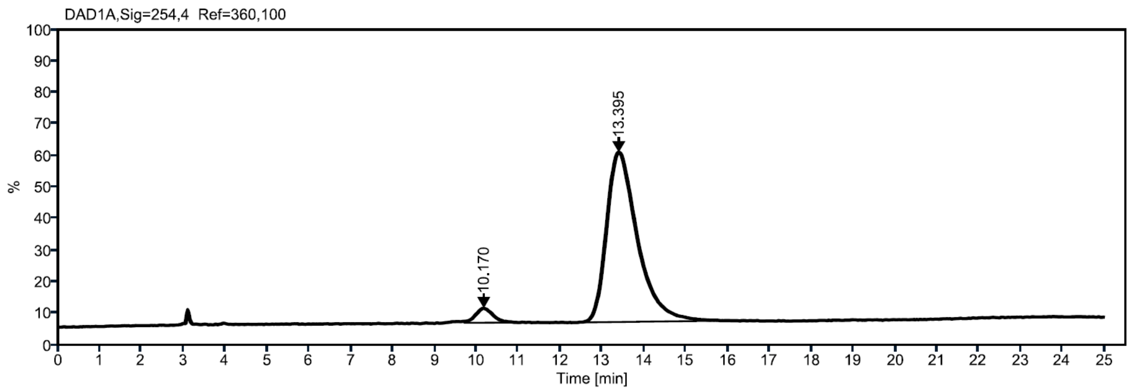

Signal: DAD1A,Sig=254,4 Ref=360,100

RT [min]	Type	Width [min]	Area	Height	Area%
10.170	MM m	0.35	34.58	1.20	4.60
13.395	MM m	0.68	716.84	13.96	95.40
		Sum	751.42		

Fig. 3, Compound (*R*)-3ah

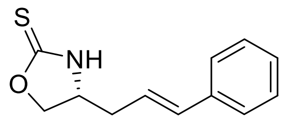

HPLC Conditions

Column: Chiralpak AD-H, Daicel Chemical Industries, Ltd.

Eluent: Hexanes/Isopropanol (90:10)

Flow rate: 1.0 mL/min

Detection: UV 254 nm

Racemic

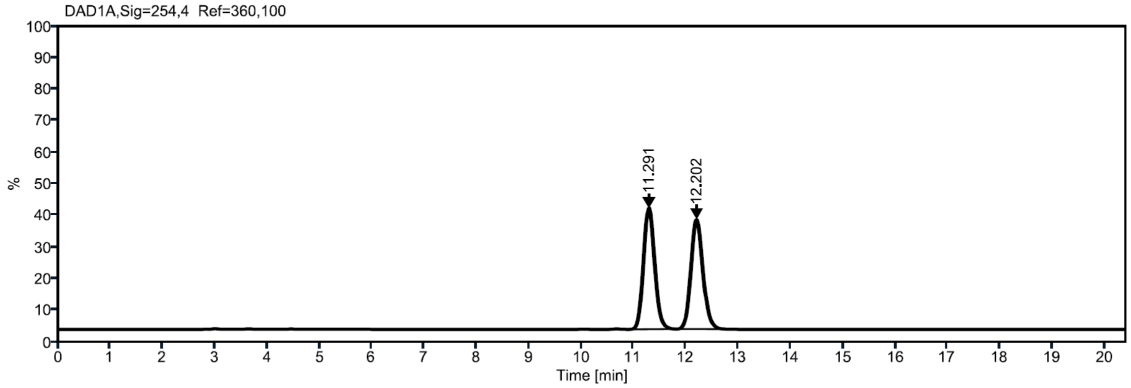

**Signal:**	DAD1A,Sig=254,4 Ref=360,100				
**RT [min]**	**Type**	**Width [min]**	**Area**	**Height**	**Area%**
11.291	MM m	0.23	2692.62	181.49	50.34
12.202	MM m	0.25	2656.65	164.42	49.66
		Sum	5349.27		

Chiral

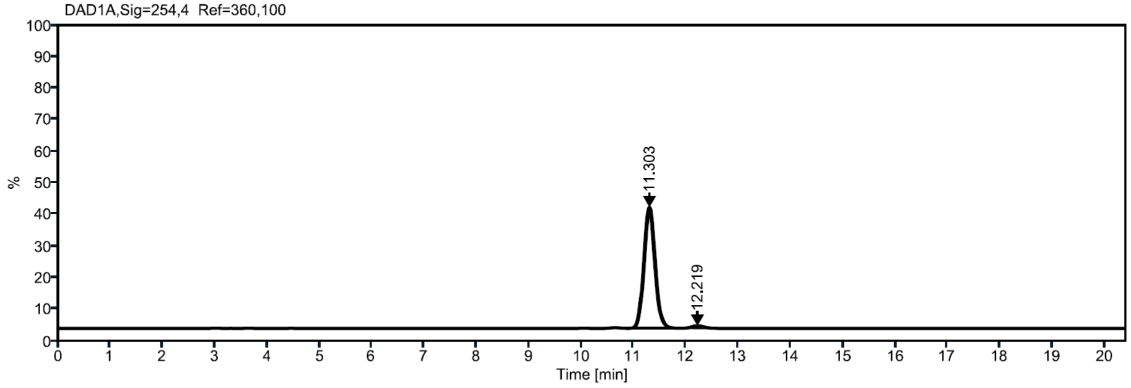

**Signal:**	DAD1A,Sig=254,4 Ref=360,100				
**RT [min]**	**Type**	**Width [min]**	**Area**	**Height**	**Area%**
11.303	MM m	0.23	7426.25	502.00	97.82
12.219	MM m	0.24	165.59	10.69	2.18
		Sum	7591.84		

Fig. 3, Compound (*S*)-3ah

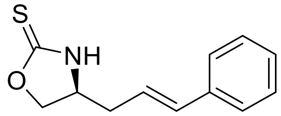

HPLC Conditions

Column: Chiralpak AD-H, Daicel Chemical Industries, Ltd.

Eluent: Hexanes/Isopropanol (90:10)

Flow rate: 1.0 mL/min

Detection: UV 254 nm

Racemic

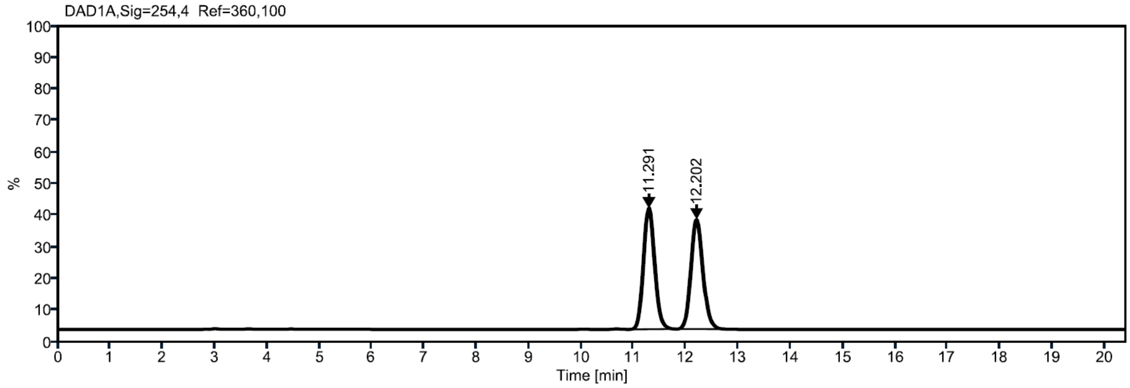

Signal: DAD1A,Sig=254,4 Ref=360,100

RT [min]	Type	Width [min]	Area	Height	Area%
11.291	MM m	0.23	2692.62	181.49	50.34
12.202	MM m	0.25	2656.65	164.42	49.66
		Sum	5349.27		

Chiral

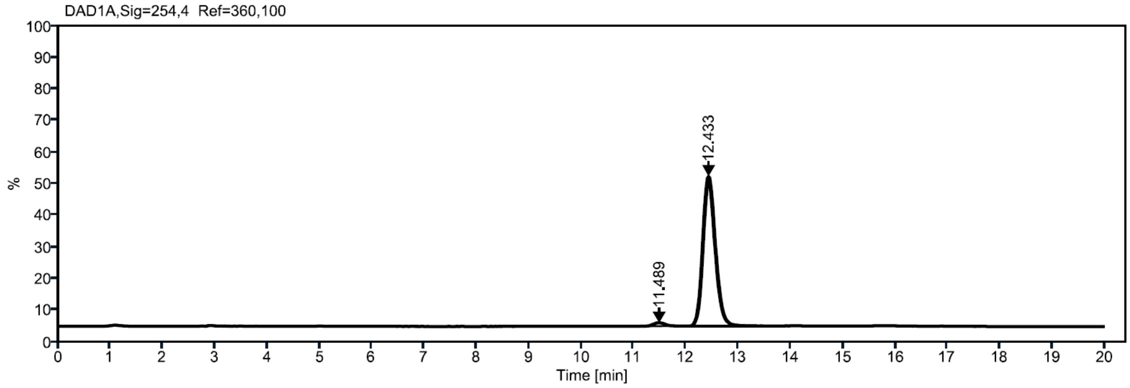

Signal: DAD1A,Sig=254,4 Ref=360,100

RT [min]	Type	Width [min]	Area	Height	Area%
11.489	MM m	0.22	28.78	1.89	2.09
12.433	MM m	0.25	1345.12	81.99	97.91
		Sum	1373.91		

Fig. 3, Compound 3ai

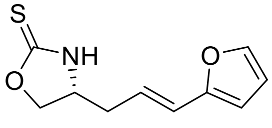

HPLC Conditions

Column: Chiralpak AD-H, Daicel Chemical Industries, Ltd.

Eluent: Hexanes/Isopropanol (90:10)

Flow rate: 1.0 mL/min

Detection: UV 254 nm

Racemic

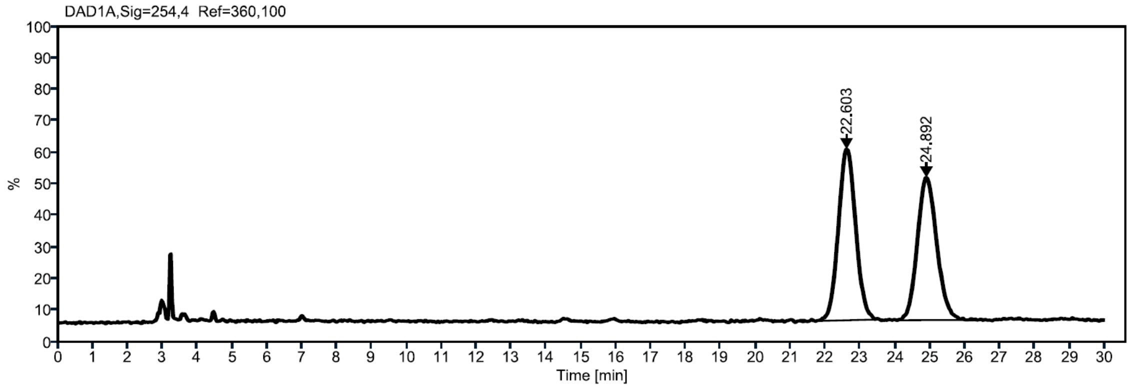

Signal: DAD1A,Sig=254,4 Ref=360,100

RT [min]	Type	Width [min]	Area	Height	Area%
22.603	MM m	0.44	216.87	6.28	51.73
24.892	MM m	0.48	202.38	5.23	48.27
		Sum	419.25		

Chiral

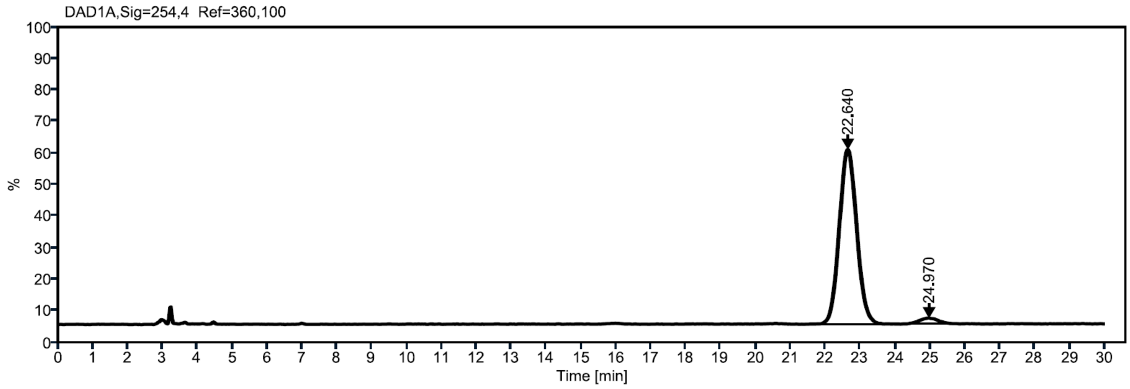

Signal: DAD1A,Sig=254,4 Ref=360,100

RT [min]	Type	Width [min]	Area	Height	Area%
22.640	MM m	0.52	1073.98	30.99	96.75
24.970	MM m	0.44	36.05	0.99	3.25
		Sum	1110.03		

Fig. 3, Compound 3aj

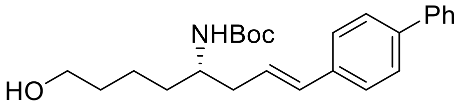

HPLC Conditions

Column: Chiralcel OJ-H, Daicel Chemical Industries, Ltd.

Eluent: Hexanes/Isopropanol (90:10)

Flow rate: 1.0 mL/min

Detection: UV 254 nm

Racemic

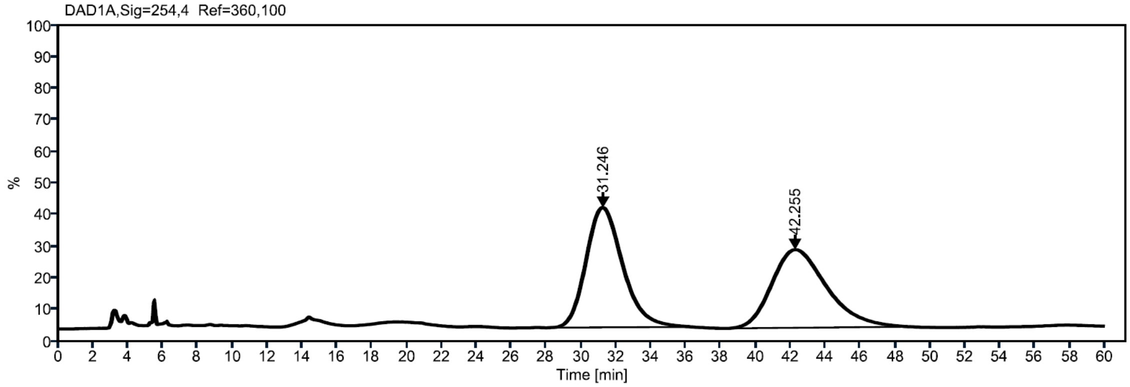

Signal: DAD1A,Sig=254,4 Ref=360,100

RT [min]	Type	Width [min]	Area	Height	Area%
31.246	MM m	1.73	2157.83	14.71	50.72
42.255	MM m	2.56	2096.85	9.58	49.28
		Sum	4254.68		

Chiral

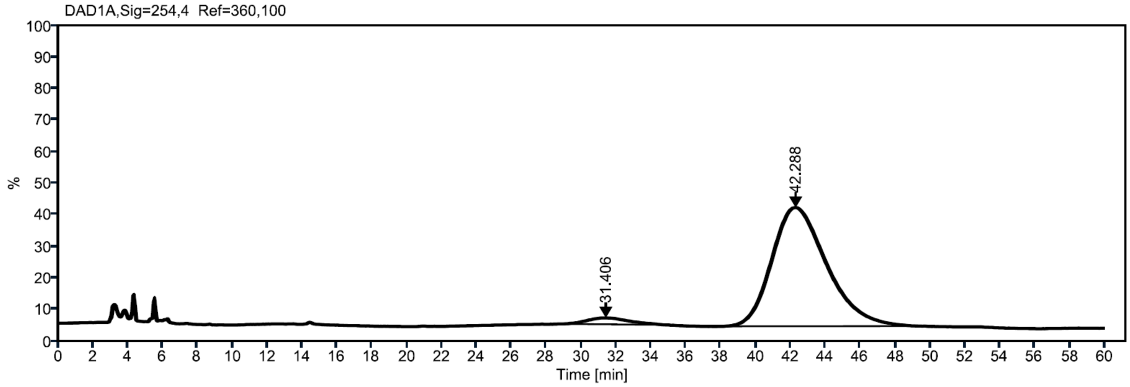

Signal: DAD1A,Sig=254,4 Ref=360,100

RT [min]	Type	Width [min]	Area	Height	Area%
31.406	MM m	1.94	111.29	0.67	4.00
42.288	MM m	2.56	2672.51	12.20	96.00
		Sum	2783.79		

Fig. 3, Compound 3ak

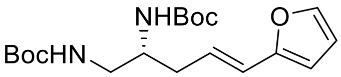

HPLC Conditions

Column: Chiralcel OD-3, Daicel Chemical Industries, Ltd.

Eluent: Hexanes/Isopropanol (95:5)

Flow rate: 1.0 mL/min

Detection: UV 254 nm

Racemic

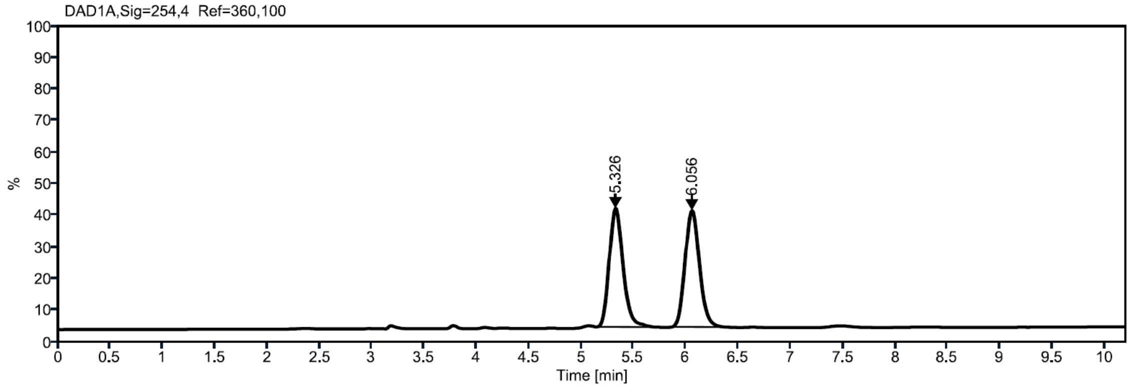

Signal: DAD1A,Sig=254,4 Ref=360,100

RT [min]	Type	Width [min]	Area	Height	Area%
5.326	MM m	0.14	354.01	38.72	49.49
6.056	MM m	0.15	361.28	38.02	50.51
		Sum	715.29		

Chiral

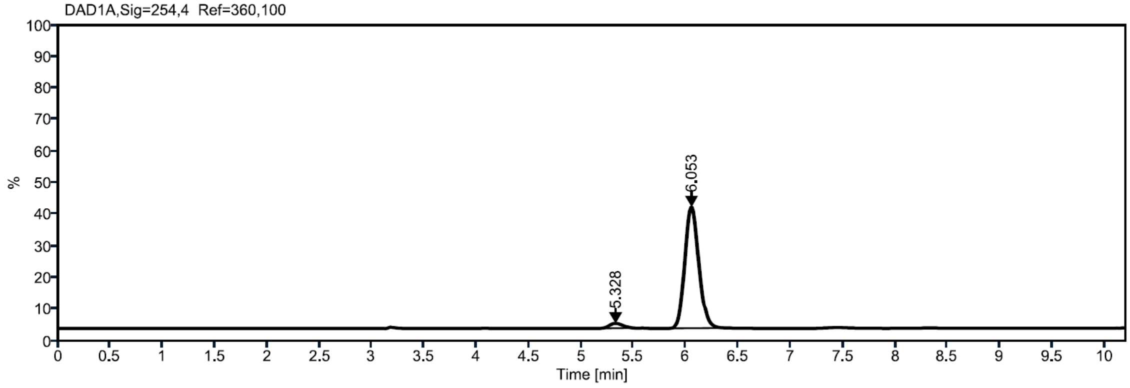

Signal: DAD1A,Sig=254,4 Ref=360,100

RT [min]	Type	Width [min]	Area	Height	Area%
5.328	MM m	0.14	34.71	3.86	3.73
6.053	MM m	0.15	896.48	95.00	96.27
		Sum	931.19		

Fig. 3, Compound 3al

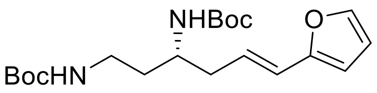

HPLC Conditions

Column: Chiralpak AD-H, Daicel Chemical Industries, Ltd.

Eluent: Hexanes/Isopropanol (95:5)

Flow rate: 0.5 mL/min

Detection: UV 254 nm

Racemic

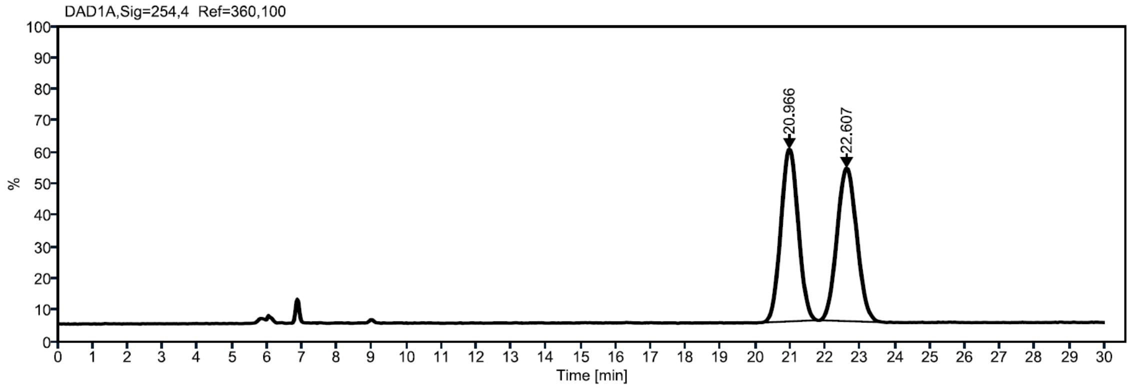

Signal:	DAD1A,Sig=254,4 Ref=360,100				
**RT [min]**	**Type**	**Width [min]**	**Area**	**Height**	**Area%**
20.966	MM m	0.53	604.23	17.40	49.72
22.607	MM m	0.59	611.00	15.44	50.28
		Sum	1215.24		

Chiral

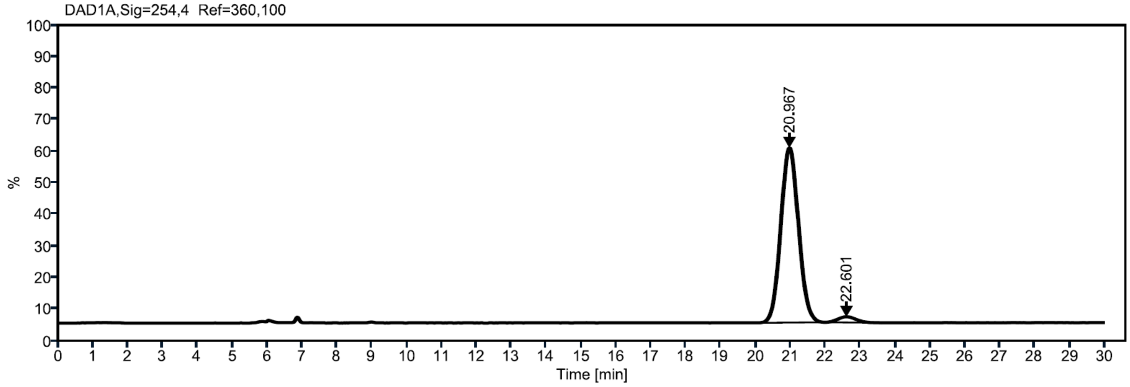

Signal:	DAD1A,Sig=254,4 Ref=360,100				
**RT [min]**	**Type**	**Width [min]**	**Area**	**Height**	**Area%**
20.967	MM m	0.56	1930.43	54.56	96.74
22.601	MM m	0.44	65.12	1.79	3.26
		Sum	1995.55		

Fig. 3, Compound 3am

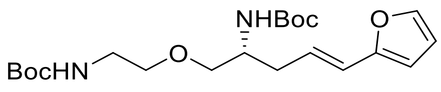

HPLC Conditions

Column: Chiralpak AS-H, Daicel Chemical Industries, Ltd.

Eluent: Hexanes/Isopropanol (90:10)

Flow rate: 0.6 mL/min

Detection: UV 254 nm

Racemic

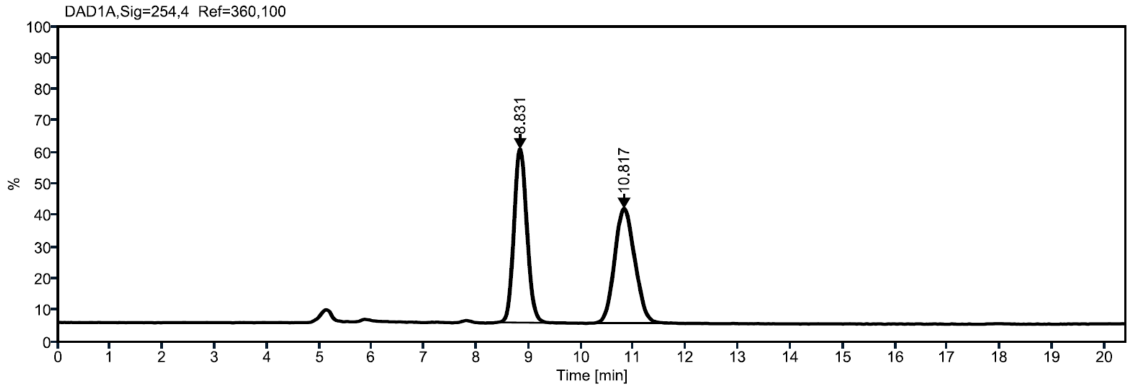

Signal: DAD1A,Sig=254,4 Ref=360,100

RT [min]	Type	Width [min]	Area	Height	Area%
8.831	MM m	0.27	676.59	39.39	49.18
10.817	MM m	0.41	699.21	26.01	50.82
		Sum	1375.80		

Chiral

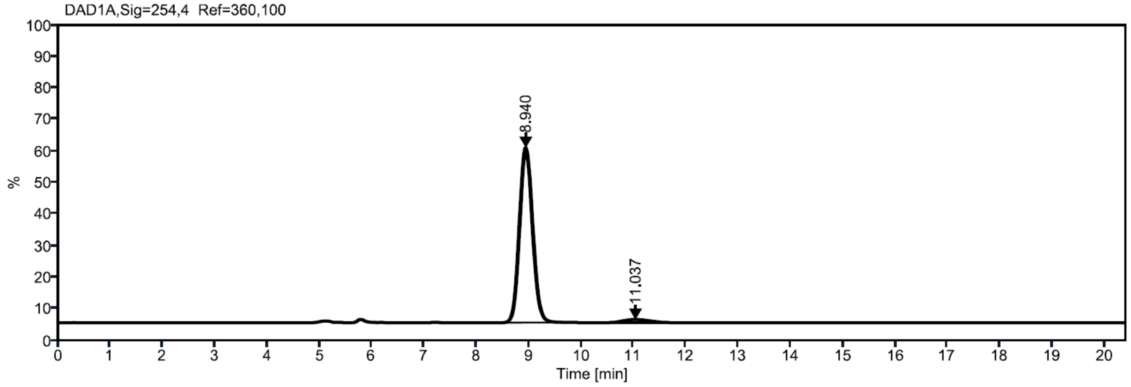

Signal: DAD1A,Sig=254,4 Ref=360,100

RT [min]	Type	Width [min]	Area	Height	Area%
8.940	MM m	0.27	5023.25	289.72	96.50
11.037	MM m	0.43	181.93	5.25	3.50
		Sum	5205.19		

Fig. 3, Compound 3an

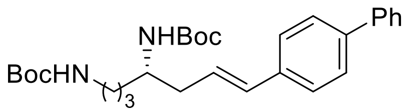

HPLC Conditions

Column: Chiralpak AD-H, Daicel Chemical Industries, Ltd.

Eluent: Hexanes/Isopropanol (95:5)

Flow rate: 1.0 mL/min

Detection: UV 254 nm

Racemic

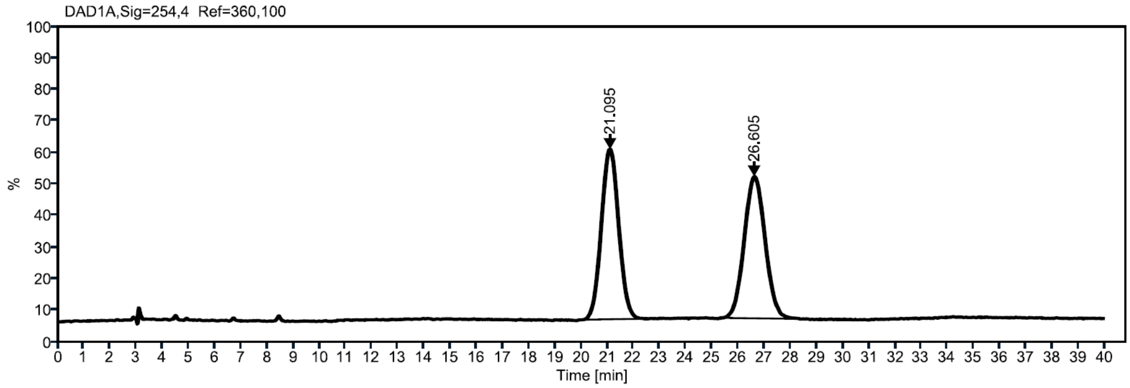

Signal: DAD1A,Sig=254,4 Ref=360,100

RT [min]	Type	Width [min]	Area	Height	Area%
21.095	MM m	0.56	281.68	6.09	49.87
26.605	MM m	0.67	283.11	5.08	50.13
		Sum	564.78		

Chiral

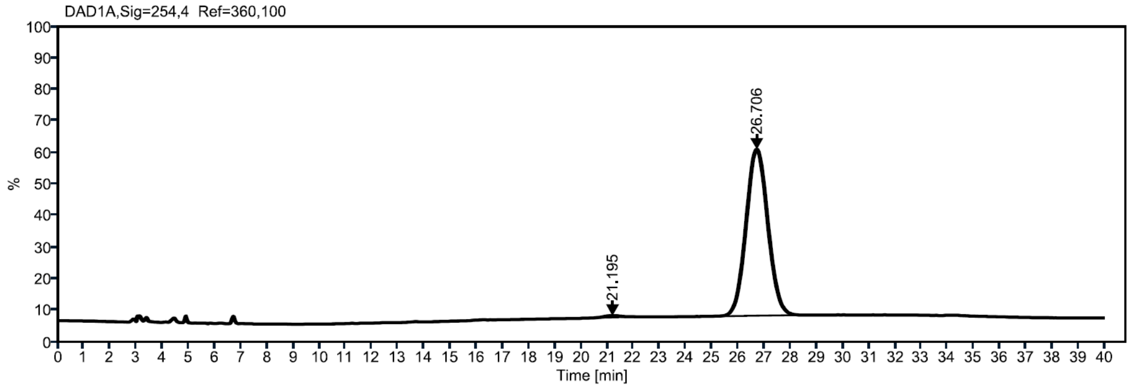

Signal: DAD1A,Sig=254,4 Ref=360,100

RT [min]	Type	Width [min]	Area	Height	Area%
21.195	MM m	0.53	14.26	0.32	0.85
26.706	MM m	0.89	1656.90	28.55	99.15
		Sum	1671.16		

Fig. 3, Compound 3ao

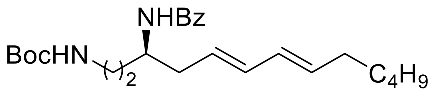

HPLC Conditions

Column: Chiralpak AD-H, Daicel Chemical Industries, Ltd.

Eluent: Hexanes/Isopropanol (95:5)

Flow rate: 1.0 mL/min

Detection: UV 254 nm

Racemic

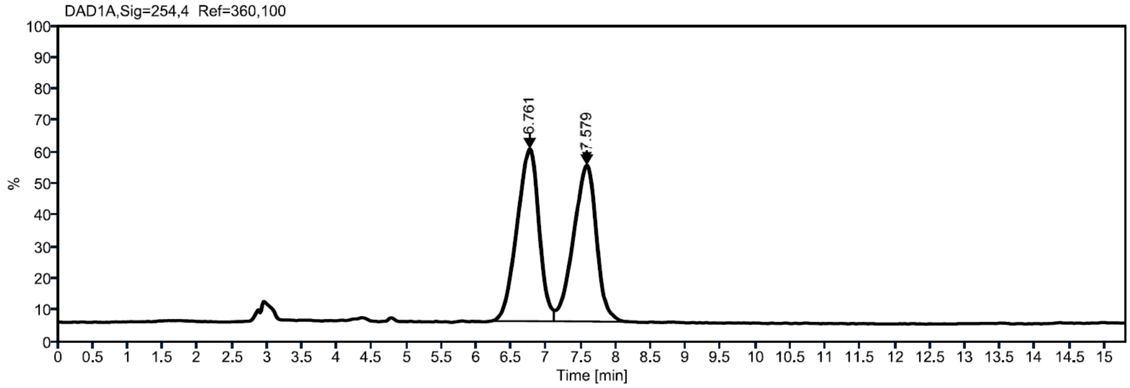

Signal: DAD1A,Sig=254,4 Ref=360,100

RT [min]	Type	Width [min]	Area	Height	Area%
6.761	MM m	0.33	413.65	18.87	51.20
7.579	MM m	0.34	394.23	17.10	48.80
		Sum	807.88		

Chiral

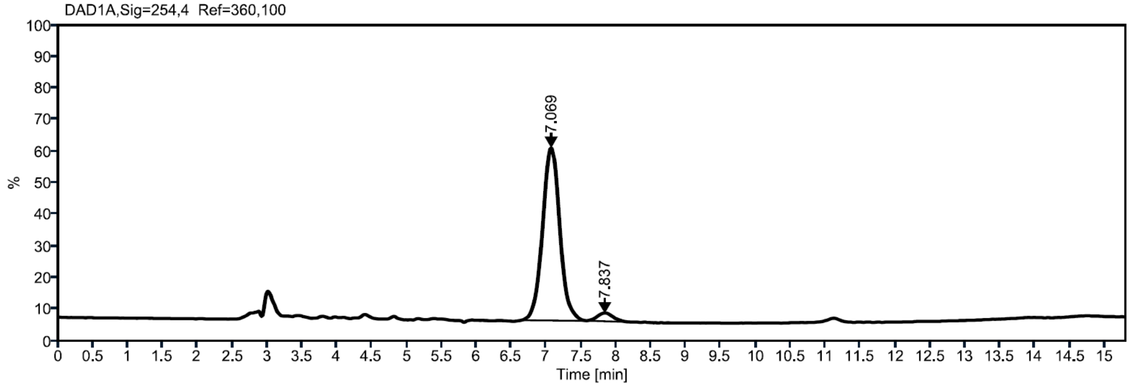

Signal: DAD1A,Sig=254,4 Ref=360,100

RT [min]	Type	Width [min]	Area	Height	Area%
7.069	MM m	0.26	108.10	6.30	95.91
7.837	MM m	0.22	4.61	0.30	4.09
		Sum	112.71		

Fig. 3, Compound 3ap

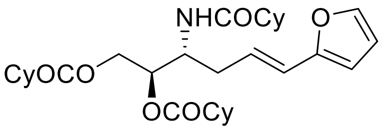

HPLC Conditions

Column: Chiralcel OD-3, Daicel Chemical Industries, Ltd.

Eluent: Hexanes/Isopropanol (95:5)

Flow rate: 0.5 mL/min

Detection: UV 254 nm

Reaction with racemic catalyst

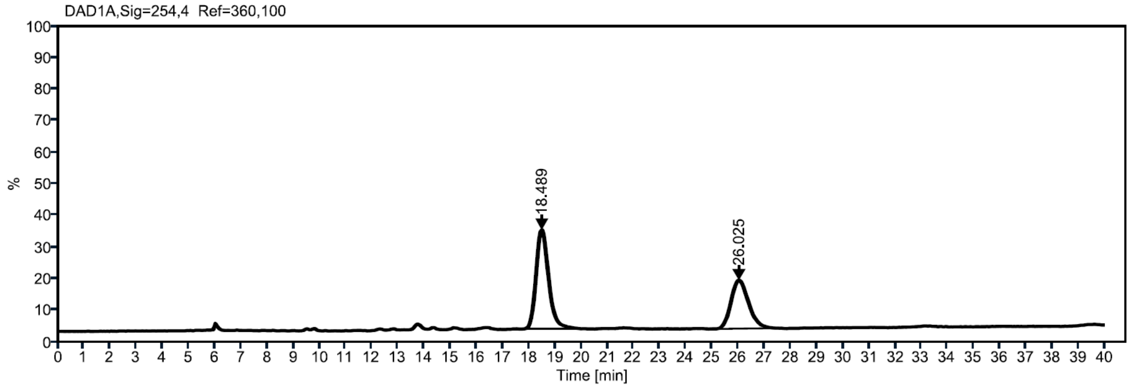

Signal: DAD1A,Sig=254,4 Ref=360,100

RT [min]	Type	Width [min]	Area	Height	Area%
18.489	MM m	0.49	478.86	14.40	59.29
26.025	MM m	0.59	328.73	7.06	40.71
		Sum	807.59		

Reaction with chiral catalyst

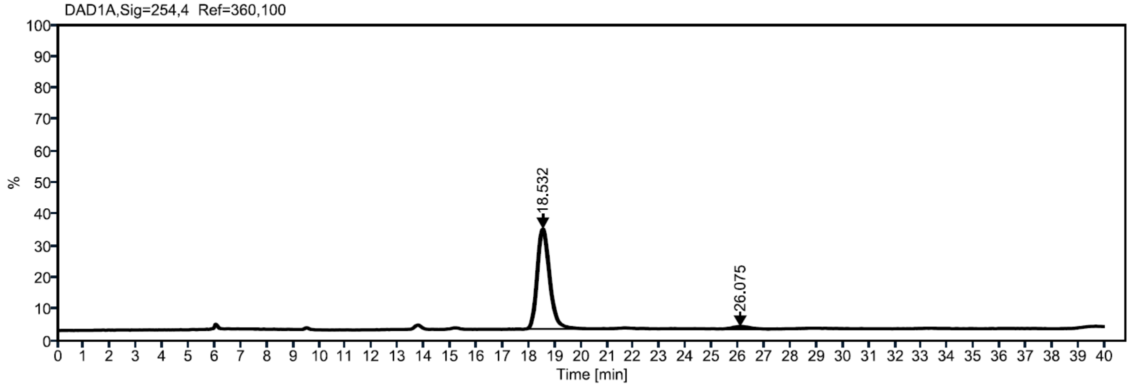

Signal: DAD1A,Sig=254,4 Ref=360,100

RT [min]	Type	Width [min]	Area	Height	Area%
18.532	MM m	0.50	736.42	22.36	96.71
26.075	MM m	0.51	25.07	0.58	3.29
		Sum	761.50		

Fig. 3, Compound 3aq

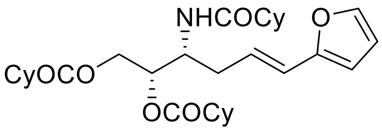

HPLC Conditions

Column: Chiralcel OD-3, Daicel Chemical Industries, Ltd.

Eluent: Hexanes/Isopropanol (95:5)

Flow rate: 0.5 mL/min

Detection: UV 254 nm

Reaction with racemic catalyst

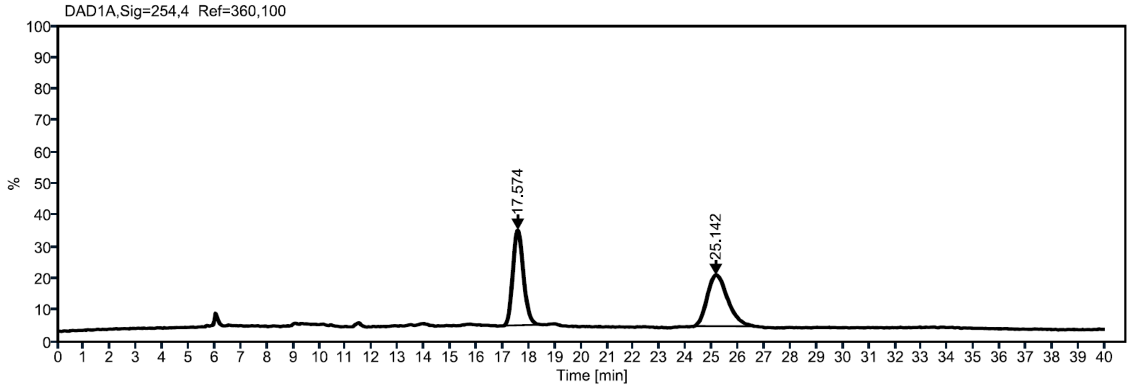

Signal: DAD1A,Sig=254,4 Ref=360,100

RT [min]	Type	Width [min]	Area	Height	Area%
17.574	MM m	0.41	183.49	6.50	49.16
25.142	MM m	0.64	189.74	3.50	50.84
		Sum	373.23		

Reaction with chiral catalyst

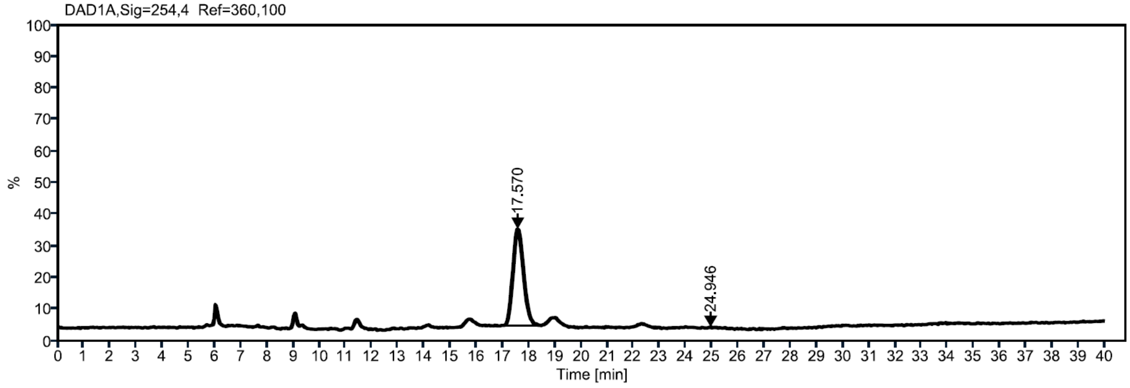

Signal: DAD1A,Sig=254,4 Ref=360,100

RT [min]	Type	Width [min]	Area	Height	Area%
17.570	MM m	0.36	111.53	3.93	98.94
24.946	MM m	0.26	1.19	0.06	1.06
		Sum	112.72		

Fig. 3, Compound 3ar

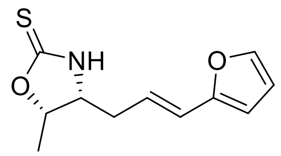

HPLC Conditions

Column: Chiralpak AD-H, Daicel Chemical Industries, Ltd.

Eluent: Hexanes/Isopropanol (95:5)

Flow rate: 1.0 mL/min

Detection: UV 254 nm

Reaction with racemic catalyst

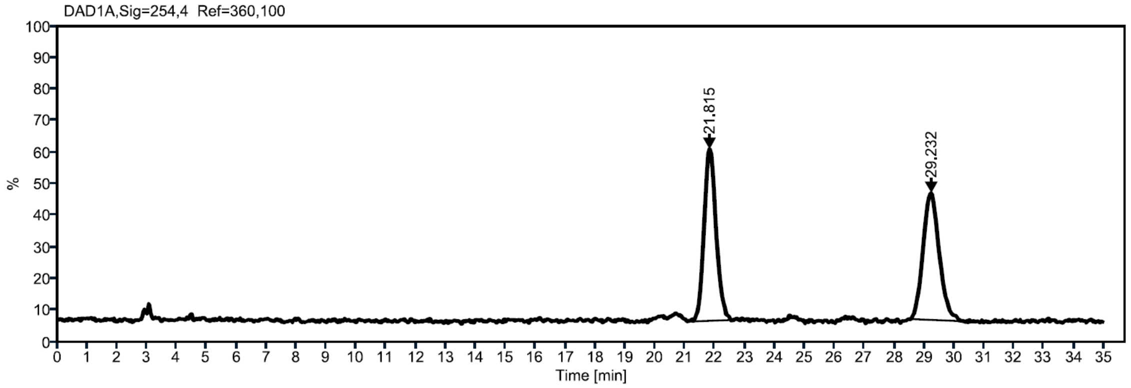

Signal: DAD1A,Sig=254,4 Ref=360,100

RT [min]	Type	Width [min]	Area	Height	Area%
21.815	MM m	0.36	166.62	5.93	50.22
29.232	MM m	0.45	165.18	4.39	49.78
		Sum	331.80		

Reaction with chiral catalyst

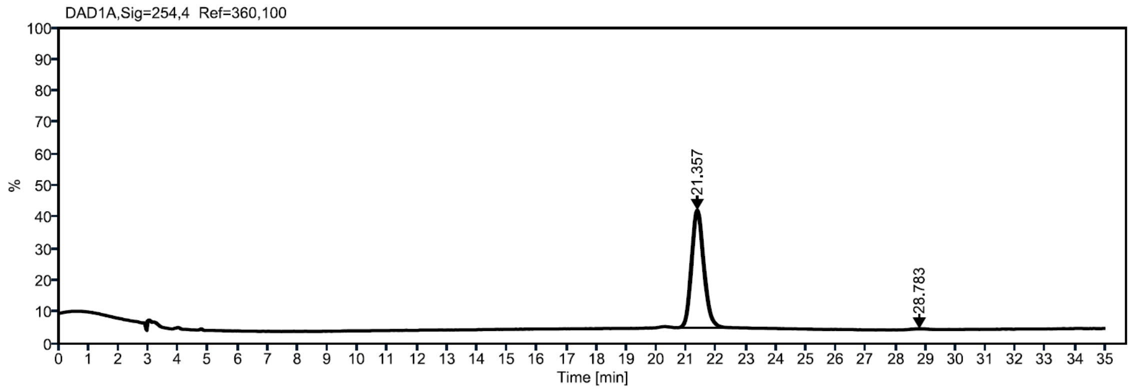

Signal: DAD1A,Sig=254,4 Ref=360,100

RT [min]	Type	Width [min]	Area	Height	Area%
21.357	MM m	0.43	293.84	10.26	98.78
28.783	MM m	0.42	3.62	0.10	1.22
		Sum	297.46		

Fig. 3, Compound 3as

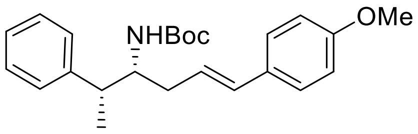

HPLC Conditions

Column: Chiralcel OJ-H, Daicel Chemical Industries, Ltd.

Eluent: Hexanes/Isopropanol (90:10)

Flow rate: 0.7 mL/min

Detection: UV 254 nm

Reaction with racemic catalyst

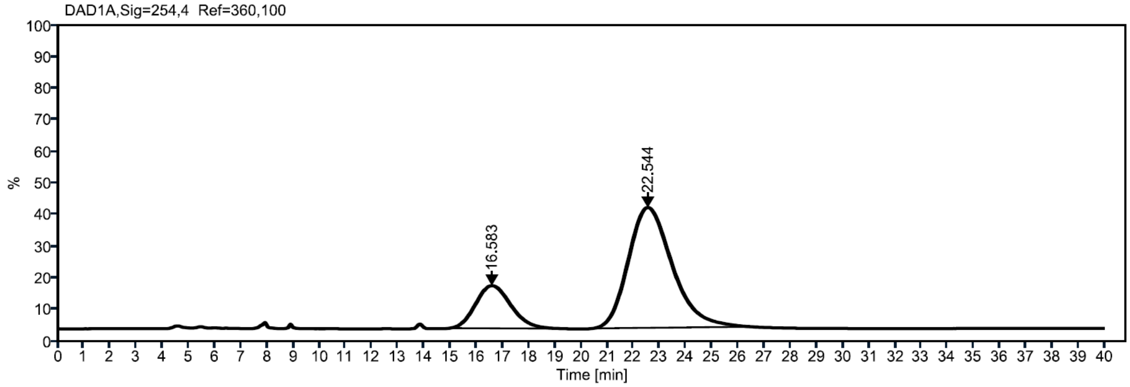

Signal: DAD1A,Sig=254,4 Ref=360,100

RT [min]	Type	Width [min]	Area	Height	Area%
16.583	MM m	1.26	1981.00	21.14	22.13
22.544	MM m	1.65	6971.70	59.49	77.87
		Sum	8952.71		

Reaction with chiral catalyst

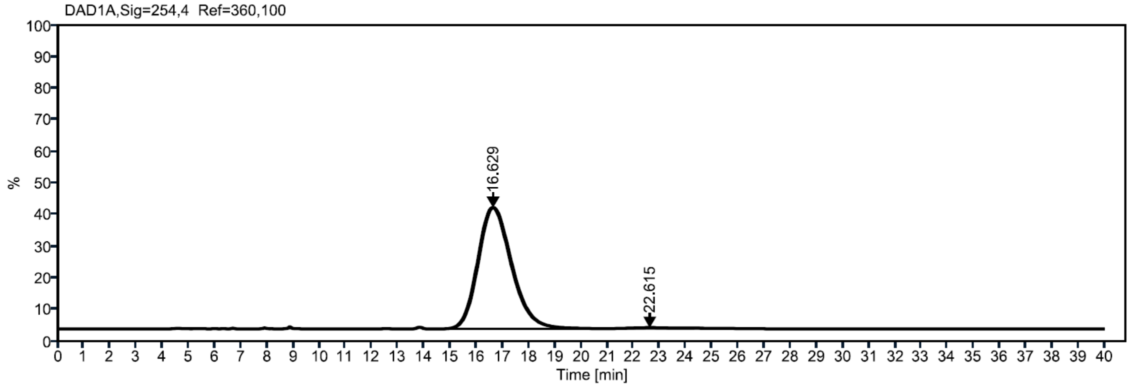

Signal: DAD1A,Sig=254,4 Ref=360,100

RT [min]	Type	Width [min]	Area	Height	Area%
16.629	BM m	1.38	28123.90	312.56	98.59
22.615	MM m	1.66	401.98	2.84	1.41
		Sum	28525.89		

Fig. 3, Compound 3at

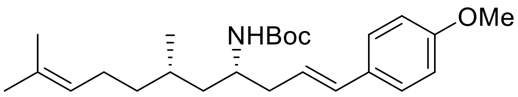

HPLC Conditions

Column: Chiralpak AD-H,

Daicel Chemical Industries, Ltd.

Eluent: Hexanes/Isopropanol (95:5)

Flow rate: 1.0 mL/min

Reaction with racemic catalyst

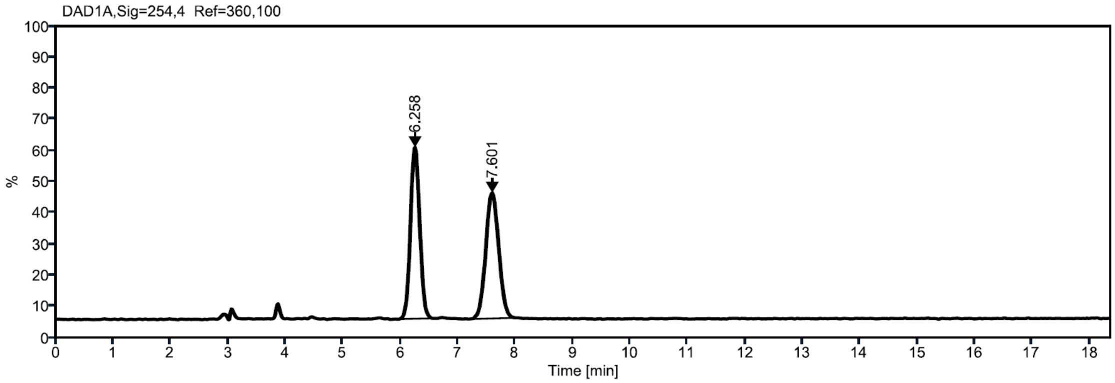

Signal: DAD1A,Sig=254,4 Ref=360,100

RT [min]	Type	Width [min]	Area	Height	Area%
6.258	MM m	0.17	108.11	9.65	49.28
7.601	MM m	0.25	111.25	7.08	50.72
		Sum	219.36		

Reaction with chiral catalyst

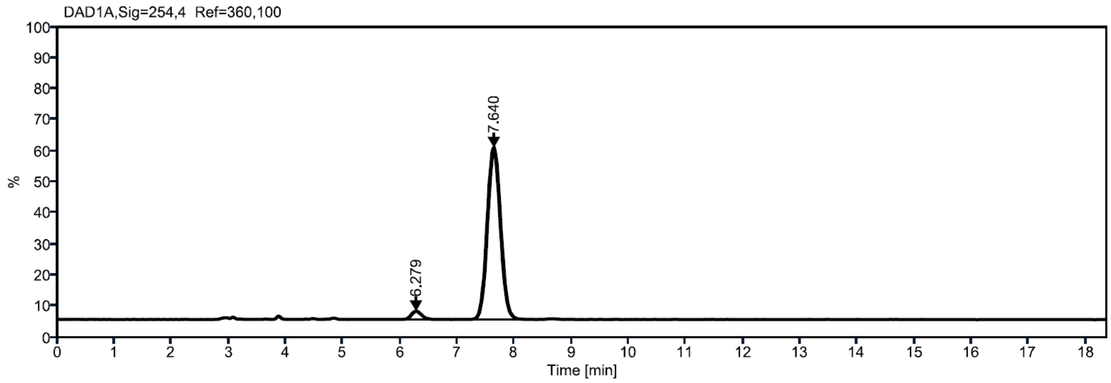

Signal: DAD1A,Sig=254,4 Ref=360,100

RT [min]	Type	Width [min]	Area	Height	Area%
6.279	MM m	0.18	31.03	2.56	3.49
7.640	MM m	0.25	857.58	53.61	96.51
		Sum	888.61		

Fig. 3, Compound 3au

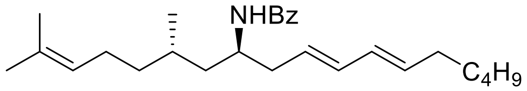

HPLC Conditions

Column: Chiralpak AD-H,
Daicel Chemical Industries, Ltd.

Eluent: Hexanes/Isopropanol (97:3)

Flow rate: 1.0 mL/min

Detection: UV 230 nm

Reaction with racemic catalyst

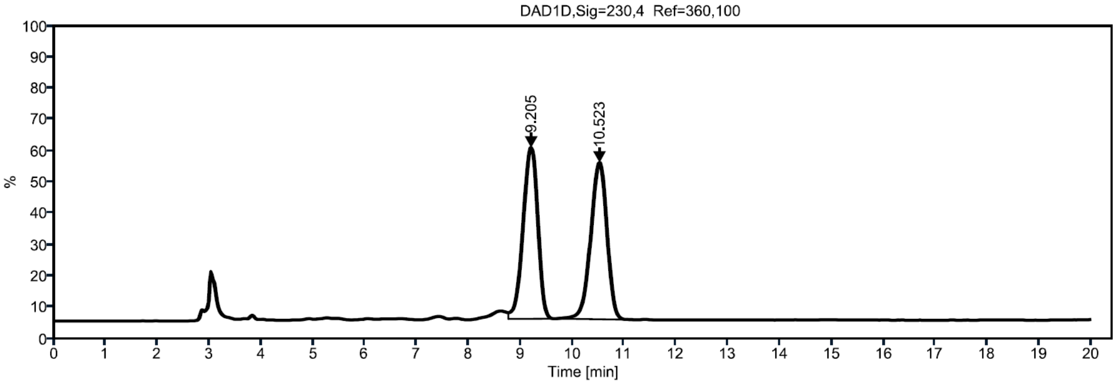

Signal: DAD1D,Sig=230,4 Ref=360,100

RT [min]	Type	Width [min]	Area	Height	Area%
9.205	MM m	0.31	723.28	36.71	49.69
10.523	MM m	0.34	732.28	33.59	50.31
		Sum	1455.57		

Reaction with chiral catalyst

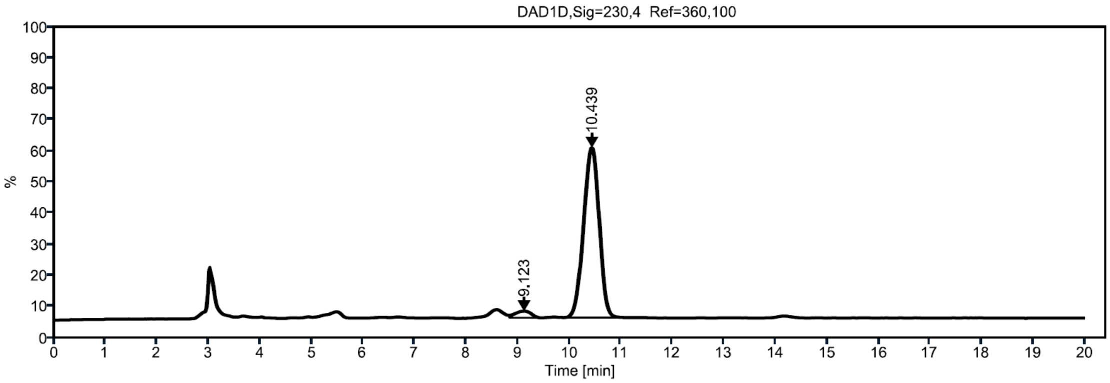

Signal: DAD1D,Sig=230,4 Ref=360,100

RT [min]	Type	Width [min]	Area	Height	Area%
9.123	MM m	0.33	30.23	1.39	3.83
10.439	MM m	0.33	759.44	36.12	96.17
		Sum	789.68		

Fig. 3, Compound 3av

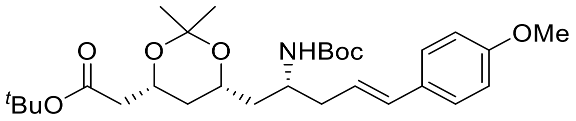

HPLC Conditions

Column: Chiralpak AD-H,
Daicel Chemical Industries, Ltd.

Eluent: Hexanes/Isopropanol (95:5)

Flow rate: 1.0 mL/min

Detection: UV 254 nm

Reaction with racemic catalyst

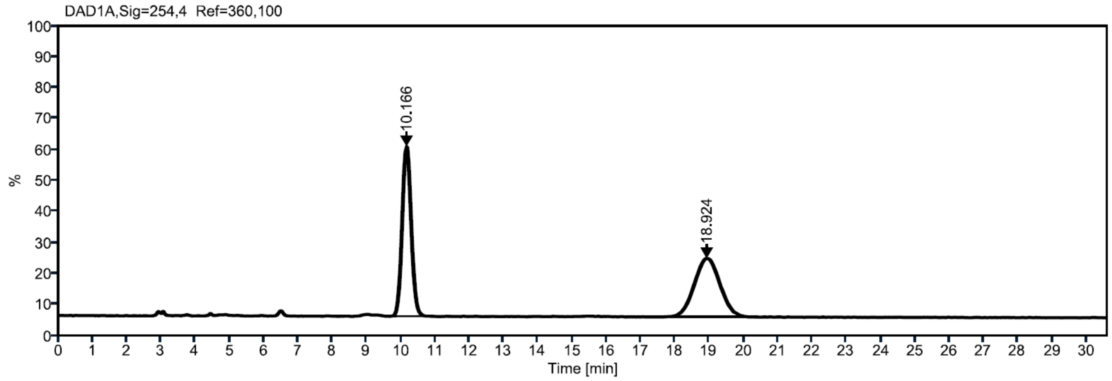

Signal: DAD1A,Sig=254,4 Ref=360,100

RT [min]	Type	Width [min]	Area	Height	Area%
10.166	MM m	0.29	594.97	32.48	50.62
18.924	MM m	0.62	580.40	11.16	49.38
		Sum	1175.37		

Reaction with chiral catalyst

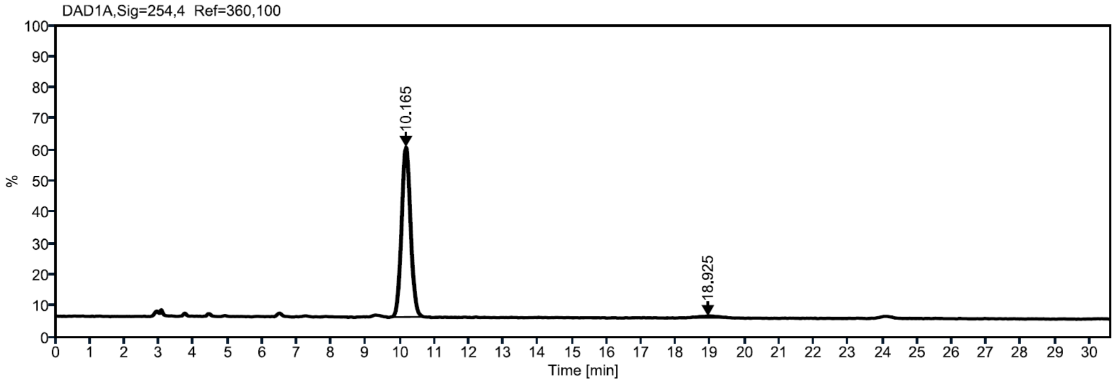

Signal: DAD1A,Sig=254,4 Ref=360,100

RT [min]	Type	Width [min]	Area	Height	Area%
10.165	MM m	0.29	398.65	21.81	97.52
18.925	MM m	0.49	10.15	0.25	2.48
		Sum	408.80		

Fig. 3, Compound 3aw

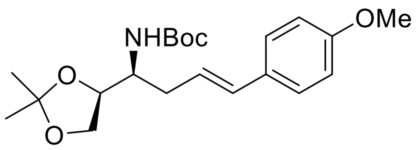

HPLC Conditions

Column: Chiralpak AD-H, Daicel Chemical Industries, Ltd.

Eluent: Hexanes/Isopropanol (95:5)

Flow rate: 1.0 mL/min

Detection: UV 254 nm

Reaction with racemic catalyst

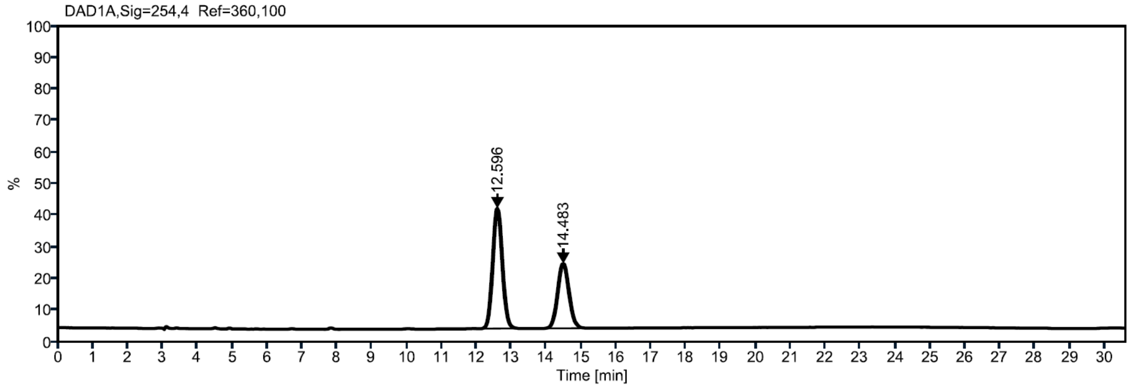

Signal: DAD1A,Sig=254,4 Ref=360,100

RT [min]	Type	Width [min]	Area	Height	Area%
12.596	MM m	0.30	500.32	26.15	61.01
14.483	MM m	0.35	319.68	14.06	38.99
		Sum	820.00		

Reaction with chiral catalyst

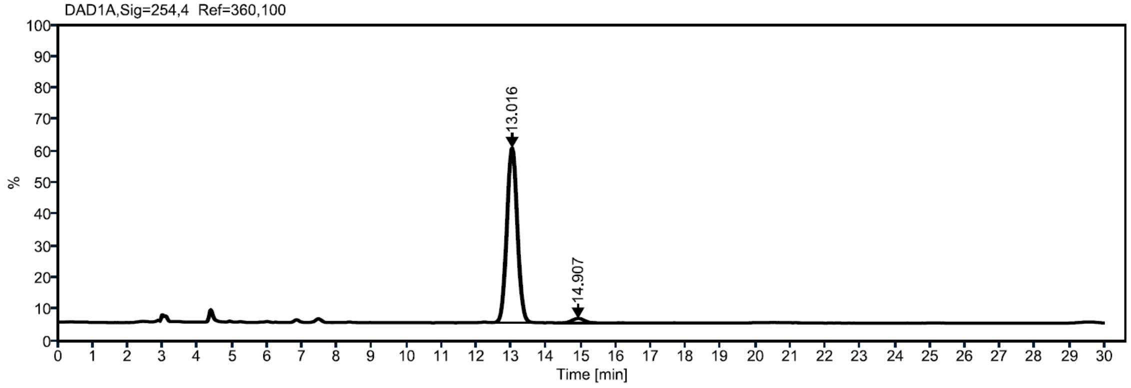

Signal: DAD1A,Sig=254,4 Ref=360,100

RT [min]	Type	Width [min]	Area	Height	Area%
13.016	MM m	0.33	473.90	22.65	97.16
14.907	MM m	0.30	13.87	0.57	2.84
		Sum	487.78		

Fig. 3, Compound 3ax

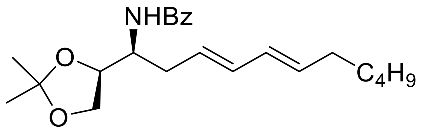

HPLC Conditions

Column: Chiralpak AD-H, Daicel Chemical Industries, Ltd.

Eluent: Hexanes/Isopropanol (97:3)

Flow rate: 1.0 mL/min

Detection: UV 230 nm

Reaction with racemic catalyst

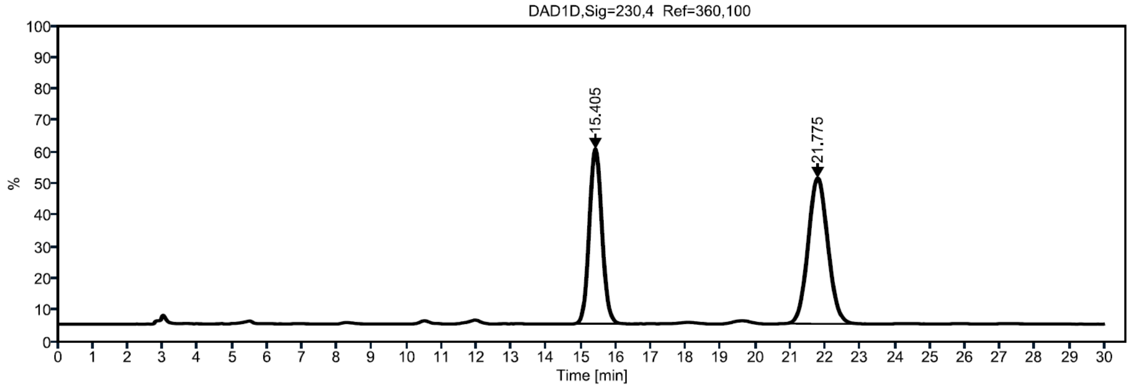

Signal: DAD1D,Sig=230,4 Ref=360,100

RT [min]	Type	Width [min]	Area	Height	Area%
15.405	MM m	0.39	2738.55	108.61	43.50
21.775	MM m	0.60	3556.76	90.41	56.50
		Sum	6295.31		

Reaction with chiral catalyst

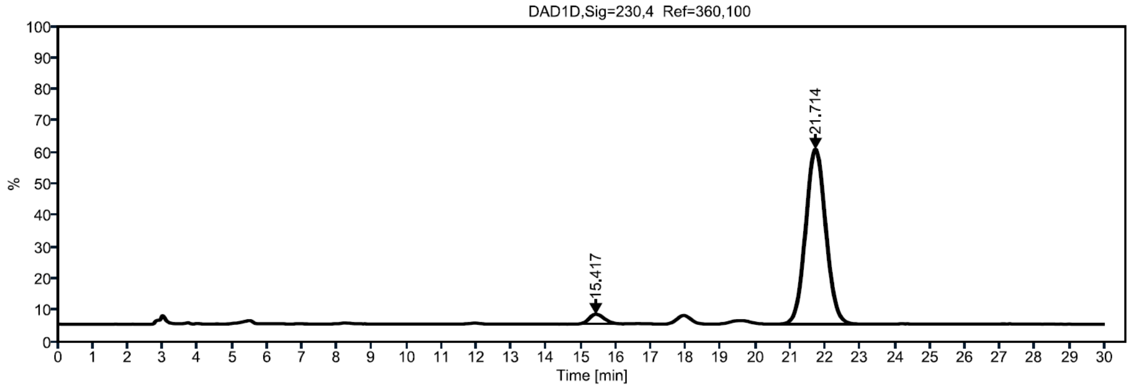

Signal: DAD1D,Sig=230,4 Ref=360,100

RT [min]	Type	Width [min]	Area	Height	Area%
15.417	MM m	0.42	206.65	6.94	3.93
21.714	MM m	0.62	5052.65	125.74	96.07
		Sum	5259.30		

Fig. 3, Compound 3ay

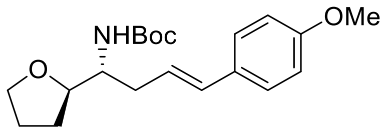

HPLC Conditions

Column: Chiralpak AS-H, Daicel Chemical Industries, Ltd.

Eluent: Hexanes/Isopropanol (90:10)

Flow rate: 0.6 mL/min

Detection: UV 254 nm

Reaction with racemic catalyst

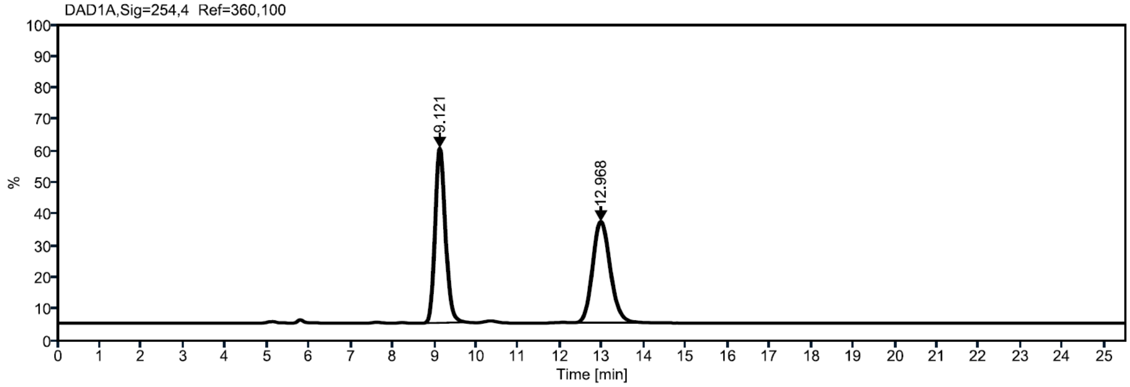

Signal: DAD1A,Sig=254,4 Ref=360,100

RT [min]	Type	Width [min]	Area	Height	Area%
9.121	MM m	0.27	4673.29	270.97	50.49
12.968	MM m	0.46	4582.61	156.47	49.51
		Sum	9255.90		

Reaction with chiral catalyst

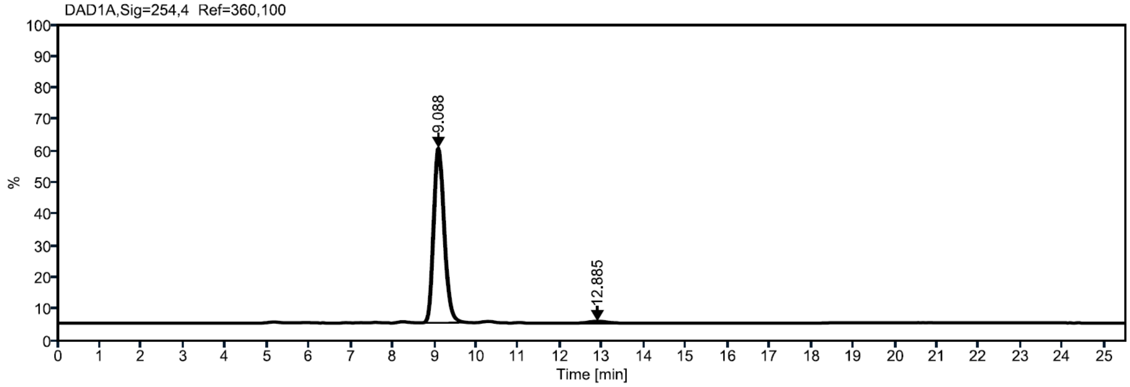

Signal: DAD1A,Sig=254,4 Ref=360,100

RT [min]	Type	Width [min]	Area	Height	Area%
9.088	MM m	0.27	4017.86	227.58	98.21
12.885	MM m	0.36	73.15	2.55	1.79
		Sum	4091.01		

Fig. 3, Compound 3az

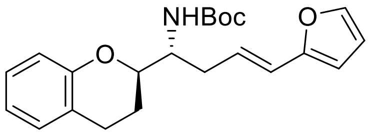

HPLC Conditions

Column: Chiralpak AD-H, Daicel Chemical Industries, Ltd.

Eluent: Hexanes/Isopropanol (95:5)

Flow rate: 1.0 mL/min

Detection: UV 254 nm

Reaction with racemic catalyst

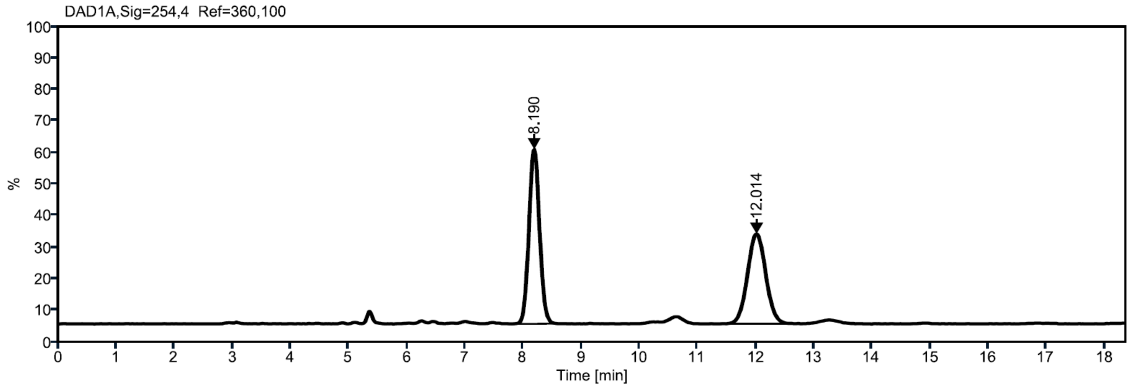

Signal: DAD1A,Sig=254,4 Ref=360,100

RT [min]	Type	Width [min]	Area	Height	Area%
8.190	MM m	0.20	781.14	61.45	53.09
12.014	MM m	0.34	690.14	31.62	46.91
		Sum	1471.28		

Reaction with chiral catalyst

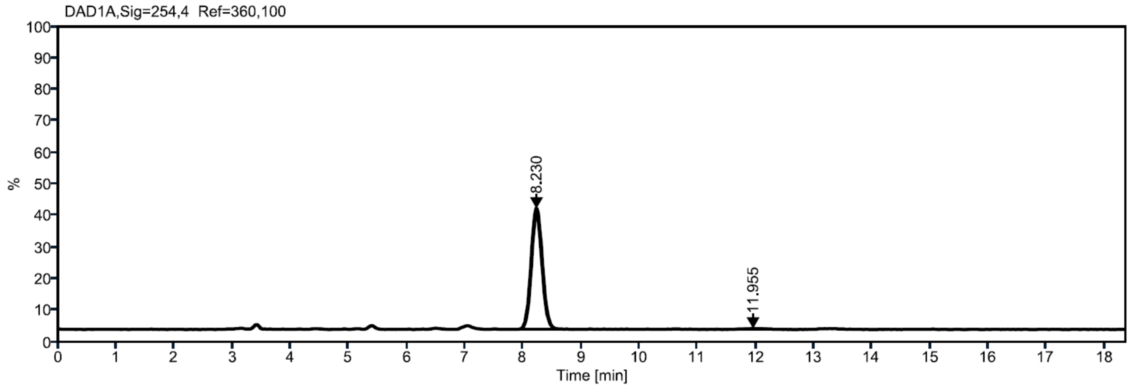

Signal: DAD1A,Sig=254,4 Ref=360,100

RT [min]	Type	Width [min]	Area	Height	Area%
8.230	MM m	0.20	2295.87	176.13	98.58
11.955	MM m	0.26	33.05	1.56	1.42
		Sum	2328.91		

Fig. 3, Compound 3ba

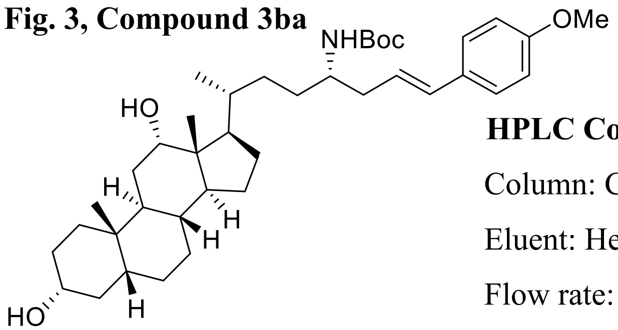

HPLC Conditions

Column: Chiralpak AD-H, Daicel Chemical Industries, Ltd.

Eluent: Hexanes/Isopropanol (80:20)

Flow rate: 1.0 mL/min; Detection: UV 254 nm

Reaction with racemic catalyst

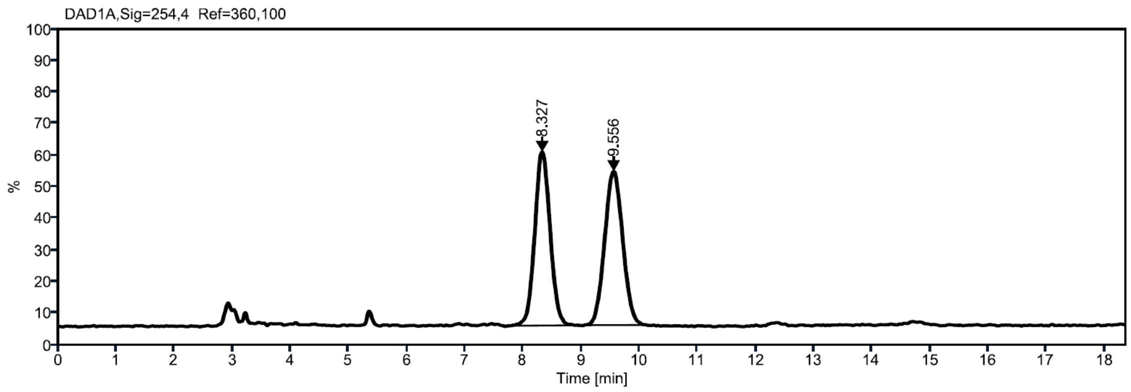

Signal: DAD1A,Sig=254,4 Ref=360,100

RT [min]	Type	Width [min]	Area	Height	Area%
8.327	MM m	0.28	163.02	8.86	49.00
9.556	MM m	0.33	169.68	7.85	51.00
		Sum	332.69		

Reaction with chiral catalyst

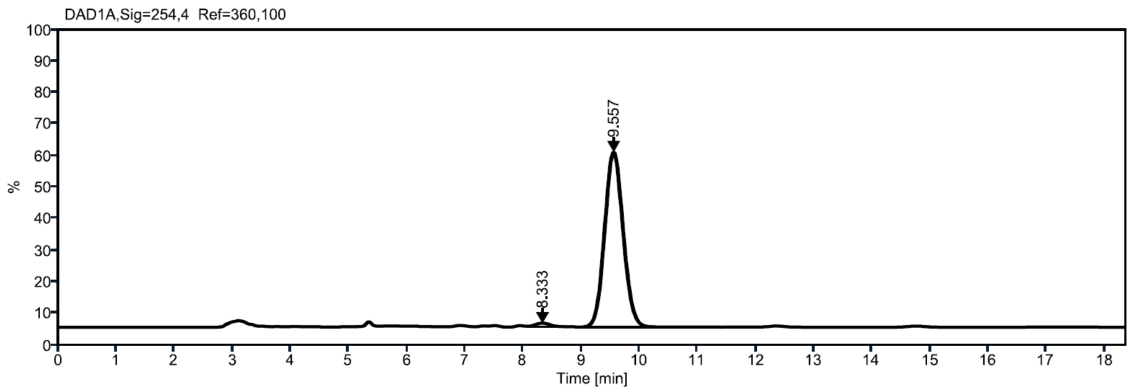

Signal: DAD1A,Sig=254,4 Ref=360,100

RT [min]	Type	Width [min]	Area	Height	Area%
8.333	MM m	0.26	76.48	4.42	1.60
9.557	MM m	0.35	4694.63	212.19	98.40
		Sum	4771.11		

Fig. 4, Compound 10

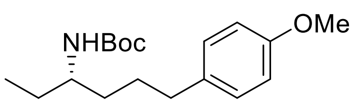

HPLC Conditions

Column: Chiralpak AD-H, Daicel Chemical Industries, Ltd.

Eluent: Hexanes/Isopropanol (98:2)

Flow rate: 1.0 mL/min

Detection: UV 230 nm

Racemic

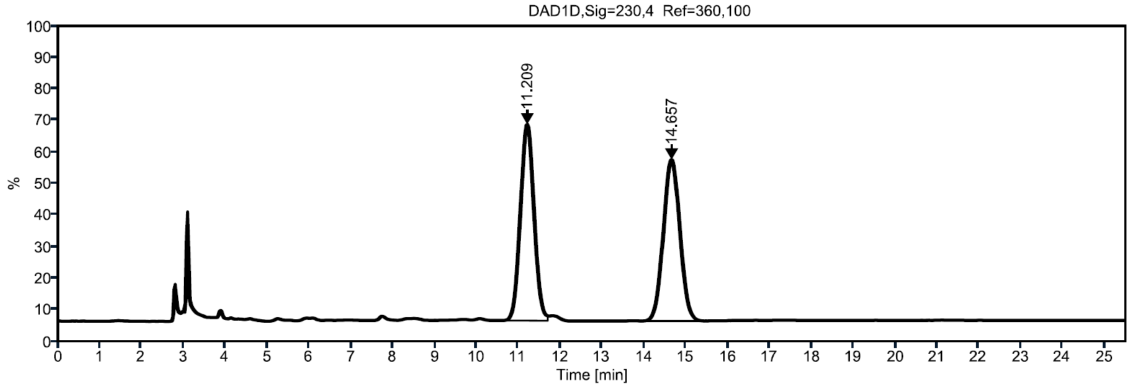

Signal: DAD1D,Sig=230,4 Ref=360,100

RT [min]	Type	Width [min]	Area	Height	Area%
11.209	MM m	0.35	961.14	42.39	50.02
14.657	MM m	0.43	960.28	34.87	49.98
		Sum	1921.41		

Chiral

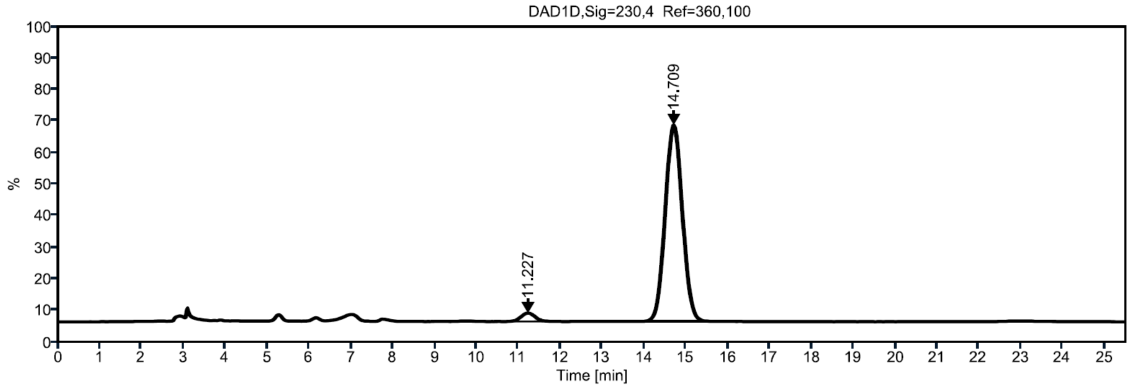

Signal: DAD1D,Sig=230,4 Ref=360,100

RT [min]	Type	Width [min]	Area	Height	Area%
11.227	MM m	0.35	116.01	4.98	3.36
14.709	MM m	0.45	3332.50	116.58	96.64
		Sum	3448.51		

Fig. 4, Compound (2*S*,3*R*,5*S*)-11

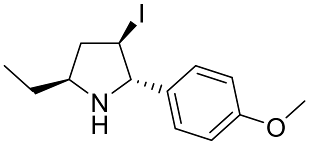

HPLC Conditions

Column: Chiralpak AD-H, Daicel Chemical Industries, Ltd.

Eluent: Hexanes/Isopropanol (97:3)

Flow rate: 1.0 mL/min

Detection: UV 230 nm

Cyclization of racemic substrate

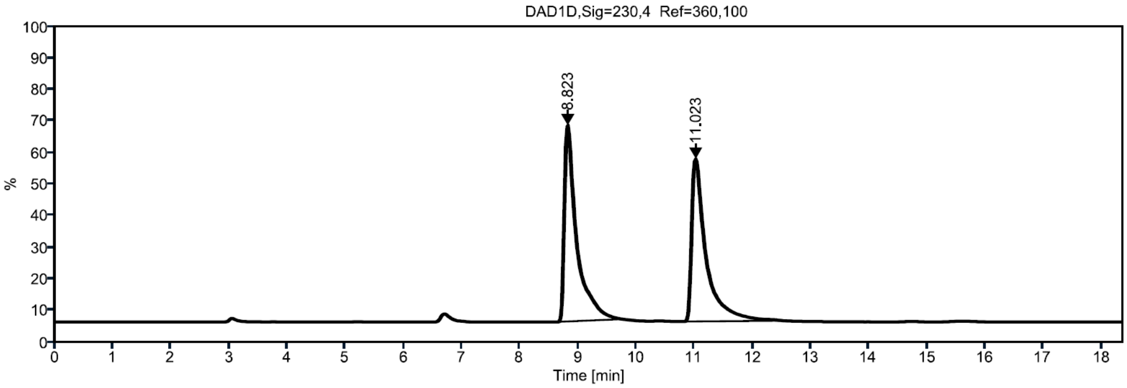

Signal: DAD1D,Sig=230,4 Ref=360,100

RT [min]	Type	Width [min]	Area	Height	Area%
8.823	MM m	0.21	4996.70	341.35	50.57
11.023	MM m	0.25	4884.31	282.96	49.43
		Sum	9881.01		

Cyclization of chiral substrate

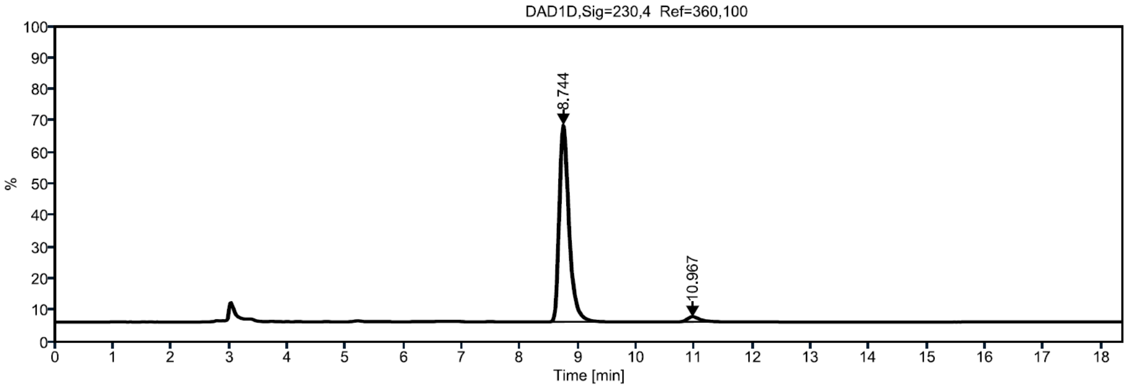

Signal: DAD1D,Sig=230,4 Ref=360,100

RT [min]	Type	Width [min]	Area	Height	Area%
8.744	MM m	0.18	3458.70	298.22	96.80
10.967	MM m	0.22	114.22	7.91	3.20
		Sum	3572.92		

Fig. 4, Compound (2*R*,3*S*,5*S*)-11

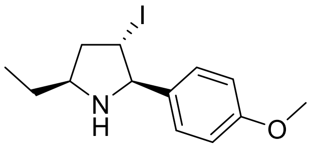

HPLC Conditions

Column: Chiralpak AD-H, Daicel Chemical Industries, Ltd.

Eluent: Hexanes/Isopropanol (97:3)

Flow rate: 1.0 mL/min

Detection: UV 230 nm

Cyclization of racemic substrate

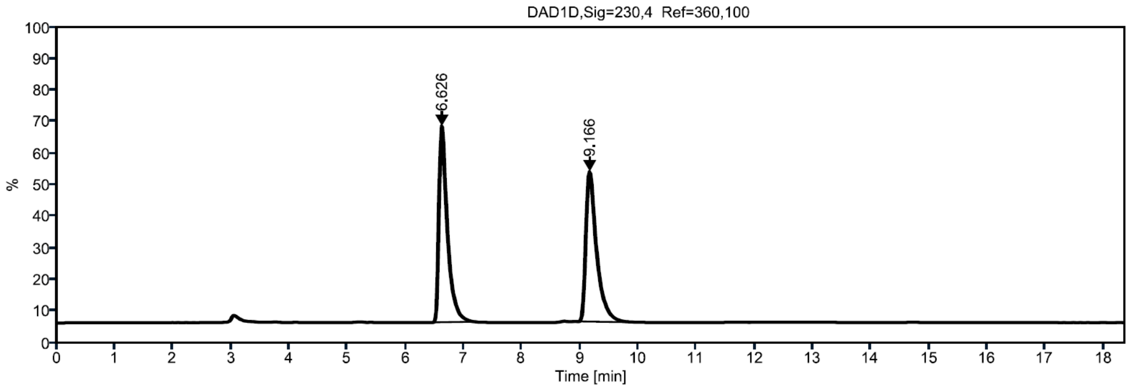

Signal: DAD1D,Sig=230,4 Ref=360,100

RT [min]	Type	Width [min]	Area	Height	Area%
6.626	MM m	0.15	1667.44	165.18	50.47
9.166	MM m	0.19	1636.07	126.37	49.53
		Sum	3303.51		

Cyclization of chiral substrate

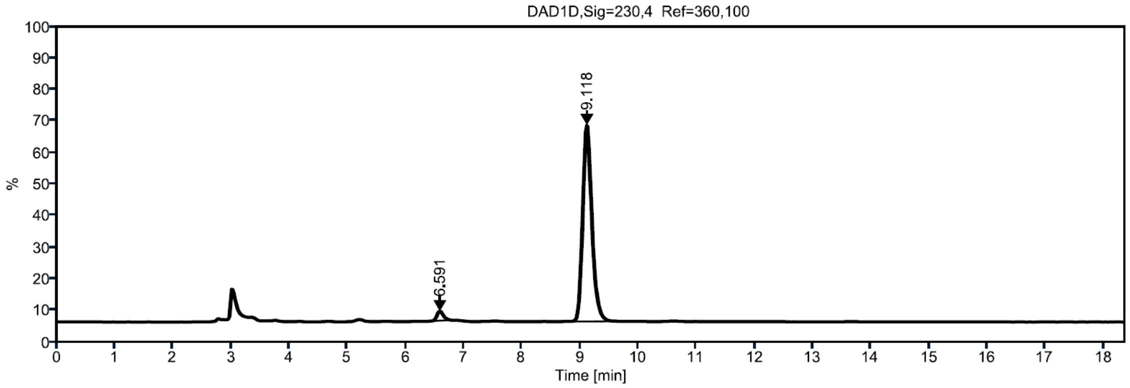

Signal: DAD1D,Sig=230,4 Ref=360,100

RT [min]	Type	Width [min]	Area	Height	Area%
6.591	MM m	0.12	44.20	5.61	3.31
9.118	MM m	0.17	1291.08	116.22	96.69
		Sum	1335.28		

Fig. 4, Compound 15

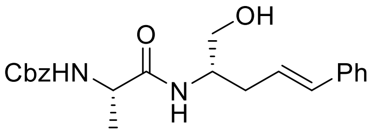

HPLC Conditions

Column: Chiralpak AD-H, Daicel Chemical Industries, Ltd.

Eluent: Hexanes/Isopropanol (80:20)

Flow rate: 1.0 mL/min

Detection: UV 254 nm

Condensation with racemic amine

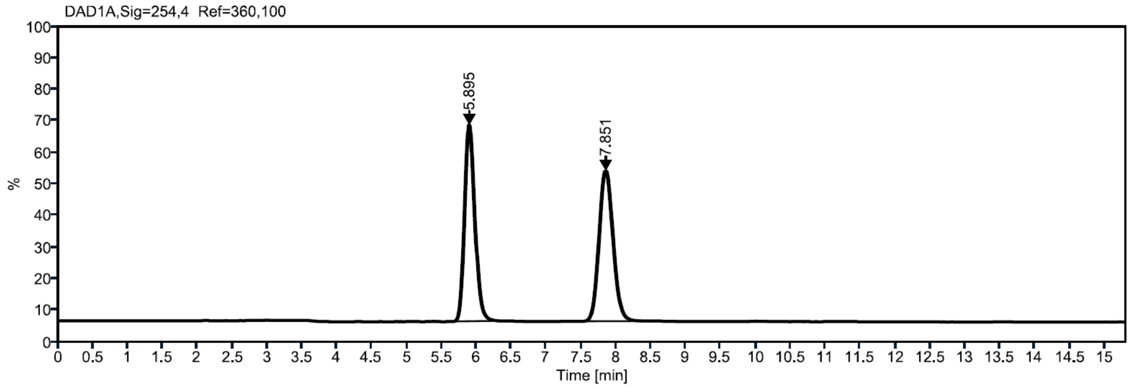

Signal: DAD1A,Sig=254,4 Ref=360,100

RT [min]	Type	Width [min]	Area	Height	Area%
5.895	MM m	0.16	896.98	86.56	49.42
7.851	MM m	0.21	918.12	66.26	50.58
		Sum	1815.10		

Condensation with chiral amine

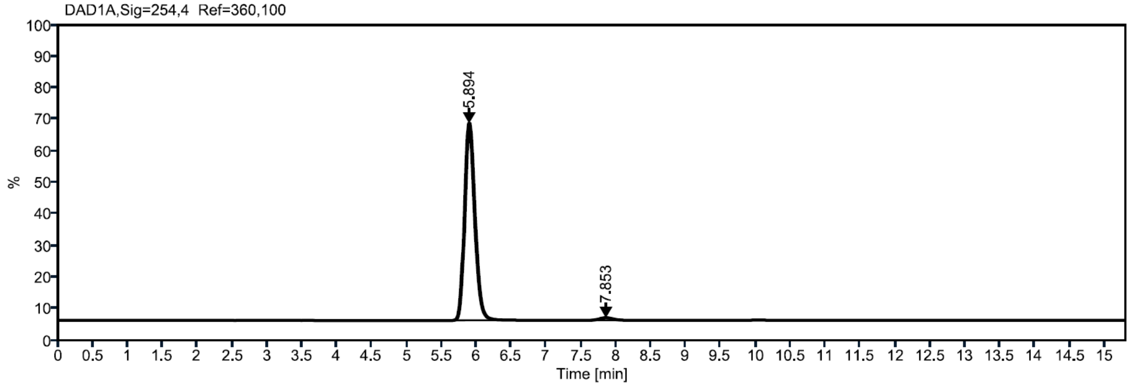

Signal: DAD1A,Sig=254,4 Ref=360,100

RT [min]	Type	Width [min]	Area	Height	Area%
5.894	MM m	0.16	2137.13	208.08	98.42
7.853	MM m	0.21	34.41	2.64	1.58
		Sum	2171.54		

4. Supplementary References

1. Chen, J., *et al.* Carbonyl catalysis enables a biomimetic asymmetric Mannich reaction. *Science* **360**, 1438-1442 (2018).
2. Trost, B. M., Silverman, S. M. & Stambuli, J. P. Development of an Asymmetric Trimethylenemethane Cycloaddition Reaction: Application in the Enantioselective Synthesis of Highly Substituted Carbocycles. *J. Am. Chem. Soc.* **133**, 19483-19497 (2011).
3. Shi, W.-J., Wang, L.-X., Fu, Y., Zhu, S.-F. & Zhou, Q.-L. Highly regioselective asymmetric copper-catalyzed allylic alkylation with dialkylzincs using monodentate chiral spiro phosphoramidite and phosphite ligands. *Tetrahedron: Asymmetry* **14**, 3867-3872 (2003).
4. Leitner, A., Shekhar, S., Pouy, M. J. & Hartwig, J. F. A Simple Iridium Catalyst with a Single Resolved Stereocenter for Enantioselective Allylic Amination. Catalyst Selection from Mechanistic Analysis. *J. Am. Chem. Soc.* **127**, 15506-15514 (2005).
5. Rodríguez, J., *et al.* Lanesoic Acid: A Cytotoxic Zwitterion from *Theonella sp*. *Org. Lett.* **18**, 5832-5835 (2016).
6. Tyski, S., Markiewicz, M., Gulewicz, K. & Twardowski, T. The Effect of Lupin Alkaloids and Ethanol Extracts from Seeds of *Lupinus angustifolius* on Selected Bacterial Strains. *J. Plant Physiol.* **133**, 240-242 (1988).
7. Daly, J. W., *et al.* Histrionicotoxins: Roentgen-Ray Analysis of the Novel Allenic and Acetylenic Spiroalkaloids Isolated from a Colombian Frog, Dendrobates histrionicus. *Proc. Nat. Acad. Sci. USA* **68**, 1870-1875 (1971).
8. Kanoh, N., *et al.* Design, Synthesis, and Antifungal Activity of 16,17-Dihydroheronamide C and *ent*-Heronamide C. *J. Org. Chem.* **86**, 16249-16258 (2021).
9. Villard, V., Meunier, J., Chevallier, N. & Maurice, T. Pharmacological Interaction With the Sigma$_1$ (σ_1)-Receptor in the Acute Behavioral Effects of Antidepressants. *J. Pharmacol. Sci.* **115**, 279-292 (2011).
10. Sugawara, K., *et al.* Eponemycin, a new antibiotic active against B16 melanoma. I. Production, isolation, structure and biological activity. *J. Antibiot.* **43**, 8-18 (1990).
11. Hayden, F. G., *et al.* Efficacy and Safety of the Neuraminidase Inhibitor Zanamivir in the Treatment of Influenzavirus Infections. *New Engl. J. Med.* **337**, 874-880 (1997).
12. Brostrom, L. & Falck, J. R. Arachidonic acid analogs and methods for analgesic treatment using same. Patent WO2011066414 (2011).
13. Decker, A. M., Partilla, J. S., Baumann, M. H., Rothman, R. B. & Blough, B. E. The biogenic amine transporter activity of vinylogous amphetamine analogs. *Med. Chem. Commun.* **7**, 1657-1663 (2016).
14. Dunbar, G. C., *et al.* Effect of ispronicline, a neuronal nicotinic acetylcholine receptor partial agonist, in subjects with age associated memory impairment (AAMI). *J. Psychopharmacol.* **21**, 171-178 (2007).
15. Ito, Y., *et al.* Structural Requirements of Sphingosine Molecules for Inhibition of DNA Primase: Biochemical and Computational Analyses. *Biochemistry* **40**, 11571-11577 (2001).

16. Lampe, J. W., *et al.* Cardiotonic agents. 6. Histamine analogs as potential cardiovascular selective H_2 agonists. *J. Med. Chem.* **33**, 1688-1697 (1990).

17. Raub, M. F., Cardellina, J. H. & Spande, T. F. The piclavines, antimicrobial indolizidines from the tunicate Clavelina picta. *Tetrahedron Lett.* **33**, 2257-2260 (1992).

18. Barrow, R. A., Moore, R. E., Li, L.-H. & Tius, M. A. Synthesis of 1-Aza-cryptophycin 1, an Unstable Cryptophycin. An Unusual Skeletal Rearrangement. *Tetrahedron* **56**, 3339-3351 (2000).

19. Groll, M., *et al.* A plant pathogen virulence factor inhibits the eukaryotic proteasome by a novel mechanism. *Nature* **452**, 755-758 (2008).

20. Pei, Z., *et al.* Pharmaceutical compositions as inhibitors of dipeptidyl peptidase-IV (DPP-IV). Patent US20070049596 (2007).

21. Borzilleri, R. M., *et al.* A Novel Application of a Pd(0)-Catalyzed Nucleophilic Substitution Reaction to the Regio- and Stereoselective Synthesis of Lactam Analogues of the Epothilone Natural Products. *J. Am. Chem. Soc.* **122**, 8890-8897 (2000).

22. Qu, F. & Mantlo, N. B. Pyrazole compounds as SGLT1 inhibitors. Patent WO2013169546 (2013).

23. Clark, T. J. Five-membered heteroaromatic olefinic azacyclic compounds, pharmaceutical compositions containing them and their use as inhibitors of nicotinic cholinergic receptors. Patent WO2004009599 (2004).

24. Hansen, B. S., *et al.* Pharmacological characterisation of a new oral GH secretagogue, NN703. *Eur. J. Endocrinol.* **141**, 180-189 (1999).

25. Igarashi, T., Aritake, S. & Yasumoto, T. Biological activities of prymnesin-2 isolated from a red tide alga *Prymnesium parvum*. *Nat. Toxins* **6**, 35-41 (1998).

26. Huo, X., Zhang, J., Fu, J., He, R. & Zhang, W. Ir/Cu Dual Catalysis: Enantio- and Diastereodivergent Access to α,α-Disubstituted α-Amino Acids Bearing Vicinal Stereocenters. *J. Am. Chem. Soc.* **140**, 2080-2084 (2018).

27. Dolomanov, O. V., Bourhis, L. J., Gildea, R. J., Howard, J. A. K. & Puschmann, H. *OLEX2*: a complete structure solution, refinement and analysis program. *J. Appl. Cryst.* **42**, 339-341 (2009).

28. Sheldrick, G. M. *SHELXT* - Integrated space-group and crystal-structure determination. *Acta Cryst. A* **71**, 3-8 (2015).

29. Sheldrick, G. M. Crystal structure refinement with *SHELXL*. *Acta Cryst, C* **71**, 3-8 (2015).

30. Sharpless, K. B., *et al.* The osmium-catalyzed asymmetric dihydroxylation: a new ligand class and a process improvement. *J. Org. Chem.* **57**, 2768-2771 (1992).

31. Reddy, L. R., Hu, B., Prashad, M. & Prasad, K. Asymmetric Synthesis of Homoallylic Amines Bearing Adjacent Stereogenic Centers by Addition of Substituted Allylic Zinc Reagents to N-tert-Butanesulfinylimines. *Org. Lett.* **10**, 3109-3112 (2008).

32. Peng, Y., Huo, X., Luo, Y., Wu, L. & Zhang, W. Enantio- and Diastereodivergent Synthesis of Spirocycles through Dual-Metal-Catalyzed [3+2] Annulation of 2-Vinyloxiranes with Nucleophilic Dipoles. *Angew. Chem. Int. Ed.* **60**, 24941-24949 (2021).

33. Frisch, M. J. *et al*. Gaussian 09, Revision A.02, Gaussian, Inc. (Wallingford, CT, 2009).

34. Becke, A. D. Density-functional thermochemistry. III. The role of exact exchange. *J. Chem. Phys.* **98**, 5648-5652 (1993).

35. Zhao, Y. & Truhlar, D. G. The M06 suite of density functionals for main group thermochemistry, thermochemical kinetics, noncovalent interactions, excited states, and transition elements: two new functionals and systematic testing of four M06-class functionals and 12 other functionals. *Theor. Chem. Acc.* **120**, 215-241 (2008).

36. Marenich, A. V., Cramer, C. J. & Truhlar, D. G. Universal Solvation Model Based on Solute Electron Density and on a Continuum Model of the Solvent Defined by the Bulk Dielectric Constant and Atomic Surface Tensions. *J. Phys. Chem. B* **113**, 6378-6396 (2009).

37. Lu, T. & Chen, F. Multiwfn: A multifunctional wavefunction analyzer. *J. Comput. Chem.* **33**, 580-592 (2012).

38. Yang, C., Xue, X.-S., Jin, J.-L., Li, X. & Cheng, J.-P. Theoretical Study on the Acidities of Chiral Phosphoric Acids in Dimethyl Sulfoxide: Hints for Organocatalysis. *J. Org. Chem.* **78**, 7076-7085 (2013).

39. Bordwell, F. G. & Lynch, T. Y. Radical stabilization energies and synergistic (captodative) effects. *J. Am. Chem. Soc.* **111**, 7558-7562 (1989).
