# Supplementary material for: Asymmetric α-C(sp3)−H allylic alkylation of primary alkylamines by synergistic Ir/ketone catalysis
Source: Nat Commun. 2024 Jan 31;15:939. doi: 10.1038/s41467-024-45131-3 (PMC10830461; doi:10.1038/s41467-024-45131-3)
Supplement: Supplementary file 3 — Description of Additional Supplementary Files [file 41467_2024_45131_MOESM3_ESM.docx]

**Description of Additional Supplementary Files**

**File Name: Supplementary Data 1**

**Description:** Coordinates of the optimized structures for the computational studies.
